# Supplementary figures and images for: Convergence in Amino Acid Outsourcing Between Animals and Predatory Bacteria
Source: Int J Mol Sci. 2025 Mar 26;26(7):3024. doi: 10.3390/ijms26073024 (PMC11988736; doi:10.3390/ijms26073024)

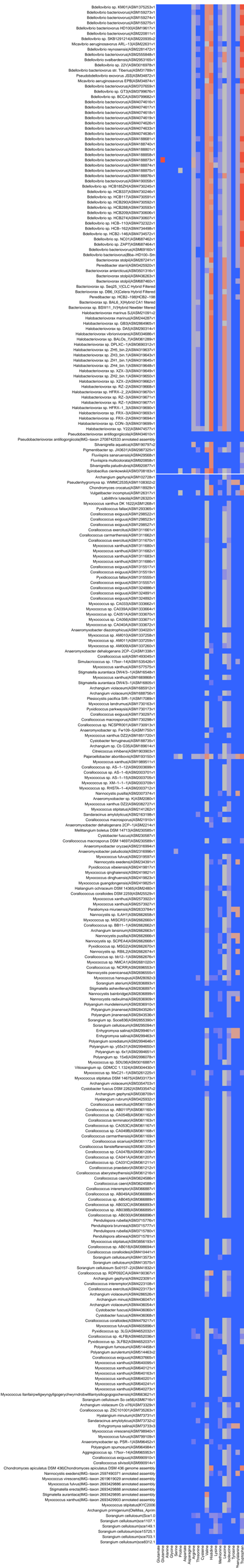

Supplement: Supplementary file 1 [file ijms-26-03024-s001.zip › File S1 Full heatmap.pdf]
