# Supplementary material for: Convergence in Amino Acid Outsourcing Between Animals and Predatory Bacteria
Source: Int J Mol Sci. 2025 Mar 26;26(7):3024. doi: 10.3390/ijms26073024 (PMC11988736; doi:10.3390/ijms26073024)
Supplement: Supplementary file 1 [file File S2 per-species statistics on binary encoded auxotrophies.pdf › File S2 per-species statistics on binary encoded auxotrophies.pdf]

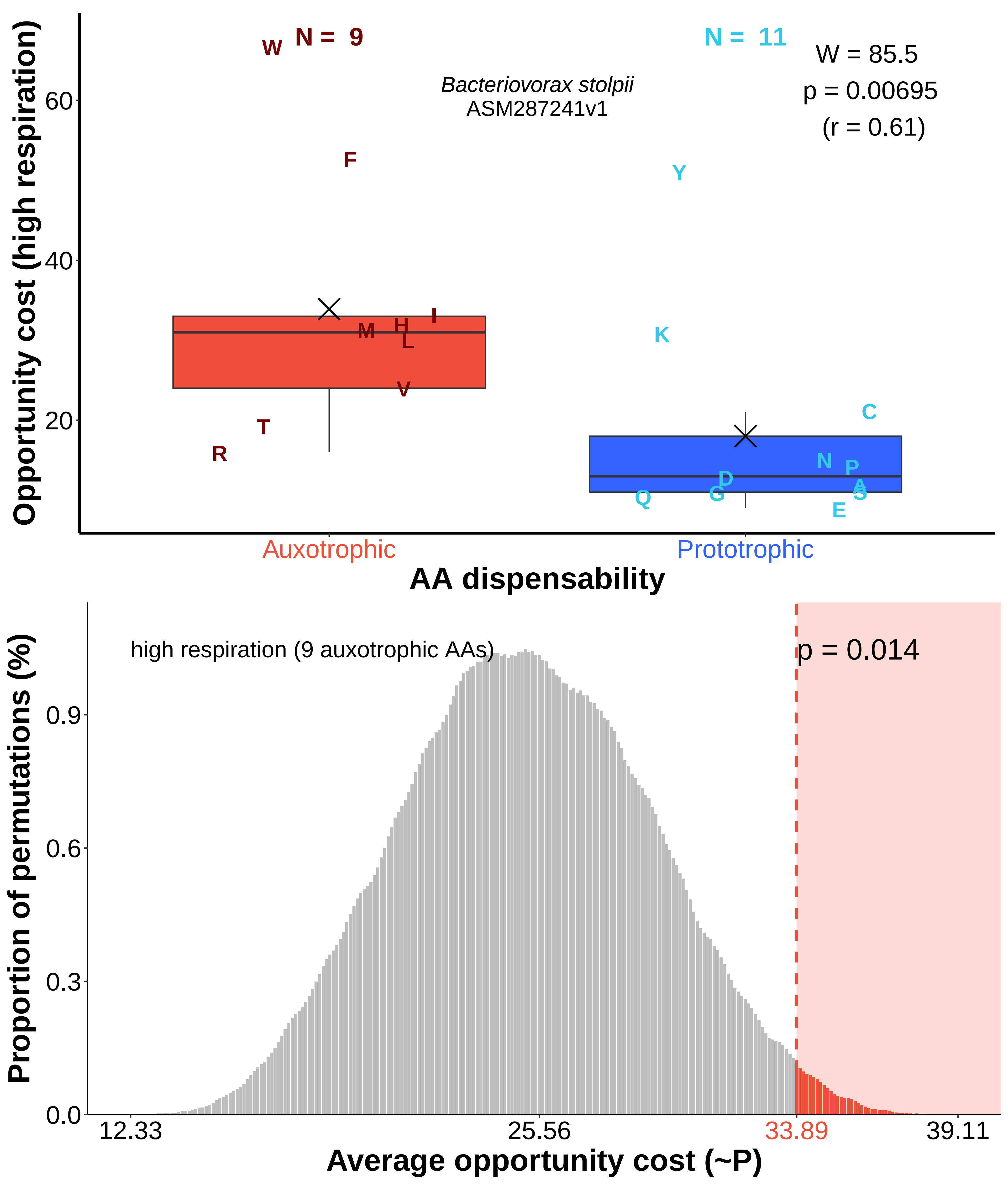

N = 9
N = 11
W = 85.5
p = 0.00695
(r = 0.61)
Bacteriovorax stolpii
ASM287241v1
W
F
Y
I
M
H
L
K
V
C
T
R
N
P
D
A
S
Q
G
E
Opportunity cost (high respiration)
60
40
20
Auxotrophic
Prototrophic
AA dispensability
high respiration (9 auxotrophic AAs)
p = 0.014
Proportion of permutations (%)
0.9
0.6
0.3
0.0
12.33
25.56
33.89
39.11
Average opportunity cost (~P)

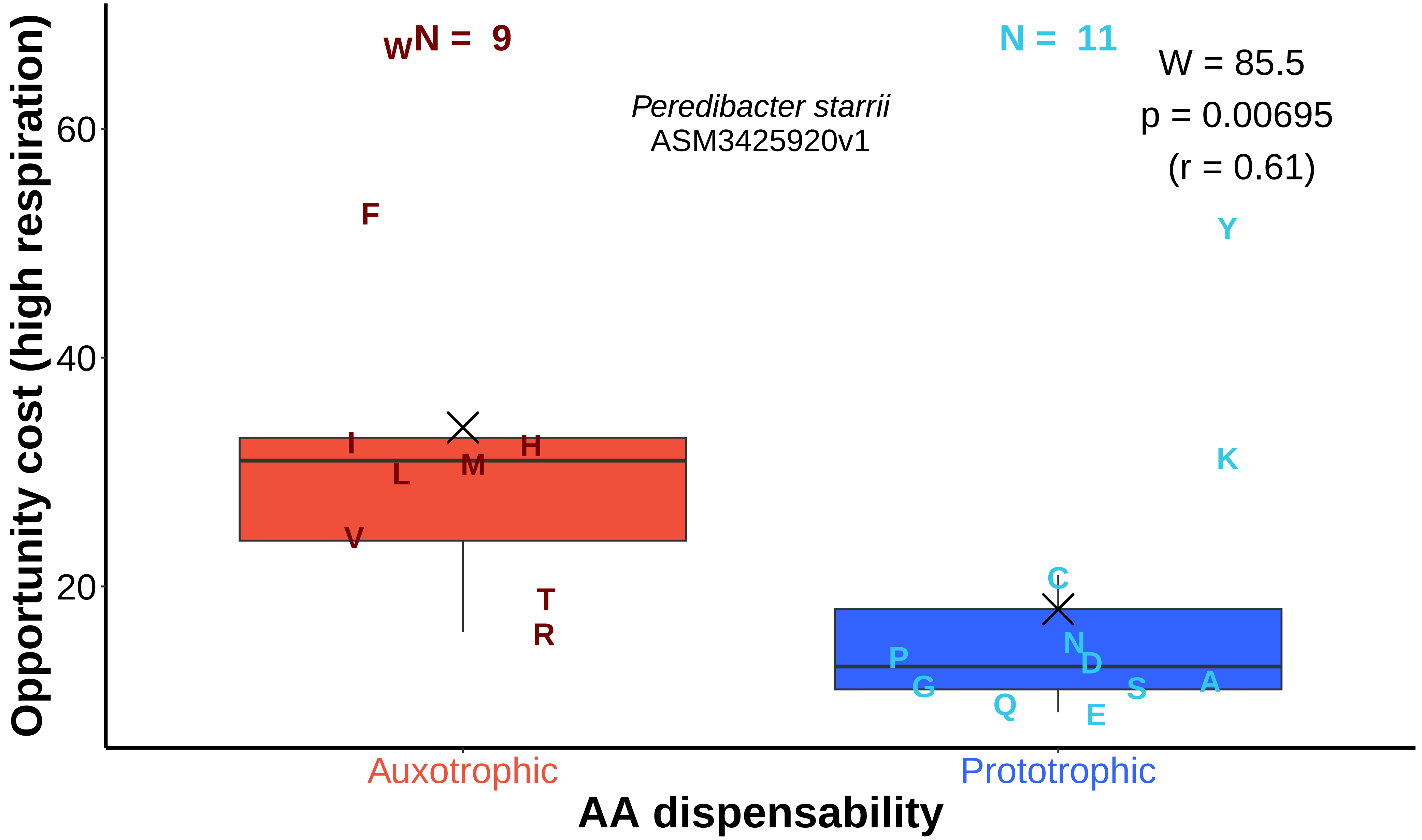

N = 9
N = 11
W = 85.5
p = 0.00695
(r = 0.61)
Peredibacter starrii
ASM3425920v1
Opportunity cost (high respiration)
60
40
20
F
I
H
M
L
V
T
R
Y
K
C
N
D
P
G
S
A
Q
E
Auxotrophic
Prototrophic
AA dispensability

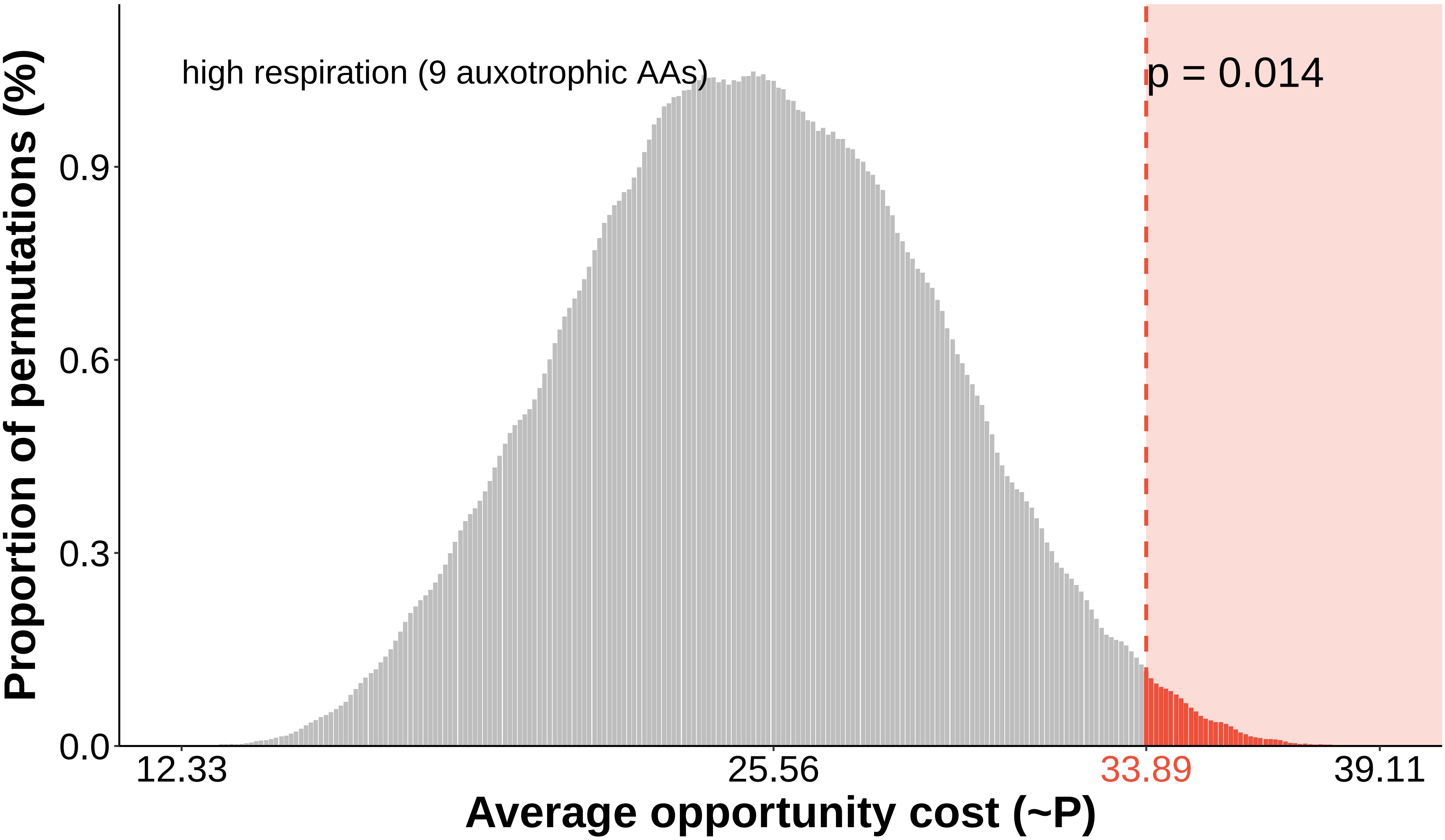

high respiration (9 auxotrophic AAs)
p = 0.014
Proportion of permutations (%)
0.9
0.6
0.3
0.0
12.33
25.56
33.89
39.11
Average opportunity cost (~P)

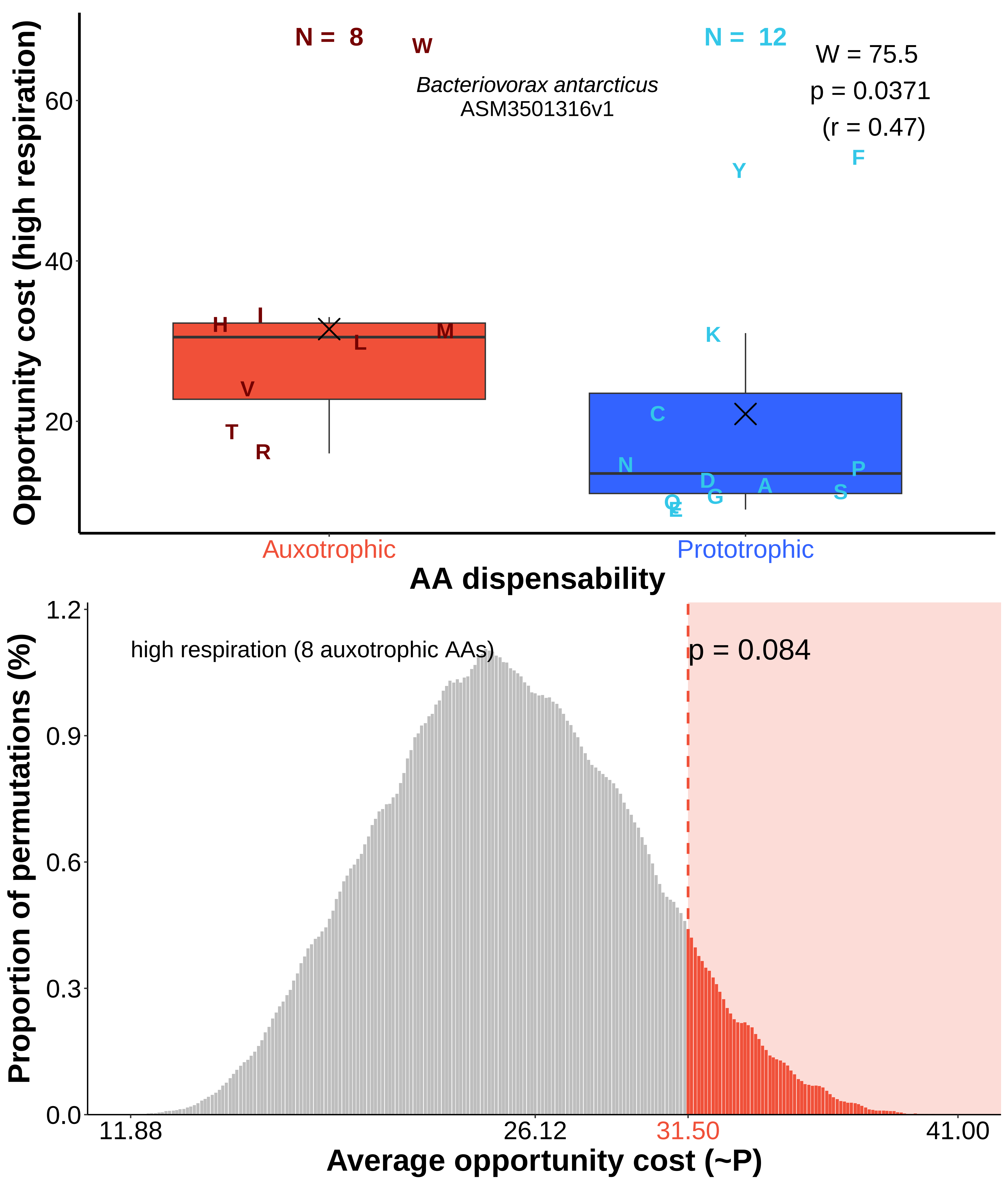
N = 8
N = 12
W = 75.5
p = 0.0371
(r = 0.47)
Bacteriovorax antarcticus
ASM3501316v1
W
Y
F
H
I
L
M
K
V
C
T
R
N
P
D
A
G
S
Q
E
Opportunity cost (high respiration)
60
40
20
Auxotrophic
Prototrophic
AA dispensability
high respiration (8 auxotrophic AAs)
p = 0.084
Proportion of permutations (%)
1.2
0.9
0.6
0.3
0.0
11.88
26.12
31.50
41.00
Average opportunity cost (~P)

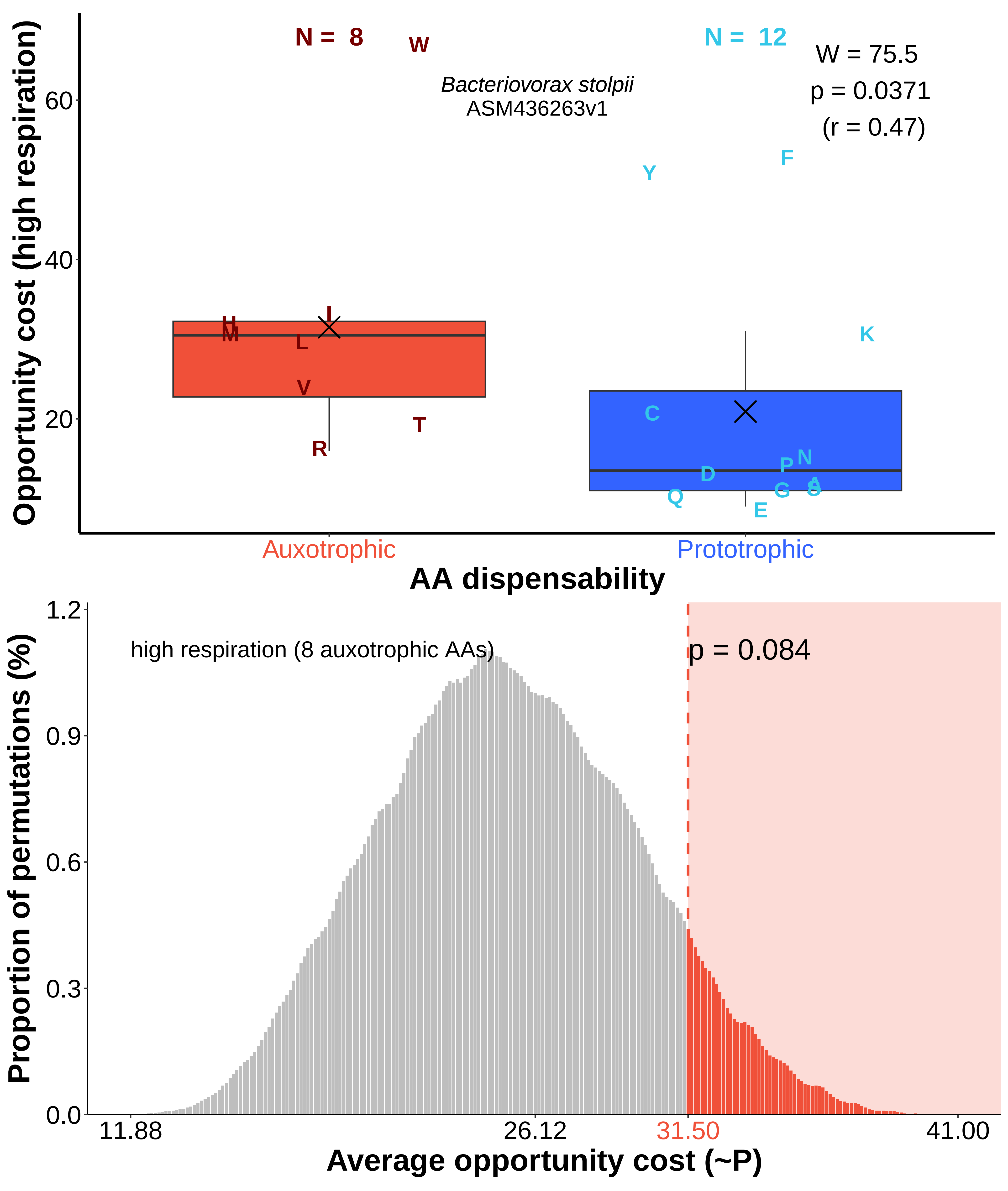

N = 8
W
N = 12
W = 75.5
p = 0.0371
(r = 0.47)
Bacteriovorax stolpii
ASM436263v1
Opportunity cost (high respiration)
60
40
20
Y
F
H
M
I
L
V
R
T
K
C
D
P
N
Q
G
A
S
E
Auxotrophic
Prototrophic
AA dispensability
1.2
0.9
0.6
0.3
0.0
high respiration (8 auxotrophic AAs)
p = 0.084
Proportion of permutations (%)
11.88
26.12
31.50
41.00
Average opportunity cost (~P)

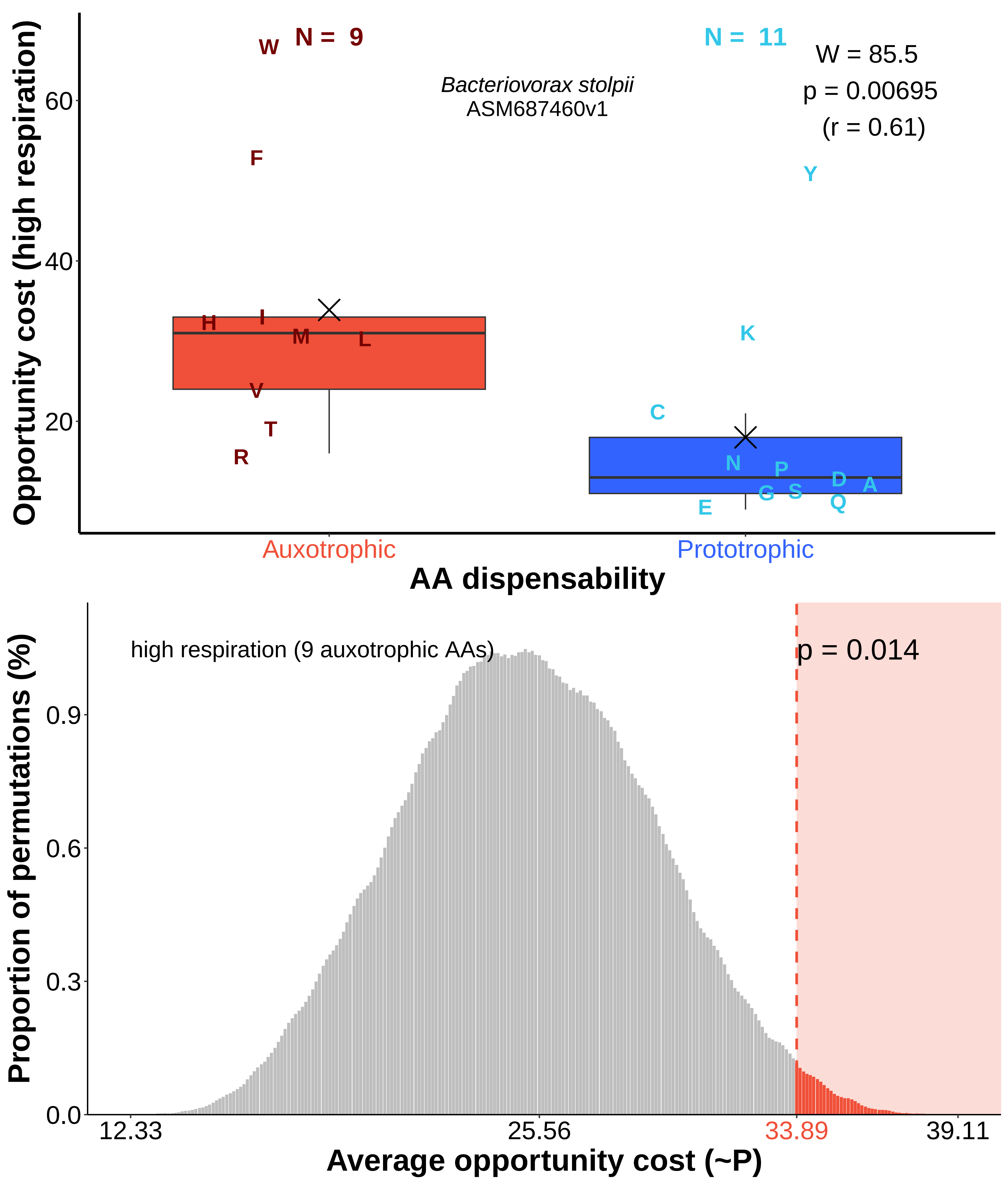

N = 9
N = 11
W = 85.5
p = 0.00695
(r = 0.61)
Bacteriovorax stolpii
ASM687460v1
W
F
H
I
M
L
V
T
R
Y
K
C
N
P
D
A
G
S
Q
E
Opportunity cost (high respiration)
60
40
20
Auxotrophic
Prototrophic
AA dispensability
high respiration (9 auxotrophic AAs)
p = 0.014
Proportion of permutations (%)
0.9
0.6
0.3
0.0
12.33
25.56
33.89
39.11
Average opportunity cost (~P)

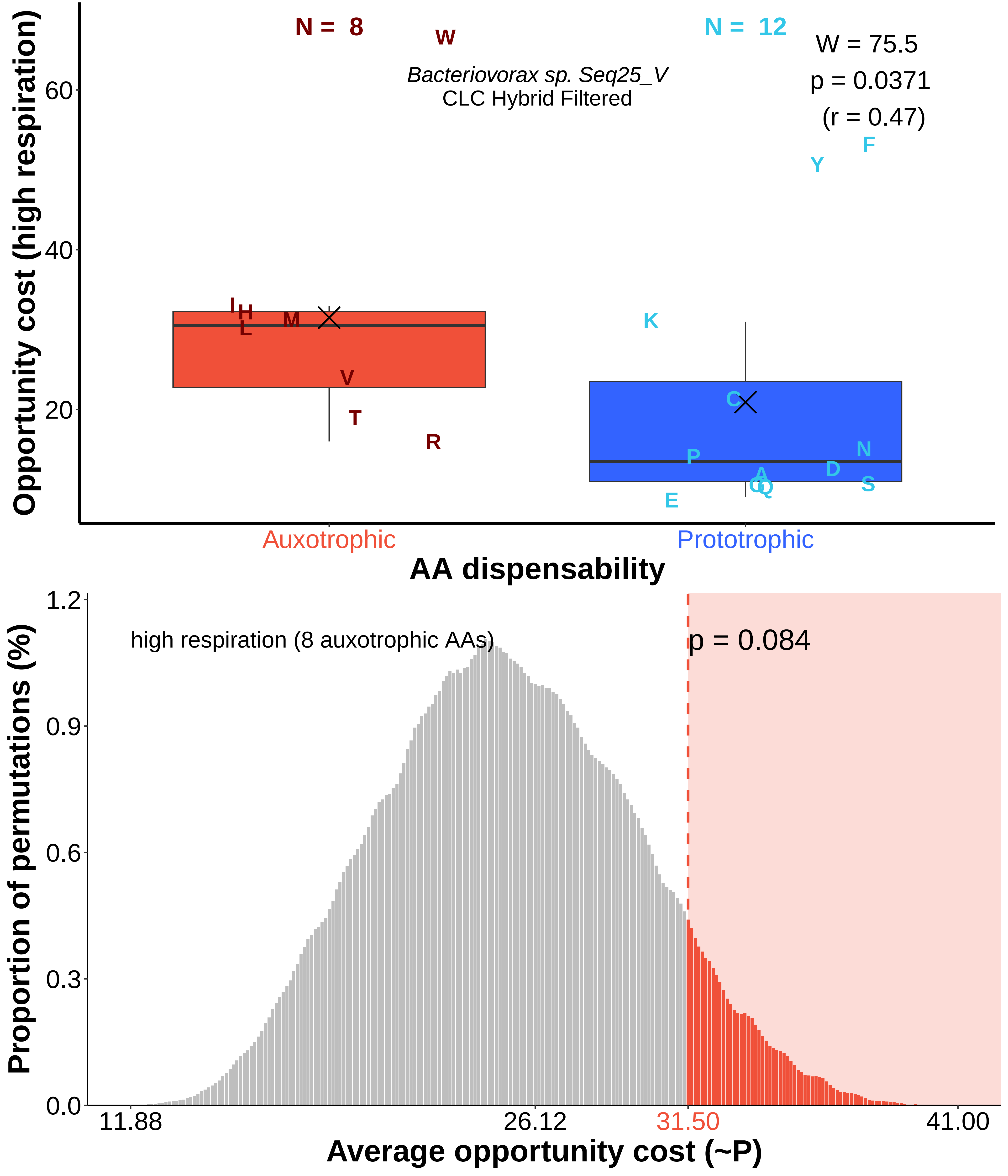

N = 8
N = 12
W = 75.5
p = 0.0371
(r = 0.47)
Bacteriovorax sp. Seq25_V
CLC Hybrid Filtered
Opportunity cost (high respiration)
Auxotrophic
Prototrophic
AA dispensability
high respiration (8 auxotrophic AAs)
p = 0.084
Proportion of permutations (%)
11.88
26.12
31.50
41.00
Average opportunity cost (~P)

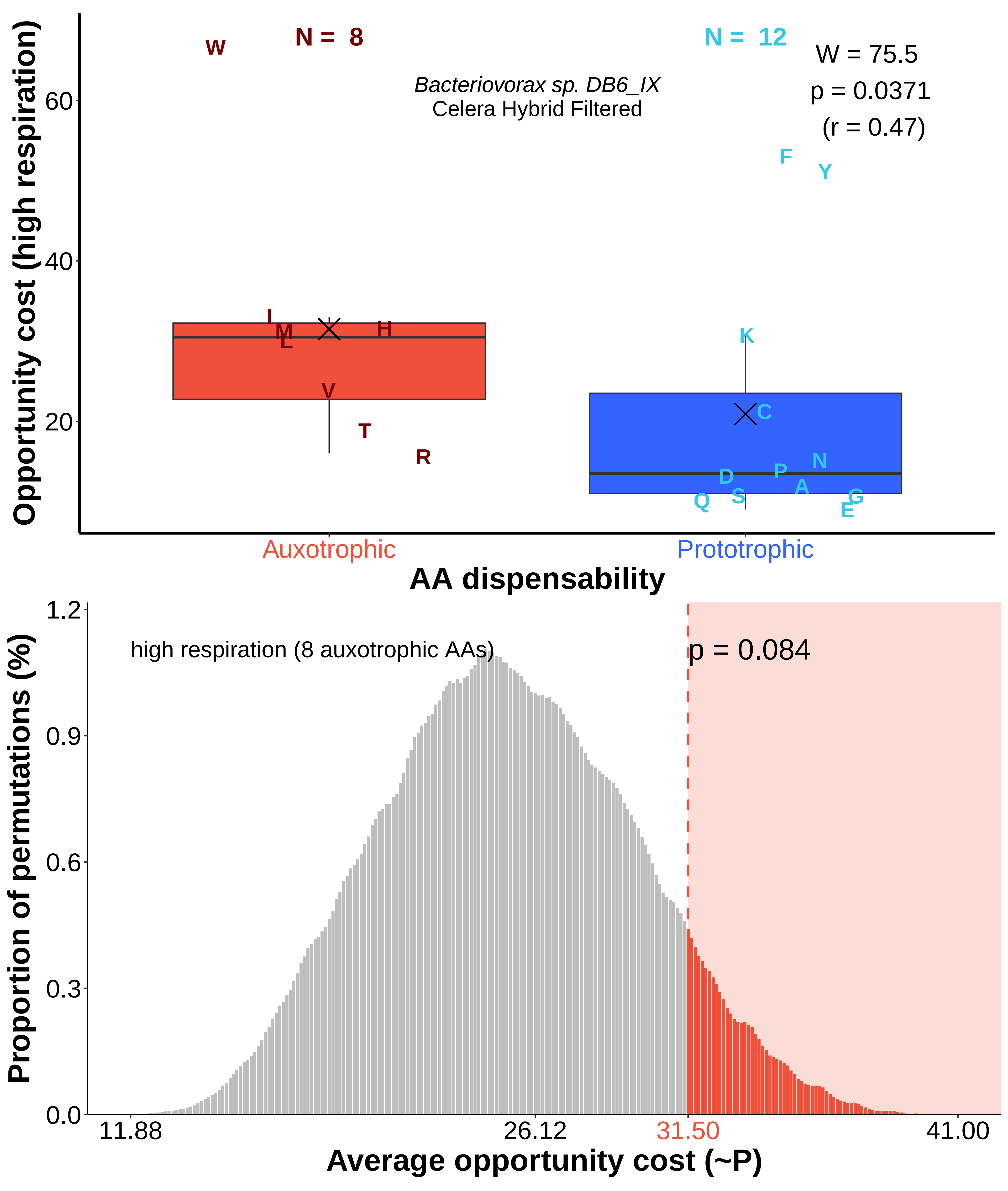

N = 8
N = 12
W = 75.5
p = 0.0371
(r = 0.47)
Bacteriovorax sp. DB6_IX
Celera Hybrid Filtered
Opportunity cost (high respiration)
60
40
20
W
I
M
L
H
V
T
R
F
Y
K
C
N
D
P
A
Q
S
G
E
Auxotrophic
Prototrophic
AA dispensability
1.2
0.9
0.6
0.3
0.0
high respiration (8 auxotrophic AAs)
p = 0.084
Proportion of permutations (%)
11.88
26.12
31.50
41.00
Average opportunity cost (~P)

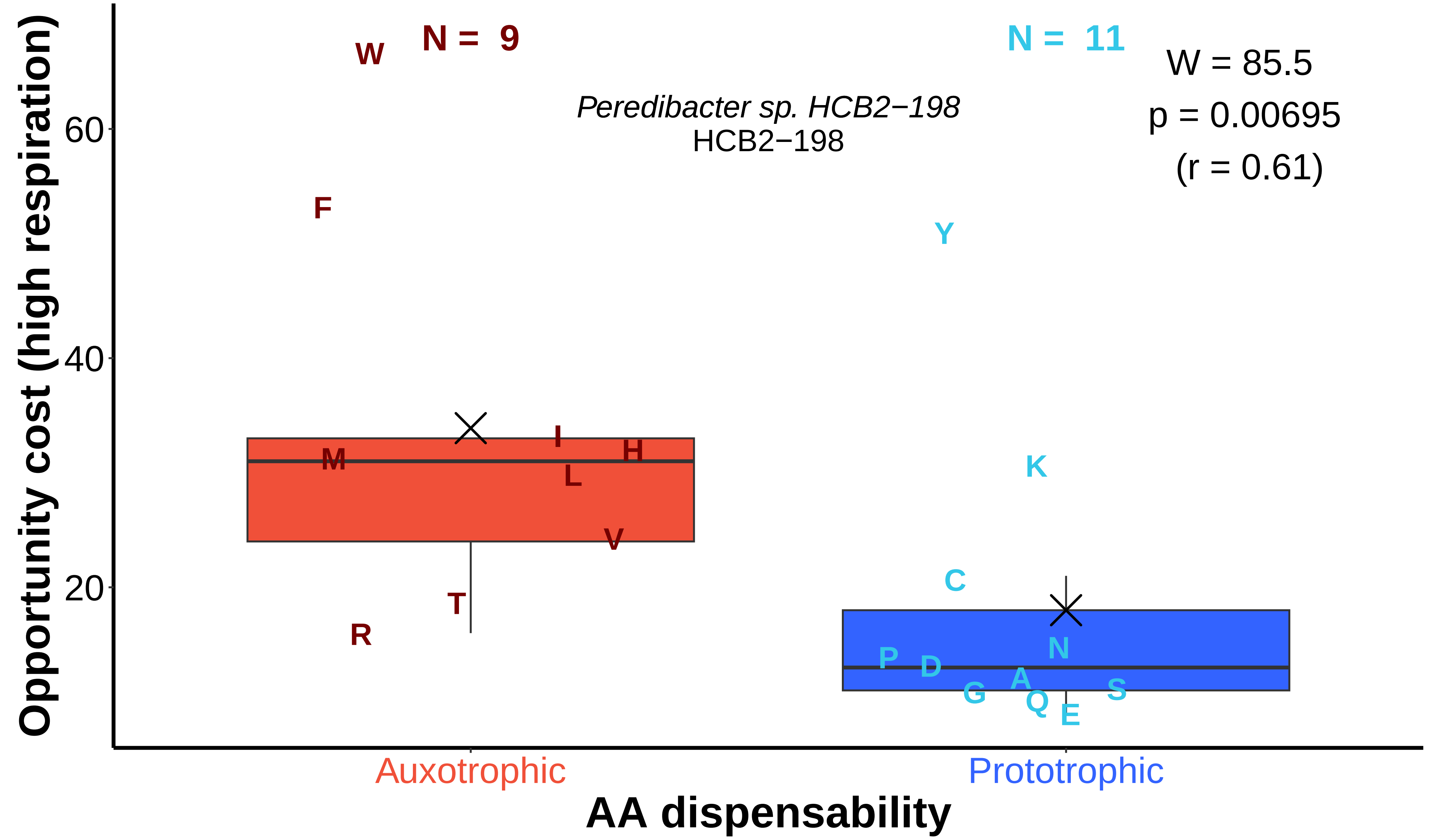

N = 9
N = 11
W = 85.5
p = 0.00695
(r = 0.61)
Peredibacter sp. HCB2−198
HCB2−198
Opportunity cost (high respiration)
60
40
20
W
F
M
I
H
L
V
T
R
Y
K
C
N
P
D
A
G
Q
S
E
Auxotrophic
Prototrophic
AA dispensability

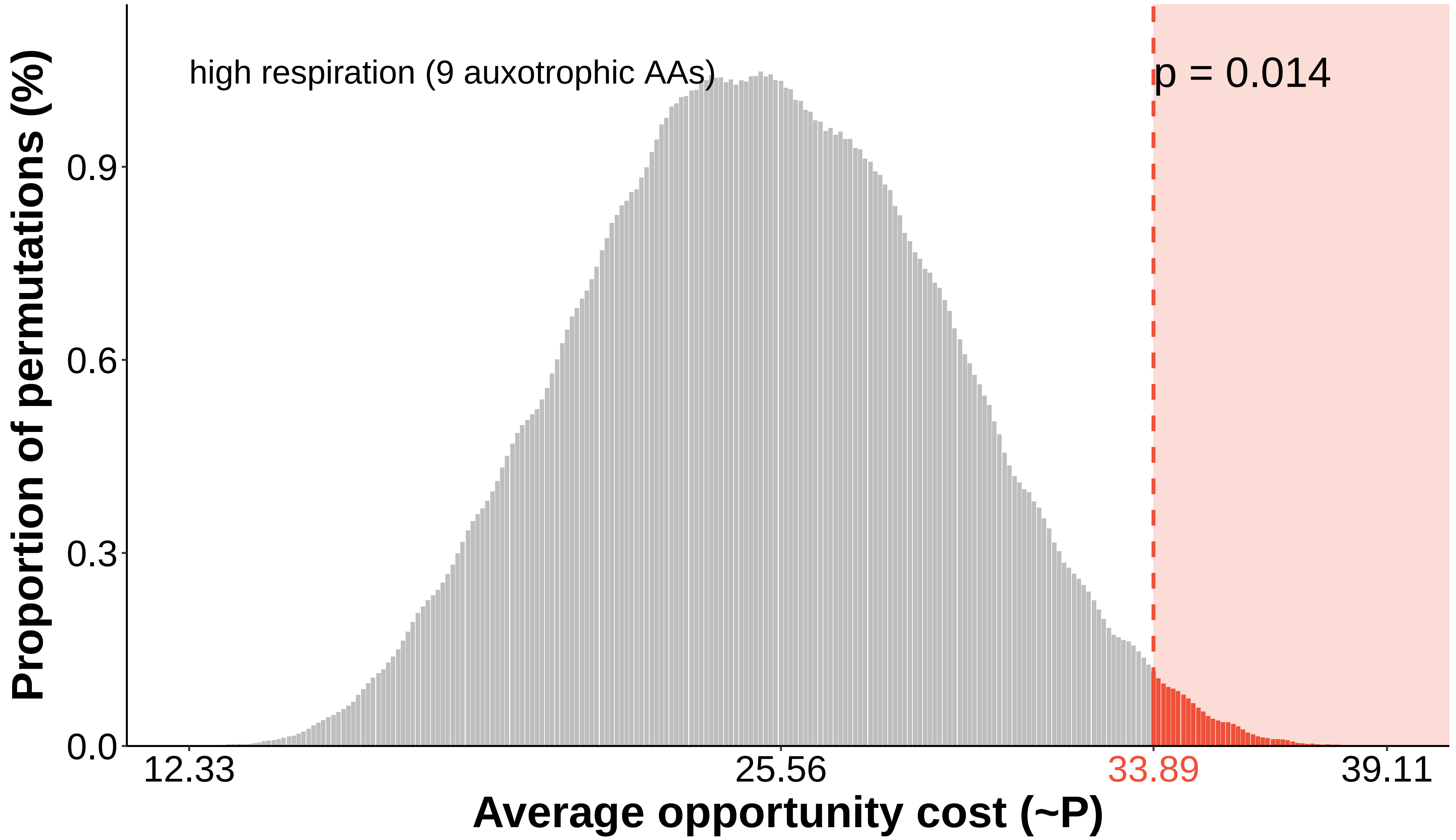

high respiration (9 auxotrophic AAs)
p = 0.014
Proportion of permutations (%)
0.9
0.6
0.3
0.0
12.33
25.56
33.89
39.11
Average opportunity cost (~P)

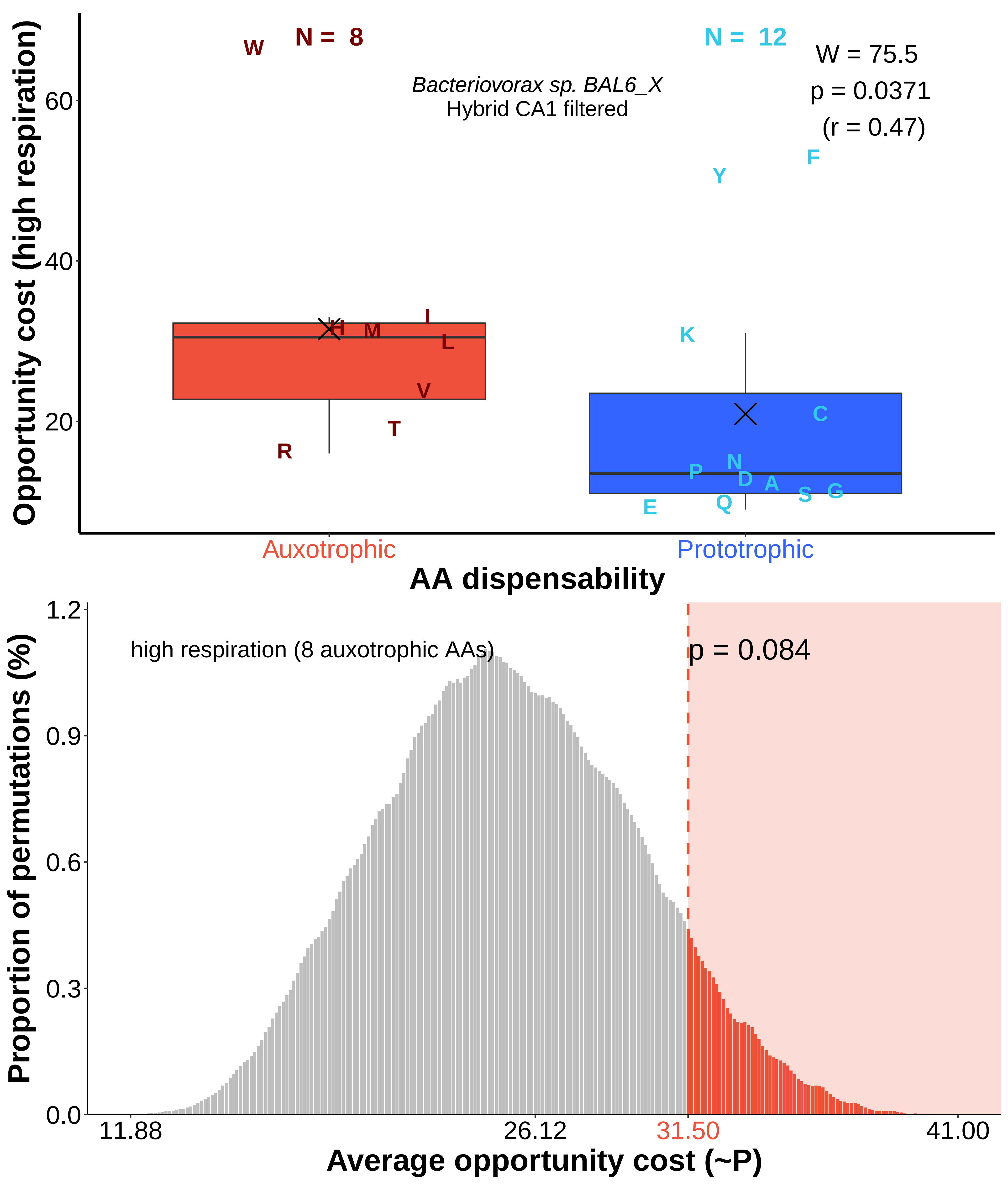
N = 8
N = 12
W = 75.5
p = 0.0371
(r = 0.47)
Bacteriovorax sp. BAL6_X
Hybrid CA1 filtered
Opportunity cost (high respiration)
W
I
H
M
L
V
T
R
K
F
Y
C
N
P
D
A
S
G
Q
E
Auxotrophic
Prototrophic
AA dispensability
60
40
20
Proportion of permutations (%)
high respiration (8 auxotrophic AAs)
p = 0.084
1.2
0.9
0.6
0.3
0.0
11.88
26.12
31.50
41.00
Average opportunity cost (~P)

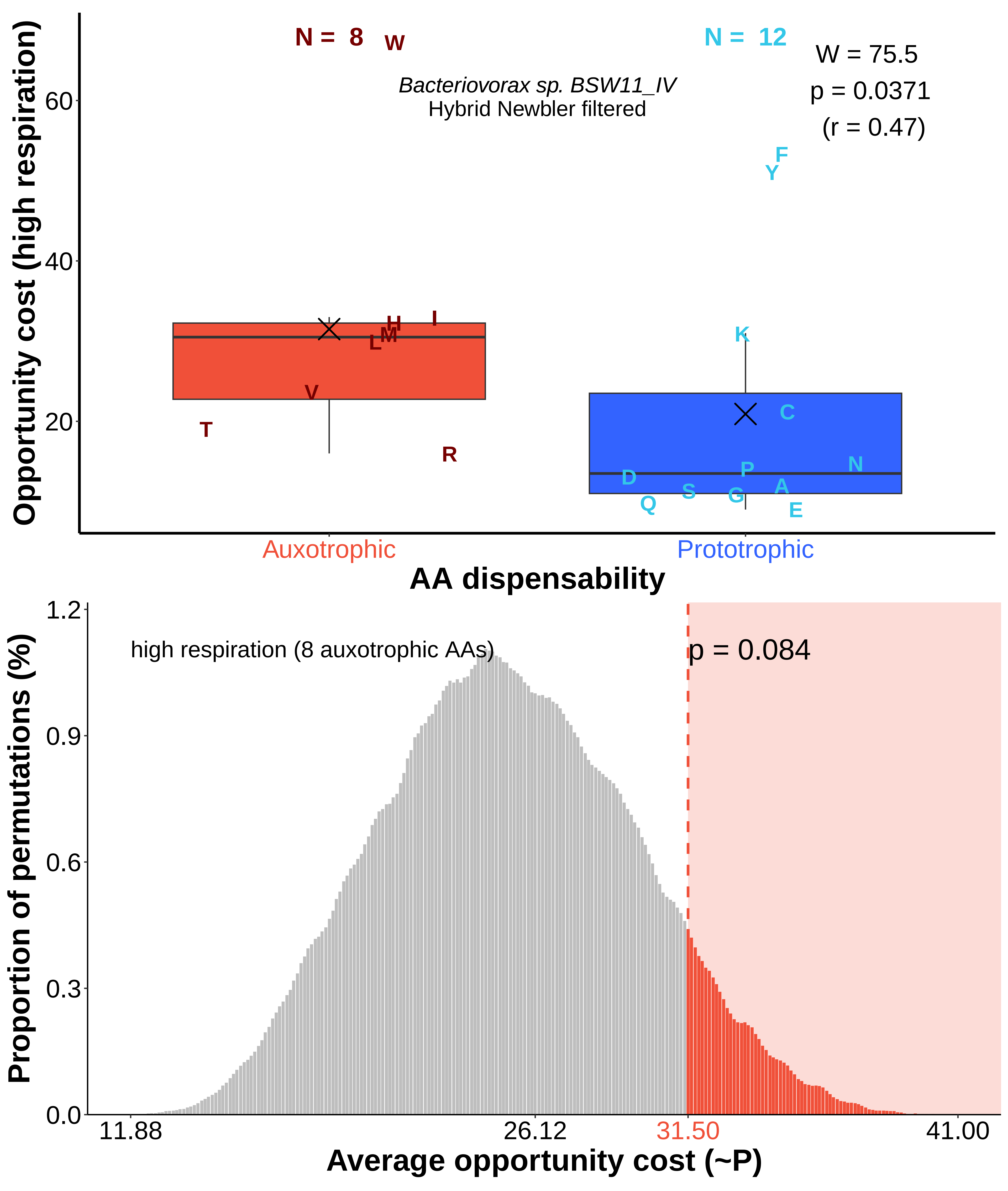

N = 8
W
N = 12
W = 75.5
p = 0.0371
(r = 0.47)
Bacteriovorax sp. BSW11_IV
Hybrid Newbler filtered
F
Y
I
H
L
M
K
V
C
T
R
N
D
P
S
A
G
Q
E
60
40
20
Opportunity cost (high respiration)
Auxotrophic
Prototrophic
AA dispensability
1.2
0.9
0.6
0.3
0.0
high respiration (8 auxotrophic AAs)
p = 0.084
Proportion of permutations (%)
11.88
26.12
31.50
41.00
Average opportunity cost (~P)

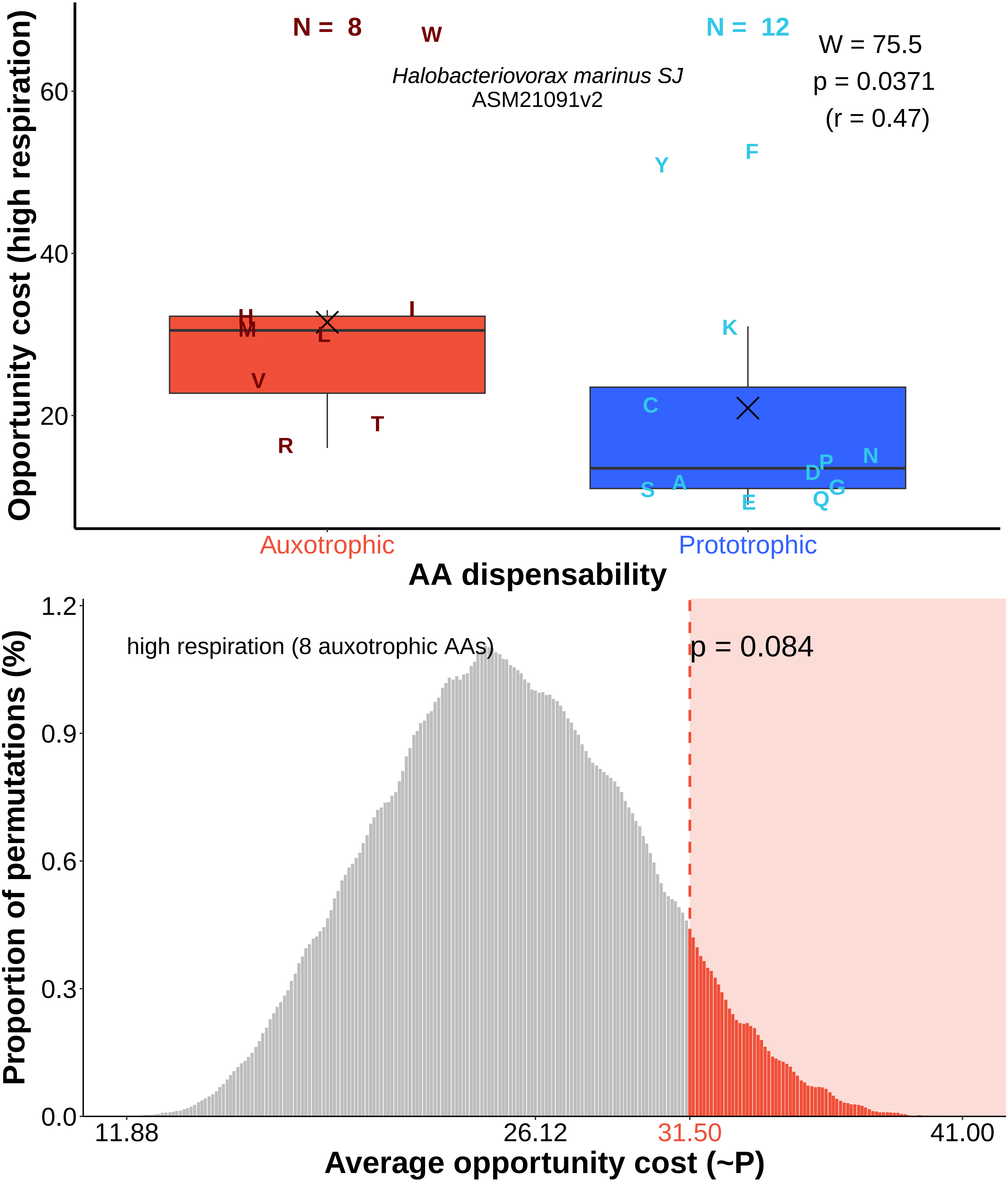

N = 8
W
N = 12
W = 75.5
p = 0.0371
(r = 0.47)
Halobacteriovorax marinus SJ
ASM21091v2
Y
F
H
M
I
L
K
V
C
T
R
P
N
D
A
S
G
Q
E
60
40
20
Opportunity cost (high respiration)
Auxotrophic
Prototrophic
AA dispensability
1.2
0.9
0.6
0.3
0.0
high respiration (8 auxotrophic AAs)
p = 0.084
Proportion of permutations (%)
11.88
26.12
31.50
41.00
Average opportunity cost (~P)

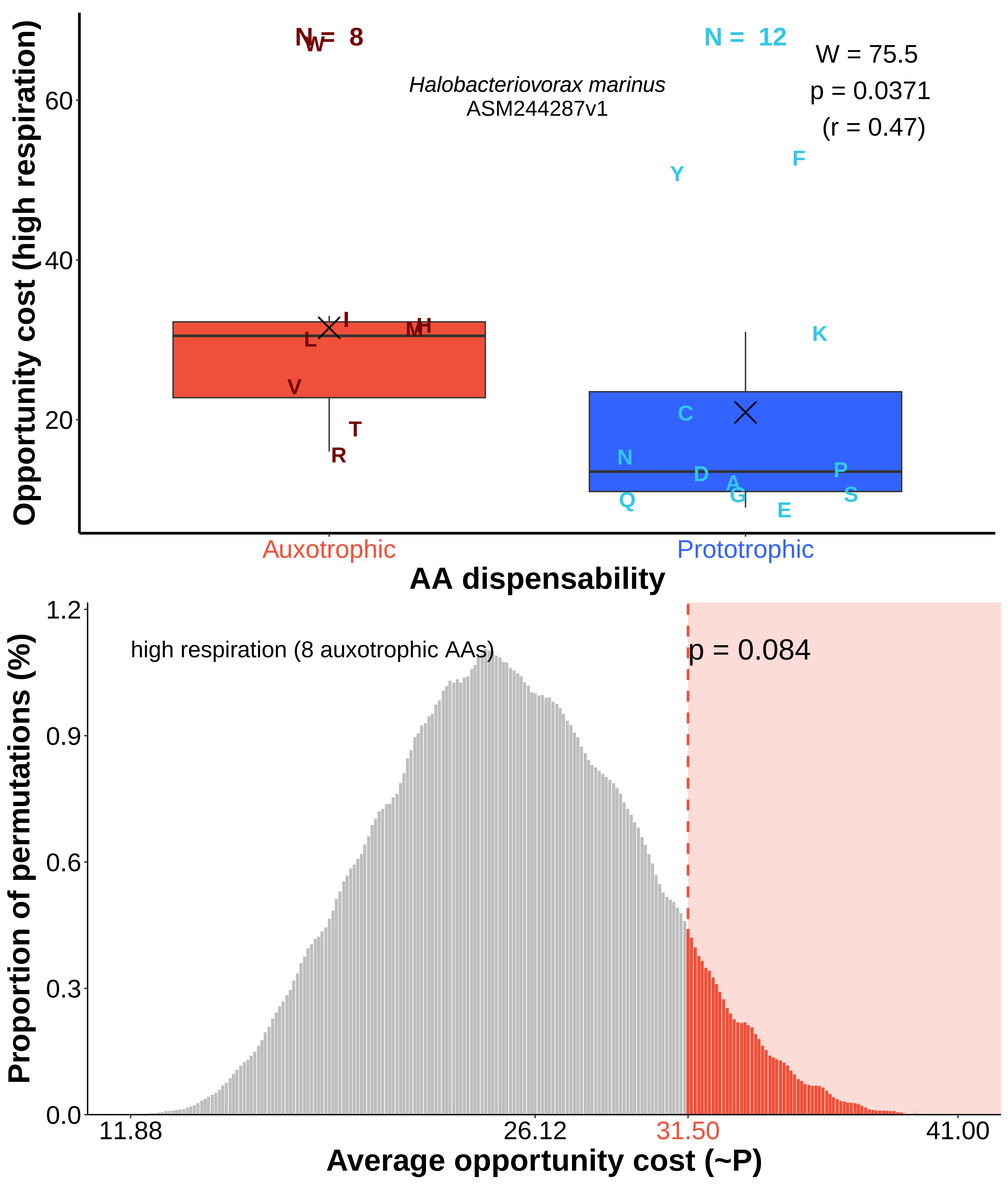

N = 8
N = 12
W = 75.5
p = 0.0371
(r = 0.47)
Halobacteriovorax marinus
ASM244287v1
Opportunity cost (high respiration)
Auxotrophic
Prototrophic
AA dispensability
high respiration (8 auxotrophic AAs)
p = 0.084
Proportion of permutations (%)
11.88
26.12
31.50
41.00
Average opportunity cost (~P)

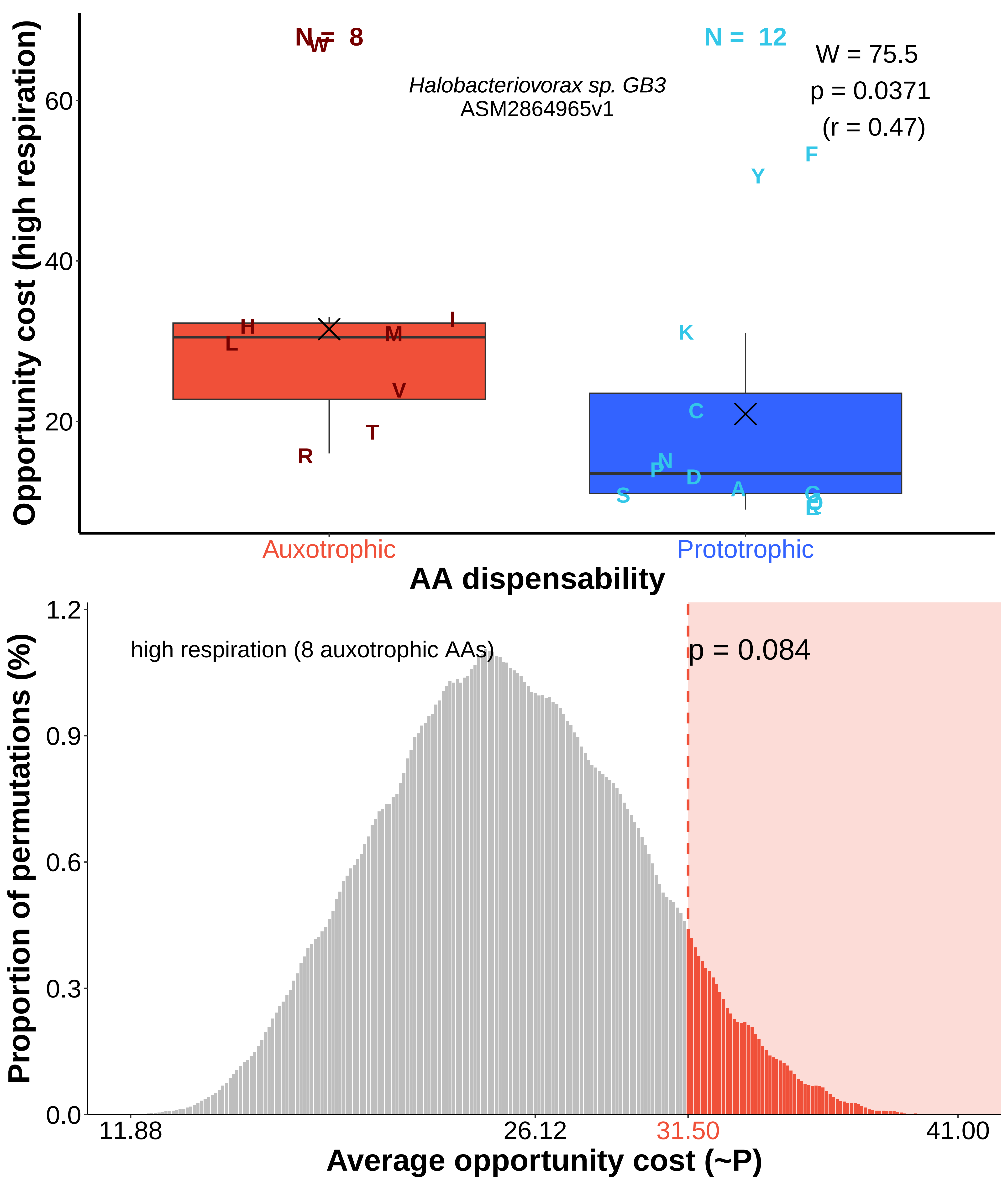
N = 8
W
N = 12
Halobacteriovorax sp. GB3
ASM2864965v1
W = 75.5
p = 0.0371
(r = 0.47)
Opportunity cost (high respiration)
60
40
20
F
Y
I
H
L
M
K
V
C
T
R
N
P
D
A
S
G
Q
E
Auxotrophic
Prototrophic
AA dispensability
Proportion of permutations (%)
1.2
0.9
0.6
0.3
0.0
high respiration (8 auxotrophic AAs)
p = 0.084
11.88
26.12
31.50
41.00
Average opportunity cost (~P)

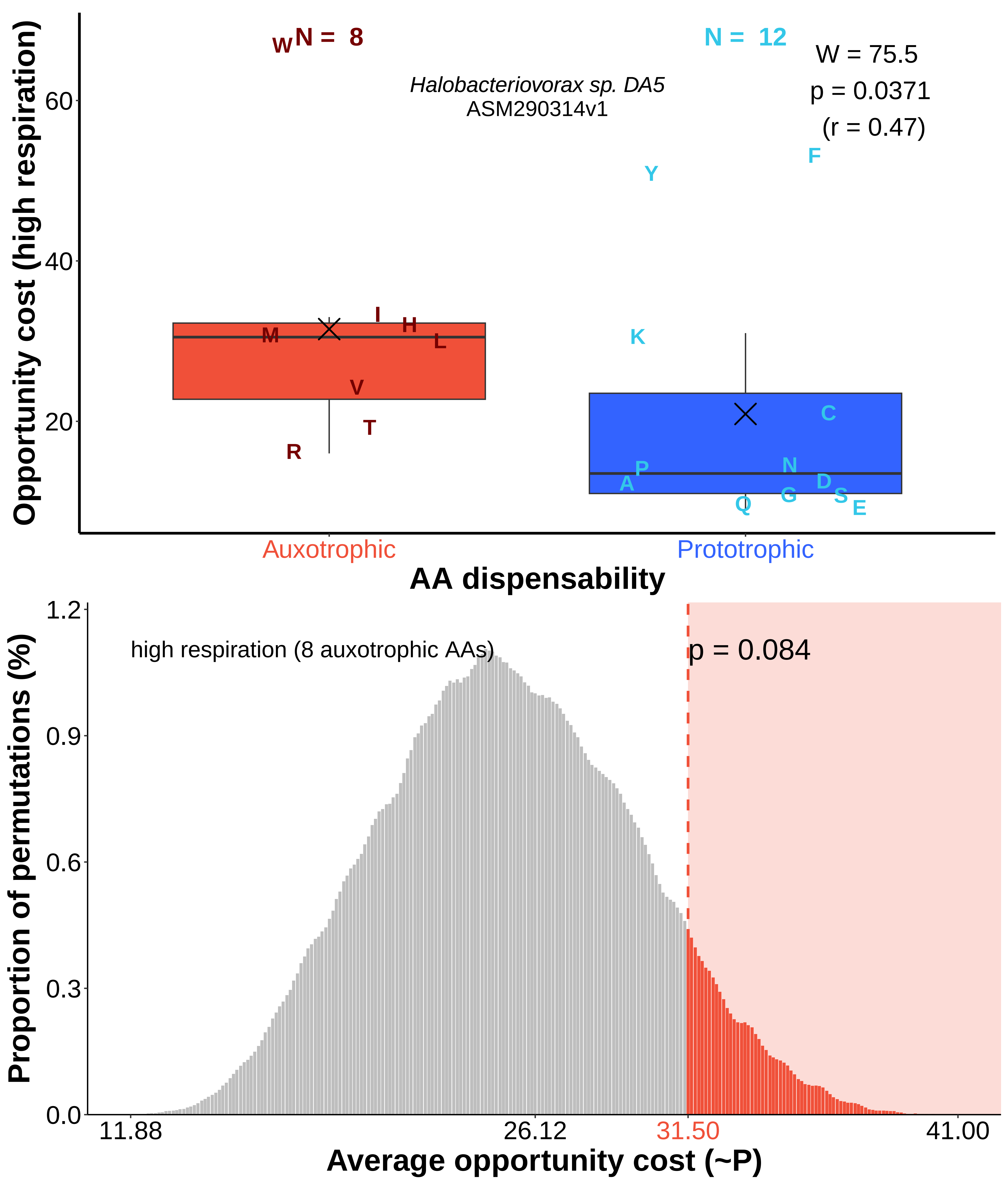

W
N = 8
N = 12
W = 75.5
p = 0.0371
(r = 0.47)
Halobacteriovorax sp. DA5
ASM290314v1
Opportunity cost (high respiration)
60
40
20
I
H
M
L
V
T
R
F
Y
K
C
P
N
A
D
G
S
Q
E
Auxotrophic
Prototrophic
AA dispensability
Proportion of permutations (%)
1.2
0.9
0.6
0.3
0.0
high respiration (8 auxotrophic AAs)
p = 0.084
11.88
26.12
31.50
41.00
Average opportunity cost (~P)

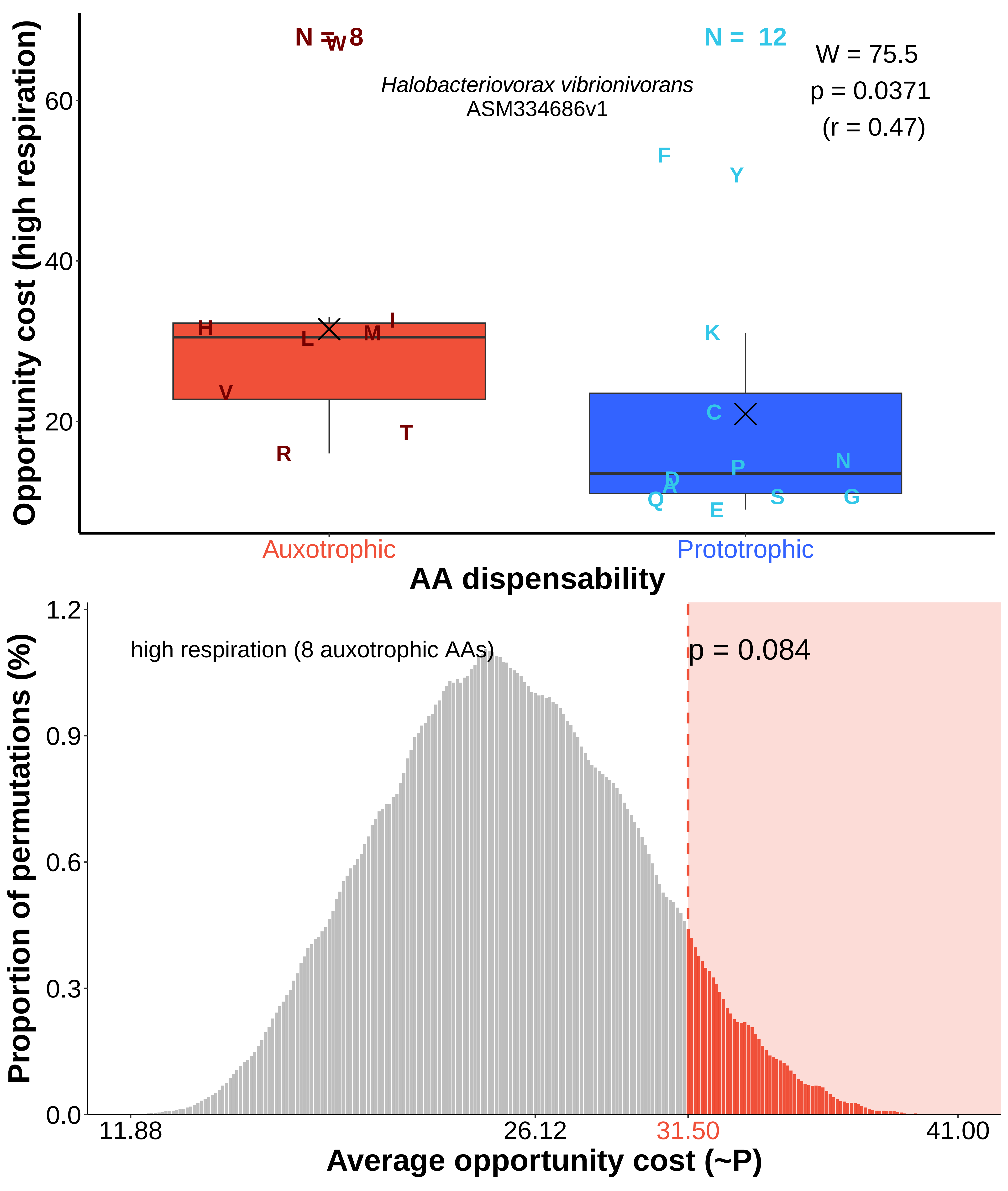
N = 8
W
N = 12
W = 75.5
p = 0.0371
(r = 0.47)
Halobacteriovorax vibrionivorans
ASM334686v1
F
Y
K
I
H
L
M
V
T
R
C
N
P
D
A
Q
S
G
E
60
40
20
Opportunity cost (high respiration)
Auxotrophic
Prototrophic
AA dispensability
high respiration (8 auxotrophic AAs)
p = 0.084
1.2
0.9
0.6
0.3
0.0
Proportion of permutations (%)
11.88
26.12
31.50
41.00
Average opportunity cost (~P)

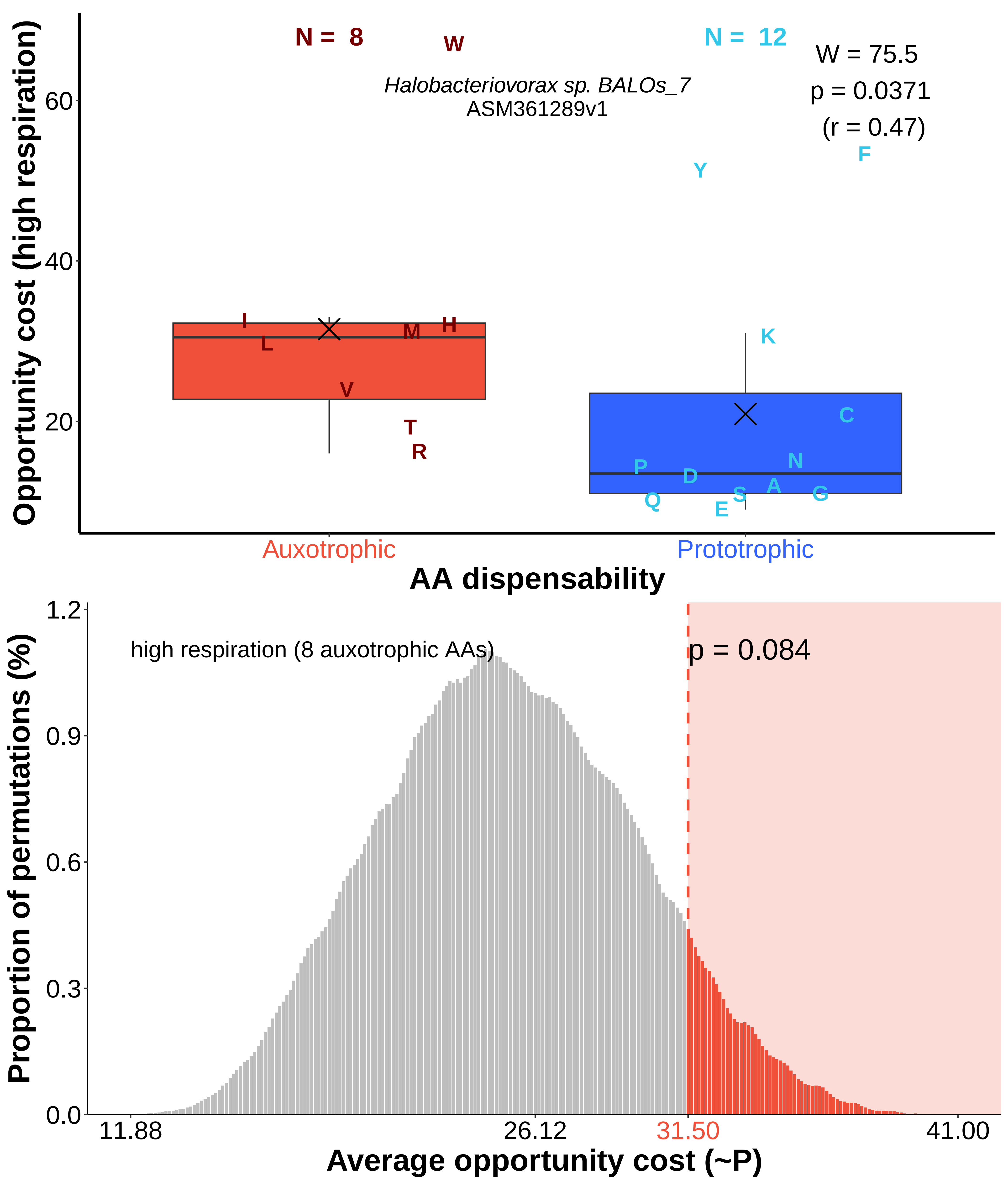
N = 8
W
N = 12
W = 75.5
p = 0.0371
(r = 0.47)
Halobacteriovorax sp. BALOs_7
ASM361289v1
F
Y
I
H
M
L
K
V
C
T
R
N
P
D
A
S
G
Q
E
60
40
20
Opportunity cost (high respiration)
Auxotrophic
Prototrophic
AA dispensability
1.2
0.9
0.6
0.3
0.0
high respiration (8 auxotrophic AAs)
p = 0.084
Proportion of permutations (%)
11.88
26.12
31.50
41.00
Average opportunity cost (~P)

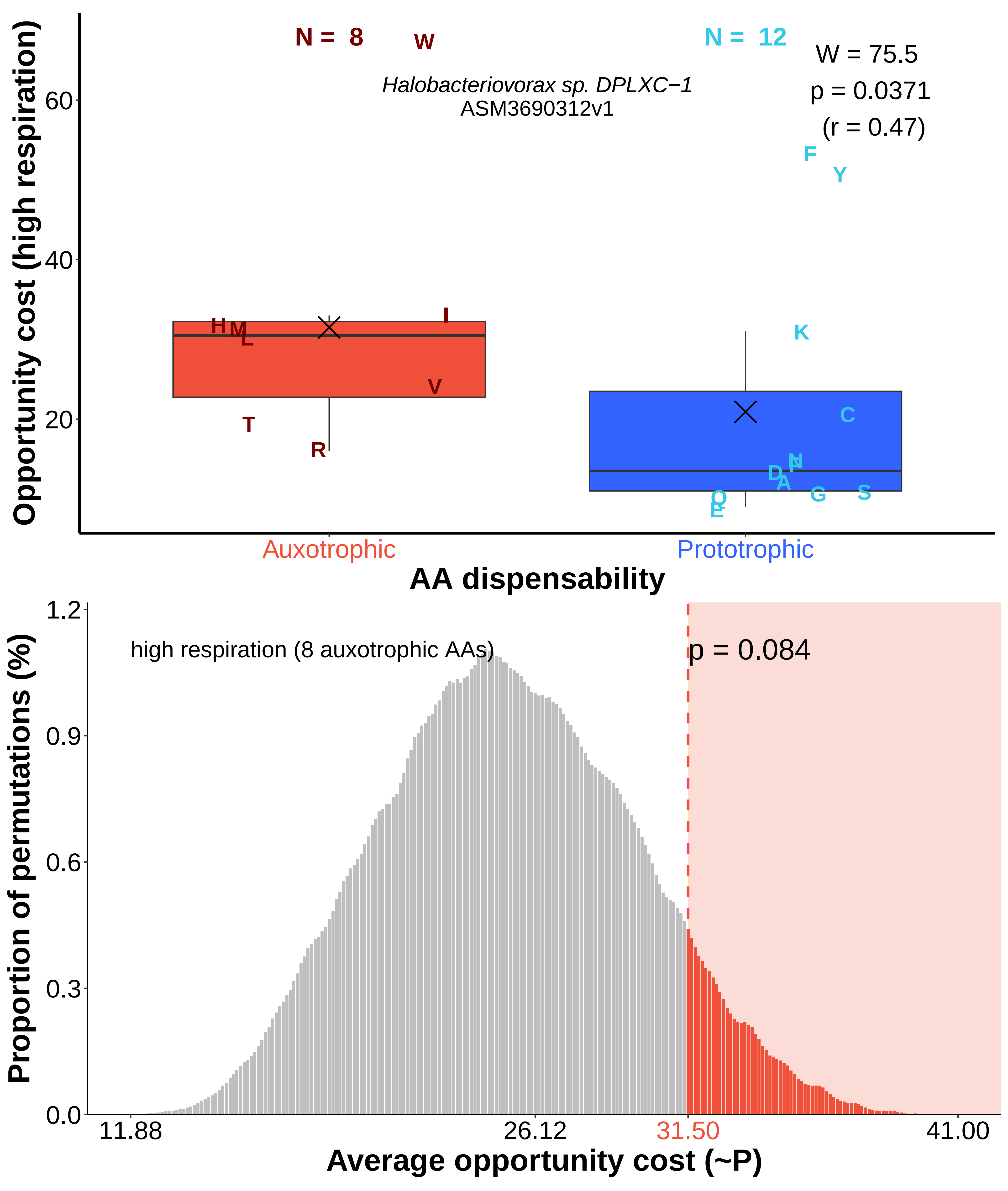
N = 8
W
N = 12
W = 75.5
p = 0.0371
(r = 0.47)
Halobacteriovorax sp. DPLXC−1
ASM3690312v1
Opportunity cost (high respiration)
Auxotrophic
Prototrophic
AA dispensability
high respiration (8 auxotrophic AAs)
p = 0.084
Proportion of permutations (%)
11.88
26.12
31.50
41.00
Average opportunity cost (~P)

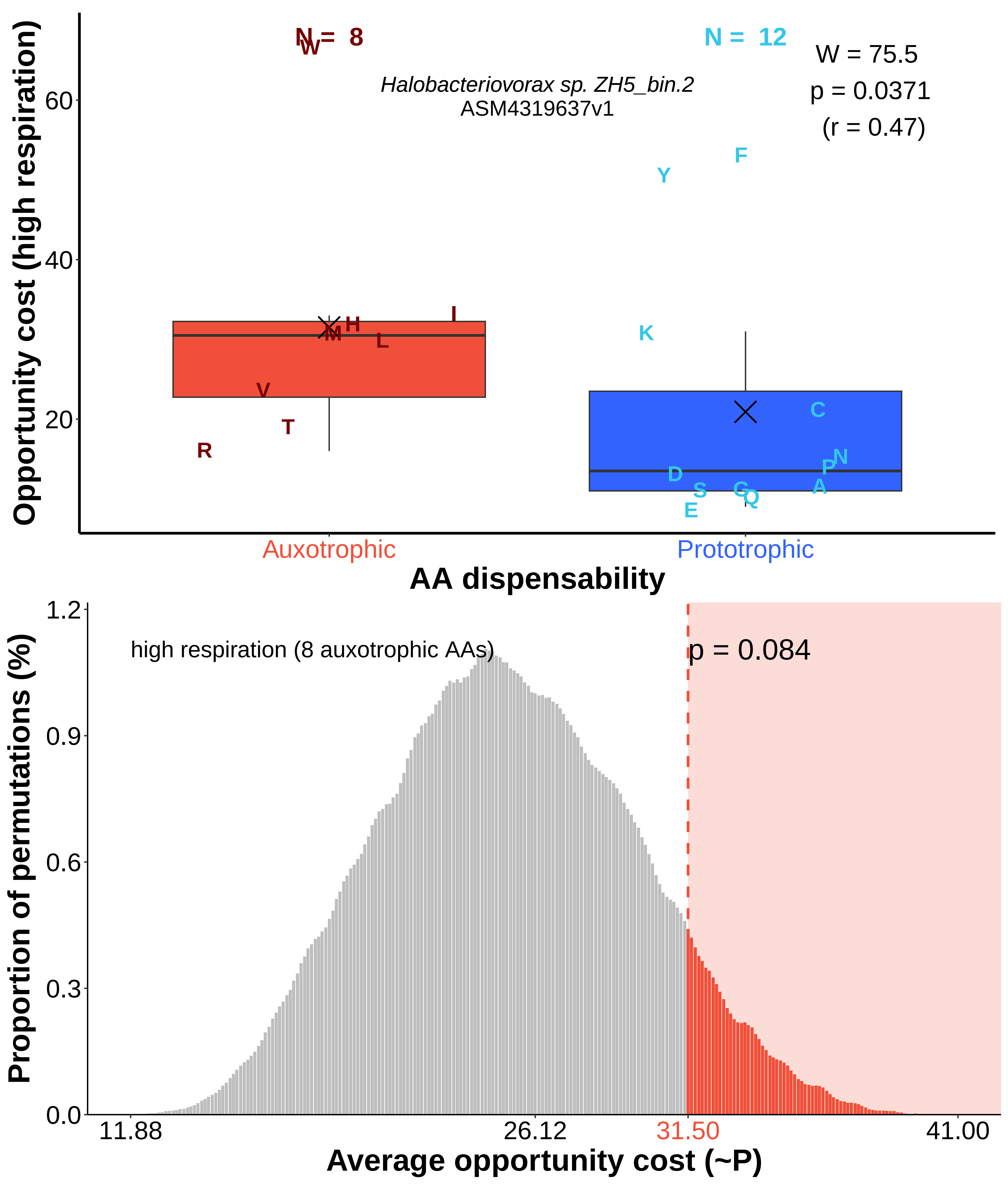

N = 8
N = 12
W = 75.5
p = 0.0371
(r = 0.47)
Halobacteriovorax sp. ZH5_bin.2
ASM4319637v1
Opportunity cost (high respiration)
60
40
20
W
I
H
M
L
V
T
R
F
Y
K
C
N
P
D
S
G
Q
A
E
Auxotrophic
Prototrophic
AA dispensability
Proportion of permutations (%)
1.2
0.9
0.6
0.3
0.0
high respiration (8 auxotrophic AAs)
p = 0.084
11.88
26.12
31.50
41.00
Average opportunity cost (~P)

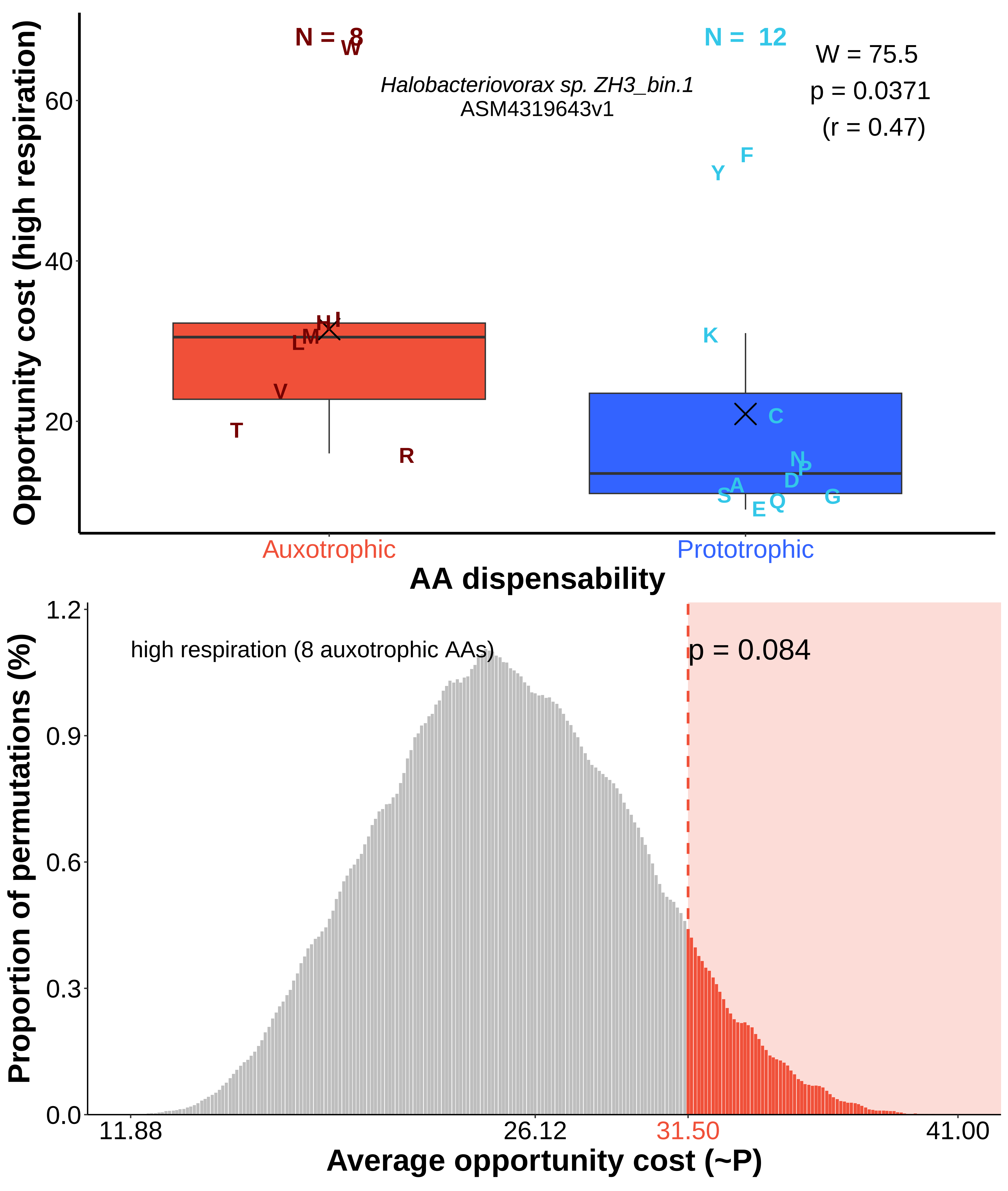

N = 8
N = 12
W = 75.5
p = 0.0371
(r = 0.47)
Halobacteriovorax sp. ZH3_bin.1
ASM4319643v1
Opportunity cost (high respiration)
60
40
20
W
H
I
M
L
V
T
R
F
Y
K
C
N
P
D
A
S
Q
G
E
Auxotrophic
Prototrophic
AA dispensability
Proportion of permutations (%)
1.2
0.9
0.6
0.3
0.0
high respiration (8 auxotrophic AAs)
p = 0.084
11.88
26.12
31.50
41.00
Average opportunity cost (~P)

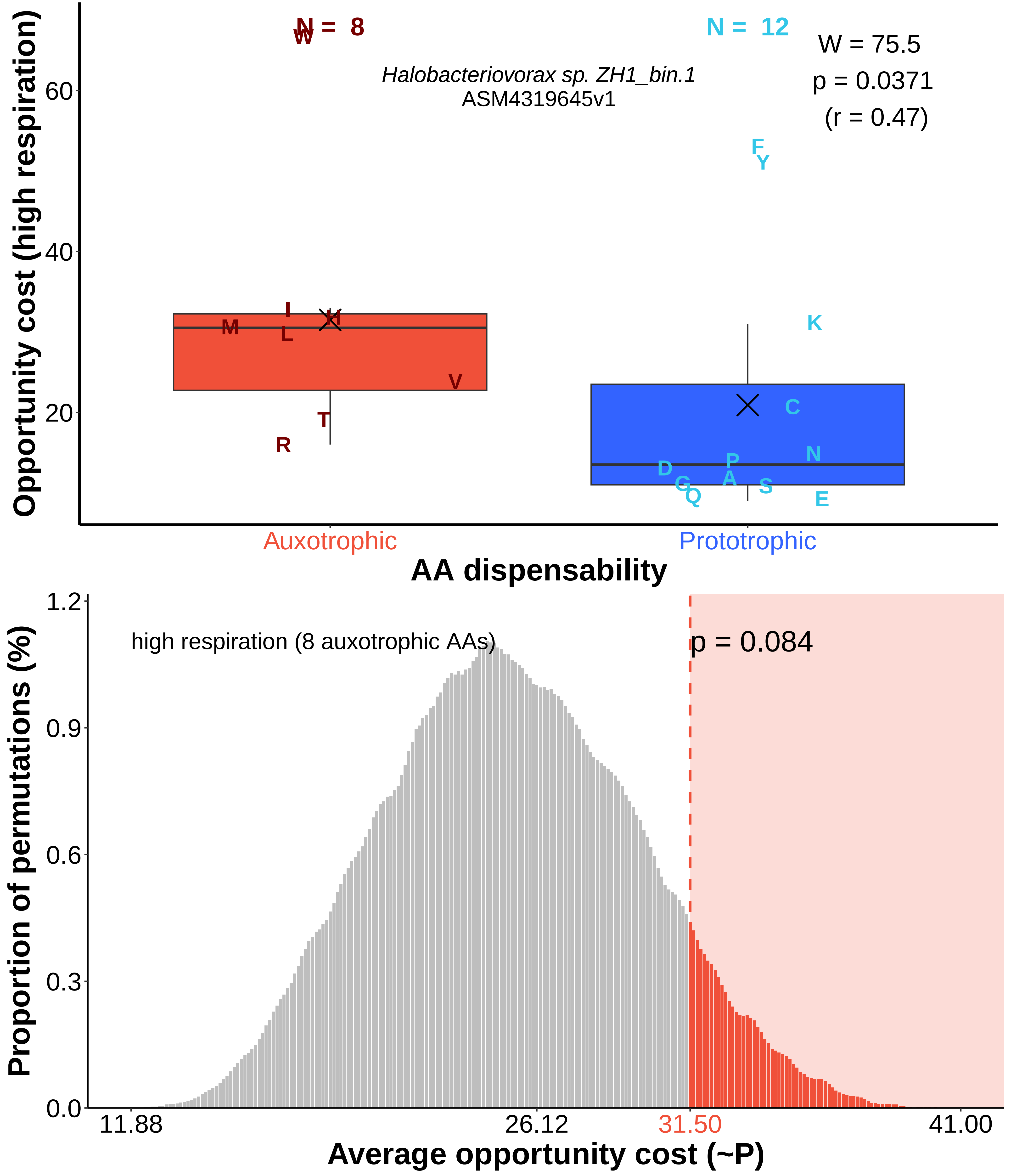
N = 8
N = 12
W = 75.5
p = 0.0371
(r = 0.47)
Halobacteriovorax sp. ZH1_bin.1
ASM4319645v1
Opportunity cost (high respiration)
Auxotrophic
Prototrophic
AA dispensability
high respiration (8 auxotrophic AAs)
p = 0.084
Proportion of permutations (%)
Average opportunity cost (~P)

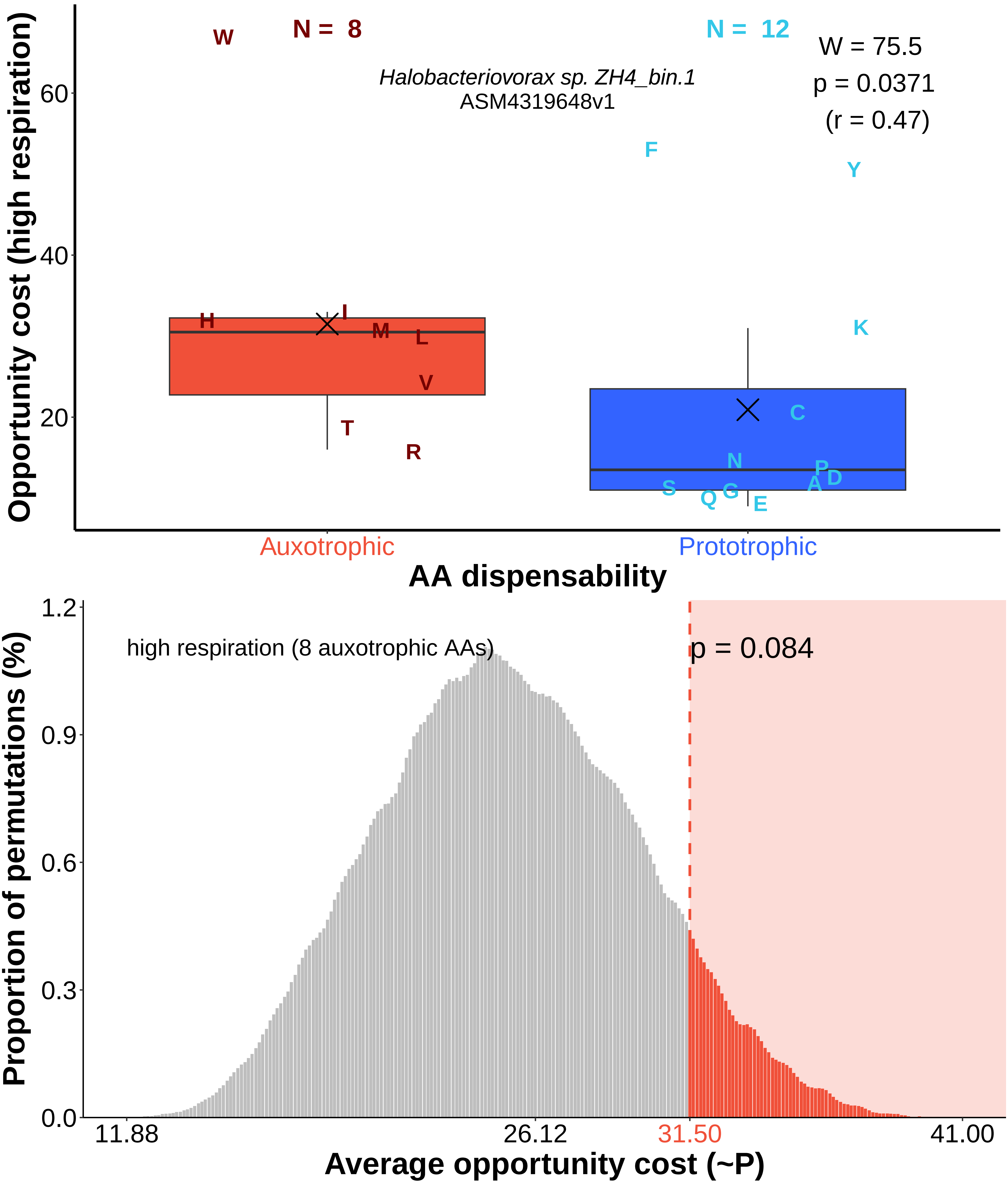

N = 8
N = 12
W = 75.5
p = 0.0371
(r = 0.47)
Halobacteriovorax sp. ZH4_bin.1
ASM4319648v1
Opportunity cost (high respiration)
Auxotrophic
Prototrophic
AA dispensability
high respiration (8 auxotrophic AAs)
p = 0.084
Proportion of permutations (%)
Average opportunity cost (~P)

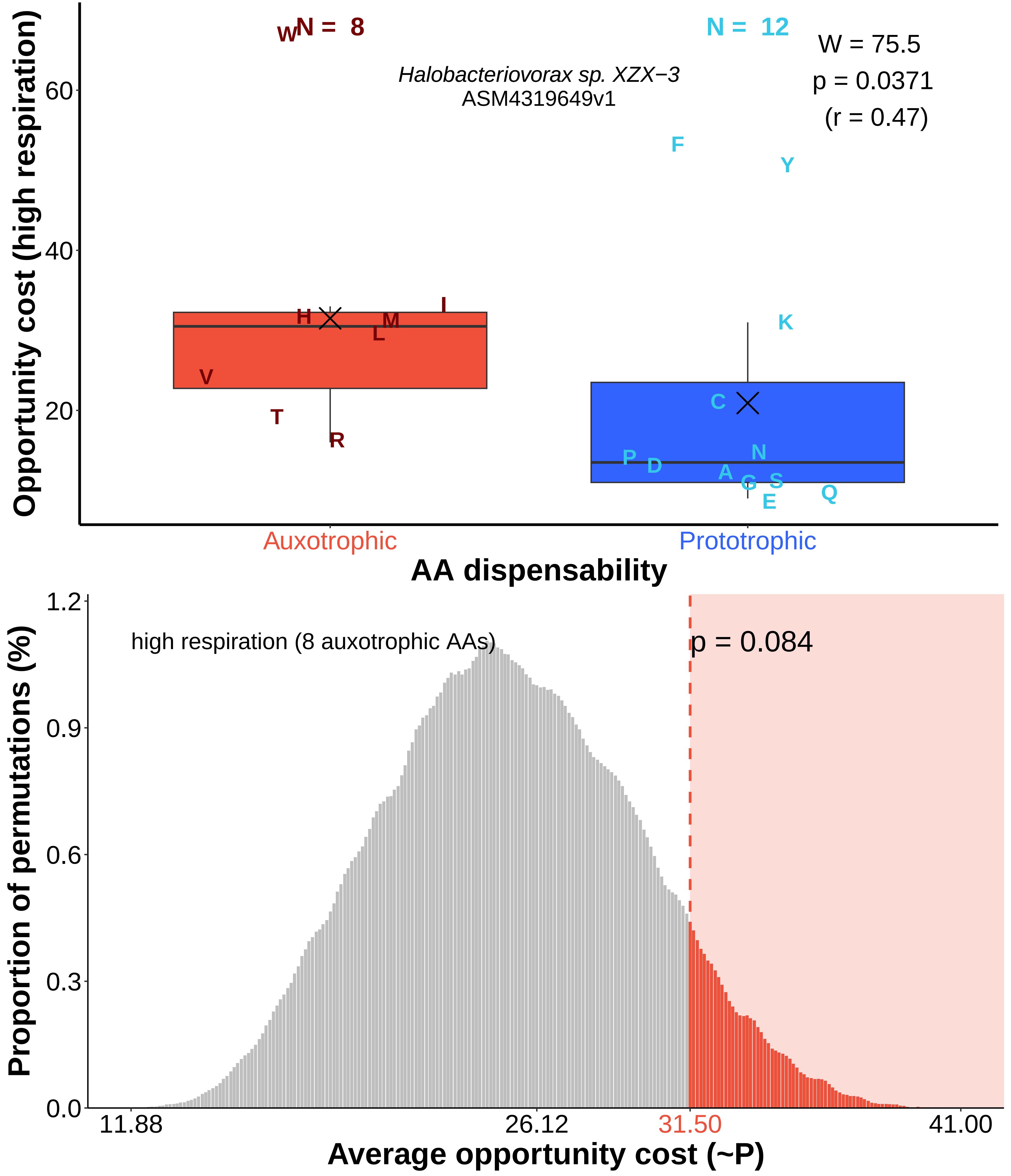

N = 8
N = 12
W = 75.5
p = 0.0371
(r = 0.47)
Halobacteriovorax sp. XZX−3
ASM4319649v1
Opportunity cost (high respiration)
60
40
20
W
H
M
L
I
V
T
R
F
Y
K
C
P
D
N
A
G
S
E
Q
Auxotrophic
Prototrophic
AA dispensability
Proportion of permutations (%)
1.2
0.9
0.6
0.3
0.0
high respiration (8 auxotrophic AAs)
p = 0.084
11.88
26.12
31.50
41.00
Average opportunity cost (~P)

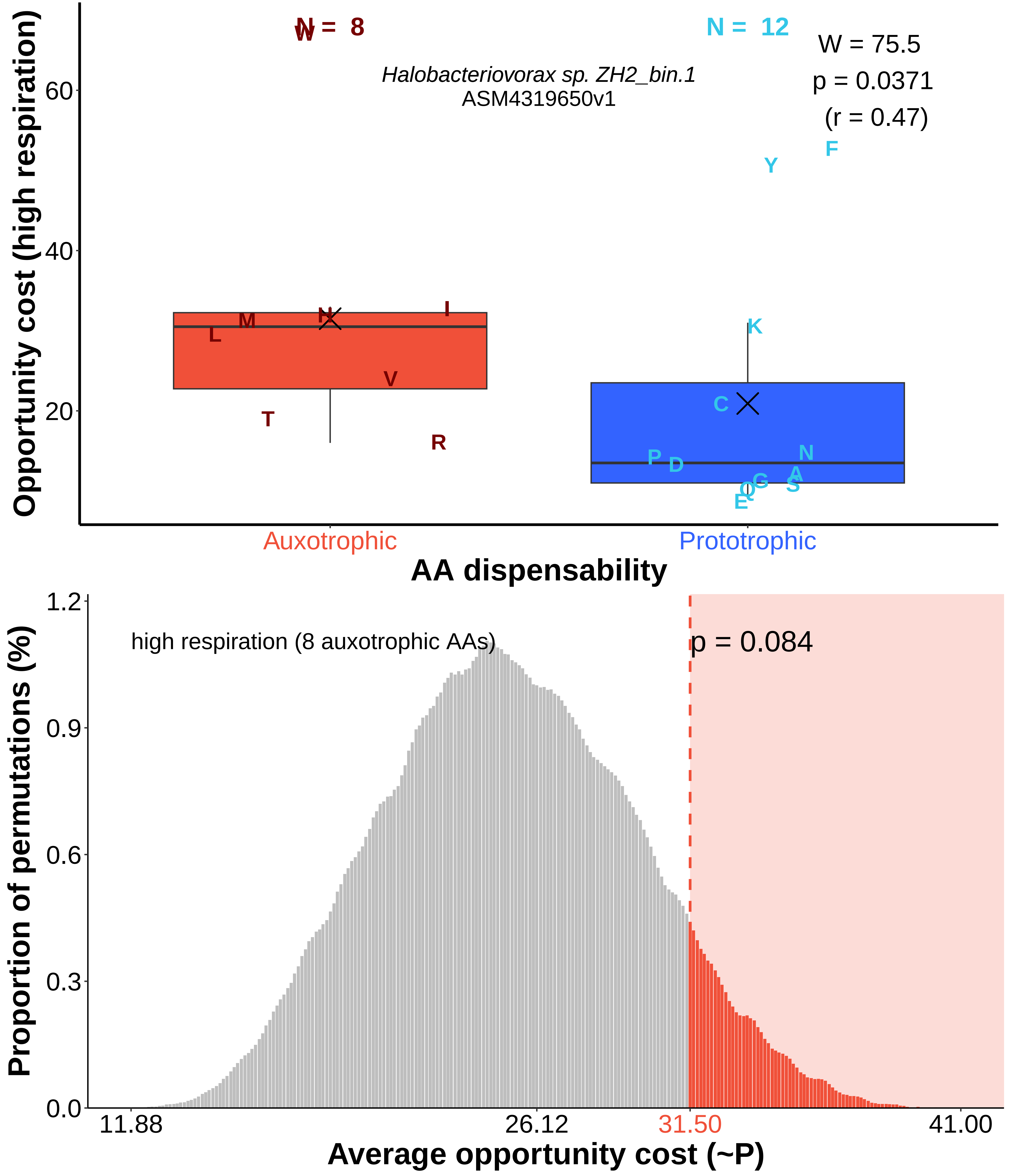

N = 8
N = 12
W = 75.5
p = 0.0371
(r = 0.47)
Halobacteriovorax sp. ZH2_bin.1
ASM4319650v1
Opportunity cost (high respiration)
60
40
20
Auxotrophic
Prototrophic
AA dispensability
Proportion of permutations (%)
1.2
0.9
0.6
0.3
0.0
high respiration (8 auxotrophic AAs)
p = 0.084
11.88
26.12
31.50
41.00
Average opportunity cost (~P)

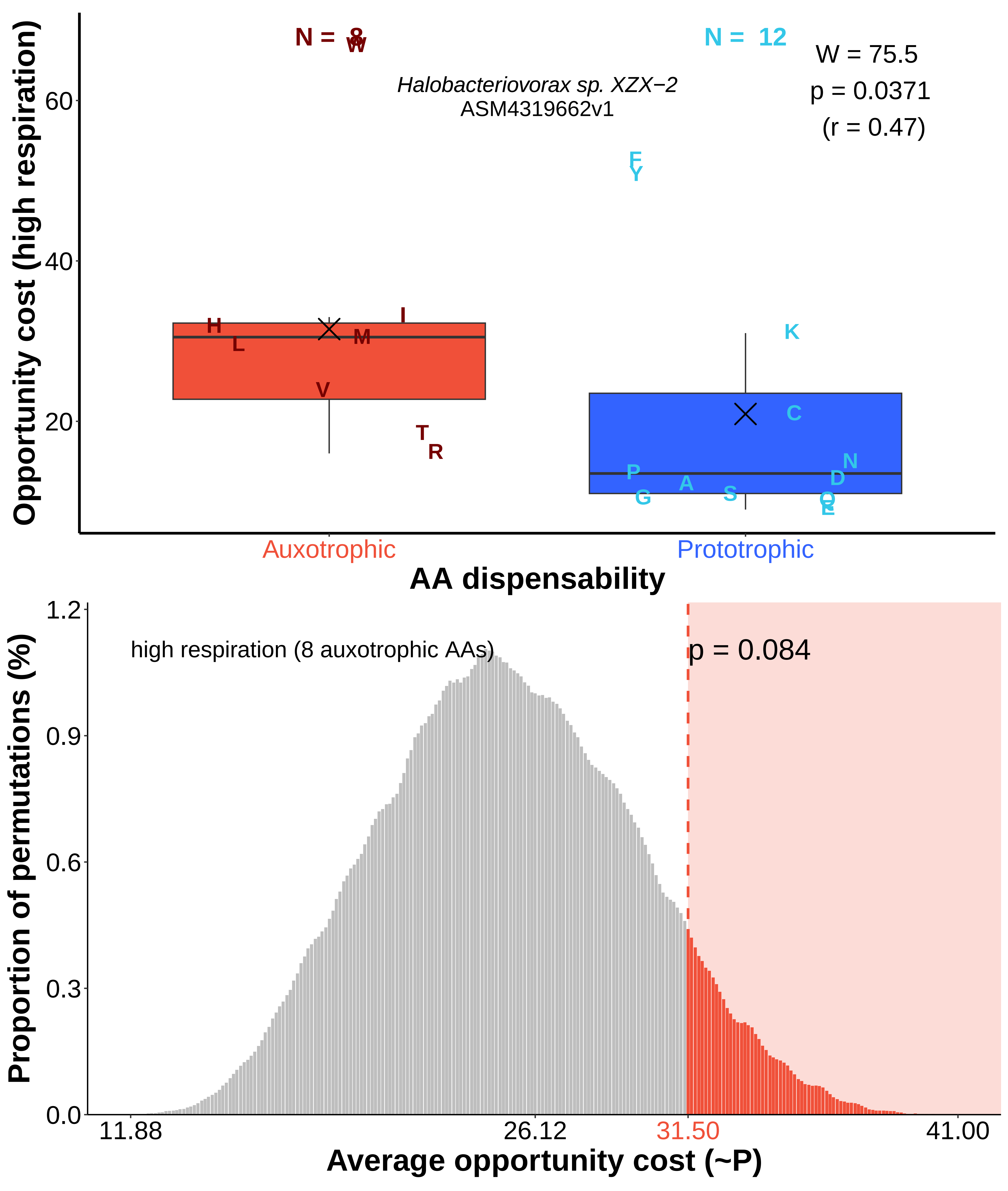
N = 8
N = 12
W = 75.5
p = 0.0371
(r = 0.47)
Halobacteriovorax sp. XZX−2
ASM4319662v1
Opportunity cost (high respiration)
Auxotrophic
Prototrophic
AA dispensability
high respiration (8 auxotrophic AAs)
p = 0.084
Proportion of permutations (%)
11.88
26.12
31.50
41.00
Average opportunity cost (~P)

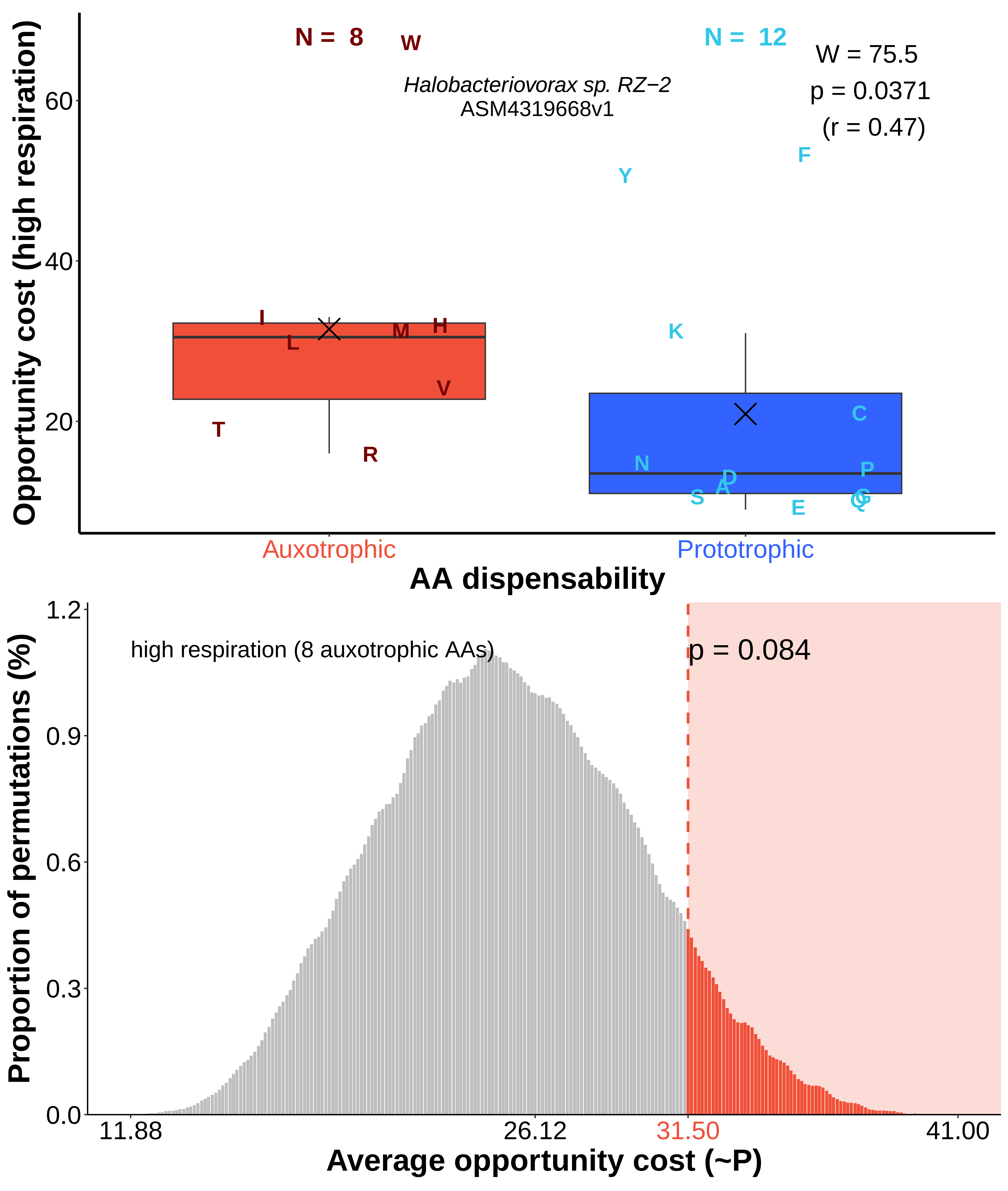

N = 8
N = 12
W = 75.5
p = 0.0371
(r = 0.47)
Halobacteriovorax sp. RZ−2
ASM4319668v1
Opportunity cost (high respiration)
Auxotrophic
Prototrophic
AA dispensability
high respiration (8 auxotrophic AAs)
p = 0.084
Proportion of permutations (%)
11.88
26.12
31.50
41.00
Average opportunity cost (~P)

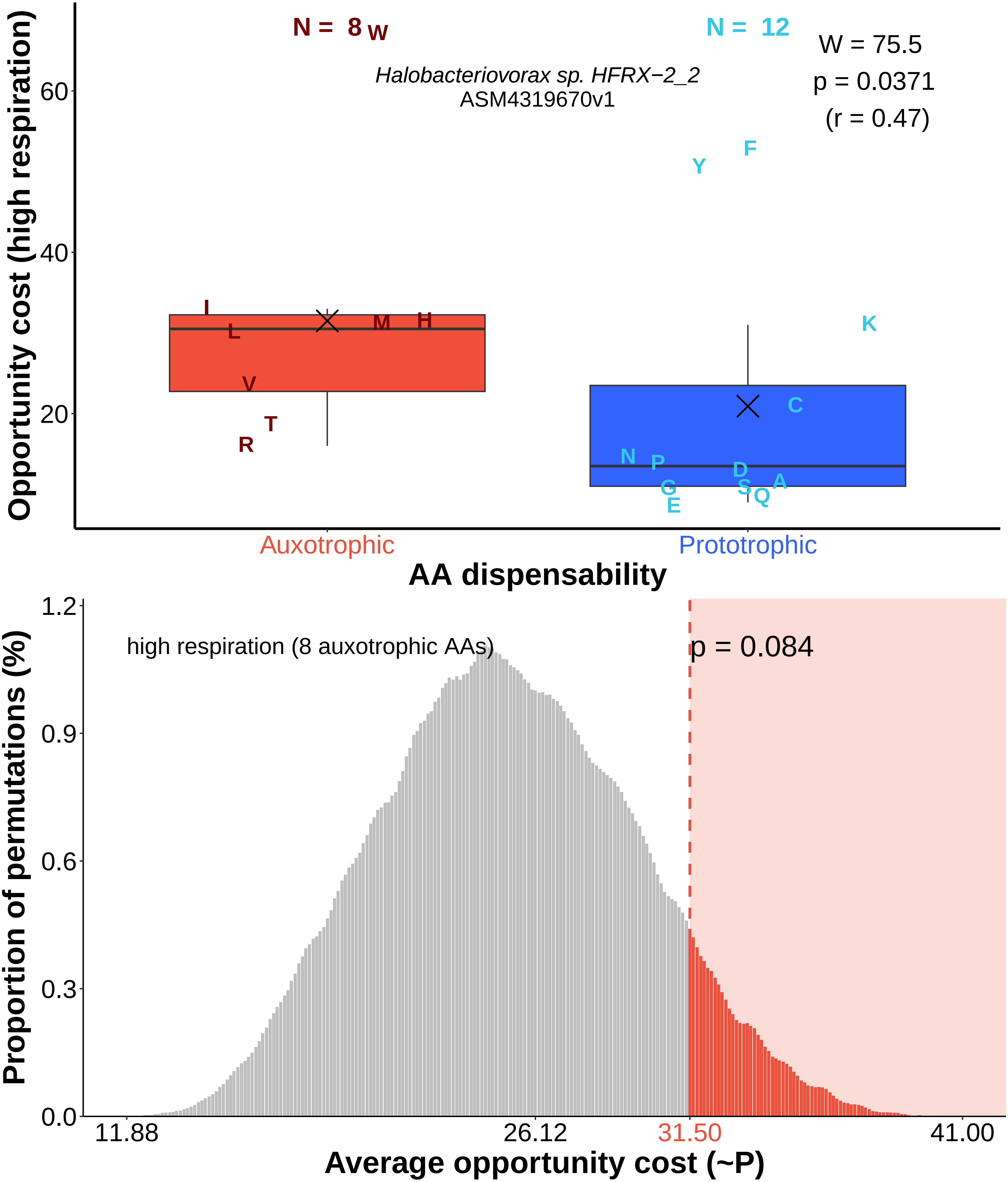
N = 8
N = 12
W = 75.5
p = 0.0371
(r = 0.47)
Halobacteriovorax sp. HFRX−2_2
ASM4319670v1
W
I
L
M
H
V
T
R
Y
F
K
C
N
P
D
G
S
A
Q
E
Opportunity cost (high respiration)
60
40
20
Auxotrophic
Prototrophic
AA dispensability
high respiration (8 auxotrophic AAs)
p = 0.084
Proportion of permutations (%)
1.2
0.9
0.6
0.3
0.0
11.88
26.12
31.50
41.00
Average opportunity cost (~P)

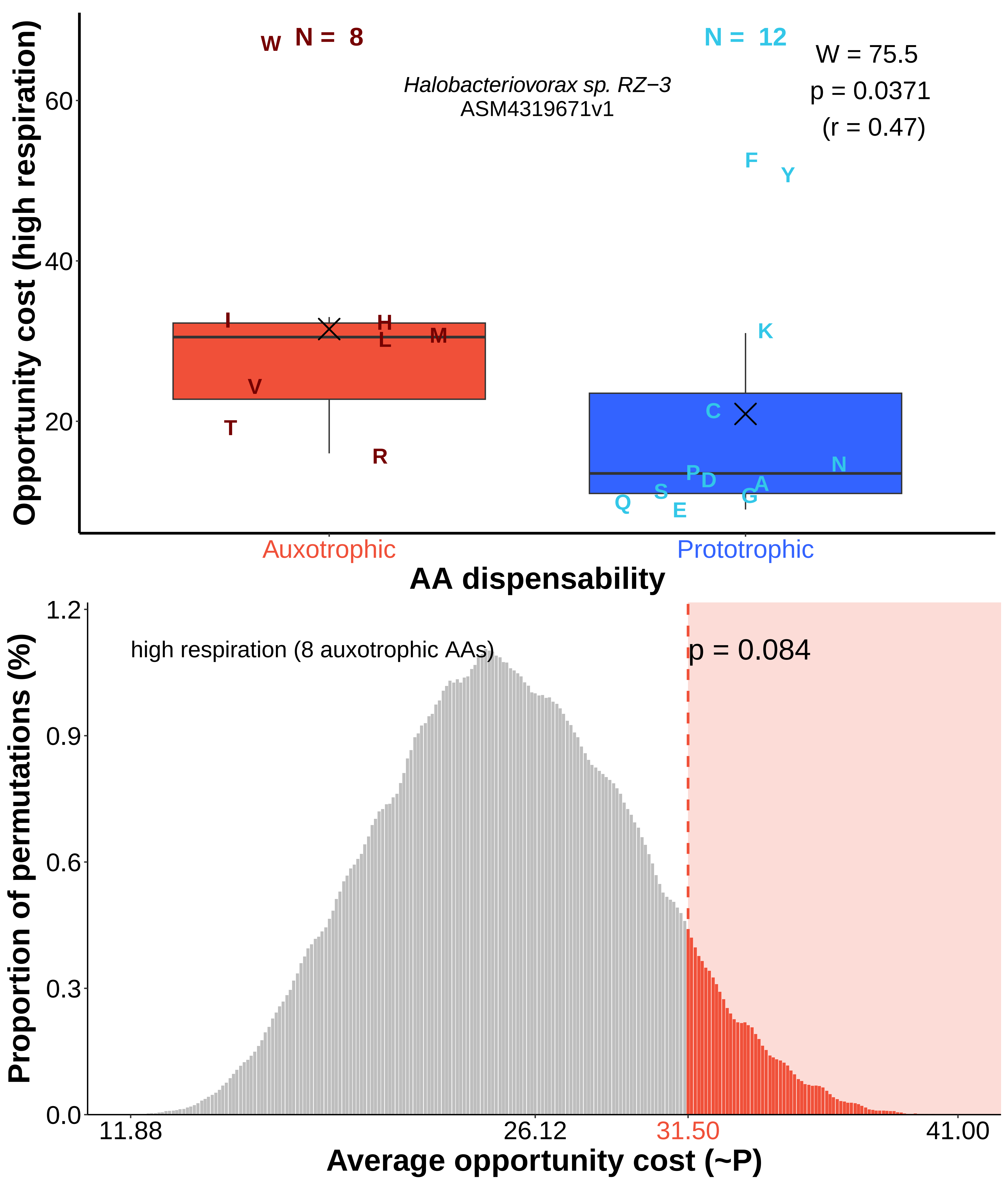

W N = 8
N = 12
Halobacteriovorax sp. RZ−3
ASM4319671v1
W = 75.5
p = 0.0371
(r = 0.47)
Opportunity cost (high respiration)
Auxotrophic
Prototrophic
AA dispensability
I H L M V T R
F Y K C P D S A G Q E N
60
40
20
Proportion of permutations (%)
high respiration (8 auxotrophic AAs)
p = 0.084
1.2
0.9
0.6
0.3
0.0
11.88
26.12
31.50
41.00
Average opportunity cost (~P)

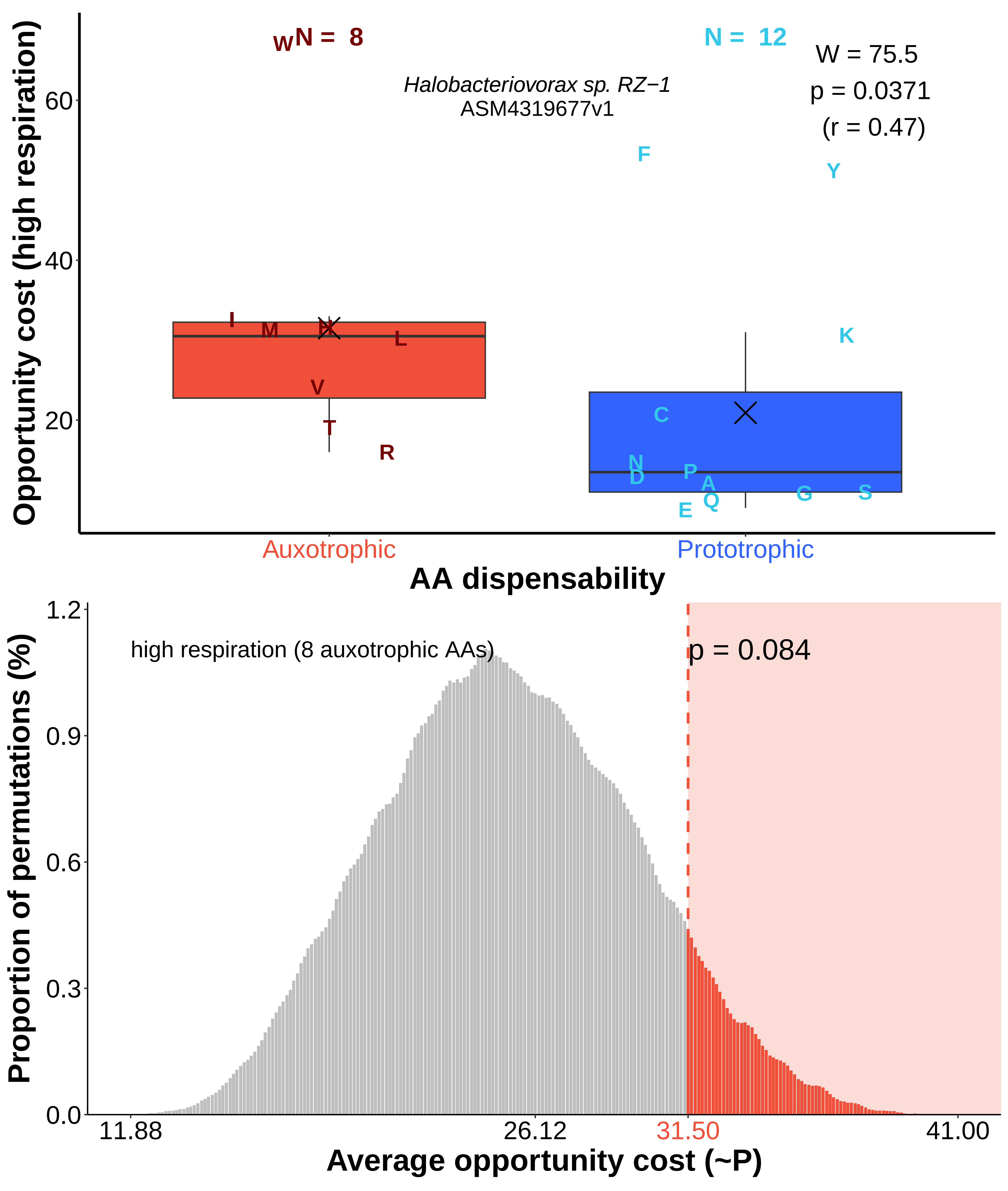

W
N = 8
N = 12
Halobacteriovorax sp. RZ−1
ASM4319677v1
W = 75.5
p = 0.0371
(r = 0.47)
Opportunity cost (high respiration)
60
40
20
F
Y
I
M
H
L
V
T
R
K
C
N
D
P
A
Q
E
G
S
Auxotrophic
Prototrophic
AA dispensability
Proportion of permutations (%)
1.2
0.9
0.6
0.3
0.0
high respiration (8 auxotrophic AAs)
p = 0.084
11.88
26.12
31.50
41.00
Average opportunity cost (~P)

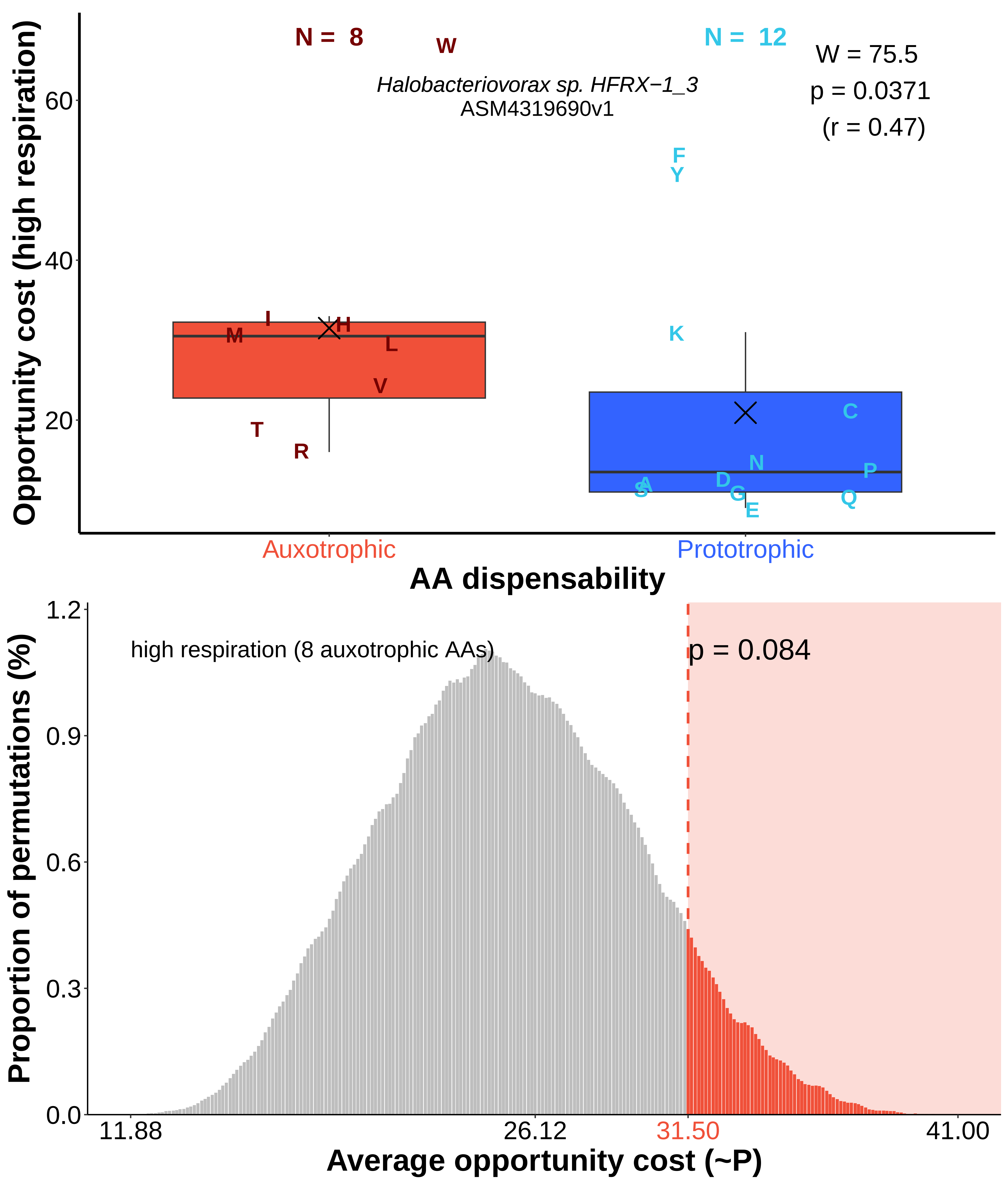
N = 8
W
N = 12
W = 75.5
p = 0.0371
(r = 0.47)
Halobacteriovorax sp. HFRX−1_3
ASM4319690v1
F
Y
I
M
H
L
V
T
R
K
C
N
P
D
A
S
G
Q
E
60
40
20
Opportunity cost (high respiration)
Auxotrophic
Prototrophic
AA dispensability
high respiration (8 auxotrophic AAs)
p = 0.084
1.2
0.9
0.6
0.3
0.0
Proportion of permutations (%)
11.88
26.12
31.50
41.00
Average opportunity cost (~P)

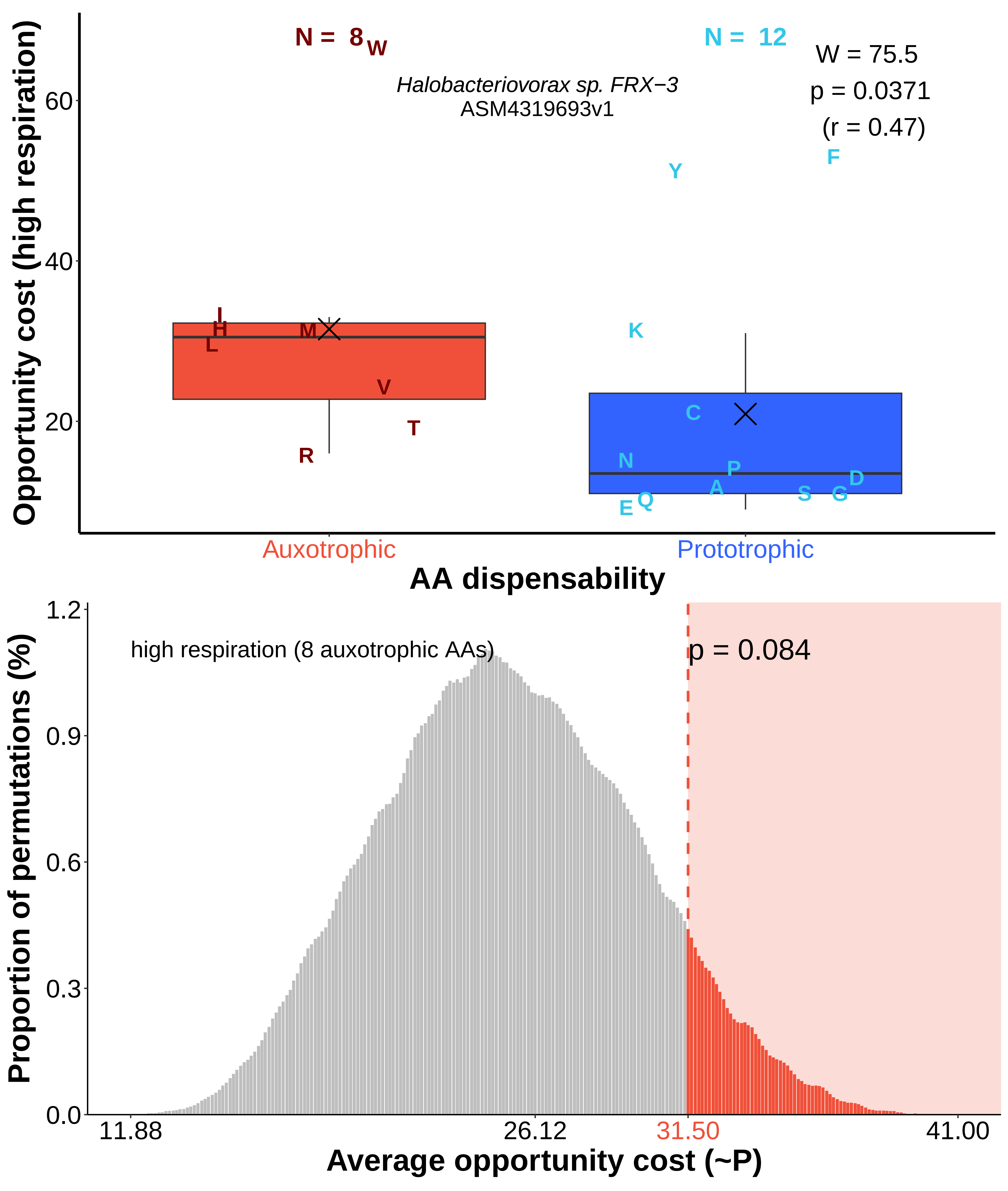

N = 8
N = 12
W = 75.5
p = 0.0371
(r = 0.47)
Halobacteriovorax sp. FRX−3
ASM4319693v1
Opportunity cost (high respiration)
Auxotrophic
Prototrophic
AA dispensability
high respiration (8 auxotrophic AAs)
p = 0.084
Proportion of permutations (%)
11.88
26.12
31.50
41.00
Average opportunity cost (~P)

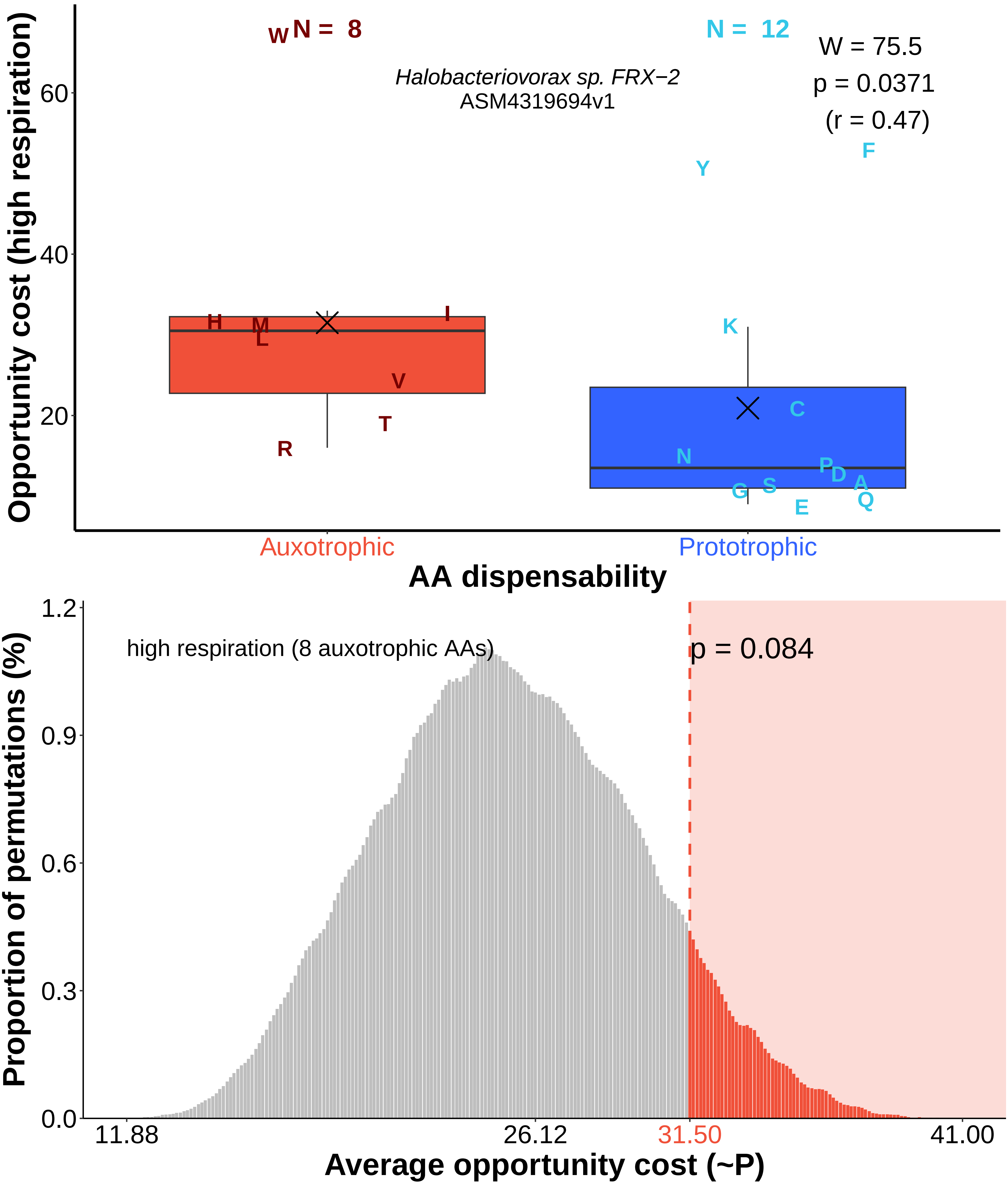

W N = 8
N = 12
Halobacteriovorax sp. FRX−2
ASM4319694v1
W = 75.5
p = 0.0371
(r = 0.47)
Opportunity cost (high respiration)
60
40
20
H M L I V T R
Y F K C N P D A G S Q E
Auxotrophic
Prototrophic
AA dispensability
1.2
0.9
0.6
0.3
0.0
high respiration (8 auxotrophic AAs)
p = 0.084
Proportion of permutations (%)
11.88
26.12
31.50
41.00
Average opportunity cost (~P)

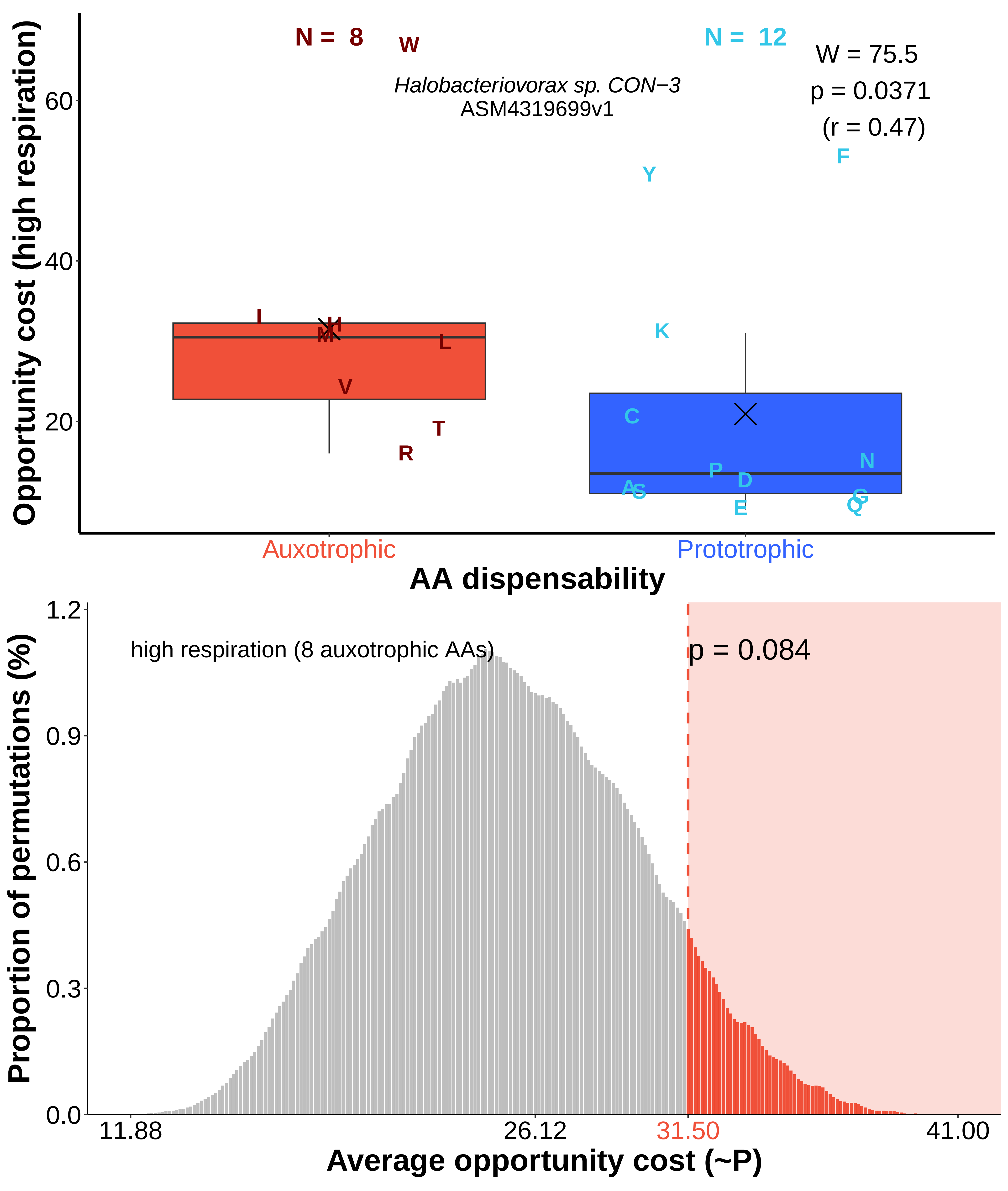

N = 8
N = 12
Halobacteriovorax sp. CON−3
ASM4319699v1
W = 75.5
p = 0.0371
(r = 0.47)
Opportunity cost (high respiration)
Auxotrophic
Prototrophic
AA dispensability
high respiration (8 auxotrophic AAs)
p = 0.084
Proportion of permutations (%)
11.88
26.12
31.50
41.00
Average opportunity cost (~P)

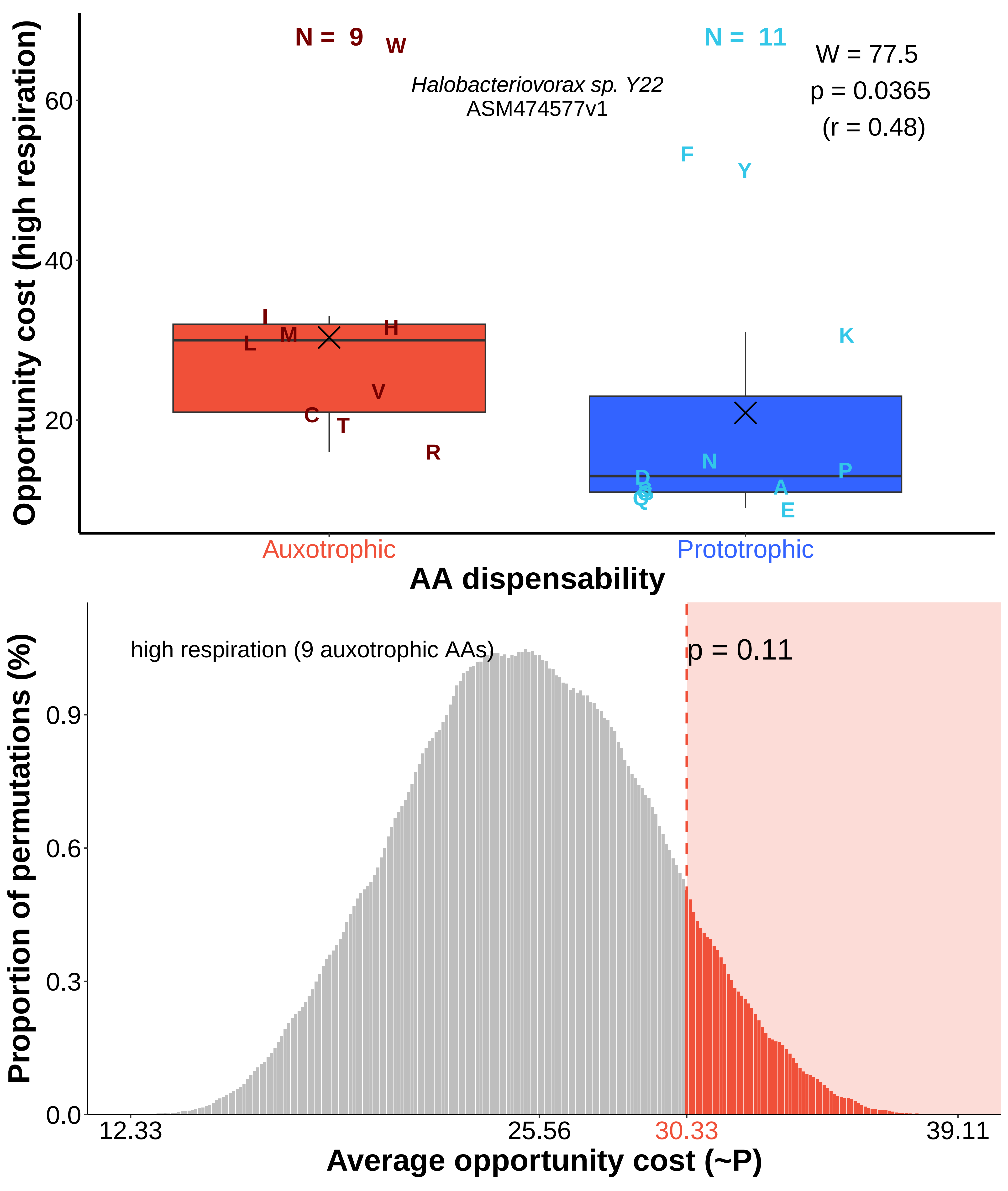
N = 9
N = 11
W = 77.5
p = 0.0365
(r = 0.48)
Halobacteriovorax sp. Y22
ASM474577v1
Opportunity cost (high respiration)
Auxotrophic
Prototrophic
AA dispensability
high respiration (9 auxotrophic AAs)
p = 0.11
Proportion of permutations (%)
12.33
25.56
30.33
39.11
Average opportunity cost (~P)

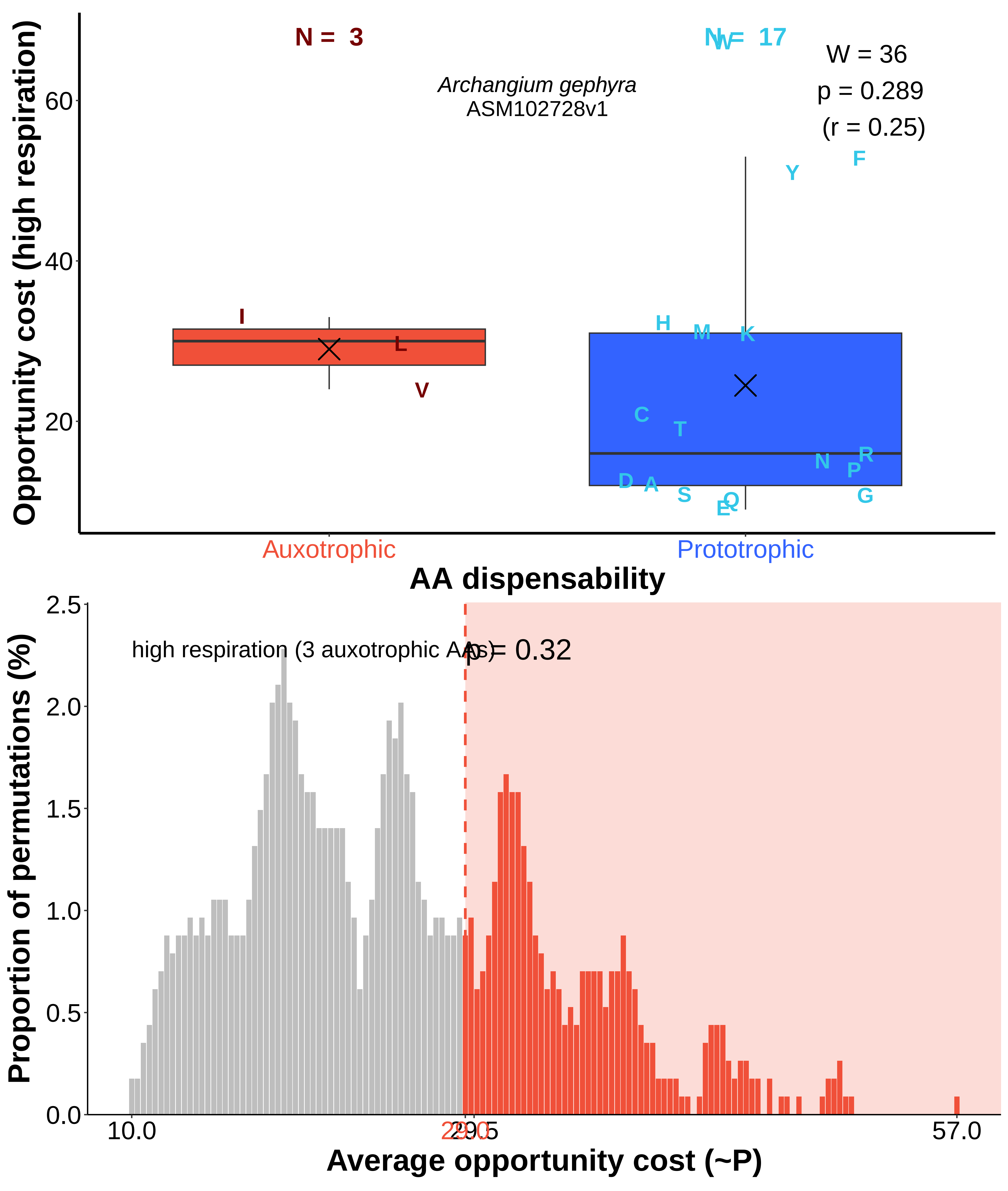

N = 3
N = 17
W
W = 36
p = 0.289
(r = 0.25)
Archangium gephyra
ASM102728v1
Opportunity cost (high respiration)
60
40
20
I
L
V
H
M
K
Y
F
C
T
N
R
P
D
A
S
Q
E
G
Auxotrophic
Prototrophic
AA dispensability
Proportion of permutations (%)
2.5
2.0
1.5
1.0
0.5
0.0
high respiration (3 auxotrophic AAs)
p = 0.32
10.0
29.0
29.5
57.0
Average opportunity cost (~P)

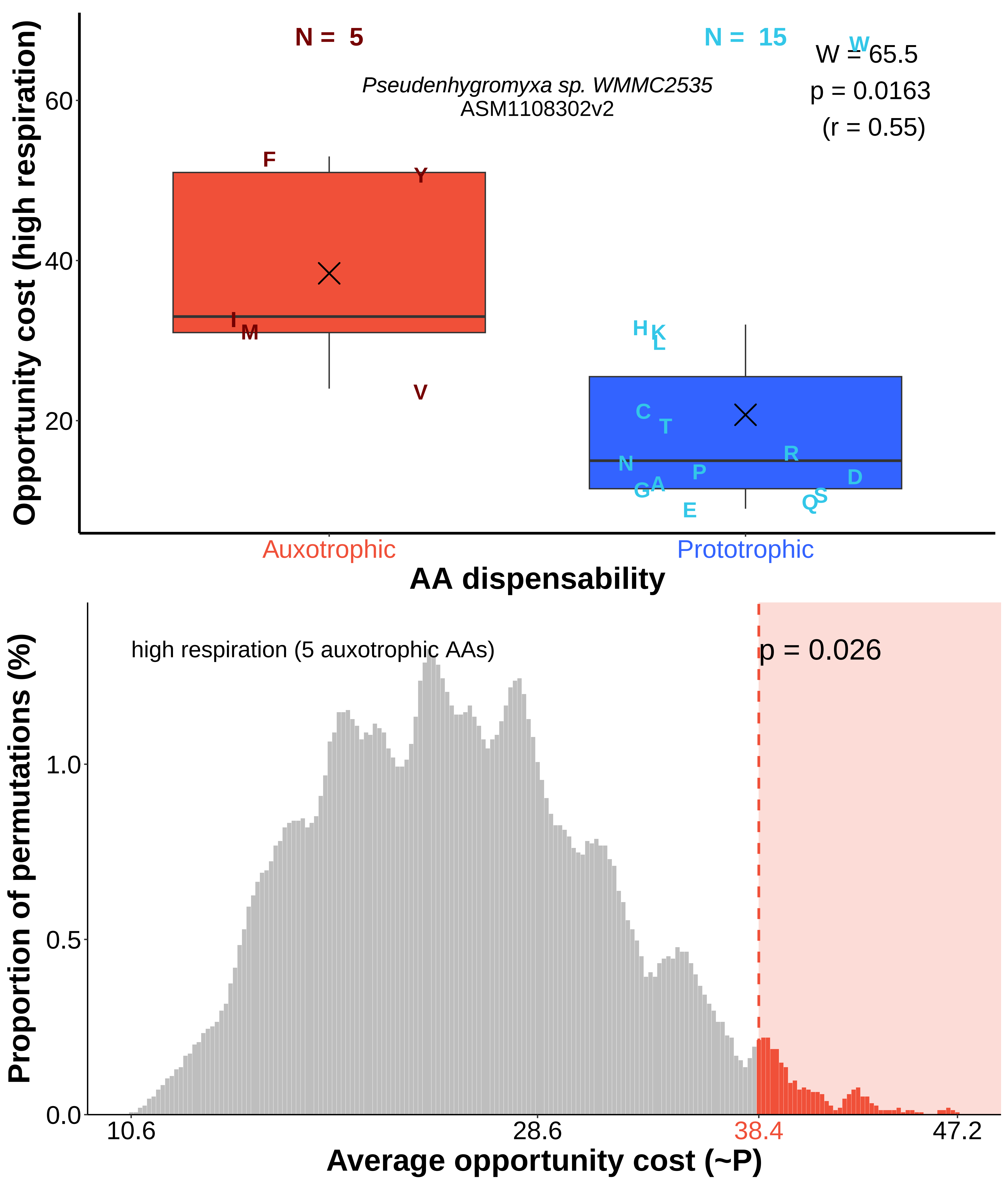
N = 5
N = 15
W = 65.5
p = 0.0163
(r = 0.55)
Pseudenhygromyxa sp. WMMC2535
ASM1108302v2
Opportunity cost (high respiration)
Auxotrophic
Prototrophic
AA dispensability
high respiration (5 auxotrophic AAs)
p = 0.026
Proportion of permutations (%)
Average opportunity cost (~P)

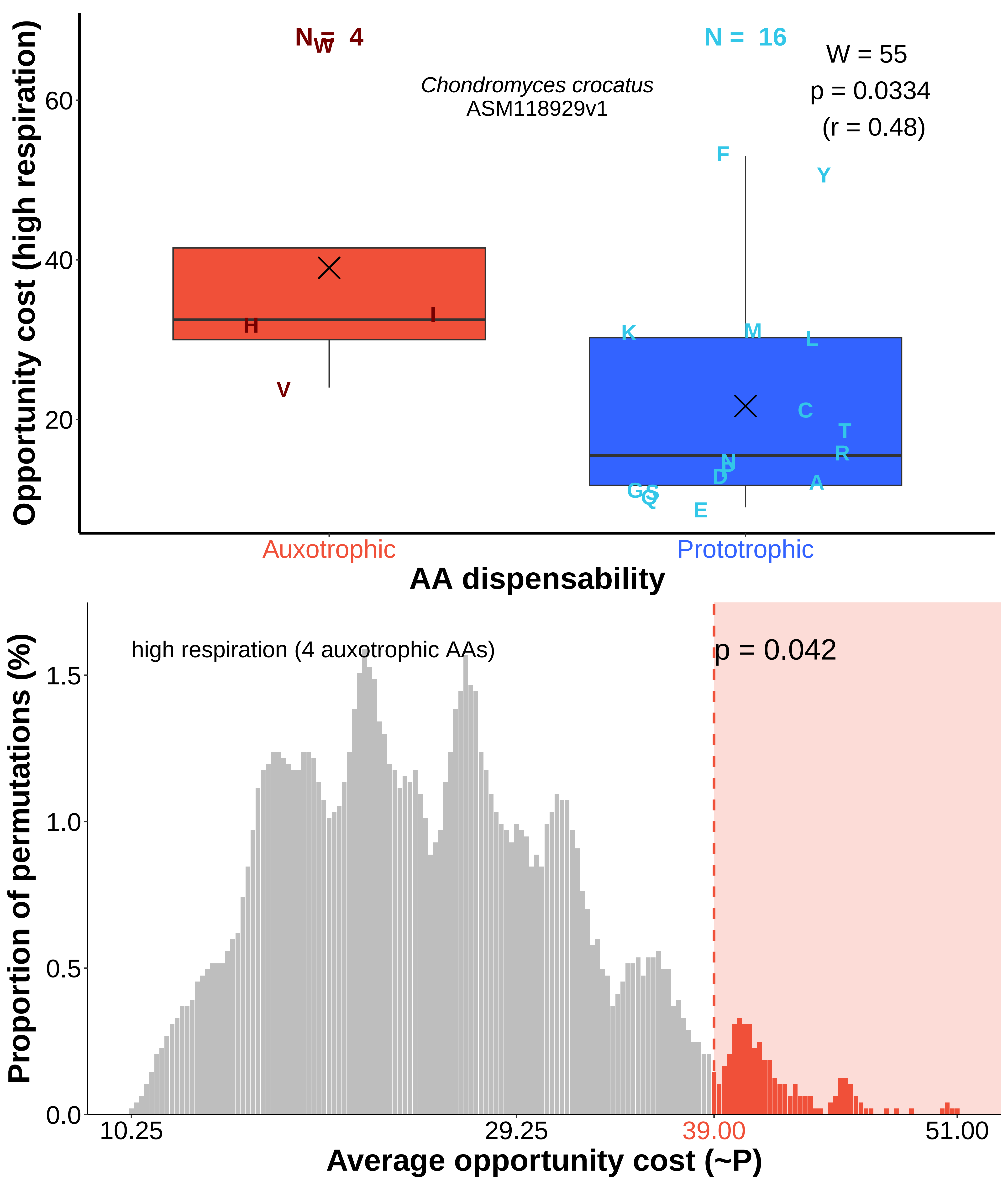

N = 4
N = 16
W = 55
p = 0.0334
(r = 0.48)
Chondromyces crocatus
ASM118929v1
Opportunity cost (high respiration)
Auxotrophic
Prototrophic
AA dispensability
high respiration (4 auxotrophic AAs)
p = 0.042
Proportion of permutations (%)
10.25
29.25
39.00
51.00
Average opportunity cost (~P)

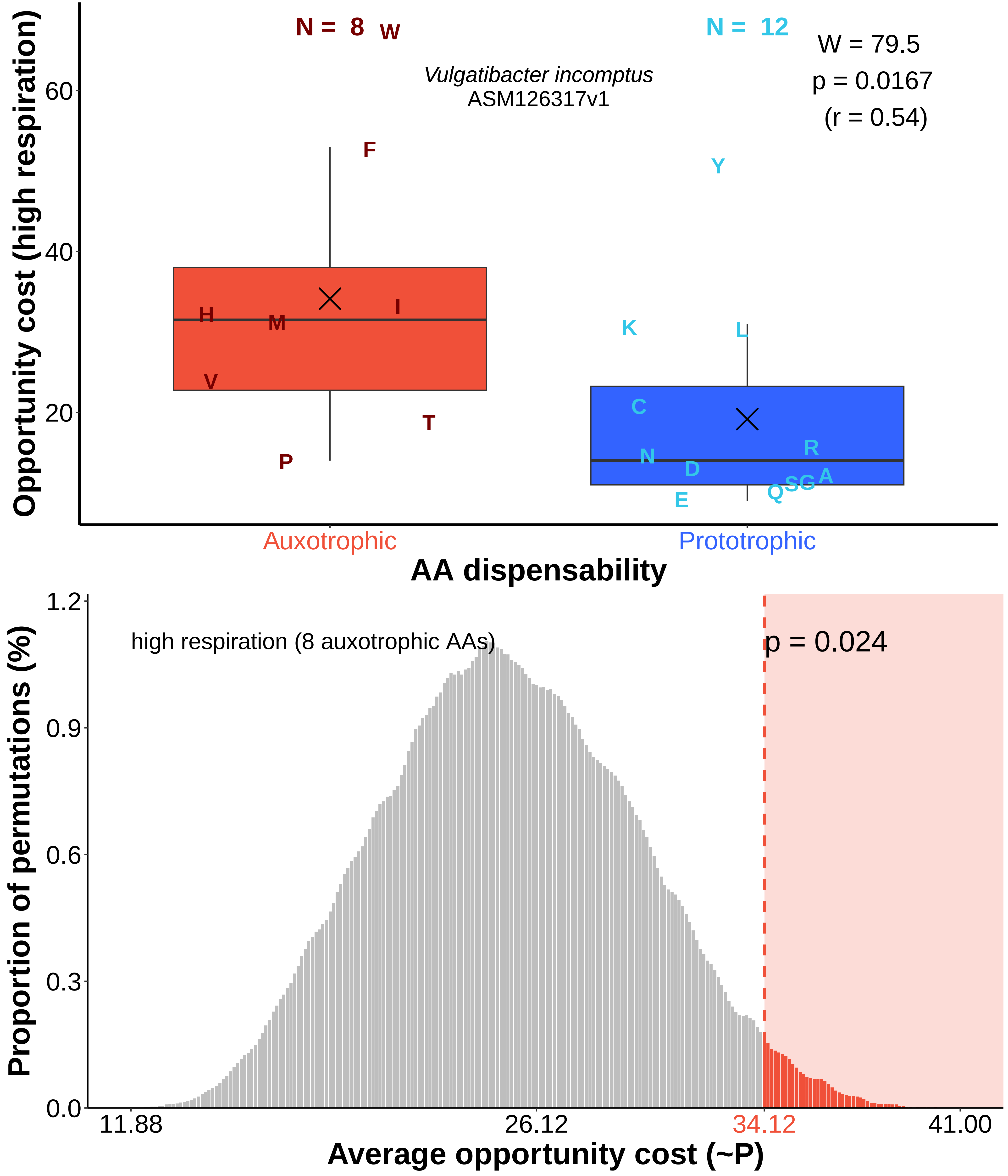
N = 8
W
N = 12
W = 79.5
p = 0.0167
(r = 0.54)
Vulgatibacter incomptus
ASM126317v1
F
Y
H
M
I
K
L
V
C
T
N
R
D
P
Q
S
G
A
E
Opportunity cost (high respiration)
60
40
20
Auxotrophic
Prototrophic
AA dispensability
1.2
0.9
0.6
0.3
0.0
high respiration (8 auxotrophic AAs)
p = 0.024
Proportion of permutations (%)
11.88
26.12
34.12
41.00
Average opportunity cost (~P)

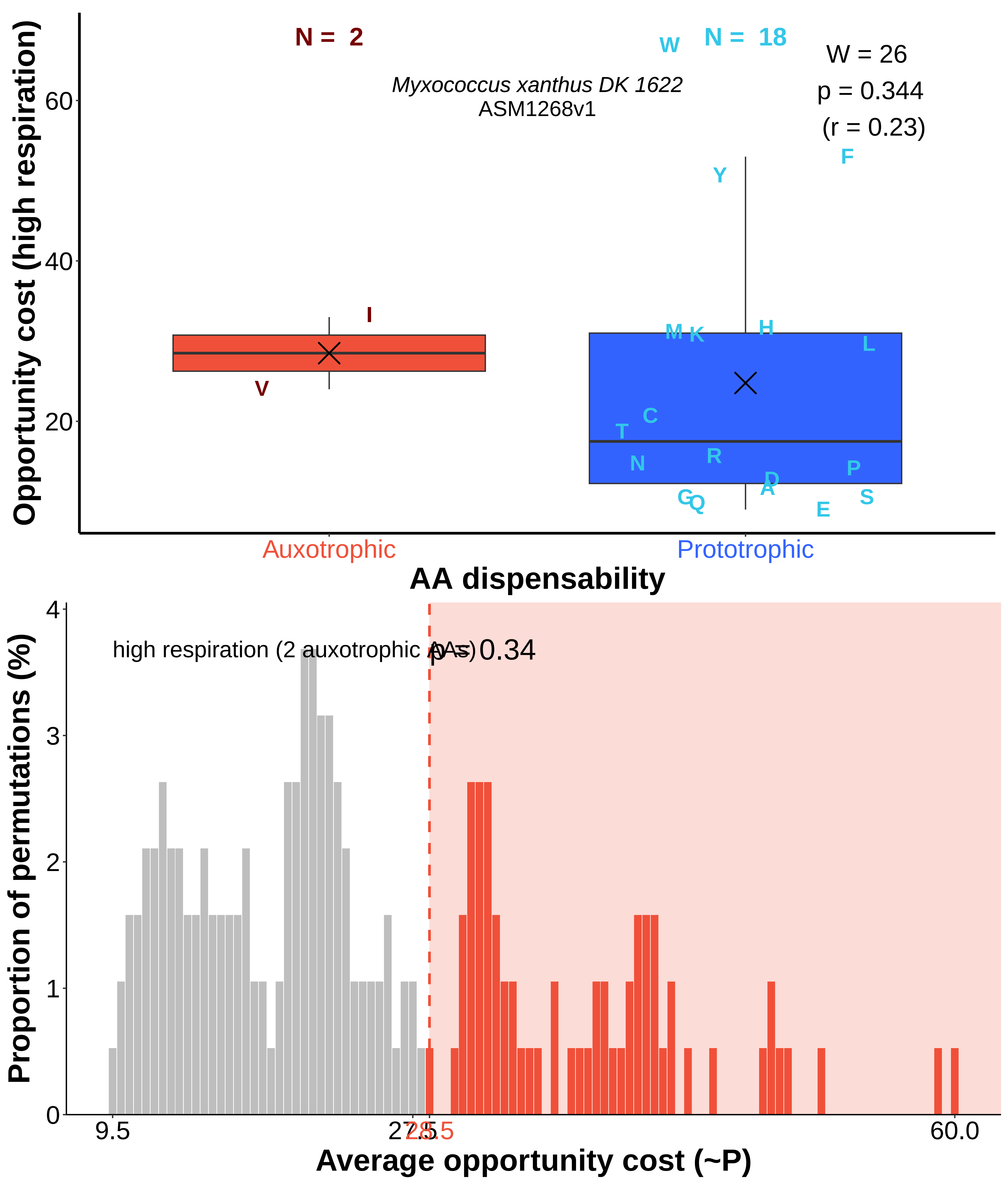

N = 2
N = 18
W = 26
p = 0.344
(r = 0.23)
Myxococcus xanthus DK 1622
ASM1268v1
Opportunity cost (high respiration)
60
40
20
I
V
W
Y
F
M K
H
L
C
T
N
R
D
A
P
G Q
E
S
Auxotrophic
Prototrophic
AA dispensability
high respiration (2 auxotrophic AAs)
p = 0.34
Proportion of permutations (%)
4
3
2
1
0
9.5
27.5
28.5
60.0
Average opportunity cost (~P)

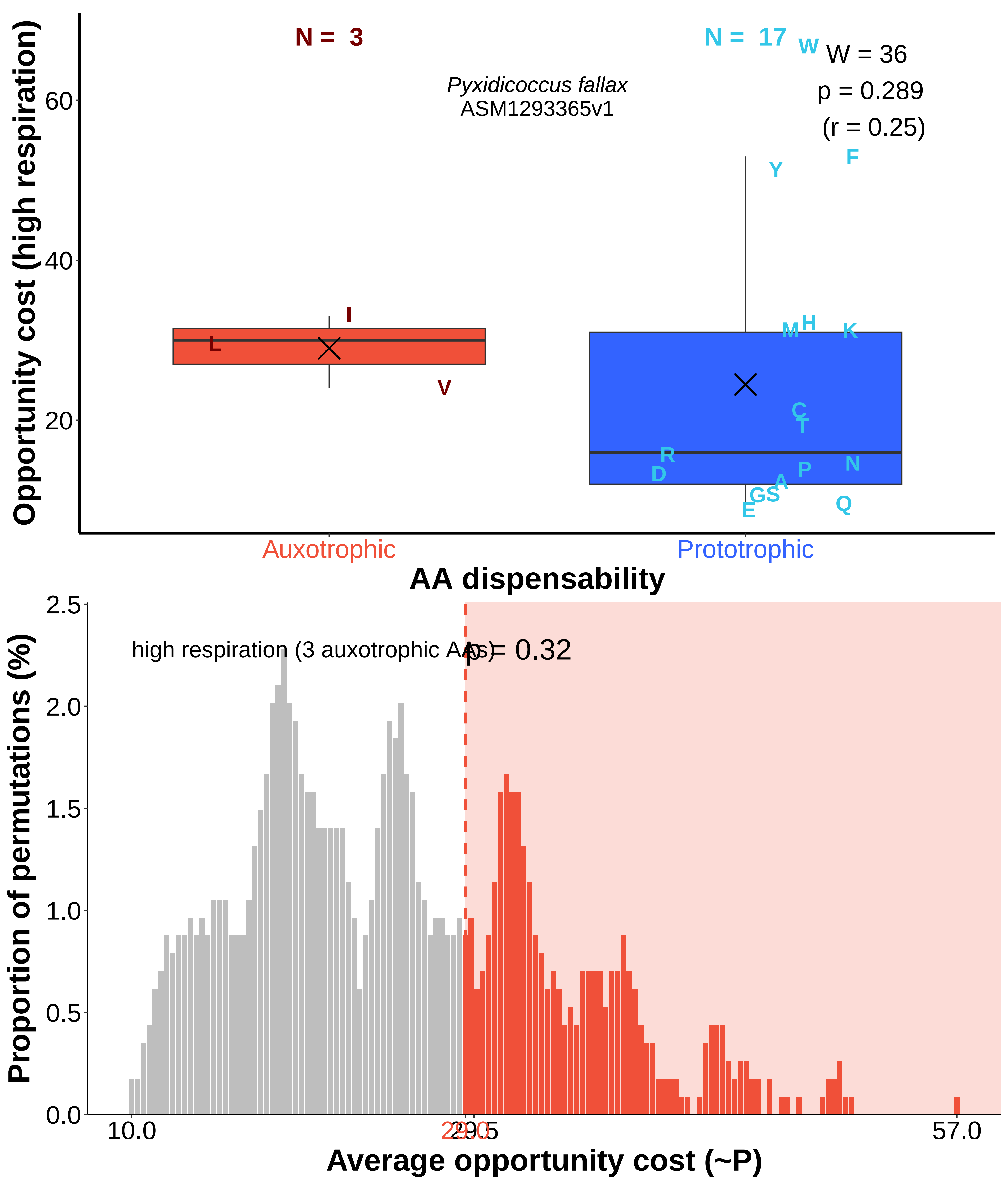

N = 3
N = 17
W = 36
p = 0.289
(r = 0.25)
Pyxidicoccus fallax
ASM1293365v1
Opportunity cost (high respiration)
60
40
20
Auxotrophic
Prototrophic
AA dispensability
high respiration (3 auxotrophic AAs)
p = 0.32
Proportion of permutations (%)
2.5
2.0
1.5
1.0
0.5
0.0
10.0
29.0
29.5
57.0
Average opportunity cost (~P)

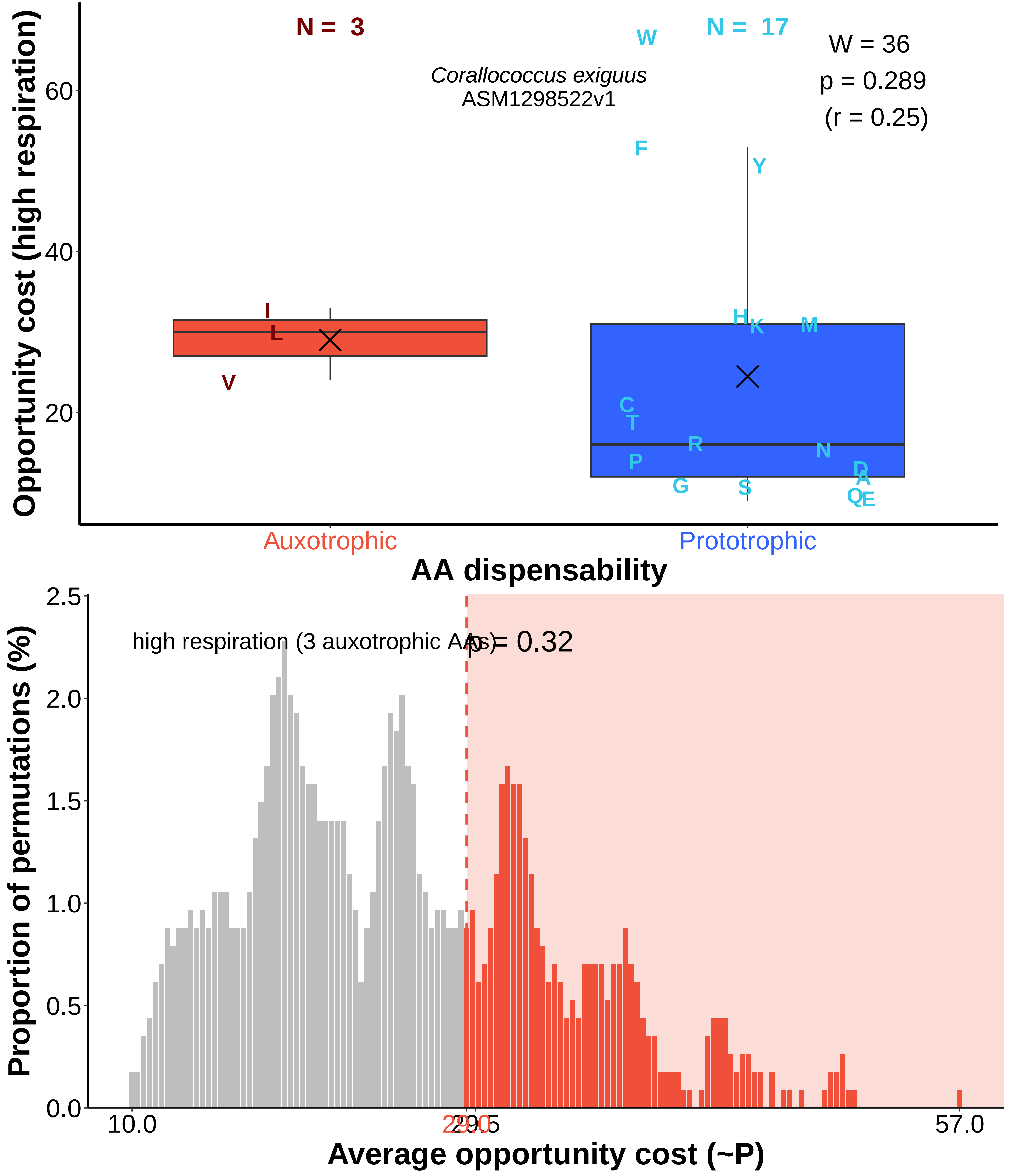

N = 3
N = 17
W = 36
p = 0.289
(r = 0.25)
Corallococcus exiguus
ASM1298522v1
Opportunity cost (high respiration)
60
40
20
I
L
V
W
F
Y
H
K
M
C
T
R
N
P
D
A
G
S
Q
E
Auxotrophic
Prototrophic
AA dispensability
Proportion of permutations (%)
2.5
2.0
1.5
1.0
0.5
0.0
high respiration (3 auxotrophic AAs)
p = 0.32
10.0
29.0
29.5
57.0
Average opportunity cost (~P)

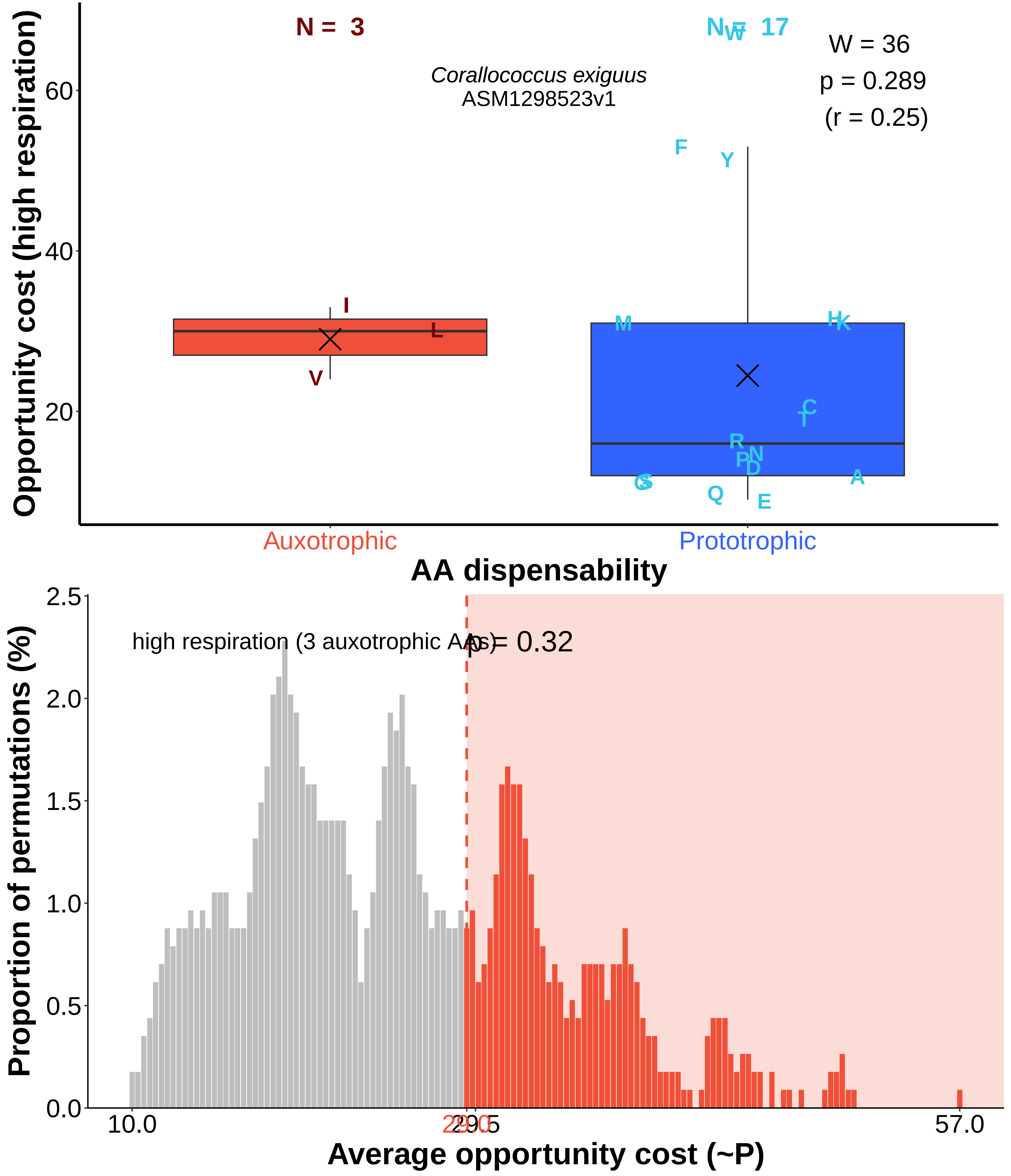
N = 3
N = 17
W = 36
p = 0.289
(r = 0.25)
Corallococcus exiguus
ASM1298523v1
Opportunity cost (high respiration)
60
40
20
I
L
V
F
Y
M
H
K
C
T
R
N
P
D
G
S
Q
E
A
W
Auxotrophic
Prototrophic
AA dispensability
high respiration (3 auxotrophic AAs)
p = 0.32
Proportion of permutations (%)
2.5
2.0
1.5
1.0
0.5
0.0
10.0
29.0
29.5
57.0
Average opportunity cost (~P)

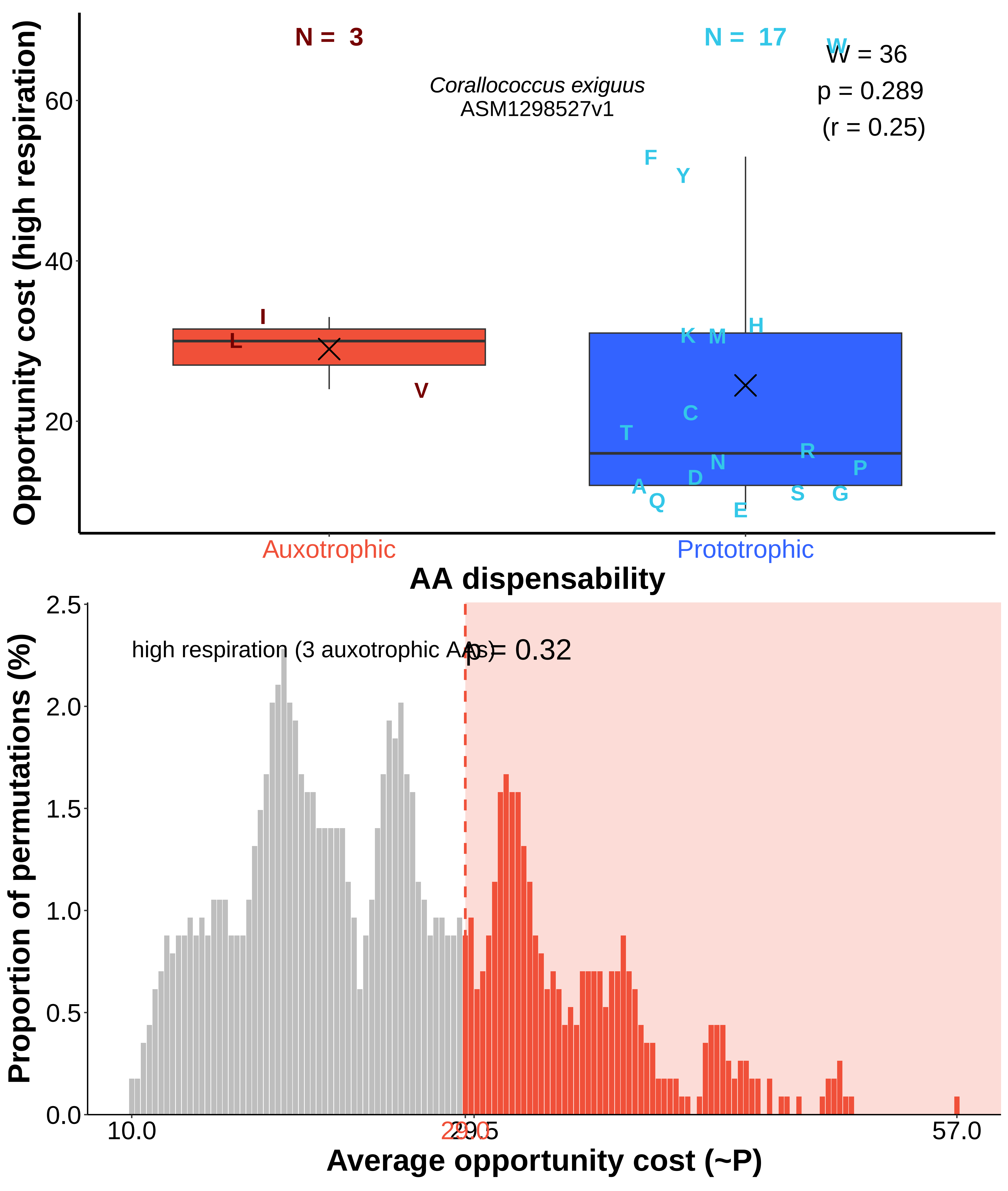

N = 3
N = 17
W = 36
p = 0.289
(r = 0.25)
Corallococcus exiguus
ASM1298527v1
Opportunity cost (high respiration)
60
40
20
I
L
V
F
Y
H
K
M
C
T
R
N
P
D
A
Q
S
G
E
W
Auxotrophic
Prototrophic
AA dispensability
Proportion of permutations (%)
2.5
2.0
1.5
1.0
0.5
0.0
high respiration (3 auxotrophic AAs)
p = 0.32
10.0
29.0
29.5
57.0
Average opportunity cost (~P)

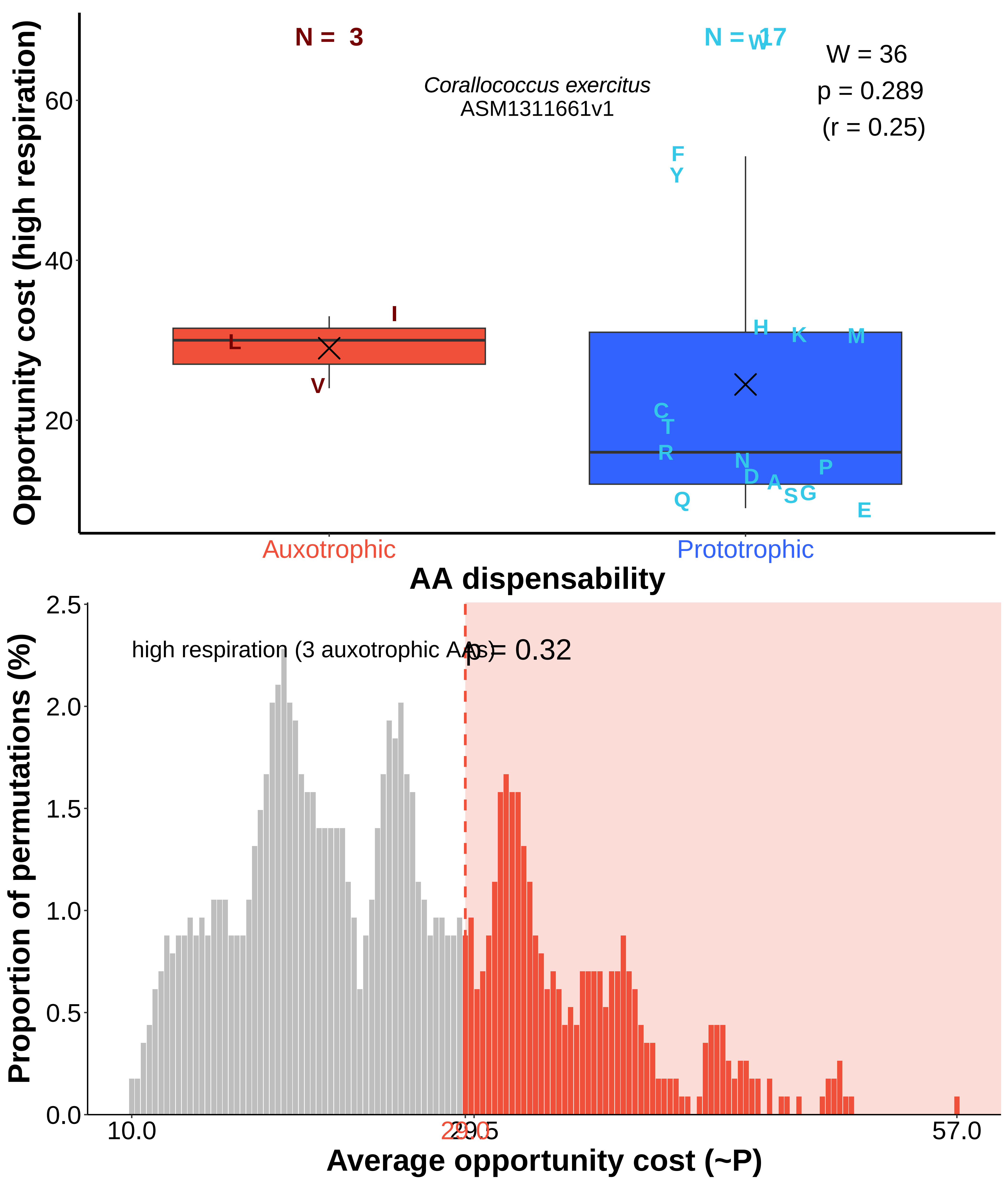
N = 3
N = 17
W
W = 36
p = 0.289
(r = 0.25)
Corallococcus exercitus
ASM1311661v1
Opportunity cost (high respiration)
60
40
20
F
Y
I
L
V
H
K
M
C
T
R
N
D
A
P
S
G
Q
E
Auxotrophic
Prototrophic
AA dispensability
high respiration (3 auxotrophic AAs)
p = 0.32
Proportion of permutations (%)
2.5
2.0
1.5
1.0
0.5
0.0
10.0
29.0
29.5
57.0
Average opportunity cost (~P)

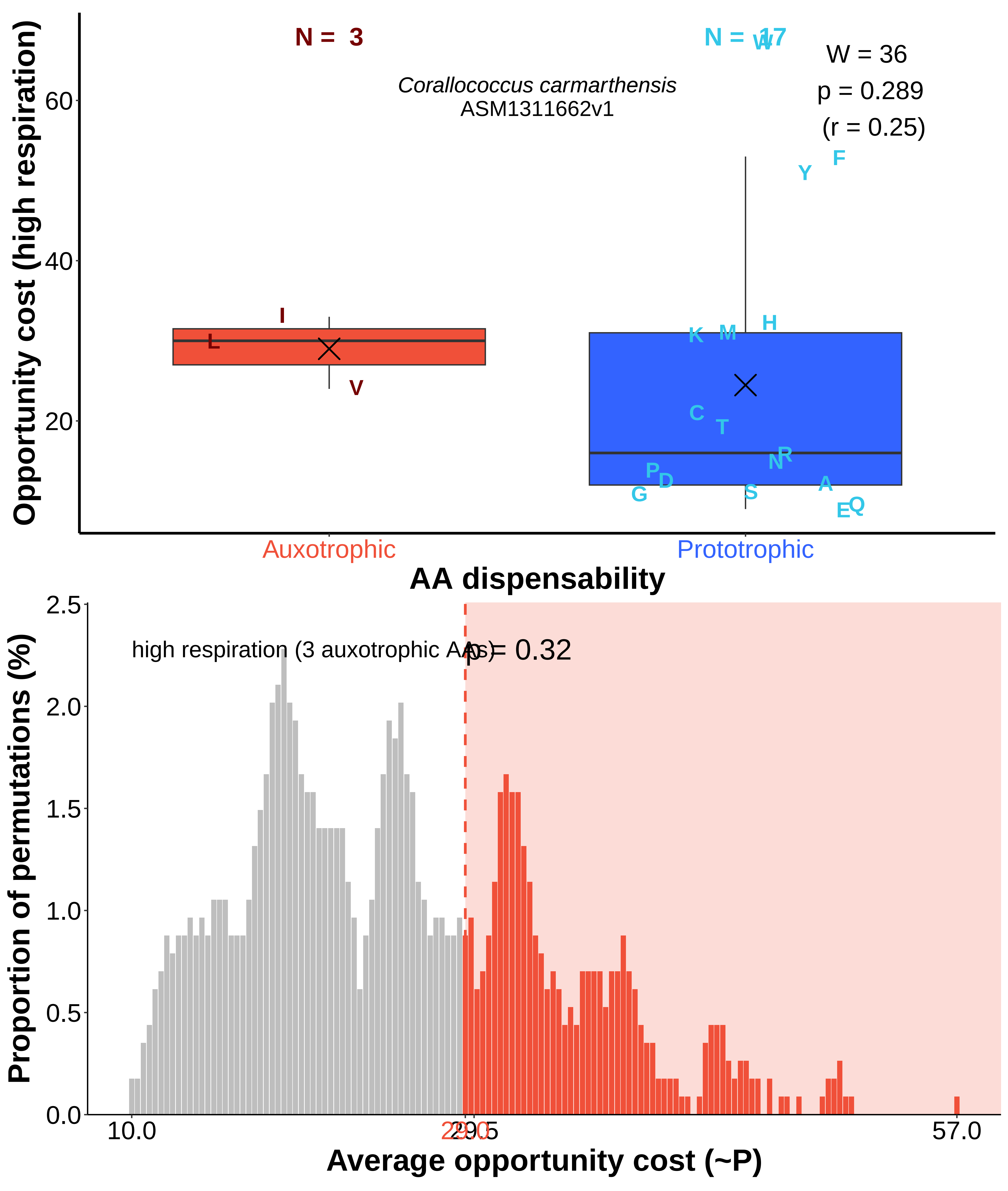

N = 3
N = 17
W
W = 36
p = 0.289
(r = 0.25)
Corallococcus carmarthensis
ASM1311662v1
Opportunity cost (high respiration)
60
40
20
I
L
V
F
Y
H
K
M
C
T
R
N
P
D
G
S
A
E
Q
Auxotrophic
Prototrophic
AA dispensability
high respiration (3 auxotrophic AAs)
p = 0.32
Proportion of permutations (%)
2.5
2.0
1.5
1.0
0.5
0.0
10.0
29.0
29.5
57.0
Average opportunity cost (~P)

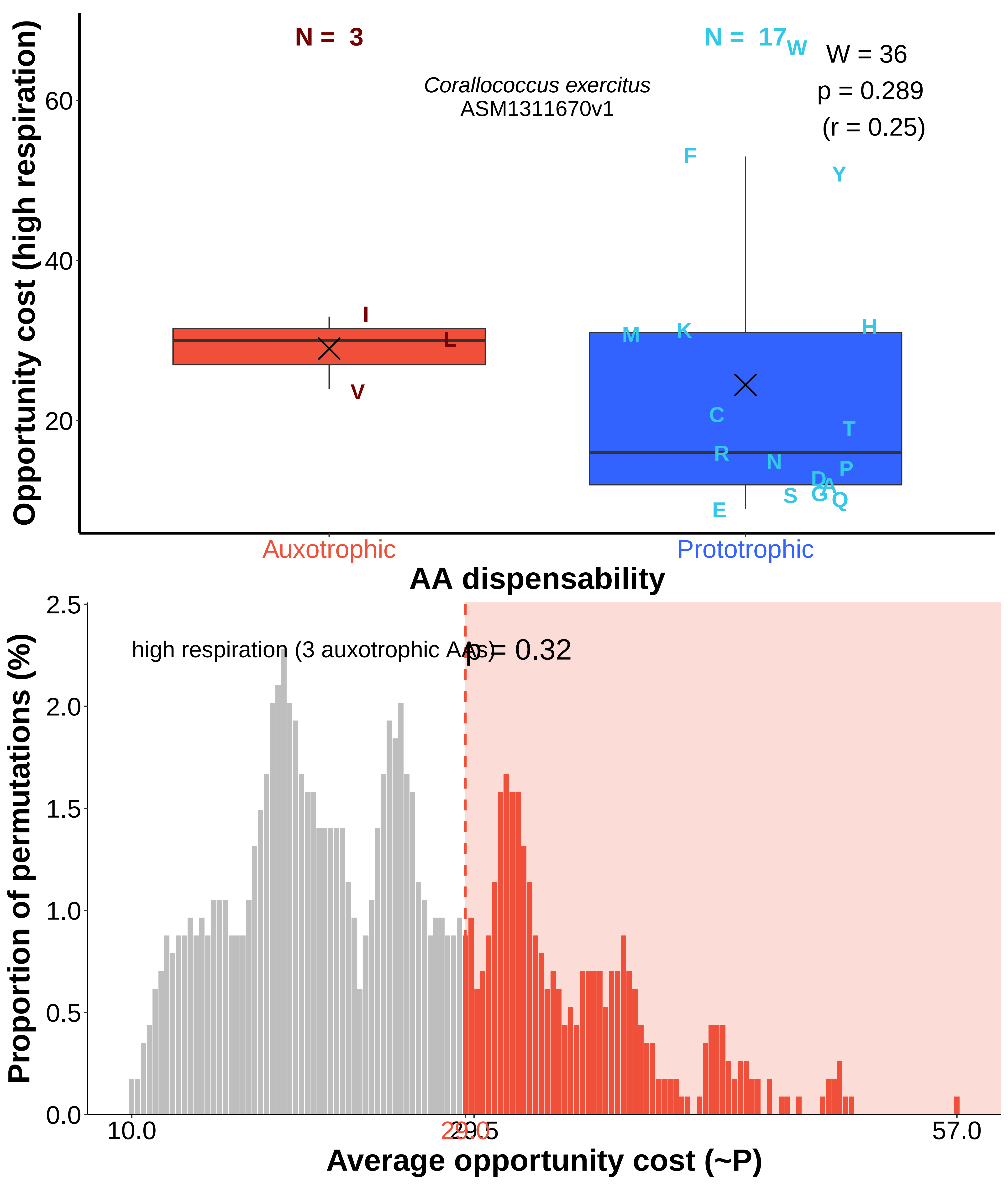

N = 3
N = 17
W = 36
p = 0.289
(r = 0.25)
Corallococcus exercitus
ASM1311670v1
Opportunity cost (high respiration)
Auxotrophic
Prototrophic
AA dispensability
high respiration (3 auxotrophic AAs)
p = 0.32
Proportion of permutations (%)
Average opportunity cost (~P)
10.0
29.0
29.5
57.0

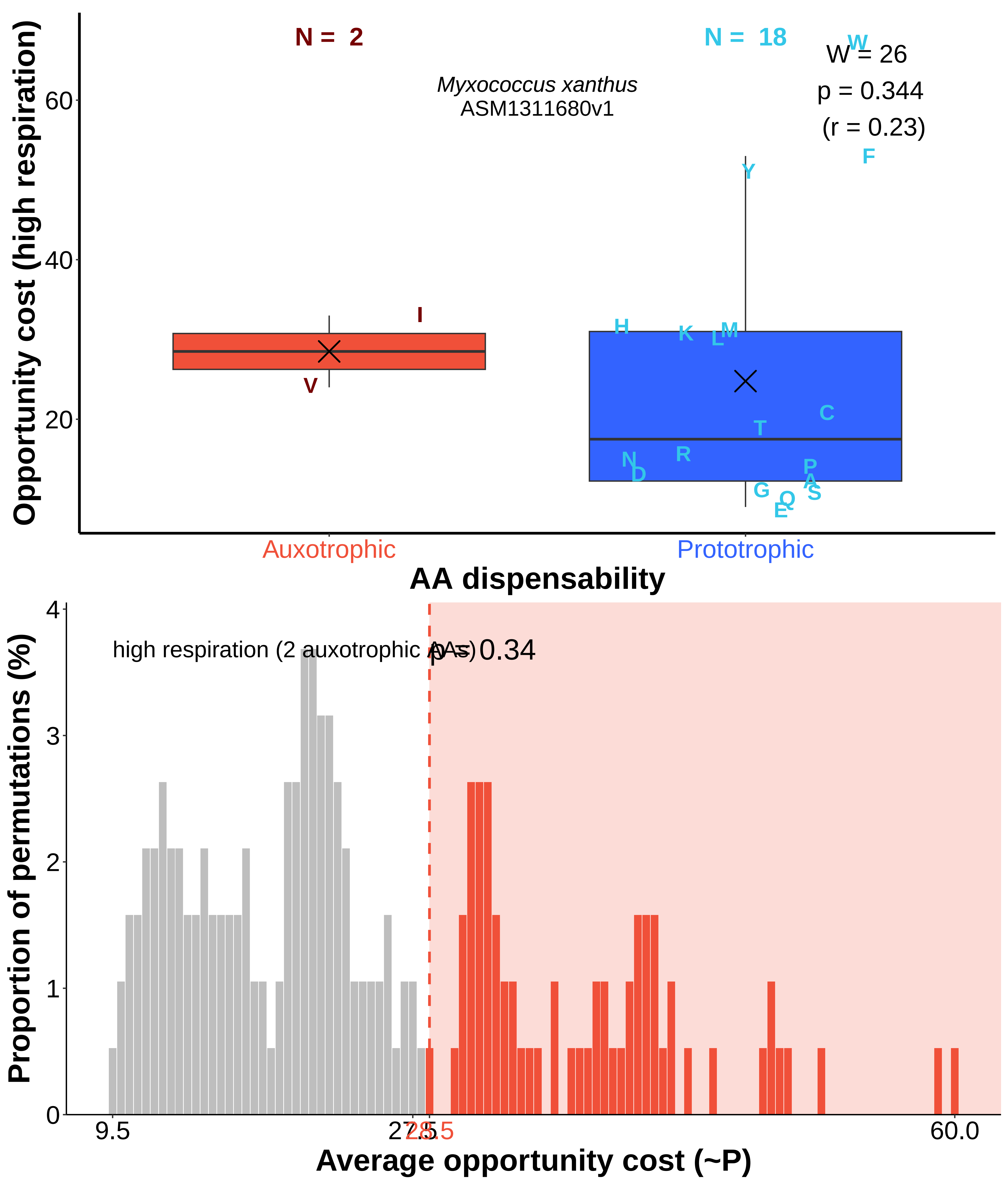

N = 2
N = 18
Myxococcus xanthus
ASM1311680v1
W = 26
p = 0.344
(r = 0.23)
Opportunity cost (high respiration)
Auxotrophic
Prototrophic
AA dispensability
high respiration (2 auxotrophic AAs)
p = 0.34
Proportion of permutations (%)
Average opportunity cost (~P)
9.5
27.5
28.5
60.0

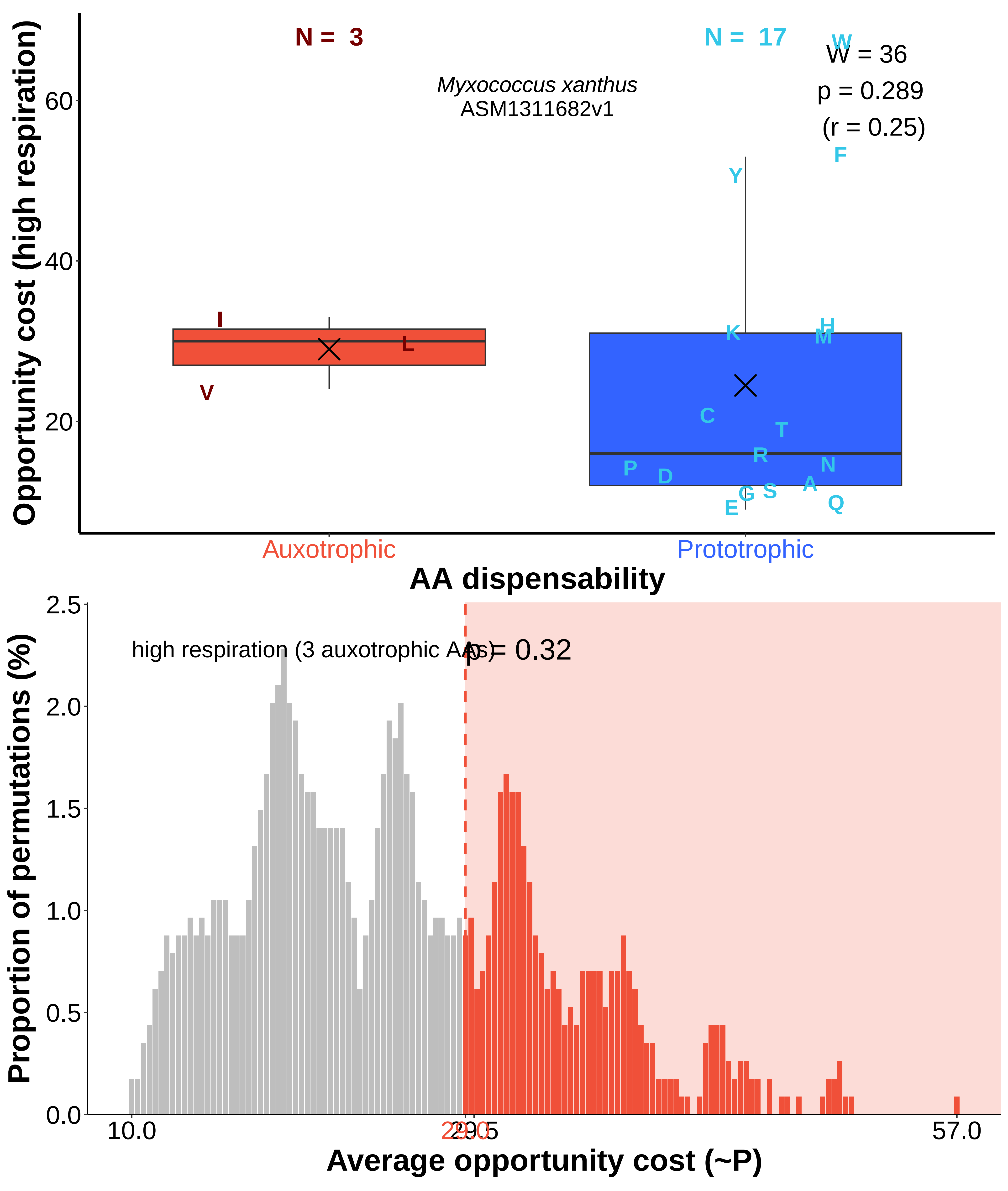
N = 3
N = 17
Myxococcus xanthus
ASM1311682v1
W = 36
p = 0.289
(r = 0.25)
W
F
Y
I
K
H
M
L
V
C
T
R
N
P
D
A
G
S
E
Q
60
40
20
Opportunity cost (high respiration)
Auxotrophic
Prototrophic
AA dispensability
2.5
2.0
1.5
1.0
0.5
0.0
high respiration (3 auxotrophic AAs)
p = 0.32
Proportion of permutations (%)
10.0
29.0
29.5
57.0
Average opportunity cost (~P)

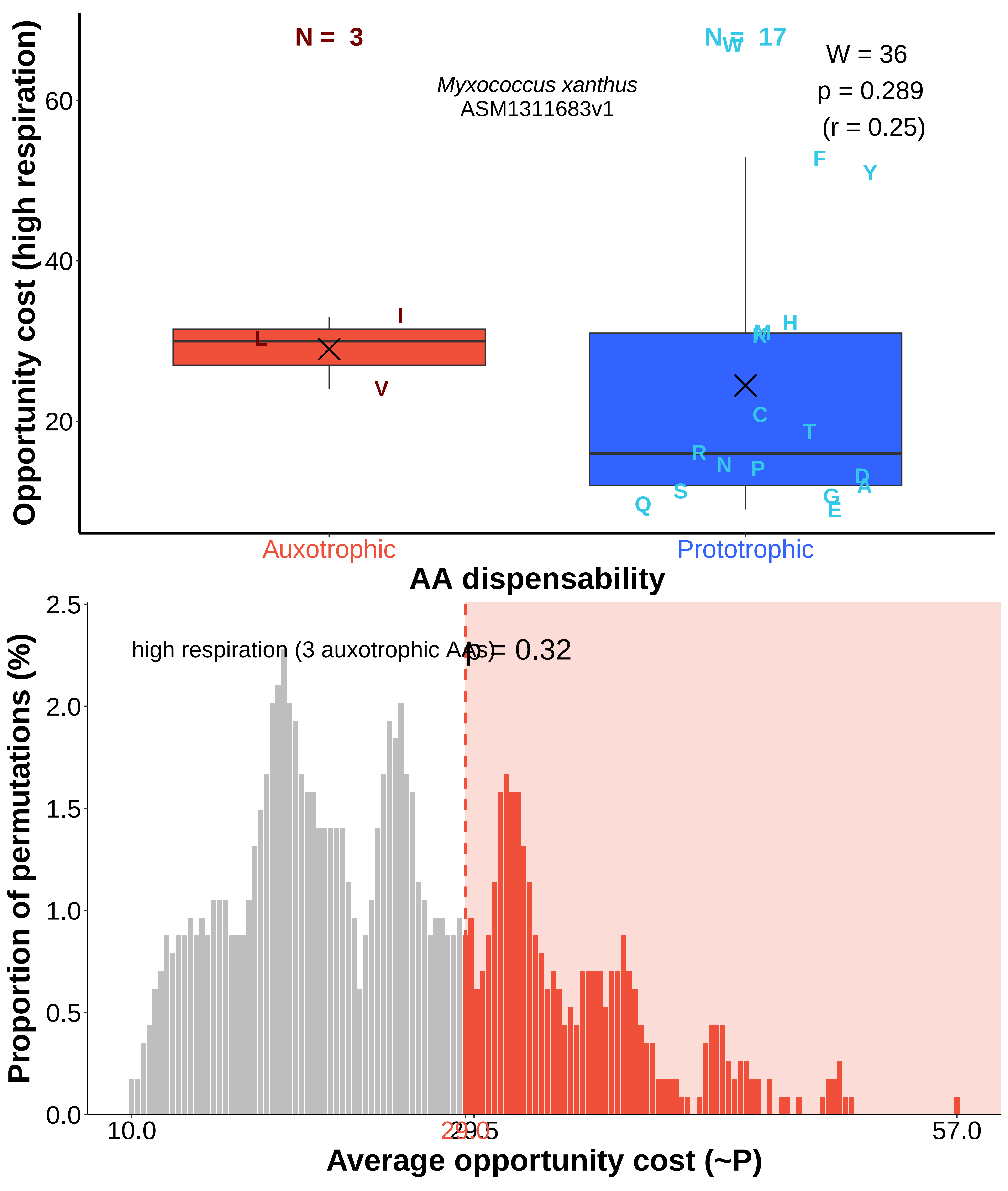

N = 3
N = 17
W
W = 36
p = 0.289
(r = 0.25)
Myxococcus xanthus
ASM1311683v1
Opportunity cost (high respiration)
60
40
20
L
I
V
F
Y
H
M
K
C
T
R
N
P
S
Q
D
A
G
E
Auxotrophic
Prototrophic
AA dispensability
high respiration (3 auxotrophic AAs)
p = 0.32
Proportion of permutations (%)
2.5
2.0
1.5
1.0
0.5
0.0
10.0
29.0
29.5
57.0
Average opportunity cost (~P)

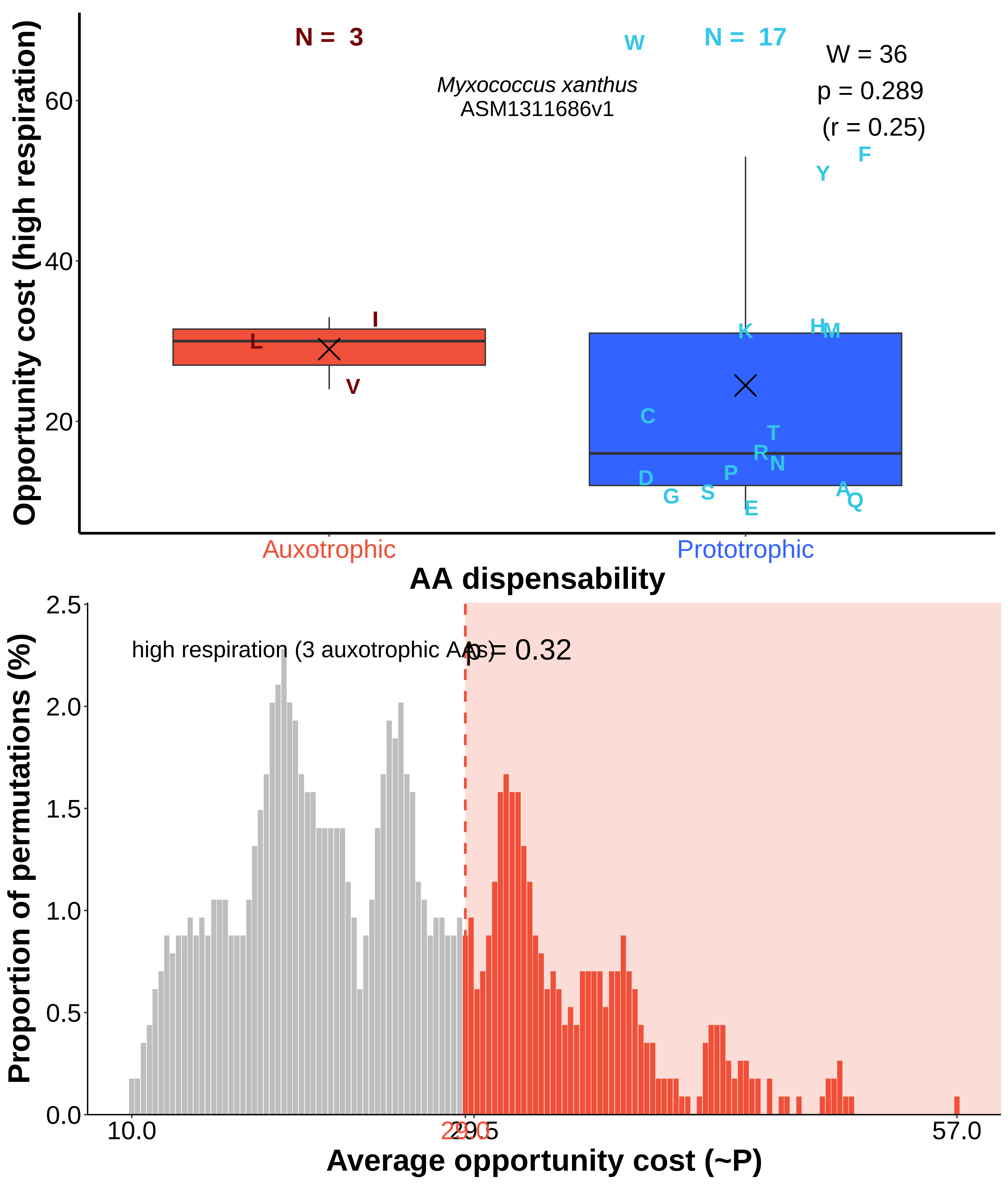
N = 3
W
N = 17
W = 36
p = 0.289
(r = 0.25)
Myxococcus xanthus
ASM1311686v1
Opportunity cost (high respiration)
60
40
20
I
L
V
K
H
M
F
Y
C
T
R
N
P
D
G
S
E
A
Q
Auxotrophic
Prototrophic
AA dispensability
high respiration (3 auxotrophic AAs)
p = 0.32
Proportion of permutations (%)
2.5
2.0
1.5
1.0
0.5
0.0
10.0
29.0
29.5
57.0
Average opportunity cost (~P)

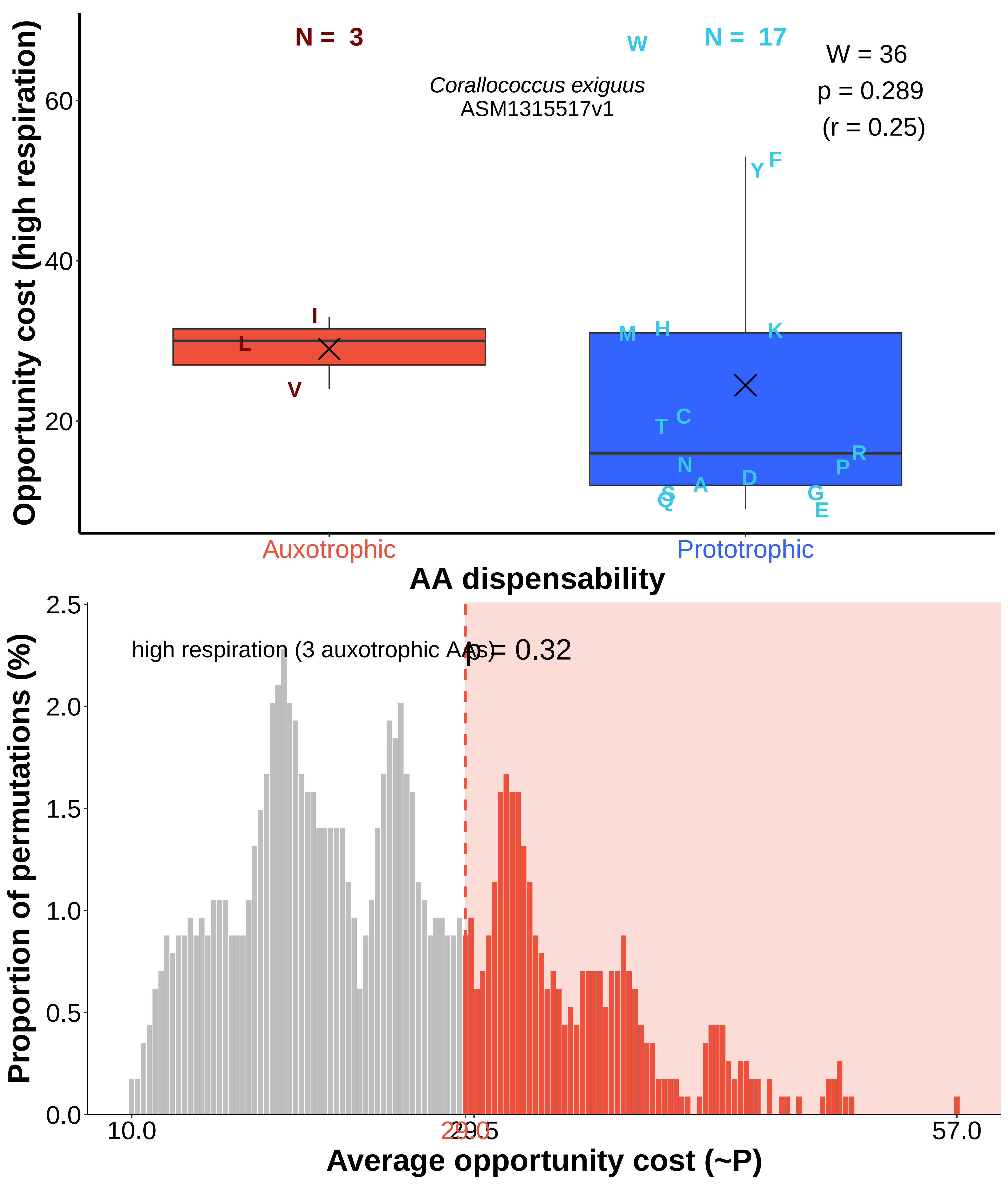
N = 3
N = 17
W
W = 36
p = 0.289
(r = 0.25)
Corallococcus exiguus
ASM1315517v1
Opportunity cost (high respiration)
60
40
20
I
L
V
M
H
K
Y
F
T
C
N
A
D
S
Q
G
E
P
R
Auxotrophic
Prototrophic
AA dispensability
Proportion of permutations (%)
2.5
2.0
1.5
1.0
0.5
0.0
high respiration (3 auxotrophic AAs)
p = 0.32
10.0
29.0
29.5
57.0
Average opportunity cost (~P)

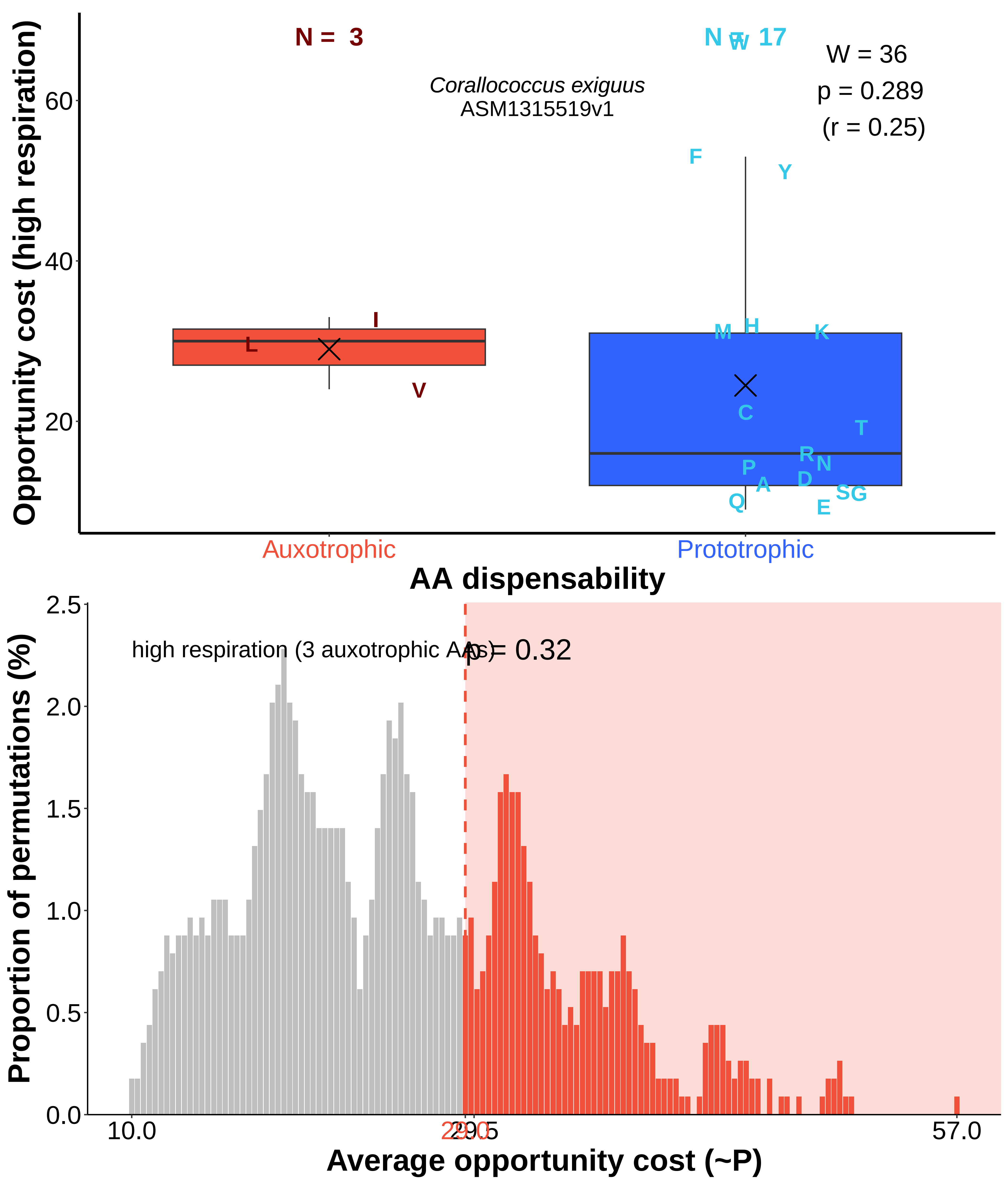

N = 3
N = 17
W = 36
p = 0.289
(r = 0.25)
Corallococcus exiguus
ASM1315519v1
Opportunity cost (high respiration)
Auxotrophic
Prototrophic
AA dispensability
high respiration (3 auxotrophic AAs)
p = 0.32
Proportion of permutations (%)
Average opportunity cost (~P)

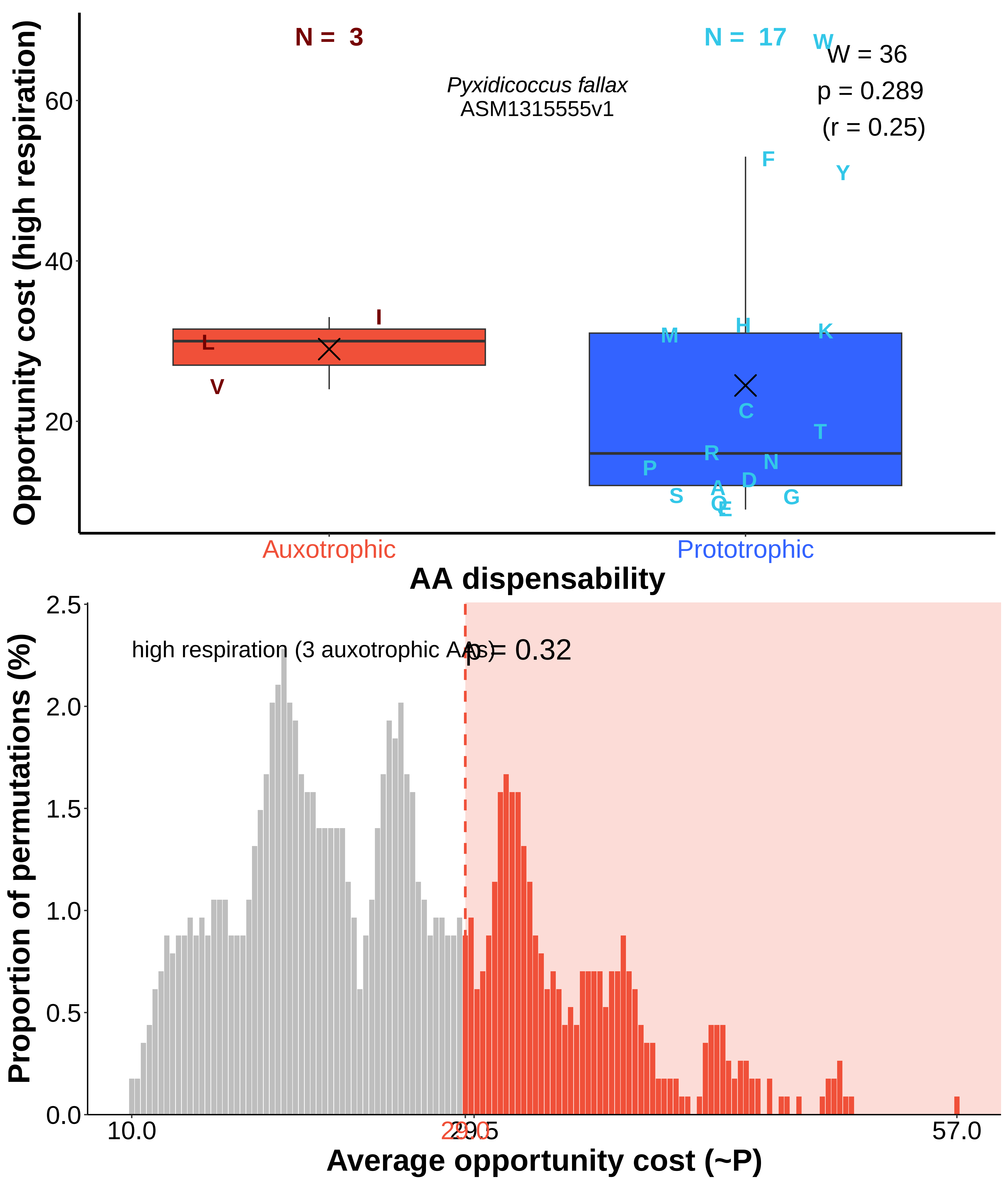
N = 3
N = 17
W = 36
p = 0.289
(r = 0.25)
Pyxidicoccus fallax
ASM1315555v1
Opportunity cost (high respiration)
60
40
20
F
Y
W
I
L
V
M
H
K
C
T
R
N
P
D
A
S
Q
E
G
Auxotrophic
Prototrophic
AA dispensability
high respiration (3 auxotrophic AAs)
p = 0.32
Proportion of permutations (%)
2.5
2.0
1.5
1.0
0.5
0.0
10.0
29.0
29.5
57.0
Average opportunity cost (~P)

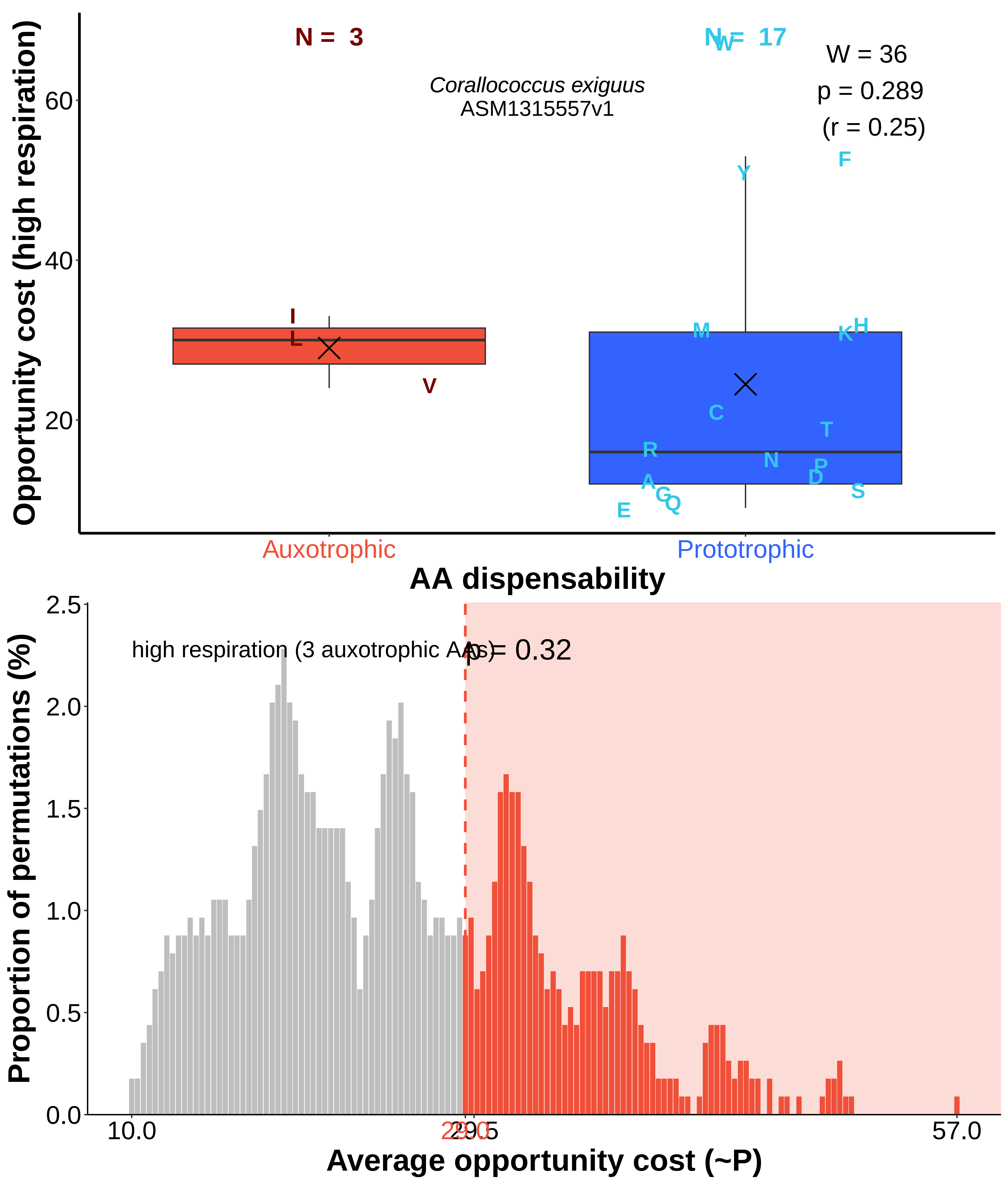
N = 3
N = 17
W
W = 36
p = 0.289
(r = 0.25)
Corallococcus exiguus
ASM1315557v1
Opportunity cost (high respiration)
60
40
20
I
L
V
Y
F
M
K
H
C
T
R
N
P
D
A
G
Q
S
E
Auxotrophic
Prototrophic
AA dispensability
2.5
2.0
1.5
1.0
0.5
0.0
high respiration (3 auxotrophic AAs)
p = 0.32
Proportion of permutations (%)
10.0
29.0
29.5
57.0
Average opportunity cost (~P)

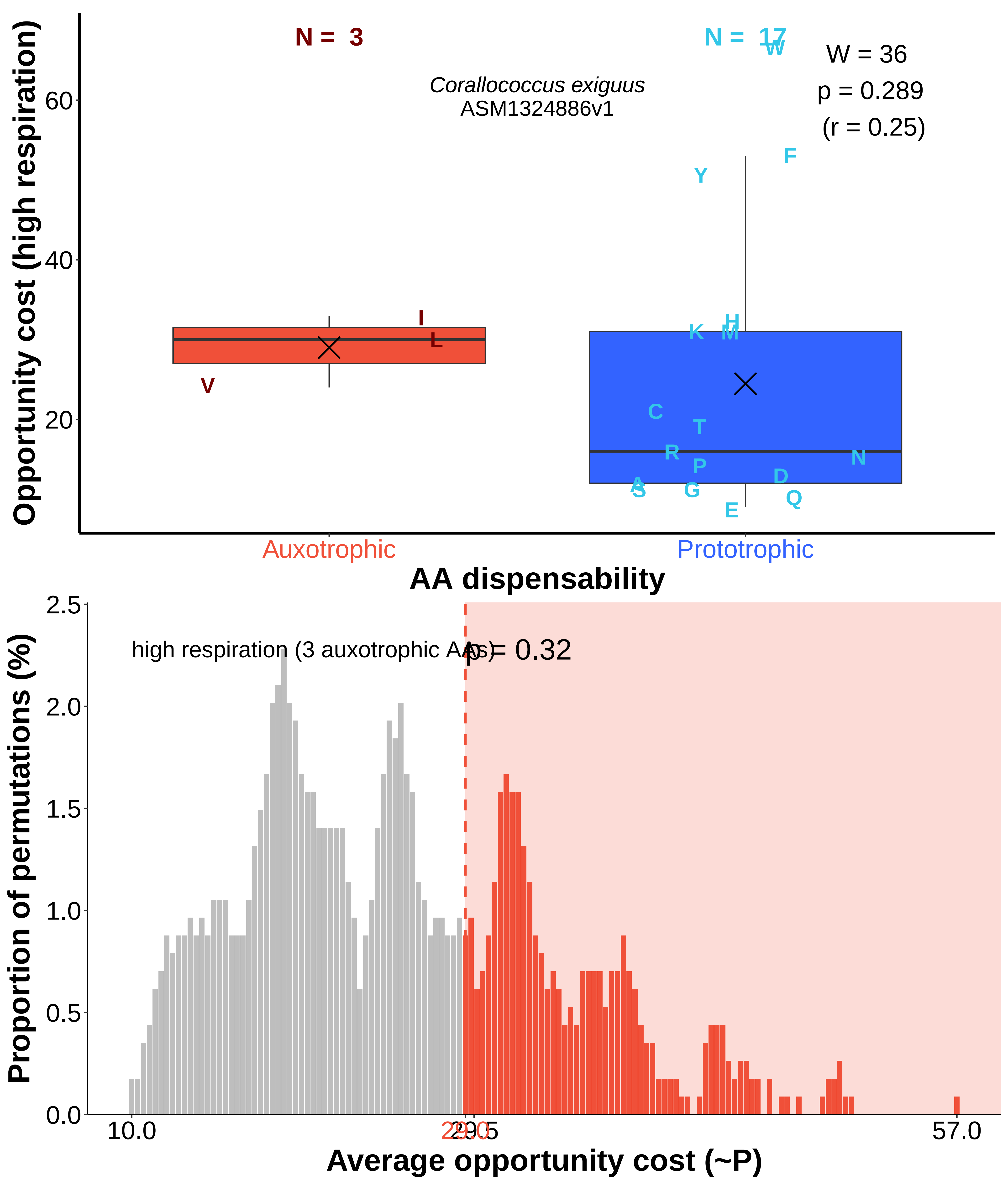

N = 3
N = 17
W = 36
p = 0.289
(r = 0.25)
Corallococcus exiguus
ASM1324886v1
Opportunity cost (high respiration)
60
40
20
I
L
V
W
F
Y
H
K
M
C
T
R
P
N
D
A
S
G
Q
E
Auxotrophic
Prototrophic
AA dispensability
high respiration (3 auxotrophic AAs)
p = 0.32
Proportion of permutations (%)
2.5
2.0
1.5
1.0
0.5
0.0
10.0
29.0
29.5
57.0
Average opportunity cost (~P)

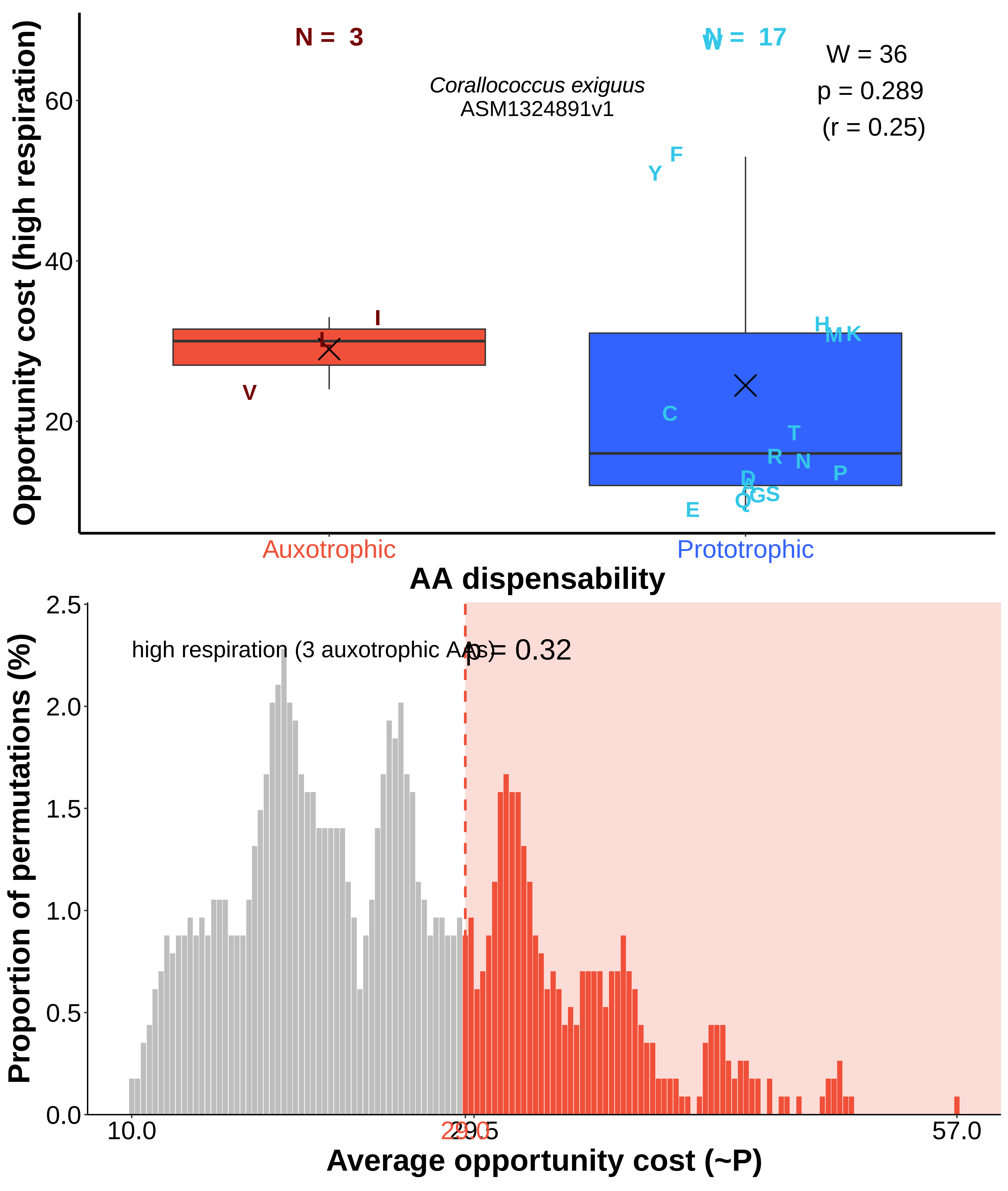
N = 3
N = 17
W = 36
p = 0.289
(r = 0.25)
Corallococcus exiguus
ASM1324891v1
Opportunity cost (high respiration)
Auxotrophic
Prototrophic
AA dispensability
high respiration (3 auxotrophic AAs)
p = 0.32
Proportion of permutations (%)
Average opportunity cost (~P)
10.0
29.0
29.5
57.0

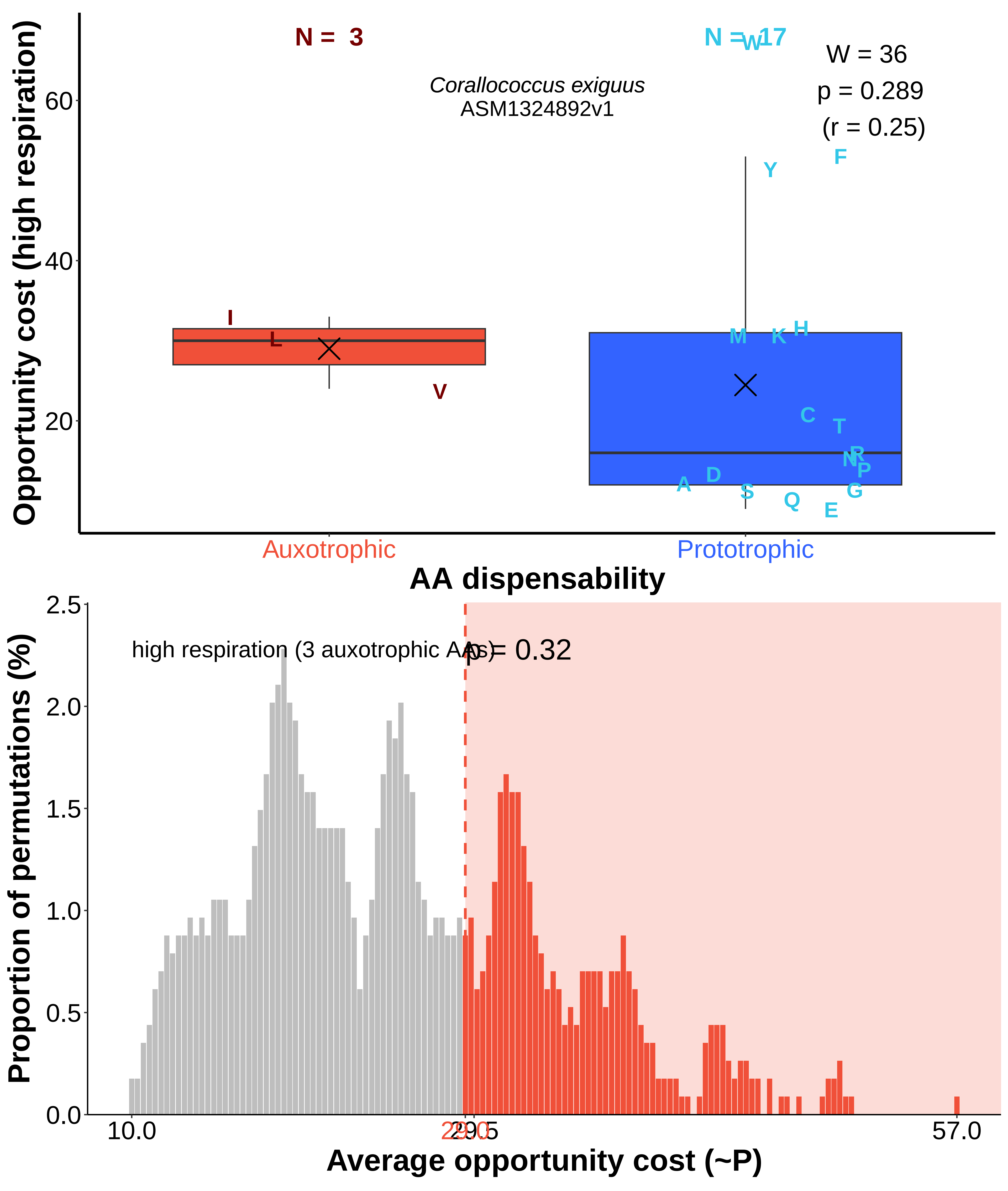

N = 3
N = 17
W
W = 36
p = 0.289
(r = 0.25)
Corallococcus exiguus
ASM1324892v1
Opportunity cost (high respiration)
60
40
20
I
L
V
Y
F
M
K
H
C
T
R
N
P
D
A
S
Q
G
E
Auxotrophic
Prototrophic
AA dispensability
2.5
2.0
1.5
1.0
0.5
0.0
high respiration (3 auxotrophic AAs)
p = 0.32
Proportion of permutations (%)
10.0
29.0
29.5
57.0
Average opportunity cost (~P)

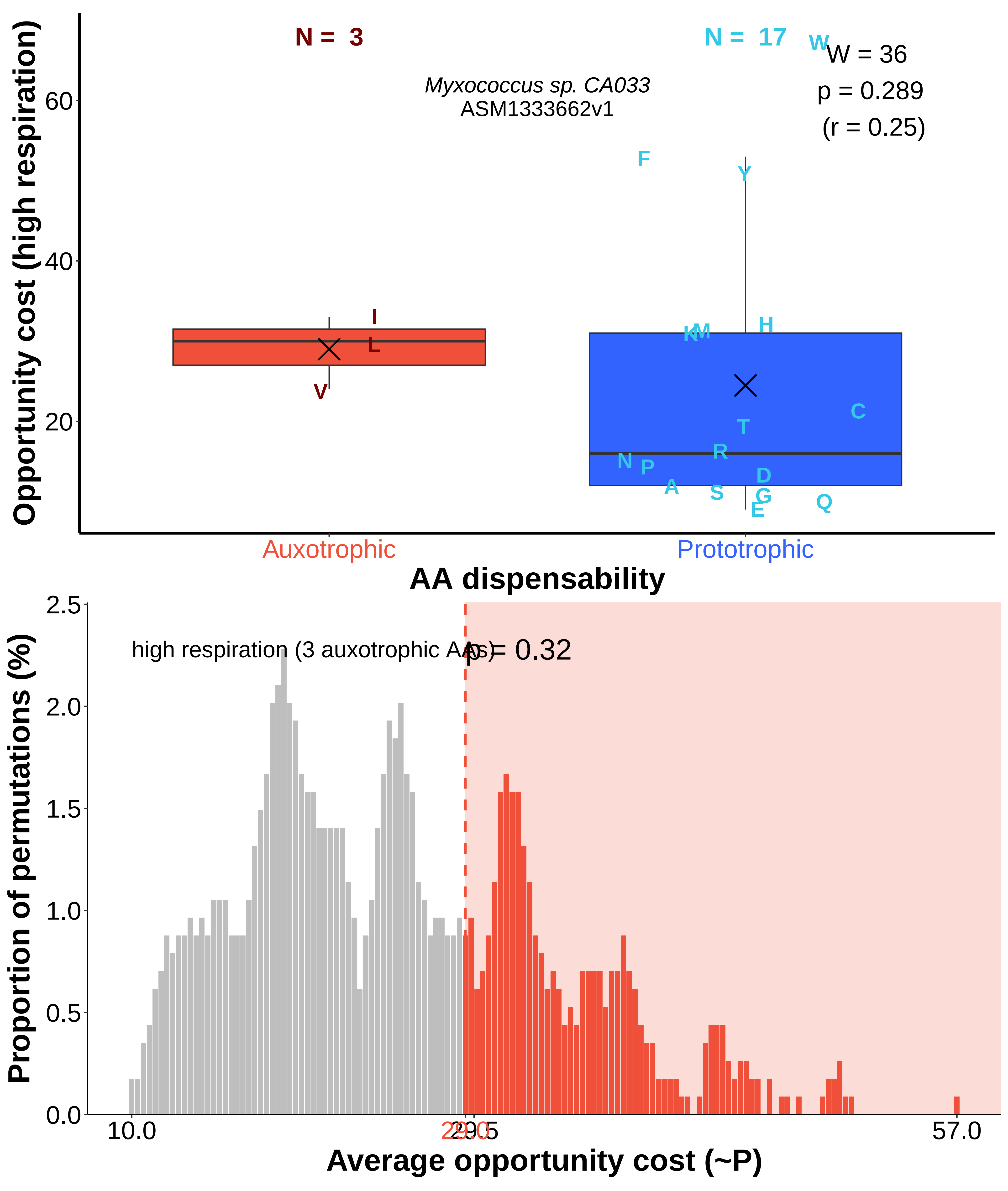

N = 3
N = 17
W
W = 36
p = 0.289
(r = 0.25)
Myxococcus sp. CA033
ASM1333662v1
Opportunity cost (high respiration)
F
Y
I
L
V
K
M
H
C
T
R
N
P
A
S
D
G
E
Q
60
40
20
Auxotrophic
Prototrophic
AA dispensability
high respiration (3 auxotrophic AAs)
p = 0.32
Proportion of permutations (%)
2.5
2.0
1.5
1.0
0.5
0.0
10.0
29.0
29.5
57.0
Average opportunity cost (~P)

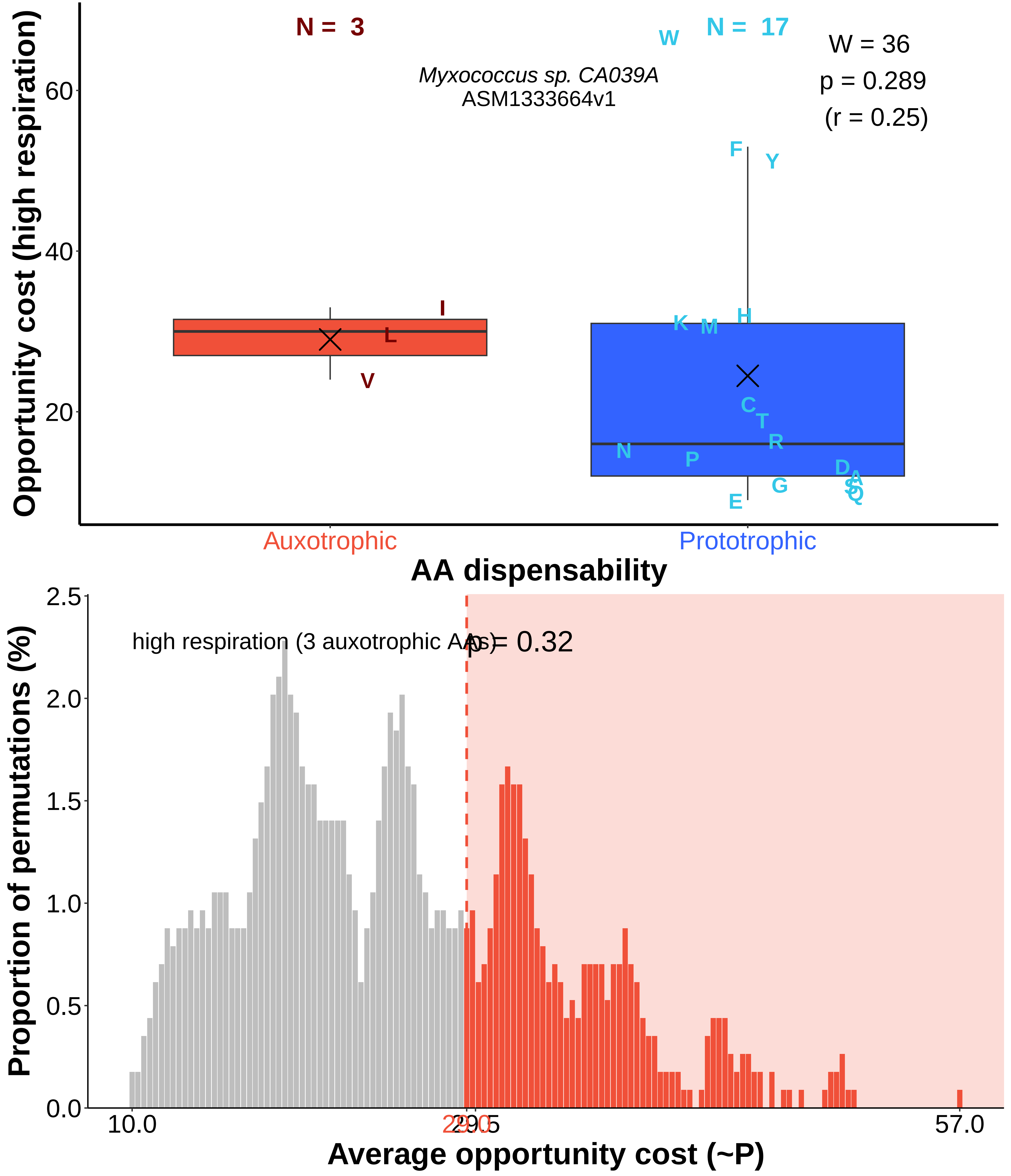

N = 3
W
N = 17
W = 36
p = 0.289
(r = 0.25)
Myxococcus sp. CA039A
ASM1333664v1
Opportunity cost (high respiration)
60
40
20
F
Y
I
L
V
K
M
H
C
T
R
N
P
D
A
S
Q
G
E
Auxotrophic
Prototrophic
AA dispensability
2.5
2.0
1.5
1.0
0.5
0.0
high respiration (3 auxotrophic AAs)
p = 0.32
Proportion of permutations (%)
10.0
29.0
29.5
57.0
Average opportunity cost (~P)

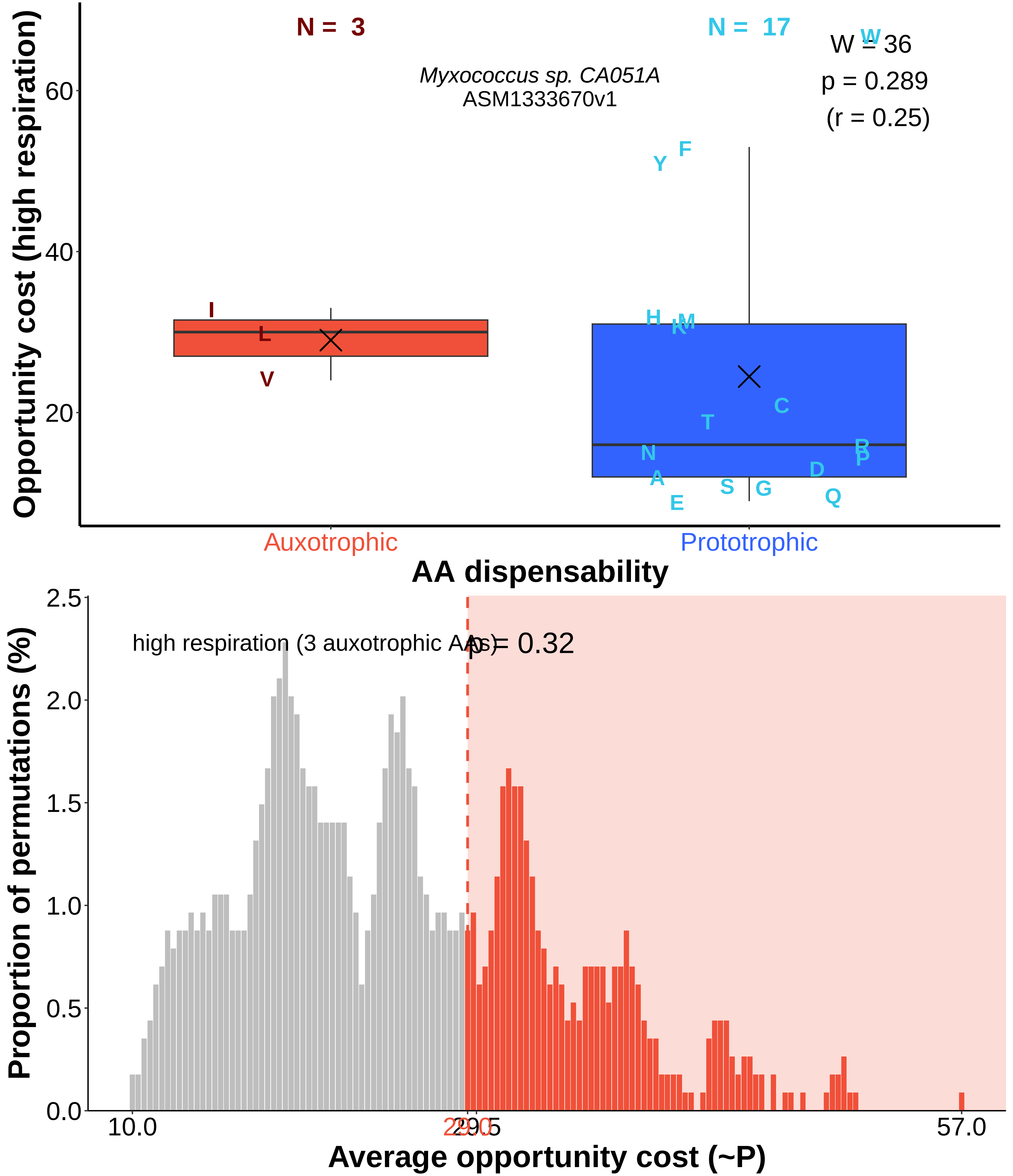

N = 3
N = 17
W = 36
p = 0.289
(r = 0.25)
Myxococcus sp. CA051A
ASM1333670v1
Opportunity cost (high respiration)
60
40
20
I
L
V
Y
F
W
H
M
K
C
T
N
R
P
D
A
S
G
E
Q
Auxotrophic
Prototrophic
AA dispensability
Proportion of permutations (%)
2.5
2.0
1.5
1.0
0.5
0.0
high respiration (3 auxotrophic AAs)
p = 0.32
10.0
29.0
29.5
57.0
Average opportunity cost (~P)

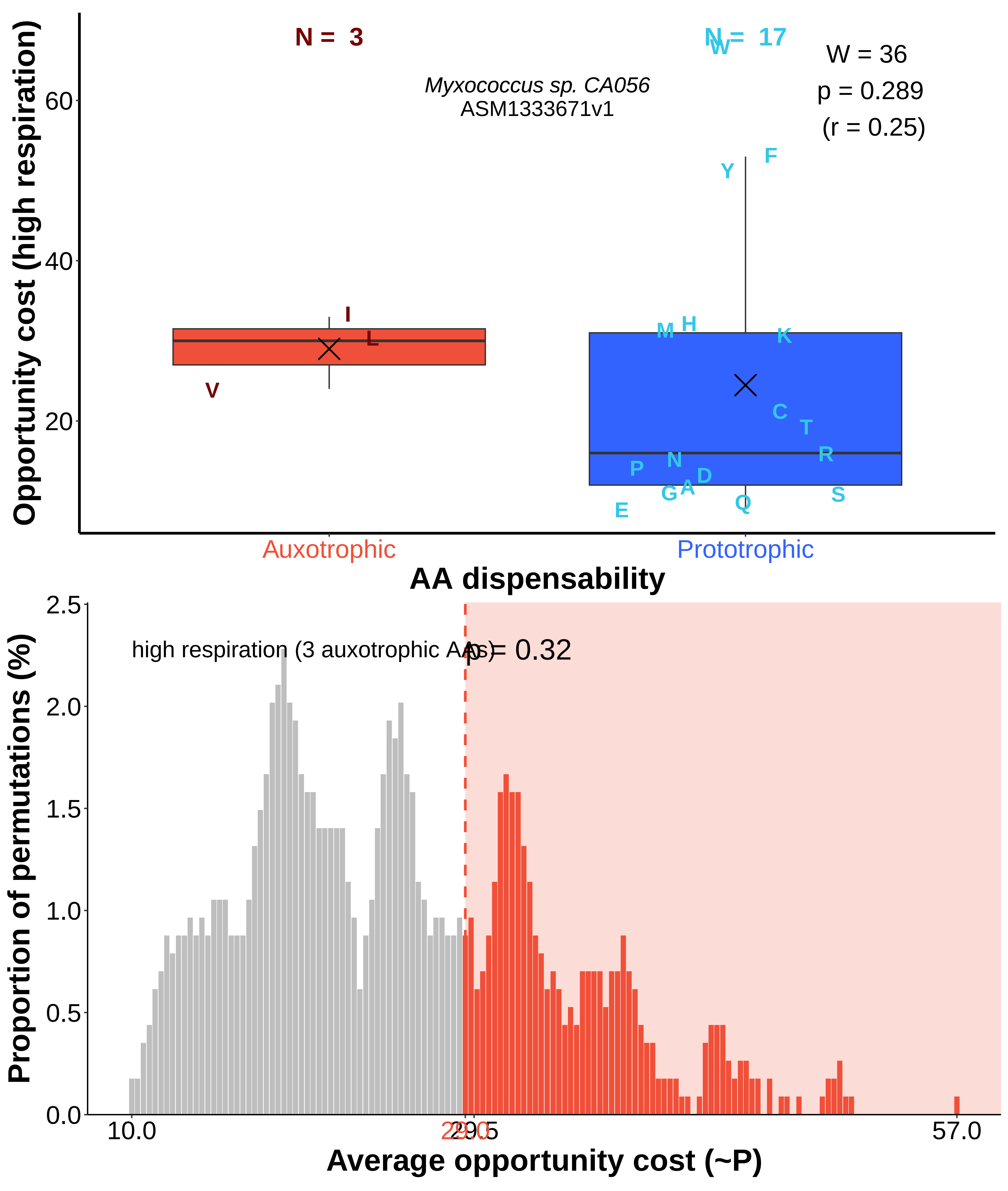

N = 3
N = 17
W = 36
p = 0.289
(r = 0.25)
Myxococcus sp. CA056
ASM1333671v1
Opportunity cost (high respiration)
Auxotrophic
Prototrophic
AA dispensability
high respiration (3 auxotrophic AAs)
p = 0.32
Proportion of permutations (%)
Average opportunity cost (~P)
10.0
29.0
57.0

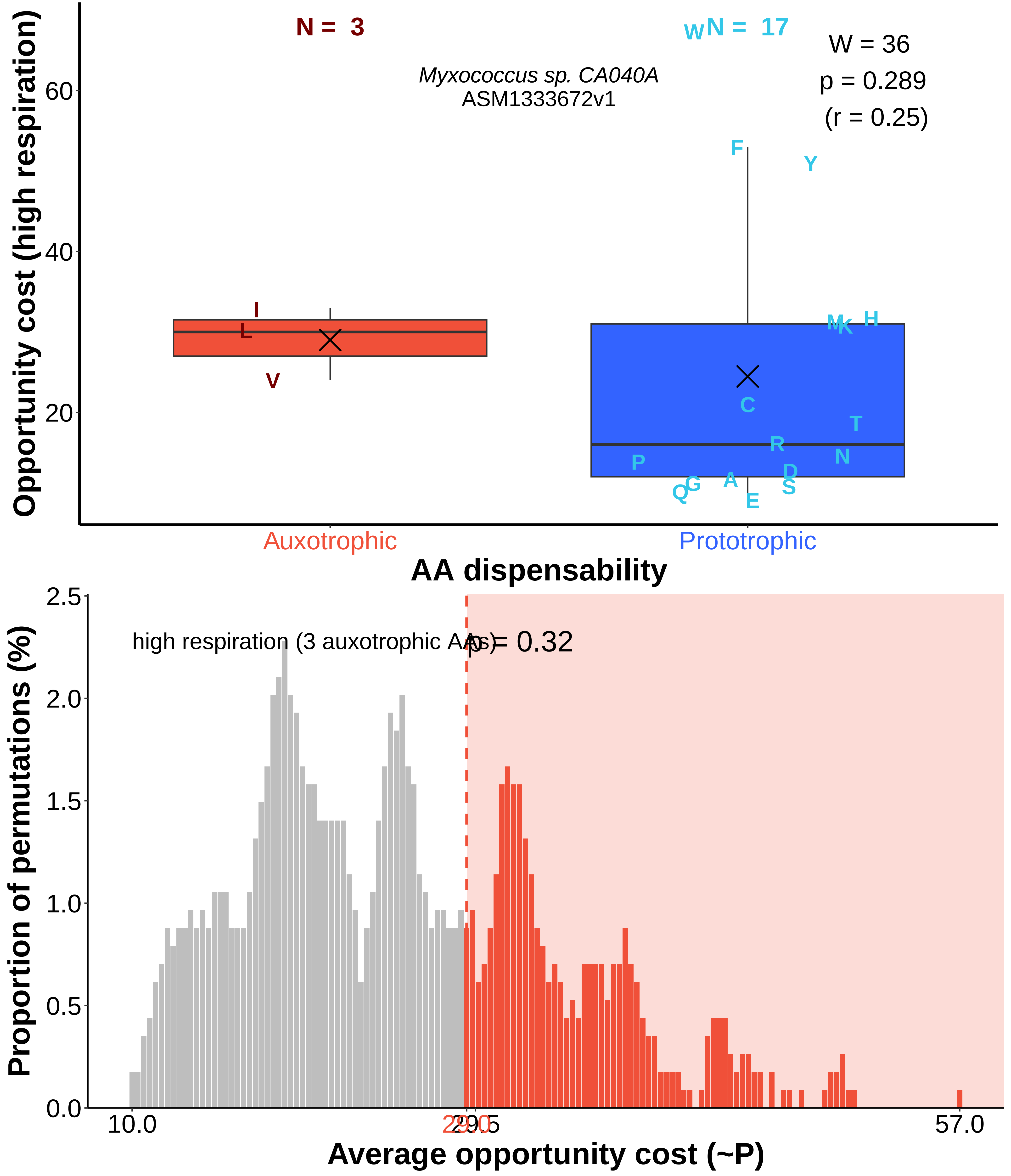

N = 3
wN = 17
W = 36
p = 0.289
(r = 0.25)
Myxococcus sp. CA040A
ASM1333672v1
Opportunity cost (high respiration)
60
40
20
I
L
V
F
Y
M
K
H
C
T
R
N
P
D
Q
G
A
S
E
Auxotrophic
Prototrophic
AA dispensability
high respiration (3 auxotrophic AAs)
p = 0.32
Proportion of permutations (%)
2.5
2.0
1.5
1.0
0.5
0.0
10.0
29.0
57.0
Average opportunity cost (~P)

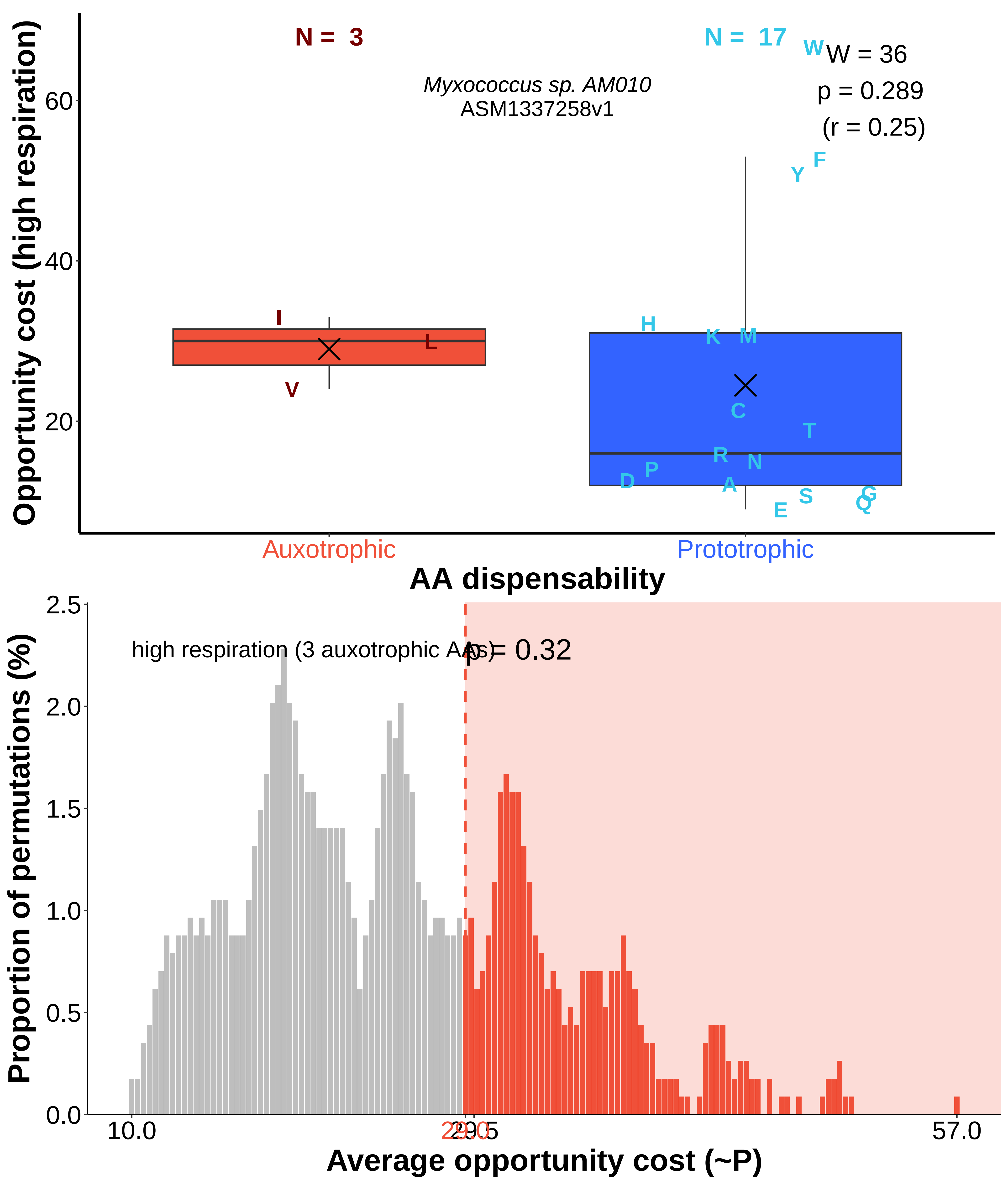

N = 3
N = 17
Myxococcus sp. AM010
ASM1337258v1
W = 36
p = 0.289
(r = 0.25)
Opportunity cost (high respiration)
60
40
20
I
L
V
W
F
Y
H
K
M
C
T
R
N
P
D
A
S
G
Q
E
Auxotrophic
Prototrophic
AA dispensability
high respiration (3 auxotrophic AAs)
p = 0.32
Proportion of permutations (%)
2.5
2.0
1.5
1.0
0.5
0.0
10.0
29.0
29.5
57.0
Average opportunity cost (~P)

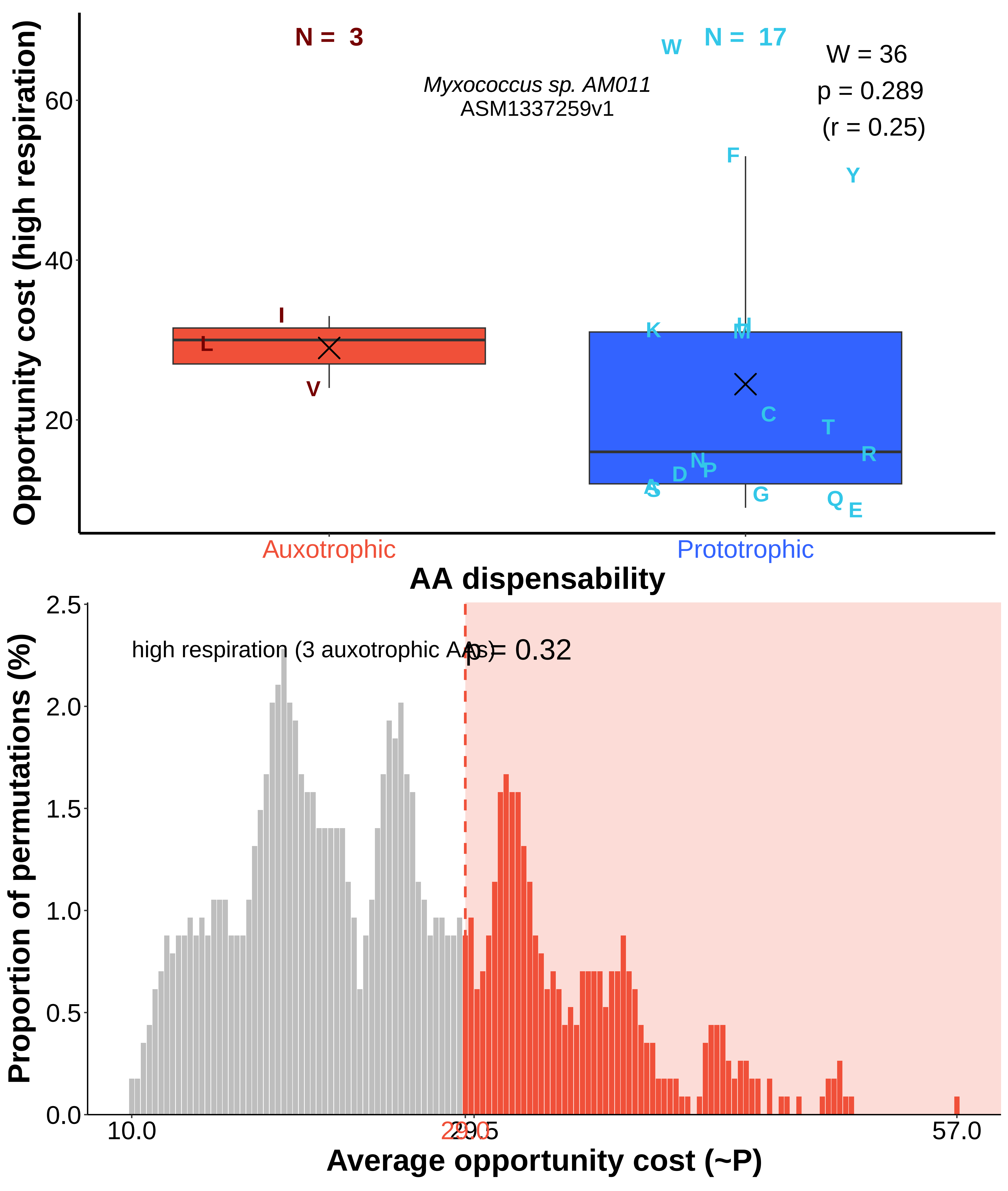
N = 3
N = 17
W
W = 36
p = 0.289
(r = 0.25)
Myxococcus sp. AM011
ASM1337259v1
Opportunity cost (high respiration)
60
40
20
F
Y
I
L
V
K
H
M
C
T
R
N
P
D
A
S
G
Q
E
Auxotrophic
Prototrophic
AA dispensability
high respiration (3 auxotrophic AAs)
p = 0.32
Proportion of permutations (%)
2.5
2.0
1.5
1.0
0.5
0.0
10.0
29.5
29.0
57.0
Average opportunity cost (~P)

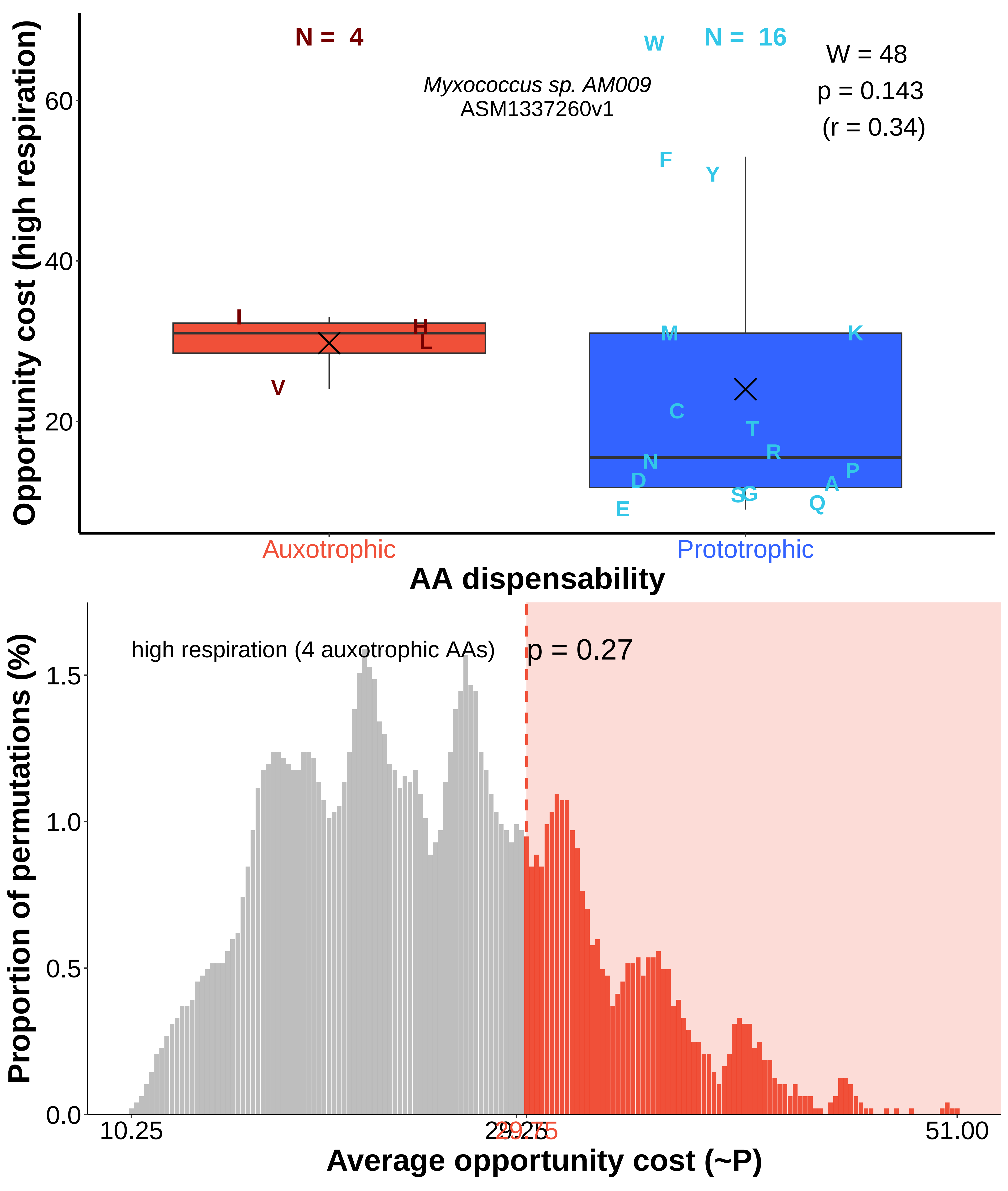
N = 4
N = 16
W
Myxococcus sp. AM009
ASM1337260v1
W = 48
p = 0.143
(r = 0.34)
Opportunity cost (high respiration)
60
40
20
I H L V
F Y M K C T R N D P A SG Q E
Auxotrophic
Prototrophic
AA dispensability
high respiration (4 auxotrophic AAs)
p = 0.27
Proportion of permutations (%)
1.5
1.0
0.5
0.0
10.25
29.75
29.25
51.00
Average opportunity cost (~P)

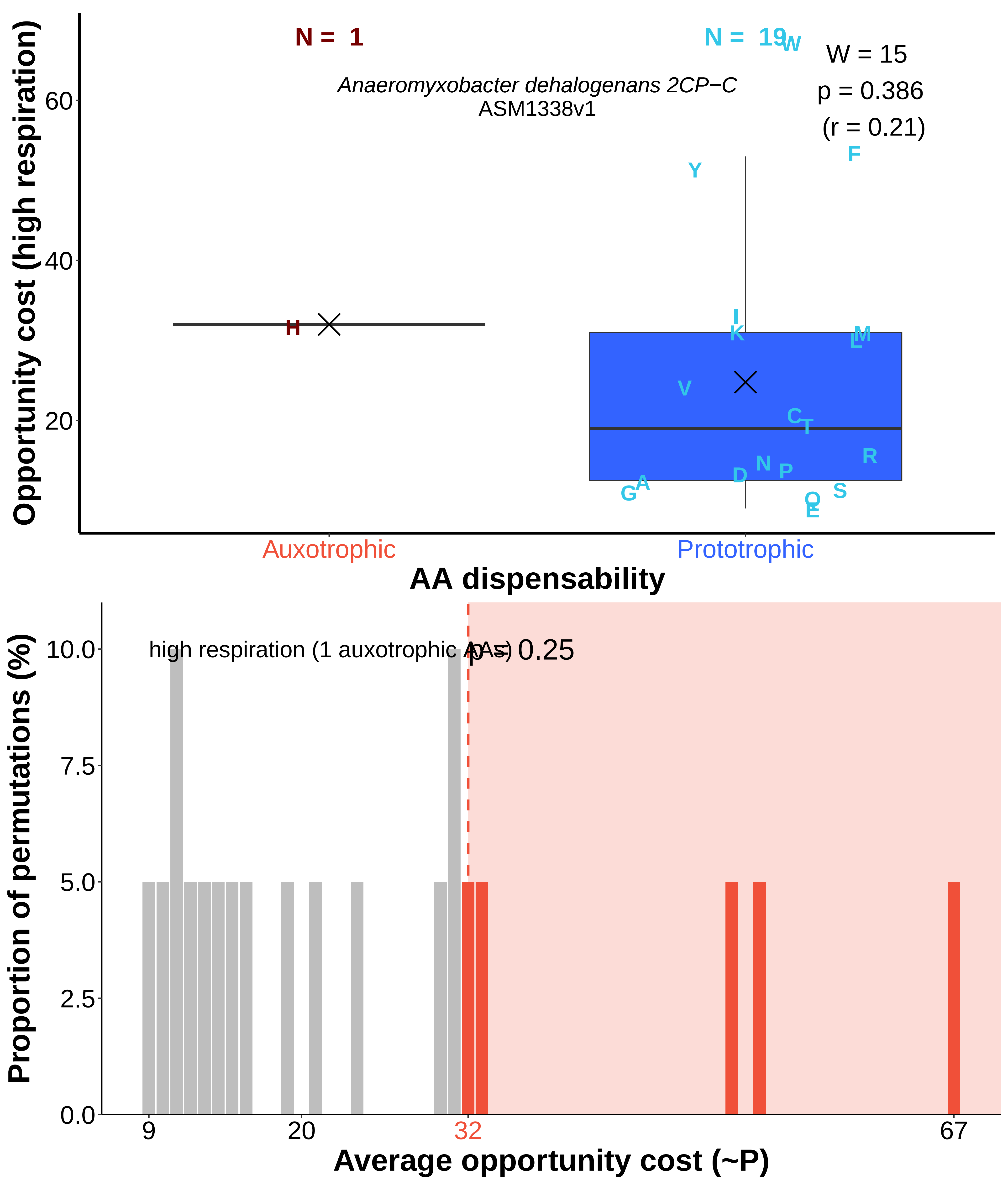

N = 1
N = 19
W = 15
p = 0.386
(r = 0.21)
Anaeromyxobacter dehalogenans 2CP−C
ASM1338v1
Opportunity cost (high respiration)
60
40
20
Auxotrophic
Prototrophic
AA dispensability
high respiration (1 auxotrophic AAs)
p = 0.25
Proportion of permutations (%)
10.0
7.5
5.0
2.5
0.0
9
20
32
67
Average opportunity cost (~P)

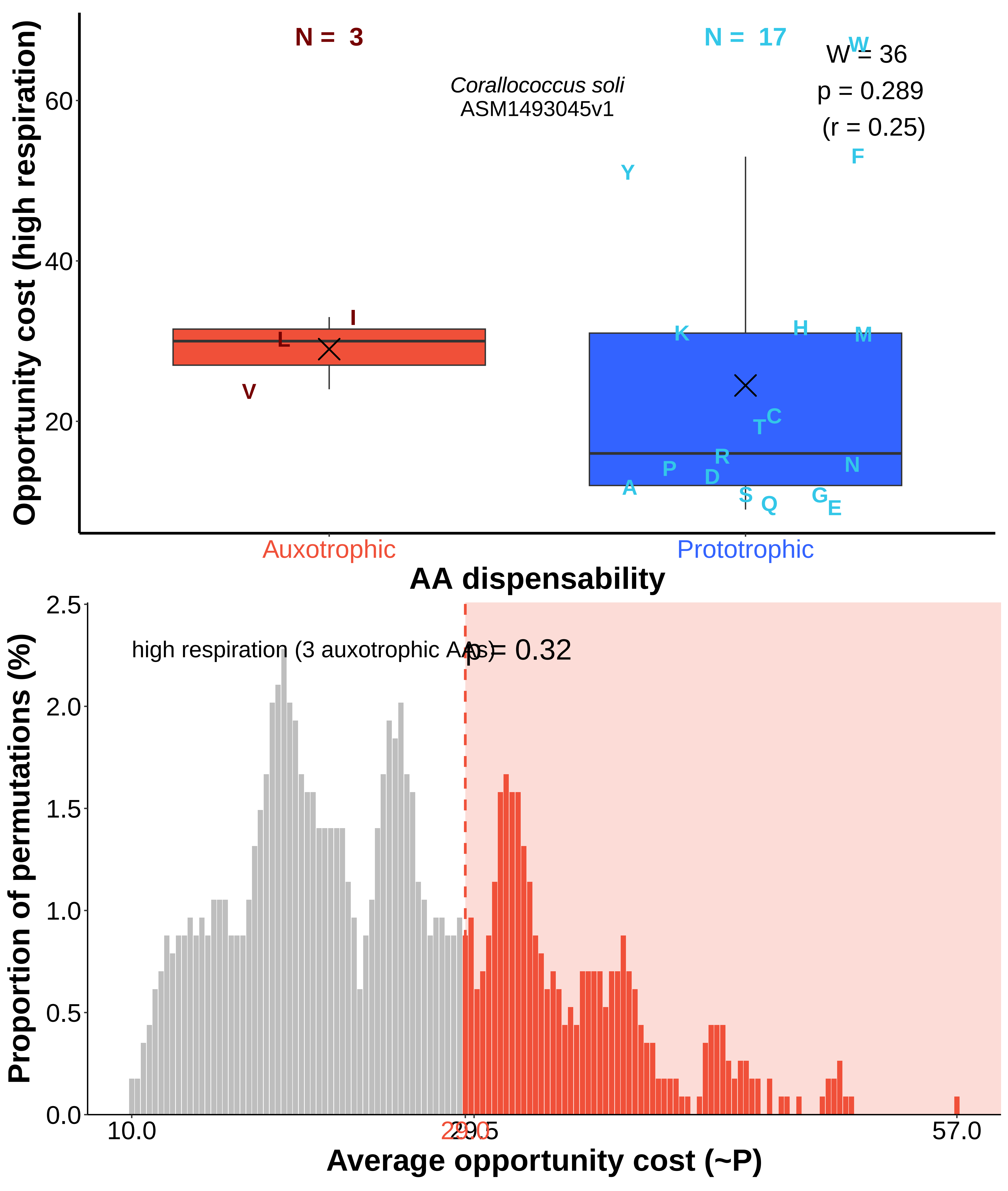

N = 3
N = 17
W = 36
p = 0.289
(r = 0.25)
Corallococcus soli
ASM1493045v1
Opportunity cost (high respiration)
60
40
20
I
L
V
Y
F
K
H
M
T
C
R
P
D
N
A
S
Q
G
E
W
Auxotrophic
Prototrophic
AA dispensability
high respiration (3 auxotrophic AAs)
p = 0.32
Proportion of permutations (%)
2.5
2.0
1.5
1.0
0.5
0.0
10.0
29.0
29.5
57.0
Average opportunity cost (~P)

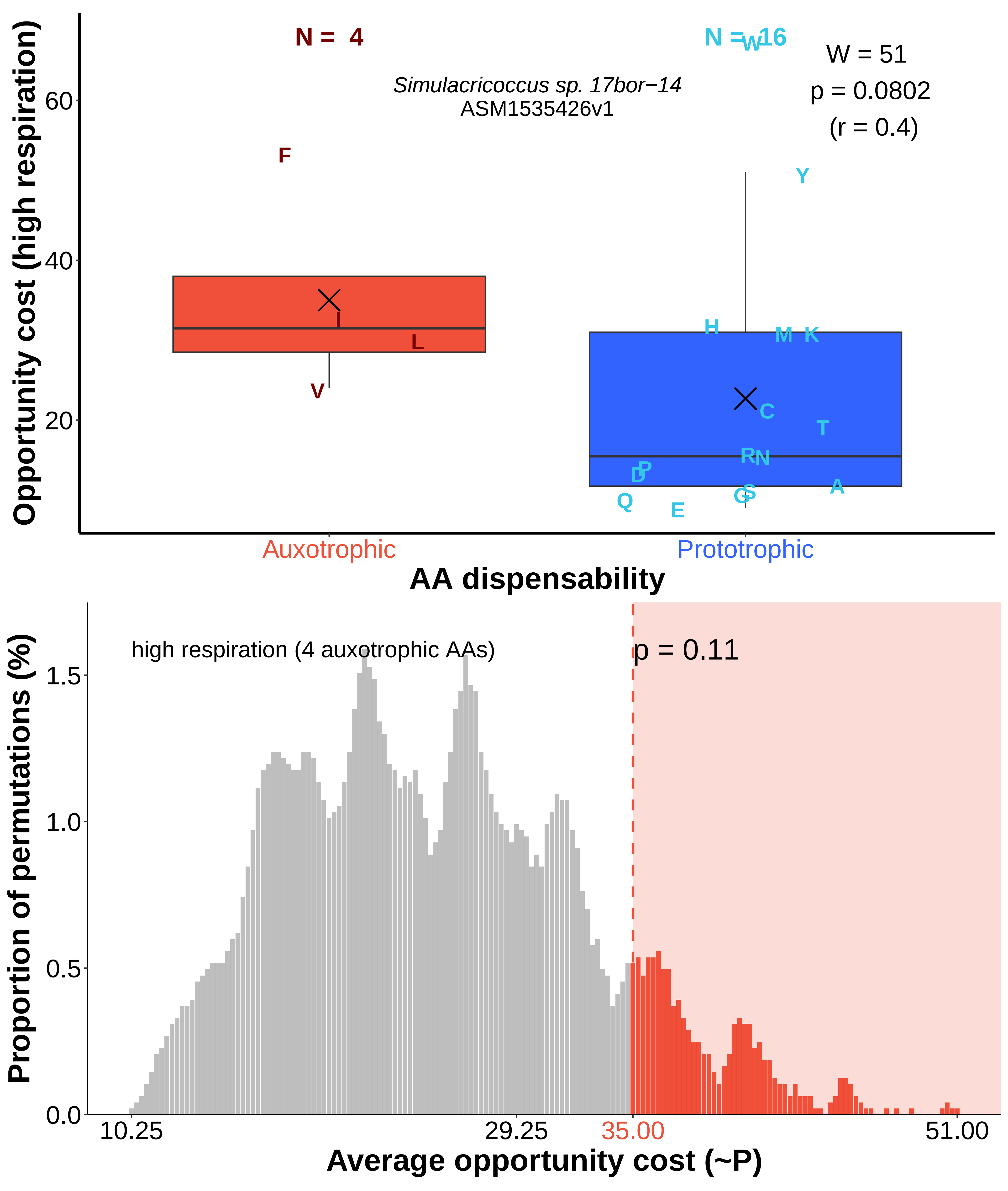
N = 4
N = 16
W
Simulacricoccus sp. 17bor−14
ASM1535426v1
W = 51
p = 0.0802
(r = 0.4)
Opportunity cost (high respiration)
60
40
20
F
I
L
V
Y
H
M
K
C
T
R
N
P
D
A
Q
G
S
E
Auxotrophic
Prototrophic
AA dispensability
high respiration (4 auxotrophic AAs)
p = 0.11
Proportion of permutations (%)
1.5
1.0
0.5
0.0
10.25
29.25
35.00
51.00
Average opportunity cost (~P)

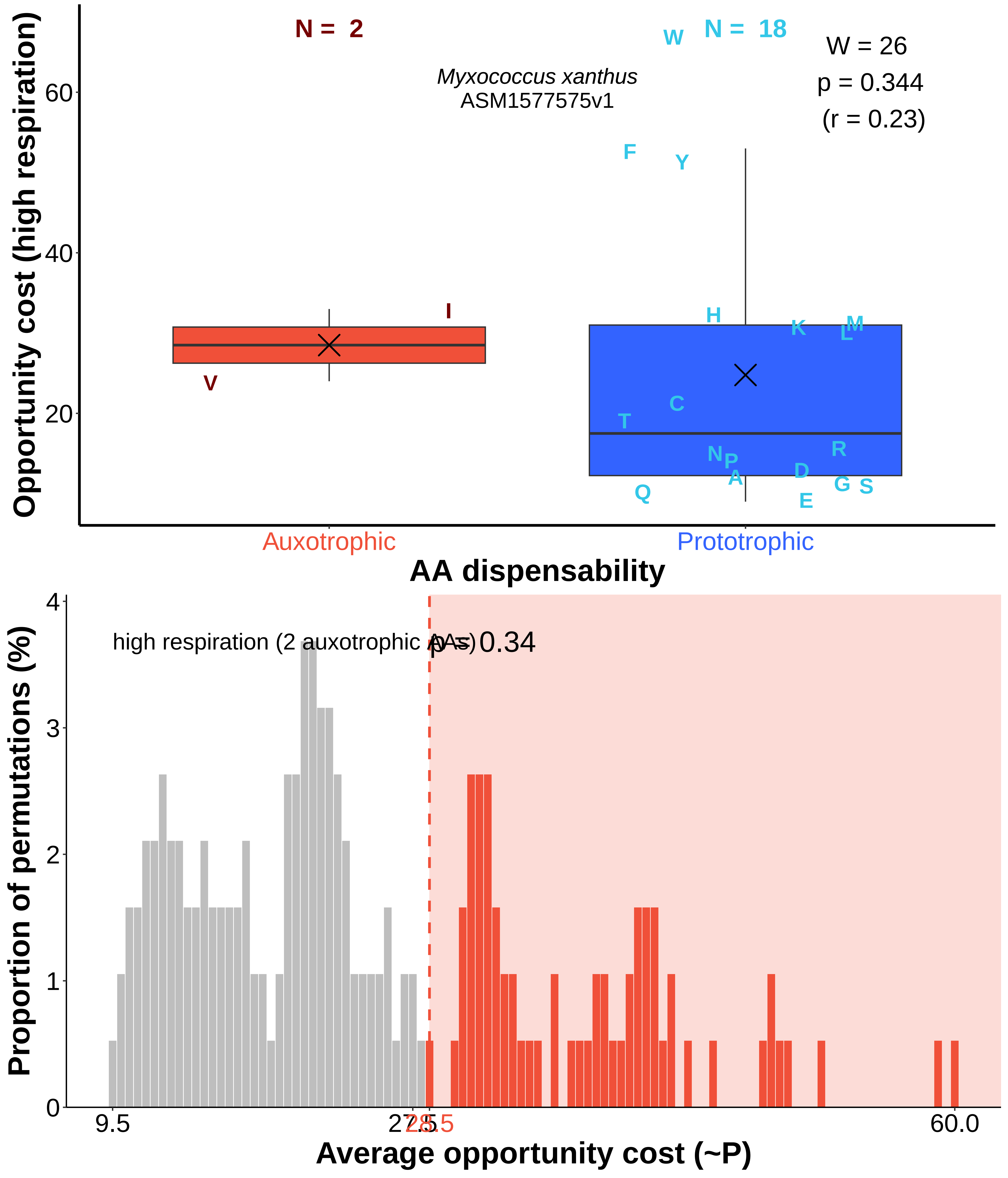
N = 2
N = 18
W = 26
p = 0.344
(r = 0.23)
Myxococcus xanthus
ASM1577575v1
Opportunity cost (high respiration)
60
40
20
W
F
Y
H
K
M
L
I
V
C
T
N
P
A
R
D
G
S
Q
E
Auxotrophic
Prototrophic
AA dispensability
Proportion of permutations (%)
4
3
2
1
0
high respiration (2 auxotrophic AAs)
p = 0.34
9.5
27.5
28.5
60.0
Average opportunity cost (~P)

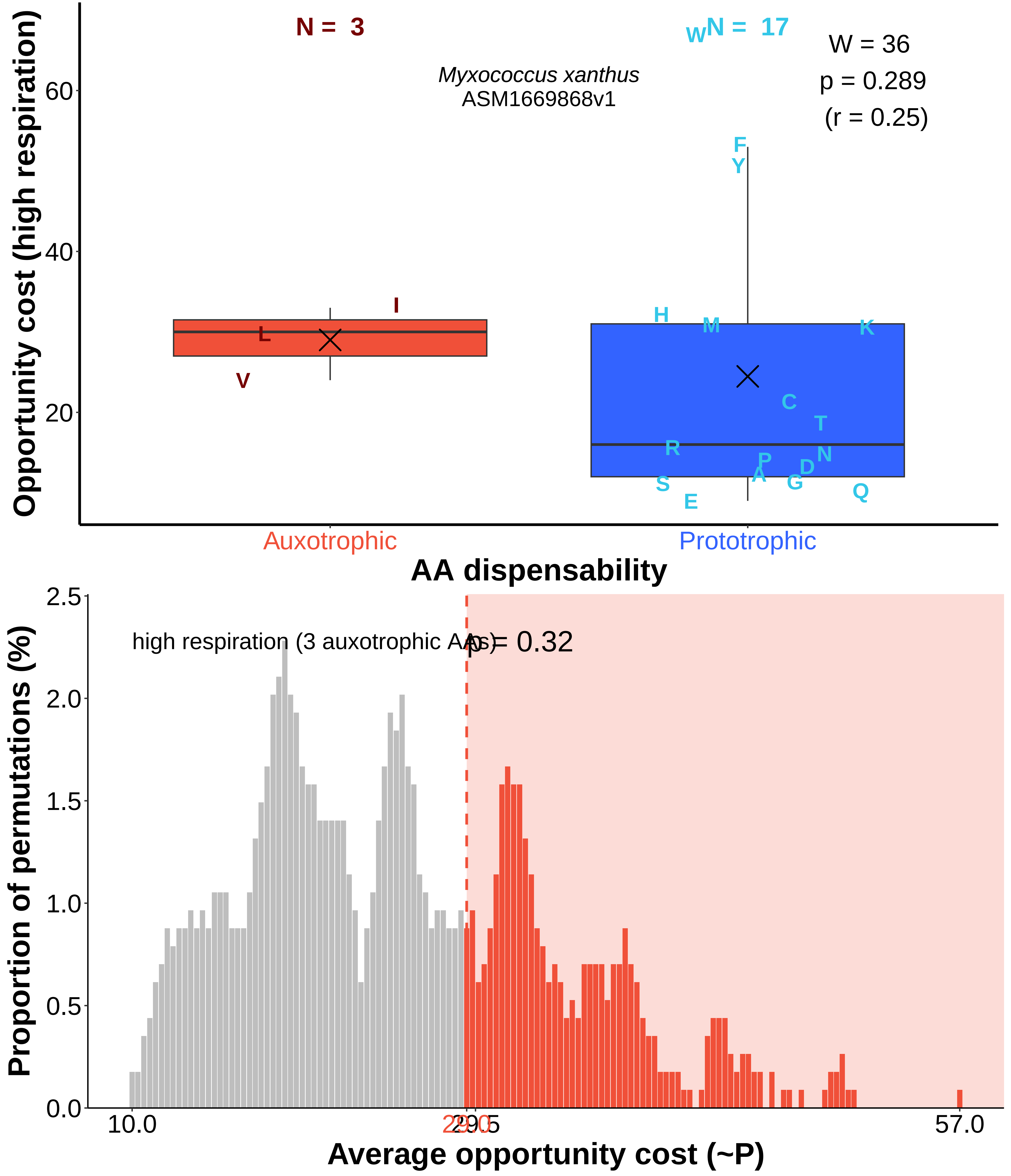
N = 3
WN = 17
W = 36
p = 0.289
(r = 0.25)
Myxococcus xanthus
ASM1669868v1
Opportunity cost (high respiration)
60
40
20
F
Y
I
H
M
K
L
V
C
T
R
N
P
D
A
G
S
Q
E
Auxotrophic
Prototrophic
AA dispensability
Proportion of permutations (%)
2.5
2.0
1.5
1.0
0.5
0.0
high respiration (3 auxotrophic AAs)
p = 0.32
10.0
29.0
29.5
57.0
Average opportunity cost (~P)

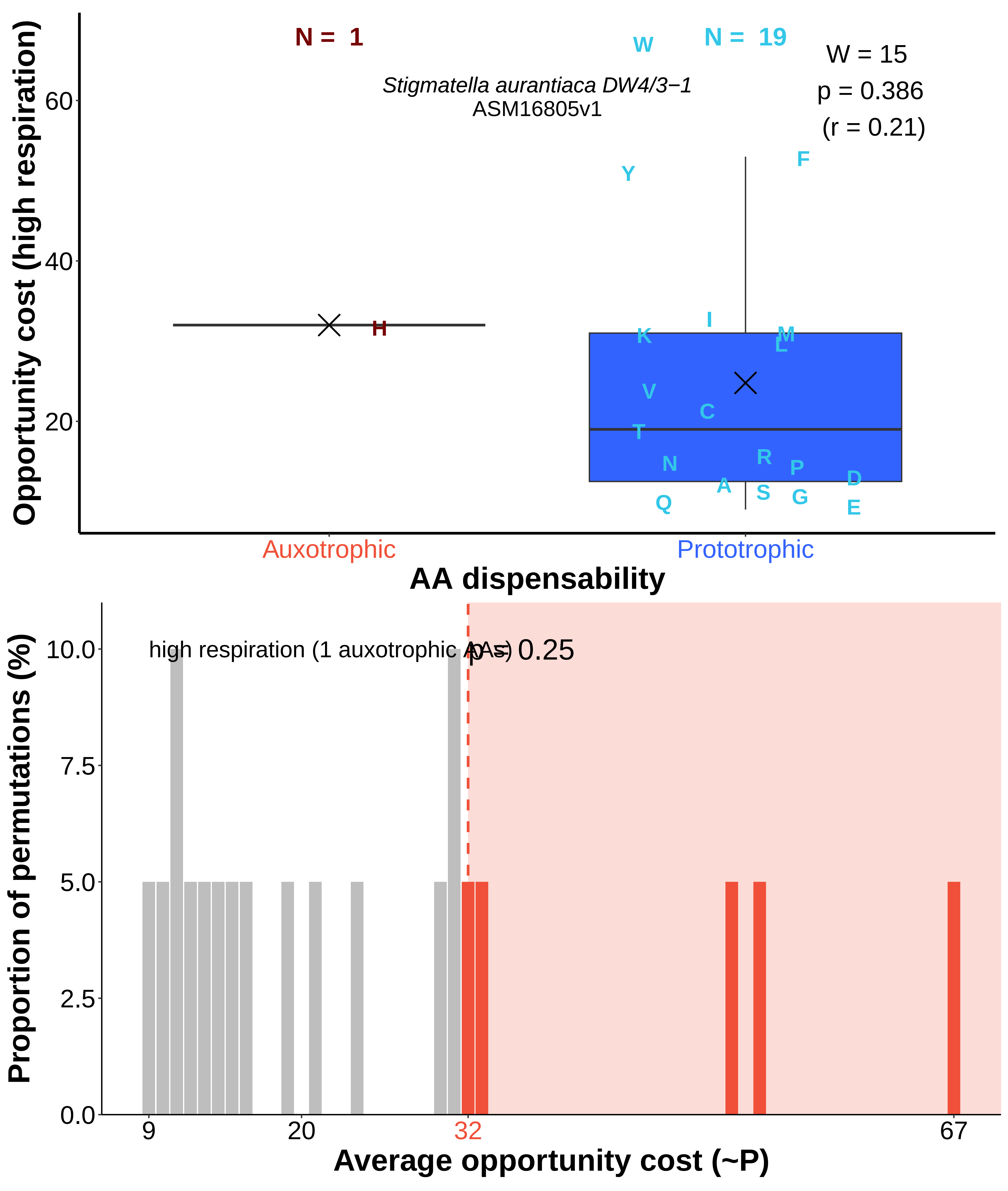
N = 1
N = 19
W = 15
p = 0.386
(r = 0.21)
Stigmatella aurantiaca DW4/3−1
ASM16805v1
Opportunity cost (high respiration)
60
40
20
W
Y
F
I
K
M
L
H
V
C
T
N
R
P
D
A
S
G
Q
E
Auxotrophic
Prototrophic
AA dispensability
high respiration (1 auxotrophic AAs)
p = 0.25
Proportion of permutations (%)
10.0
7.5
5.0
2.5
0.0
9
20
32
67
Average opportunity cost (~P)

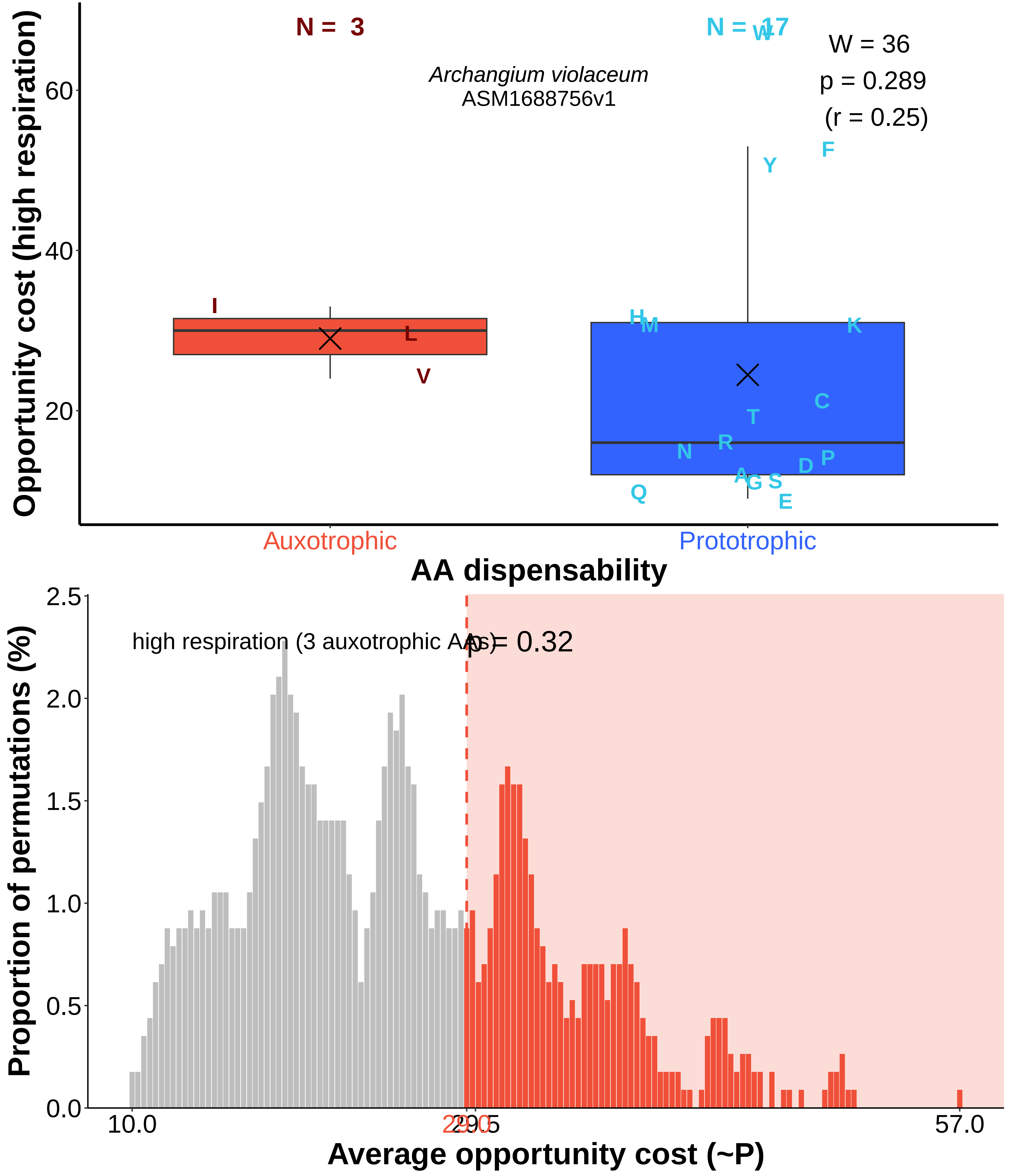

N = 3
N = 17
W
W = 36
p = 0.289
(r = 0.25)
Archangium violaceum
ASM1688756v1
Opportunity cost (high respiration)
60
40
20
Y
F
I
H
M
K
L
V
C
T
R
N
P
D
A
G
S
Q
E
Auxotrophic
Prototrophic
AA dispensability
2.5
2.0
1.5
1.0
0.5
0.0
high respiration (3 auxotrophic AAs)
p = 0.32
Proportion of permutations (%)
10.0
29.0
29.5
57.0
Average opportunity cost (~P)

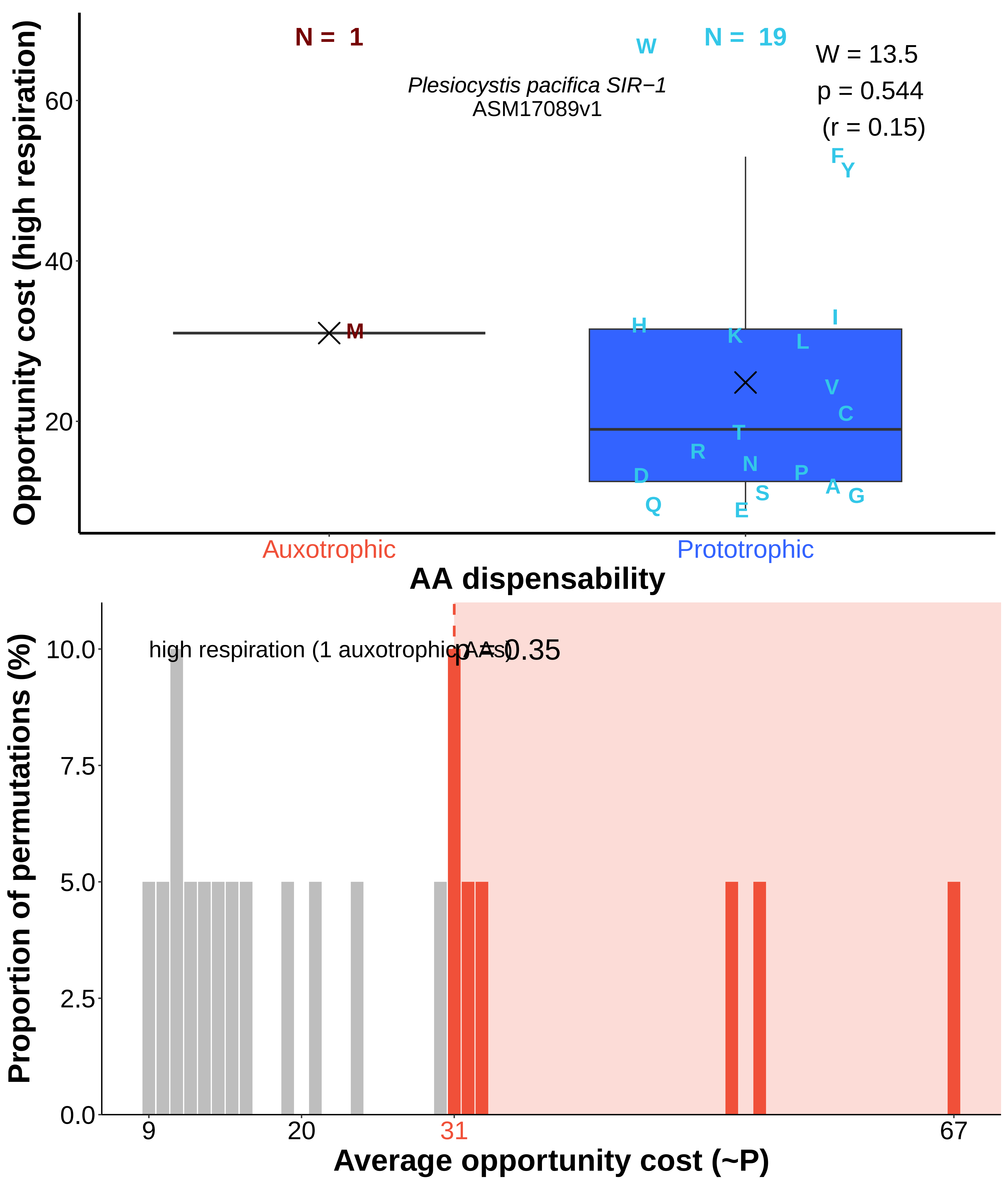

N = 1
W
N = 19
W = 13.5
p = 0.544
(r = 0.15)
Plesiocystis pacifica SIR−1
ASM17089v1
Opportunity cost (high respiration)
60
40
20
F
Y
M
H
K
I
L
V
C
T
R
N
D
P
A
S
G
Q
E
Auxotrophic
Prototrophic
AA dispensability
high respiration (1 auxotrophic AAs)
p = 0.35
Proportion of permutations (%)
10.0
7.5
5.0
2.5
0.0
9
20
31
67
Average opportunity cost (~P)

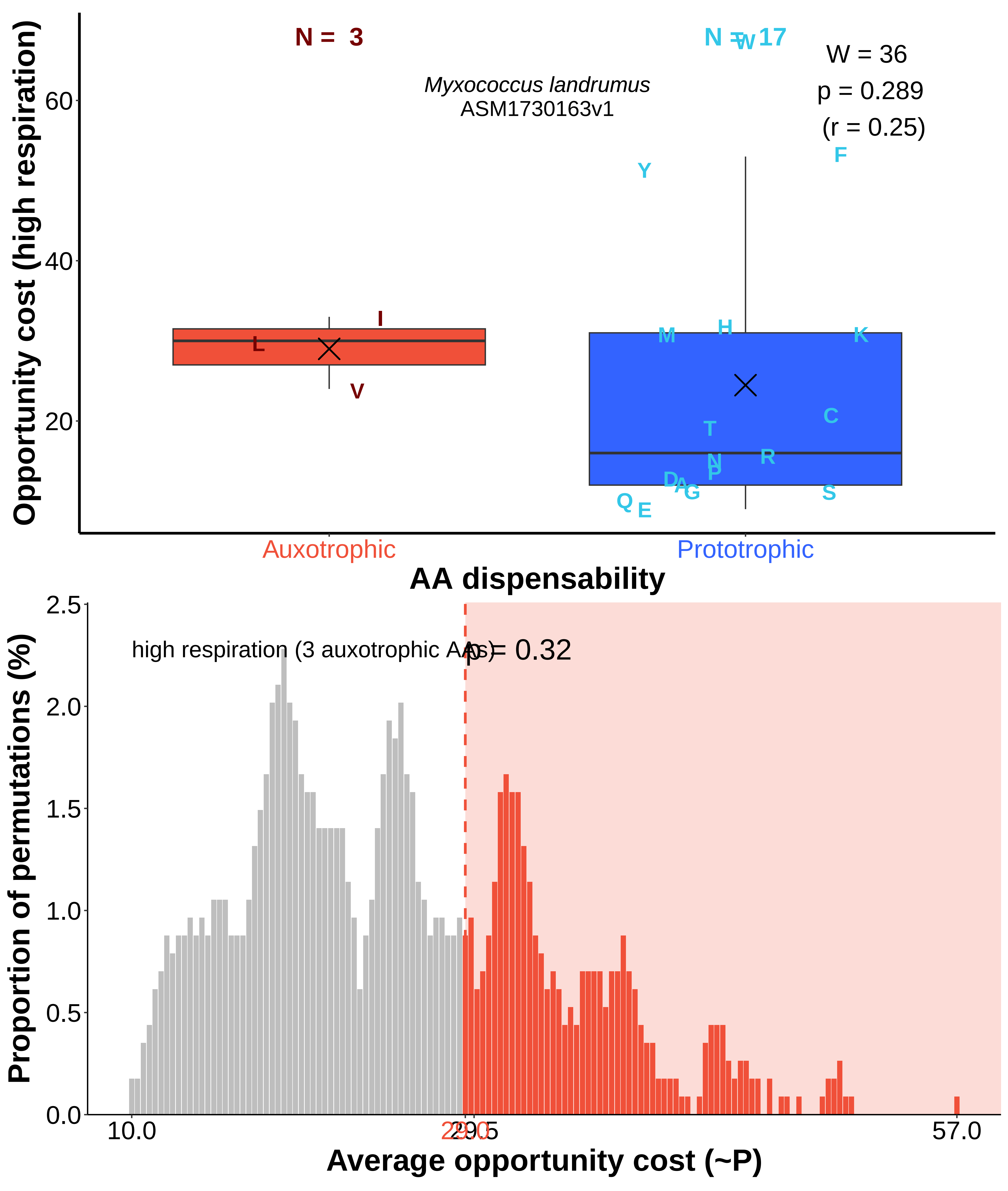

N = 3
N = 17
W
W = 36
p = 0.289
(r = 0.25)
Myxococcus landrumus
ASM1730163v1
Opportunity cost (high respiration)
60
40
20
Y
F
I
M
H
K
L
V
C
T
N
R
P
D
A
G
S
Q
E
Auxotrophic
Prototrophic
AA dispensability
Proportion of permutations (%)
2.5
2.0
1.5
1.0
0.5
0.0
high respiration (3 auxotrophic AAs)
p = 0.32
10.0
29.0
29.5
57.0
Average opportunity cost (~P)

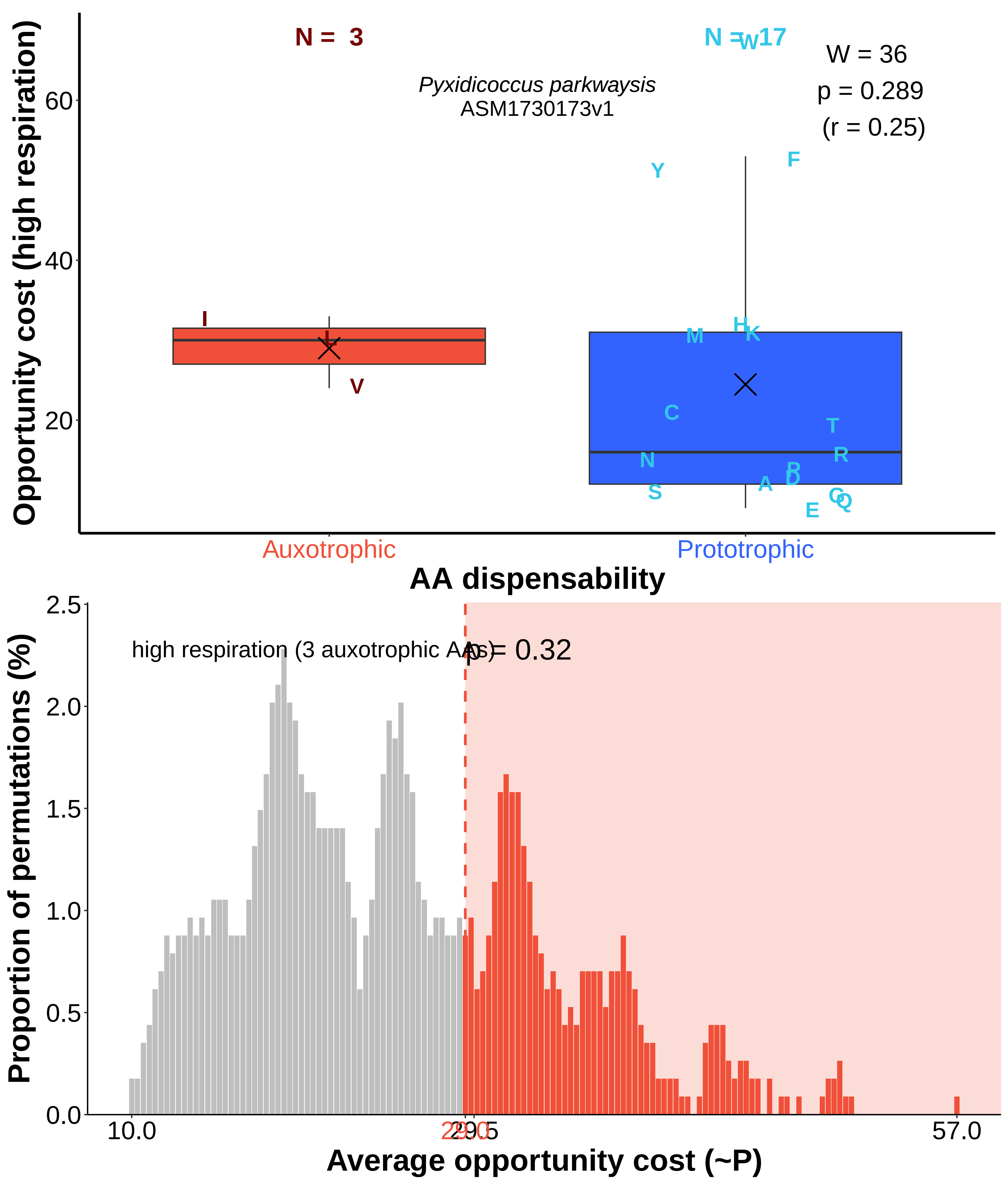

N = 3
N = 17
W
W = 36
p = 0.289
(r = 0.25)
Pyxidicoccus parkwaysis
ASM1730173v1
Opportunity cost (high respiration)
60
40
20
I
L
V
Y
F
H
K
M
C
T
R
N
P
D
A
S
G
Q
E
Auxotrophic
Prototrophic
AA dispensability
Proportion of permutations (%)
2.5
2.0
1.5
1.0
0.5
0.0
high respiration (3 auxotrophic AAs)
p = 0.32
10.0
29.0
29.5
57.0
Average opportunity cost (~P)

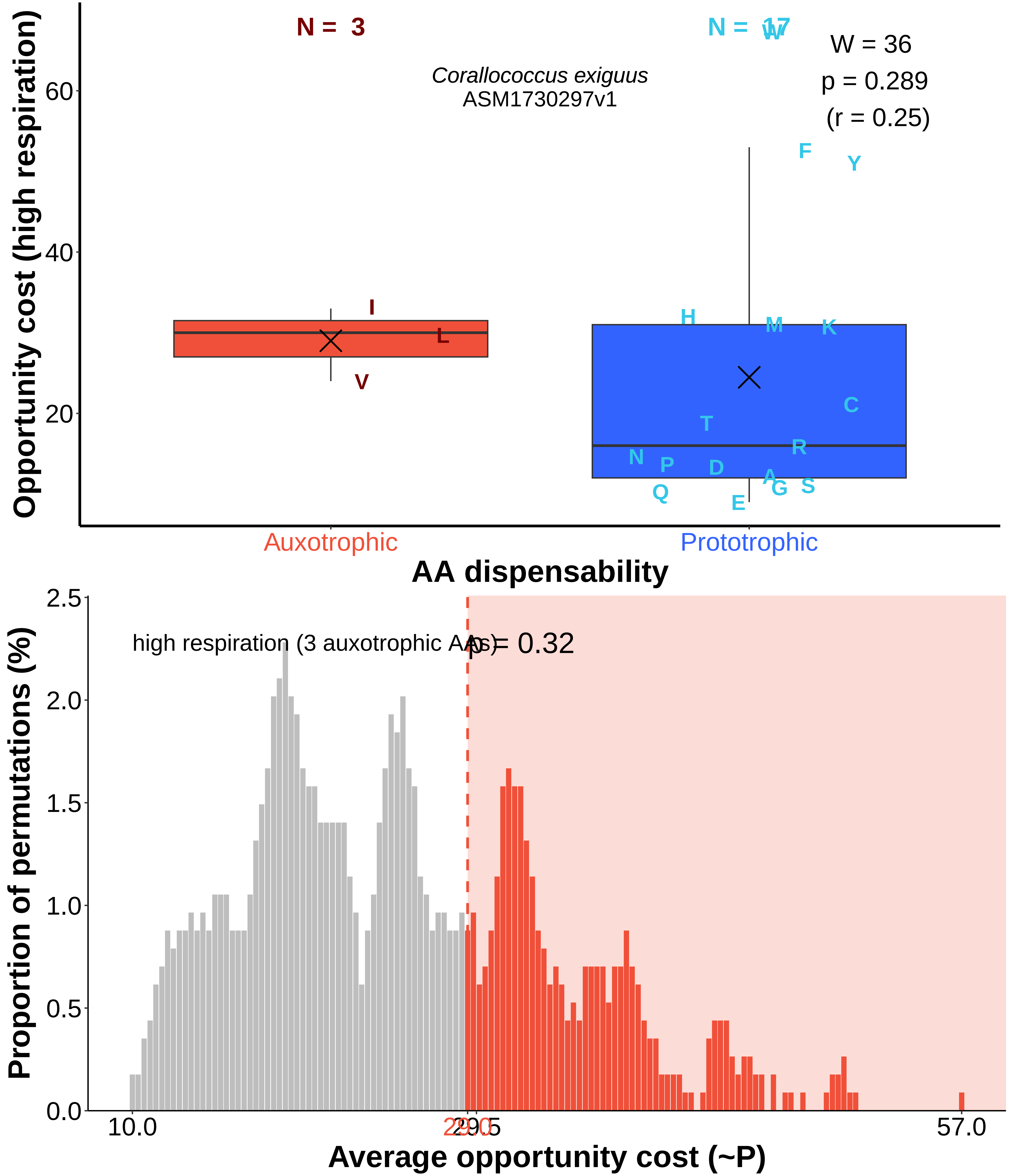

N = 3
N = 17
W
Corallococcus exiguus
ASM1730297v1
W = 36
p = 0.289
(r = 0.25)
Opportunity cost (high respiration)
60
40
20
F
Y
I
L
V
H
M
K
C
T
R
N
P
D
A
G
S
Q
E
Auxotrophic
Prototrophic
AA dispensability
Proportion of permutations (%)
2.5
2.0
1.5
1.0
0.5
0.0
high respiration (3 auxotrophic AAs)
p = 0.32
10.0
29.0
29.5
57.0
Average opportunity cost (~P)

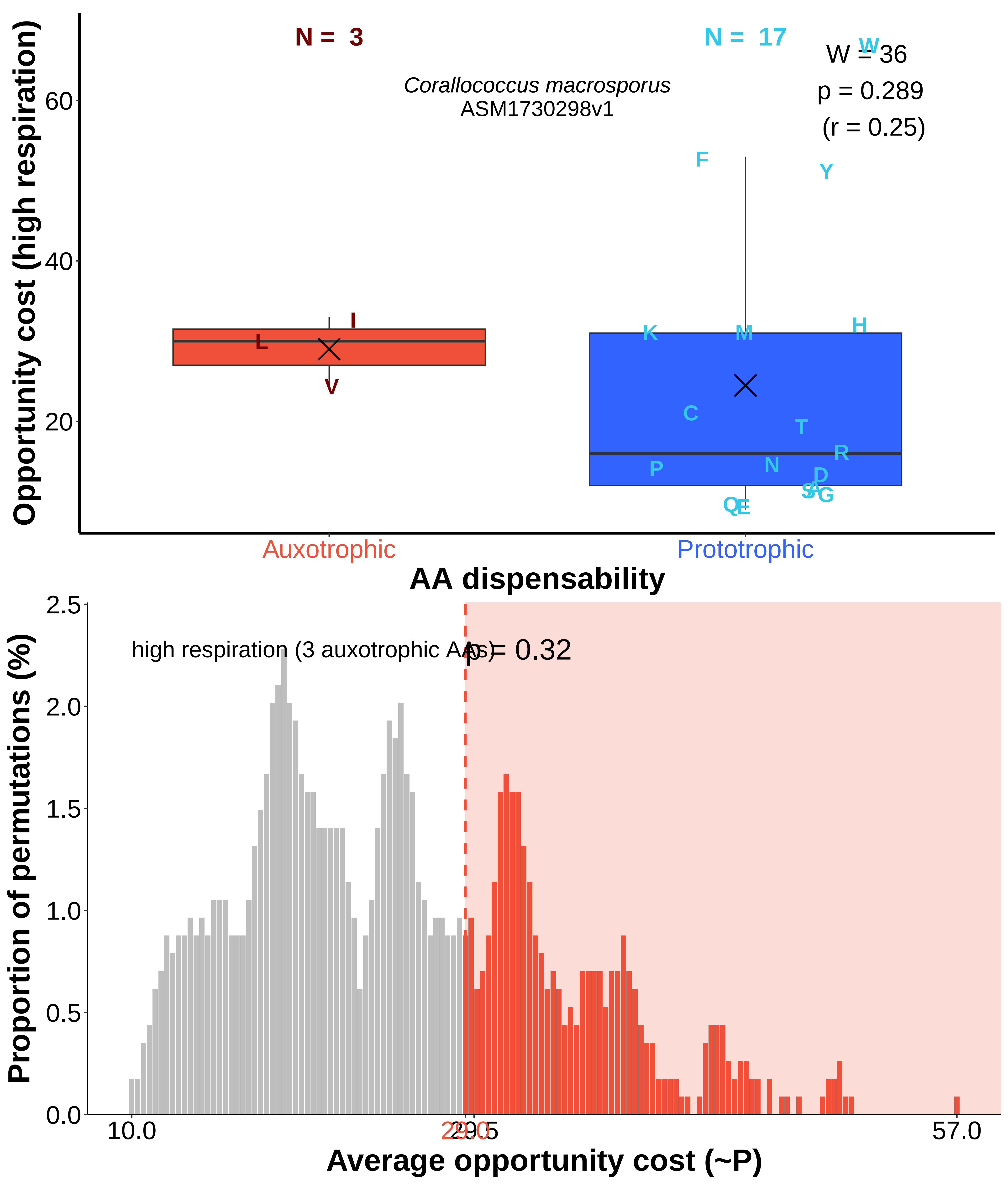
N = 3
N = 17
W = 36
p = 0.289
(r = 0.25)
Corallococcus macrosporus
ASM1730298v1
Opportunity cost (high respiration)
60
40
20
I
L
V
F
Y
K
M
H
C
T
R
P
N
D
S
A
G
Q
E
W
Auxotrophic
Prototrophic
AA dispensability
high respiration (3 auxotrophic AAs)
p = 0.32
Proportion of permutations (%)
2.5
2.0
1.5
1.0
0.5
0.0
10.0
29.0
29.5
57.0
Average opportunity cost (~P)

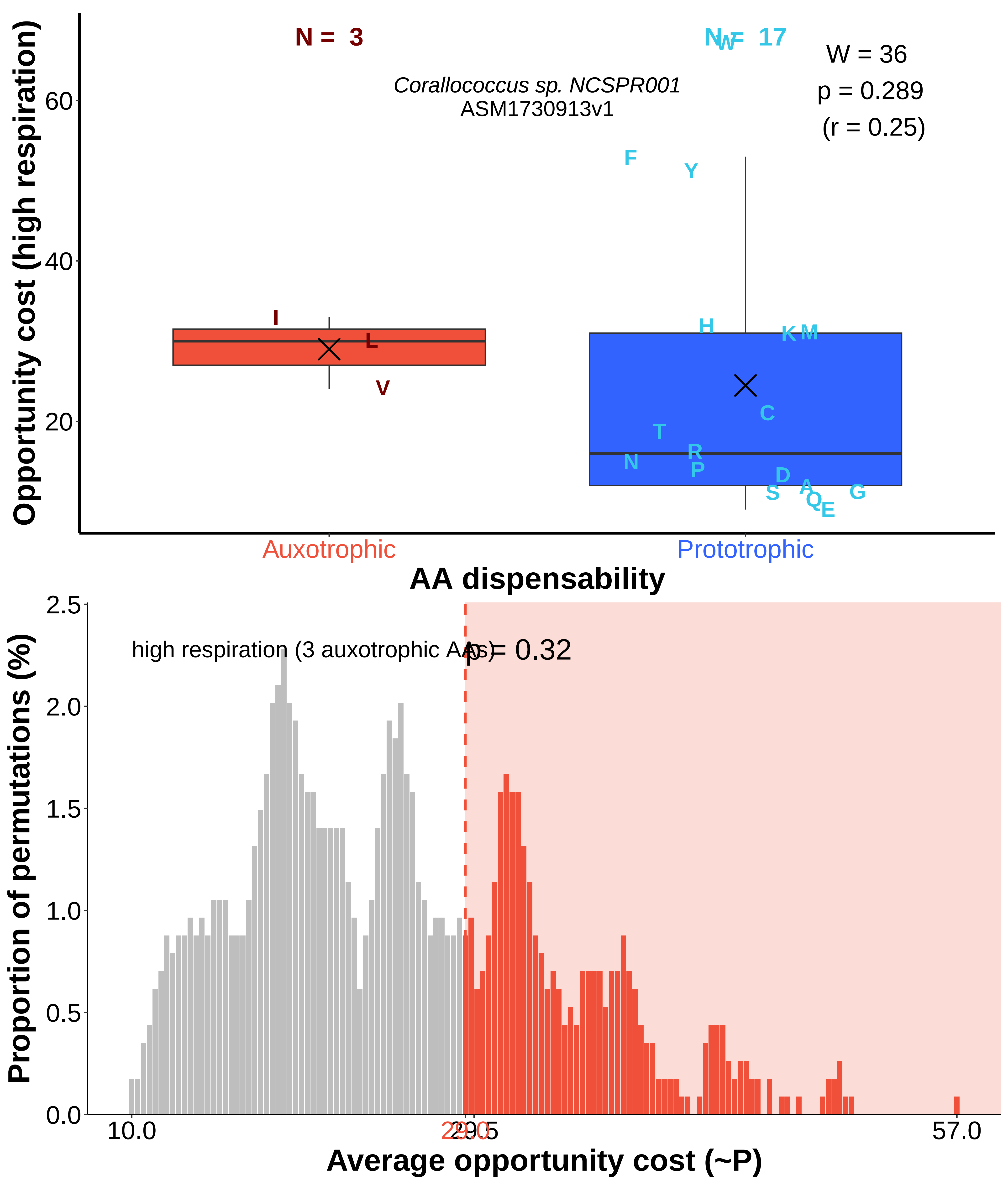

N = 3
N = 17
W = 36
p = 0.289
(r = 0.25)
Corallococcus sp. NCSPR001
ASM1730913v1
Opportunity cost (high respiration)
Auxotrophic
Prototrophic
AA dispensability
high respiration (3 auxotrophic AAs)
p = 0.32
Proportion of permutations (%)
Average opportunity cost (~P)
10.0
29.0
57.0

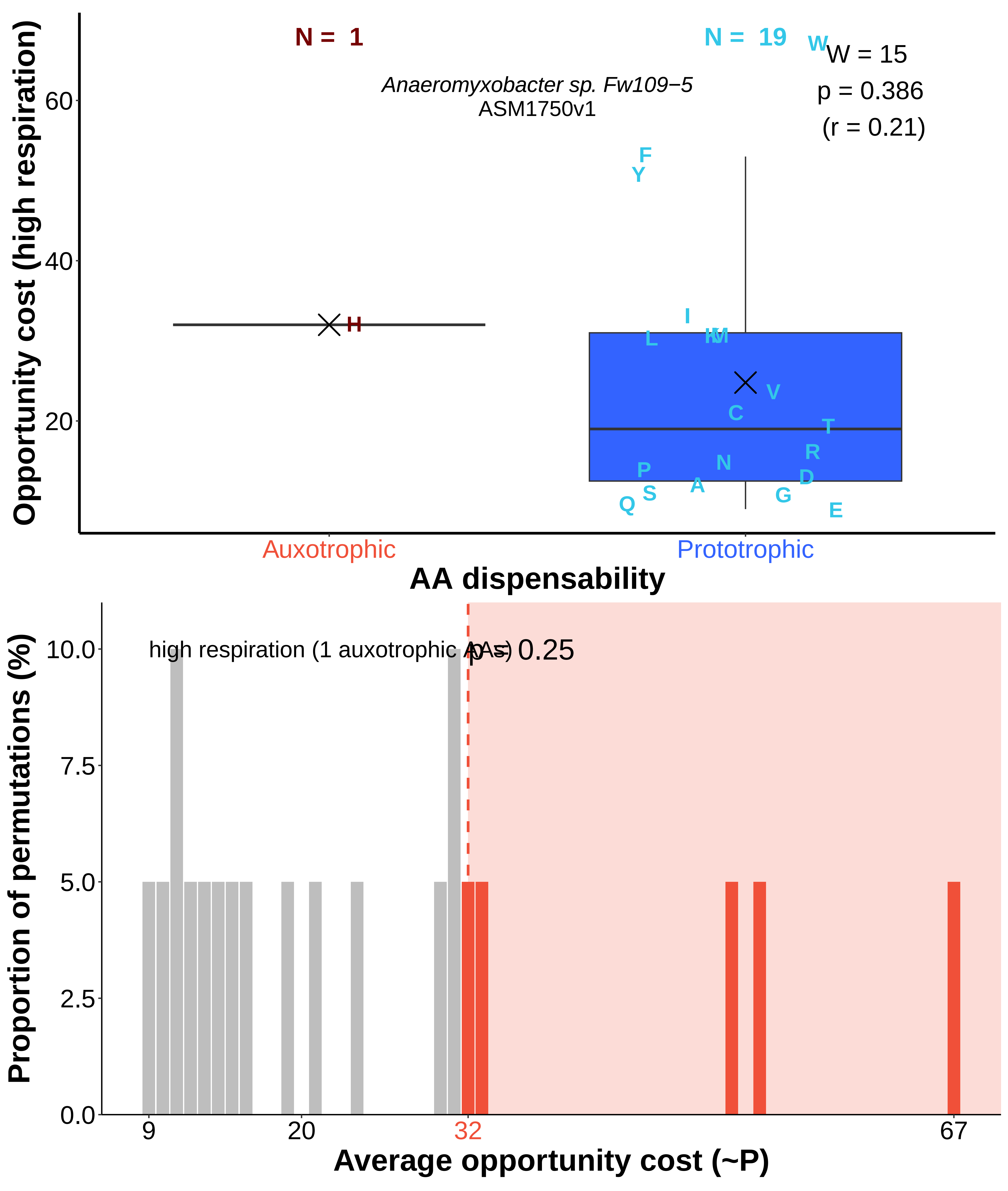

N = 1
N = 19
Anaeromyxobacter sp. Fw109−5
ASM1750v1
W = 15
p = 0.386
(r = 0.21)
Opportunity cost (high respiration)
Auxotrophic
Prototrophic
AA dispensability
high respiration (1 auxotrophic AAs)
p = 0.25
Proportion of permutations (%)
Average opportunity cost (~P)

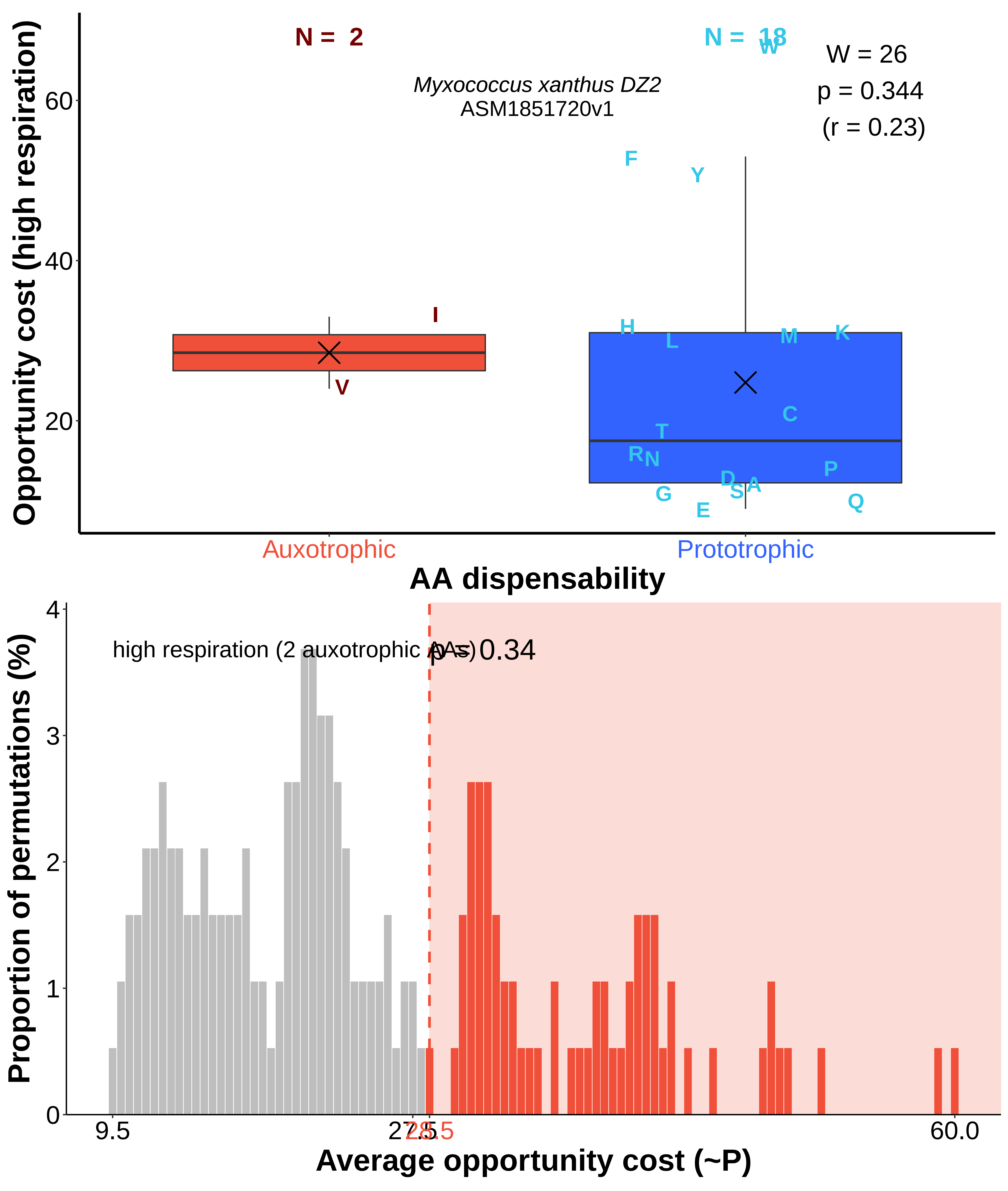

N = 2
N = 18
Myxococcus xanthus DZ2
ASM1851720v1
W = 26
p = 0.344
(r = 0.23)
Opportunity cost (high respiration)
60
40
20
Auxotrophic
Prototrophic
AA dispensability
high respiration (2 auxotrophic AAs)
p = 0.34
Proportion of permutations (%)
4
3
2
1
0
9.5
27.5
28.5
60.0
Average opportunity cost (~P)

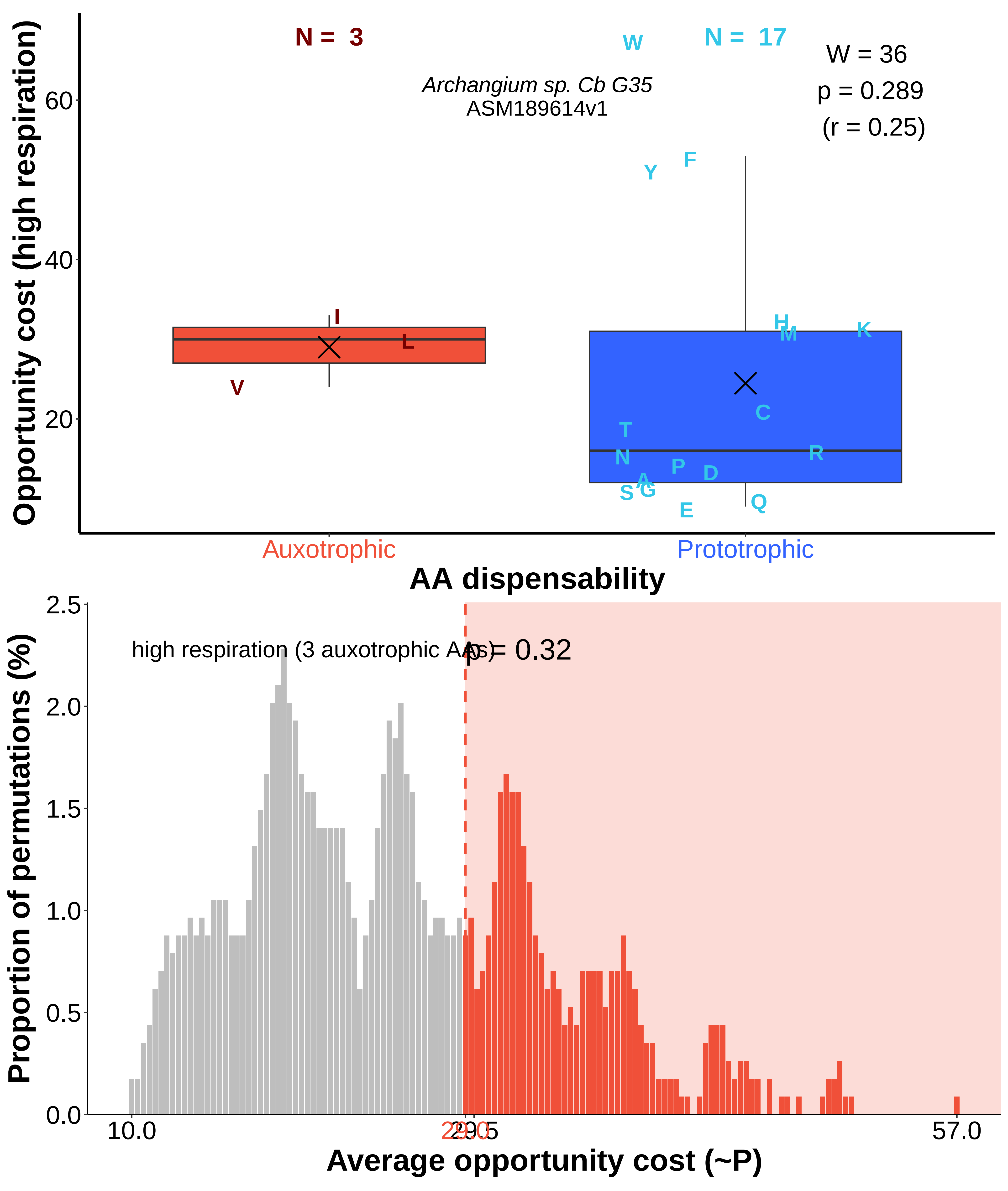

N = 3
N = 17
W
W = 36
p = 0.289
(r = 0.25)
Archangium sp. Cb G35
ASM189614v1
Opportunity cost (high respiration)
60
40
20
I
L
V
Y
F
H
M
K
C
T
N
R
P
D
A
S
G
E
Q
Auxotrophic
Prototrophic
AA dispensability
2.5
2.0
1.5
1.0
0.5
0.0
high respiration (3 auxotrophic AAs)
p = 0.32
Proportion of permutations (%)
10.0
29.0
29.5
57.0
Average opportunity cost (~P)

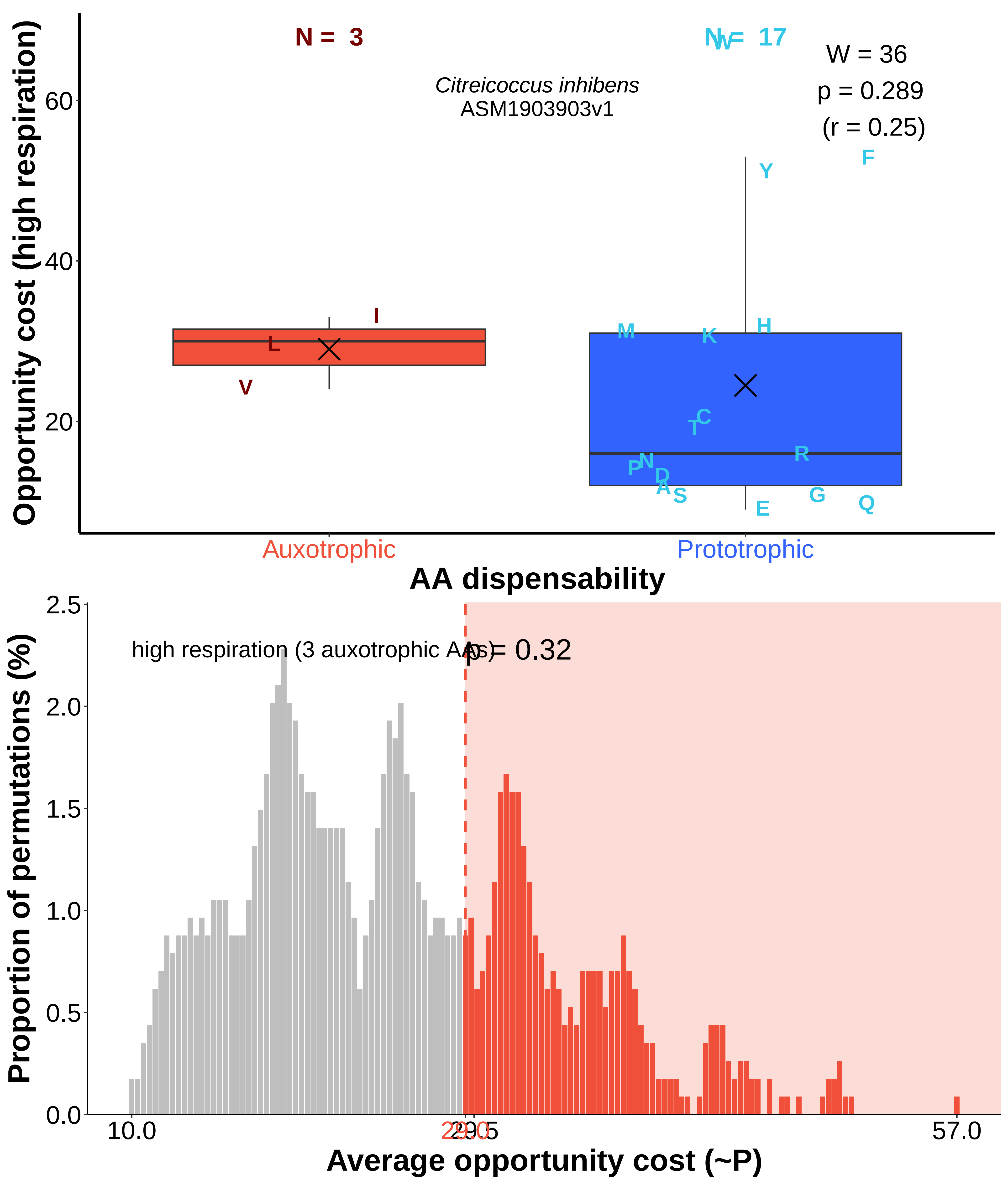

N = 3
N = 17
W
W = 36
p = 0.289
(r = 0.25)
Citreicoccus inhibens
ASM1903903v1
Opportunity cost (high respiration)
60
40
20
Y
F
I
L
V
M
K
H
C
T
R
P
N
D
A
S
E
G
Q
Auxotrophic
Prototrophic
AA dispensability
Proportion of permutations (%)
2.5
2.0
1.5
1.0
0.5
0.0
high respiration (3 auxotrophic AAs)
p = 0.32
10.0
29.0
29.5
57.0
Average opportunity cost (~P)

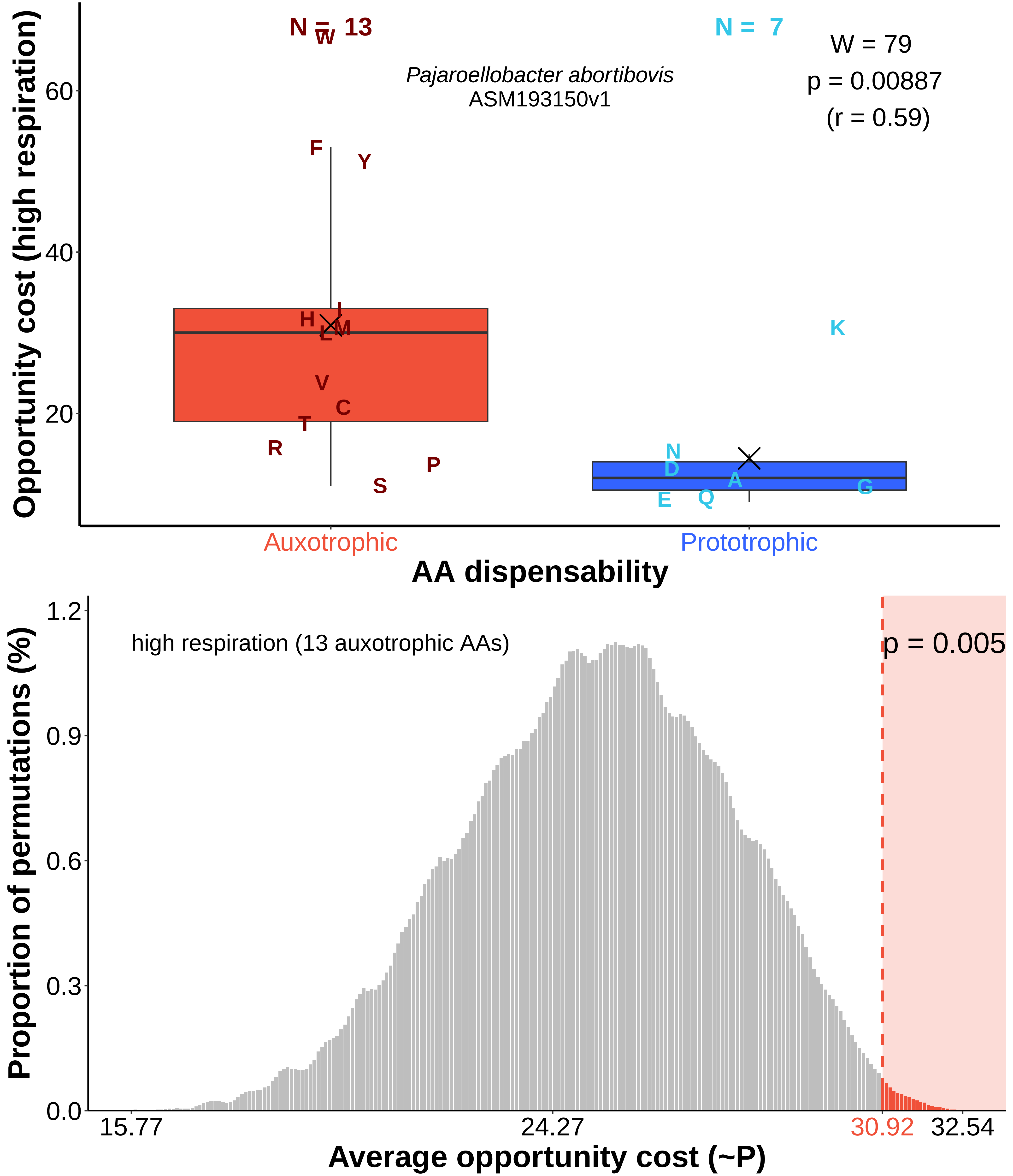

N = 13
N = 7
W = 79
p = 0.00887
(r = 0.59)
Pajaroellobacter abortibovis
ASM193150v1
Opportunity cost (high respiration)
60
40
20
W
F
Y
H
I
L
M
V
C
T
R
P
S
K
N
D
A
G
E
Q
Auxotrophic
Prototrophic
AA dispensability
Proportion of permutations (%)
1.2
0.9
0.6
0.3
0.0
high respiration (13 auxotrophic AAs)
p = 0.005
15.77
24.27
30.92
32.54
Average opportunity cost (~P)

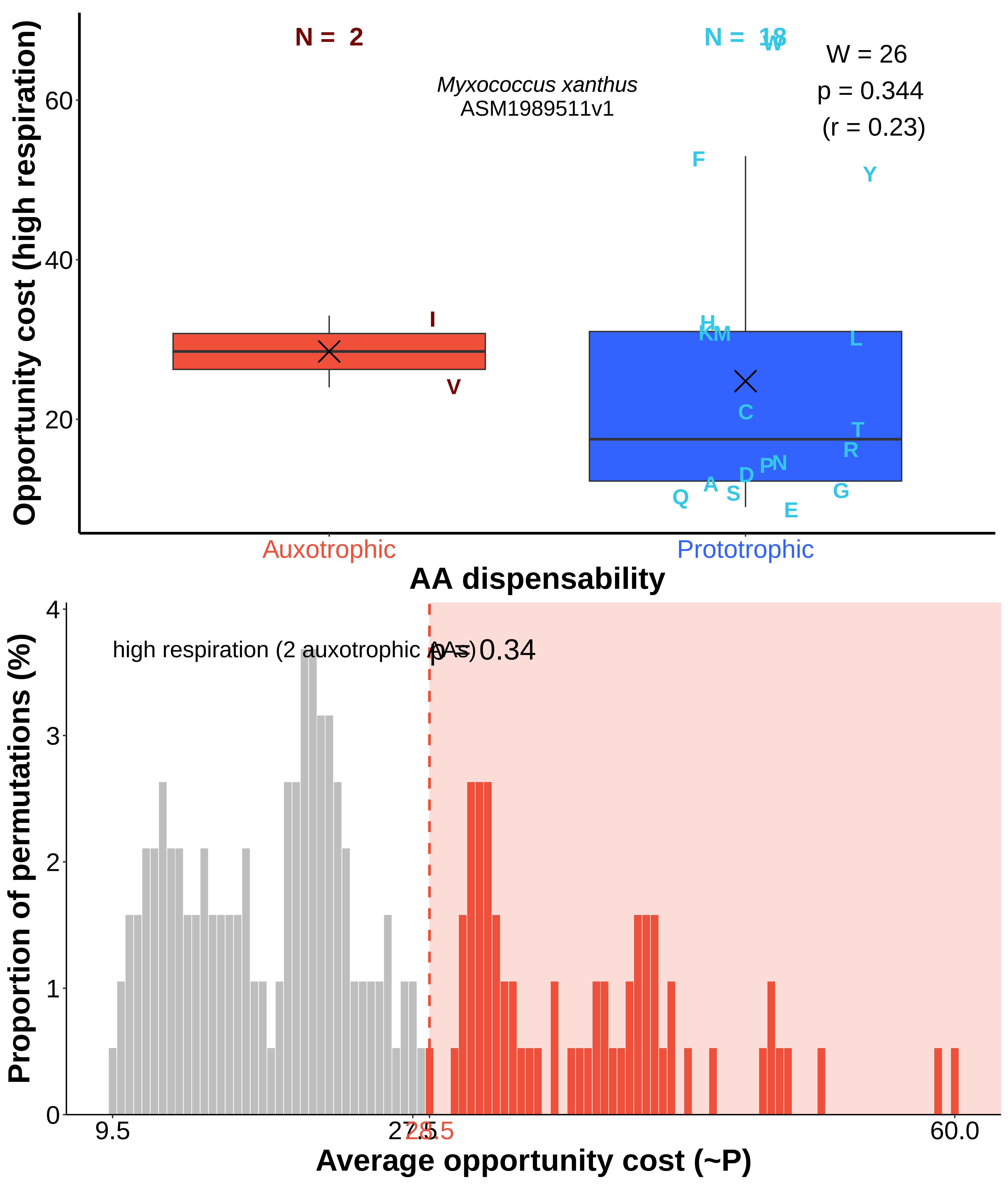
N = 2
N = 18
W
Myxococcus xanthus
ASM1989511v1
W = 26
p = 0.344
(r = 0.23)
Opportunity cost (high respiration)
60
40
20
F
Y
I
H
KM
L
V
C
T
R
PN
D
A
S
Q
G
E
Auxotrophic
Prototrophic
AA dispensability
high respiration (2 auxotrophic AAs)
p = 0.34
Proportion of permutations (%)
4
3
2
1
0
9.5
27.5
28.5
60.0
Average opportunity cost (~P)

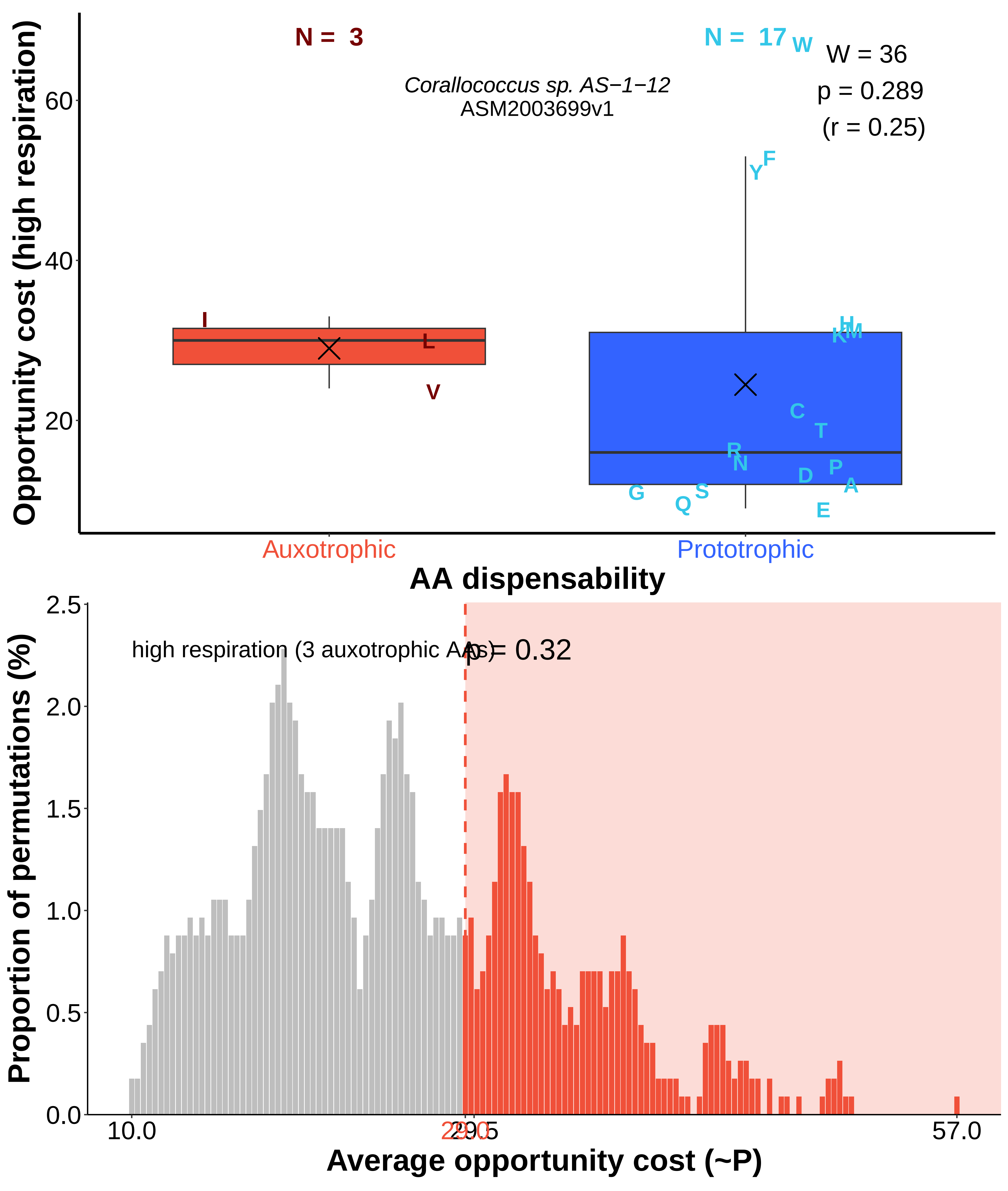

N = 3
N = 17
W
W = 36
p = 0.289
(r = 0.25)
Corallococcus sp. AS−1−12
ASM2003699v1
Opportunity cost (high respiration)
60
40
20
I
L
V
Y
F
H
K
M
C
T
R
N
P
D
A
G
S
Q
E
Auxotrophic
Prototrophic
AA dispensability
high respiration (3 auxotrophic AAs)
p = 0.32
Proportion of permutations (%)
2.5
2.0
1.5
1.0
0.5
0.0
10.0
29.0
29.5
57.0
Average opportunity cost (~P)

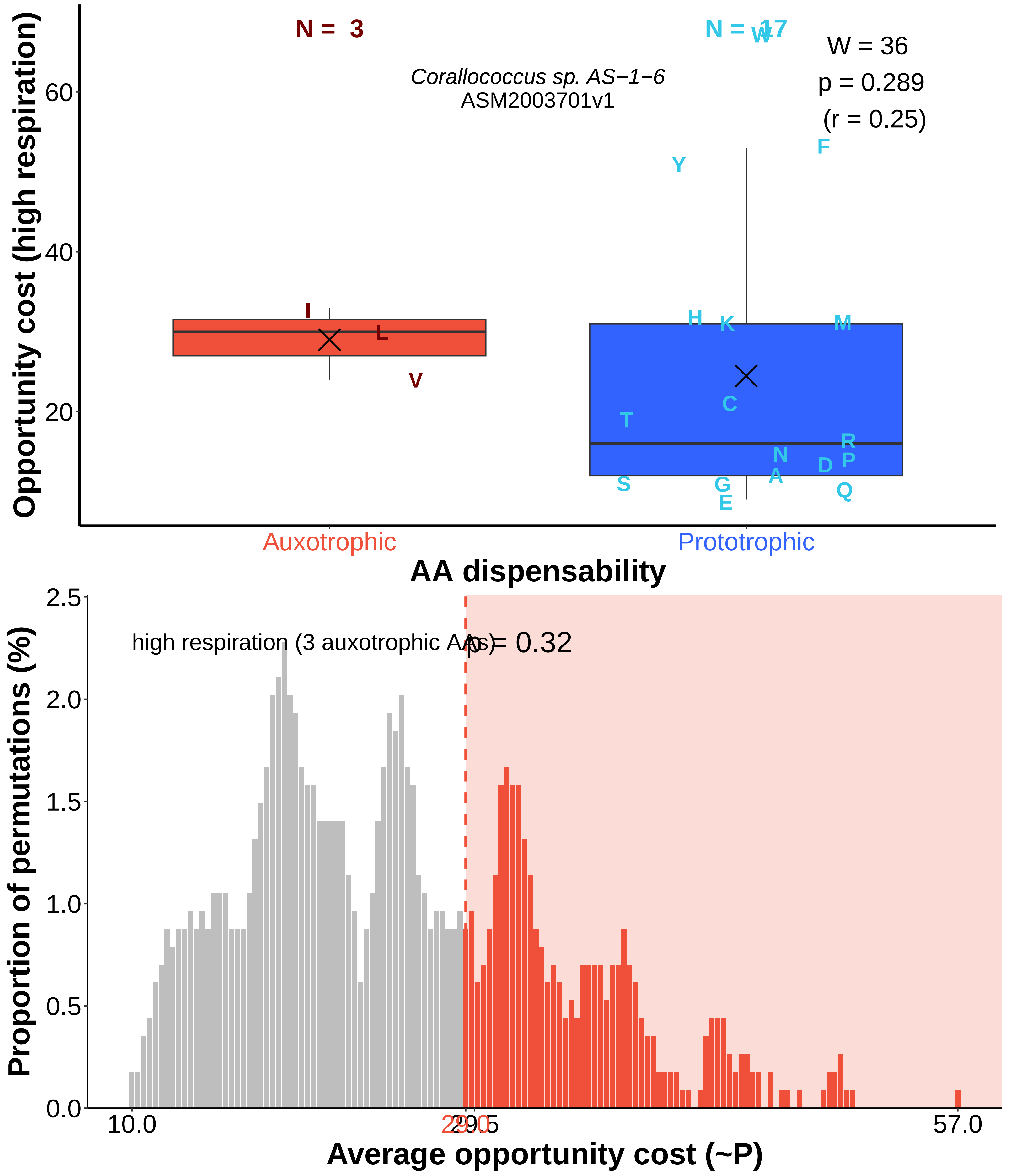

N = 3
N = 17
W
W = 36
p = 0.289
(r = 0.25)
Corallococcus sp. AS−1−6
ASM2003701v1
Opportunity cost (high respiration)
60
40
20
I
L
V
Y
F
H
K
M
C
T
R
N
D
P
A
S
G
E
Q
Auxotrophic
Prototrophic
AA dispensability
high respiration (3 auxotrophic AAs)
p = 0.32
Proportion of permutations (%)
2.5
2.0
1.5
1.0
0.5
0.0
10.0
29.0
29.5
57.0
Average opportunity cost (~P)

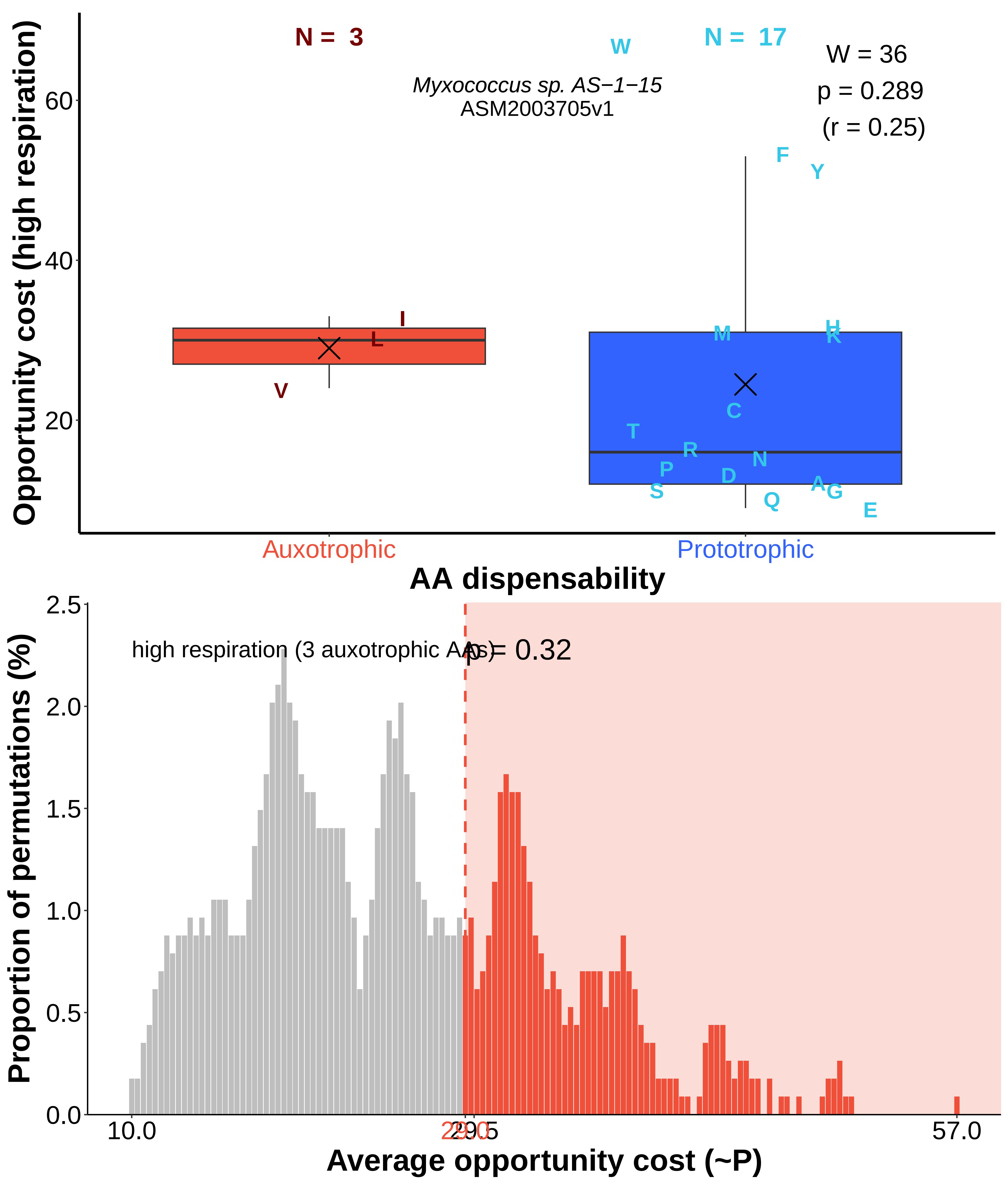

N = 3
N = 17
W = 36
p = 0.289
(r = 0.25)
Myxococcus sp. AS−1−15
ASM2003705v1
Opportunity cost (high respiration)
60
40
20
Auxotrophic
Prototrophic
AA dispensability
high respiration (3 auxotrophic AAs)
p = 0.32
Proportion of permutations (%)
2.5
2.0
1.5
1.0
0.5
0.0
10.0
29.0
29.5
57.0
Average opportunity cost (~P)

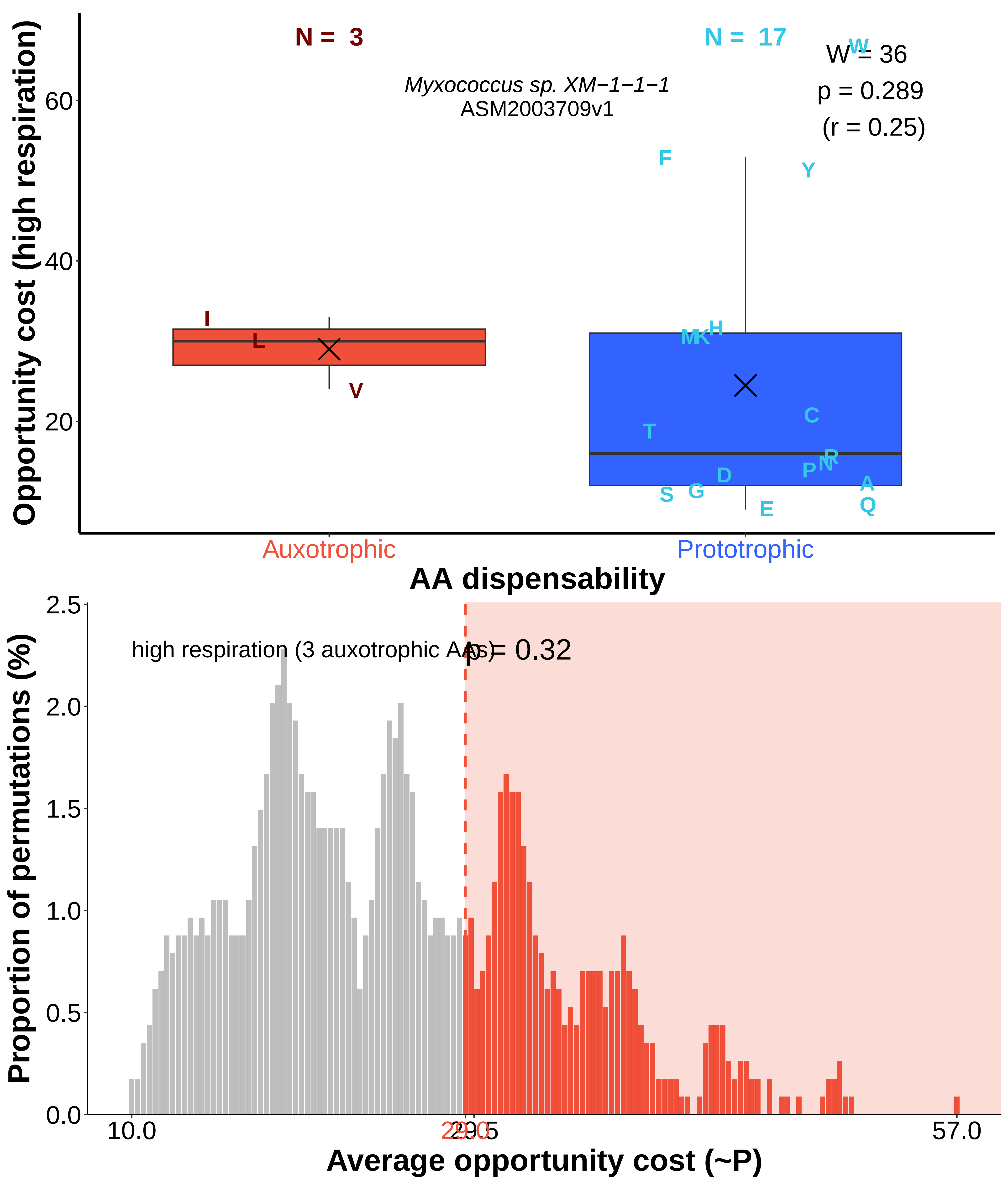

N = 3
N = 17
Myxococcus sp. XM−1−1−1
ASM2003709v1
W = 36
p = 0.289
(r = 0.25)
Opportunity cost (high respiration)
Auxotrophic
Prototrophic
AA dispensability
high respiration (3 auxotrophic AAs)
p = 0.32
Proportion of permutations (%)
Average opportunity cost (~P)
10.0
29.0
29.5
57.0

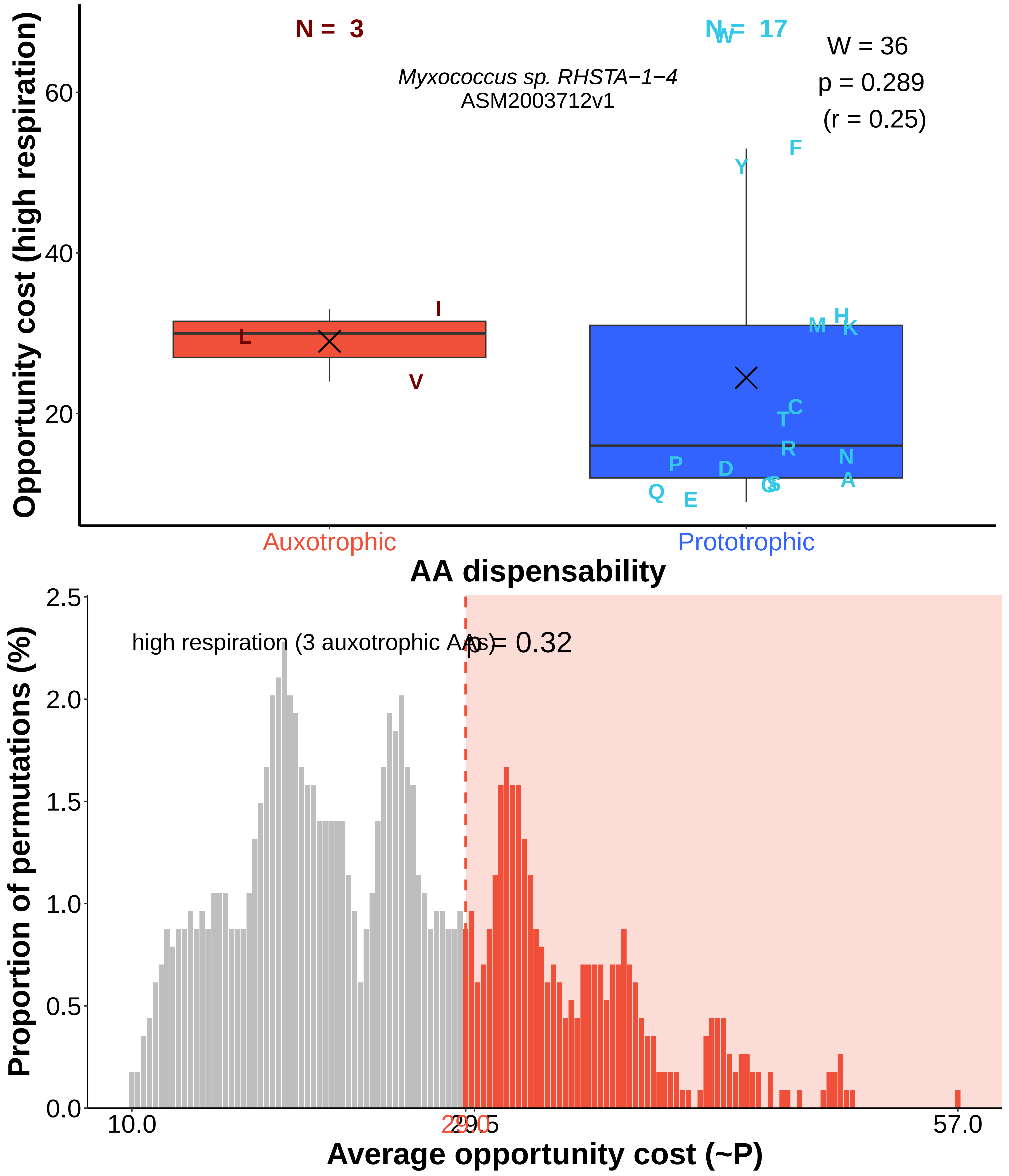
N = 3
N = 17
W
W = 36
p = 0.289
(r = 0.25)
Myxococcus sp. RHSTA−1−4
ASM2003712v1
Opportunity cost (high respiration)
60
40
20
F
Y
I
L
V
M
H
K
C
T
R
P
D
N
Q
E
G
S
A
Auxotrophic
Prototrophic
AA dispensability
Proportion of permutations (%)
2.5
2.0
1.5
1.0
0.5
0.0
high respiration (3 auxotrophic AAs)
p = 0.32
10.0
29.0
29.5
57.0
Average opportunity cost (~P)

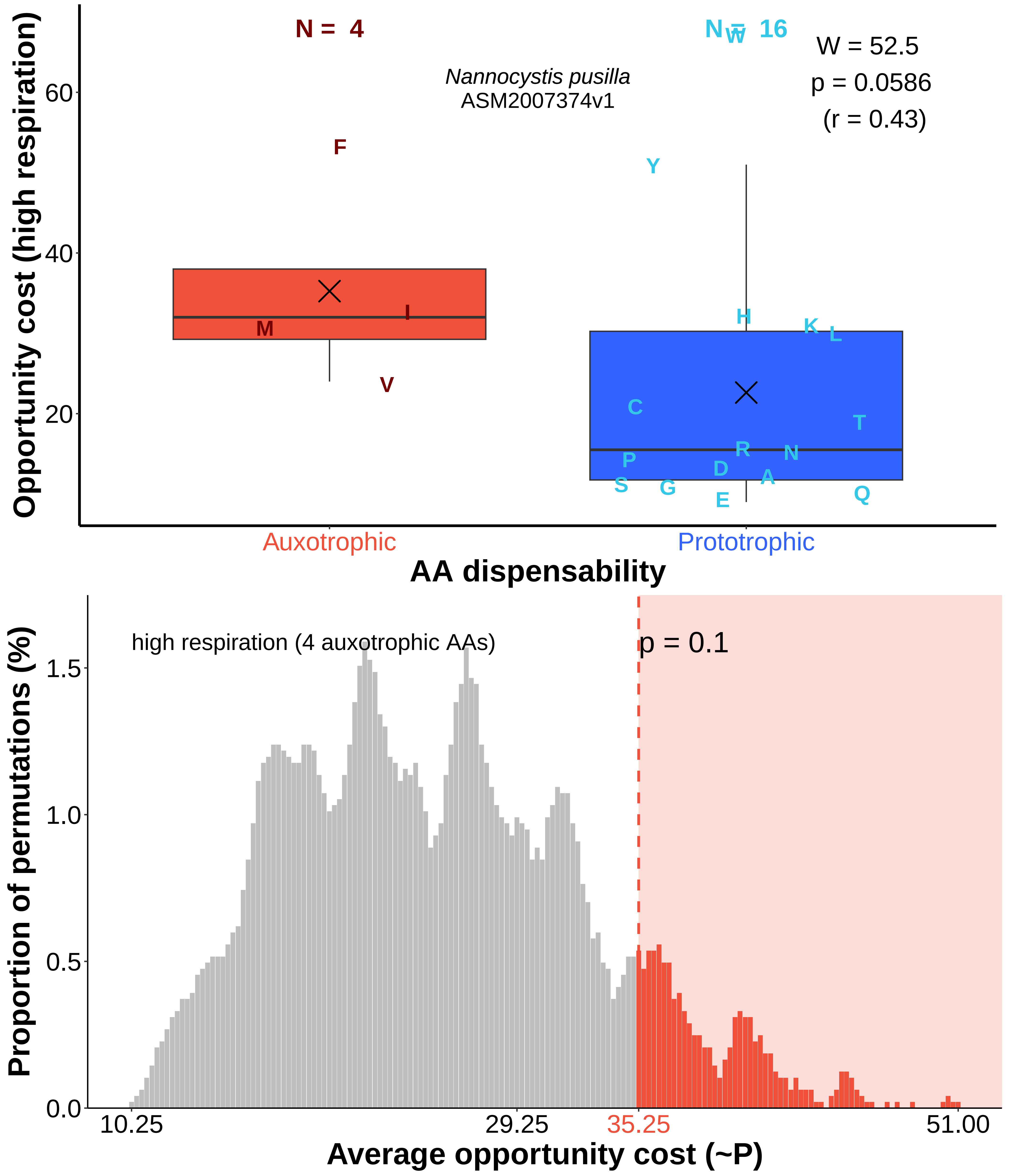
N = 4
N = 16
W
W = 52.5
p = 0.0586
(r = 0.43)
Nannocystis pusilla
ASM2007374v1
F
Y
I
M
V
H
K
L
C
T
R
N
P
D
A
S
G
E
Q
60
40
20
Opportunity cost (high respiration)
Auxotrophic
Prototrophic
AA dispensability
high respiration (4 auxotrophic AAs)
p = 0.1
1.5
1.0
0.5
0.0
Proportion of permutations (%)
10.25
29.25
35.25
51.00
Average opportunity cost (~P)

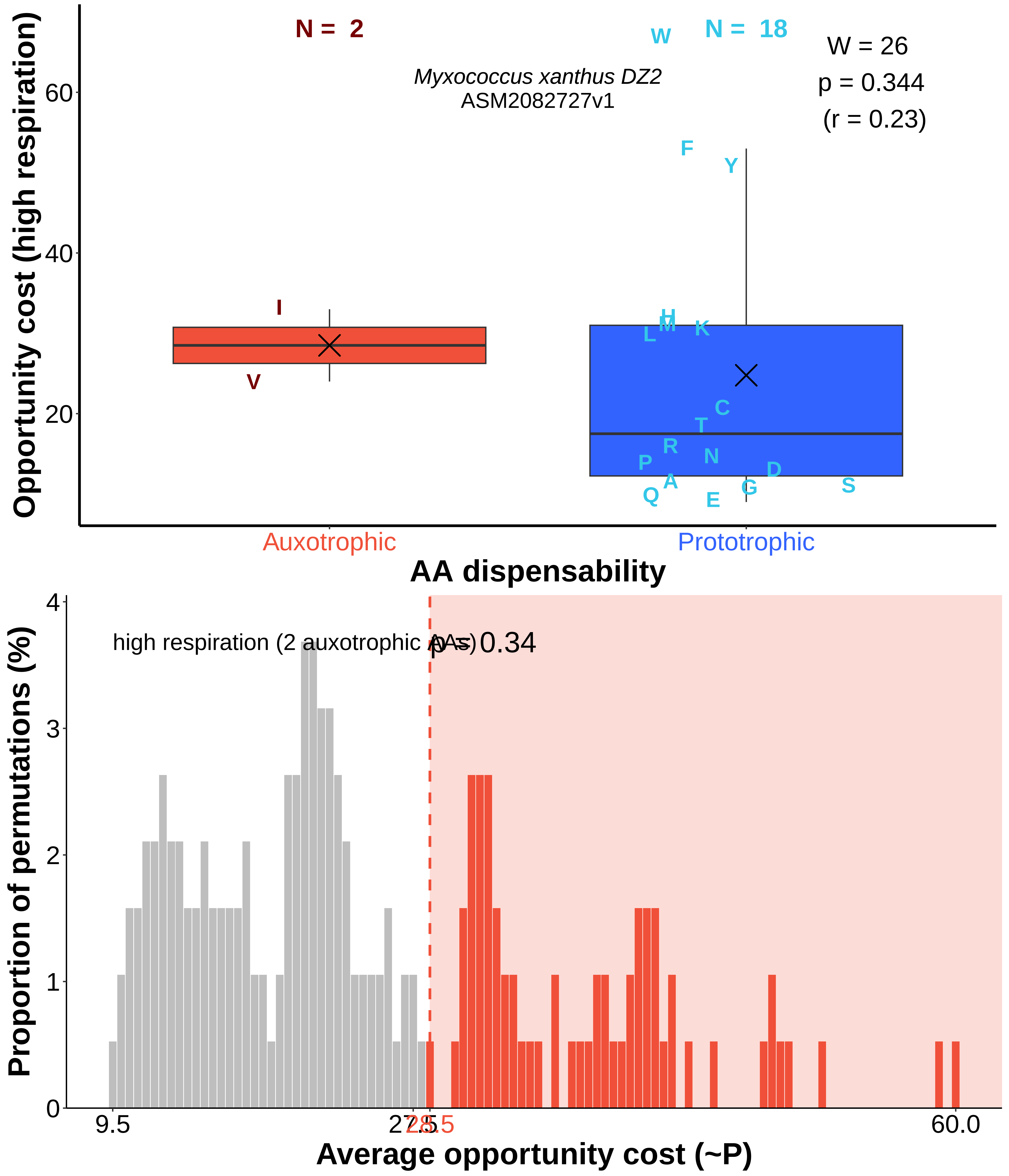

N = 2
N = 18
W = 26
p = 0.344
(r = 0.23)
Myxococcus xanthus DZ2
ASM2082727v1
Opportunity cost (high respiration)
Auxotrophic
Prototrophic
AA dispensability
high respiration (2 auxotrophic AAs)
p = 0.34
Proportion of permutations (%)
9.5
27.5
28.5
60.0
Average opportunity cost (~P)

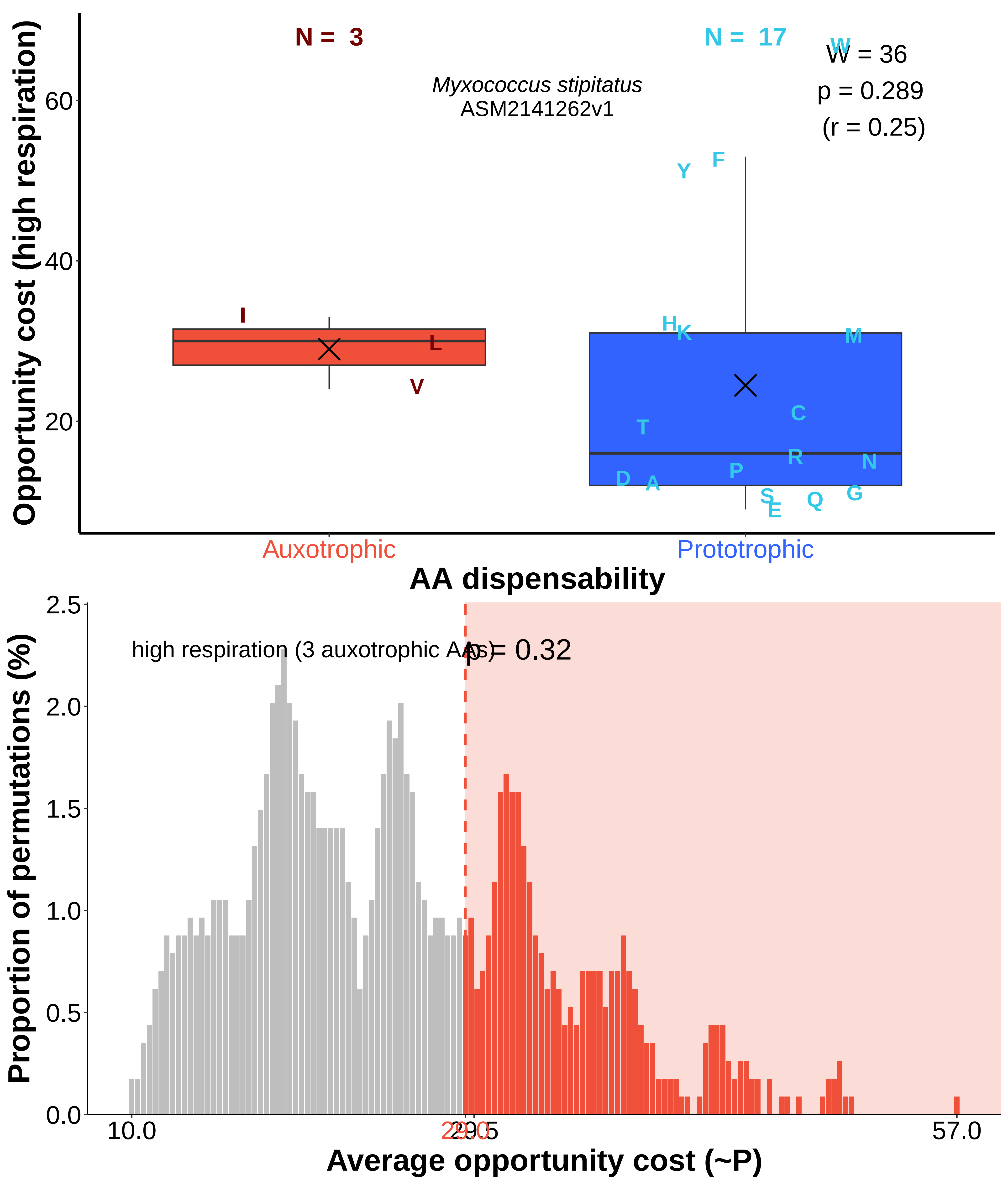

N = 3
N = 17
W = 36
p = 0.289
(r = 0.25)
Myxococcus stipitatus
ASM2141262v1
Opportunity cost (high respiration)
60
40
20
I
L
V
W
Y
F
H
K
M
C
T
R
N
P
D
A
S
E
Q
G
Auxotrophic
Prototrophic
AA dispensability
Proportion of permutations (%)
2.5
2.0
1.5
1.0
0.5
0.0
high respiration (3 auxotrophic AAs)
p = 0.32
10.0
29.0
29.5
57.0
Average opportunity cost (~P)

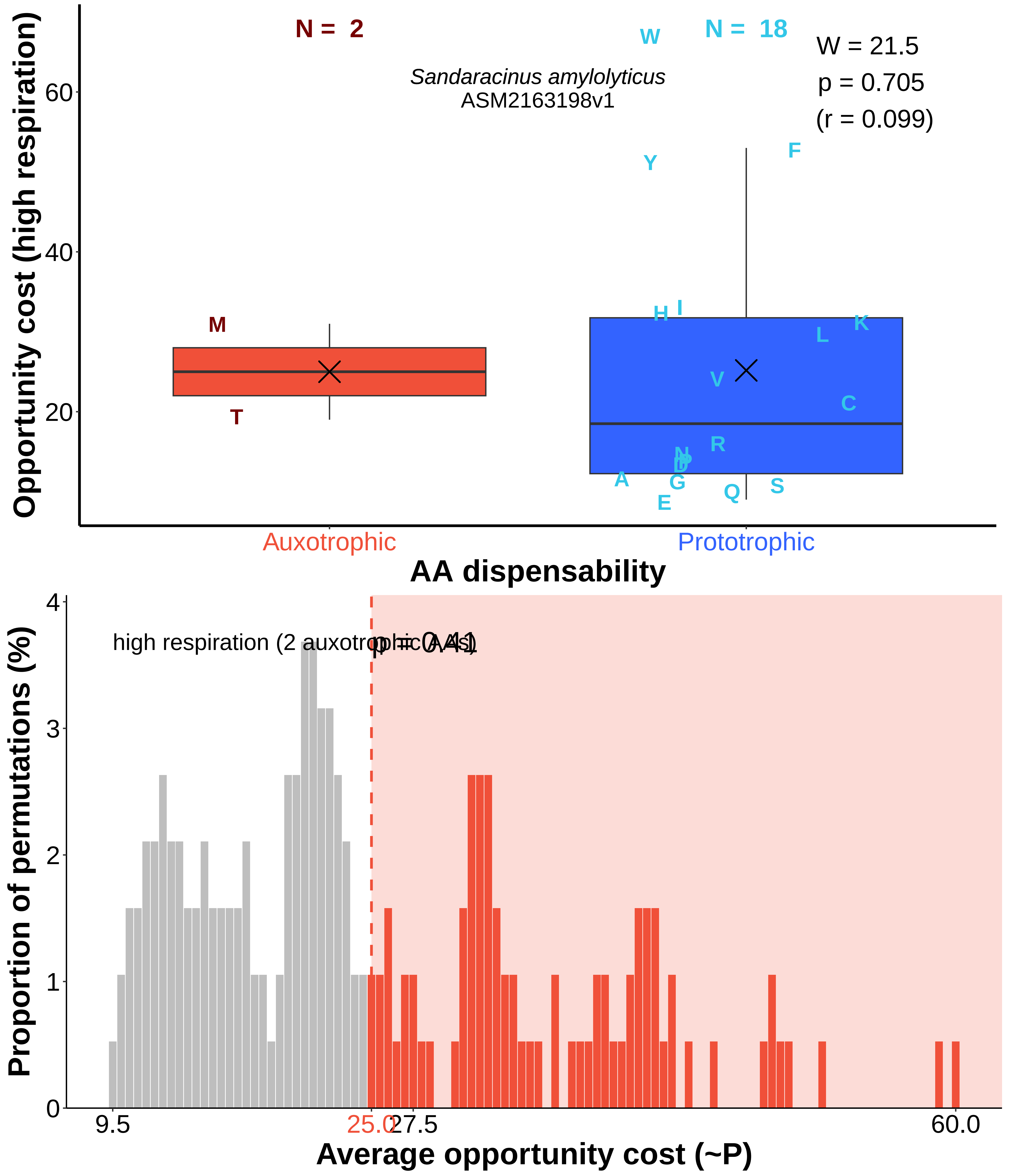
N = 2
N = 18
W = 21.5
p = 0.705
(r = 0.099)
Sandaracinus amylolyticus
ASM2163198v1
Opportunity cost (high respiration)
60
40
20
M
T
W
Y
F
H
I
L
K
V
C
R
N
P
D
A
G
Q
S
E
Auxotrophic
Prototrophic
AA dispensability
high respiration (2 auxotrophic AAs)
p = 0.41
Proportion of permutations (%)
4
3
2
1
0
9.5
25.0
27.5
60.0
Average opportunity cost (~P)

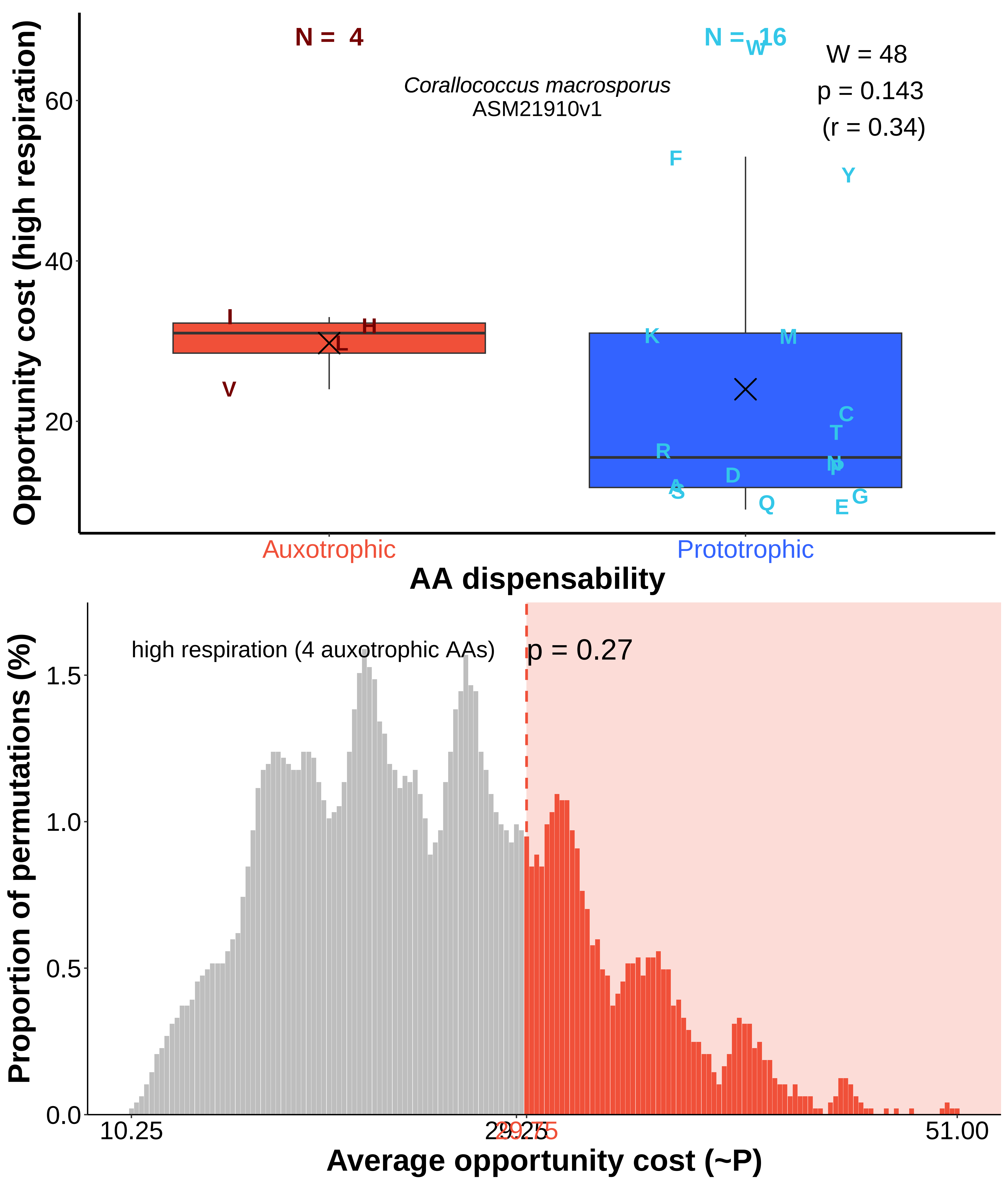

N = 4
N = 16
Corallococcus macrosporus
ASM21910v1
W = 48
p = 0.143
(r = 0.34)
Opportunity cost (high respiration)
Auxotrophic
Prototrophic
AA dispensability
high respiration (4 auxotrophic AAs)
p = 0.27
Proportion of permutations (%)
10.25
29.75
51.00
Average opportunity cost (~P)

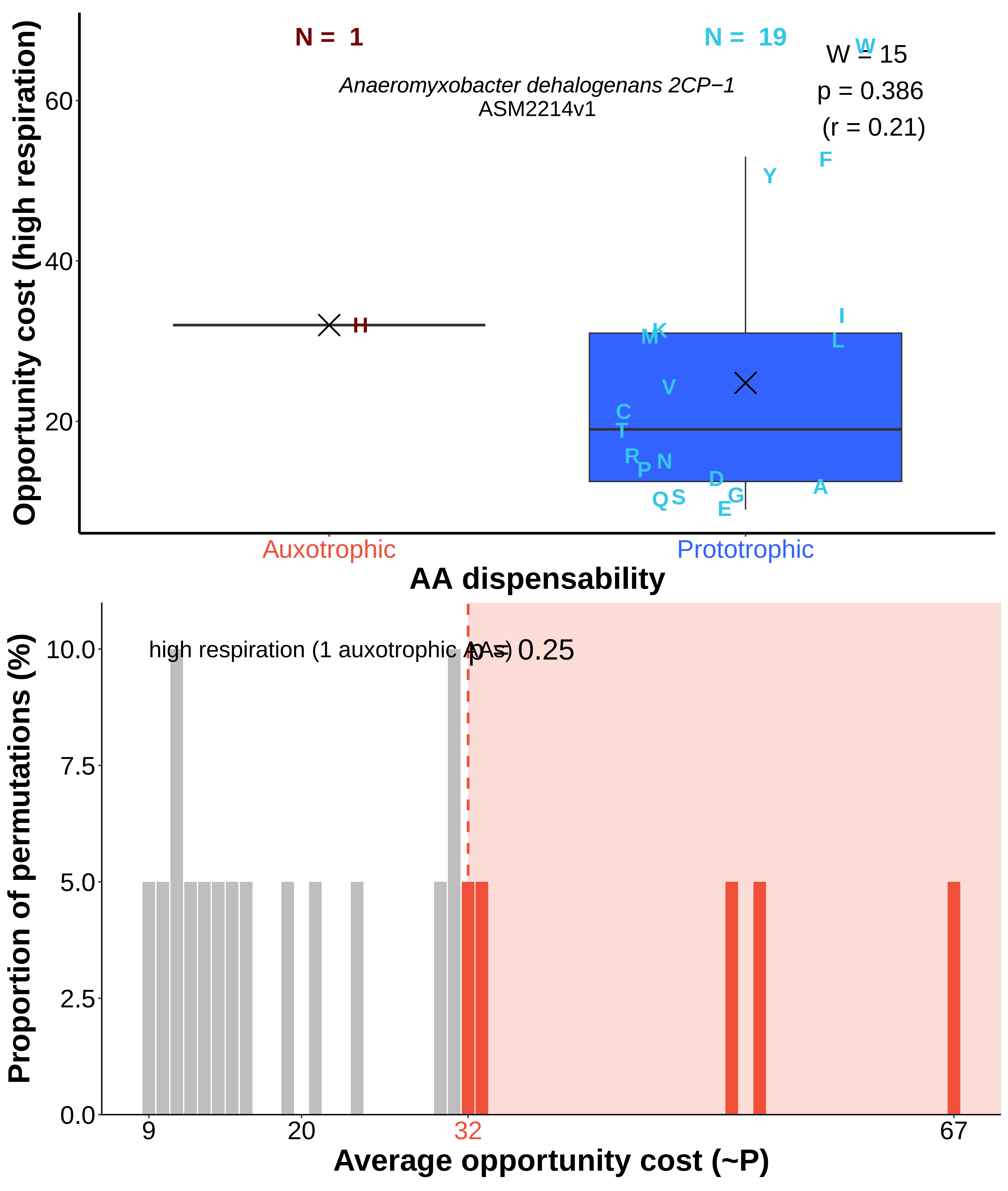

N = 1
N = 19
Anaeromyxobacter dehalogenans 2CP−1
ASM2214v1
W = 15
p = 0.386
(r = 0.21)
Opportunity cost (high respiration)
60
40
20
H
Y W F I L K M V C T R P N D A Q S G E
Auxotrophic
Prototrophic
AA dispensability
high respiration (1 auxotrophic AAs)
p = 0.25
Proportion of permutations (%)
10.0
7.5
5.0
2.5
0.0
9
20
32
67
Average opportunity cost (~P)

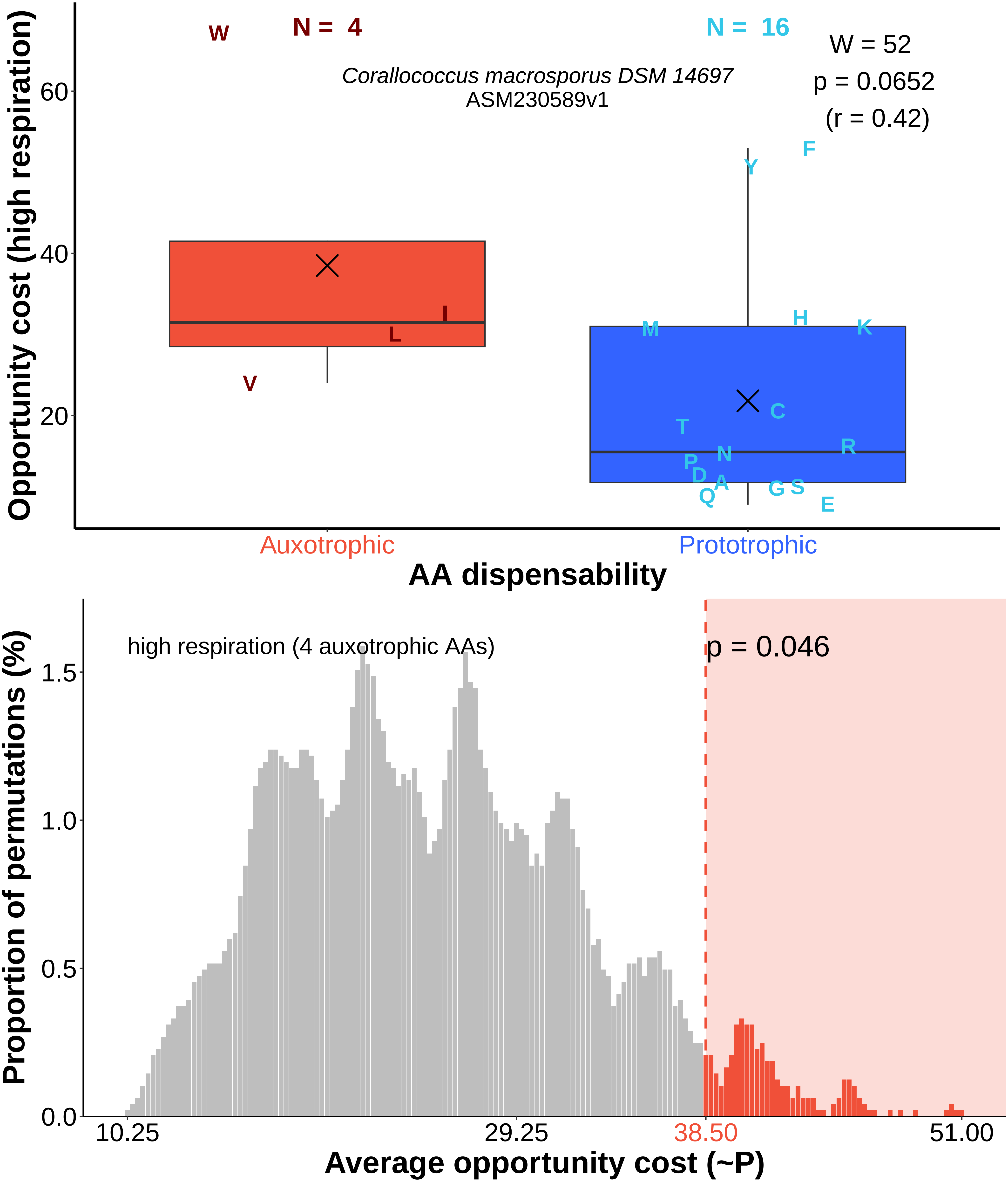

W
N = 4
N = 16
W = 52
p = 0.0652
(r = 0.42)
Corallococcus macrosporus DSM 14697
ASM230589v1
Opportunity cost (high respiration)
60
40
20
F
Y
I
L
V
M
H
K
C
T
N
R
P
D
A
Q
G
S
E
Auxotrophic
Prototrophic
AA dispensability
high respiration (4 auxotrophic AAs)
p = 0.046
Proportion of permutations (%)
1.5
1.0
0.5
0.0
10.25
29.25
38.50
51.00
Average opportunity cost (~P)

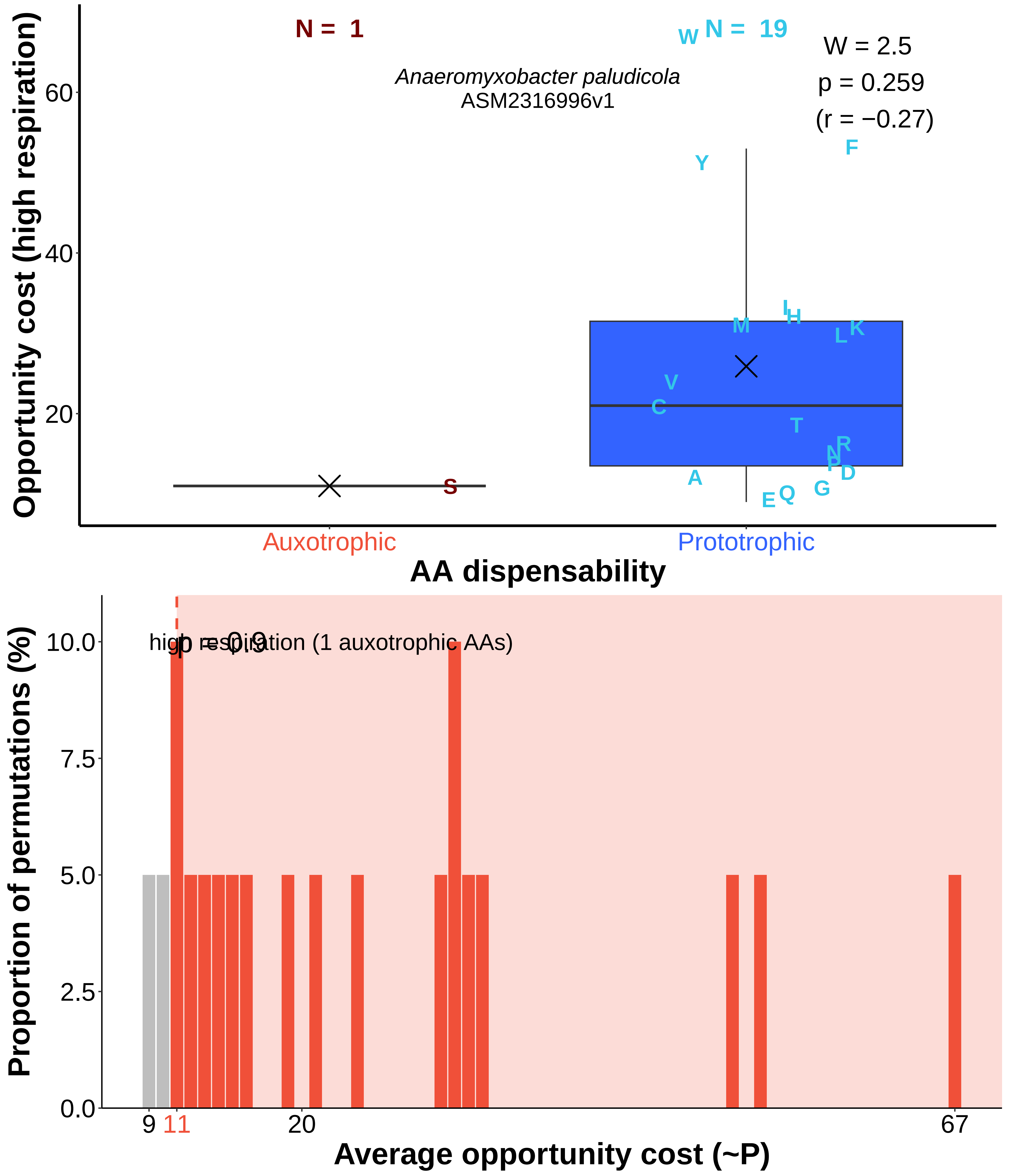

N = 1
N = 19
W
W = 2.5
p = 0.259
(r = −0.27)
Anaeromyxobacter paludicola
ASM2316996v1
Opportunity cost (high respiration)
60
40
20
Y
F
I
H
M
K
L
V
C
T
R
N
P
D
A
S
E
Q
G
Auxotrophic
Prototrophic
AA dispensability
Proportion of permutations (%)
10.0
7.5
5.0
2.5
0.0
high respiration (1 auxotrophic AAs)
p = 0.9
9
11
20
67
Average opportunity cost (~P)

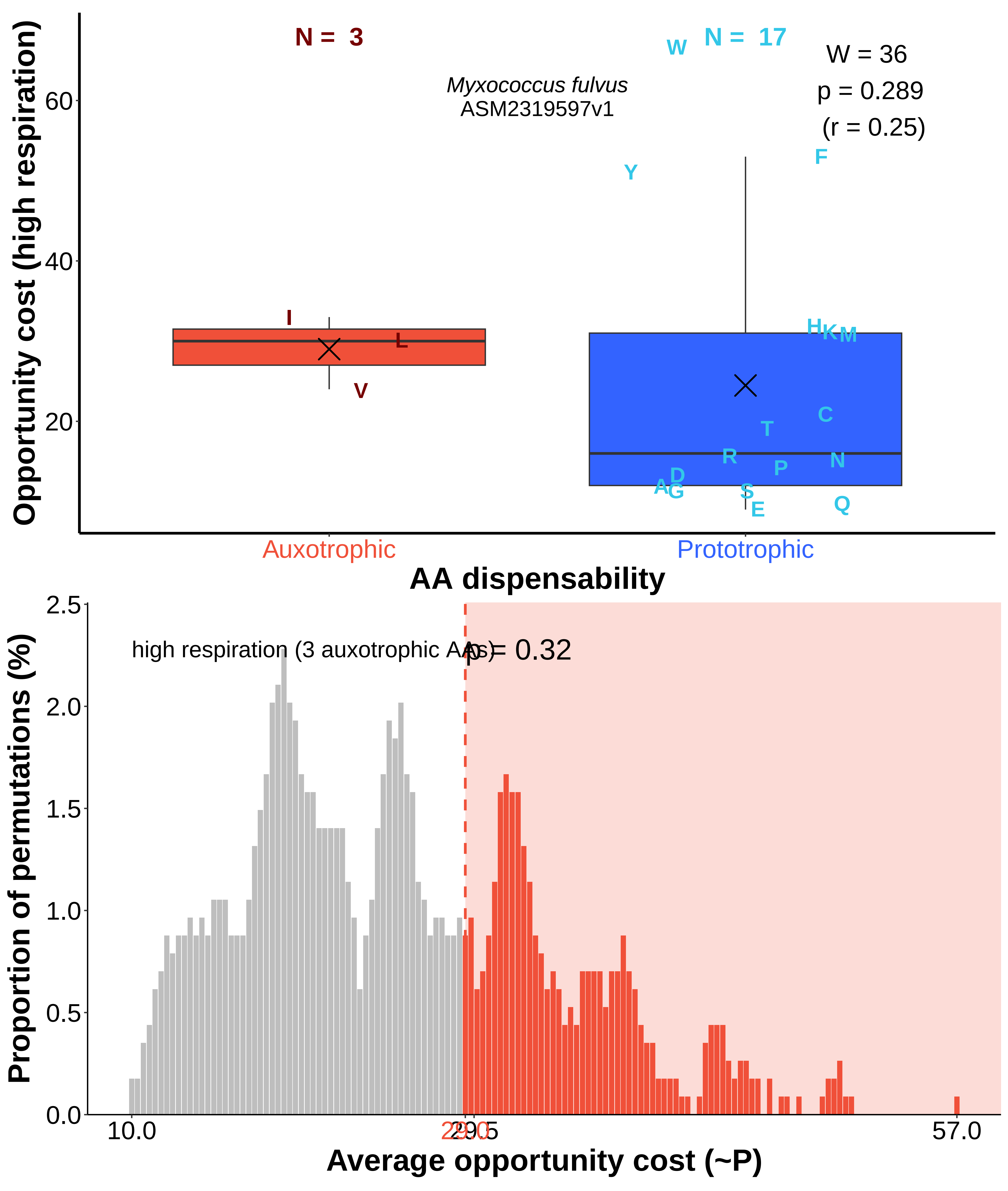

N = 3
N = 17
W
W = 36
p = 0.289
(r = 0.25)
Myxococcus fulvus
ASM2319597v1
Opportunity cost (high respiration)
Y
F
I
L
V
H K M
C
T
R
P
N
D
A G
S
E
Q
60
40
20
Auxotrophic
Prototrophic
AA dispensability
high respiration (3 auxotrophic AAs)
p = 0.32
Proportion of permutations (%)
2.5
2.0
1.5
1.0
0.5
0.0
10.0
29.0
29.5
57.0
Average opportunity cost (~P)

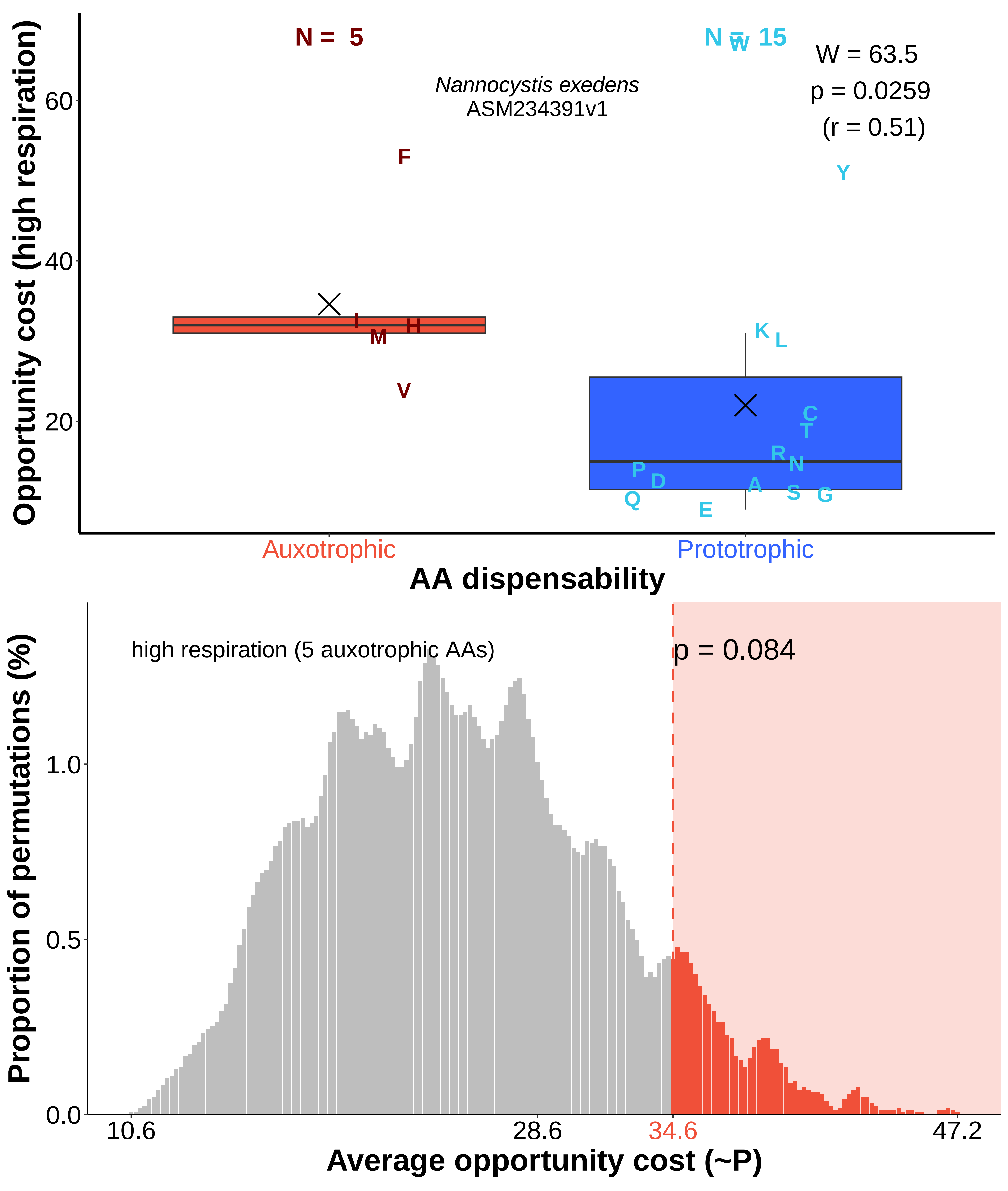
N = 5
N = 15
W
Nannocystis exedens
ASM234391v1
W = 63.5
p = 0.0259
(r = 0.51)
F
Y
I
M
H
V
K
L
C
T
R
N
P
D
A
S
G
Q
E
Opportunity cost (high respiration)
60
40
20
Auxotrophic
Prototrophic
AA dispensability
high respiration (5 auxotrophic AAs)
p = 0.084
Proportion of permutations (%)
1.0
0.5
0.0
10.6
28.6
34.6
47.2
Average opportunity cost (~P)

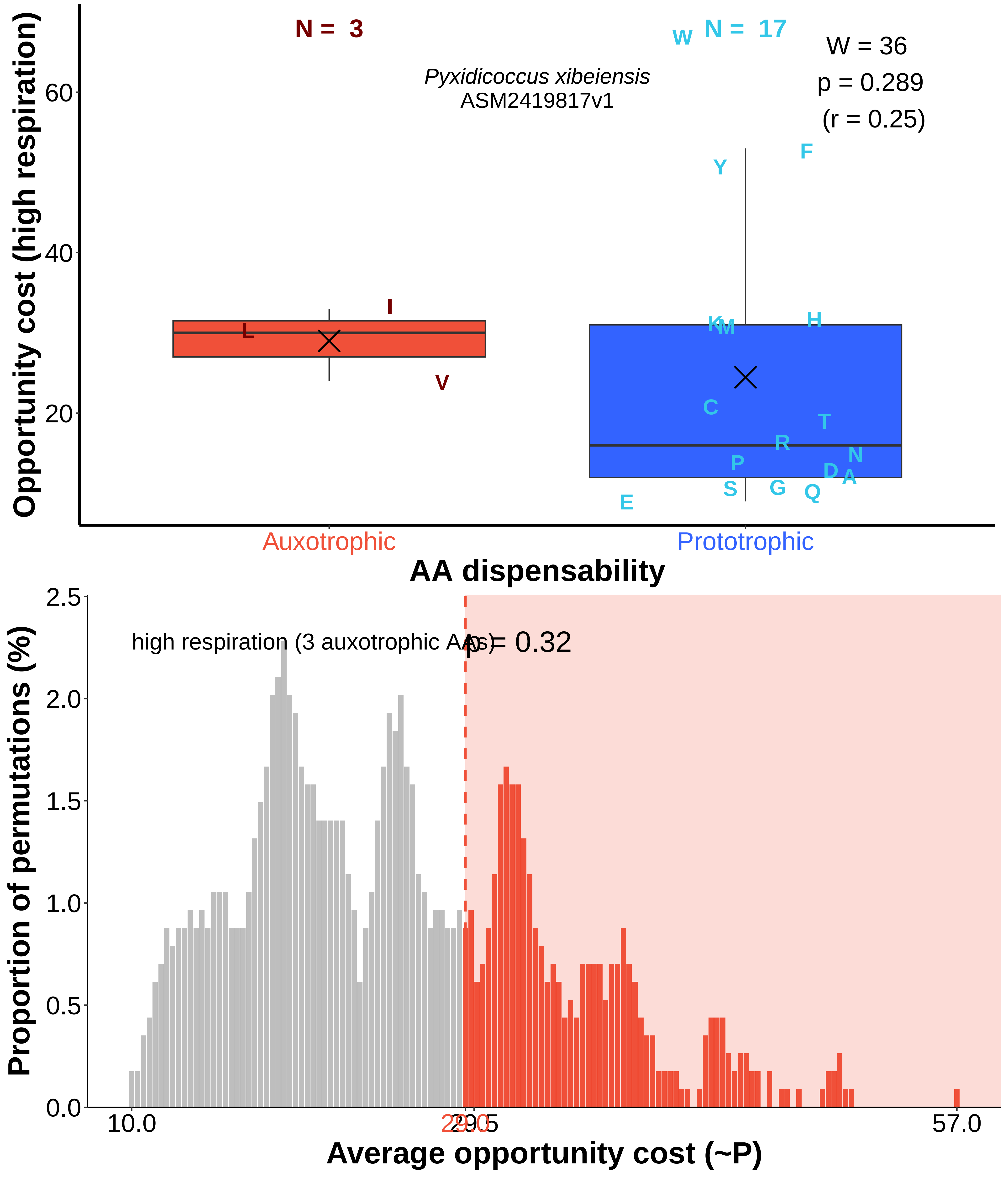
N = 3
N = 17
W
Pyxidicoccus xibeiensis
ASM2419817v1
W = 36
p = 0.289
(r = 0.25)
Opportunity cost (high respiration)
60
40
20
L
I
V
Y
F
K
M
H
C
T
R
N
P
D
A
S
G
Q
E
Auxotrophic
Prototrophic
AA dispensability
high respiration (3 auxotrophic AAs)
p = 0.32
Proportion of permutations (%)
2.5
2.0
1.5
1.0
0.5
0.0
10.0
29.0
29.5
57.0
Average opportunity cost (~P)

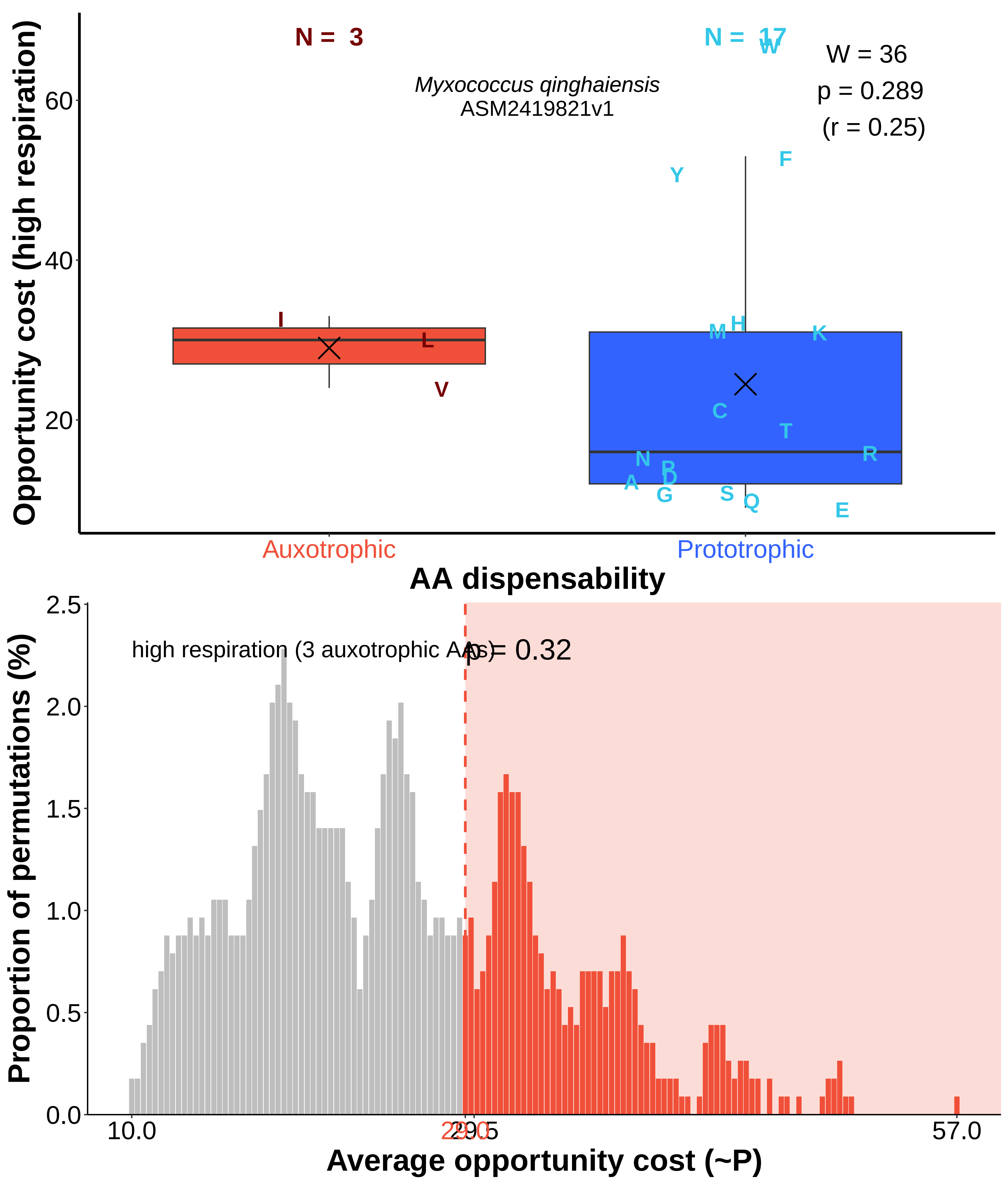
N = 3
N = 17
Myxococcus qinghaiensis
ASM2419821v1
W = 36
p = 0.289
(r = 0.25)
Opportunity cost (high respiration)
60
40
20
I
L
V
Y
F
W
M
H
K
C
T
N
R
B
D
A
G
S
Q
E
Auxotrophic
Prototrophic
AA dispensability
high respiration (3 auxotrophic AAs)
p = 0.32
Proportion of permutations (%)
2.5
2.0
1.5
1.0
0.5
0.0
10.0
29.0
29.5
57.0
Average opportunity cost (~P)

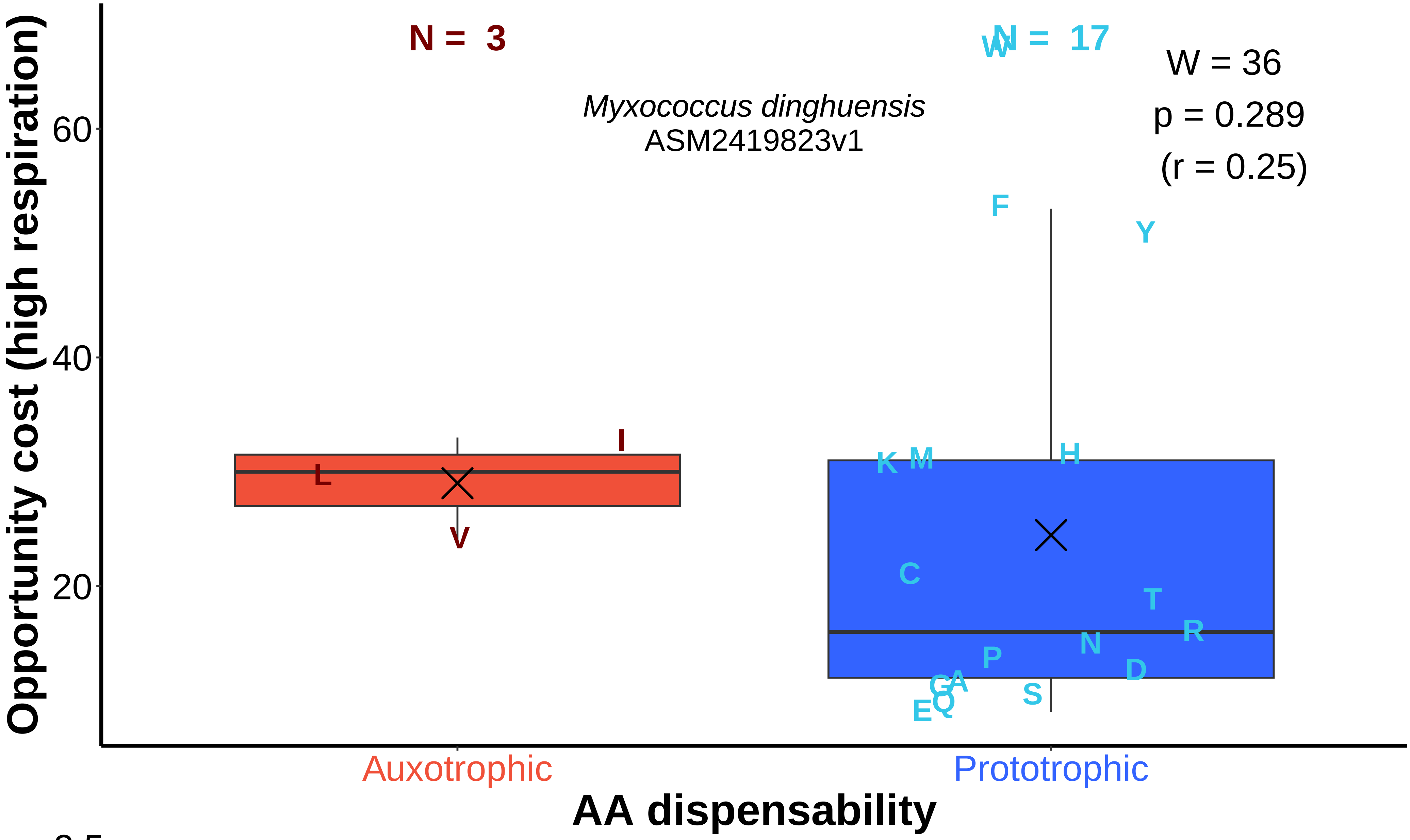

N = 3
N = 17
W
Myxococcus dinghuensis
ASM2419823v1
W = 36
p = 0.289
(r = 0.25)
Opportunity cost (high respiration)
60
40
20
F
Y
I
L
V
K
M
H
C
T
R
N
P
D
G
A
S
E
Q
Auxotrophic
Prototrophic
AA dispensability

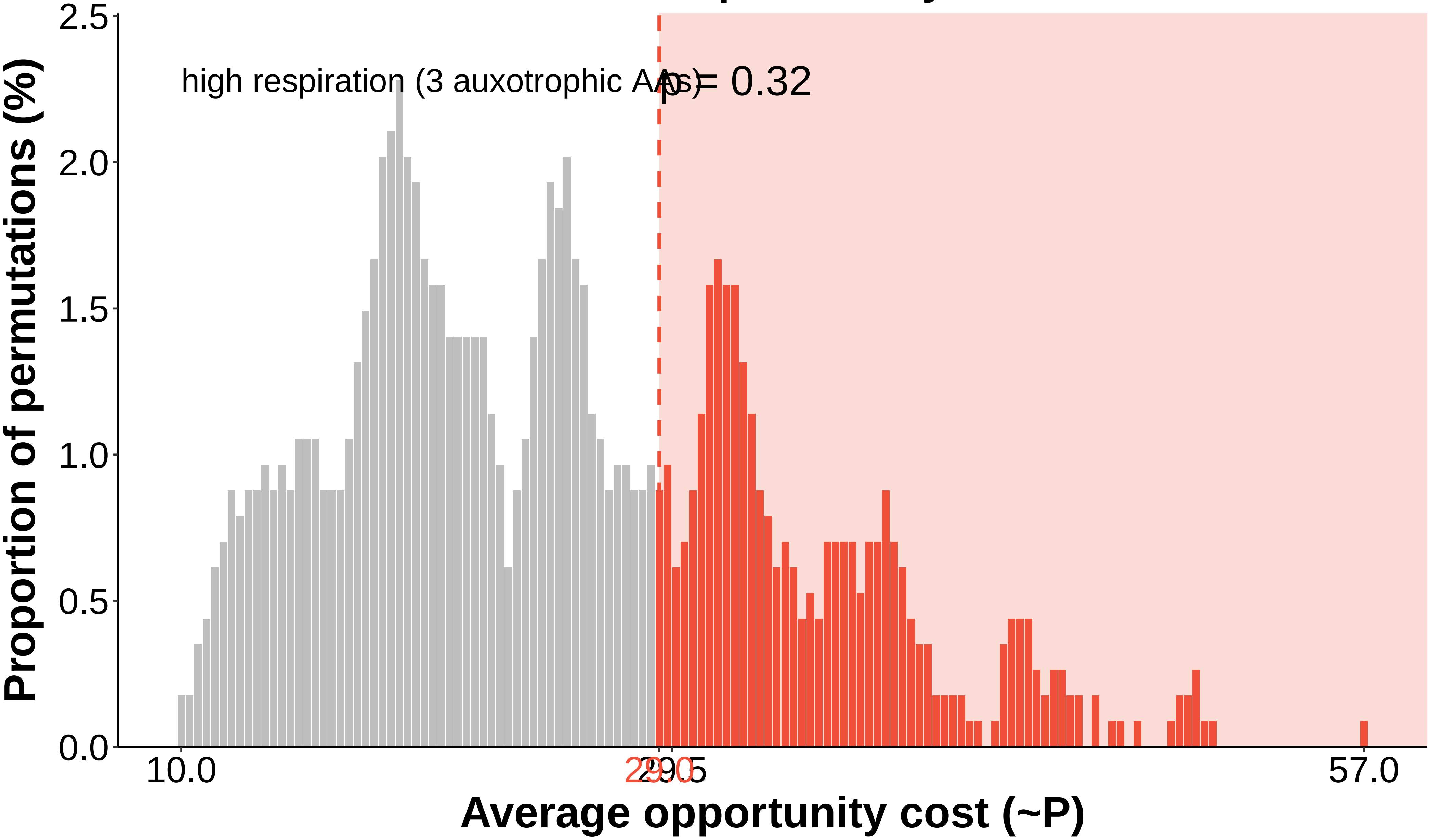

high respiration (3 auxotrophic AAs)
p = 0.32
Proportion of permutations (%)
2.5
2.0
1.5
1.0
0.5
0.0
10.0
29.0
29.5
57.0
Average opportunity cost (~P)

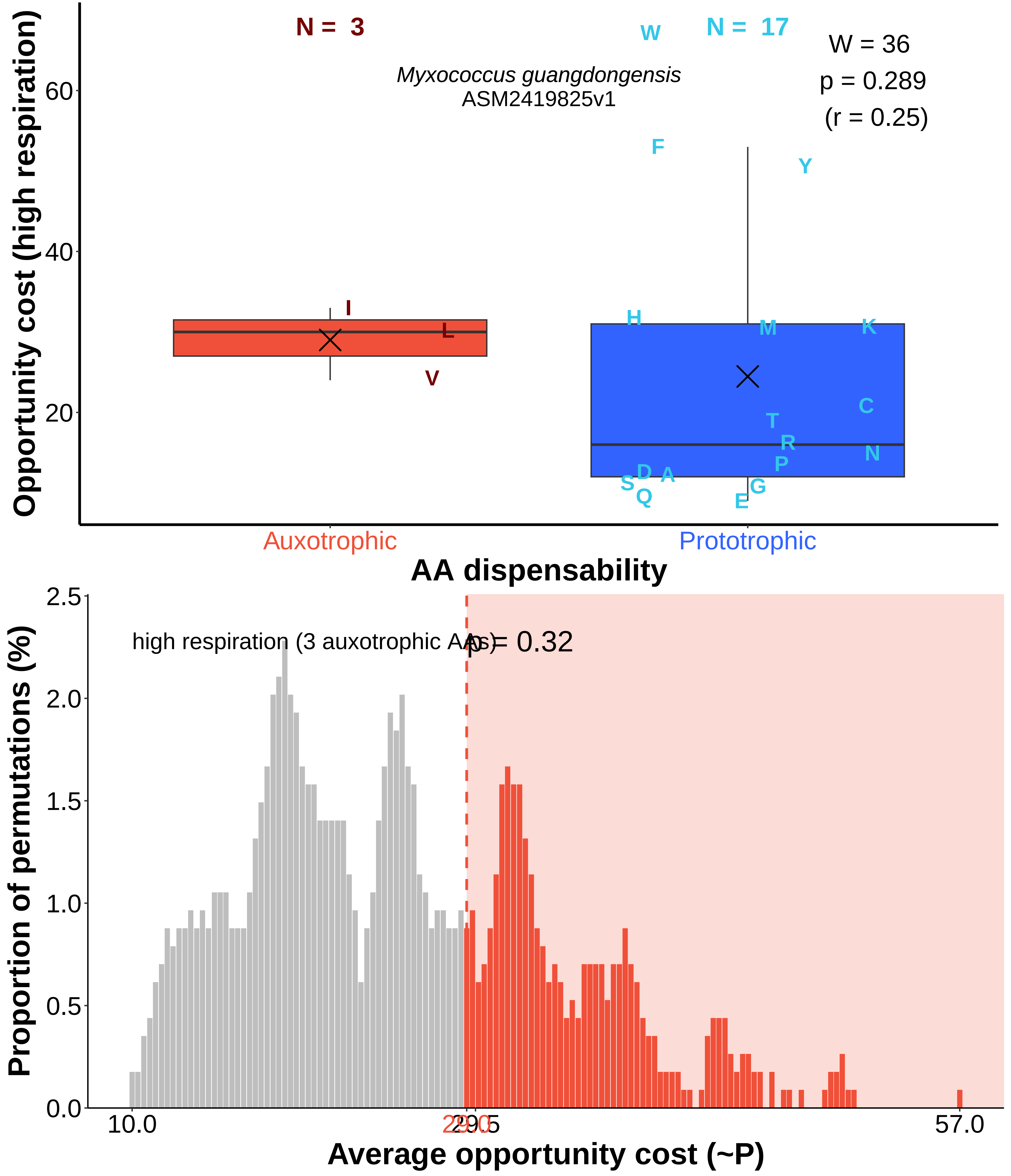
N = 3
W
N = 17
W = 36
p = 0.289
(r = 0.25)
Myxococcus guangdongensis
ASM2419825v1
Opportunity cost (high respiration)
60
40
20
F
Y
I
H
M
K
L
V
C
T
R
N
P
D
A
S
Q
G
E
Auxotrophic
Prototrophic
AA dispensability
Proportion of permutations (%)
2.5
2.0
1.5
1.0
0.5
0.0
high respiration (3 auxotrophic AAs)
p = 0.32
10.0
29.0
29.5
57.0
Average opportunity cost (~P)

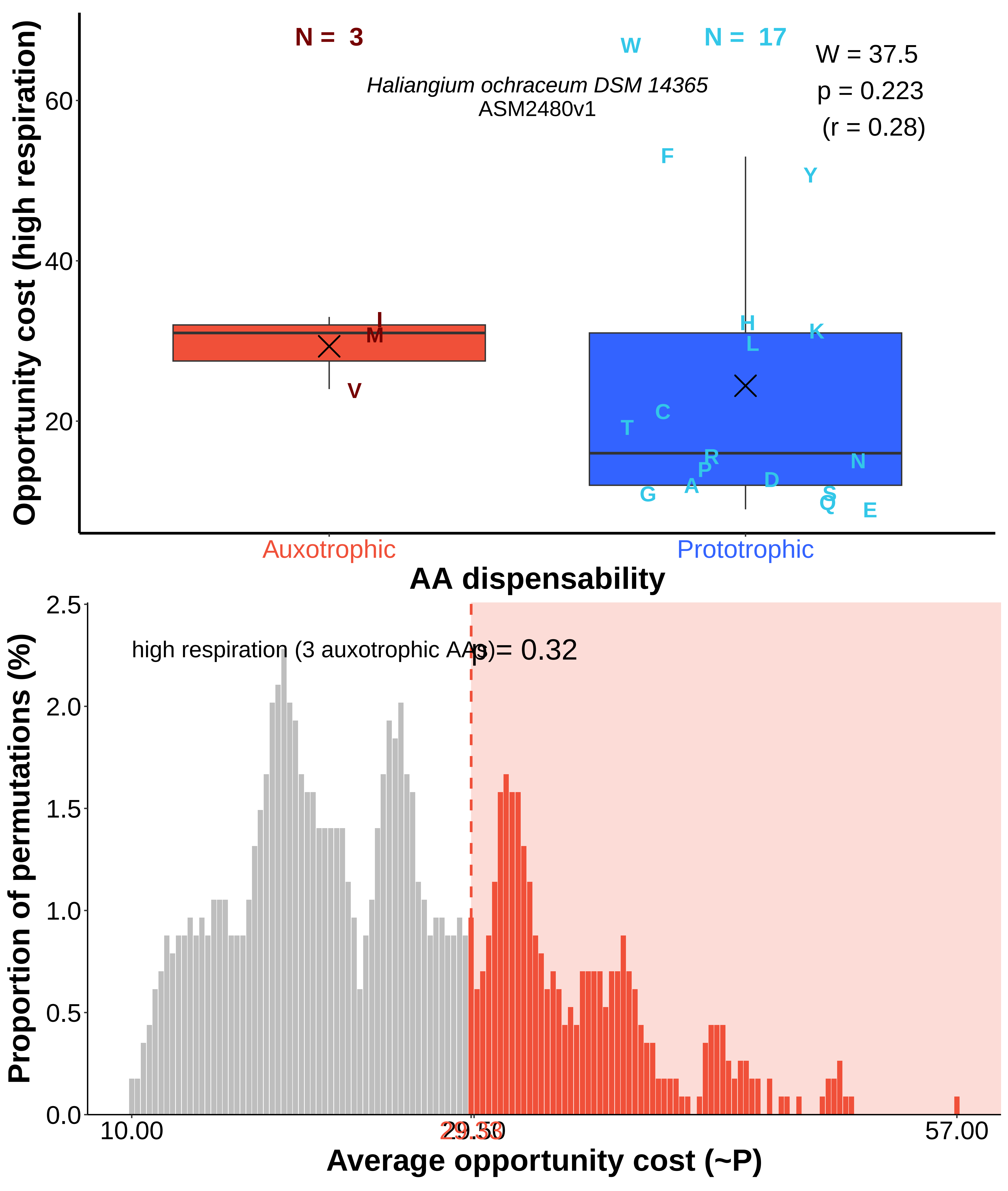
N = 3
N = 17
W
W = 37.5
p = 0.223
(r = 0.28)
Haliangium ochraceum DSM 14365
ASM2480v1
Opportunity cost (high respiration)
Auxotrophic
Prototrophic
AA dispensability
high respiration (3 auxotrophic AAs)
p = 0.32
Proportion of permutations (%)
Average opportunity cost (~P)
10.00
29.50
29.33
57.00

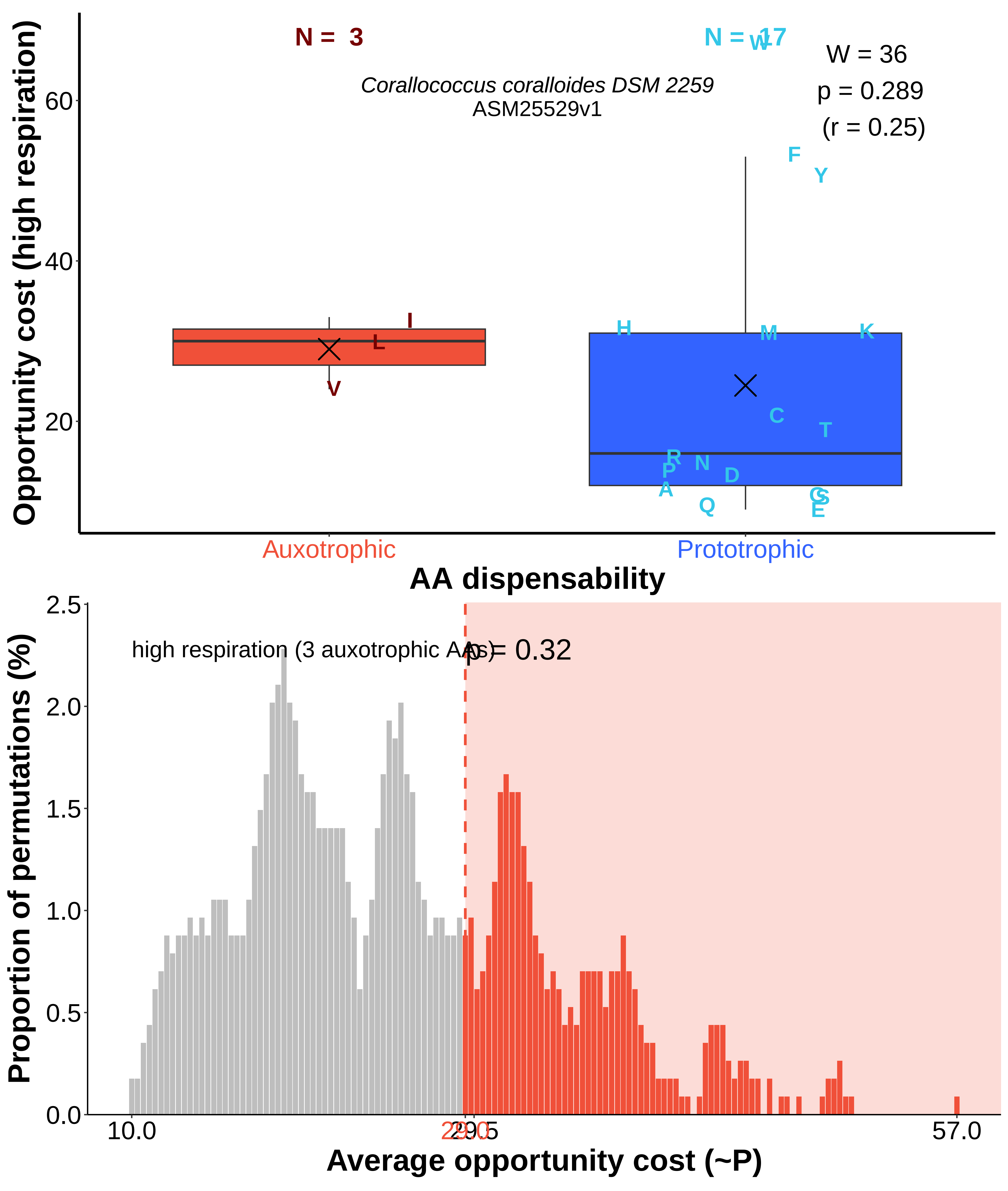

N = 3
N = 17
W = 36
p = 0.289
(r = 0.25)
Corallococcus coralloides DSM 2259
ASM25529v1
Opportunity cost (high respiration)
60
40
20
I
L
V
H
M
K
F
Y
W
C
T
R
N
P
D
A
Q
G
S
E
Auxotrophic
Prototrophic
AA dispensability
high respiration (3 auxotrophic AAs)
p = 0.32
Proportion of permutations (%)
2.5
2.0
1.5
1.0
0.5
0.0
10.0
29.0
29.5
57.0
Average opportunity cost (~P)

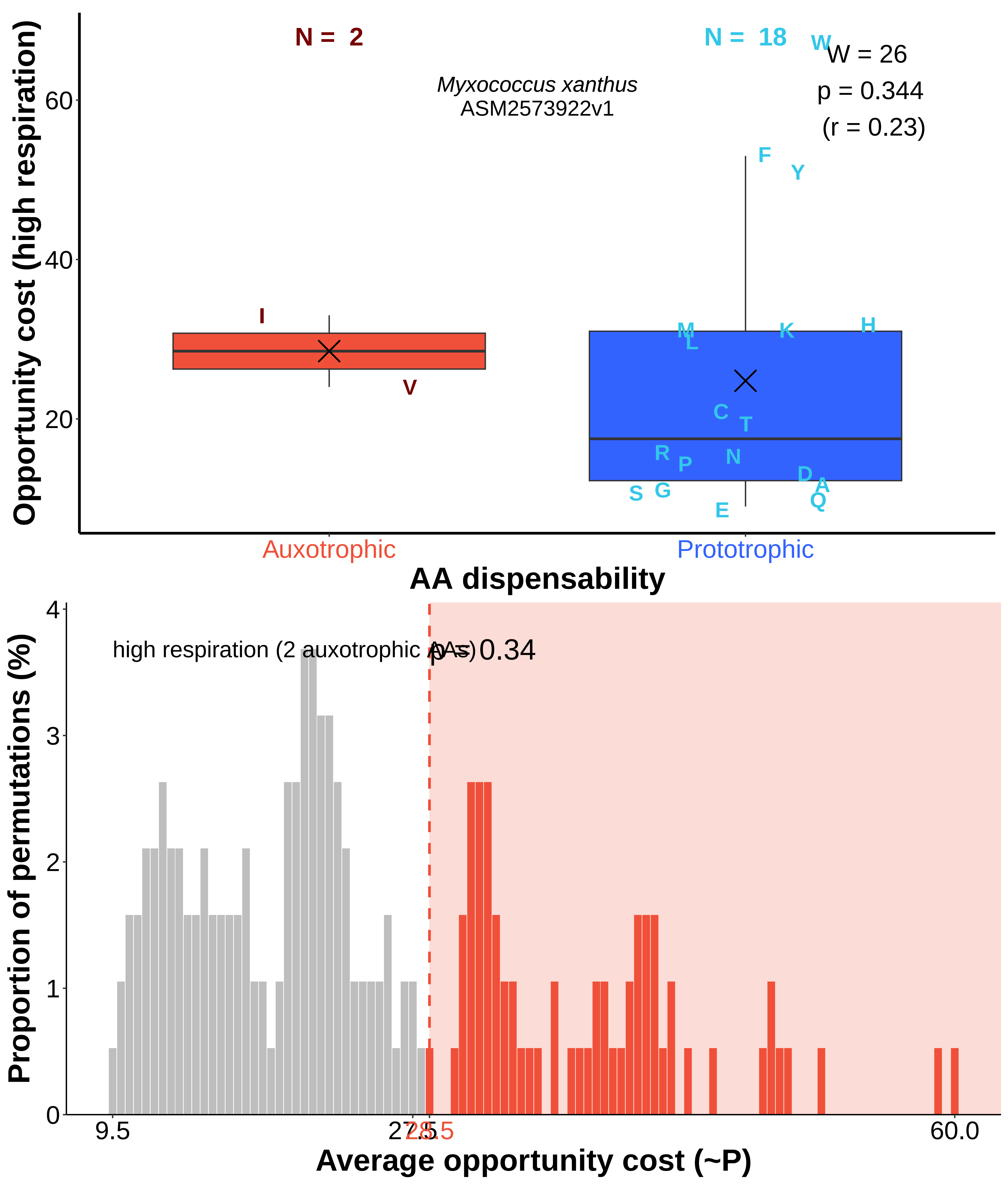

N = 2
N = 18
W
W = 26
p = 0.344
(r = 0.23)
Myxococcus xanthus
ASM2573922v1
Opportunity cost (high respiration)
60
40
20
I
V
M
L
K
H
F
Y
C
T
R
P
N
D
A
S
G
Q
E
Auxotrophic
Prototrophic
AA dispensability
Proportion of permutations (%)
4
3
2
1
0
high respiration (2 auxotrophic AAs)
p = 0.34
9.5
27.5
28.5
60.0
Average opportunity cost (~P)

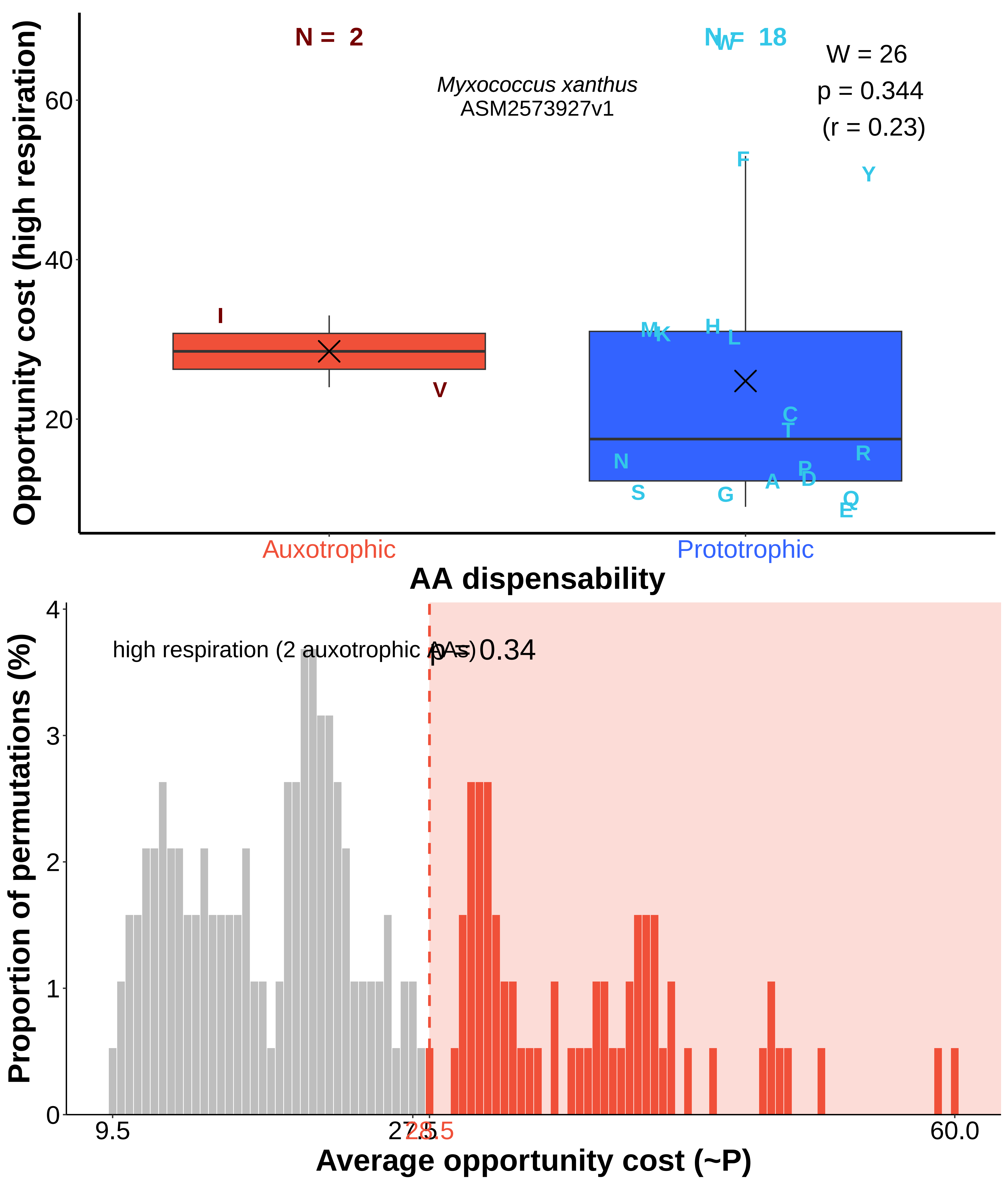
N = 2
N = 18
W = 26
p = 0.344
(r = 0.23)
Myxococcus xanthus
ASM2573927v1
Opportunity cost (high respiration)
Auxotrophic
Prototrophic
AA dispensability
high respiration (2 auxotrophic AAs)
p = 0.34
Proportion of permutations (%)
Average opportunity cost (~P)

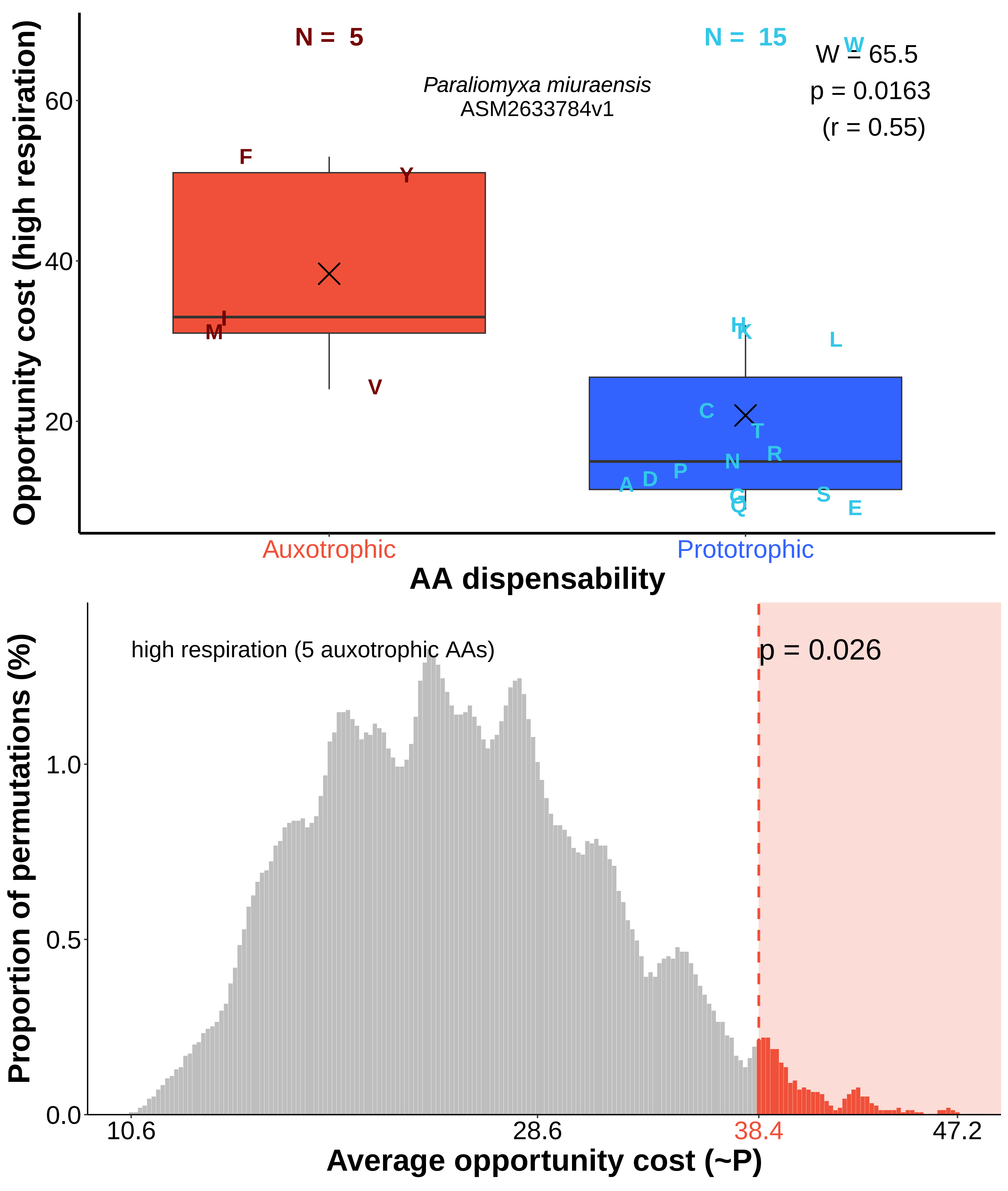

N = 5
N = 15
Paraliomyxa miuraensis
ASM2633784v1
W = 65.5
p = 0.0163
(r = 0.55)
Opportunity cost (high respiration)
60
40
20
F
Y
I
M
V
W
H
K
L
C
T
R
N
P
D
A
G
Q
S
E
Auxotrophic
Prototrophic
AA dispensability
high respiration (5 auxotrophic AAs)
p = 0.026
Proportion of permutations (%)
1.0
0.5
0.0
10.6
28.6
38.4
47.2
Average opportunity cost (~P)

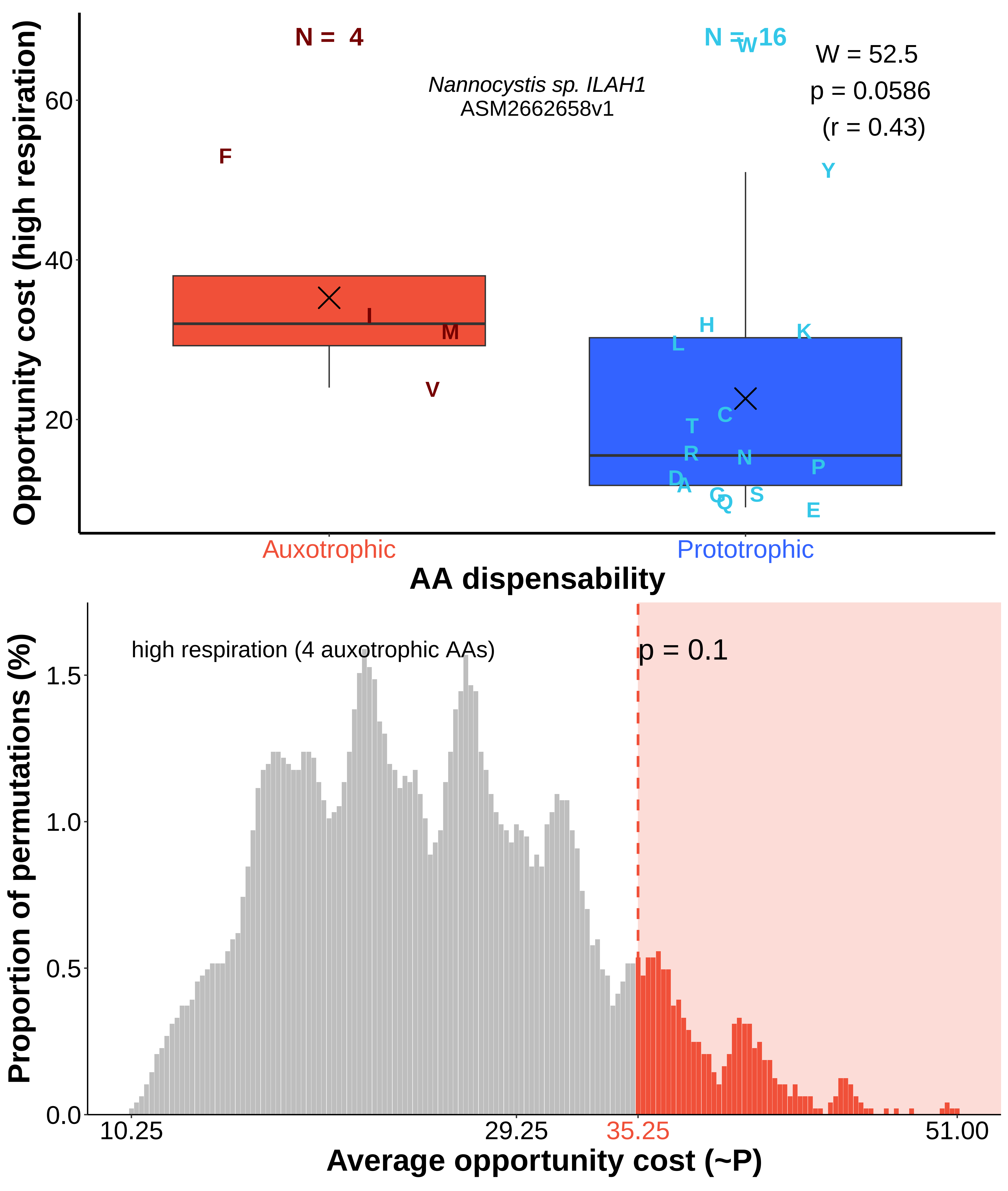

N = 4
N = 16
W
W = 52.5
p = 0.0586
(r = 0.43)
Nannocystis sp. ILAH1
ASM2662658v1
F
I
M
V
H
K
L
Y
C
T
R
N
P
D
A
G
Q
S
E
60
40
20
Opportunity cost (high respiration)
Auxotrophic
Prototrophic
AA dispensability
high respiration (4 auxotrophic AAs)
p = 0.1
1.5
1.0
0.5
0.0
Proportion of permutations (%)
10.25
29.25
35.25
51.00
Average opportunity cost (~P)

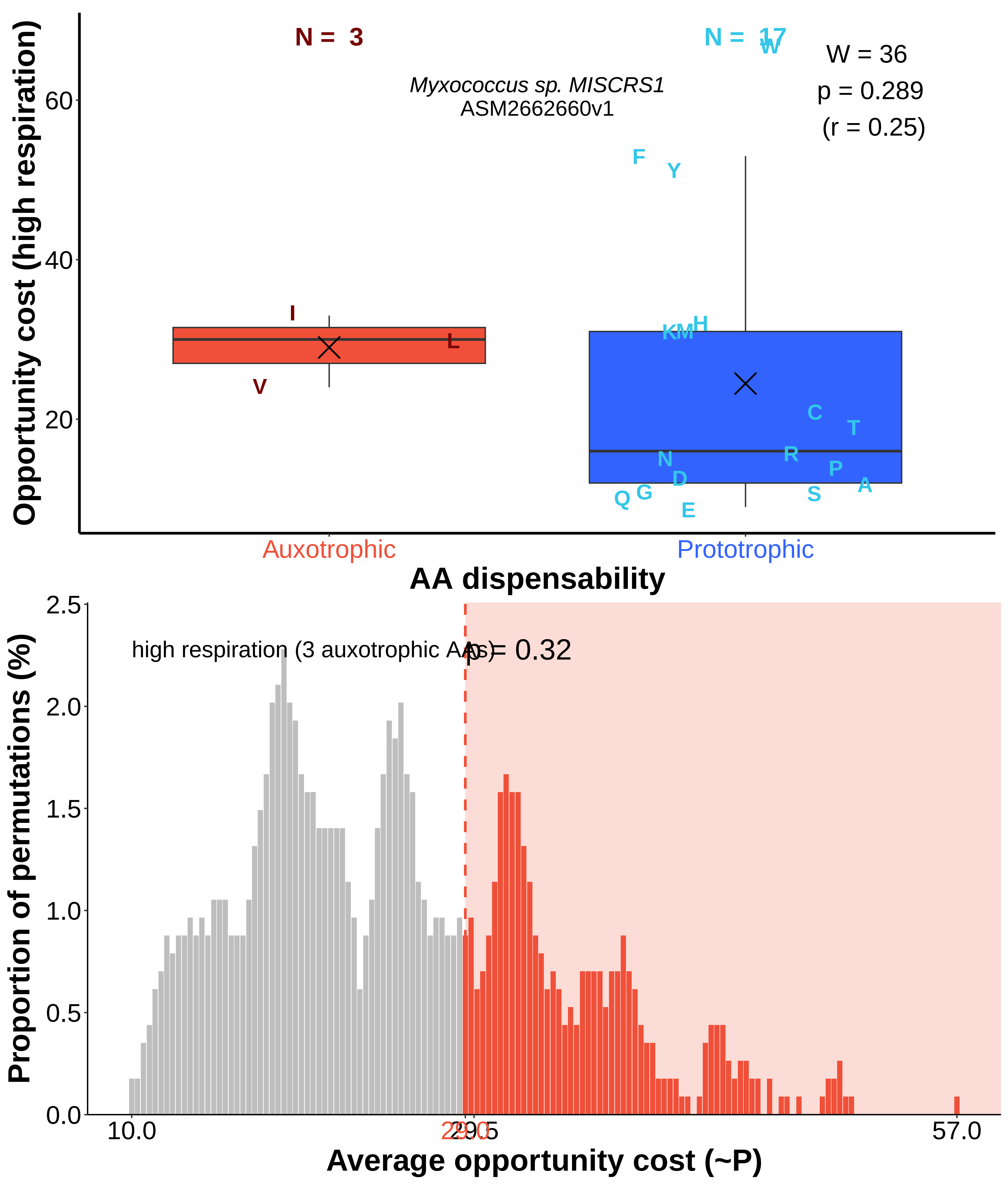

N = 3
N = 17
Myxococcus sp. MISCRS1
ASM2662660v1
W = 36
p = 0.289
(r = 0.25)
Opportunity cost (high respiration)
60
40
20
F
Y
W
I
L
V
K M H
C
T
R
P
N
D
Q G
S
A
E
Auxotrophic
Prototrophic
AA dispensability
Proportion of permutations (%)
2.5
2.0
1.5
1.0
0.5
0.0
high respiration (3 auxotrophic AAs)
p = 0.32
10.0
29.0
29.5
57.0
Average opportunity cost (~P)

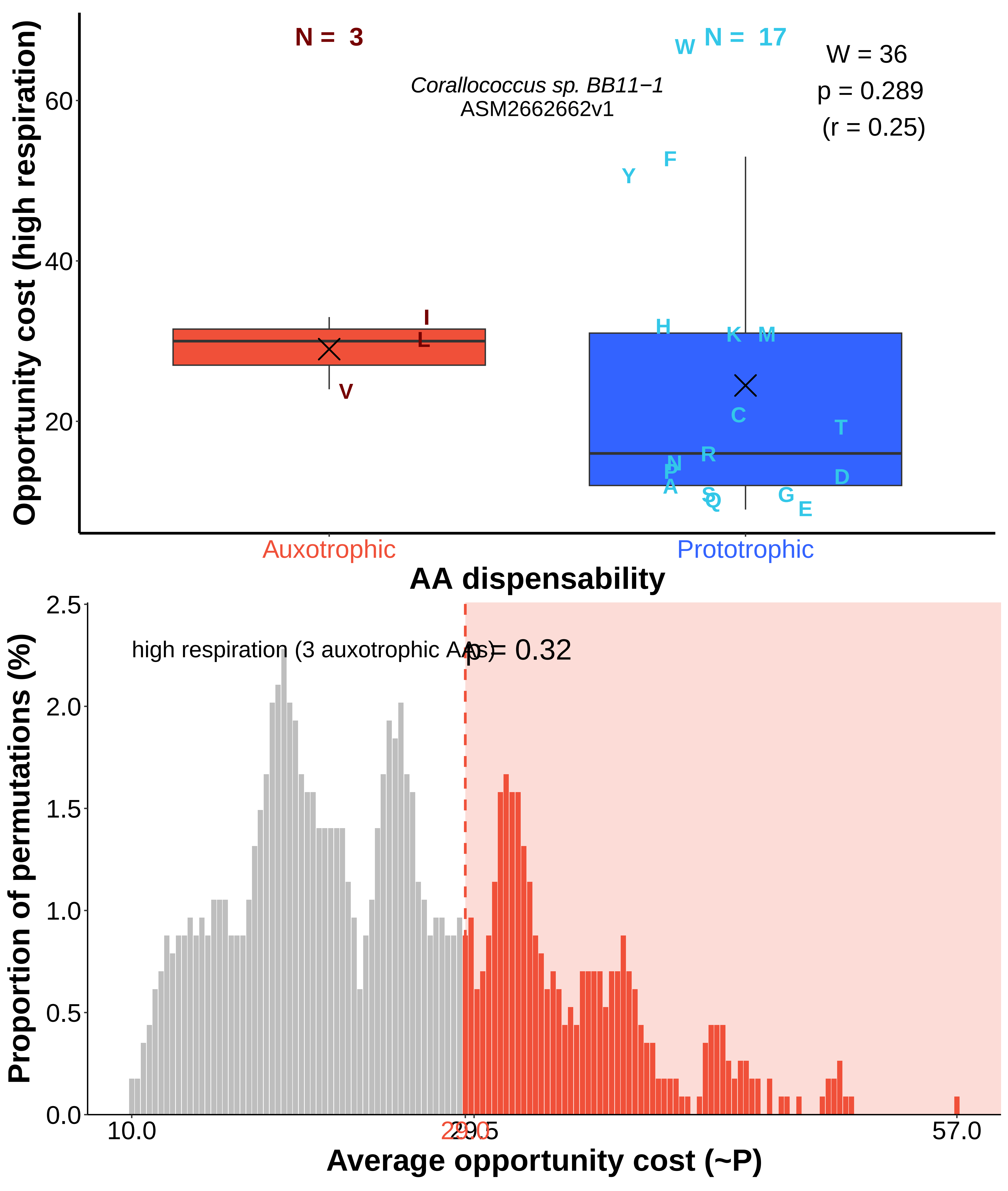
N = 3
W N = 17
Corallococcus sp. BB11−1
ASM2662662v1
W = 36
p = 0.289
(r = 0.25)
Opportunity cost (high respiration)
60
40
20
I
L
V
Y
F
H
K
M
C
T
R
N
P
A
S
Q
G
E
D
Auxotrophic
Prototrophic
AA dispensability
high respiration (3 auxotrophic AAs)
p = 0.32
Proportion of permutations (%)
2.5
2.0
1.5
1.0
0.5
0.0
10.0
29.0
29.5
57.0
Average opportunity cost (~P)

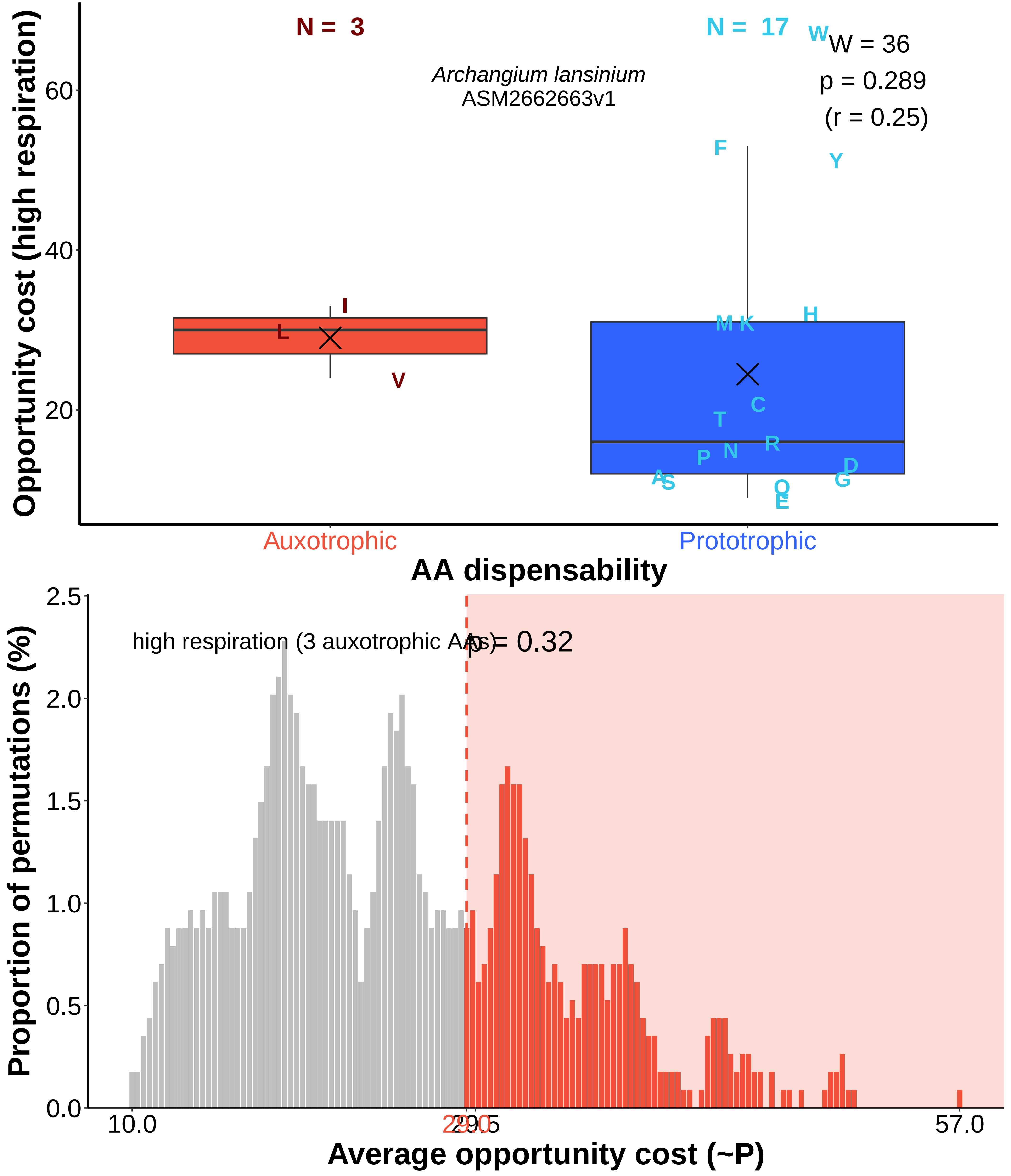
N = 3
N = 17
Archangium lansinium
ASM2662663v1
W = 36
p = 0.289
(r = 0.25)
Opportunity cost (high respiration)
Auxotrophic
Prototrophic
AA dispensability
high respiration (3 auxotrophic AAs)
p = 0.32
Proportion of permutations (%)
Average opportunity cost (~P)

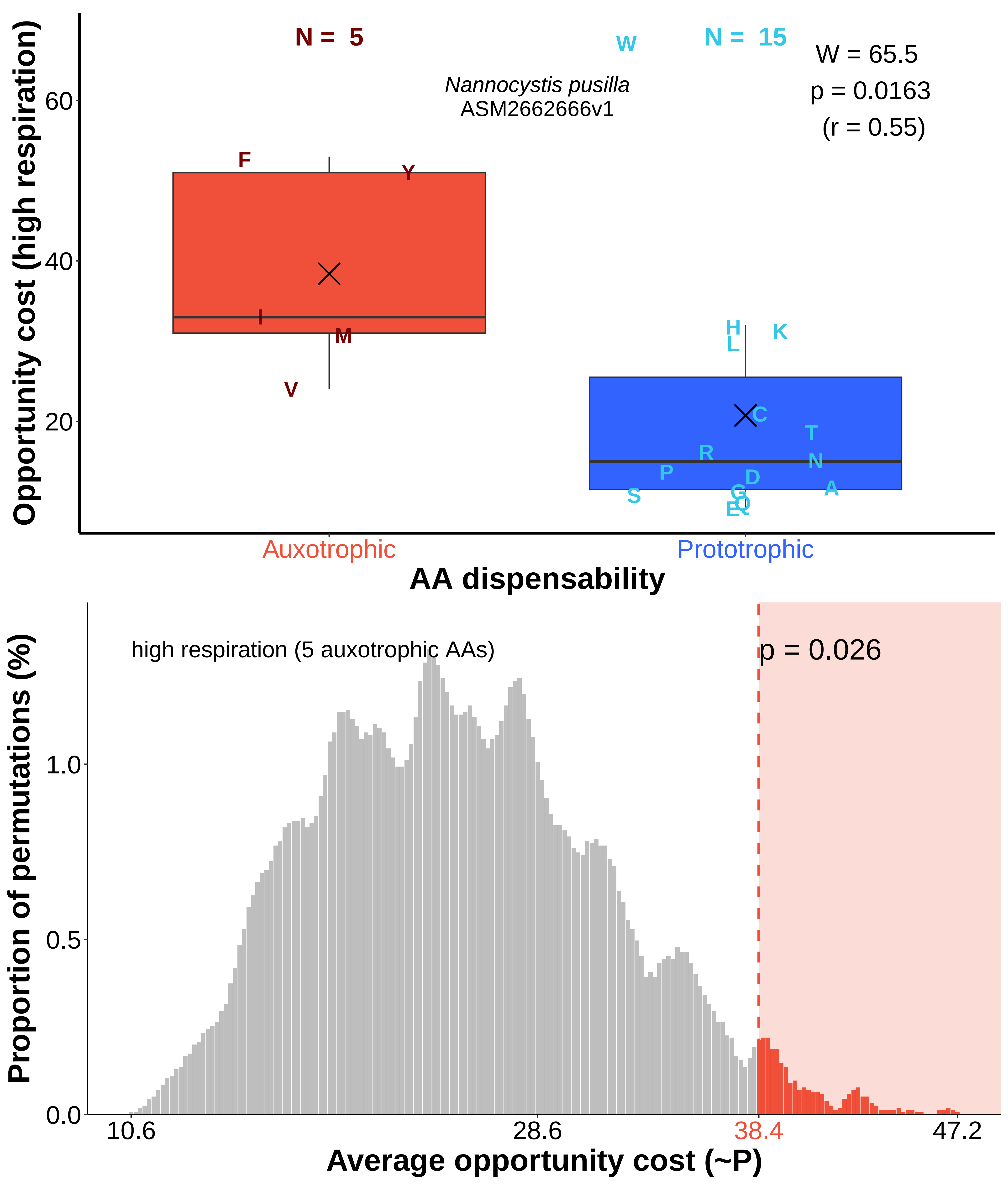
N = 5
N = 15
W
Nannocystis pusilla
ASM2662666v1
W = 65.5
p = 0.0163
(r = 0.55)
Opportunity cost (high respiration)
F
Y
I
M
V
H
L
K
C
T
R
N
P
D
A
S
G
E
Q
60
40
20
Auxotrophic
Prototrophic
AA dispensability
high respiration (5 auxotrophic AAs)
p = 0.026
Proportion of permutations (%)
1.0
0.5
0.0
10.6
28.6
38.4
47.2
Average opportunity cost (~P)

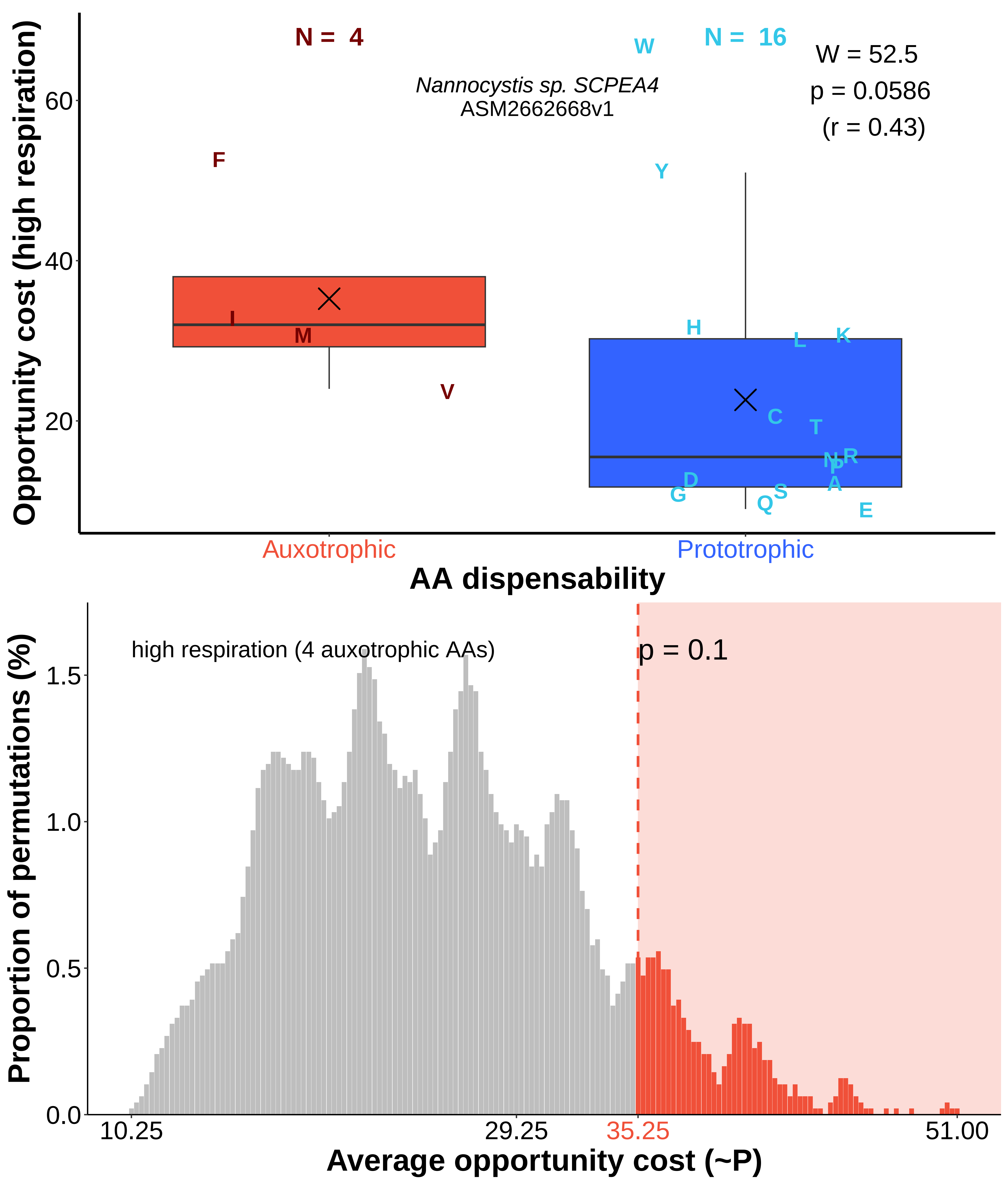

N = 4
N = 16
W = 52.5
p = 0.0586
(r = 0.43)
Nannocystis sp. SCPEA4
ASM2662668v1
Opportunity cost (high respiration)
Auxotrophic
Prototrophic
AA dispensability
high respiration (4 auxotrophic AAs)
p = 0.1
Proportion of permutations (%)
10.25
29.25
35.25
51.00
Average opportunity cost (~P)

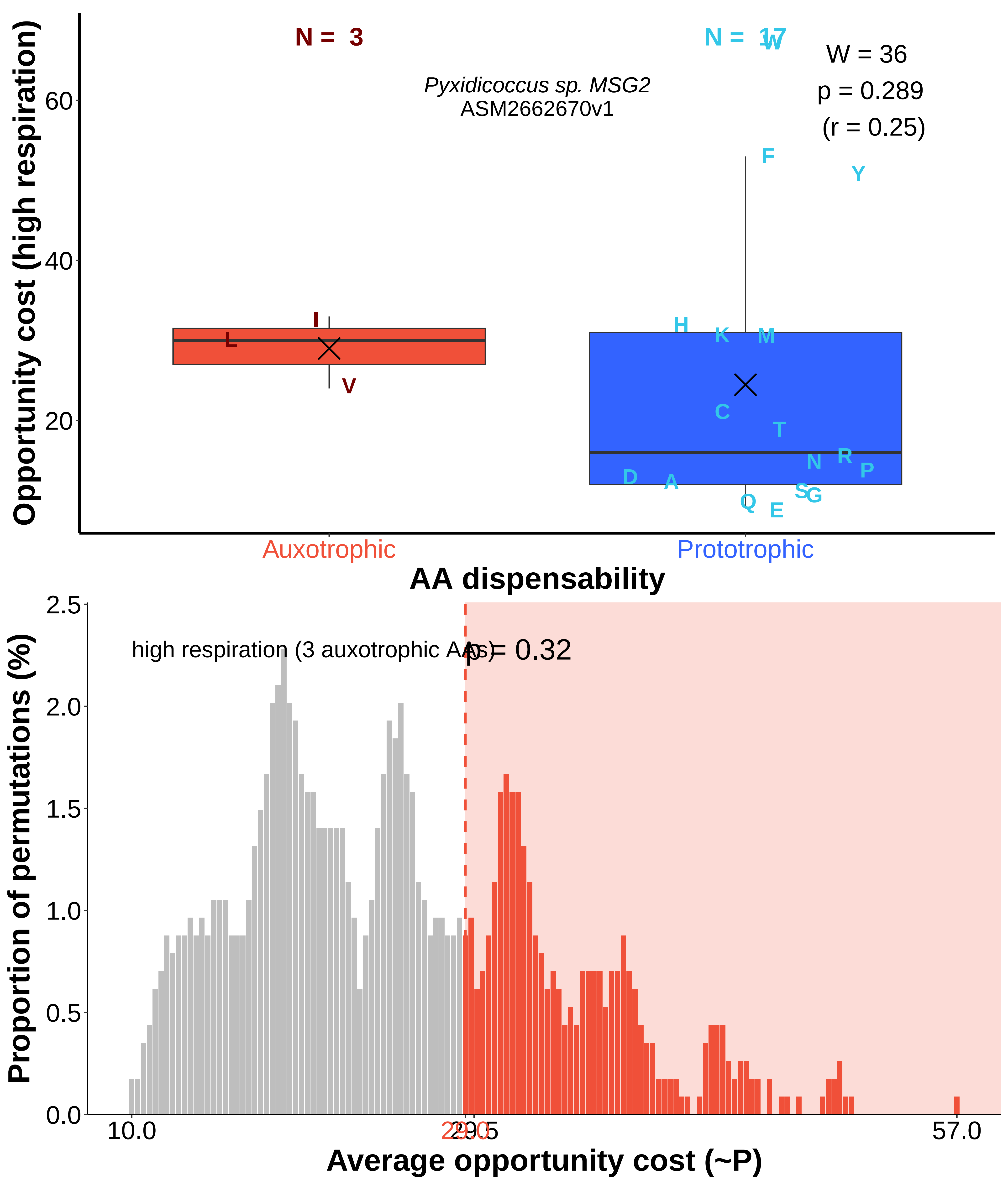

N = 3
N = 17
W = 36
p = 0.289
(r = 0.25)
Pyxidicoccus sp. MSG2
ASM2662670v1
Opportunity cost (high respiration)
Auxotrophic
Prototrophic
AA dispensability
high respiration (3 auxotrophic AAs)
p = 0.32
Proportion of permutations (%)
10.0
29.0
29.5
57.0
Average opportunity cost (~P)

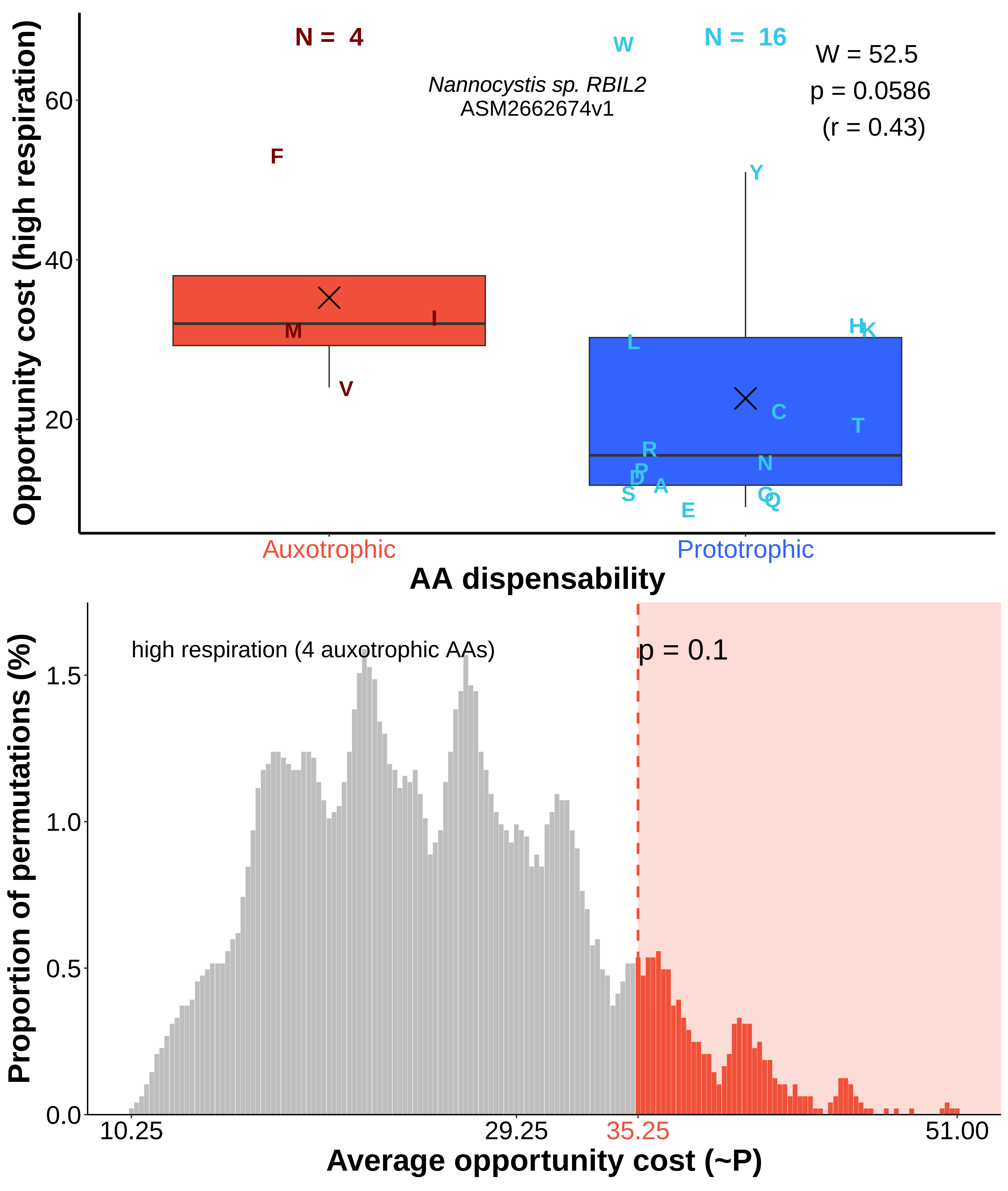

N = 4
N = 16
W = 52.5
p = 0.0586
(r = 0.43)
Nannocystis sp. RBIL2
ASM2662674v1
Opportunity cost (high respiration)
60
40
20
F
M
I
V
W
Y
H
K
L
C
T
R
N
P
D
A
S
G
Q
E
Auxotrophic
Prototrophic
AA dispensability
high respiration (4 auxotrophic AAs)
p = 0.1
Proportion of permutations (%)
1.5
1.0
0.5
0.0
10.25
29.25
35.25
51.00
Average opportunity cost (~P)

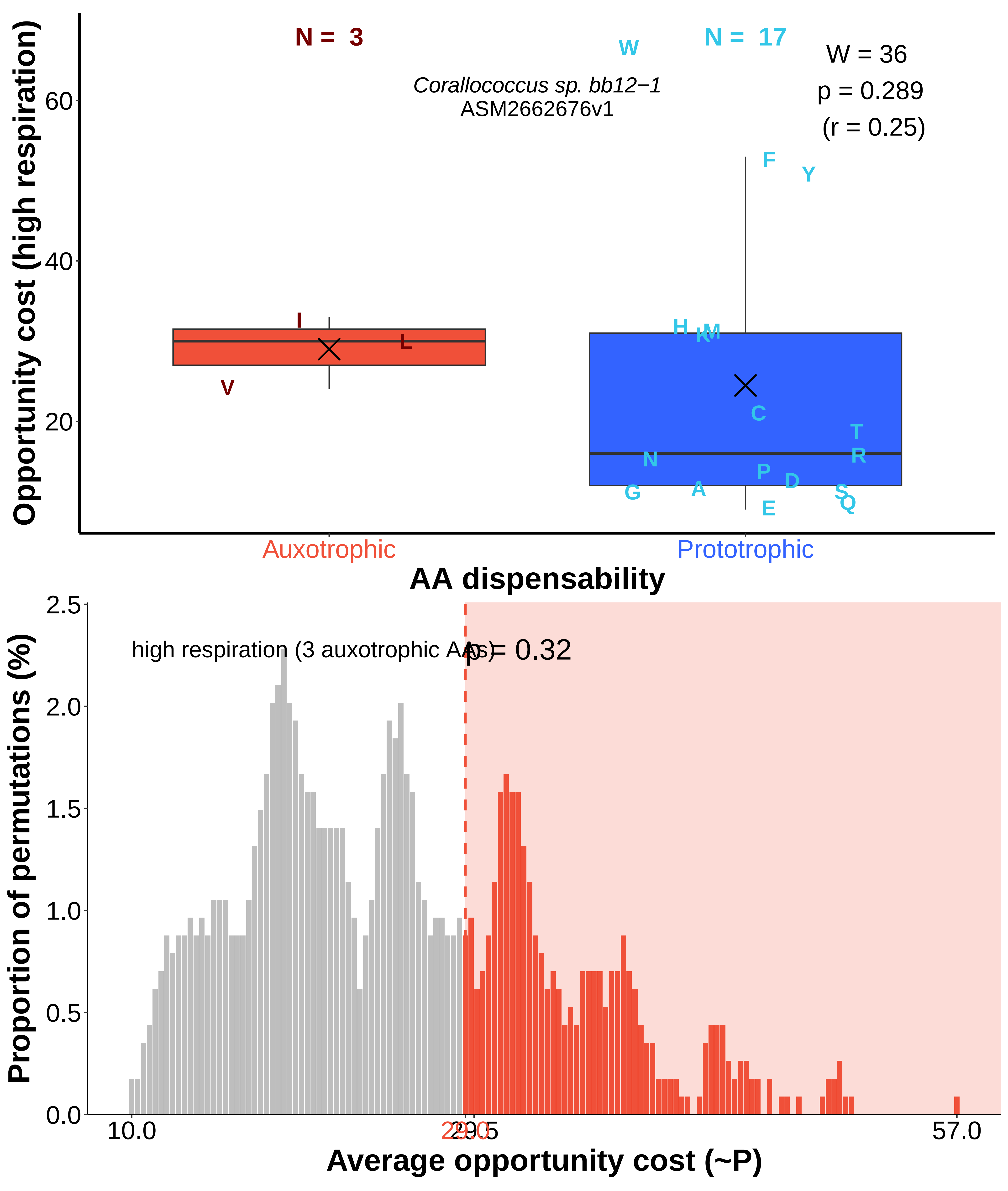

N = 3
N = 17
W = 36
p = 0.289
(r = 0.25)
Corallococcus sp. bb12−1
ASM2662676v1
Opportunity cost (high respiration)
60
40
20
I
L
V
W
F
Y
H
K
M
C
T
R
N
P
D
G
A
S
Q
E
Auxotrophic
Prototrophic
AA dispensability
high respiration (3 auxotrophic AAs)
p = 0.32
Proportion of permutations (%)
2.5
2.0
1.5
1.0
0.5
0.0
10.0
29.0
29.5
57.0
Average opportunity cost (~P)

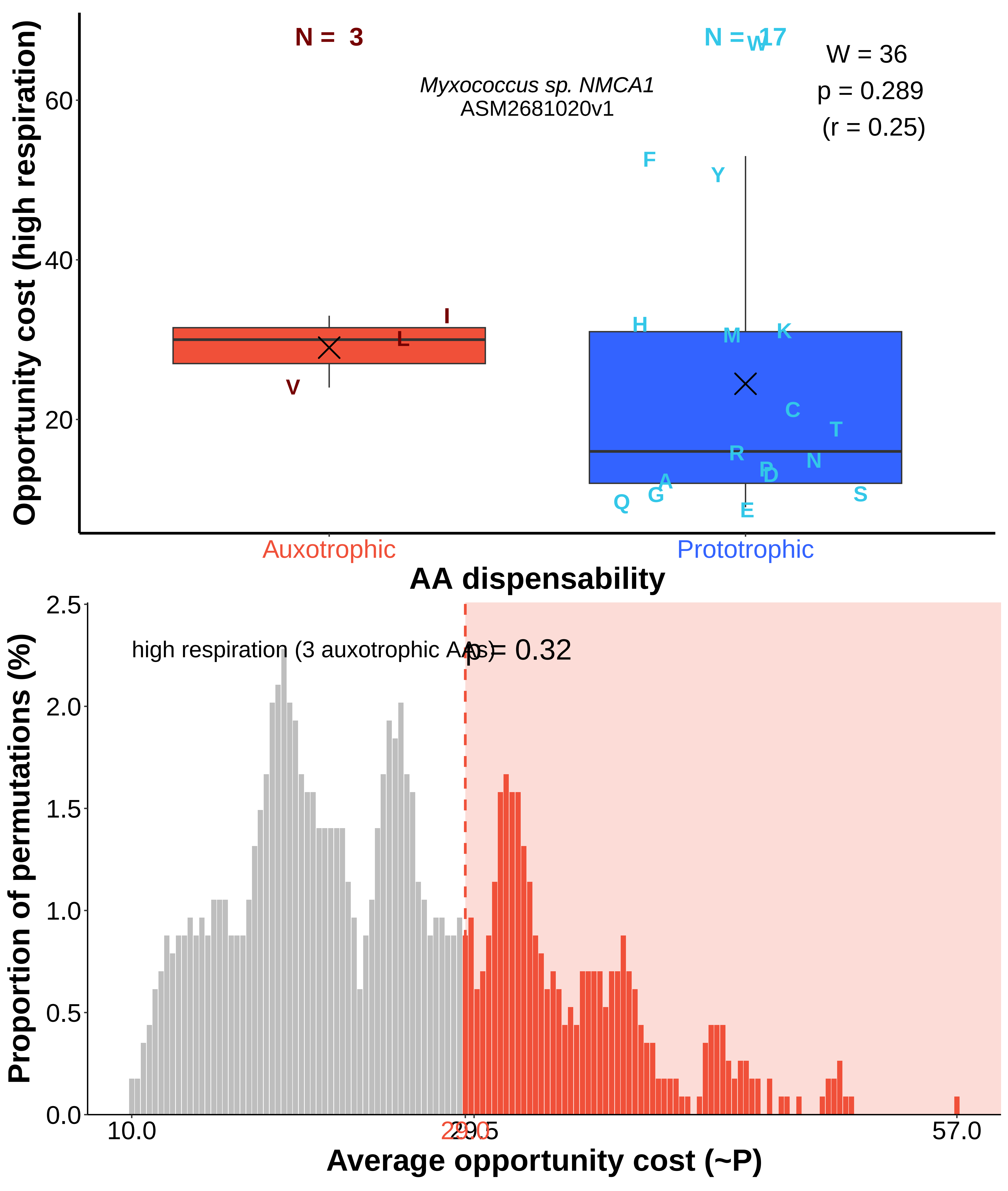
N = 3
N = 17
W
Myxococcus sp. NMCA1
ASM2681020v1
W = 36
p = 0.289
(r = 0.25)
Opportunity cost (high respiration)
60
40
20
F
Y
I
L
V
H
M
K
C
T
R
N
P
D
A
G
Q
S
E
Auxotrophic
Prototrophic
AA dispensability
Proportion of permutations (%)
2.5
2.0
1.5
1.0
0.5
0.0
high respiration (3 auxotrophic AAs)
p = 0.32
10.0
29.0
29.5
57.0
Average opportunity cost (~P)

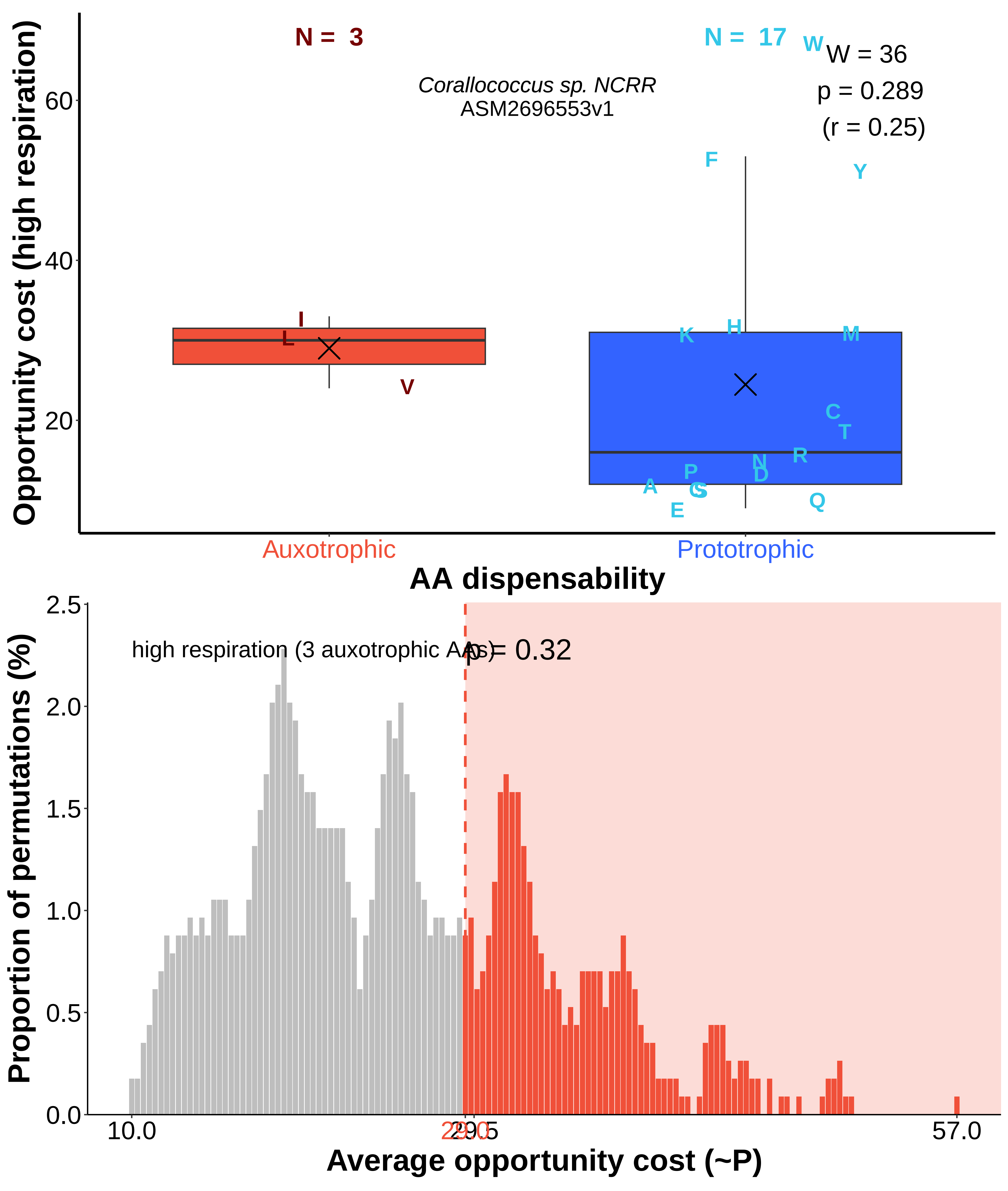
N = 3
N = 17
Corallococcus sp. NCRR
ASM2696553v1
W = 36
p = 0.289
(r = 0.25)
Opportunity cost (high respiration)
60
40
20
F
Y
I
L
V
K
H
M
C
T
R
N
D
P
A
G
S
E
Q
W
Auxotrophic
Prototrophic
AA dispensability
Proportion of permutations (%)
2.5
2.0
1.5
1.0
0.5
0.0
high respiration (3 auxotrophic AAs)
p = 0.32
10.0
29.0
57.0
Average opportunity cost (~P)

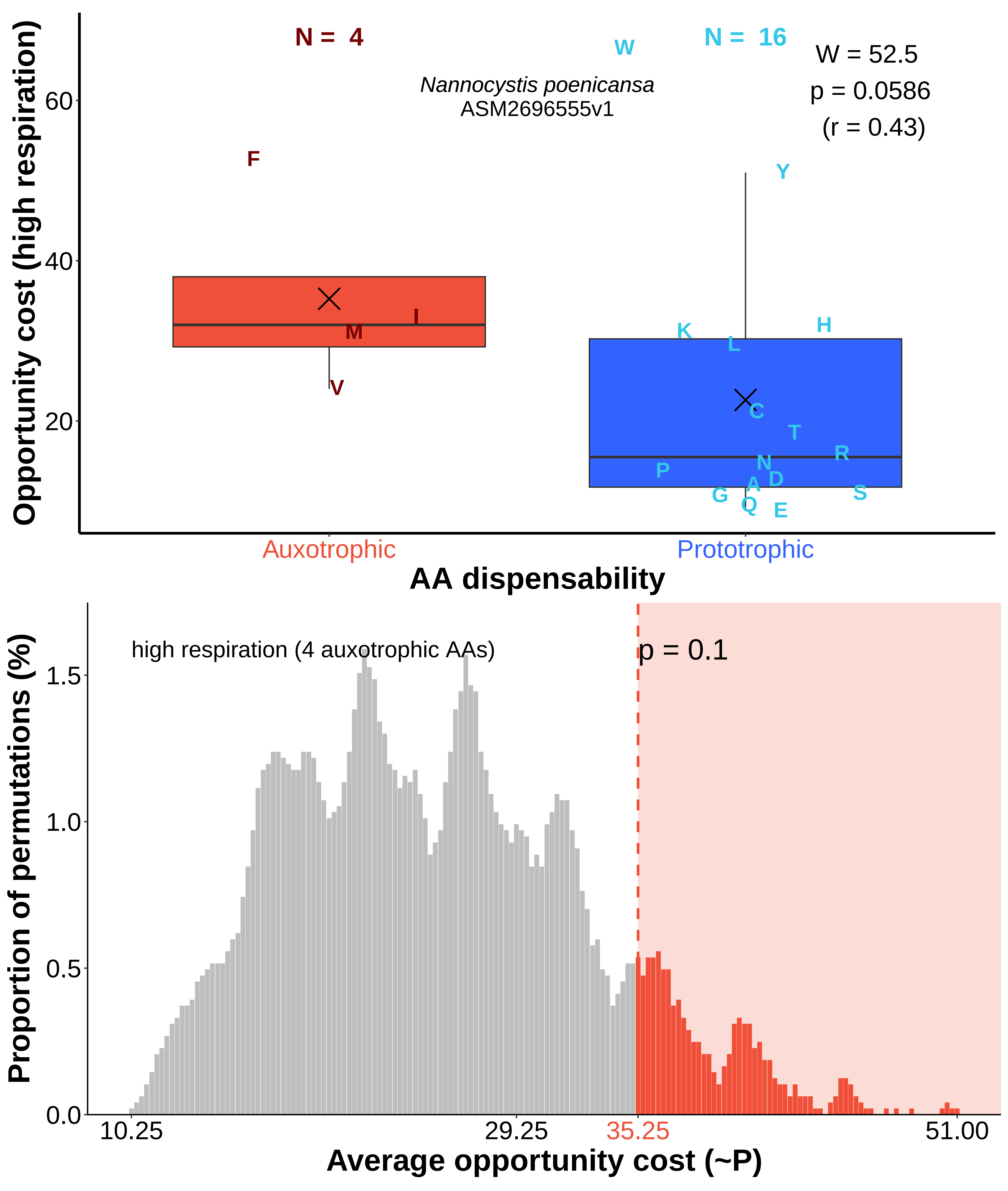
N = 4
N = 16
W = 52.5
p = 0.0586
(r = 0.43)
Nannocystis poenicansa
ASM2696555v1
Opportunity cost (high respiration)
60
40
20
F
I
M
V
W
Y
K
L
H
C
T
R
N
P
A
D
G
Q
E
S
Auxotrophic
Prototrophic
AA dispensability
high respiration (4 auxotrophic AAs)
p = 0.1
Proportion of permutations (%)
1.5
1.0
0.5
0.0
10.25
29.25
35.25
51.00
Average opportunity cost (~P)

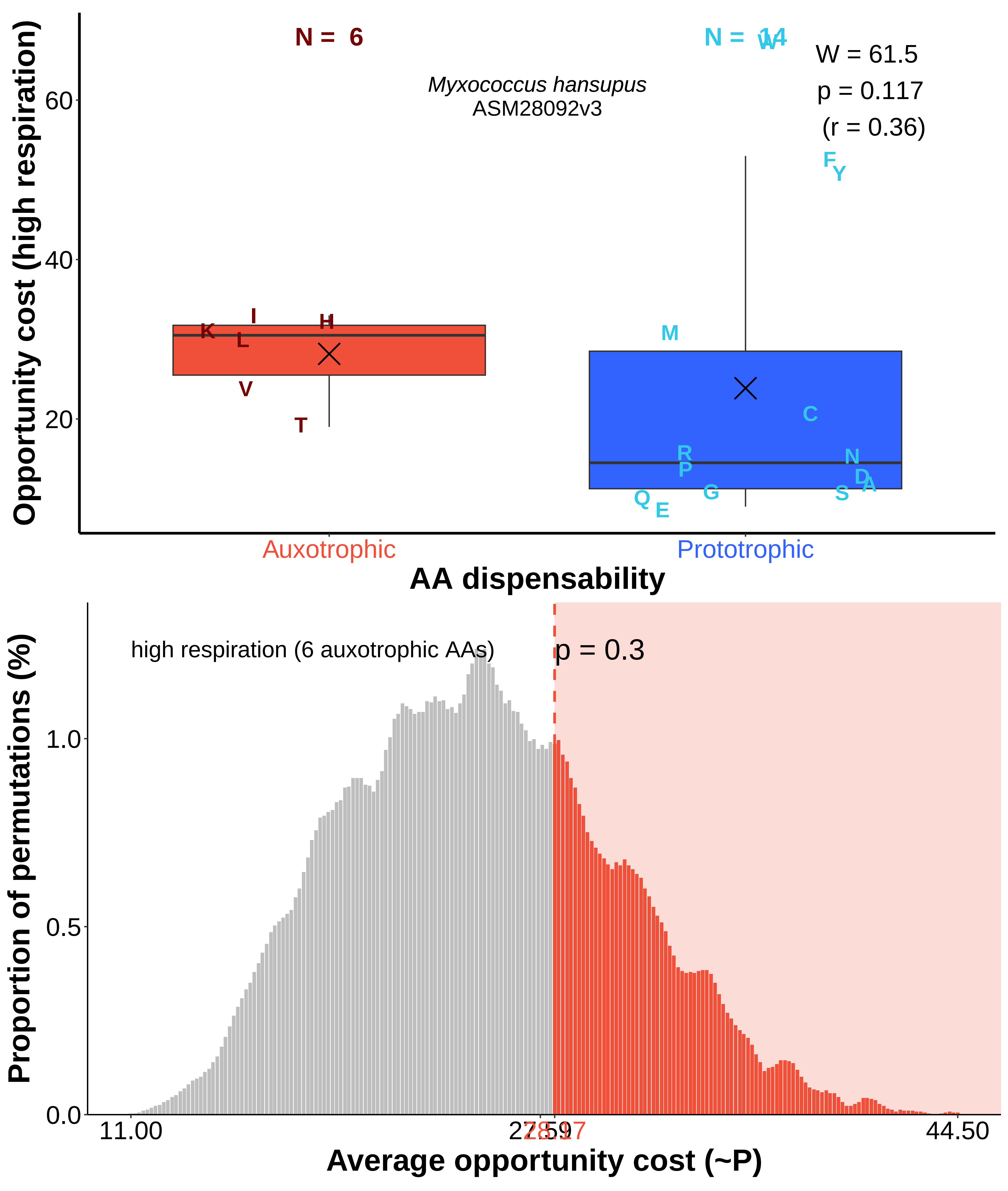

N = 6
N = 14
W
W = 61.5
p = 0.117
(r = 0.36)
Myxococcus hansupus
ASM28092v3
F
Y
I
H
K
L
M
V
T
C
R
P
N
D
A
G
S
Q
E
60
40
20
Opportunity cost (high respiration)
Auxotrophic
Prototrophic
AA dispensability
high respiration (6 auxotrophic AAs)
p = 0.3
1.0
0.5
0.0
Proportion of permutations (%)
11.00
27.91
28.17
44.50
Average opportunity cost (~P)

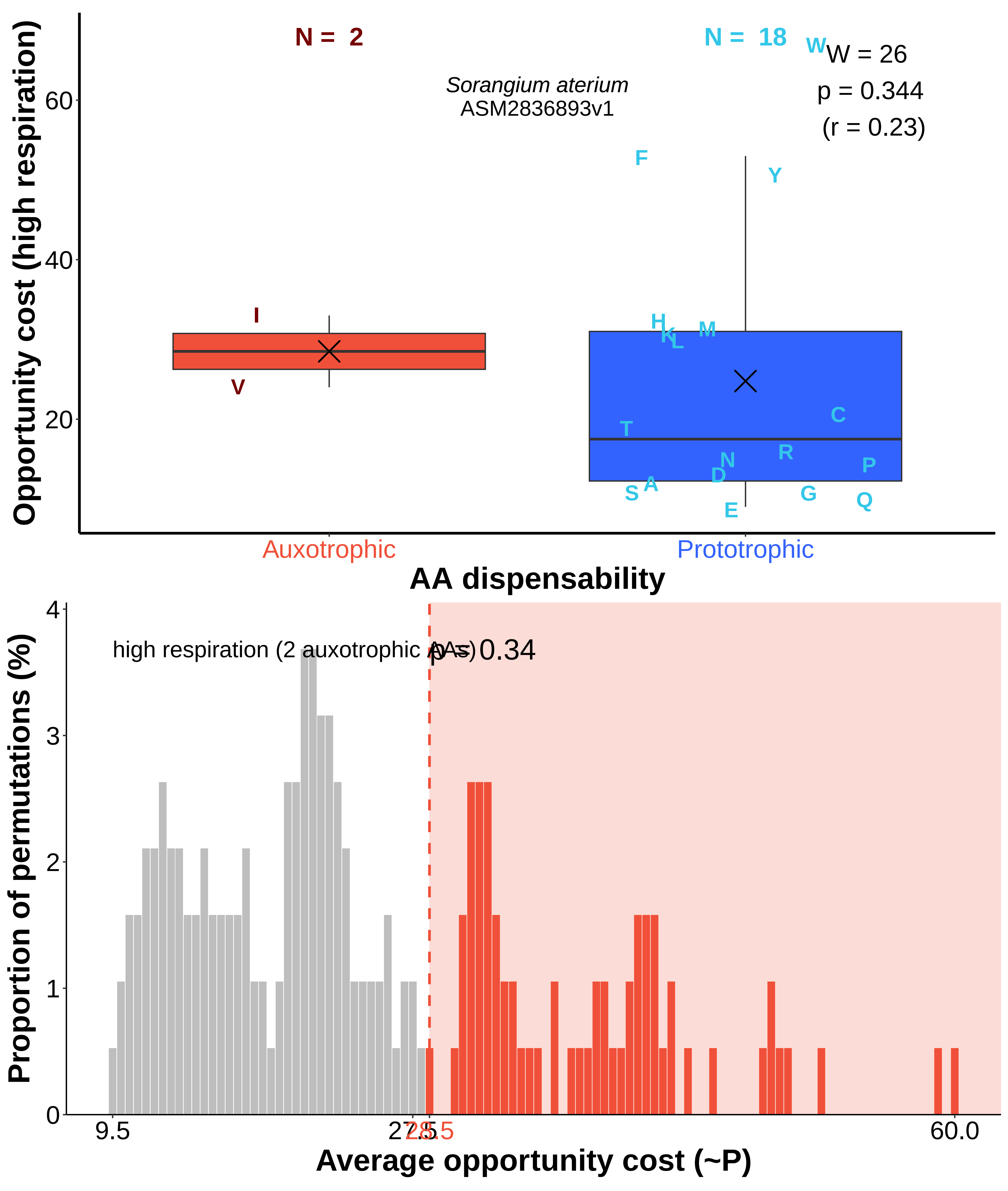
N = 2
N = 18
W = 26
p = 0.344
(r = 0.23)
Sorangium aterium
ASM2836893v1
Opportunity cost (high respiration)
Auxotrophic
Prototrophic
AA dispensability
high respiration (2 auxotrophic AAs)
p = 0.34
Proportion of permutations (%)
Average opportunity cost (~P)
9.5
27.5
28.5
60.0

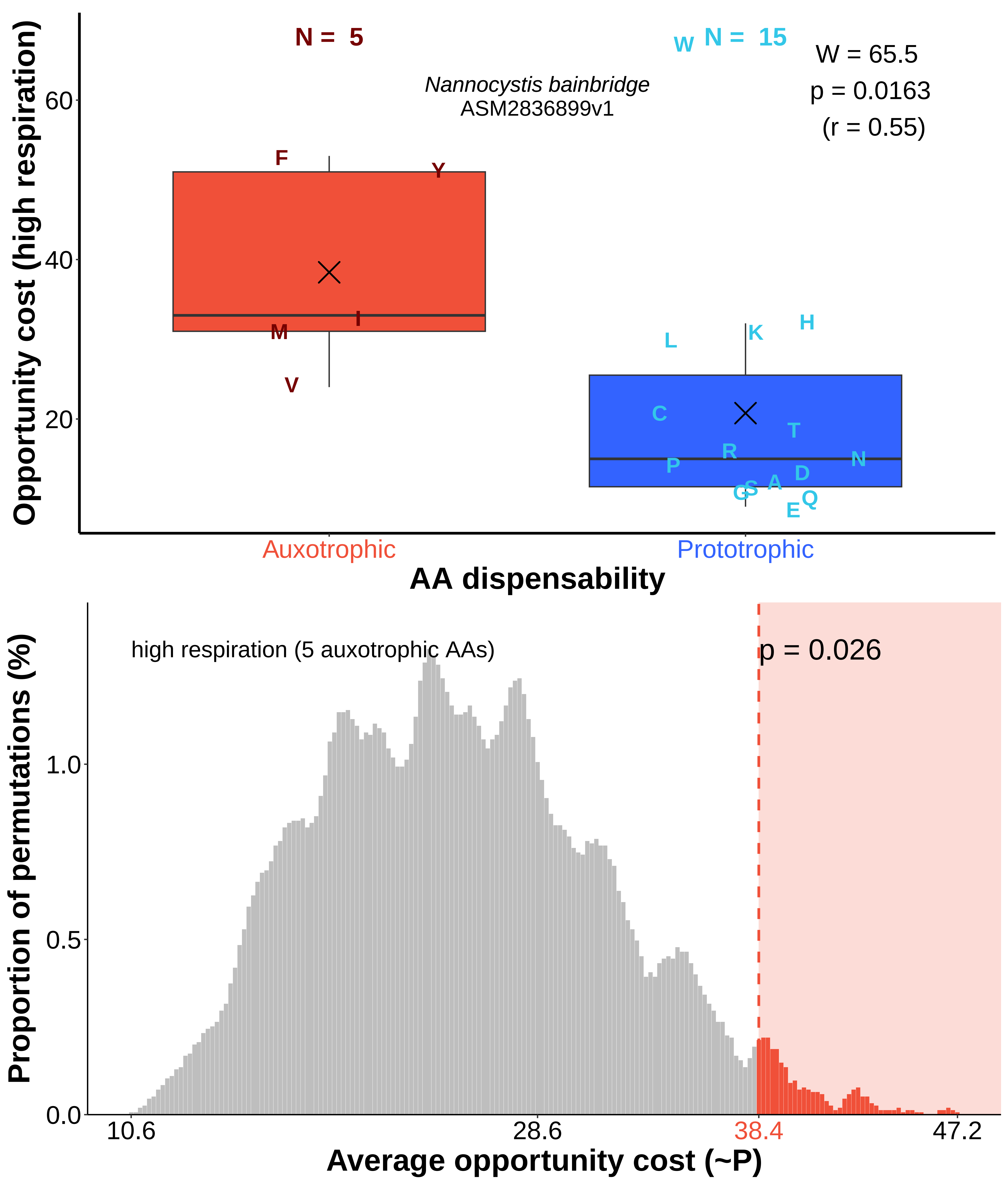
N = 5
N = 15
W = 65.5
p = 0.0163
(r = 0.55)
Nannocystis bainbridge
ASM2836899v1
Opportunity cost (high respiration)
Auxotrophic
Prototrophic
AA dispensability
high respiration (5 auxotrophic AAs)
p = 0.026
Proportion of permutations (%)
Average opportunity cost (~P)
10.6
28.6
38.4
47.2

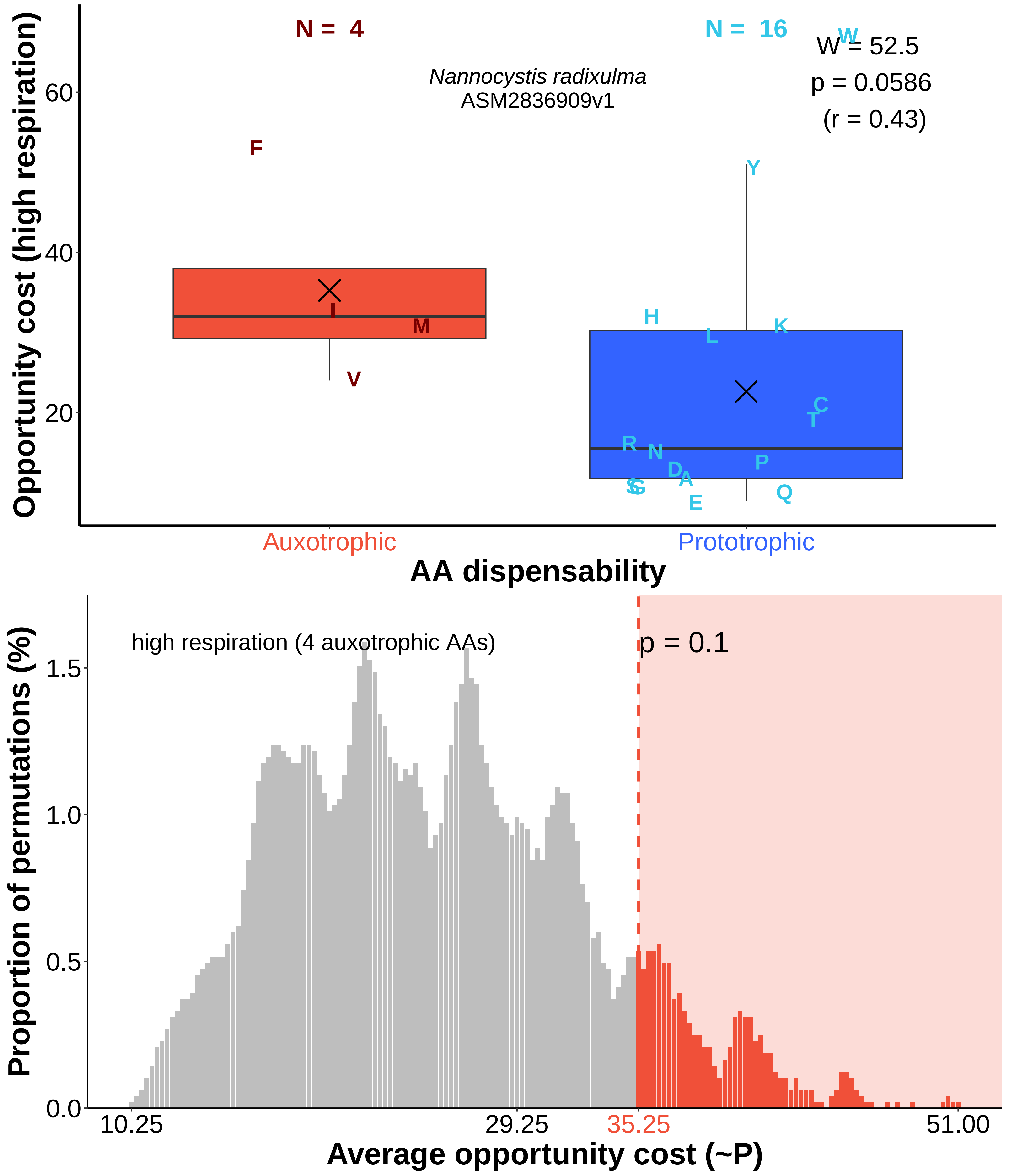

N = 4
N = 16
W = 52.5
p = 0.0586
(r = 0.43)
Nannocystis radixulma
ASM2836909v1
Opportunity cost (high respiration)
60
40
20
F
Y
I
M
V
H
L
K
C
T
R
N
P
D
A
S
G
E
Q
W
Auxotrophic
Prototrophic
AA dispensability
high respiration (4 auxotrophic AAs)
p = 0.1
Proportion of permutations (%)
1.5
1.0
0.5
0.0
10.25
29.25
35.25
51.00
Average opportunity cost (~P)

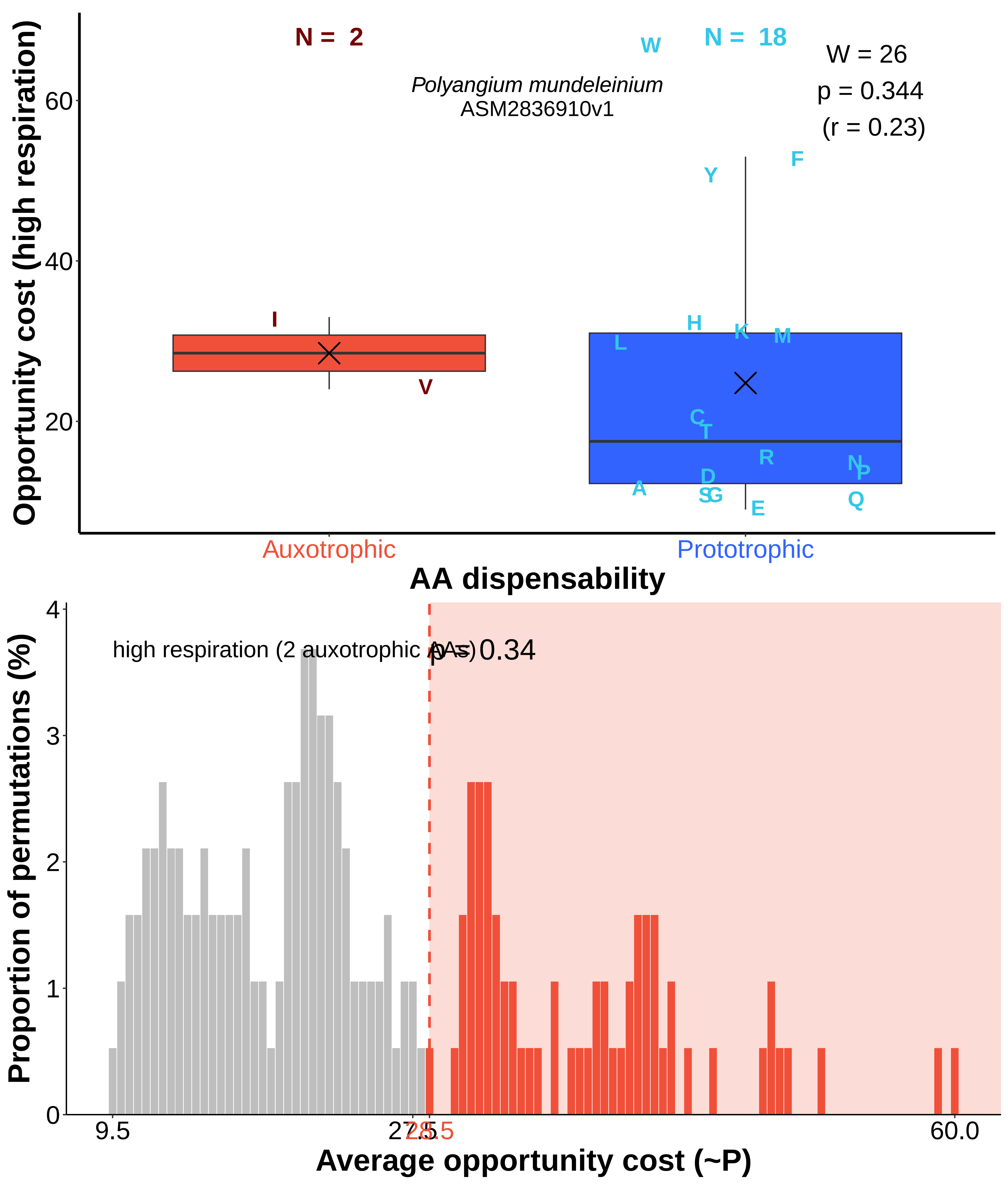

N = 2
N = 18
W = 26
p = 0.344
(r = 0.23)
Polyangium mundeleinium
ASM2836910v1
Opportunity cost (high respiration)
Auxotrophic
Prototrophic
AA dispensability
high respiration (2 auxotrophic AAs)
p = 0.34
Proportion of permutations (%)
Average opportunity cost (~P)
9.5
27.5
28.5
60.0

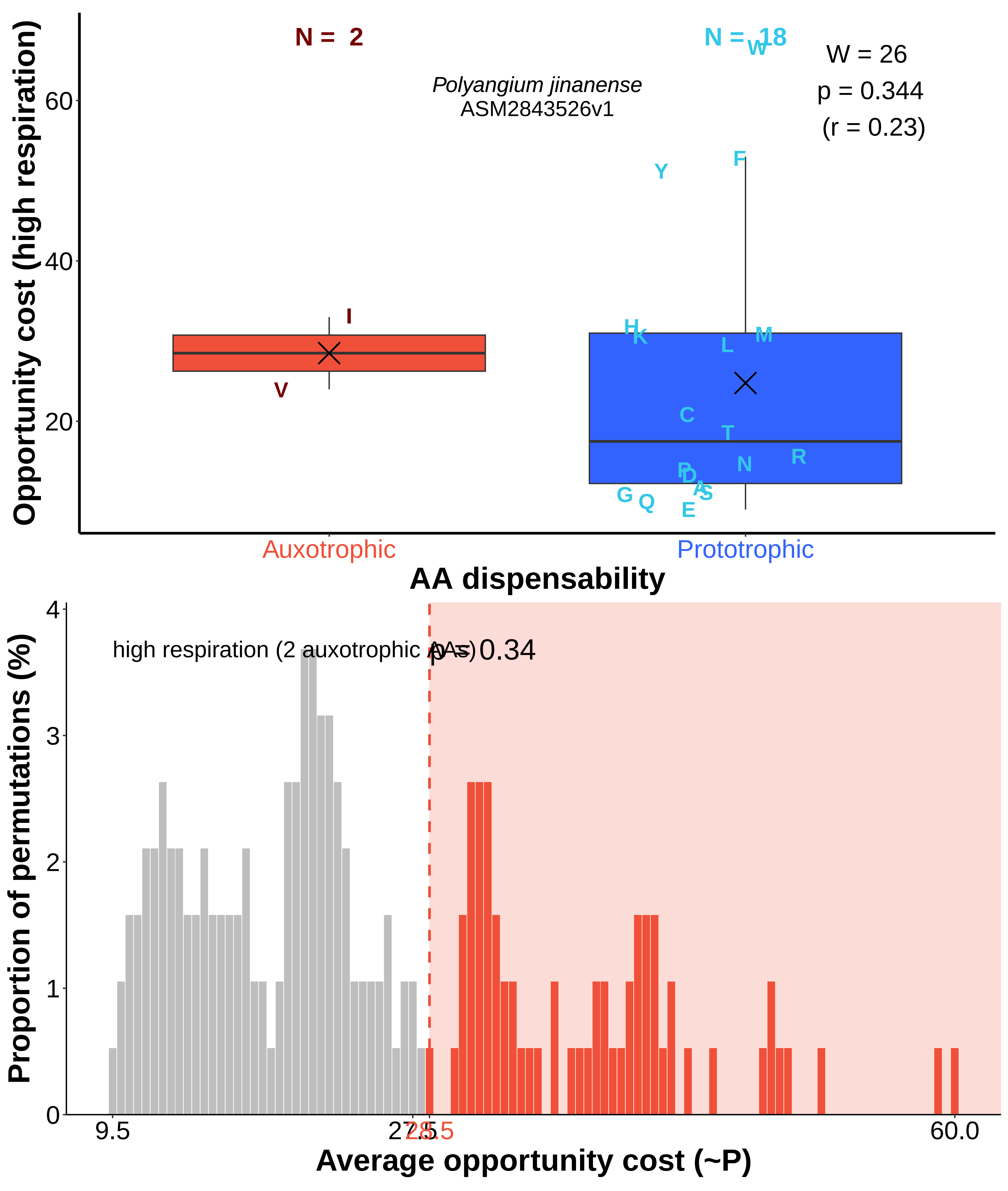

N = 2
N = 18
W = 26
p = 0.344
(r = 0.23)
Polyangium jinanense
ASM2843526v1
Opportunity cost (high respiration)
60
40
20
I
V
W
Y
F
H
K
M
L
C
T
R
N
P
D
A
S
G
Q
E
Auxotrophic
Prototrophic
AA dispensability
Proportion of permutations (%)
4
3
2
1
0
high respiration (2 auxotrophic AAs)
p = 0.34
9.5
27.5
28.5
60.0
Average opportunity cost (~P)

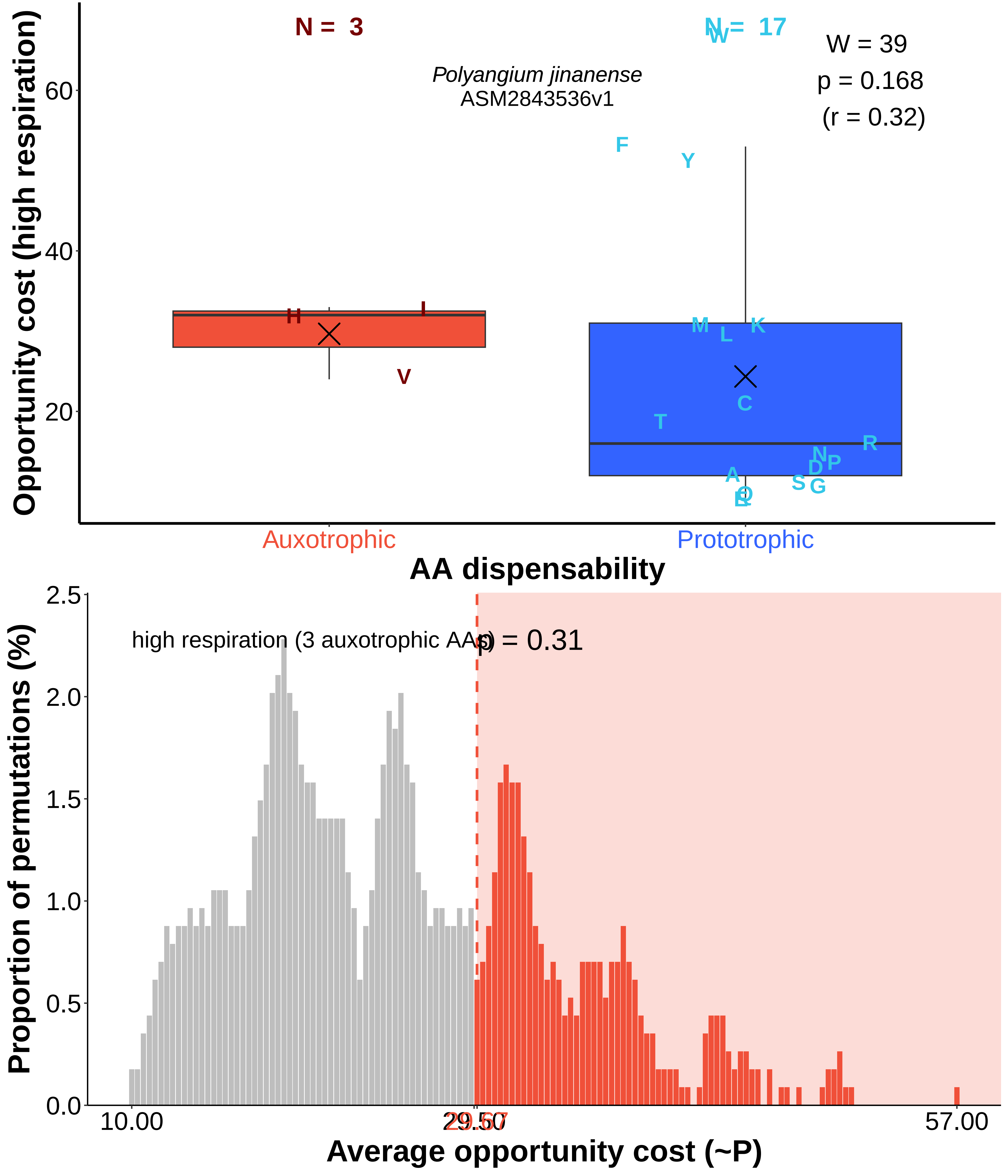

N = 3
N = 17
W
W = 39
p = 0.168
(r = 0.32)
Polyangium jinanense
ASM2843536v1
Opportunity cost (high respiration)
60
40
20
F
Y
H
I
V
M
L
K
C
T
R
N
P
D
A
S
G
Q
E
Auxotrophic
Prototrophic
AA dispensability
Proportion of permutations (%)
2.5
2.0
1.5
1.0
0.5
0.0
high respiration (3 auxotrophic AAs)
p = 0.31
10.00
29.57
57.00
Average opportunity cost (~P)

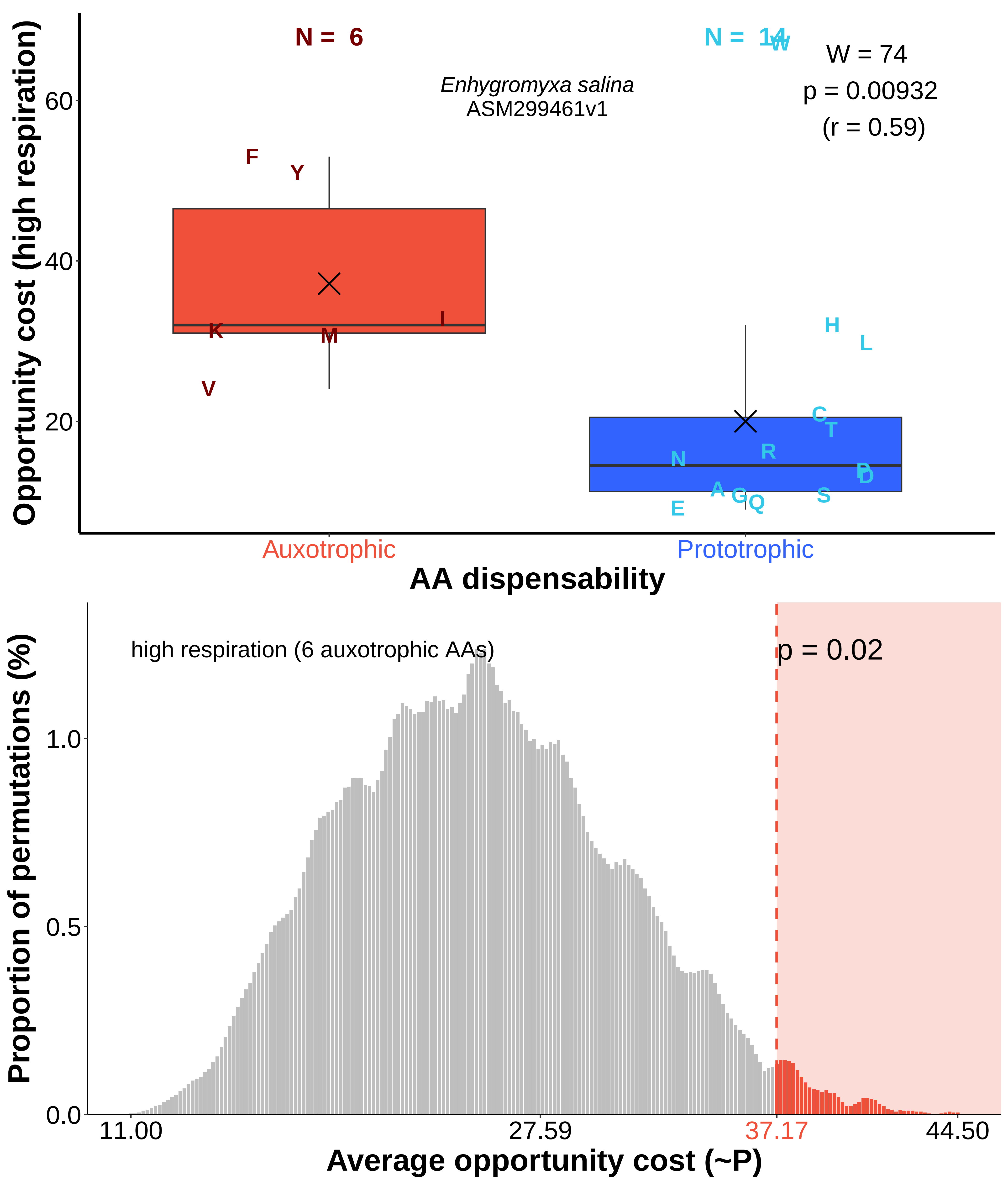

N = 6
N = 14
Enhygromyxa salina
ASM299461v1
W = 74
p = 0.00932
(r = 0.59)
Opportunity cost (high respiration)
60
40
20
F
Y
I
K
M
V
W
H
L
C
T
R
N
P
D
A
G
Q
S
E
Auxotrophic
Prototrophic
AA dispensability
high respiration (6 auxotrophic AAs)
p = 0.02
Proportion of permutations (%)
1.0
0.5
0.0
11.00
27.59
37.17
44.50
Average opportunity cost (~P)

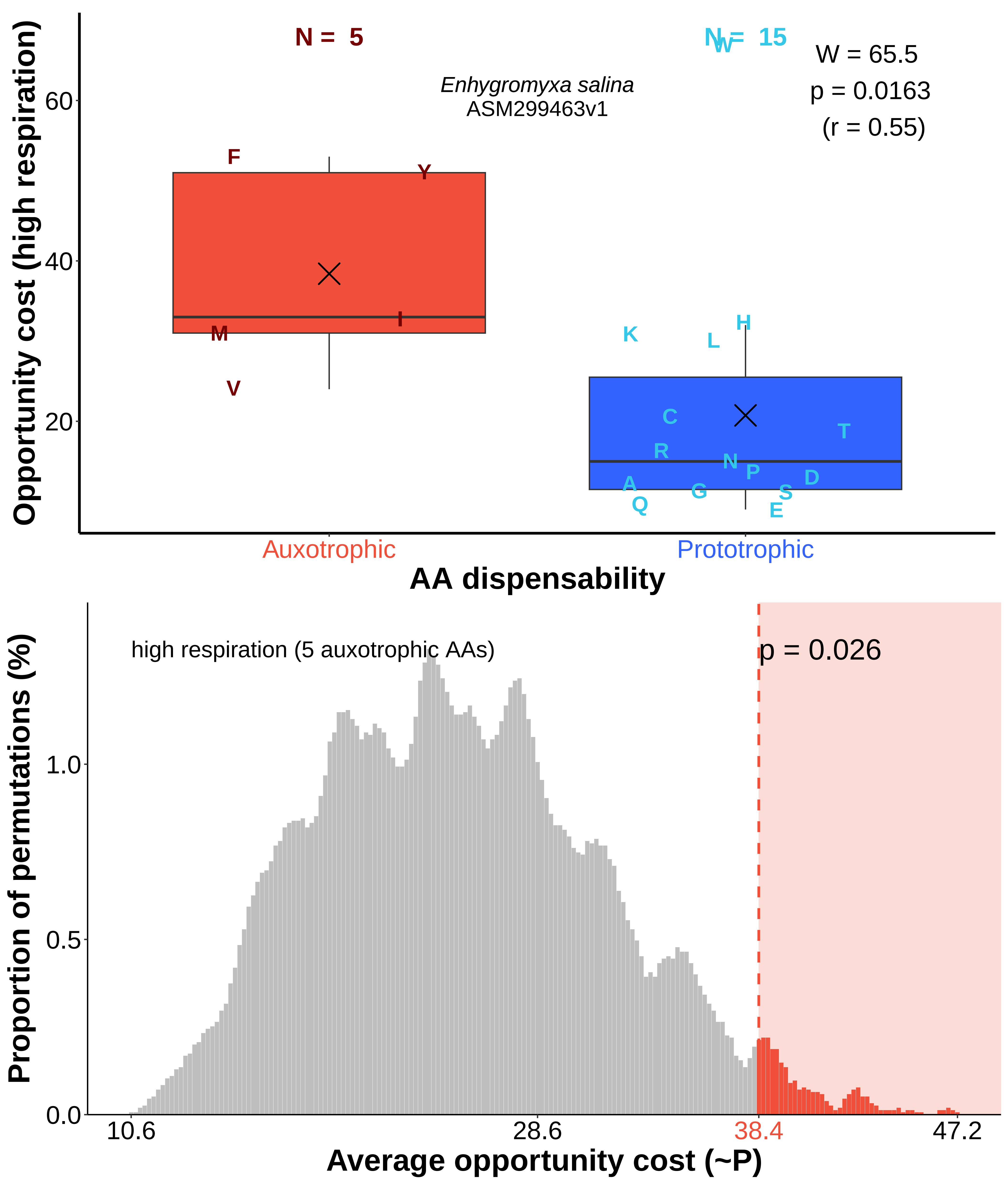
N = 5
N = 15
W
W = 65.5
p = 0.0163
(r = 0.55)
Enhygromyxa salina
ASM299463v1
F
Y
I
M
V
K
L
H
C
T
R
N
P
D
A
G
S
Q
E
60
40
20
Opportunity cost (high respiration)
Auxotrophic
Prototrophic
AA dispensability
high respiration (5 auxotrophic AAs)
p = 0.026
1.0
0.5
0.0
Proportion of permutations (%)
10.6
28.6
38.4
47.2
Average opportunity cost (~P)

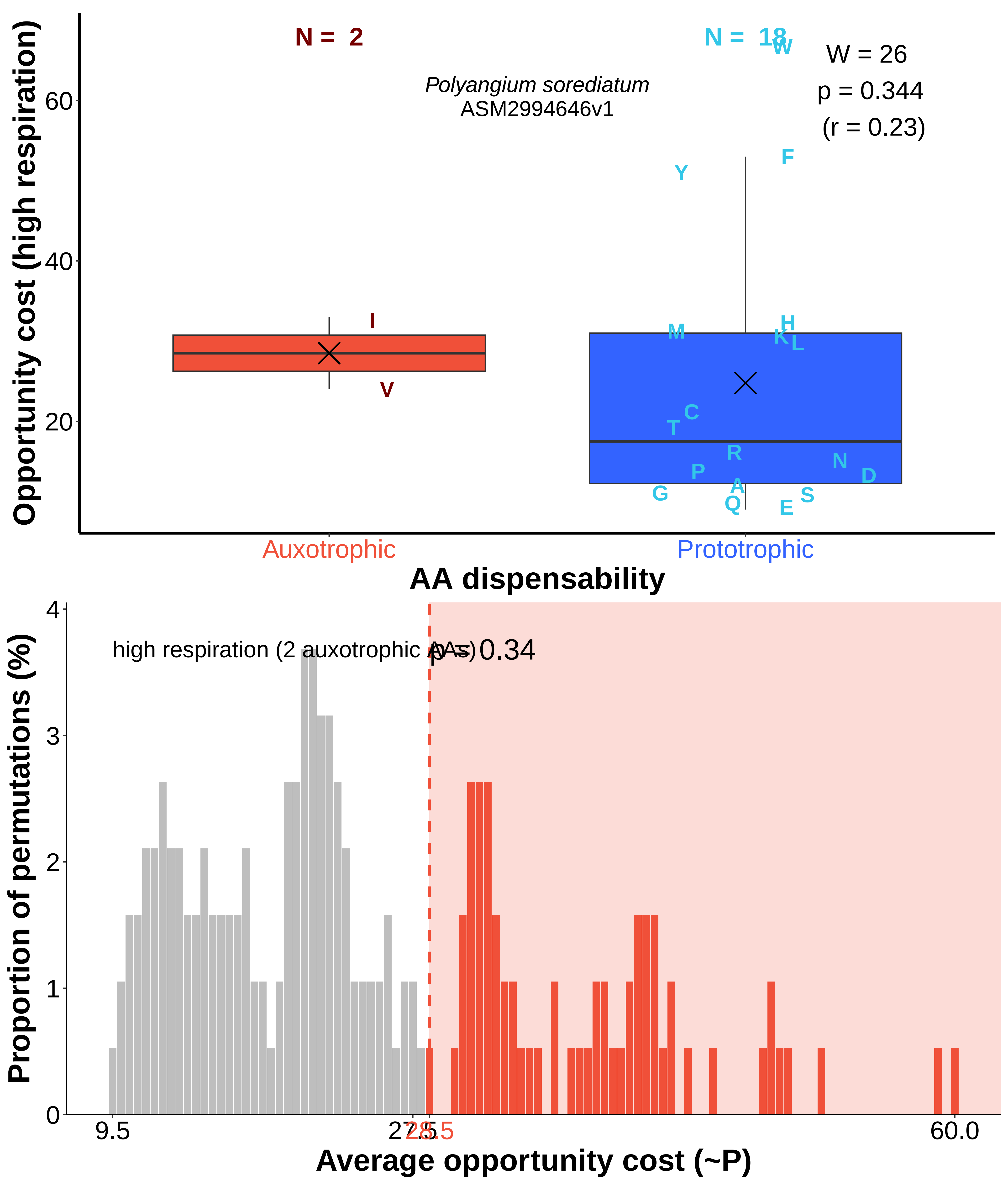

N = 2
N = 18
W = 26
p = 0.344
(r = 0.23)
Polyangium sorediatum
ASM2994646v1
Opportunity cost (high respiration)
60
40
20
I
V
W
F
Y
M
H
K
L
C
T
R
N
P
D
A
G
S
Q
E
Auxotrophic
Prototrophic
AA dispensability
high respiration (2 auxotrophic AAs)
p = 0.34
Proportion of permutations (%)
4
3
2
1
0
9.5
27.5
28.5
60.0
Average opportunity cost (~P)

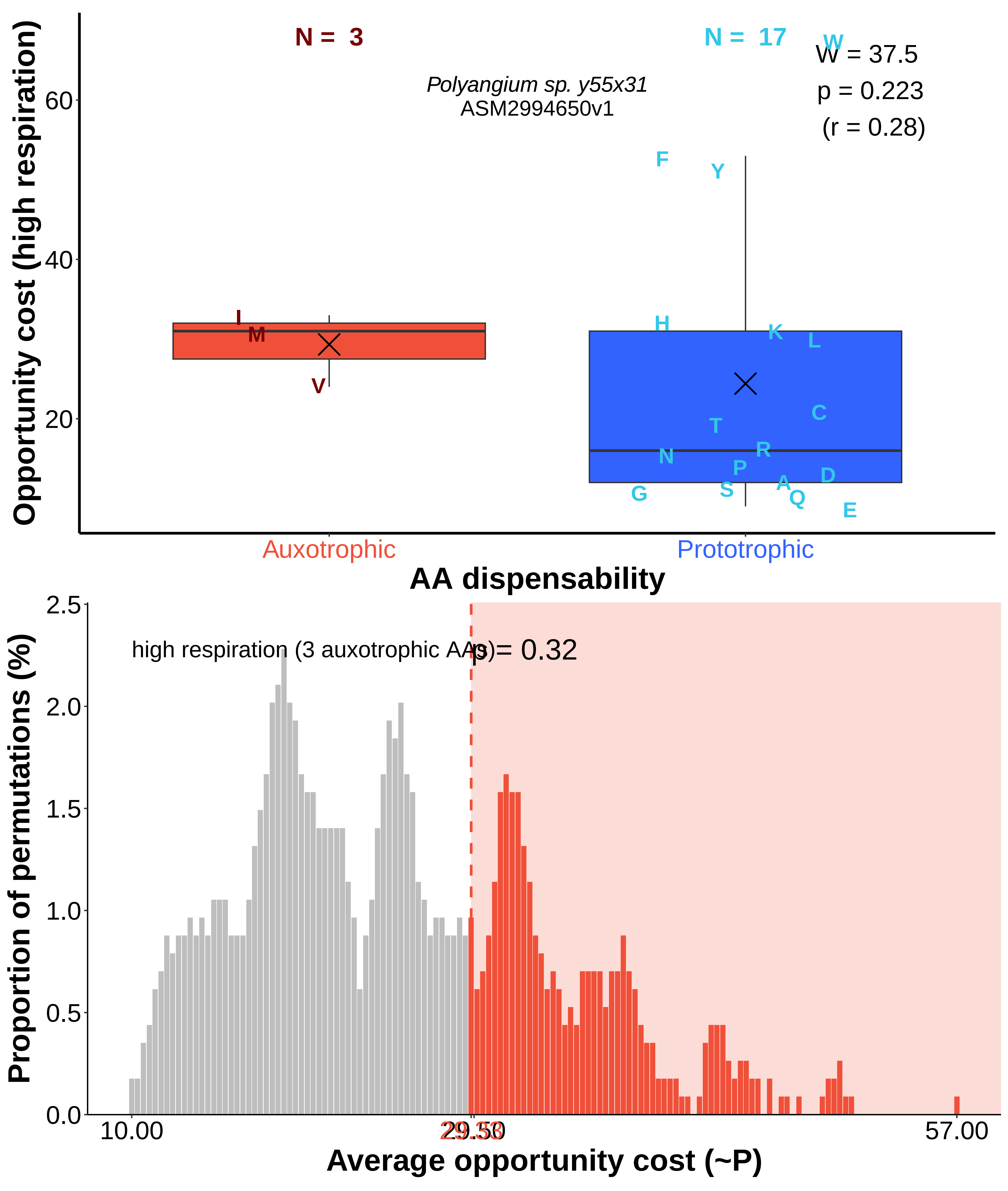

N = 3
N = 17
W = 37.5
p = 0.223
(r = 0.28)
Polyangium sp. y55x31
ASM2994650v1
Opportunity cost (high respiration)
60
40
20
I
M
V
F
Y
W
H
K
L
C
T
R
N
P
D
G
S
A
Q
E
Auxotrophic
Prototrophic
AA dispensability
Proportion of permutations (%)
2.5
2.0
1.5
1.0
0.5
0.0
high respiration (3 auxotrophic AAs)
p = 0.32
10.00
29.33
29.50
57.00
Average opportunity cost (~P)

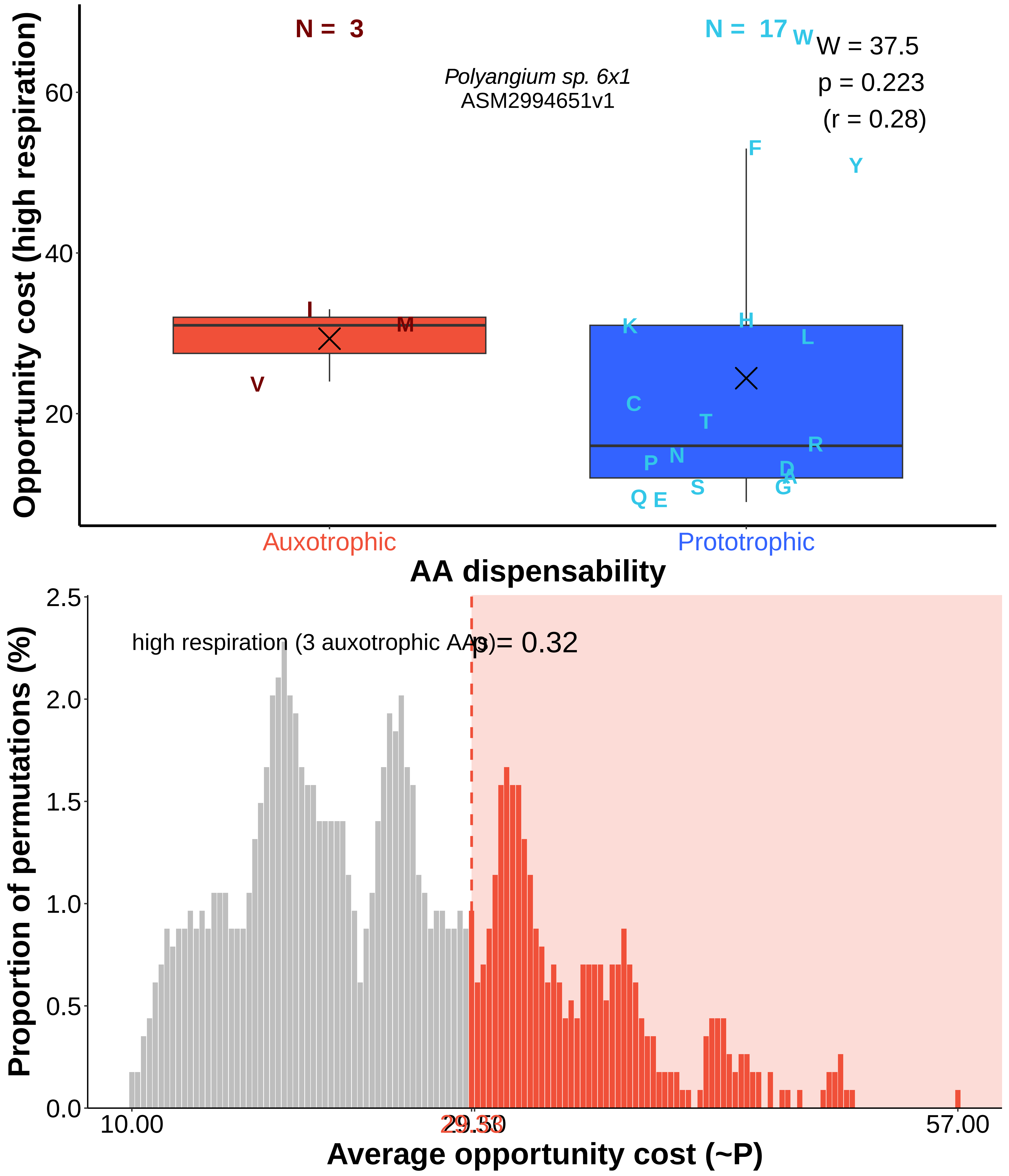

N = 3
N = 17
W = 37.5
p = 0.223
(r = 0.28)
Polyangium sp. 6x1
ASM2994651v1
Opportunity cost (high respiration)
60
40
20
Auxotrophic
Prototrophic
AA dispensability
I M V
K H L C T R P N D A S G Q E F Y W
high respiration (3 auxotrophic AAs)
p = 0.32
Proportion of permutations (%)
2.5
2.0
1.5
1.0
0.5
0.0
10.00
29.33
29.50
57.00
Average opportunity cost (~P)

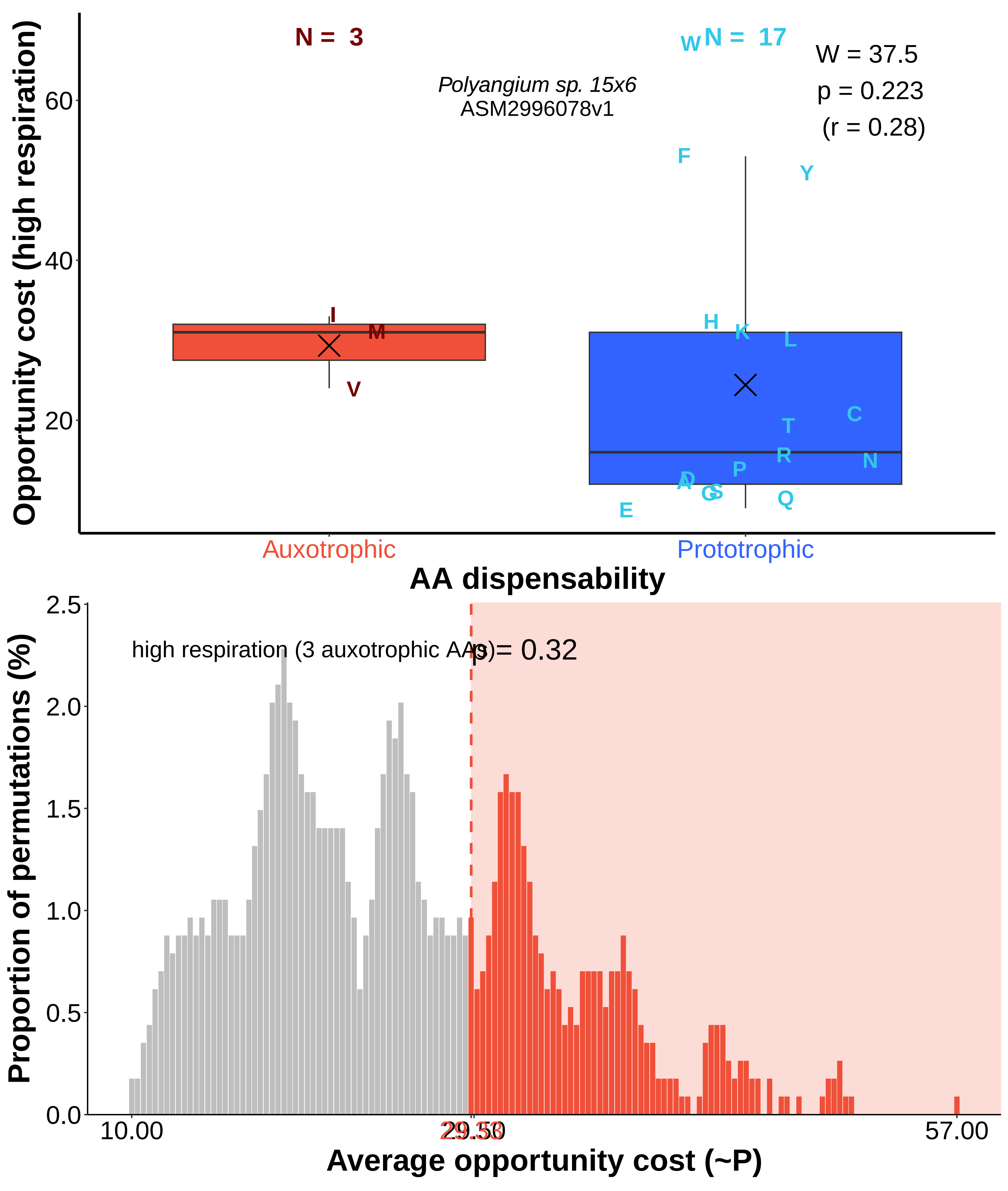

N = 3
N = 17
W
Polyangium sp. 15x6
ASM2996078v1
W = 37.5
p = 0.223
(r = 0.28)
Opportunity cost (high respiration)
60
40
20
F
Y
I
M
V
H
K
L
C
T
R
N
P
D
A
G
S
Q
E
Auxotrophic
Prototrophic
AA dispensability
high respiration (3 auxotrophic AAs)
p = 0.32
Proportion of permutations (%)
2.5
2.0
1.5
1.0
0.5
0.0
10.00
29.50
29.33
57.00
Average opportunity cost (~P)

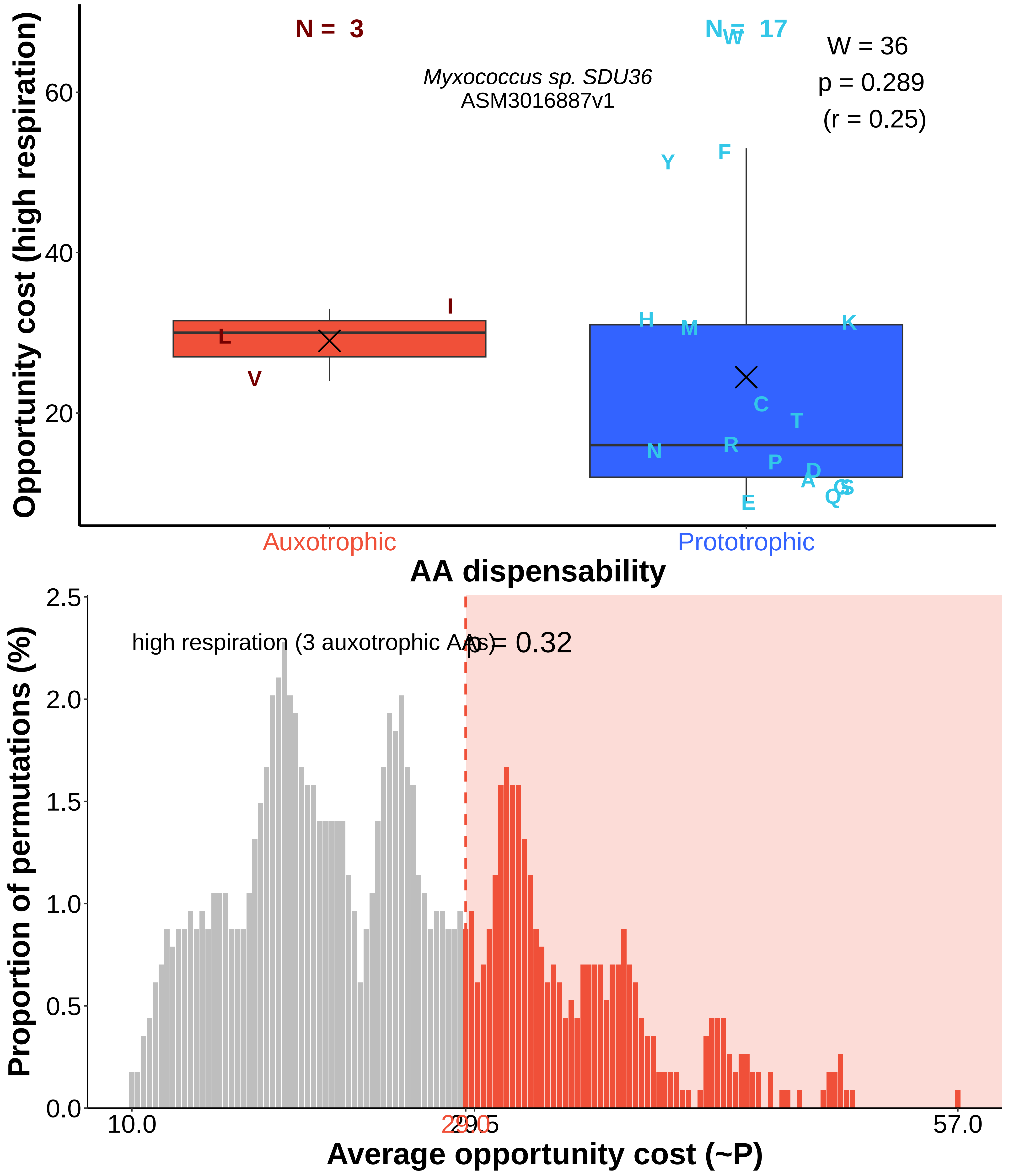
N = 3
N = 17
W = 36
p = 0.289
(r = 0.25)
Myxococcus sp. SDU36
ASM3016887v1
Opportunity cost (high respiration)
Auxotrophic
Prototrophic
AA dispensability
high respiration (3 auxotrophic AAs)
p = 0.32
Proportion of permutations (%)
Average opportunity cost (~P)

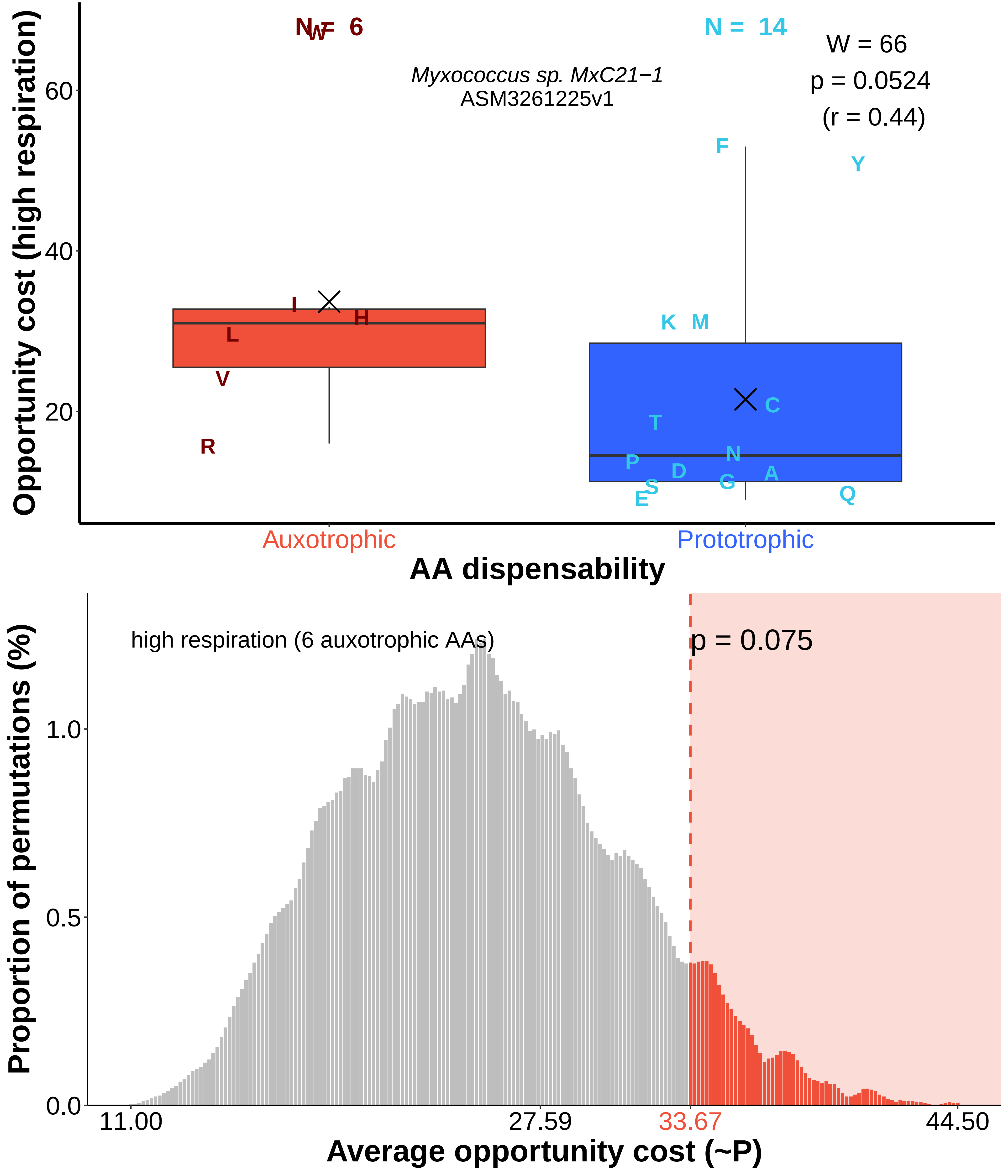
N = 6
W
N = 14
W = 66
p = 0.0524
(r = 0.44)
Myxococcus sp. MxC21−1
ASM3261225v1
F
Y
I
H
L
V
R
K M
C
T
N
P
D
A
S
G
E
Q
Opportunity cost (high respiration)
60
40
20
Auxotrophic
Prototrophic
AA dispensability
high respiration (6 auxotrophic AAs)
p = 0.075
Proportion of permutations (%)
1.0
0.5
0.0
11.00
27.59
33.67
44.50
Average opportunity cost (~P)

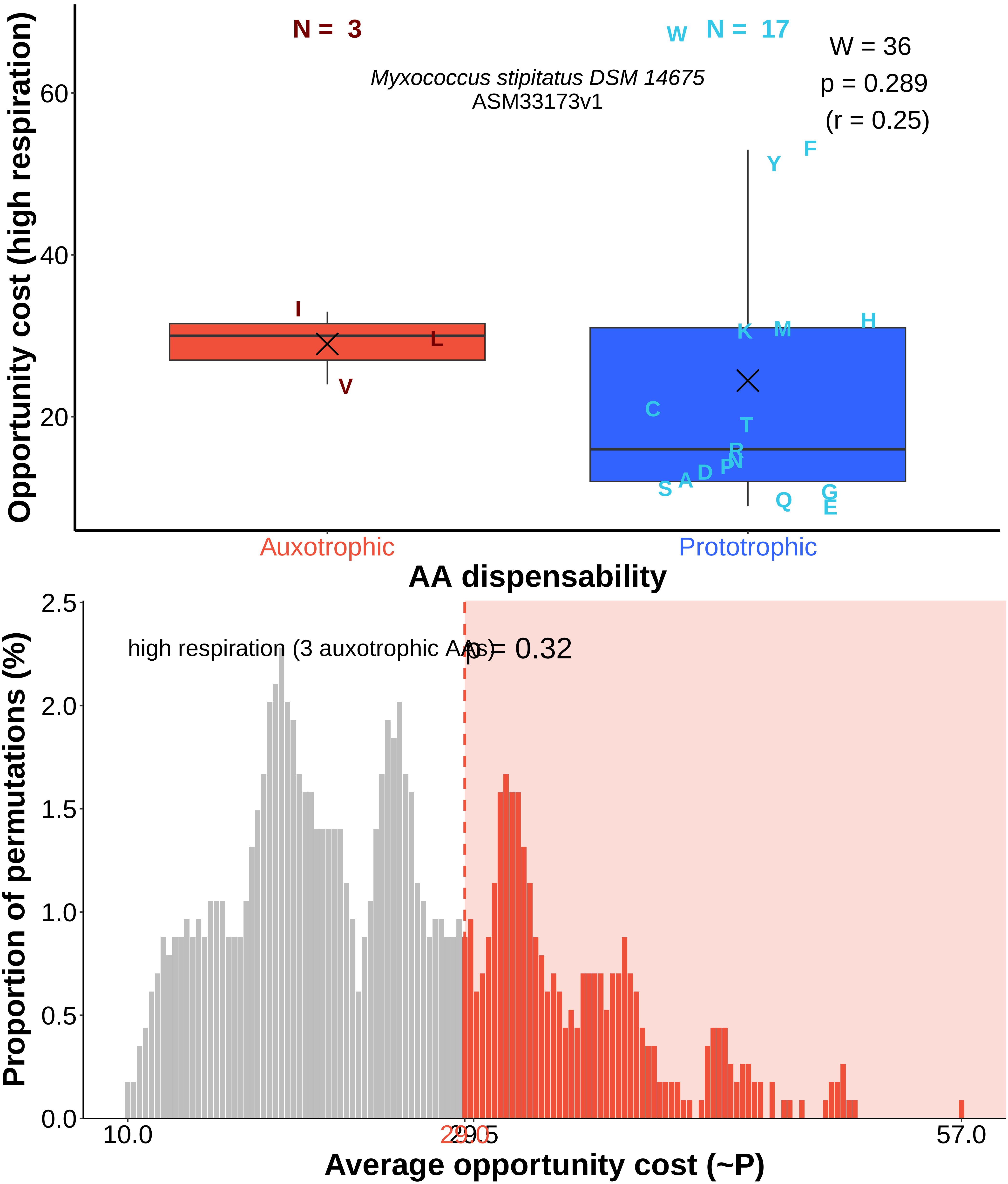
N = 3
W
N = 17
W = 36
p = 0.289
(r = 0.25)
Myxococcus stipitatus DSM 14675
ASM33173v1
Opportunity cost (high respiration)
60
40
20
I
L
V
Y
F
K
M
H
C
T
R
N
P
D
A
S
Q
G
E
Auxotrophic
Prototrophic
AA dispensability
high respiration (3 auxotrophic AAs)
p = 0.32
Proportion of permutations (%)
2.5
2.0
1.5
1.0
0.5
0.0
10.0
29.0
29.5
57.0
Average opportunity cost (~P)

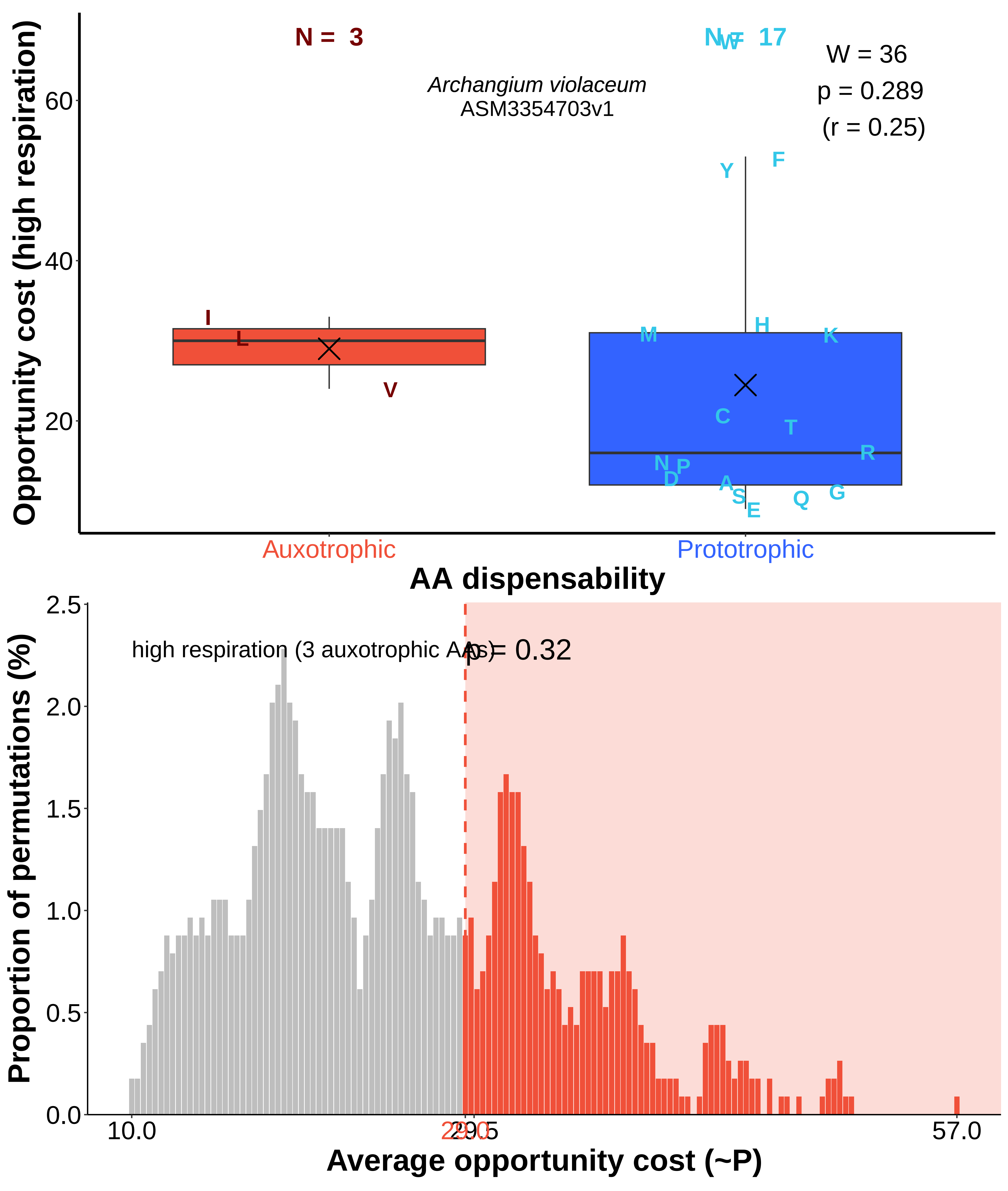

N = 3
N = 17
W
F
Archangium violaceum
ASM3354703v1
W = 36
p = 0.289
(r = 0.25)
Opportunity cost (high respiration)
60
40
20
I
L
V
Y
F
M
H
K
C
T
R
N
P
D
A
S
E
Q
G
Auxotrophic
Prototrophic
AA dispensability
2.5
2.0
1.5
1.0
0.5
0.0
Proportion of permutations (%)
high respiration (3 auxotrophic AAs)
p = 0.32
10.0
29.0
29.5
57.0
Average opportunity cost (~P)

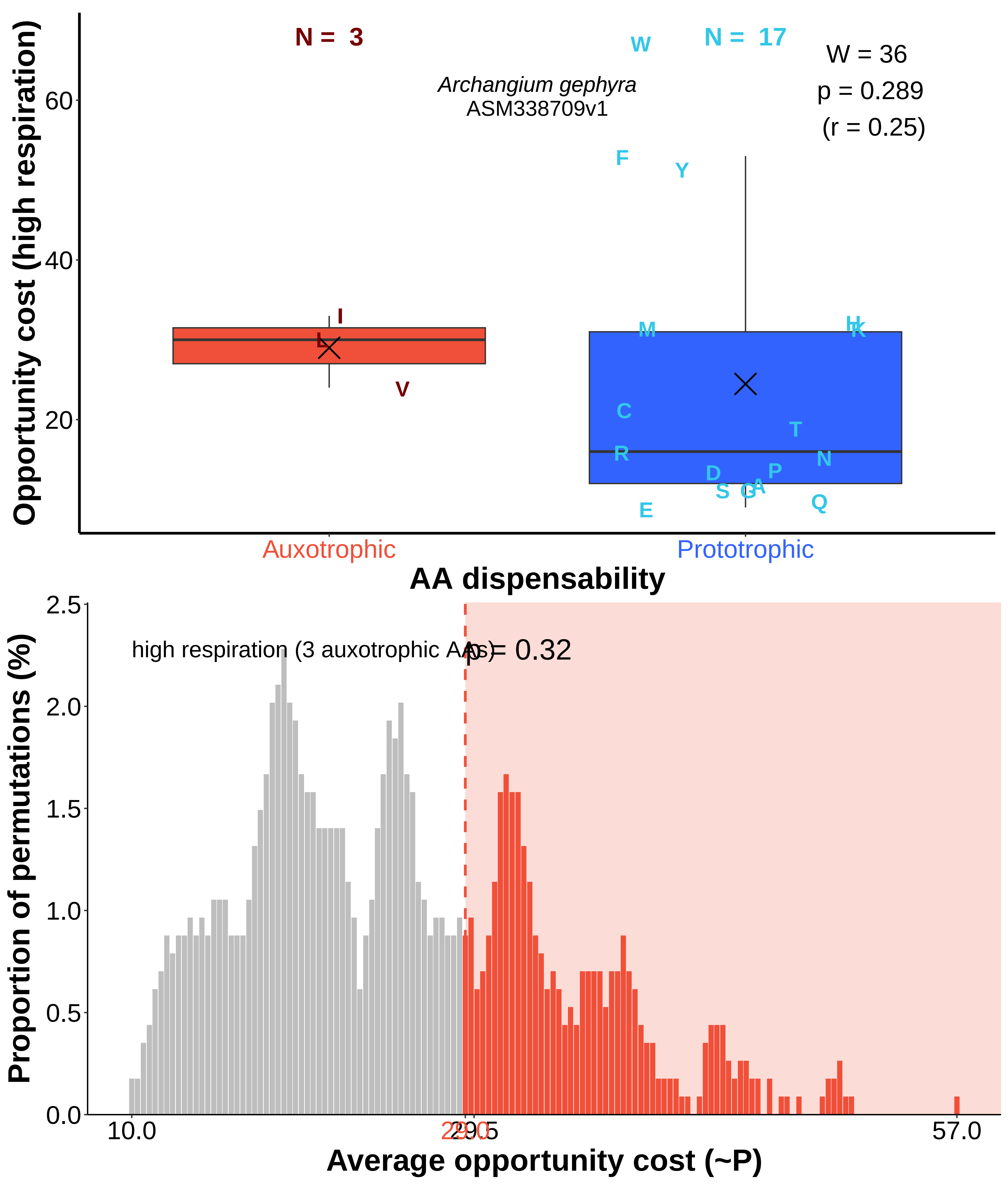
N = 3
N = 17
W
W = 36
p = 0.289
(r = 0.25)
Archangium gephyra
ASM338709v1
Opportunity cost (high respiration)
Auxotrophic
Prototrophic
AA dispensability
high respiration (3 auxotrophic AAs)
p = 0.32
Proportion of permutations (%)
Average opportunity cost (~P)
10.0
29.0
29.5
57.0

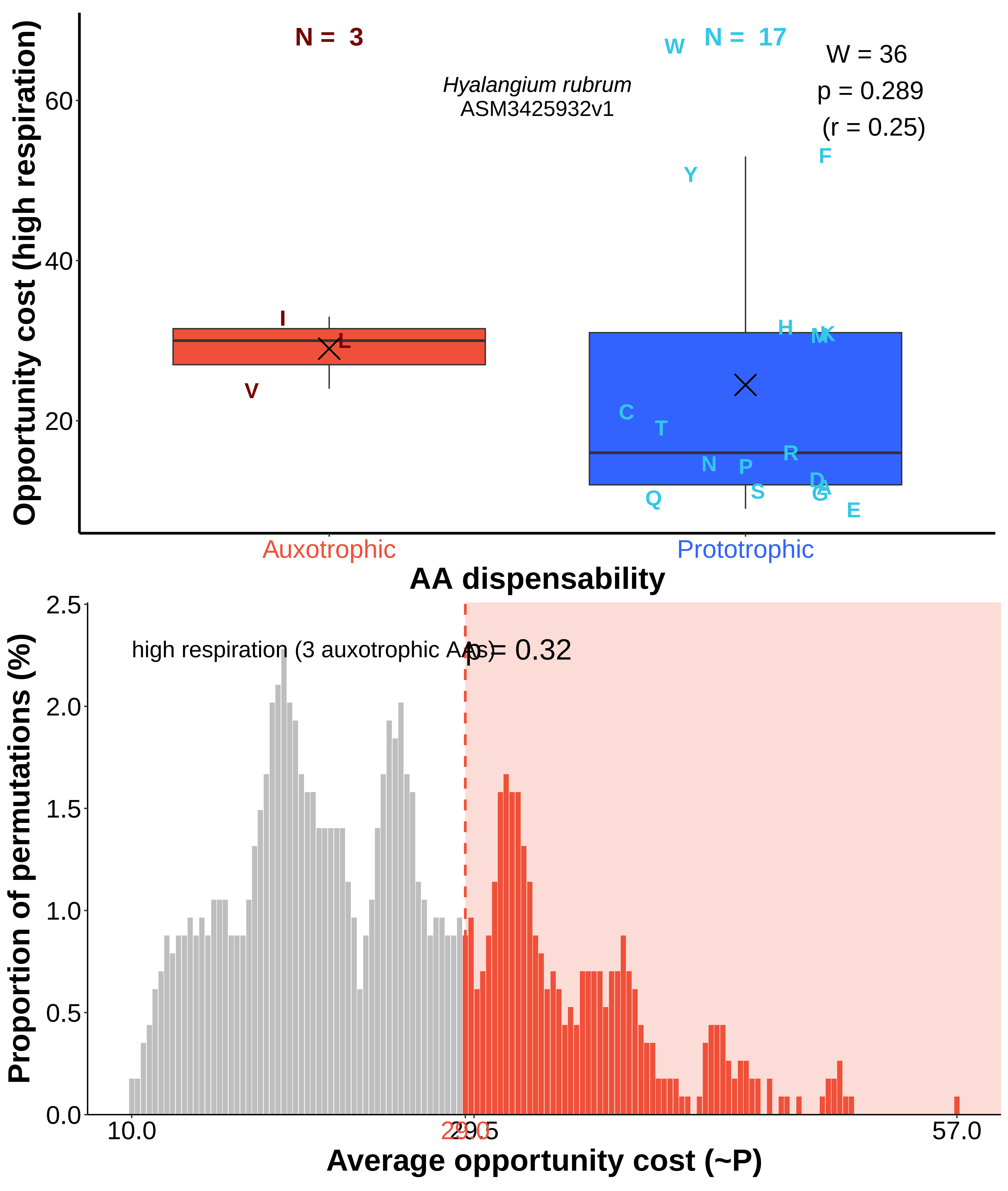

N = 3
N = 17
Hyalangium rubrum
ASM3425932v1
W = 36
p = 0.289
(r = 0.25)
Opportunity cost (high respiration)
Auxotrophic
Prototrophic
AA dispensability
high respiration (3 auxotrophic AAs)
p = 0.32
Proportion of permutations (%)
Average opportunity cost (~P)

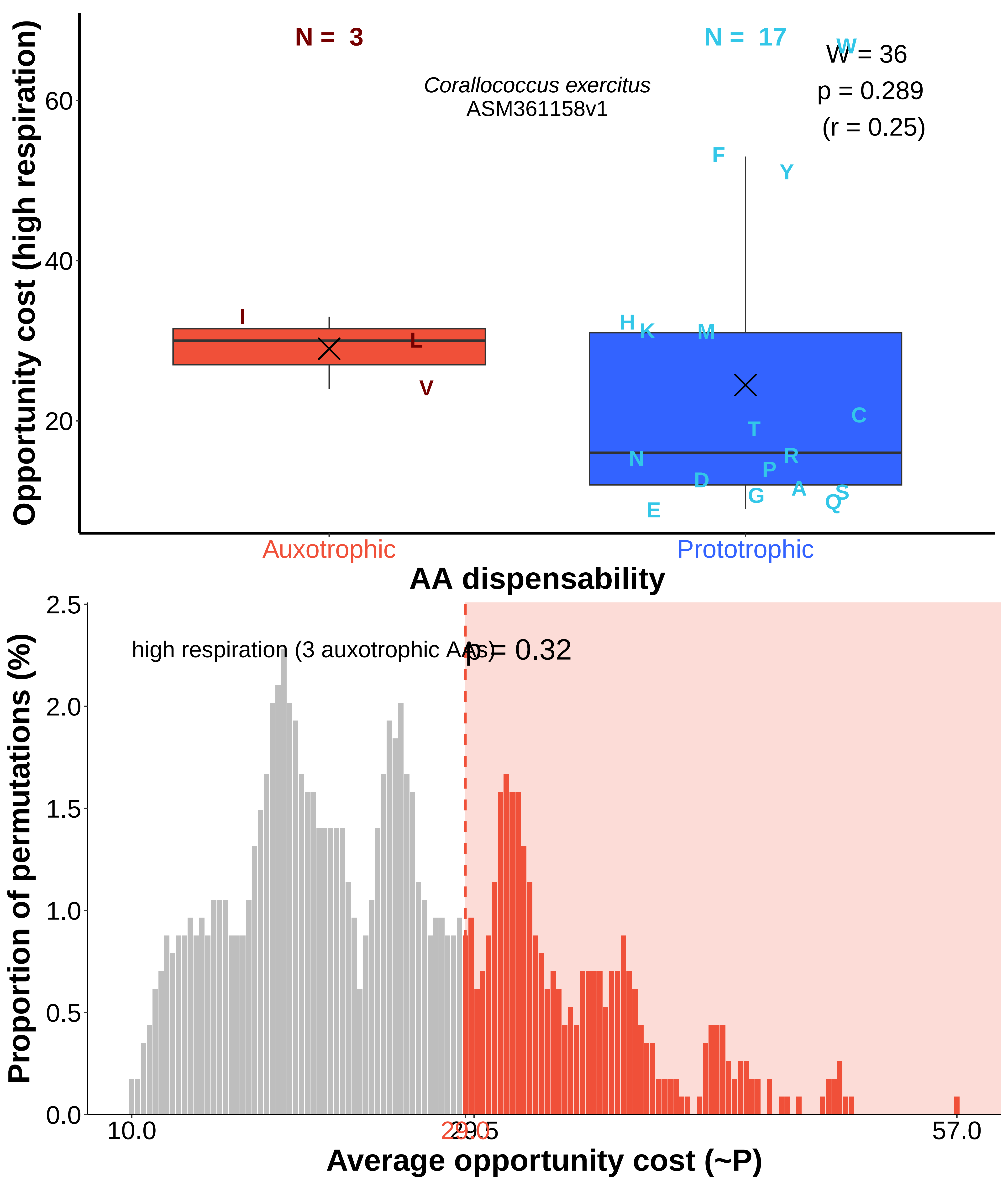

N = 3
N = 17
W = 36
p = 0.289
(r = 0.25)
Corallococcus exercitus
ASM361158v1
Opportunity cost (high respiration)
60
40
20
I
L
V
F
Y
H
K
M
C
T
N
R
P
D
G
A
S
Q
E
W
Auxotrophic
Prototrophic
AA dispensability
Proportion of permutations (%)
2.5
2.0
1.5
1.0
0.5
0.0
high respiration (3 auxotrophic AAs)
p = 0.32
10.0
29.0
29.5
57.0
Average opportunity cost (~P)

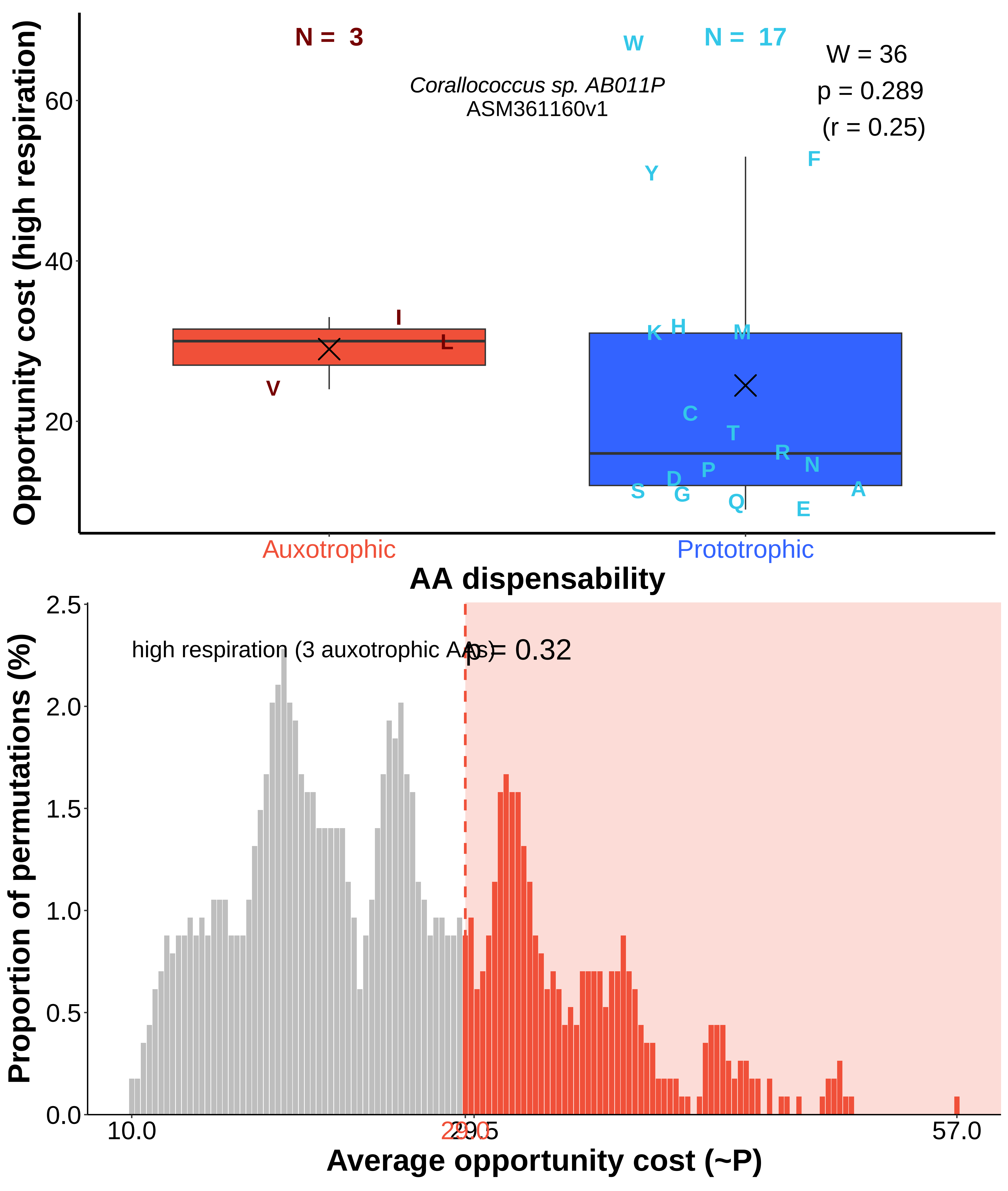

N = 3
W
N = 17
W = 36
p = 0.289
(r = 0.25)
Corallococcus sp. AB011P
ASM361160v1
Y
F
I
L
V
K
H
M
C
T
R
N
P
D
S
G
Q
E
A
Opportunity cost (high respiration)
60
40
20
Auxotrophic
Prototrophic
AA dispensability
high respiration (3 auxotrophic AAs)
p = 0.32
Proportion of permutations (%)
2.5
2.0
1.5
1.0
0.5
0.0
10.0
29.0
29.5
57.0
Average opportunity cost (~P)

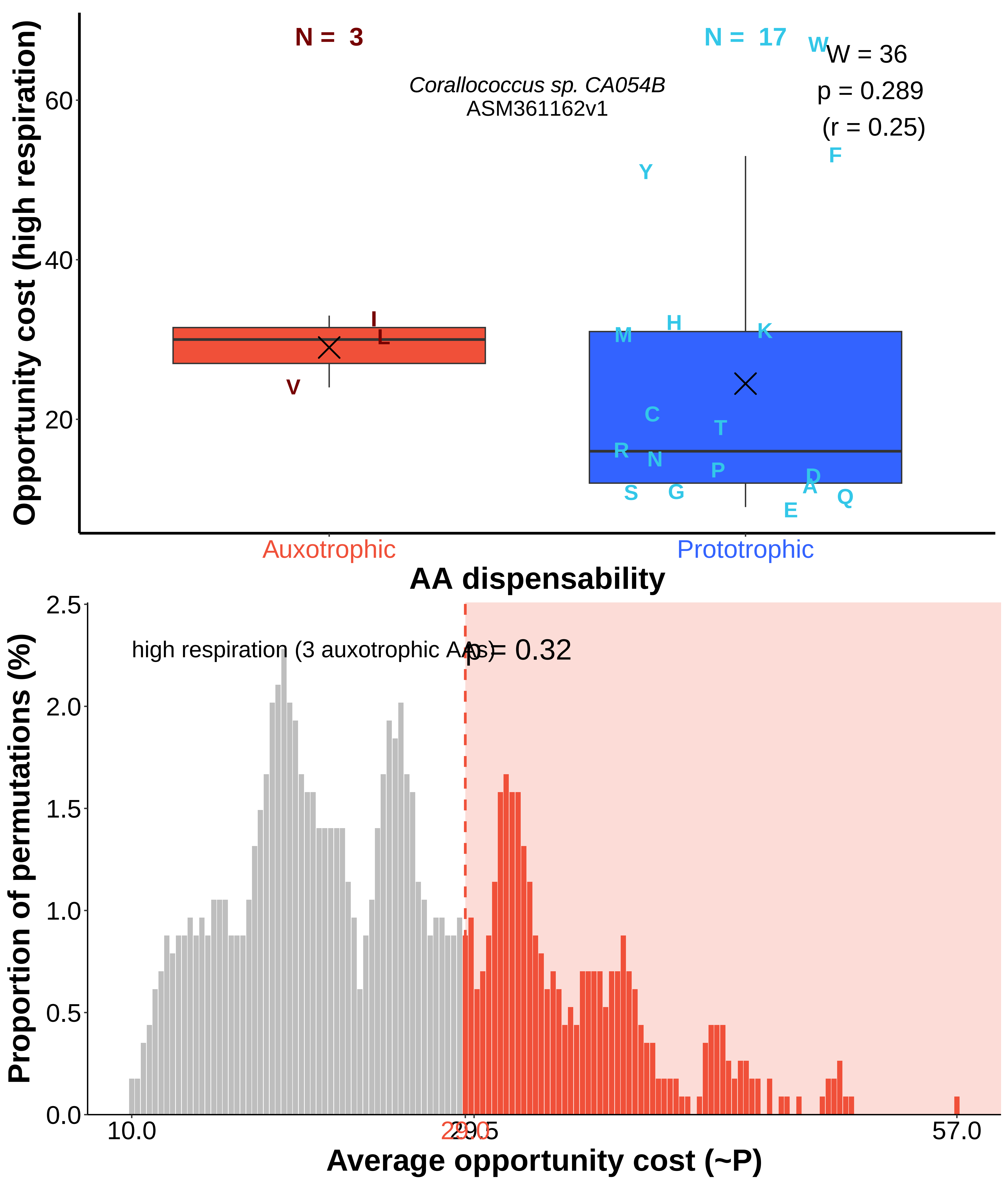
N = 3
N = 17
Corallococcus sp. CA054B
ASM361162v1
W = 36
p = 0.289
(r = 0.25)
Opportunity cost (high respiration)
60
40
20
Y
F
W
I
L
V
M
H
K
C
T
R
N
P
D
A
S
G
Q
E
Auxotrophic
Prototrophic
AA dispensability
high respiration (3 auxotrophic AAs)
p = 0.32
Proportion of permutations (%)
2.5
2.0
1.5
1.0
0.5
0.0
10.0
29.0
29.5
57.0
Average opportunity cost (~P)

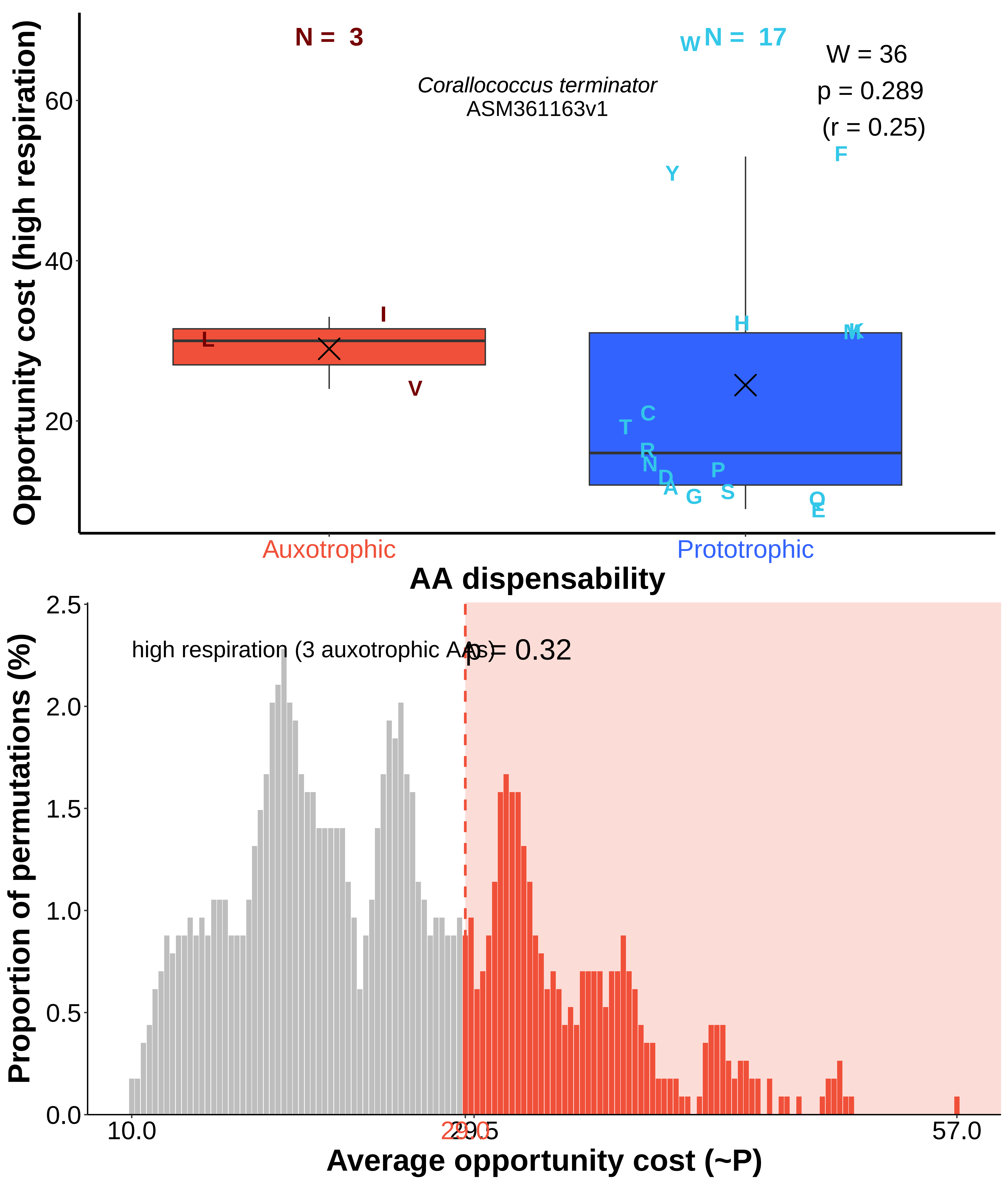

N = 3
N = 17
W
Corallococcus terminator
ASM361163v1
W = 36
p = 0.289
(r = 0.25)
Opportunity cost (high respiration)
60
40
20
Auxotrophic
Prototrophic
AA dispensability
high respiration (3 auxotrophic AAs)
p = 0.32
Proportion of permutations (%)
2.5
2.0
1.5
1.0
0.5
0.0
10.0
29.0
57.0
Average opportunity cost (~P)

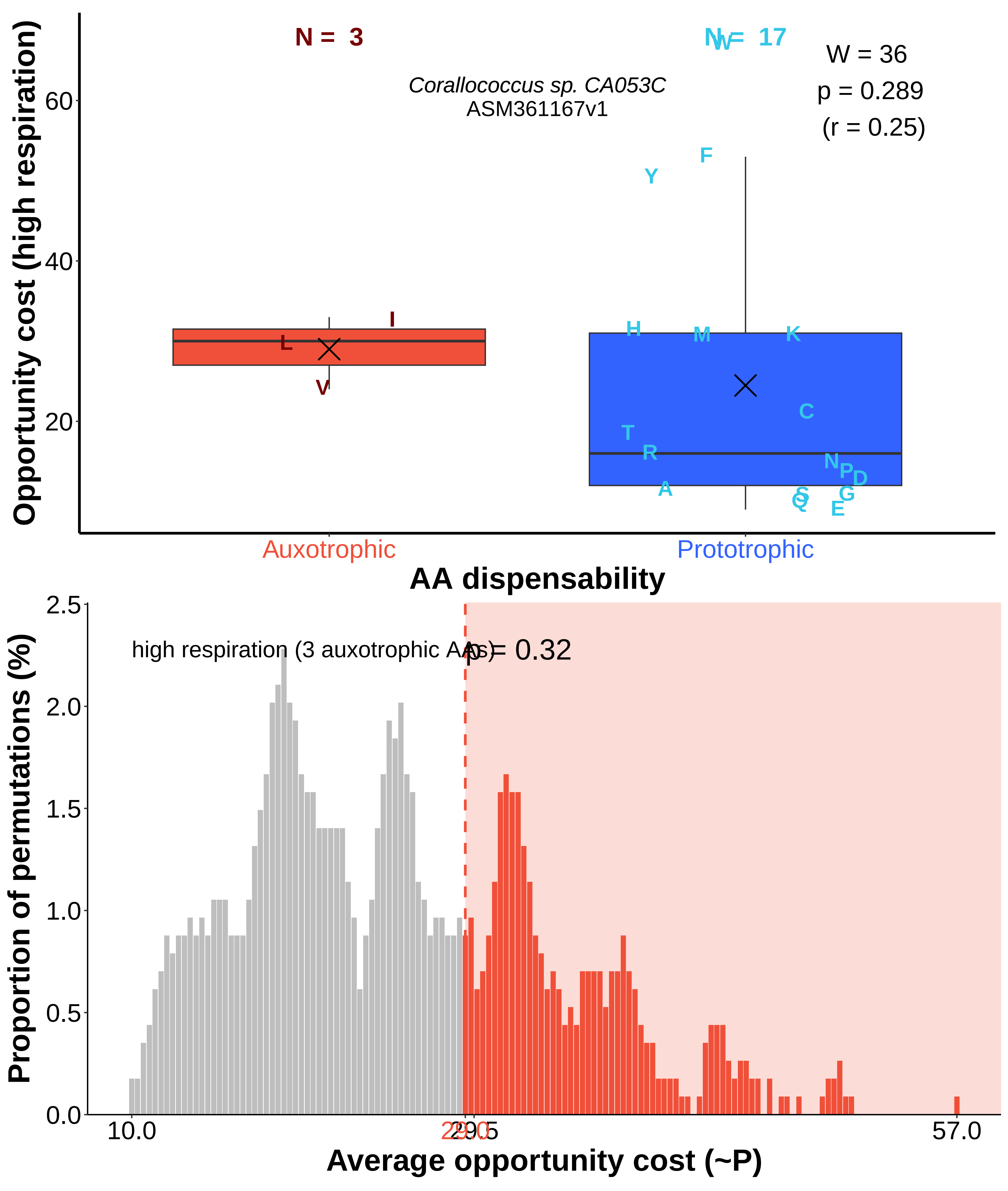
N = 3
N = 17
W
Corallococcus sp. CA053C
ASM361167v1
W = 36
p = 0.289
(r = 0.25)
Opportunity cost (high respiration)
60
40
20
F
Y
I
L
V
H
M
K
C
T
R
N
P
D
A
S
Q
G
E
Auxotrophic
Prototrophic
AA dispensability
Proportion of permutations (%)
2.5
2.0
1.5
1.0
0.5
0.0
high respiration (3 auxotrophic AAs)
p = 0.32
10.0
29.0
29.5
57.0
Average opportunity cost (~P)

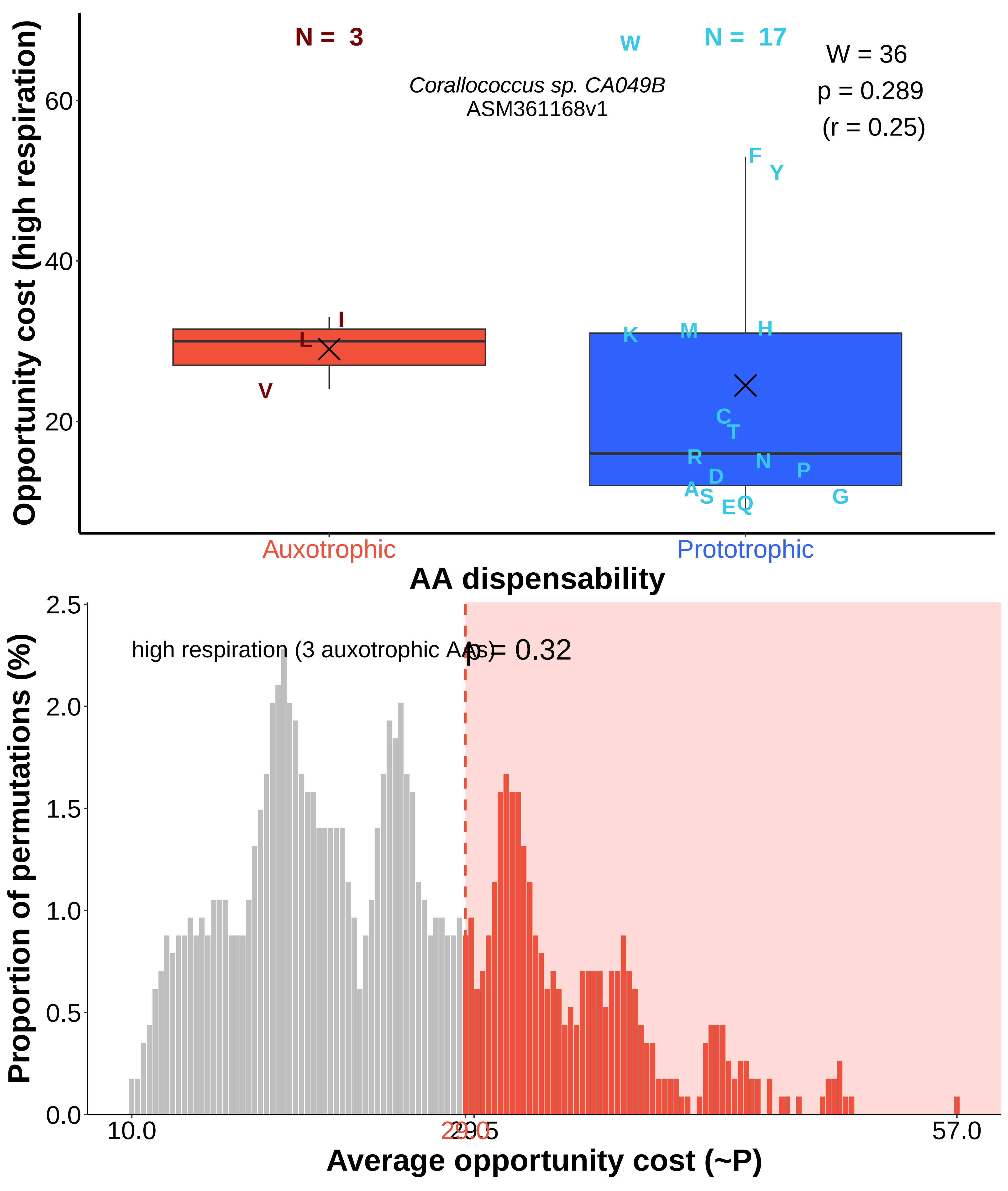

N = 3
W
N = 17
W = 36
p = 0.289
(r = 0.25)
Corallococcus sp. CA049B
ASM361168v1
Opportunity cost (high respiration)
60
40
20
F
Y
I
L
V
K
M
H
C
T
R
N
P
D
A
S
E
Q
G
Auxotrophic
Prototrophic
AA dispensability
Proportion of permutations (%)
2.5
2.0
1.5
1.0
0.5
0.0
high respiration (3 auxotrophic AAs)
p = 0.32
10.0
29.0
29.5
57.0
Average opportunity cost (~P)

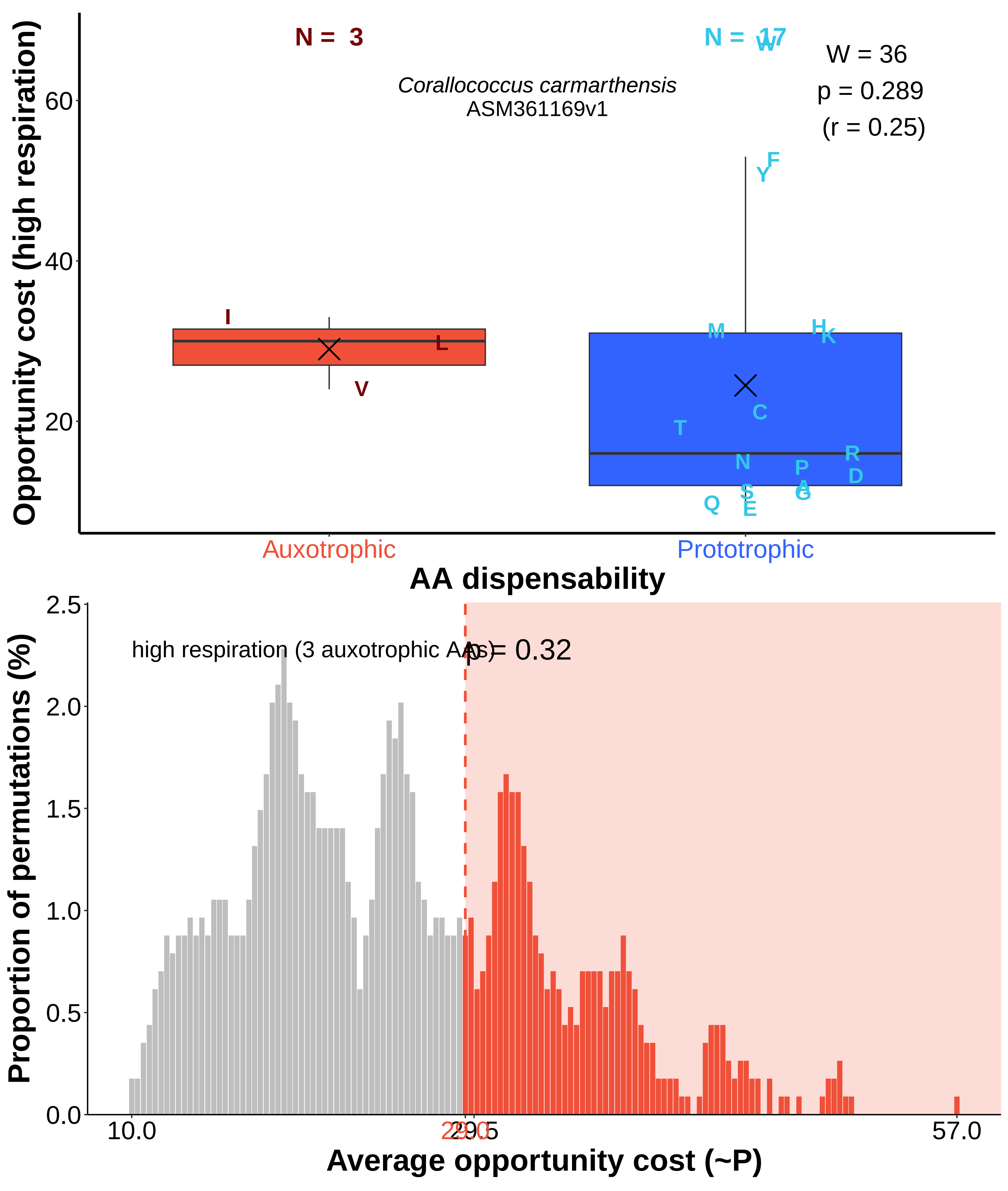
N = 3
N = 17
W = 36
p = 0.289
(r = 0.25)
Corallococcus carmarthensis
ASM361169v1
Opportunity cost (high respiration)
60
40
20
I
L
V
F
Y
M
H
K
C
T
R
N
P
D
A
G
S
Q
E
W
Auxotrophic
Prototrophic
AA dispensability
Proportion of permutations (%)
2.5
2.0
1.5
1.0
0.5
0.0
high respiration (3 auxotrophic AAs)
p = 0.32
10.0
29.0
29.5
57.0
Average opportunity cost (~P)

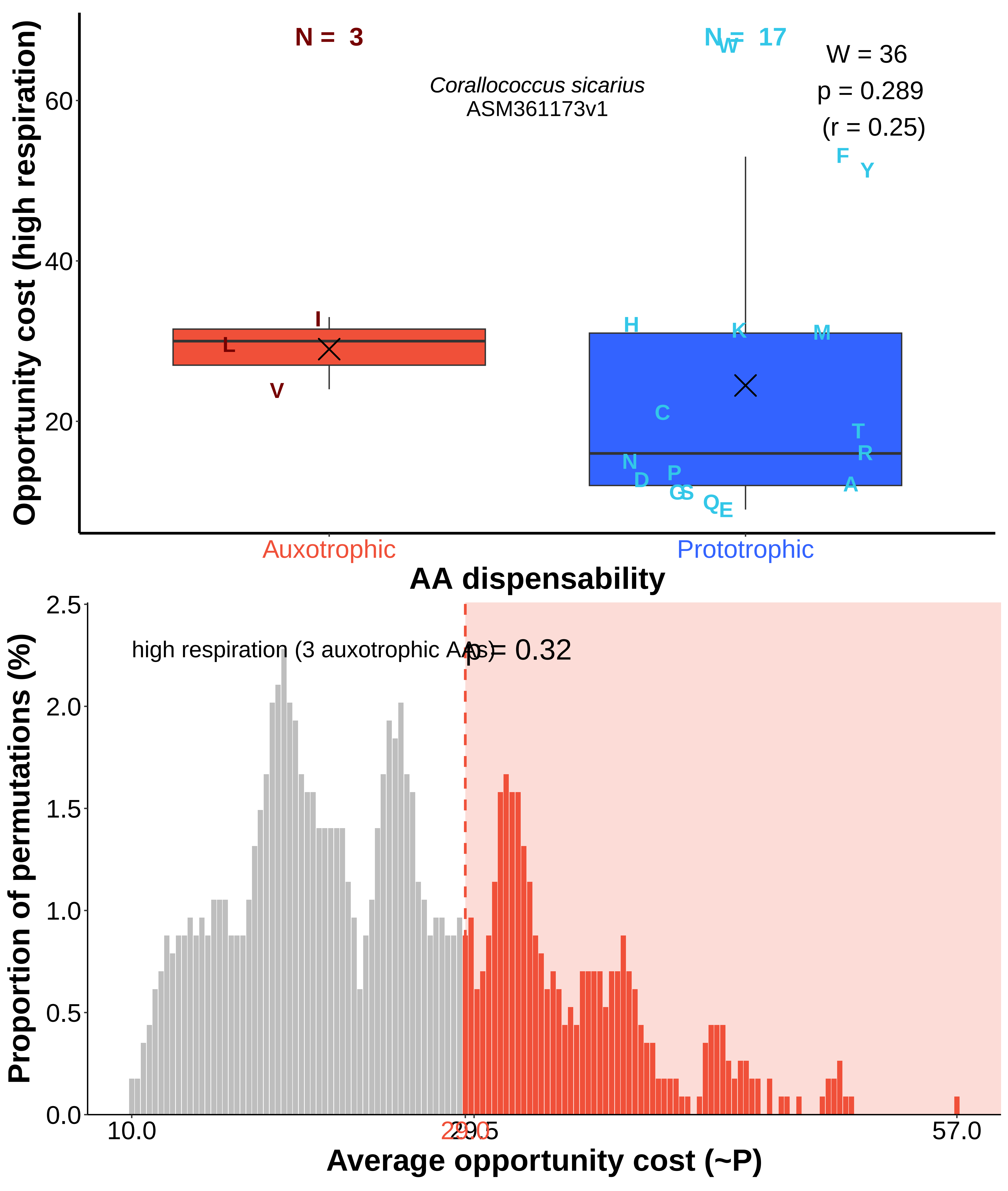
N = 3
N = 17
W = 36
p = 0.289
(r = 0.25)
Corallococcus sicarius
ASM361173v1
Opportunity cost (high respiration)
Auxotrophic
Prototrophic
AA dispensability
high respiration (3 auxotrophic AAs)
p = 0.32
Proportion of permutations (%)
Average opportunity cost (~P)
10.0
29.0
57.0

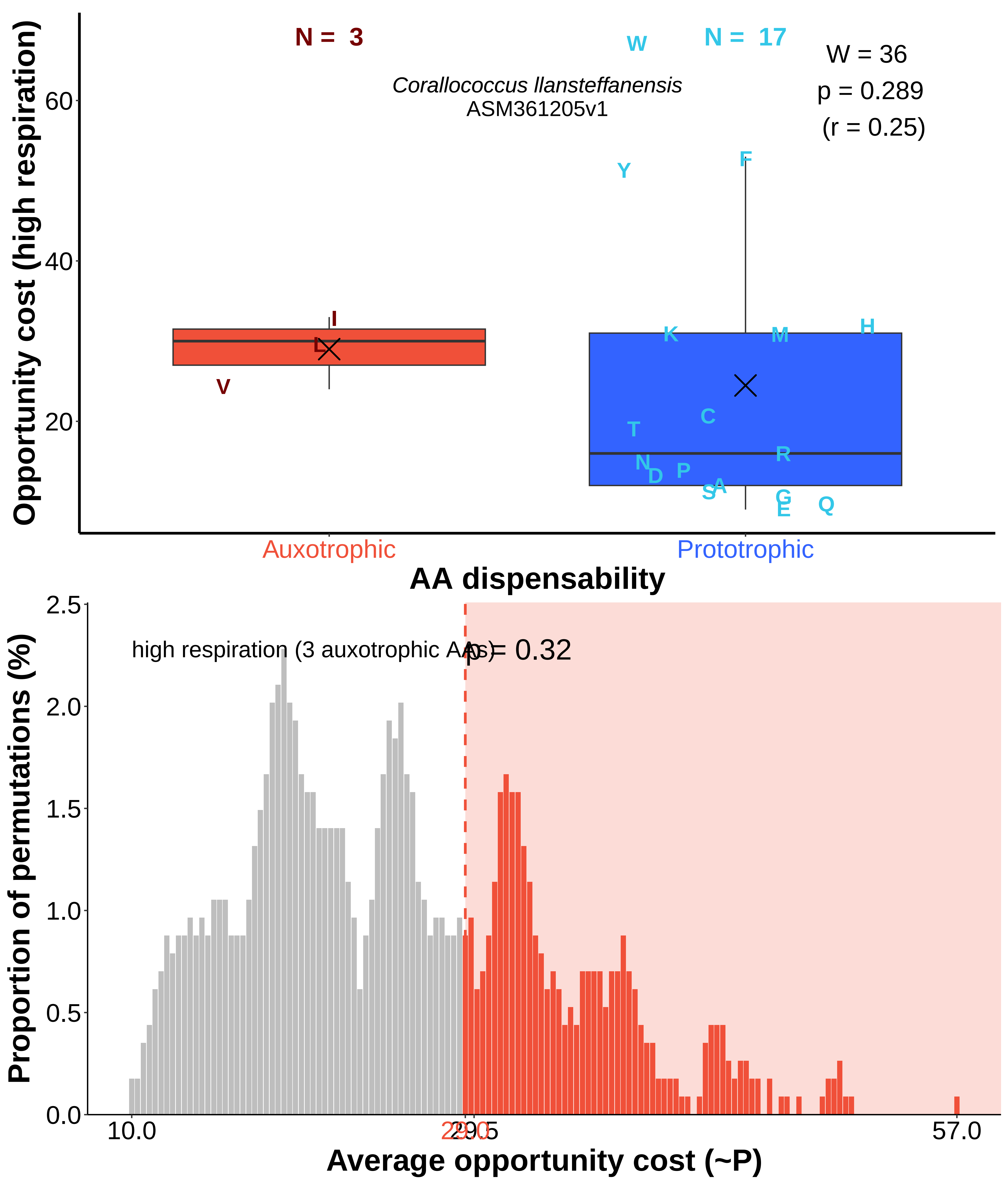
N = 3
N = 17
W = 36
p = 0.289
(r = 0.25)
Corallococcus llansteffanensis
ASM361205v1
Opportunity cost (high respiration)
60
40
20
W
Y
F
I
L
V
K
M
H
C
T
R
N
D
P
A
S
G
E
Q
Auxotrophic
Prototrophic
AA dispensability
high respiration (3 auxotrophic AAs)
p = 0.32
Proportion of permutations (%)
2.5
2.0
1.5
1.0
0.5
0.0
10.0
29.0
29.5
57.0
Average opportunity cost (~P)

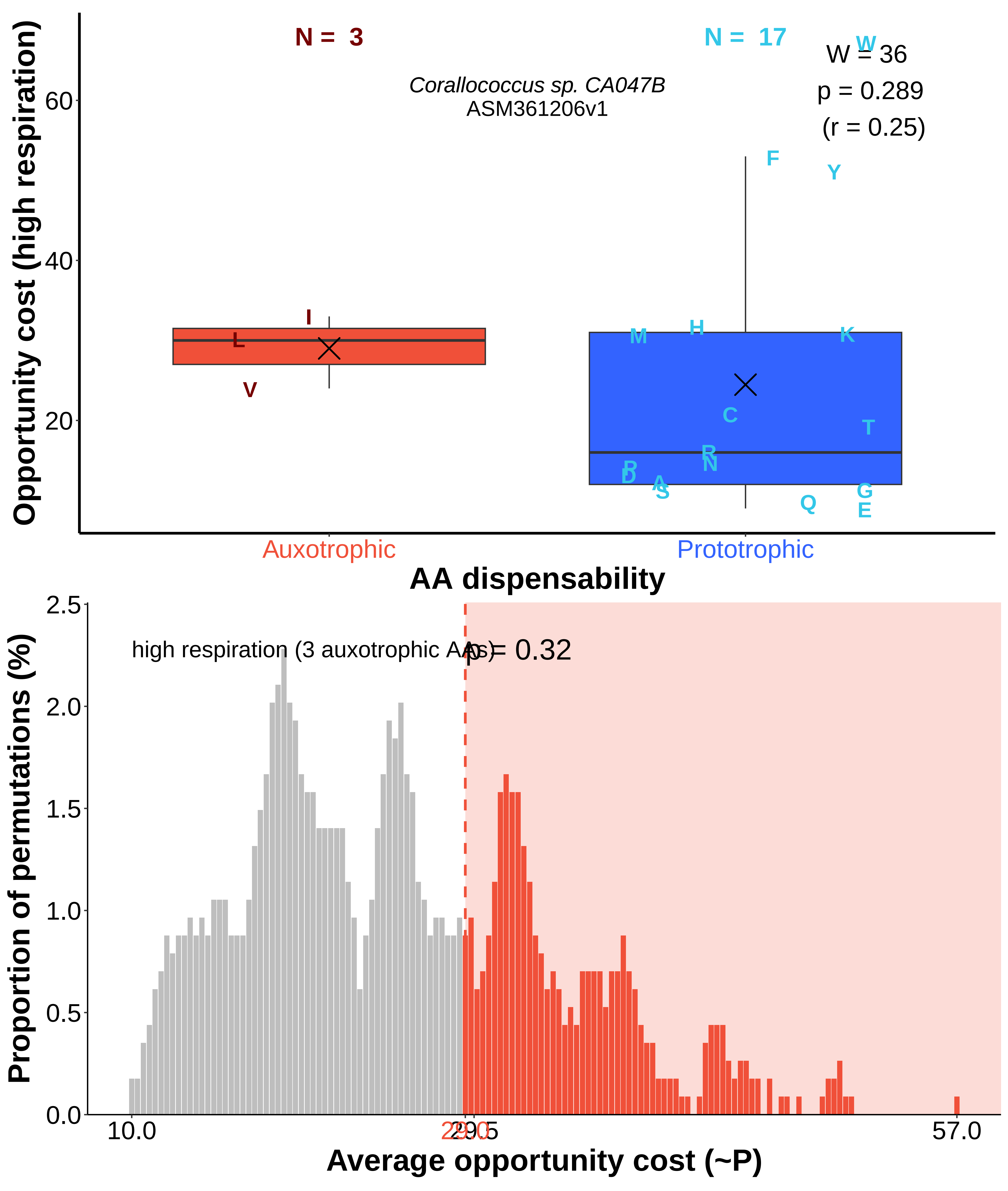
N = 3
N = 17
Corallococcus sp. CA047B
ASM361206v1
W = 36
p = 0.289
(r = 0.25)
Opportunity cost (high respiration)
Auxotrophic
Prototrophic
AA dispensability
high respiration (3 auxotrophic AAs)
p = 0.32
Proportion of permutations (%)
Average opportunity cost (~P)
10.0
29.0
29.5
57.0

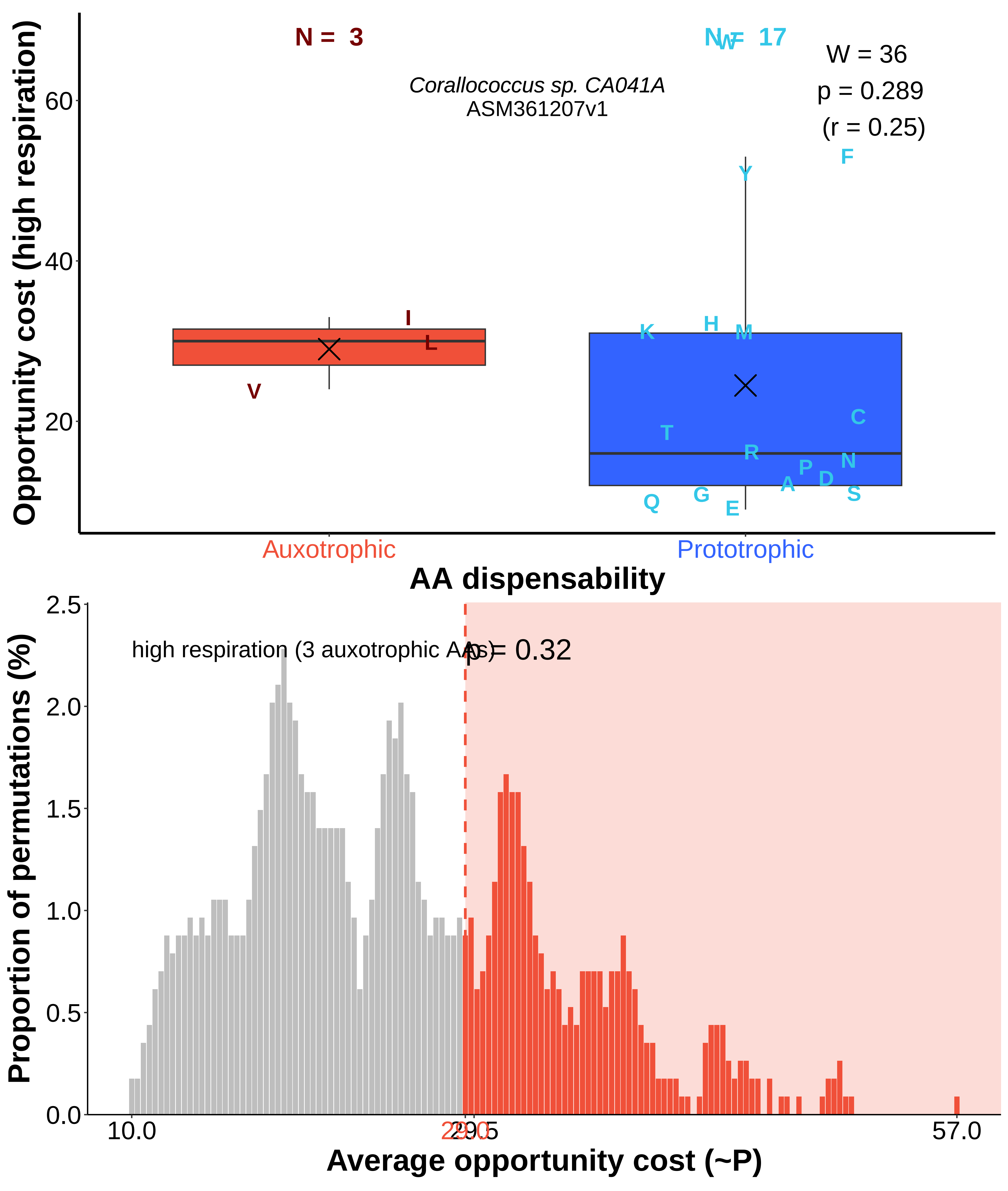

N = 3
N = 17
W
W = 36
p = 0.289
(r = 0.25)
Corallococcus sp. CA041A
ASM361207v1
Opportunity cost (high respiration)
60
40
20
F
Y
I
L
V
K
H
M
C
T
R
P
N
D
A
S
Q
G
E
Auxotrophic
Prototrophic
AA dispensability
high respiration (3 auxotrophic AAs)
p = 0.32
Proportion of permutations (%)
2.5
2.0
1.5
1.0
0.5
0.0
10.0
29.0
29.5
57.0
Average opportunity cost (~P)

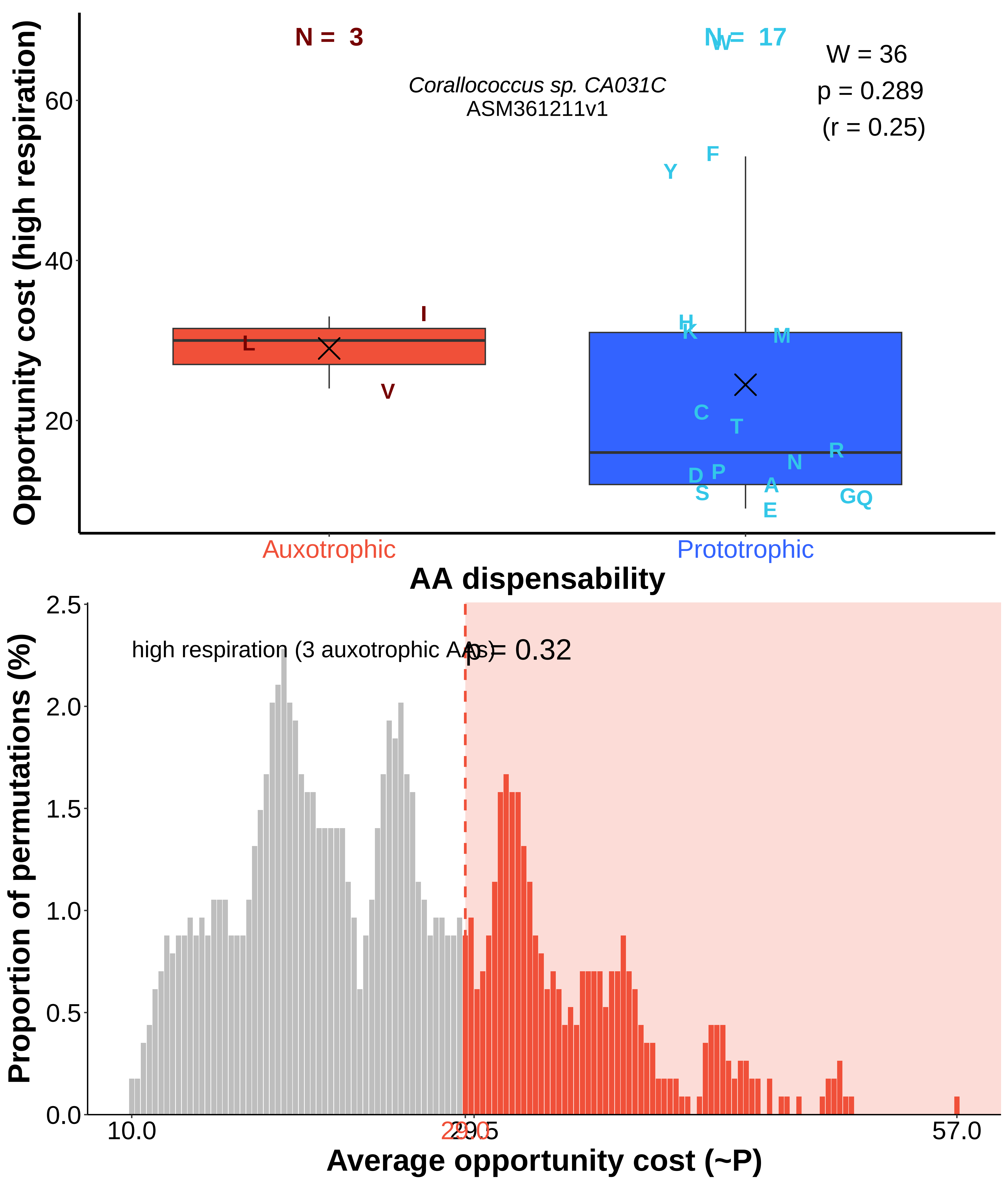

N = 3
N = 17
W
W = 36
p = 0.289
(r = 0.25)
Corallococcus sp. CA031C
ASM361211v1
Opportunity cost (high respiration)
60
40
20
F
Y
I
H
K
M
L
V
C
T
R
N
D
P
A
S
GQ
E
Auxotrophic
Prototrophic
AA dispensability
high respiration (3 auxotrophic AAs)
p = 0.32
Proportion of permutations (%)
2.5
2.0
1.5
1.0
0.5
0.0
10.0
29.0
29.5
57.0
Average opportunity cost (~P)

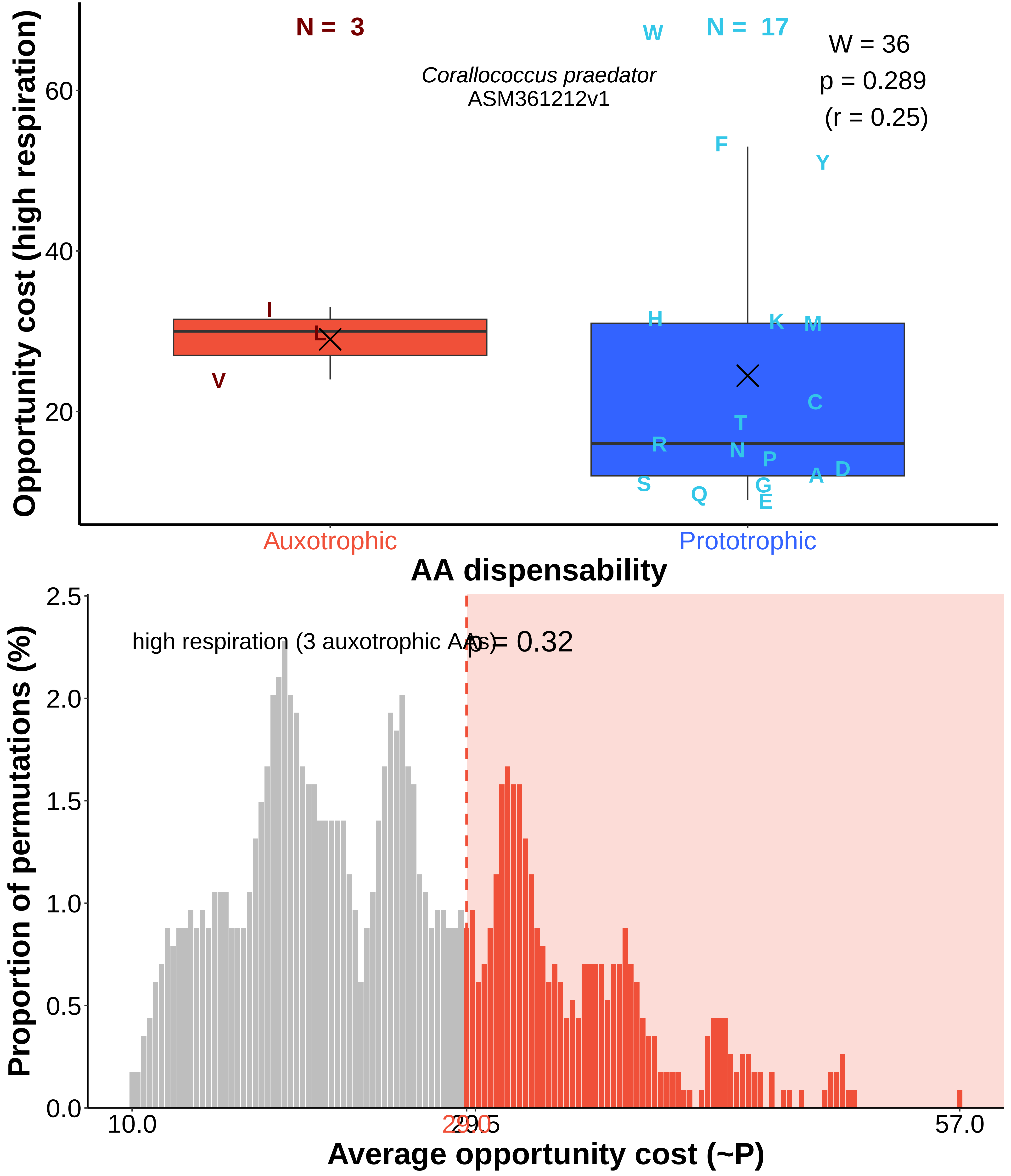
N = 3
W
N = 17
W = 36
p = 0.289
(r = 0.25)
Corallococcus praedator
ASM361212v1
F
Y
60
40
20
I
L
V
H
K
M
C
T
R
N
P
A
D
S
Q
G
E
Opportunity cost (high respiration)
Auxotrophic
Prototrophic
AA dispensability
2.5
2.0
1.5
1.0
0.5
0.0
high respiration (3 auxotrophic AAs)
p = 0.32
Proportion of permutations (%)
10.0
29.0
29.5
57.0
Average opportunity cost (~P)

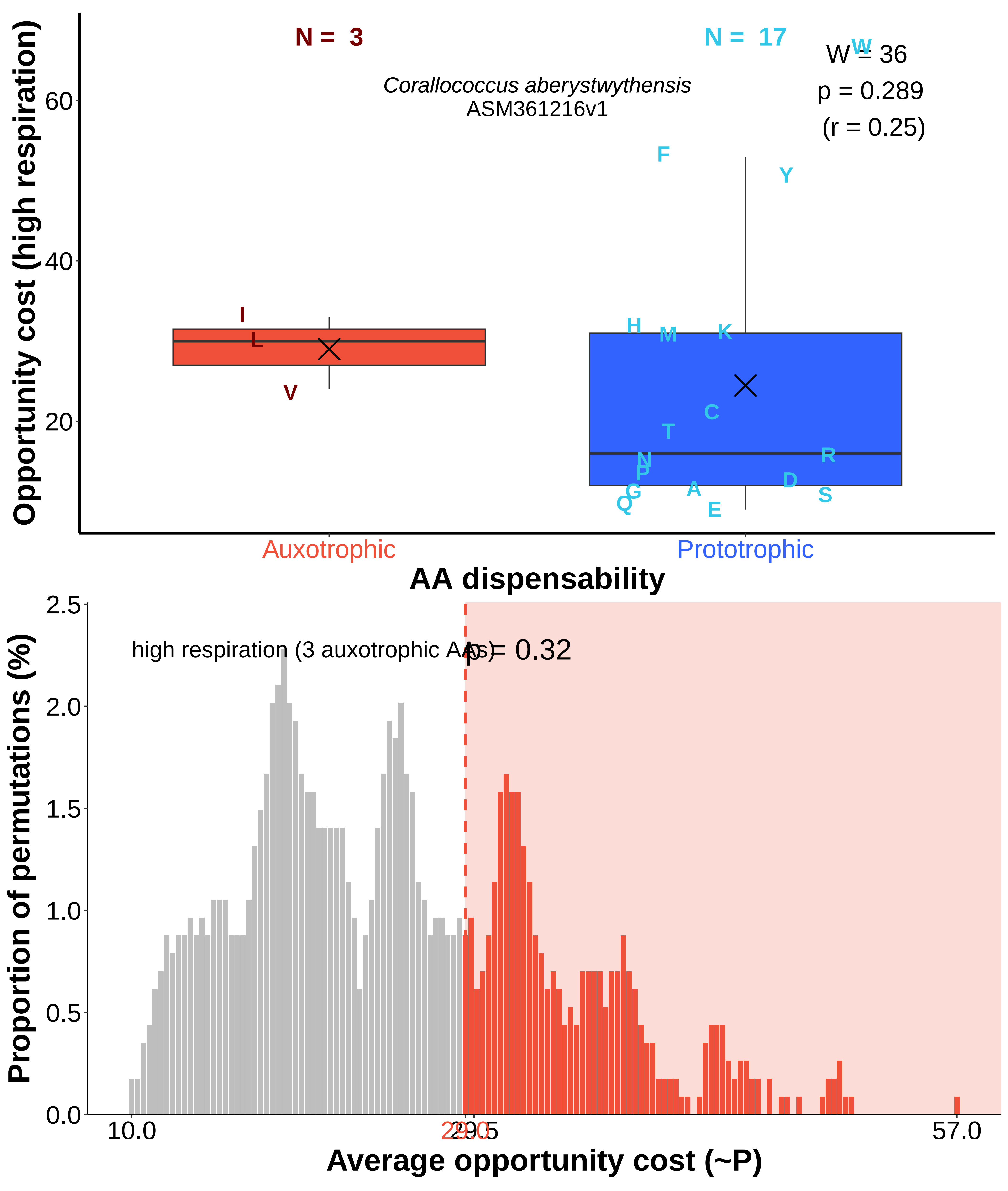
N = 3
N = 17
Corallococcus aberystwythensis
ASM361216v1
W = 36
p = 0.289
(r = 0.25)
Opportunity cost (high respiration)
60
40
20
I
L
V
F
Y
W
H
M
K
C
T
N
P
R
D
G
A
Q
S
E
Auxotrophic
Prototrophic
AA dispensability
high respiration (3 auxotrophic AAs)
p = 0.32
Proportion of permutations (%)
2.5
2.0
1.5
1.0
0.5
0.0
10.0
29.0
29.5
57.0
Average opportunity cost (~P)

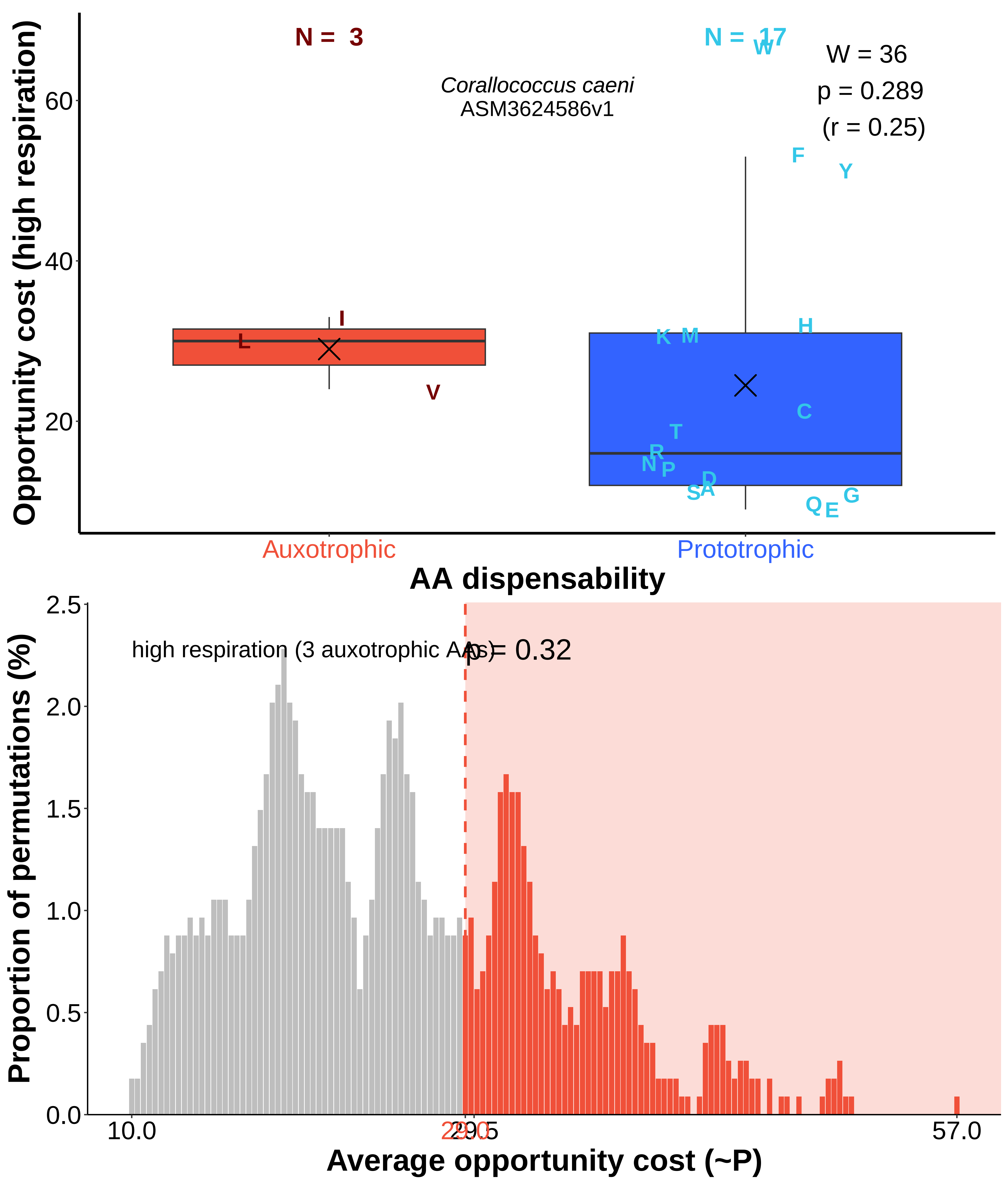

N = 3
N = 17
W = 36
p = 0.289
(r = 0.25)
Corallococcus caeni
ASM3624586v1
Opportunity cost (high respiration)
Auxotrophic
Prototrophic
AA dispensability
high respiration (3 auxotrophic AAs)
p = 0.32
Proportion of permutations (%)
Average opportunity cost (~P)
10.0
29.0
29.5
57.0

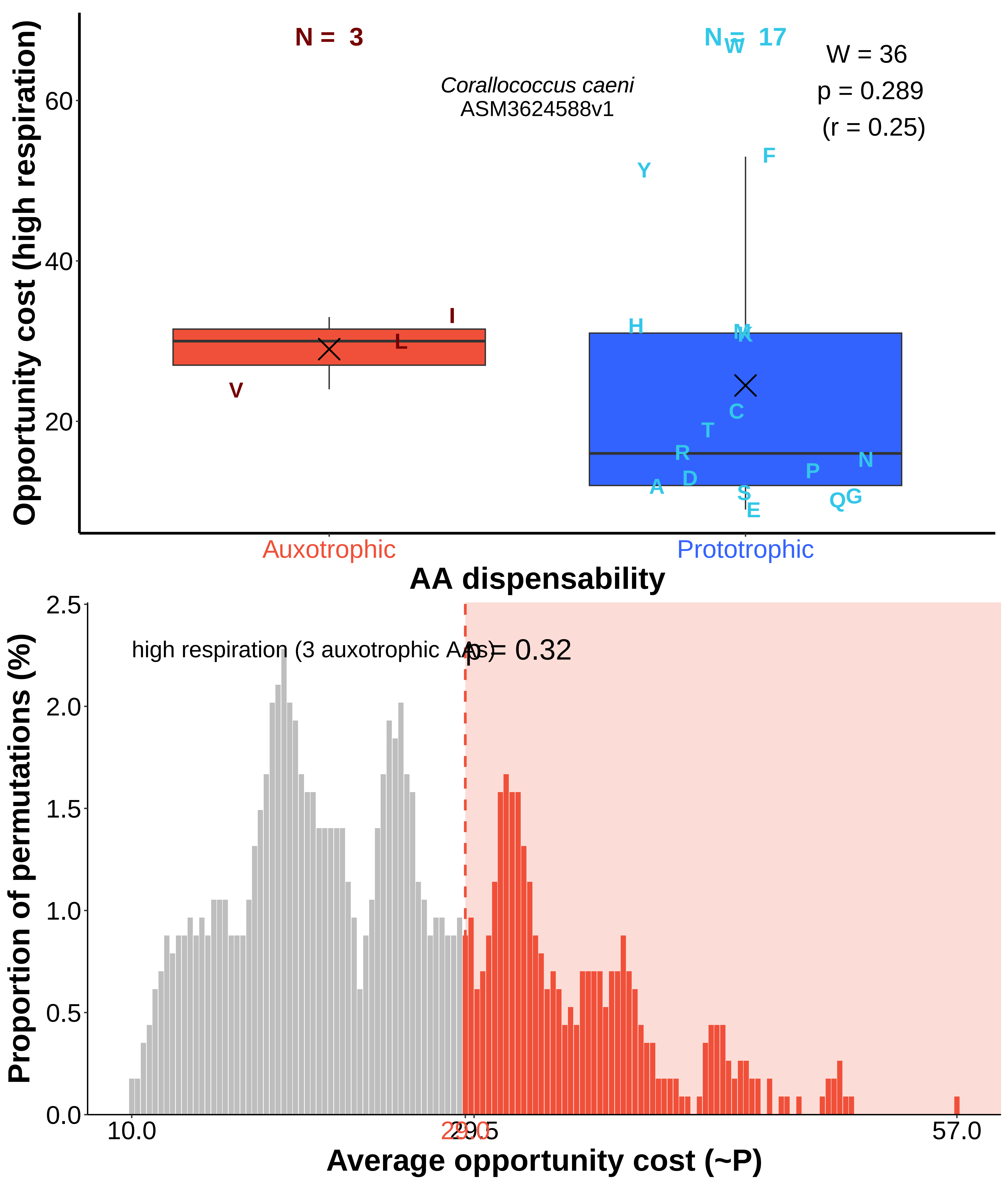

N = 3
N = 17
W = 36
p = 0.289
(r = 0.25)
Corallococcus caeni
ASM3624588v1
Opportunity cost (high respiration)
Auxotrophic
Prototrophic
AA dispensability
Proportion of permutations (%)
high respiration (3 auxotrophic AAs)
p = 0.32
10.0
29.0
29.5
57.0
Average opportunity cost (~P)

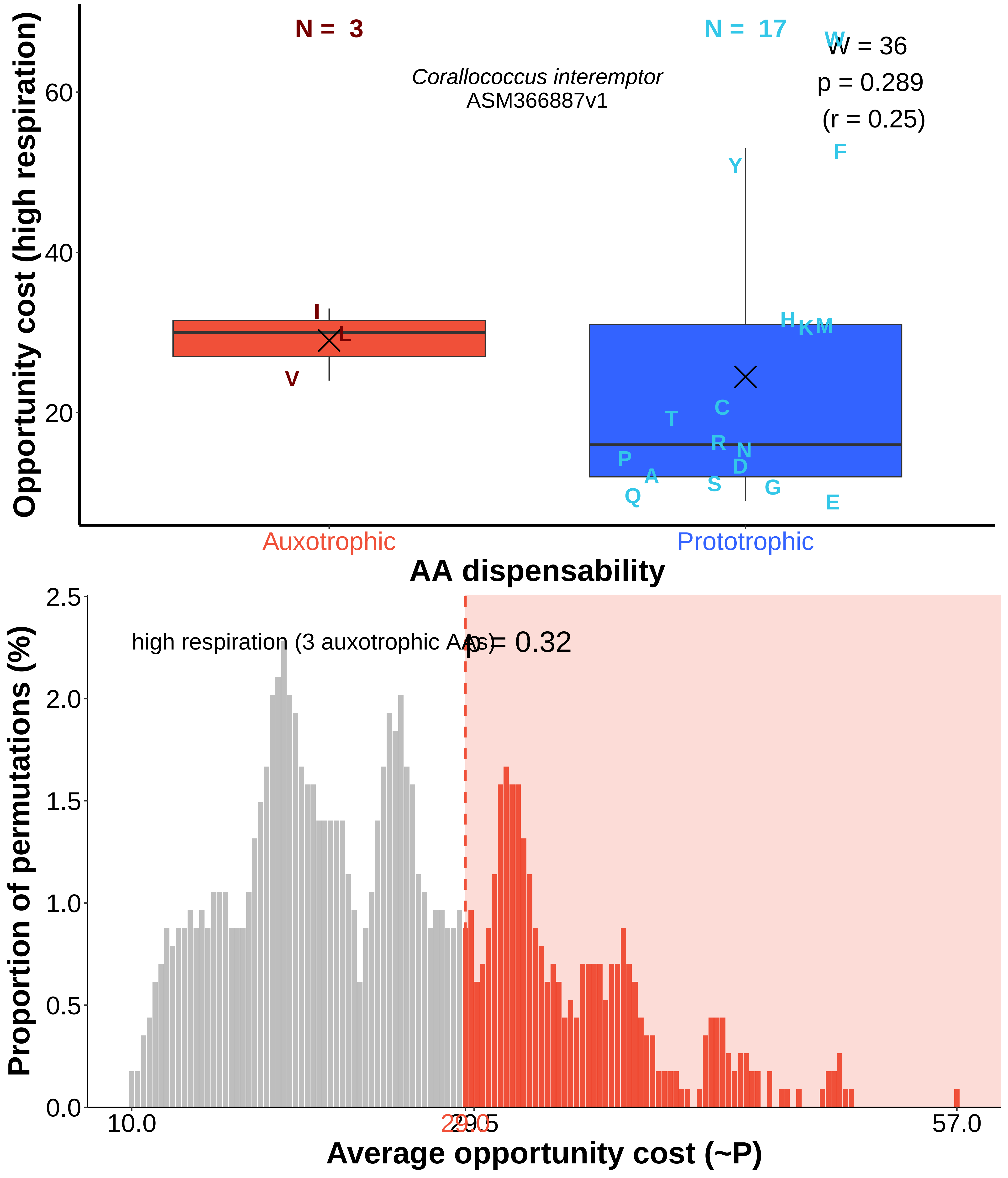
N = 3
N = 17
W = 36
p = 0.289
(r = 0.25)
Corallococcus interemptor
ASM366887v1
Opportunity cost (high respiration)
60
40
20
I
L
V
W
F
Y
H
K
M
C
T
R
N
P
D
A
S
Q
G
E
Auxotrophic
Prototrophic
AA dispensability
high respiration (3 auxotrophic AAs)
p = 0.32
Proportion of permutations (%)
2.5
2.0
1.5
1.0
0.5
0.0
10.0
29.0
29.5
57.0
Average opportunity cost (~P)

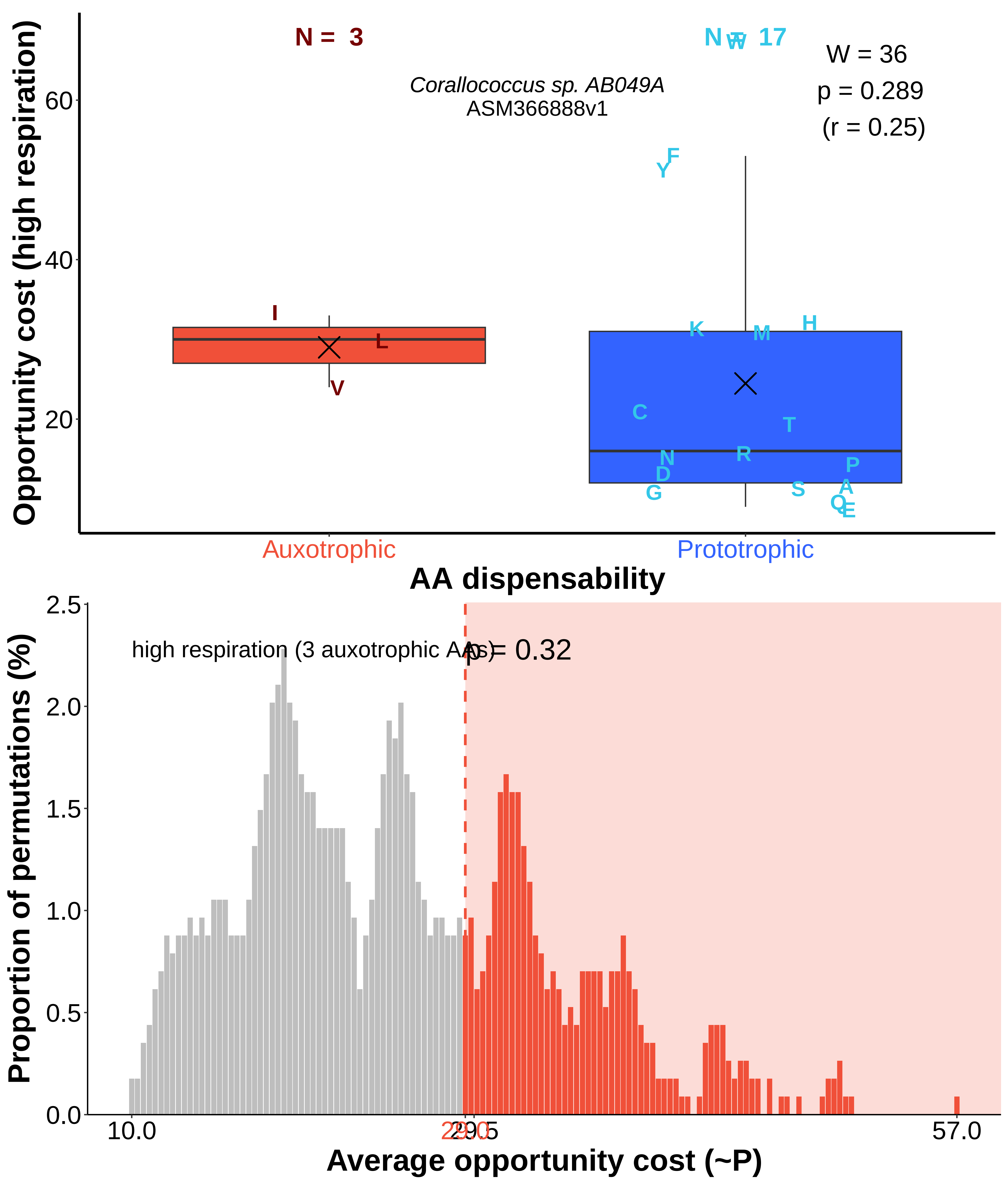
N = 3
N = 17
Corallococcus sp. AB049A
ASM366888v1
W = 36
p = 0.289
(r = 0.25)
Opportunity cost (high respiration)
Auxotrophic
Prototrophic
AA dispensability
high respiration (3 auxotrophic AAs)
p = 0.32
Proportion of permutations (%)
Average opportunity cost (~P)
10.0
29.0
57.0

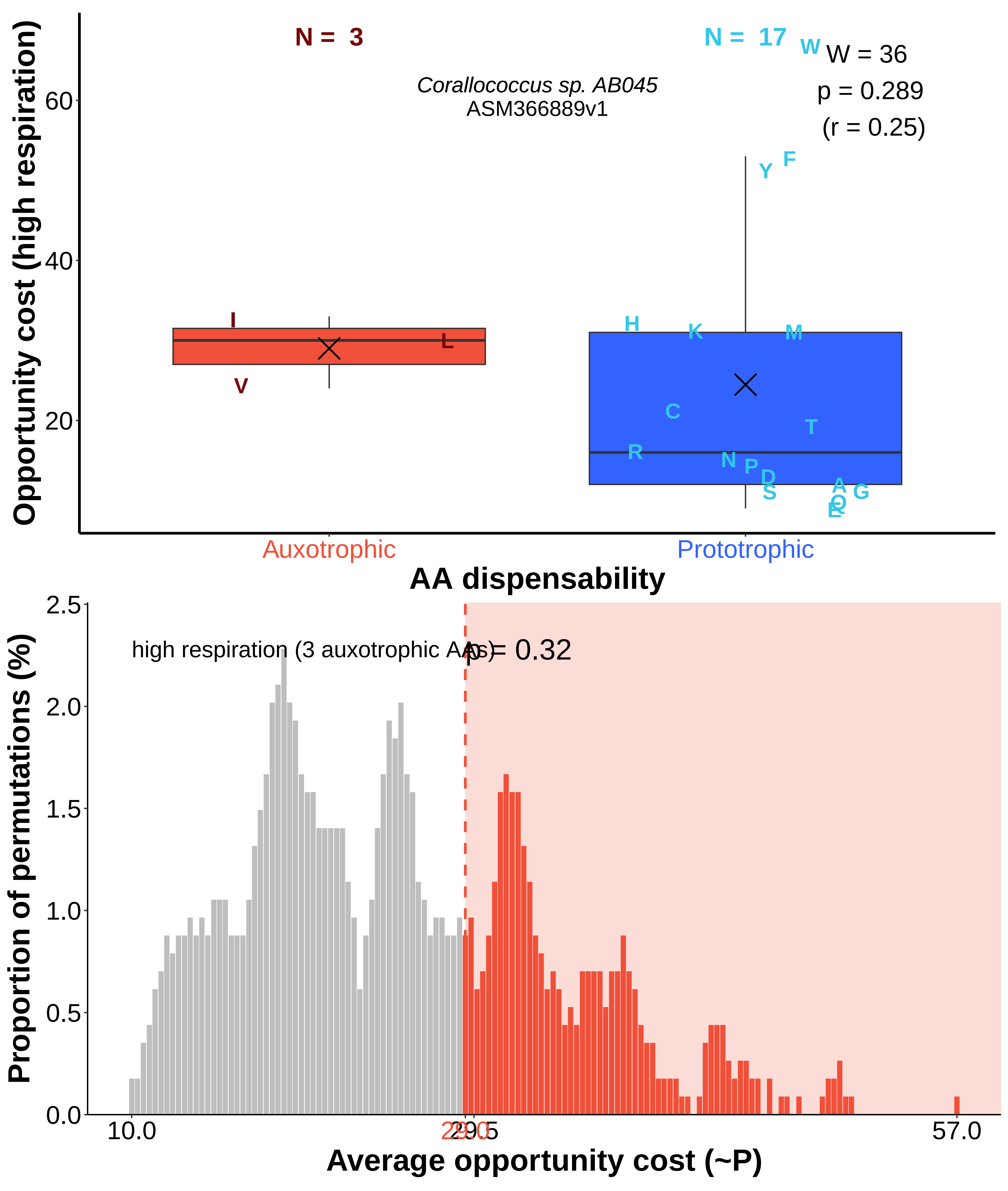

N = 3
N = 17
W = 36
p = 0.289
(r = 0.25)
Corallococcus sp. AB045
ASM366889v1
Opportunity cost (high respiration)
60
40
20
I
L
V
W
Y
F
H
K
M
C
T
R
N
P
D
S
A
G
Q
E
Auxotrophic
Prototrophic
AA dispensability
high respiration (3 auxotrophic AAs)
p = 0.32
Proportion of permutations (%)
2.5
2.0
1.5
1.0
0.5
0.0
10.0
29.0
29.5
57.0
Average opportunity cost (~P)

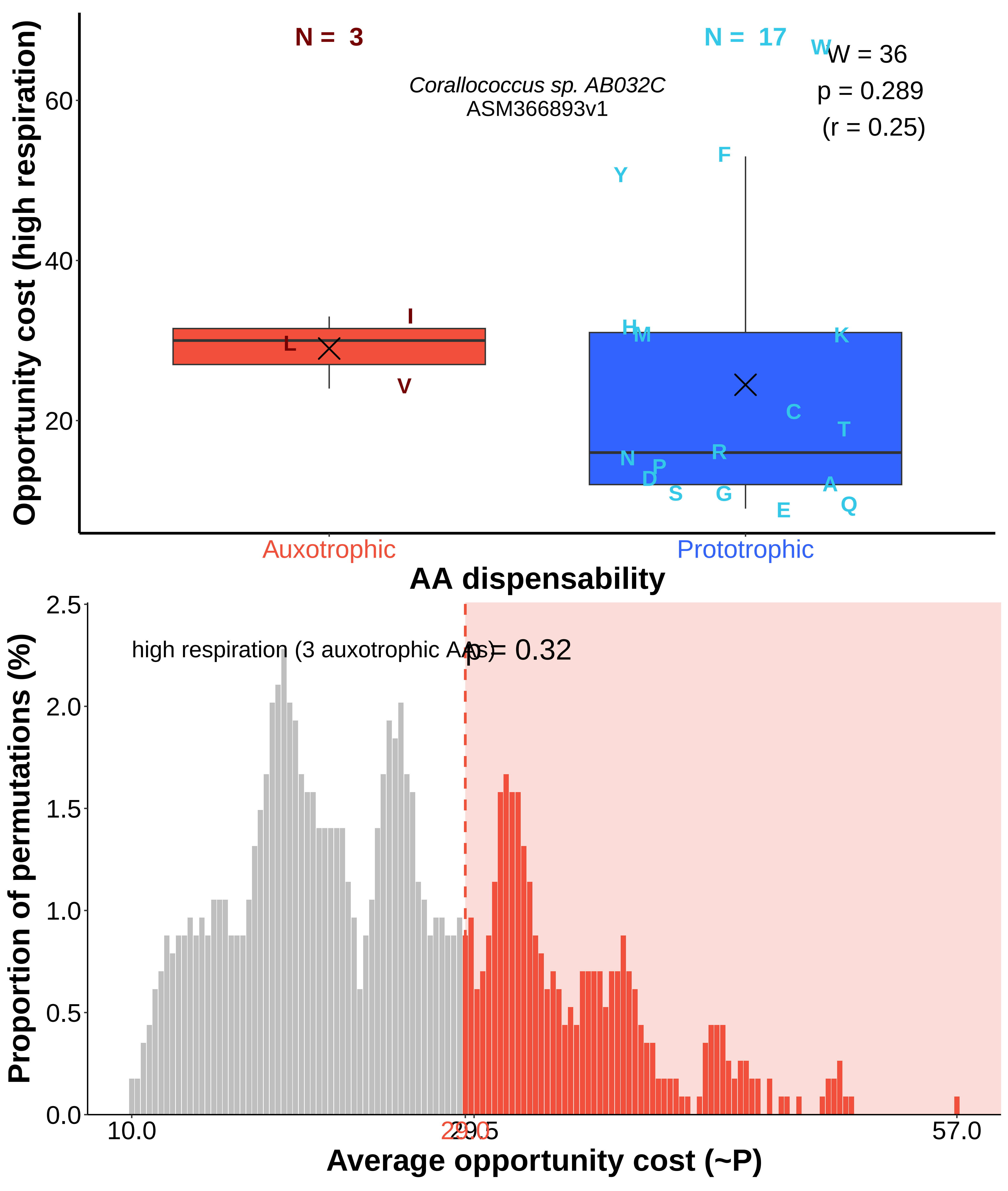

N = 3
N = 17
Corallococcus sp. AB032C
ASM366893v1
W = 36
p = 0.289
(r = 0.25)
Opportunity cost (high respiration)
Auxotrophic
Prototrophic
AA dispensability
high respiration (3 auxotrophic AAs)
p = 0.32
Proportion of permutations (%)
Average opportunity cost (~P)

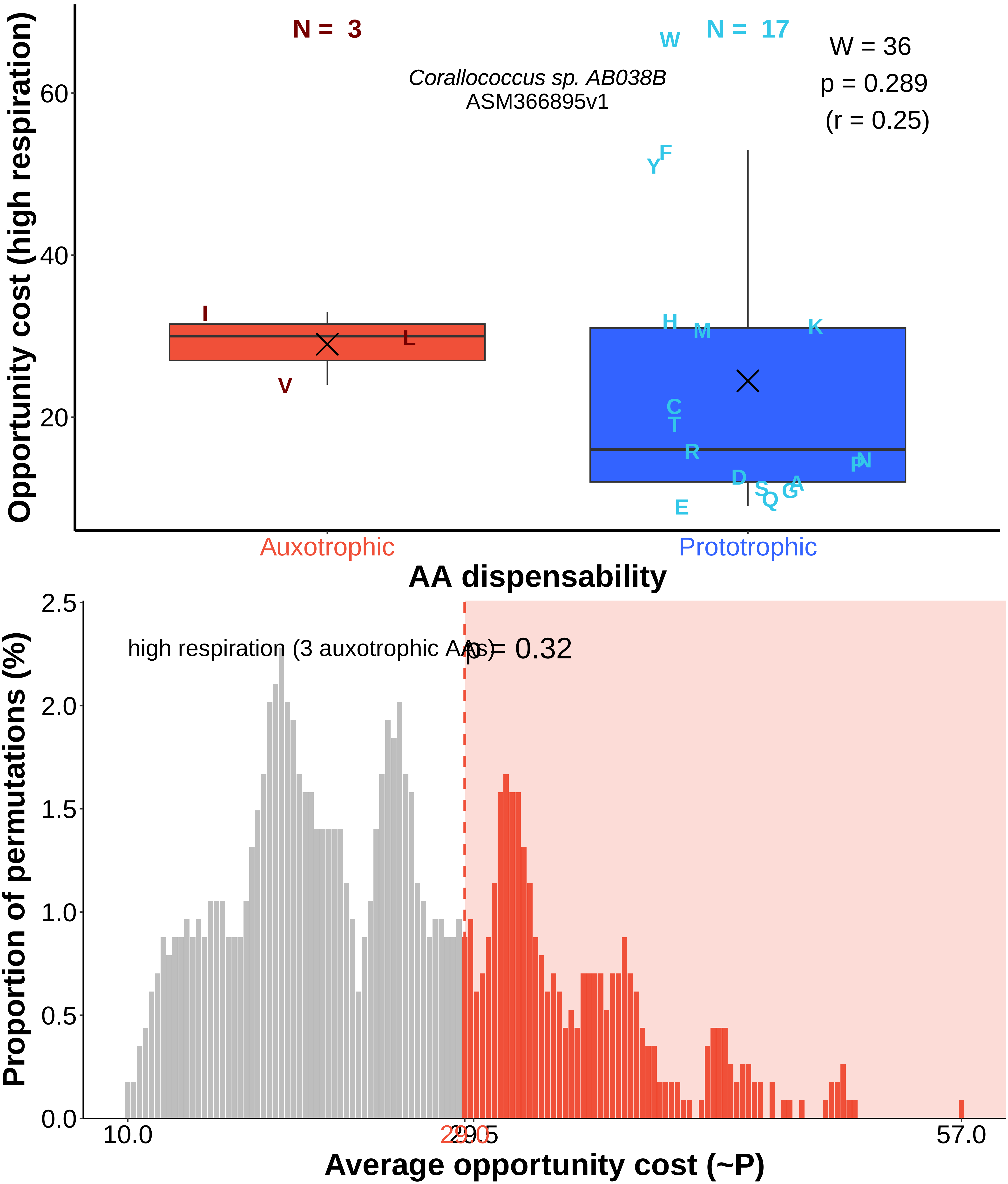
N = 3
N = 17
W = 36
p = 0.289
(r = 0.25)
Corallococcus sp. AB038B
ASM366895v1
Opportunity cost (high respiration)
60
40
20
I
L
V
W
F
Y
H
M
K
C
T
R
P
N
D
S
Q
G
A
E
Auxotrophic
Prototrophic
AA dispensability
high respiration (3 auxotrophic AAs)
p = 0.32
Proportion of permutations (%)
2.5
2.0
1.5
1.0
0.5
0.0
10.0
29.0
29.5
57.0
Average opportunity cost (~P)

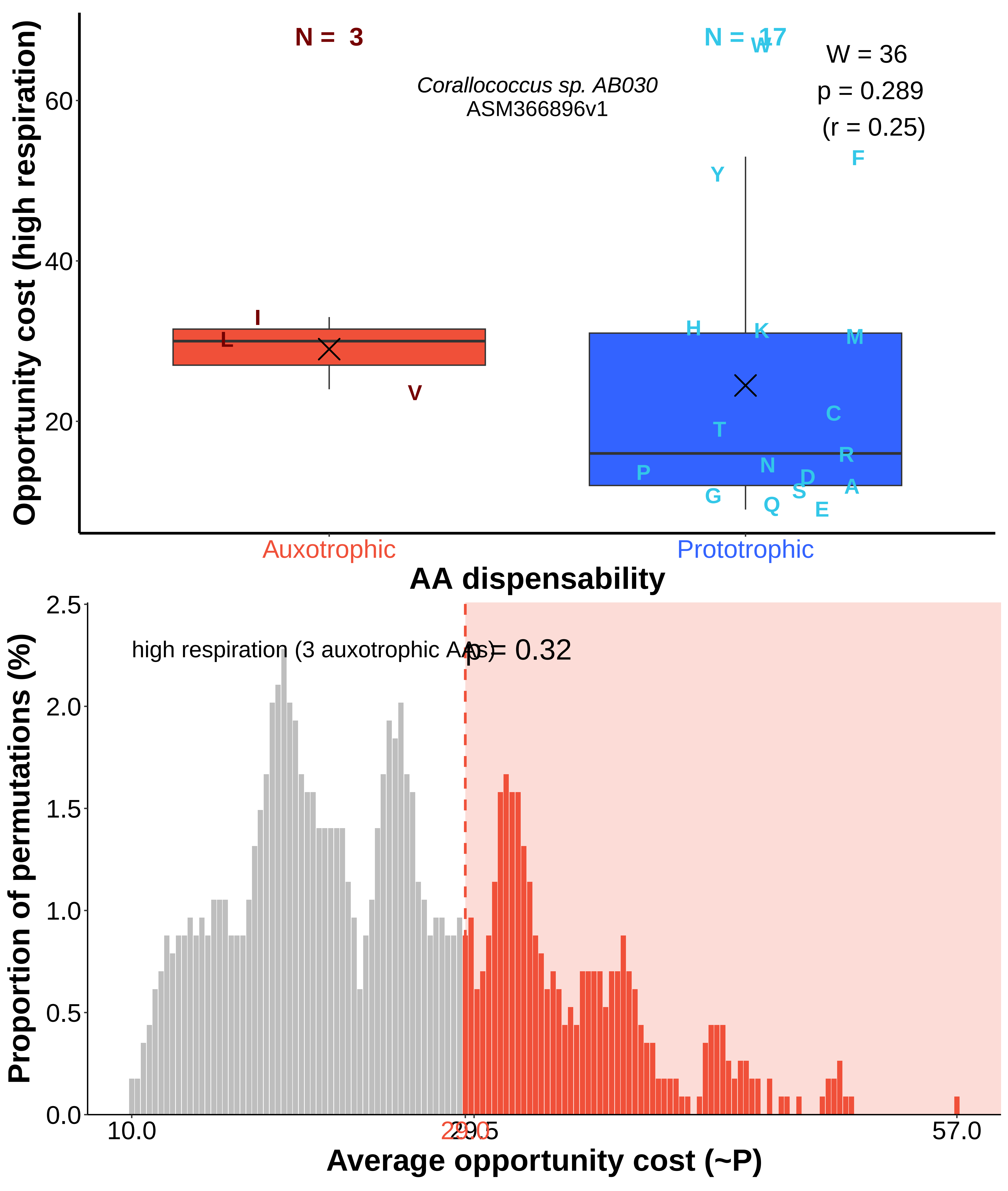
N = 3
N = 17
Corallococcus sp. AB030
ASM366896v1
W = 36
p = 0.289
(r = 0.25)
Opportunity cost (high respiration)
60
40
20
I
L
V
W
F
Y
H
K
M
C
T
R
P
N
D
S
A
G
Q
E
Auxotrophic
Prototrophic
AA dispensability
high respiration (3 auxotrophic AAs)
p = 0.32
Proportion of permutations (%)
2.5
2.0
1.5
1.0
0.5
0.0
10.0
29.0
29.5
57.0
Average opportunity cost (~P)

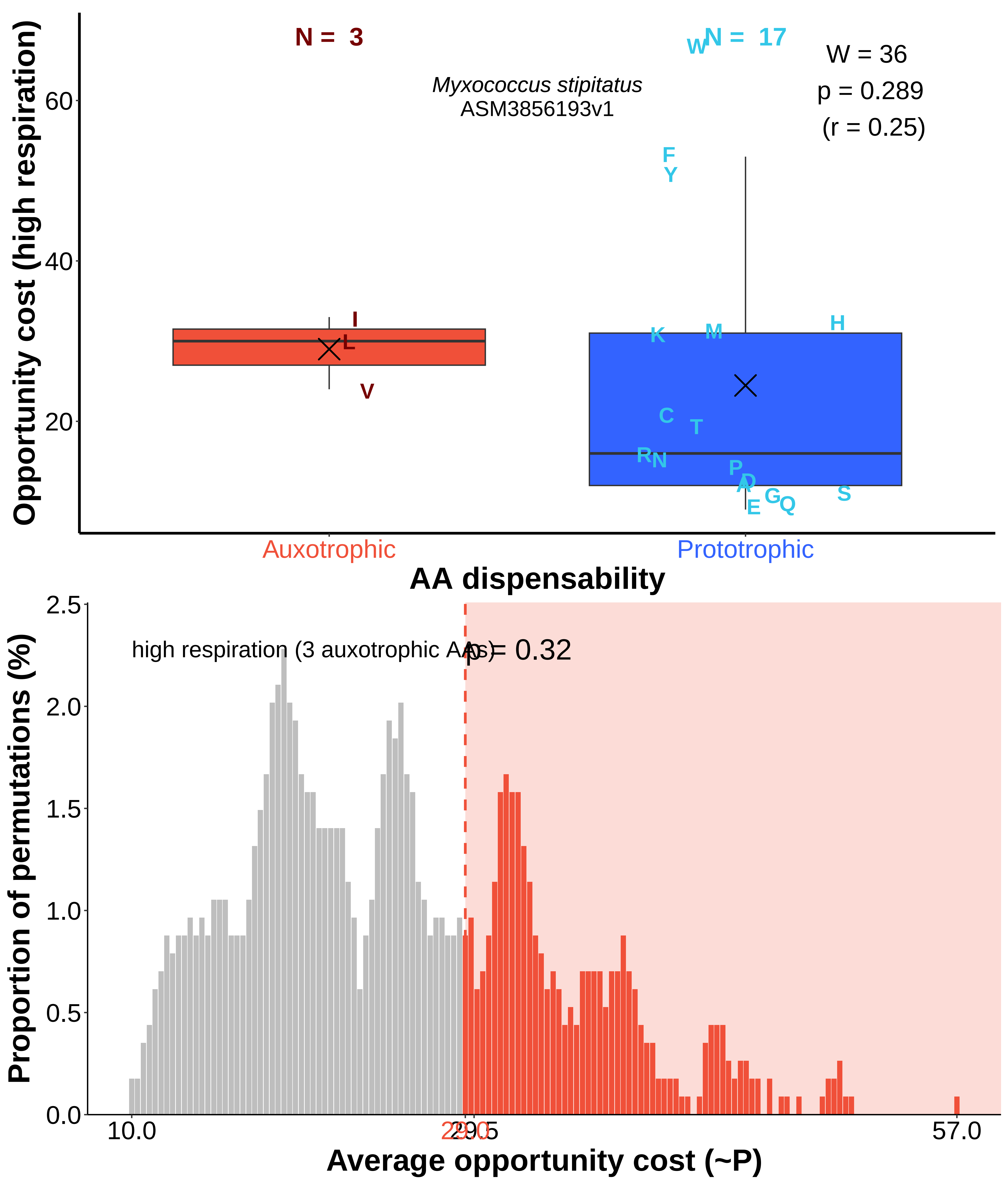
N = 3
N = 17
W
Myxococcus stipitatus
ASM3856193v1
W = 36
p = 0.289
(r = 0.25)
Opportunity cost (high respiration)
60
40
20
F
Y
I
L
V
K
M
H
C
T
R
N
P
D
A
G
E
Q
S
Auxotrophic
Prototrophic
AA dispensability
Proportion of permutations (%)
2.5
2.0
1.5
1.0
0.5
0.0
high respiration (3 auxotrophic AAs)
p = 0.32
10.0
29.0
29.5
57.0
Average opportunity cost (~P)

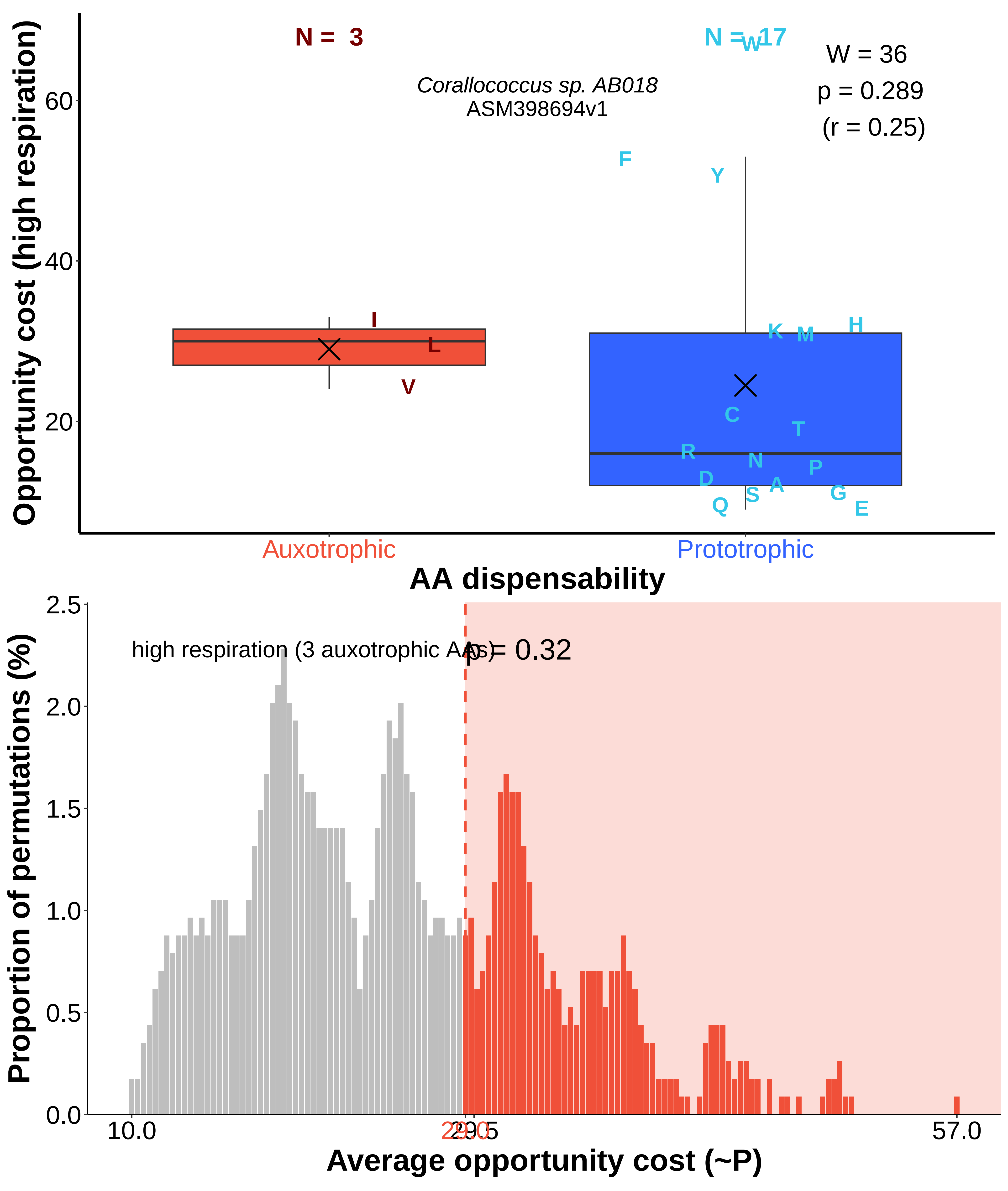

N = 3
N = 17
W
Corallococcus sp. AB018
ASM398694v1
W = 36
p = 0.289
(r = 0.25)
Opportunity cost (high respiration)
60
40
20
F
Y
I
L
V
K
M
H
C
T
R
N
P
D
A
S
G
Q
E
Auxotrophic
Prototrophic
AA dispensability
high respiration (3 auxotrophic AAs)
p = 0.32
Proportion of permutations (%)
2.5
2.0
1.5
1.0
0.5
0.0
10.0
29.0
29.5
57.0
Average opportunity cost (~P)

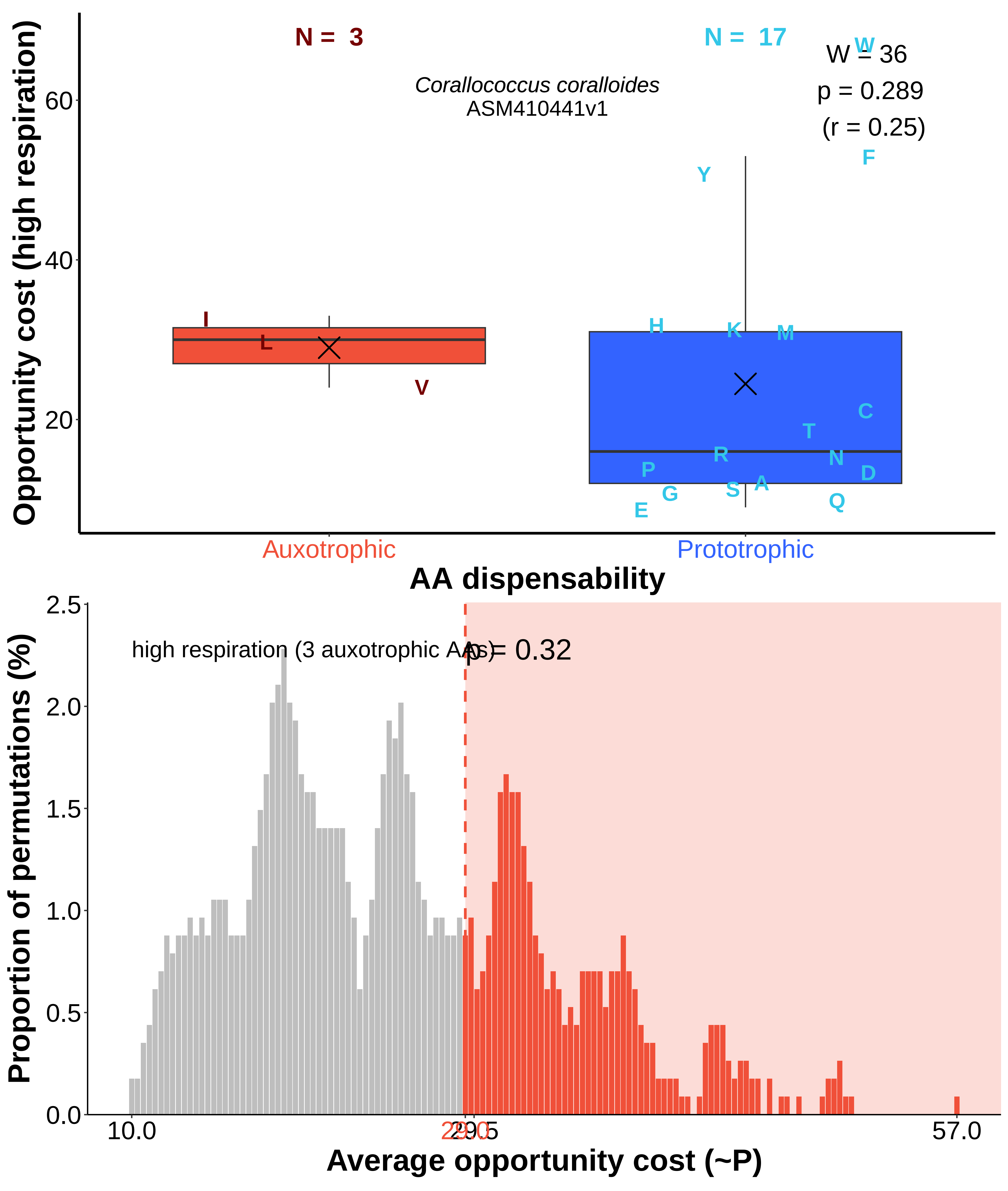
N = 3
N = 17
Corallococcus coralloides
ASM410441v1
W = 36
p = 0.289
(r = 0.25)
Opportunity cost (high respiration)
60
40
20
I
L
V
Y
F
W
H
K
M
C
T
R
N
P
D
A
S
G
Q
E
Auxotrophic
Prototrophic
AA dispensability
high respiration (3 auxotrophic AAs)
p = 0.32
Proportion of permutations (%)
2.5
2.0
1.5
1.0
0.5
0.0
10.0
29.0
29.5
57.0
Average opportunity cost (~P)

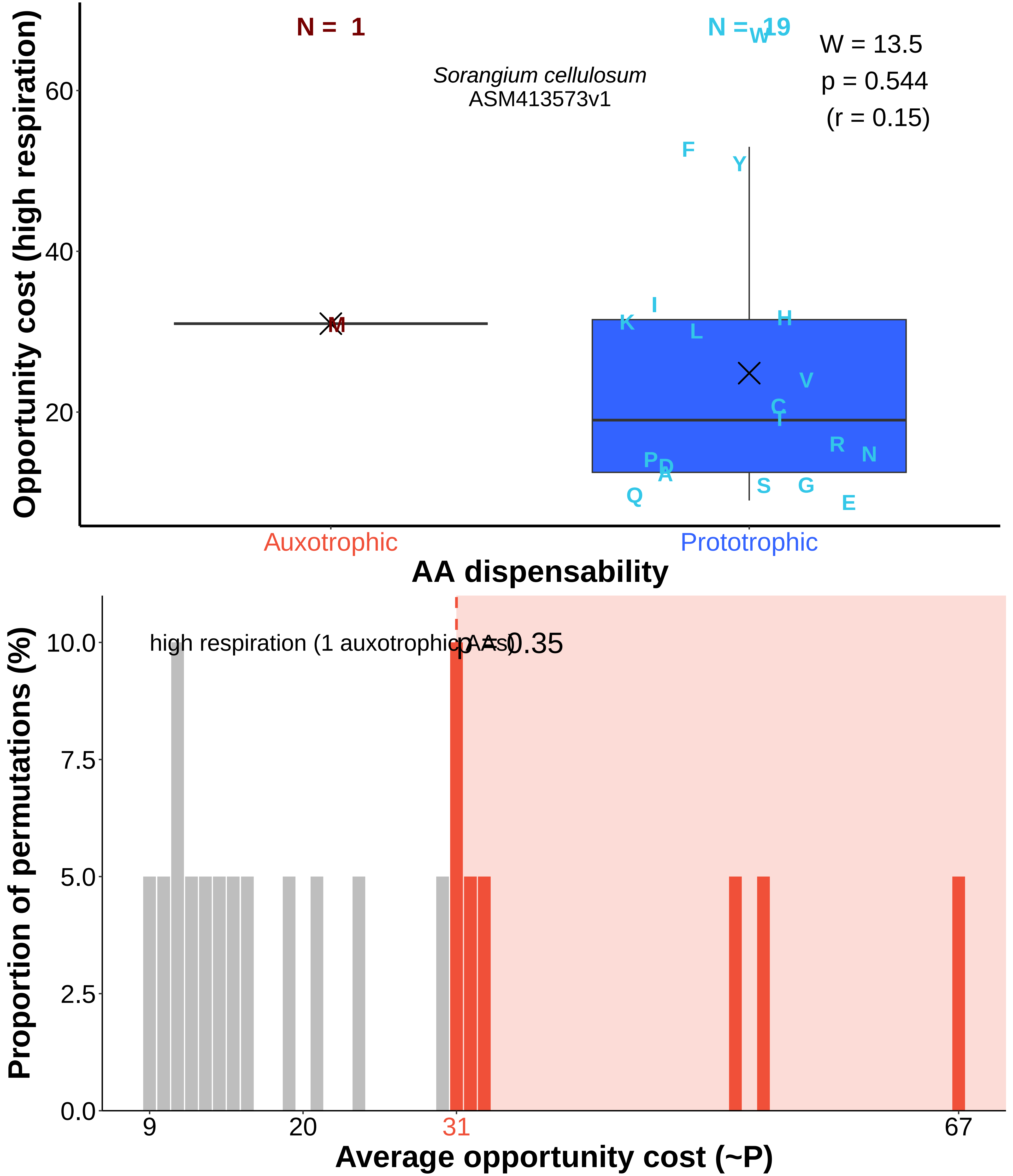

N = 1
N = 19
W = 13.5
p = 0.544
(r = 0.15)
Sorangium cellulosum
ASM413573v1
Opportunity cost (high respiration)
60
40
20
M
F
Y
W
I
K
L
H
V
C
T
R
N
P
D
A
Q
S
G
E
Auxotrophic
Prototrophic
AA dispensability
high respiration (1 auxotrophic AAs)
p = 0.35
Proportion of permutations (%)
10.0
7.5
5.0
2.5
0.0
9
20
31
67
Average opportunity cost (~P)

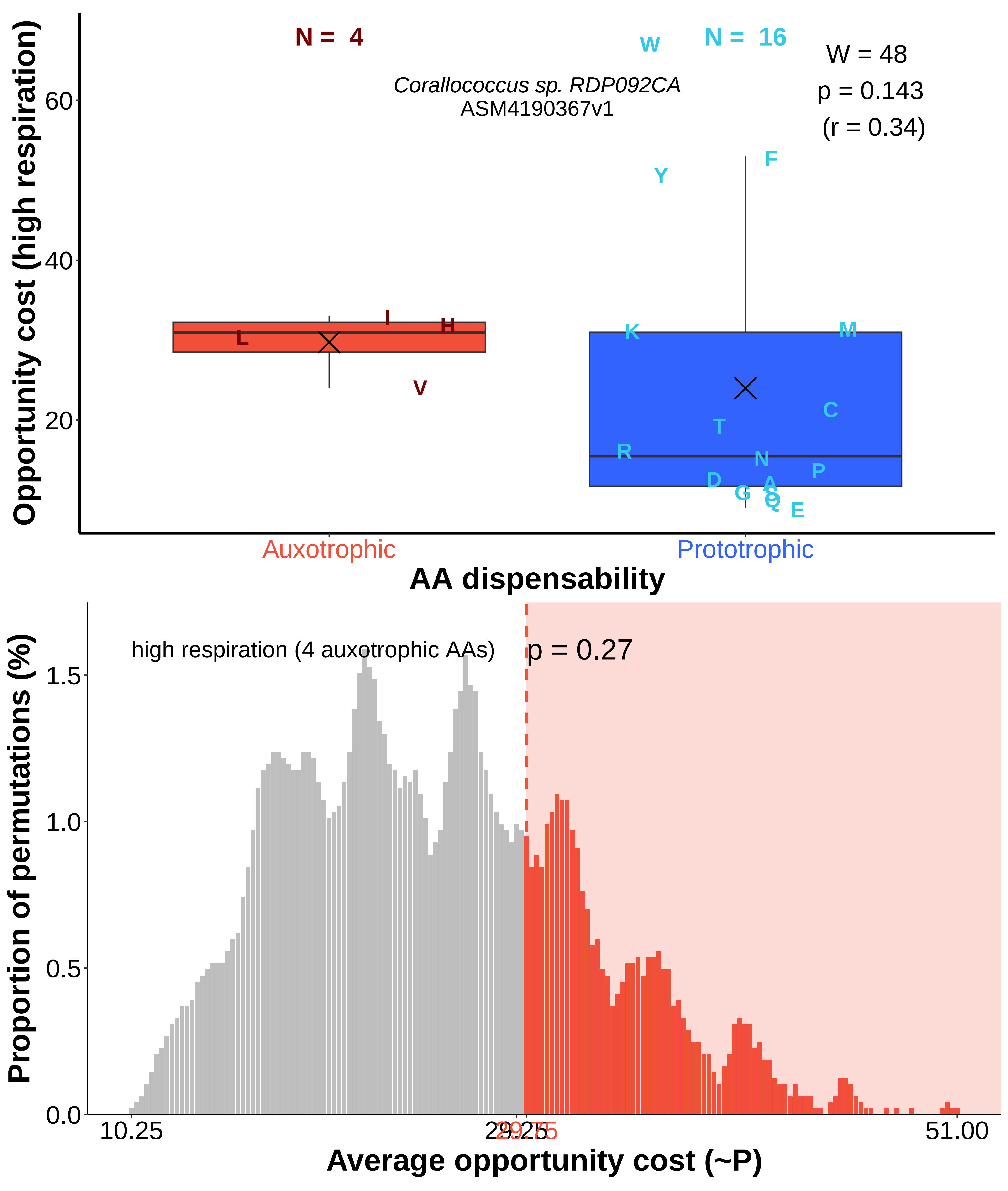

N = 4
N = 16
W
Corallococcus sp. RDP092CA
ASM4190367v1
W = 48
p = 0.143
(r = 0.34)
Opportunity cost (high respiration)
60
40
20
F
Y
I
H
L
V
K
M
C
T
R
N
P
D
A
G
S
Q
E
Auxotrophic
Prototrophic
AA dispensability
high respiration (4 auxotrophic AAs)
p = 0.27
Proportion of permutations (%)
1.5
1.0
0.5
0.0
10.25
29.75
51.00
Average opportunity cost (~P)

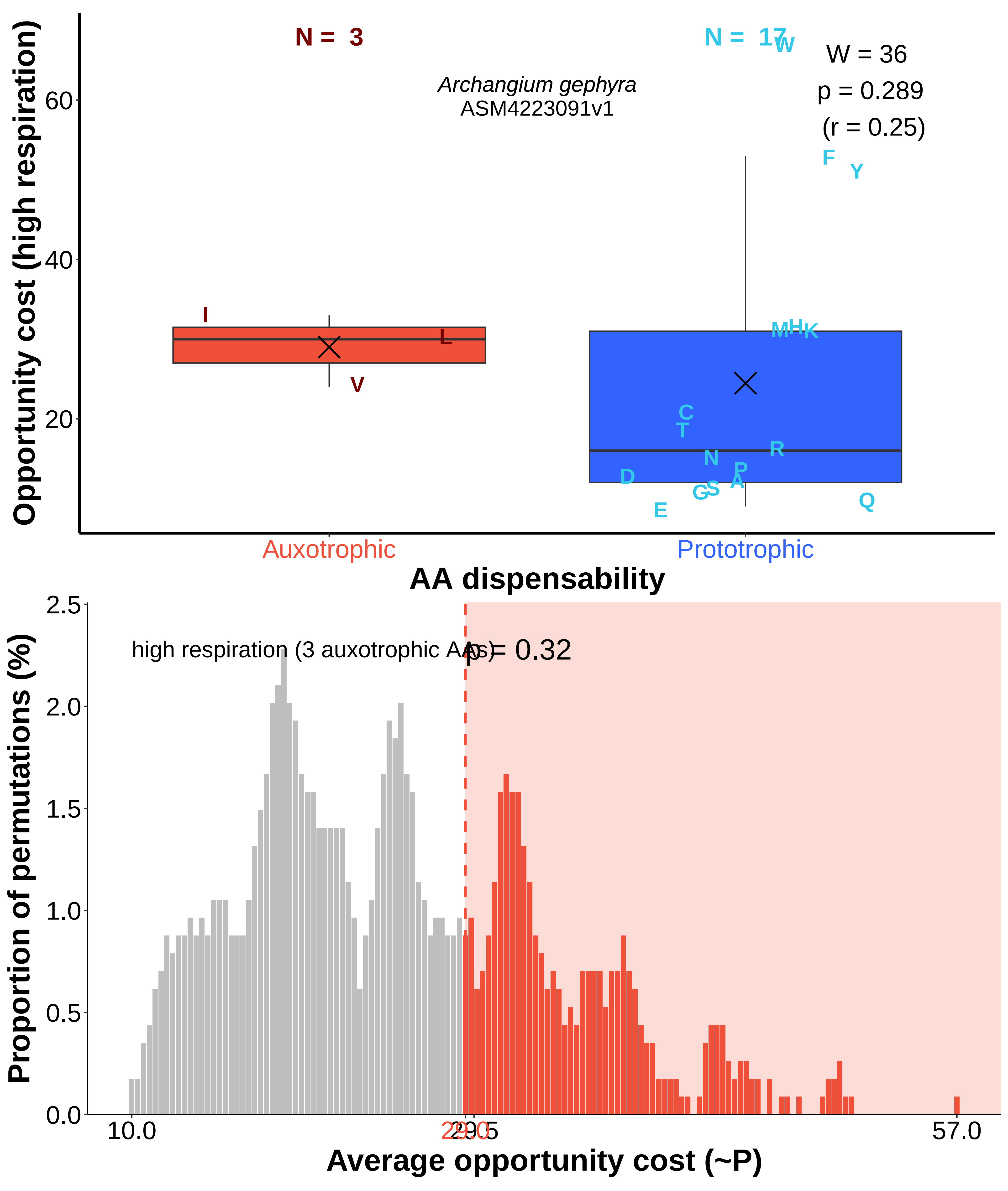

N = 3
N = 17
Archangium gephyra
ASM4223091v1
W = 36
p = 0.289
(r = 0.25)
Opportunity cost (high respiration)
Auxotrophic
Prototrophic
AA dispensability
high respiration (3 auxotrophic AAs)
p = 0.32
Proportion of permutations (%)
Average opportunity cost (~P)

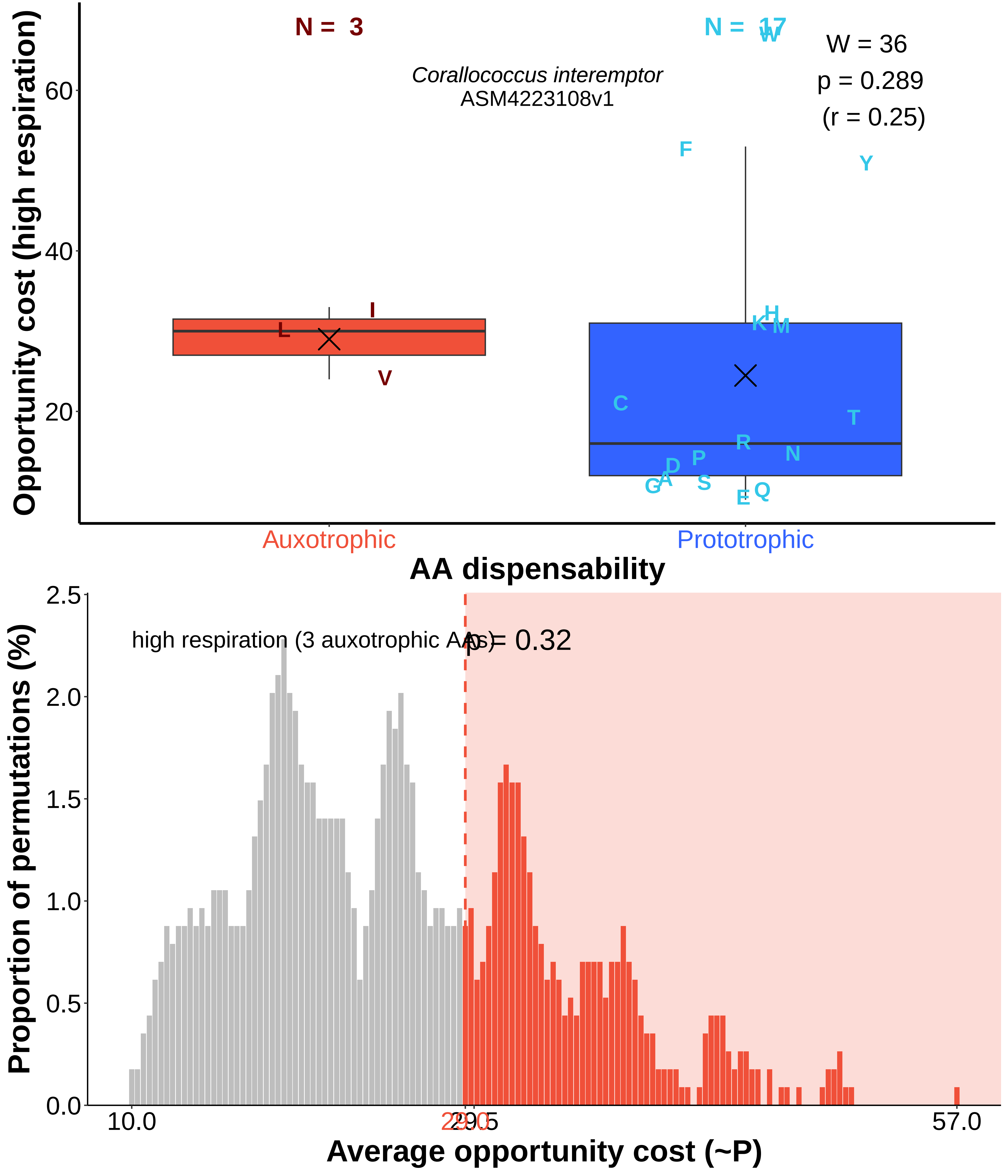

N = 3
N = 17
W = 36
p = 0.289
(r = 0.25)
Corallococcus interemptor
ASM4223108v1
Opportunity cost (high respiration)
Auxotrophic
Prototrophic
AA dispensability
Proportion of permutations (%)
high respiration (3 auxotrophic AAs)
p = 0.32
Average opportunity cost (~P)
10.0
29.0
57.0

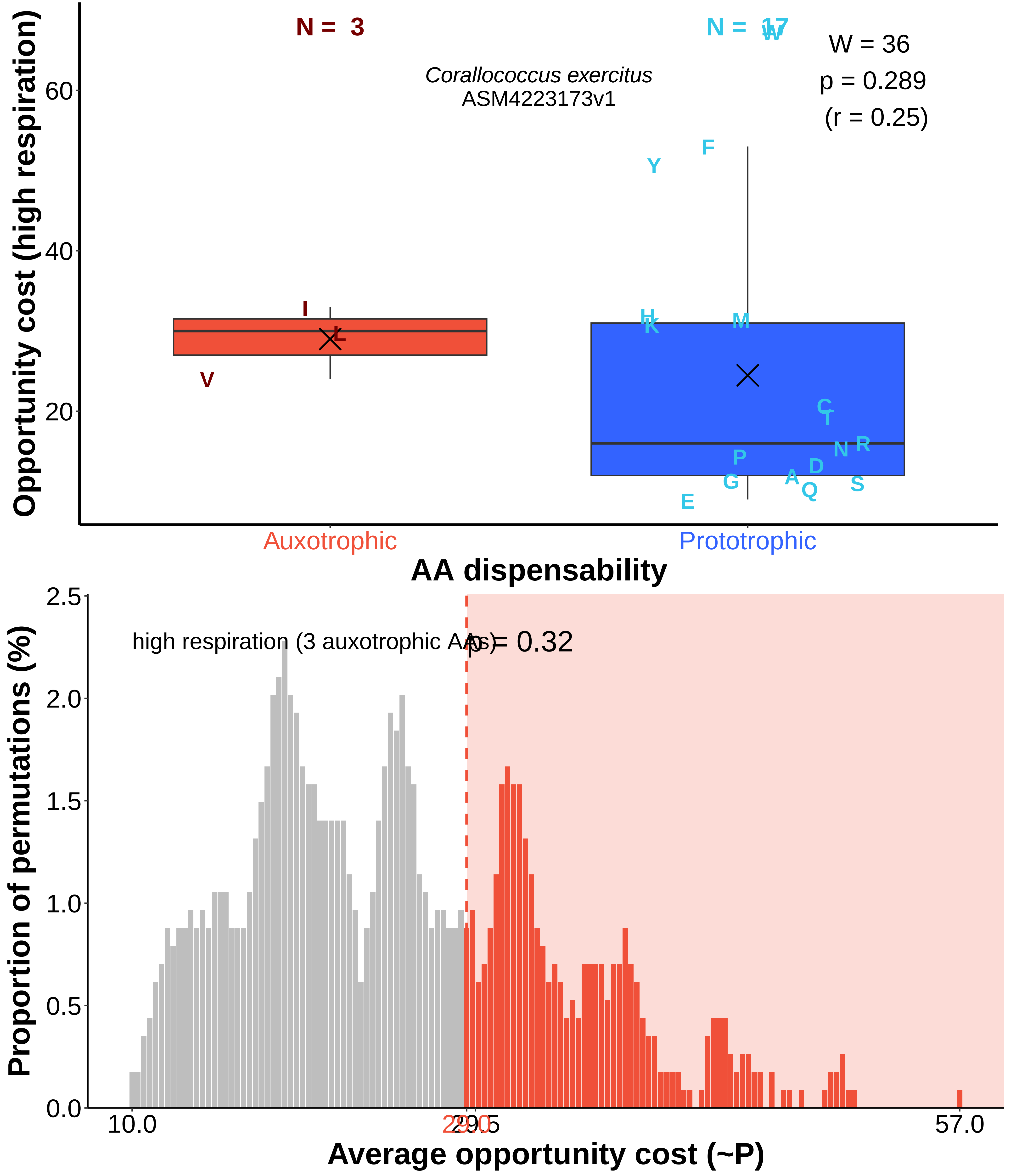

N = 3
N = 17
W = 36
p = 0.289
(r = 0.25)
Corallococcus exercitus
ASM4223173v1
Opportunity cost (high respiration)
60
40
20
I
L
V
Y
F
H
K
M
C
T
N
R
P
D
G
A
Q
S
E
W
Auxotrophic
Prototrophic
AA dispensability
high respiration (3 auxotrophic AAs)
p = 0.32
Proportion of permutations (%)
2.5
2.0
1.5
1.0
0.5
0.0
10.0
29.0
29.5
57.0
Average opportunity cost (~P)

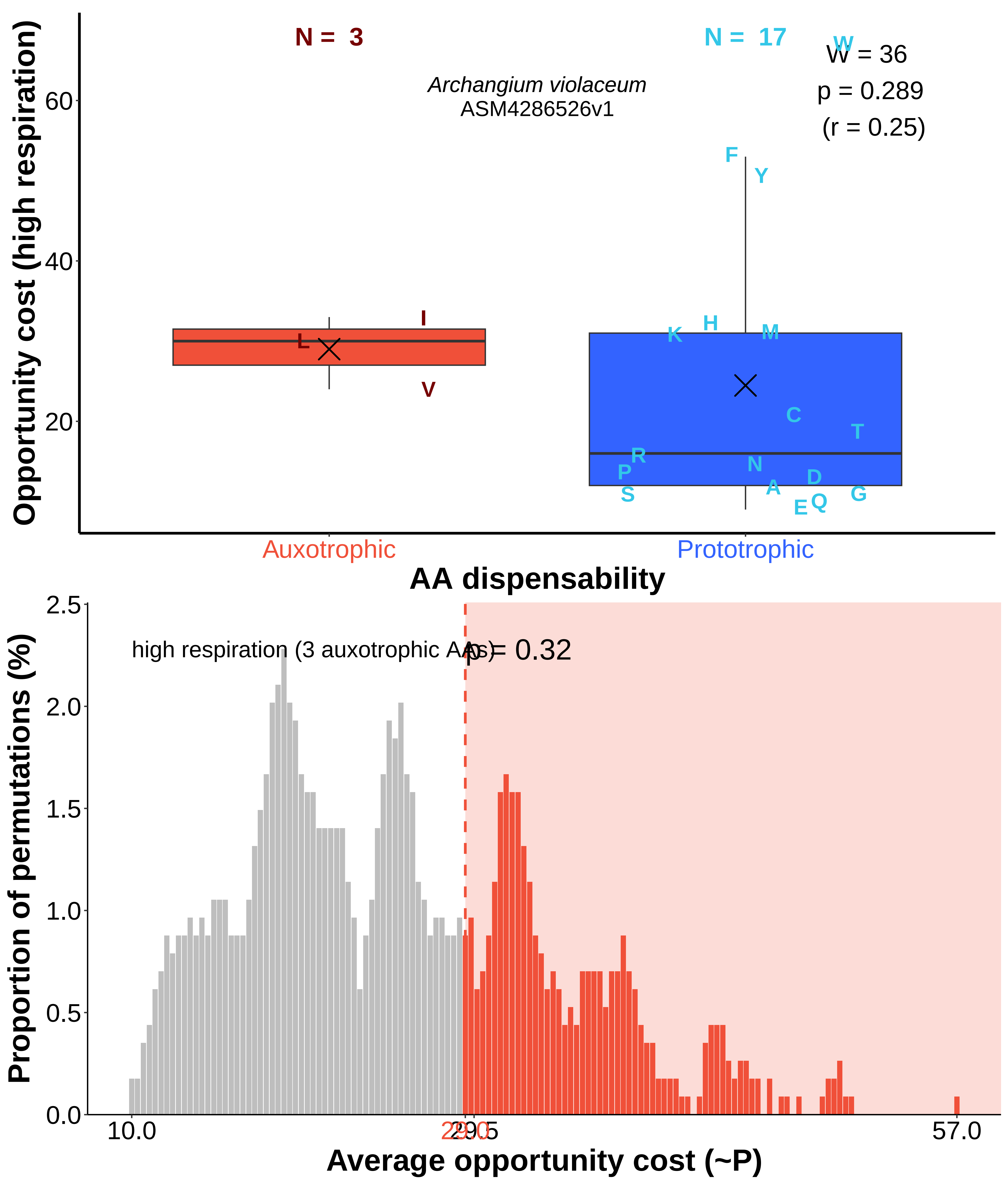

N = 3
N = 17
W = 36
p = 0.289
(r = 0.25)
Archangium violaceum
ASM4286526v1
Opportunity cost (high respiration)
60
40
20
F
Y
I
H
K
M
L
V
C
T
R
N
P
D
A
S
G
E
Q
W
Auxotrophic
Prototrophic
AA dispensability
2.5
2.0
1.5
1.0
0.5
0.0
high respiration (3 auxotrophic AAs)
p = 0.32
Proportion of permutations (%)
10.0
29.0
29.5
57.0
Average opportunity cost (~P)

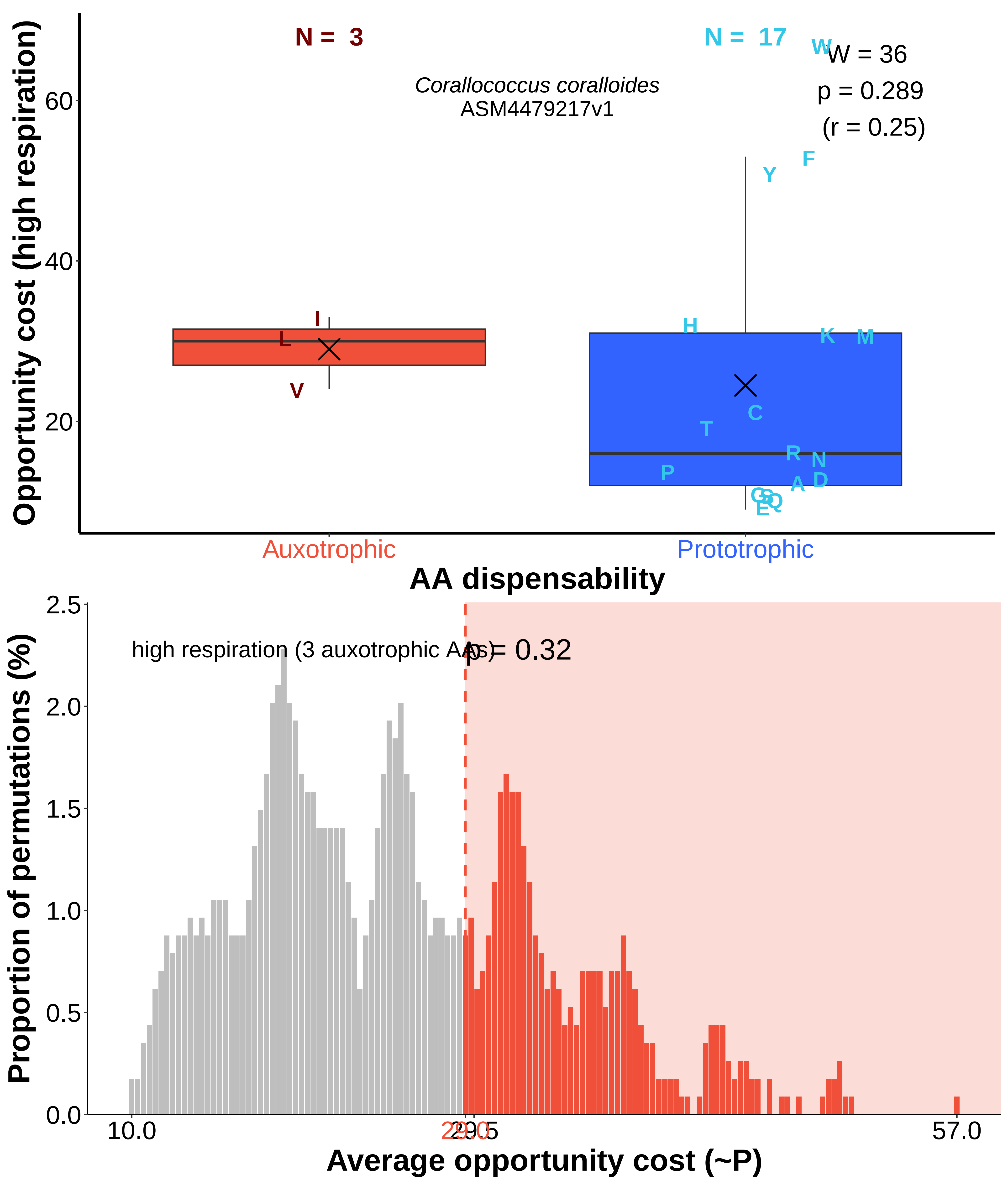

N = 3
N = 17
Corallococcus coralloides
ASM4479217v1
W = 36
p = 0.289
(r = 0.25)
Opportunity cost (high respiration)
Auxotrophic
Prototrophic
AA dispensability
high respiration (3 auxotrophic AAs)
p = 0.32
Proportion of permutations (%)
Average opportunity cost (~P)

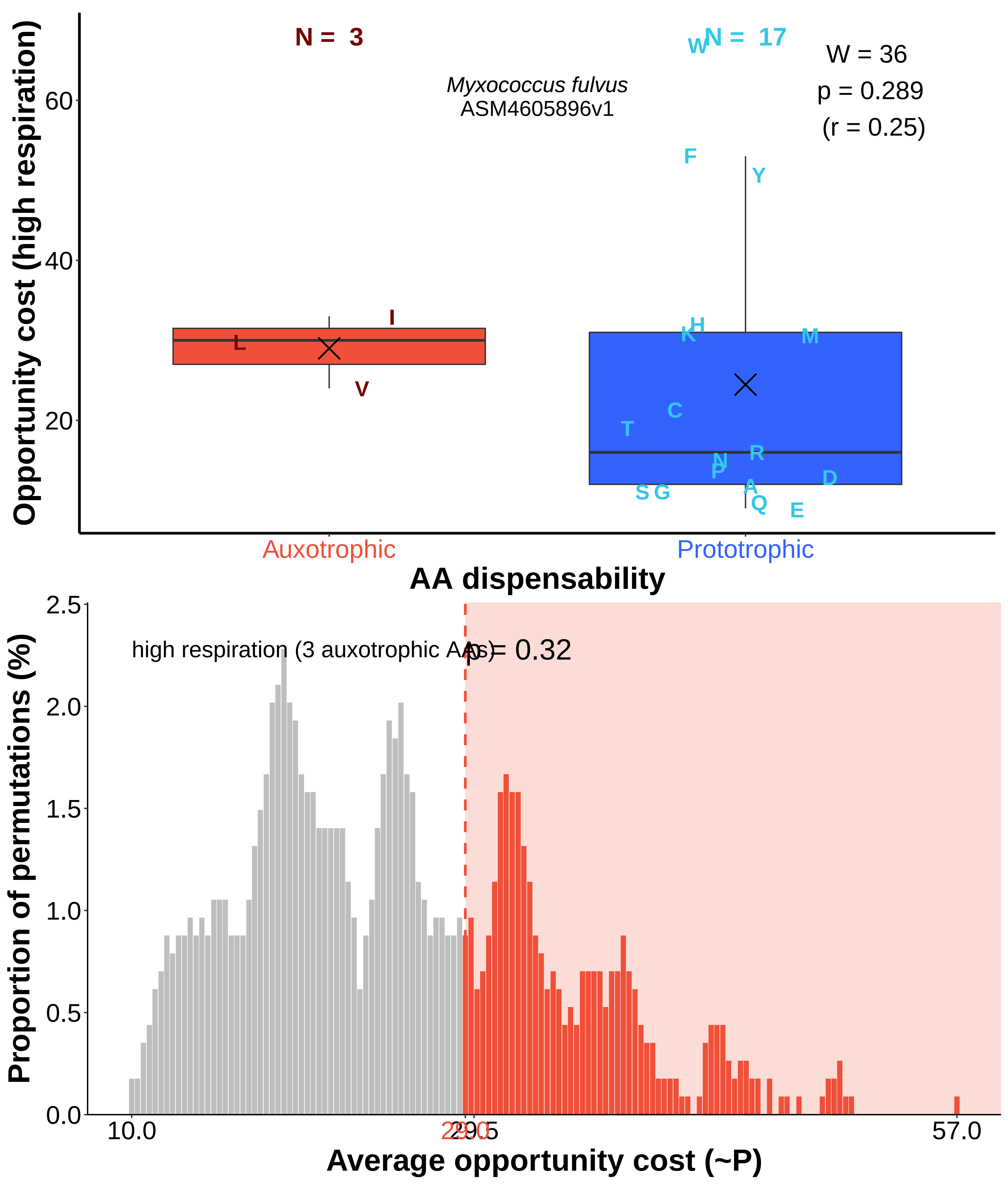

N = 3
N = 17
W
W = 36
p = 0.289
(r = 0.25)
Myxococcus fulvus
ASM4605896v1
F
Y
I
H
K
M
L
V
C
T
R
N
P
D
A
S G
Q
E
60
40
20
Opportunity cost (high respiration)
Auxotrophic
Prototrophic
AA dispensability
high respiration (3 auxotrophic AAs)
p = 0.32
2.5
2.0
1.5
1.0
0.5
0.0
Proportion of permutations (%)
10.0
29.0
29.5
57.0
Average opportunity cost (~P)

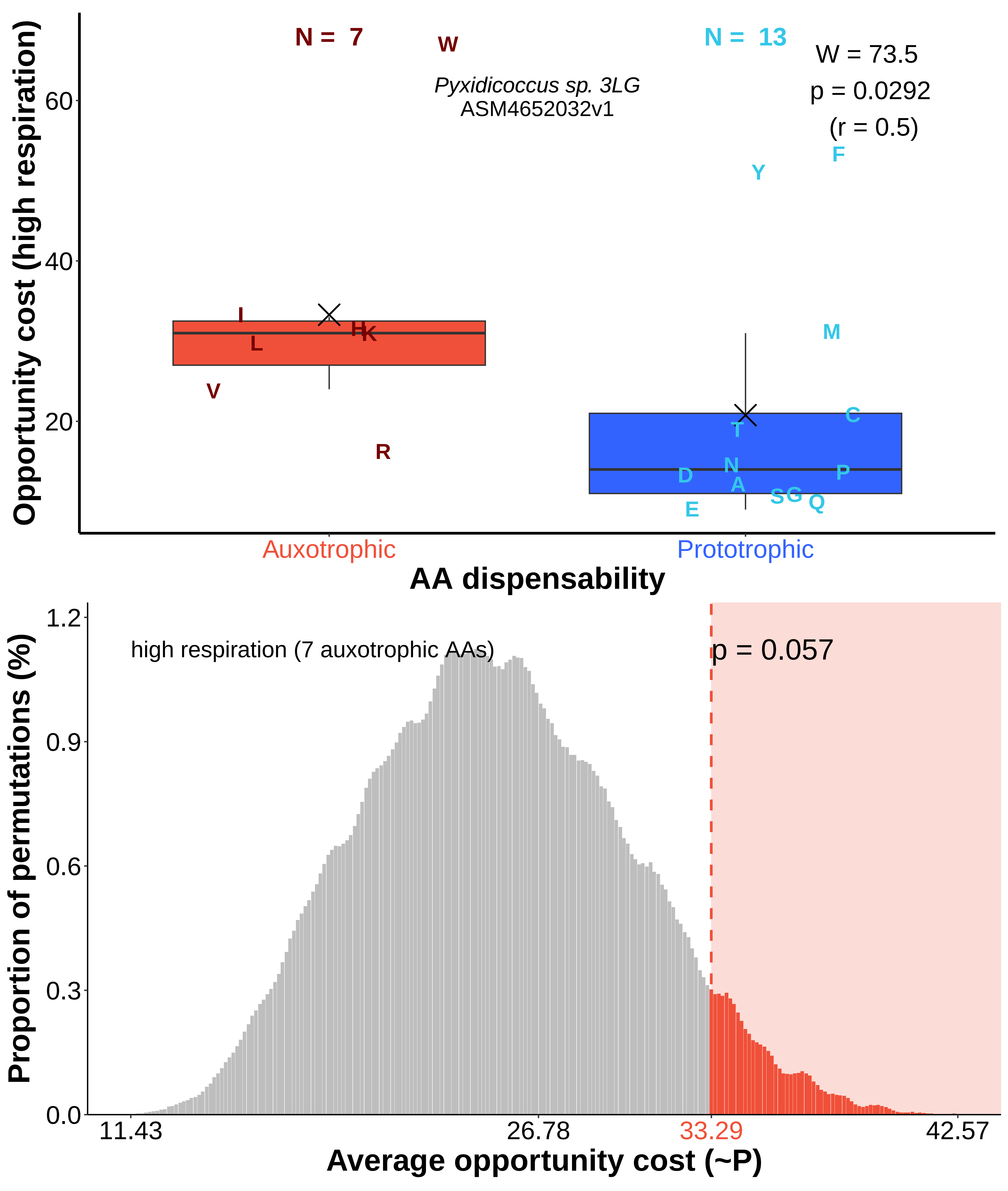
N = 7
W
N = 13
W = 73.5
p = 0.0292
(r = 0.5)
Pyxidicoccus sp. 3LG
ASM4652032v1
Opportunity cost (high respiration)
60
40
20
F
Y
I
H
K
L
V
R
M
C
T
N
D
A
P
S
G
Q
E
Auxotrophic
Prototrophic
AA dispensability
Proportion of permutations (%)
1.2
0.9
0.6
0.3
0.0
high respiration (7 auxotrophic AAs)
p = 0.057
11.43
26.78
33.29
42.57
Average opportunity cost (~P)

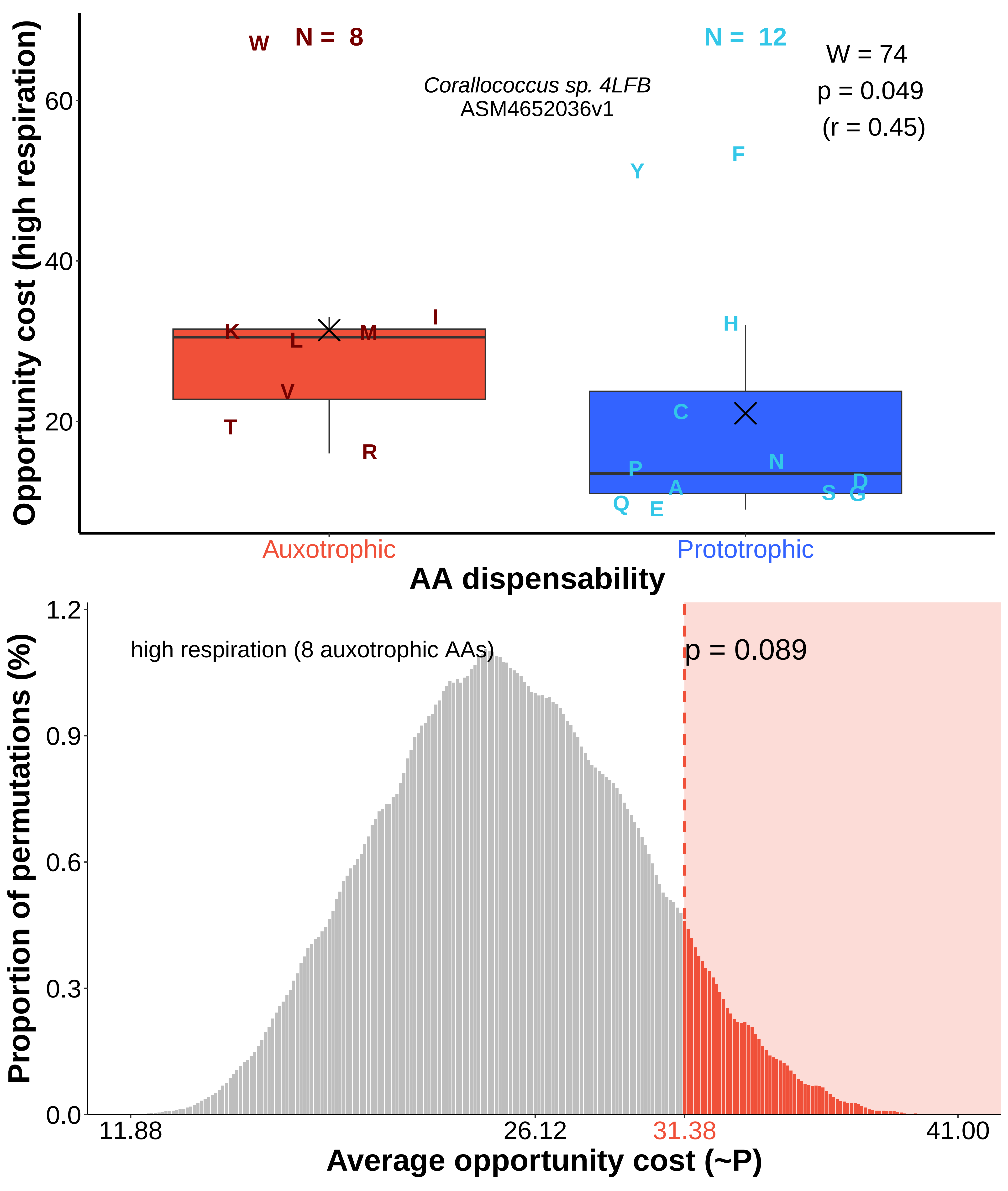

W
N = 8
N = 12
W = 74
p = 0.049
(r = 0.45)
Corallococcus sp. 4LFB
ASM4652036v1
Opportunity cost (high respiration)
60
40
20
F
Y
I
H
K
M
L
V
C
T
R
N
P
D
A
S
G
Q
E
Auxotrophic
Prototrophic
AA dispensability
1.2
0.9
0.6
0.3
0.0
high respiration (8 auxotrophic AAs)
p = 0.089
Proportion of permutations (%)
11.88
26.12
31.38
41.00
Average opportunity cost (~P)

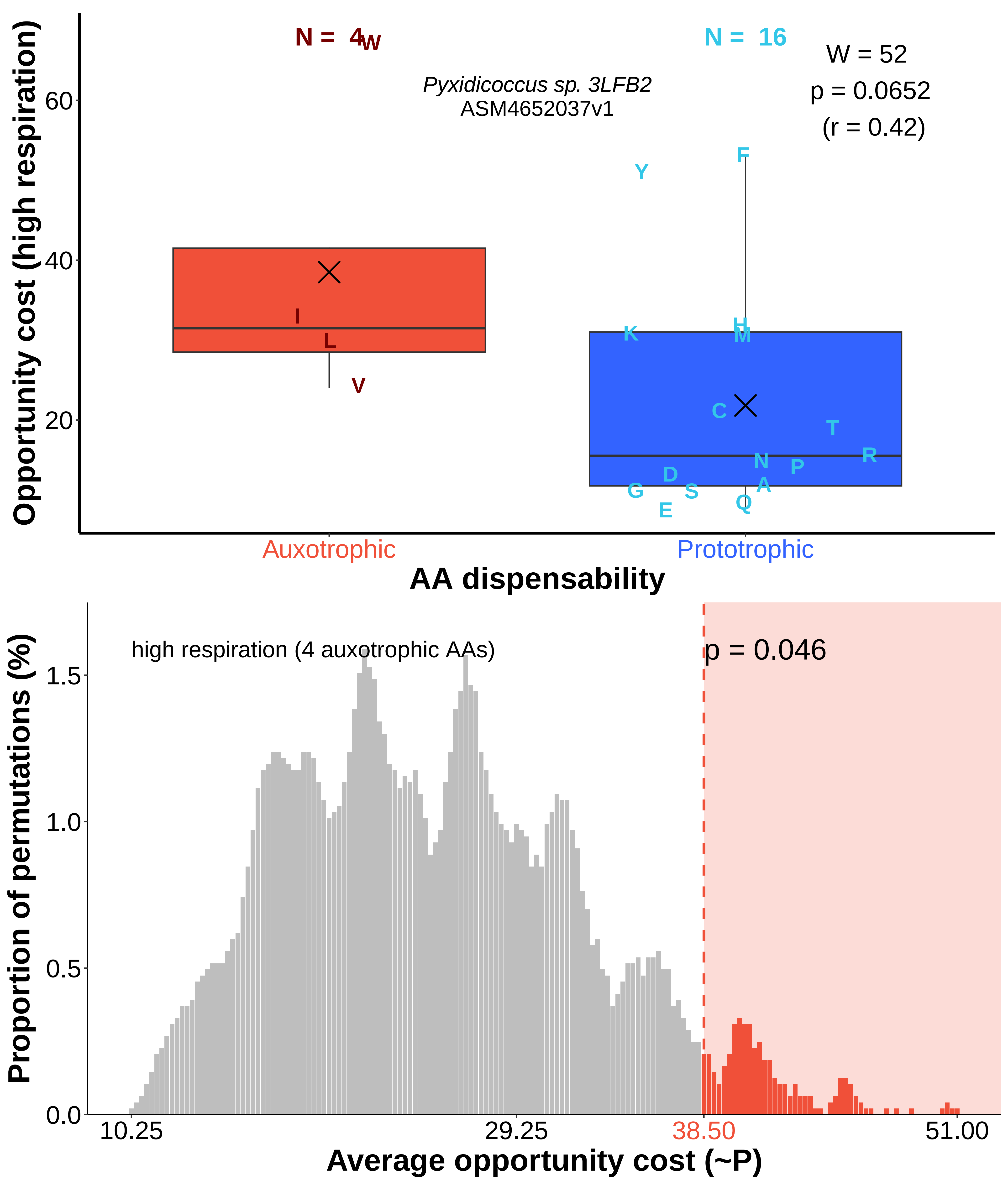
N = 4
W
N = 16
W = 52
p = 0.0652
(r = 0.42)
Pyxidicoccus sp. 3LFB2
ASM4652037v1
Opportunity cost (high respiration)
60
40
20
Y
F
I
L
V
K
H
M
C
T
N
P
R
D
G
S
A
E
Q
Auxotrophic
Prototrophic
AA dispensability
high respiration (4 auxotrophic AAs)
p = 0.046
Proportion of permutations (%)
1.5
1.0
0.5
0.0
10.25
29.25
38.50
51.00
Average opportunity cost (~P)

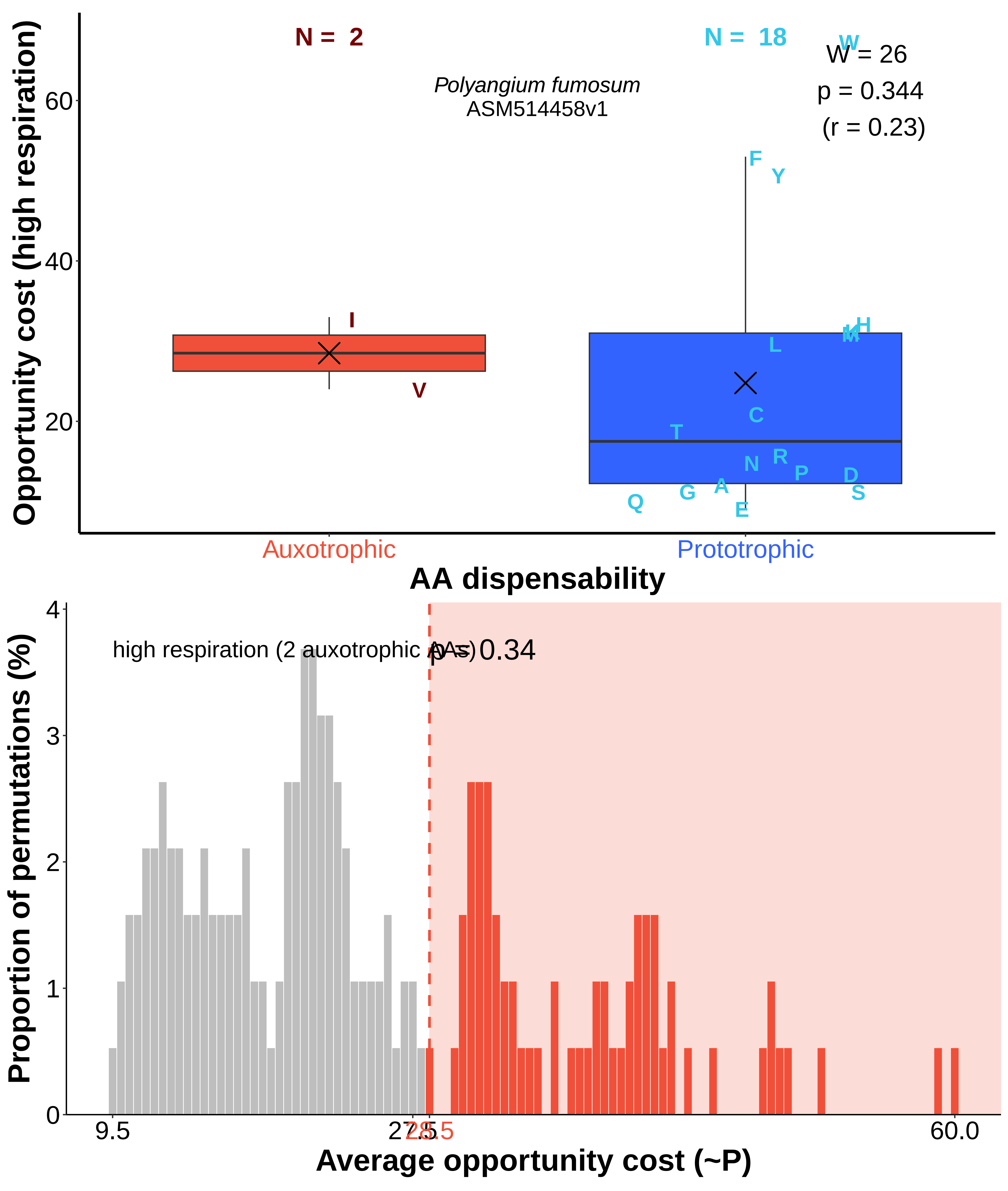

N = 2
N = 18
W = 26
p = 0.344
(r = 0.23)
Polyangium fumosum
ASM514458v1
Opportunity cost (high respiration)
60
40
20
I
V
F
Y
W
H
K
M
L
C
T
R
N
P
D
A
G
S
Q
E
Auxotrophic
Prototrophic
AA dispensability
Proportion of permutations (%)
high respiration (2 auxotrophic AAs)
p = 0.34
4
3
2
1
0
9.5
27.5
28.5
60.0
Average opportunity cost (~P)

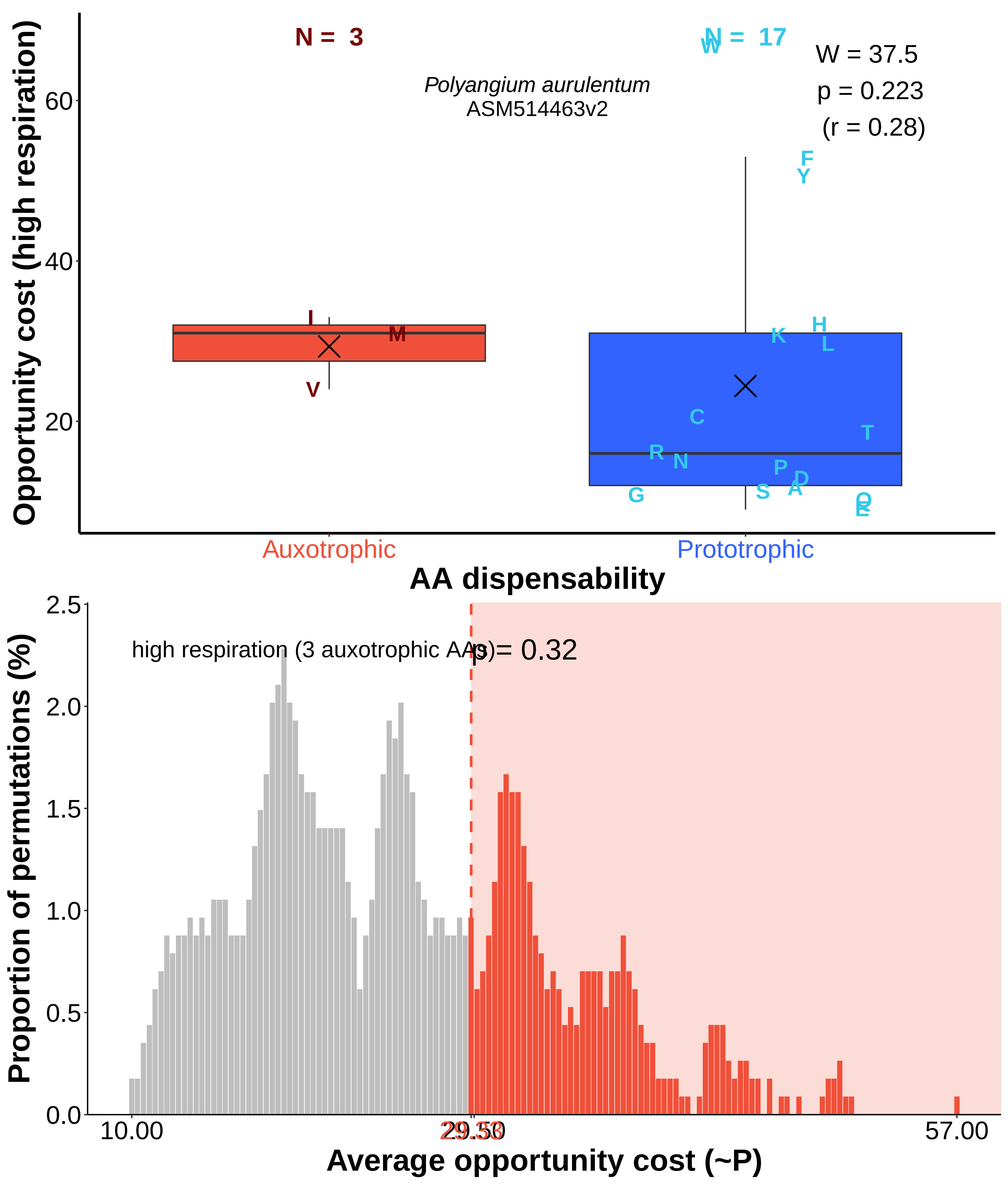
N = 3
N = 17
W
W = 37.5
p = 0.223
(r = 0.28)
Polyangium aurulentum
ASM514463v2
F
Y
I
M
V
K
H
L
C
T
R
N
P
D
A
S
G
Q
E
60
40
20
Opportunity cost (high respiration)
Auxotrophic
Prototrophic
AA dispensability
high respiration (3 auxotrophic AAs)
p = 0.32
2.5
2.0
1.5
1.0
0.5
0.0
Proportion of permutations (%)
10.00
29.50
29.33
57.00
Average opportunity cost (~P)

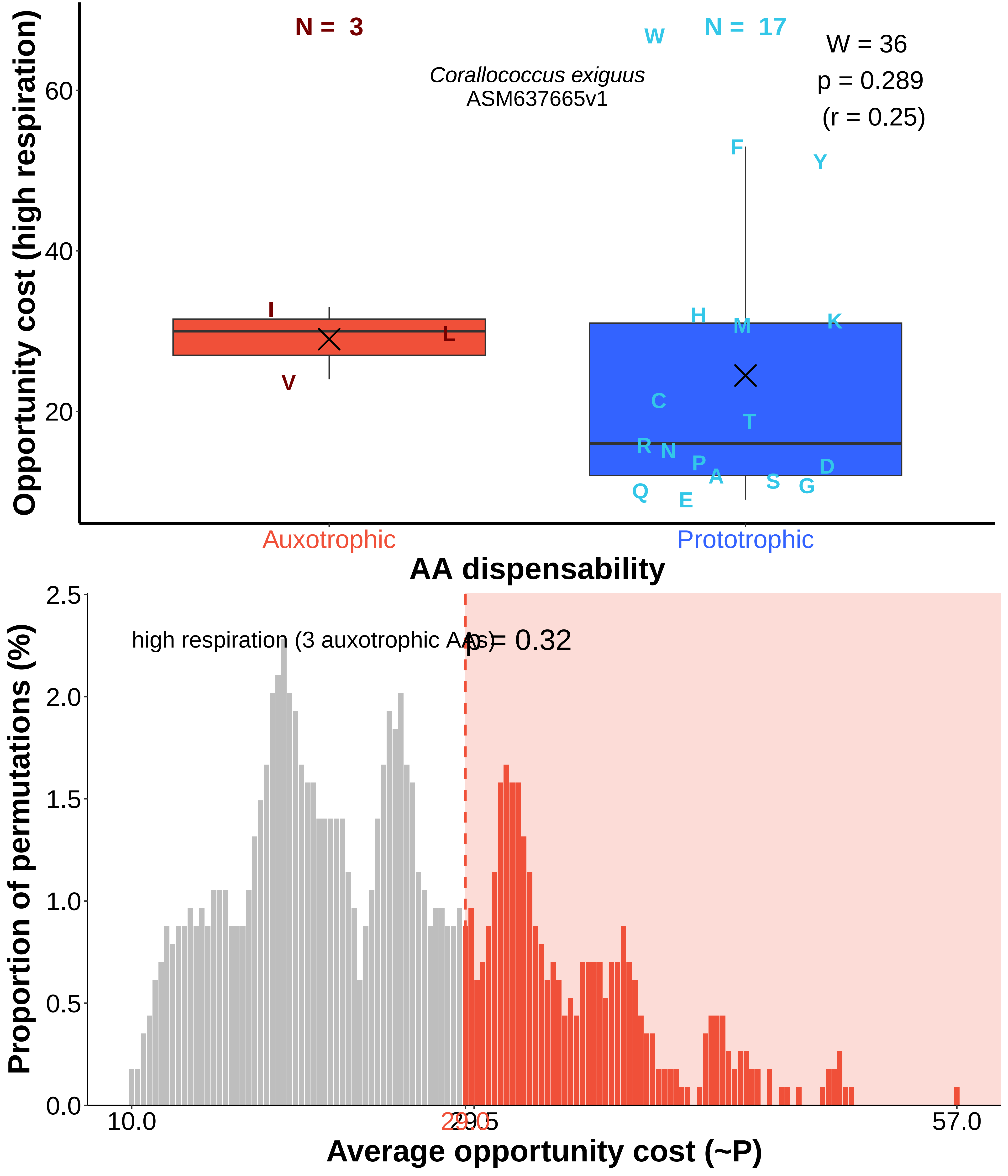
N = 3
N = 17
W
W = 36
p = 0.289
(r = 0.25)
Corallococcus exiguus
ASM637665v1
Opportunity cost (high respiration)
60
40
20
I
L
V
F
Y
H
M
K
C
T
R
N
P
A
D
S
G
Q
E
Auxotrophic
Prototrophic
AA dispensability
Proportion of permutations (%)
2.5
2.0
1.5
1.0
0.5
0.0
high respiration (3 auxotrophic AAs)
p = 0.32
10.0
29.0
29.5
57.0
Average opportunity cost (~P)

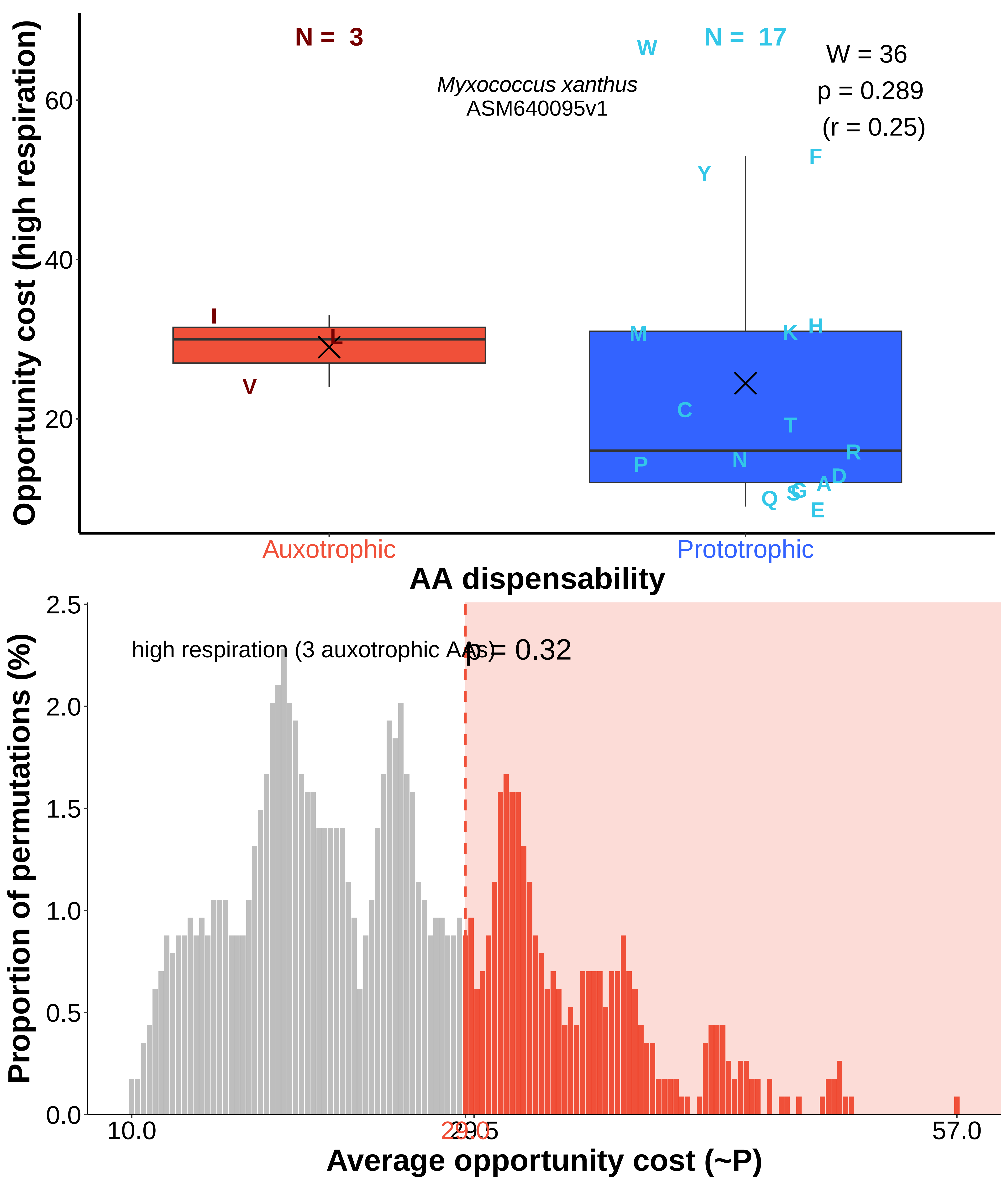

N = 3
N = 17
W = 36
p = 0.289
(r = 0.25)
Myxococcus xanthus
ASM640095v1
Opportunity cost (high respiration)
60
40
20
W
Y
F
I
L
V
M
K
H
C
T
R
P
N
D
A
Q
S
G
E
Auxotrophic
Prototrophic
AA dispensability
high respiration (3 auxotrophic AAs)
p = 0.32
Proportion of permutations (%)
2.5
2.0
1.5
1.0
0.5
0.0
10.0
29.0
29.5
57.0
Average opportunity cost (~P)

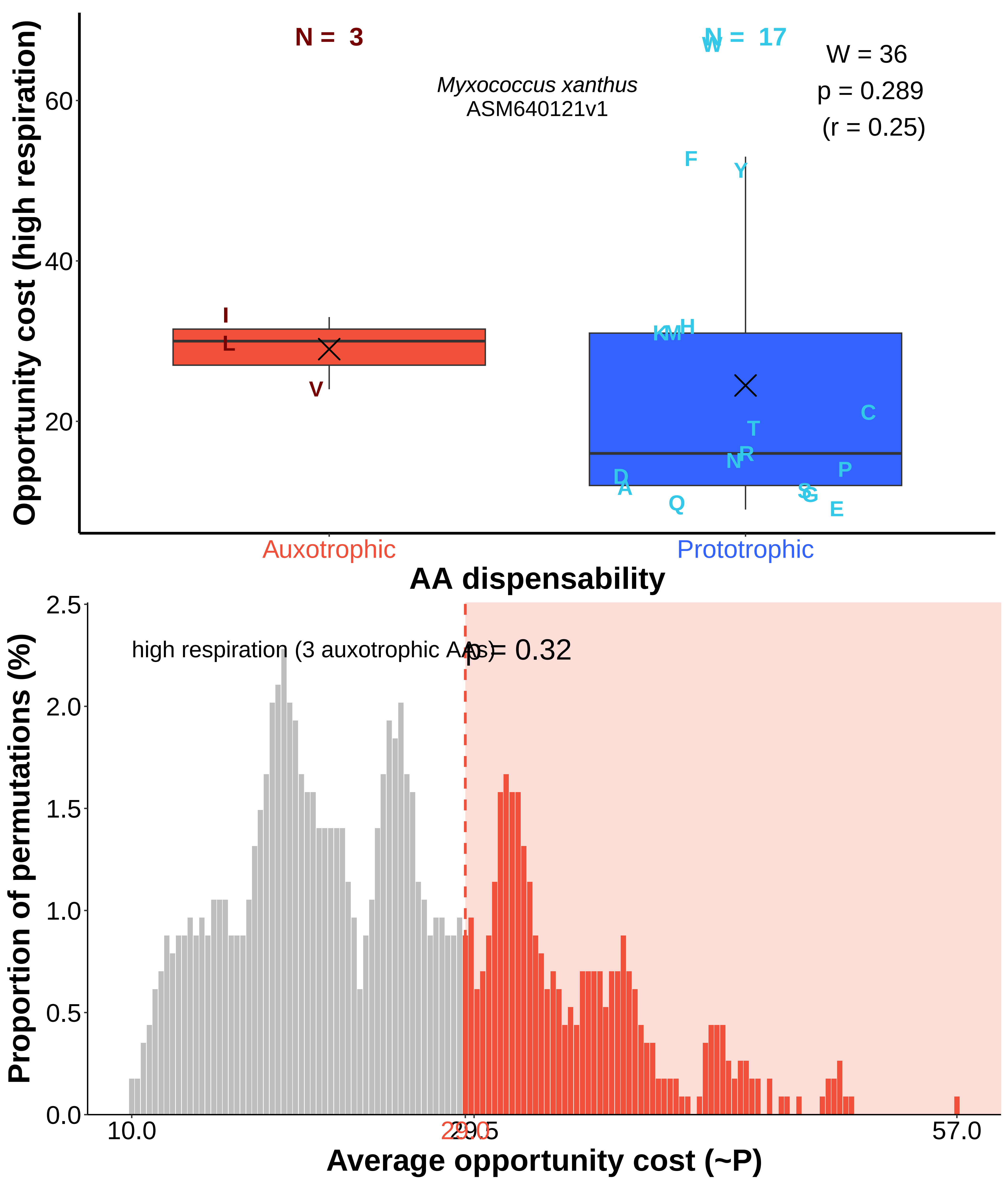
N = 3
N = 17
W
W = 36
p = 0.289
(r = 0.25)
Myxococcus xanthus
ASM640121v1
Opportunity cost (high respiration)
60
40
20
I
L
V
F
Y
K
M
H
C
T
R
N
P
D
A
Q
S
G
E
Auxotrophic
Prototrophic
AA dispensability
high respiration (3 auxotrophic AAs)
p = 0.32
Proportion of permutations (%)
2.5
2.0
1.5
1.0
0.5
0.0
10.0
29.0
29.5
57.0
Average opportunity cost (~P)

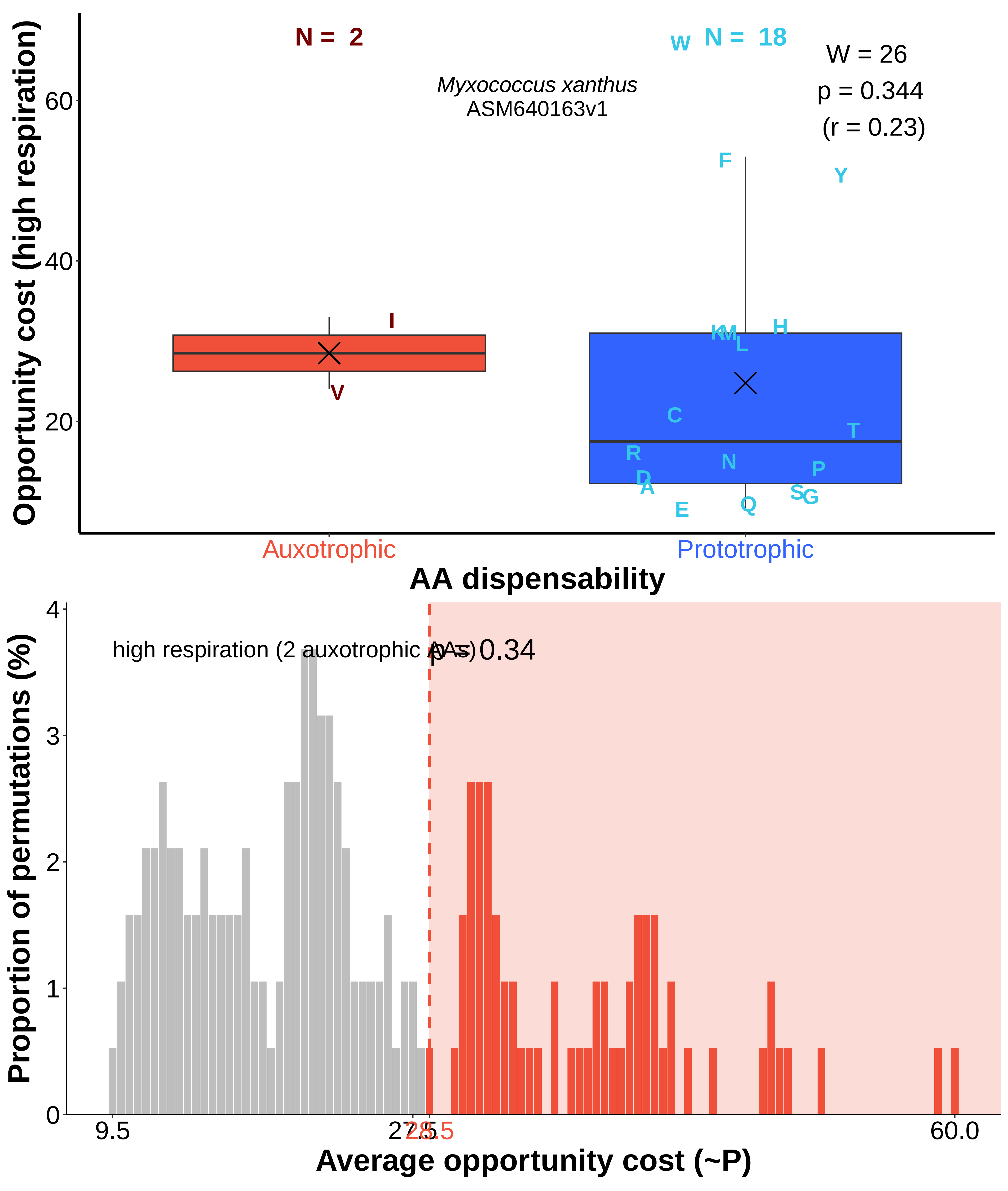

N = 2
W N = 18
W = 26
p = 0.344
(r = 0.23)
Myxococcus xanthus
ASM640163v1
Opportunity cost (high respiration)
Auxotrophic
Prototrophic
AA dispensability
high respiration (2 auxotrophic AAs)
p = 0.34
Proportion of permutations (%)
9.5
27.5
28.5
60.0
Average opportunity cost (~P)

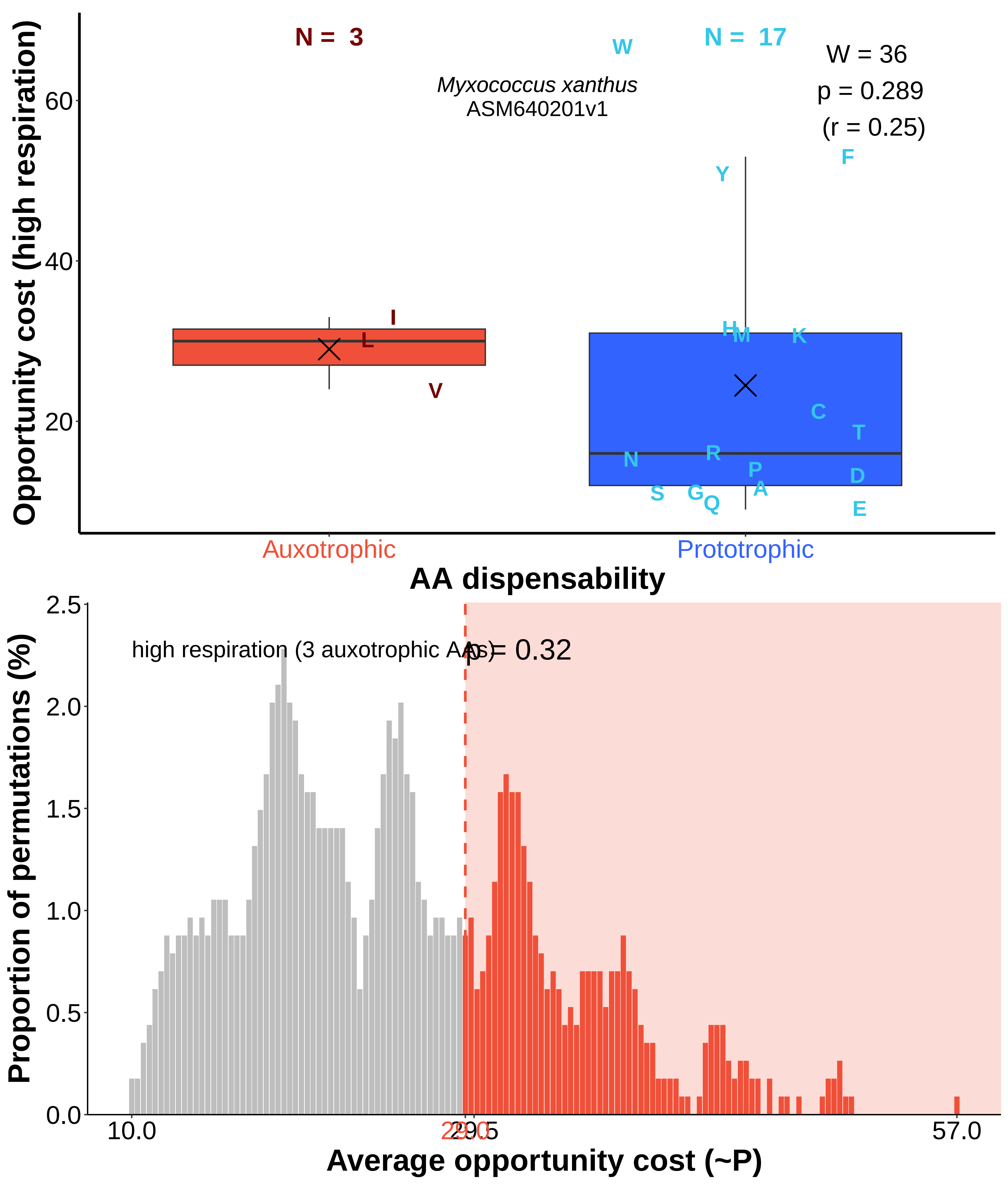
N = 3
W
N = 17
W = 36
p = 0.289
(r = 0.25)
Myxococcus xanthus
ASM640201v1
Opportunity cost (high respiration)
60
40
20
F
Y
I
L
V
H
M
K
C
T
N
R
P
D
S
G
Q
A
E
Auxotrophic
Prototrophic
AA dispensability
Proportion of permutations (%)
2.5
2.0
1.5
1.0
0.5
0.0
high respiration (3 auxotrophic AAs)
p = 0.32
10.0
29.0
29.5
57.0
Average opportunity cost (~P)

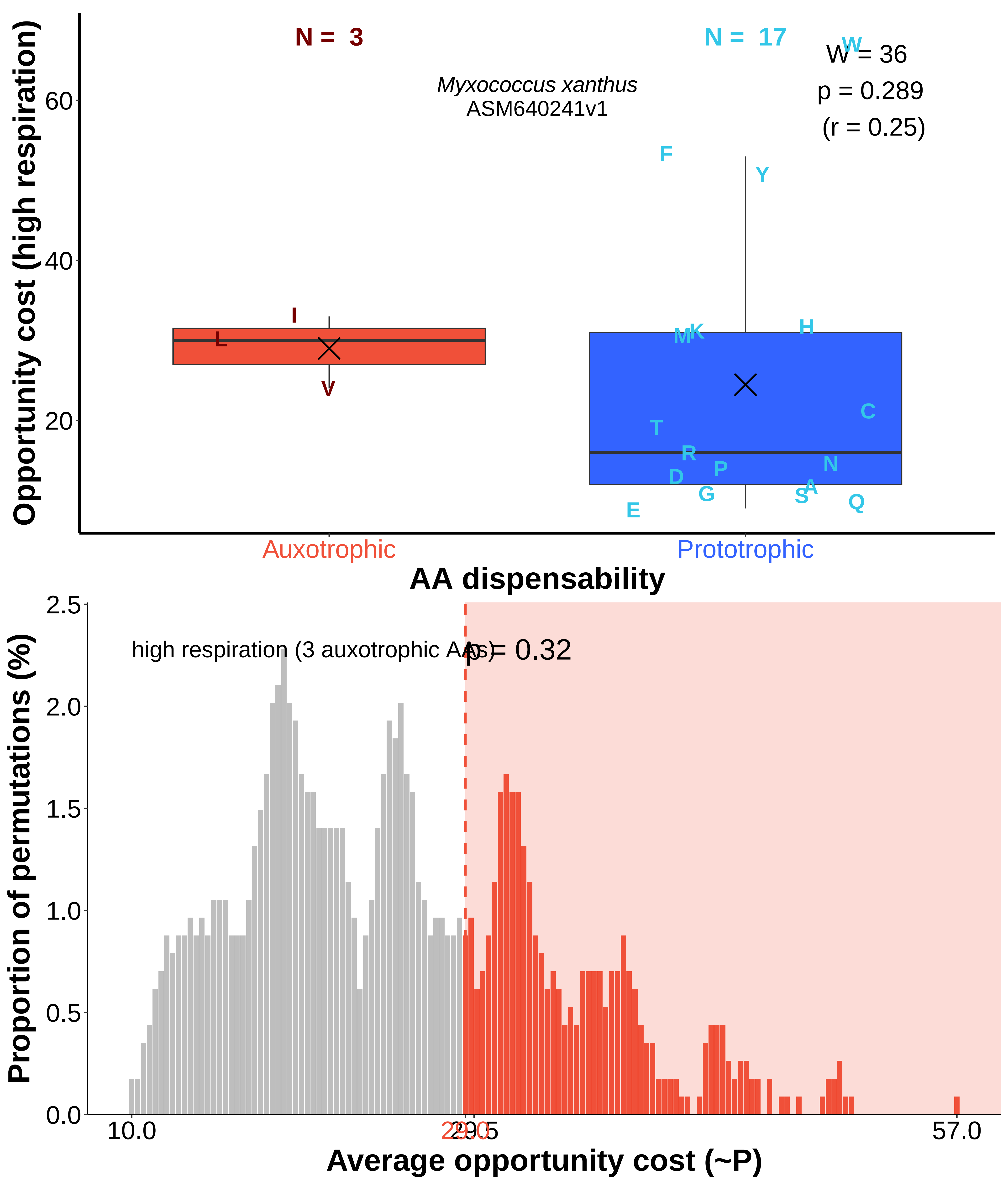

N = 3
N = 17
W = 36
p = 0.289
(r = 0.25)
Myxococcus xanthus
ASM640241v1
Opportunity cost (high respiration)
60
40
20
I
L
V
F
Y
M
K
H
C
T
R
P
N
D
A
S
G
Q
E
W
Auxotrophic
Prototrophic
AA dispensability
high respiration (3 auxotrophic AAs)
p = 0.32
Proportion of permutations (%)
2.5
2.0
1.5
1.0
0.5
0.0
10.0
29.0
29.5
57.0
Average opportunity cost (~P)

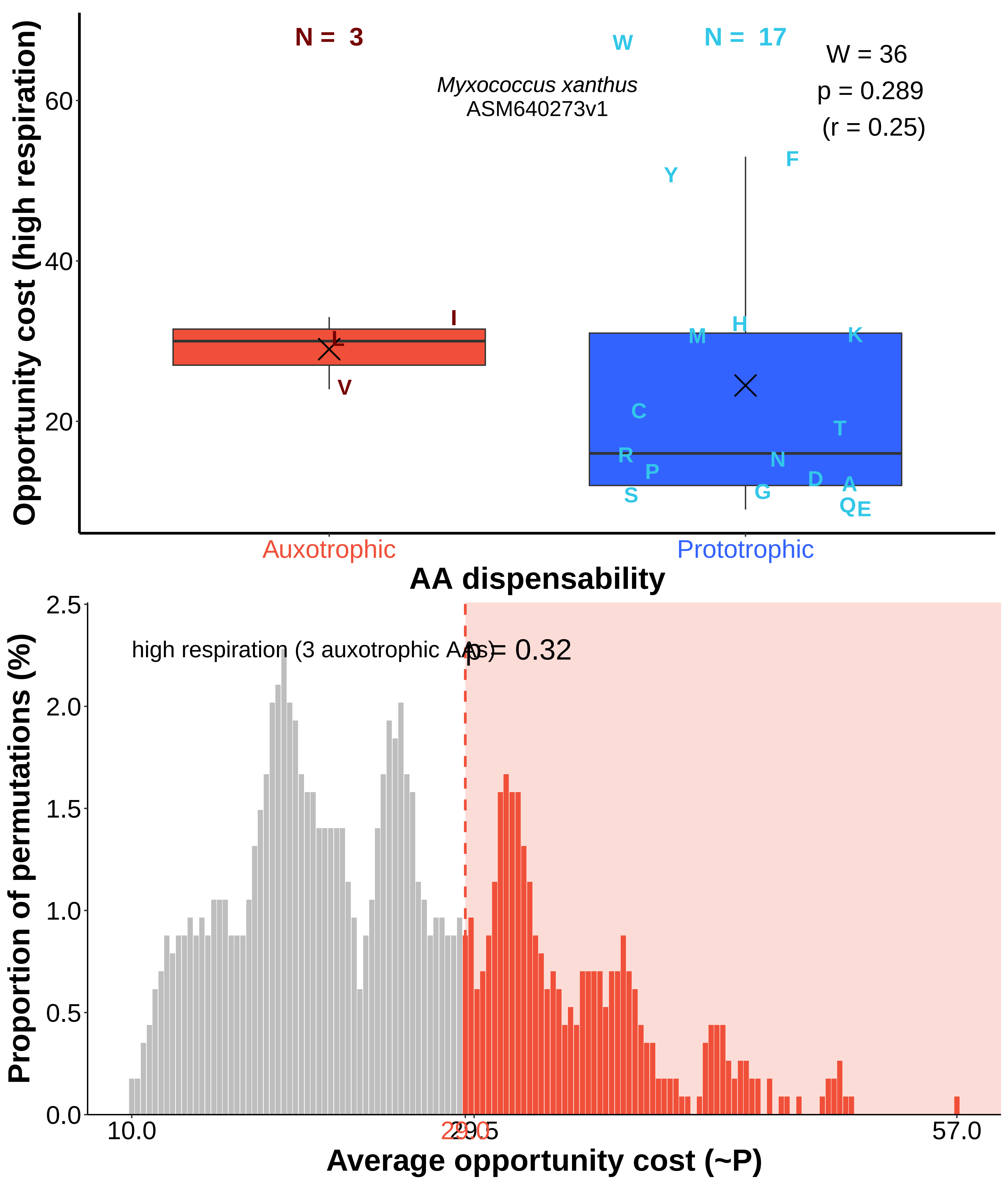
N = 3
W
N = 17
W = 36
p = 0.289
(r = 0.25)
Myxococcus xanthus
ASM640273v1
Opportunity cost (high respiration)
60
40
20
F
Y
I
L
V
H
M
K
C
T
R
N
P
D
A
S
G
Q
E
Auxotrophic
Prototrophic
AA dispensability
high respiration (3 auxotrophic AAs)
p = 0.32
Proportion of permutations (%)
2.5
2.0
1.5
1.0
0.5
0.0
10.0
29.0
29.5
57.0
Average opportunity cost (~P)

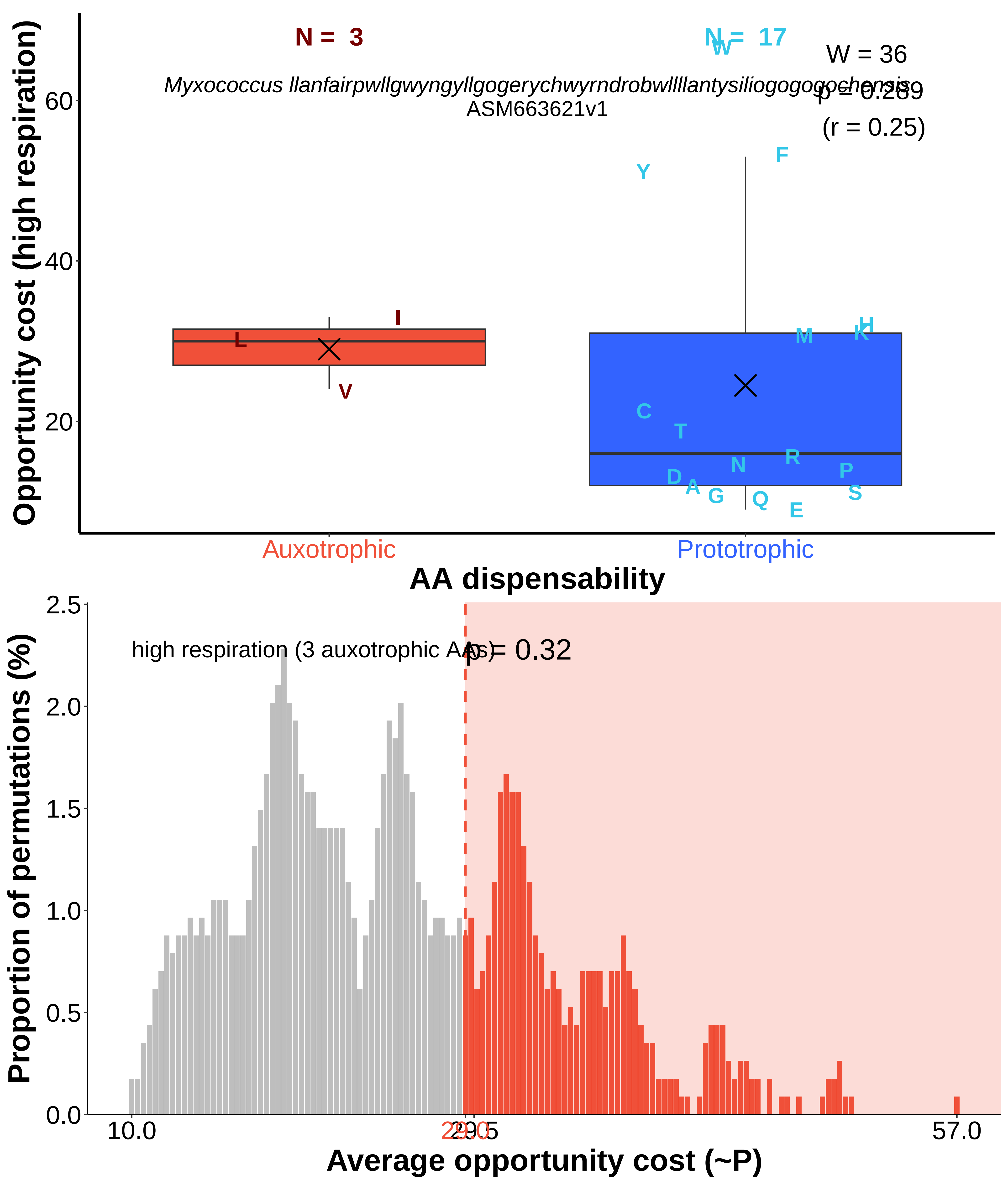

N = 3
N = 17
W
W = 36
p = 0.289
(r = 0.25)
Myxococcus llanfairpwllgwyngyllgogerychwyrndrobwllllantysiliogogogochensis
ASM663621v1
Opportunity cost (high respiration)
60
40
20
L
I
V
Y
F
M
H
K
C
T
R
N
D
A
G
Q
E
P
S
Auxotrophic
Prototrophic
AA dispensability
high respiration (3 auxotrophic AAs)
p = 0.32
Proportion of permutations (%)
2.5
2.0
1.5
1.0
0.5
0.0
10.0
29.0
29.5
57.0
Average opportunity cost (~P)

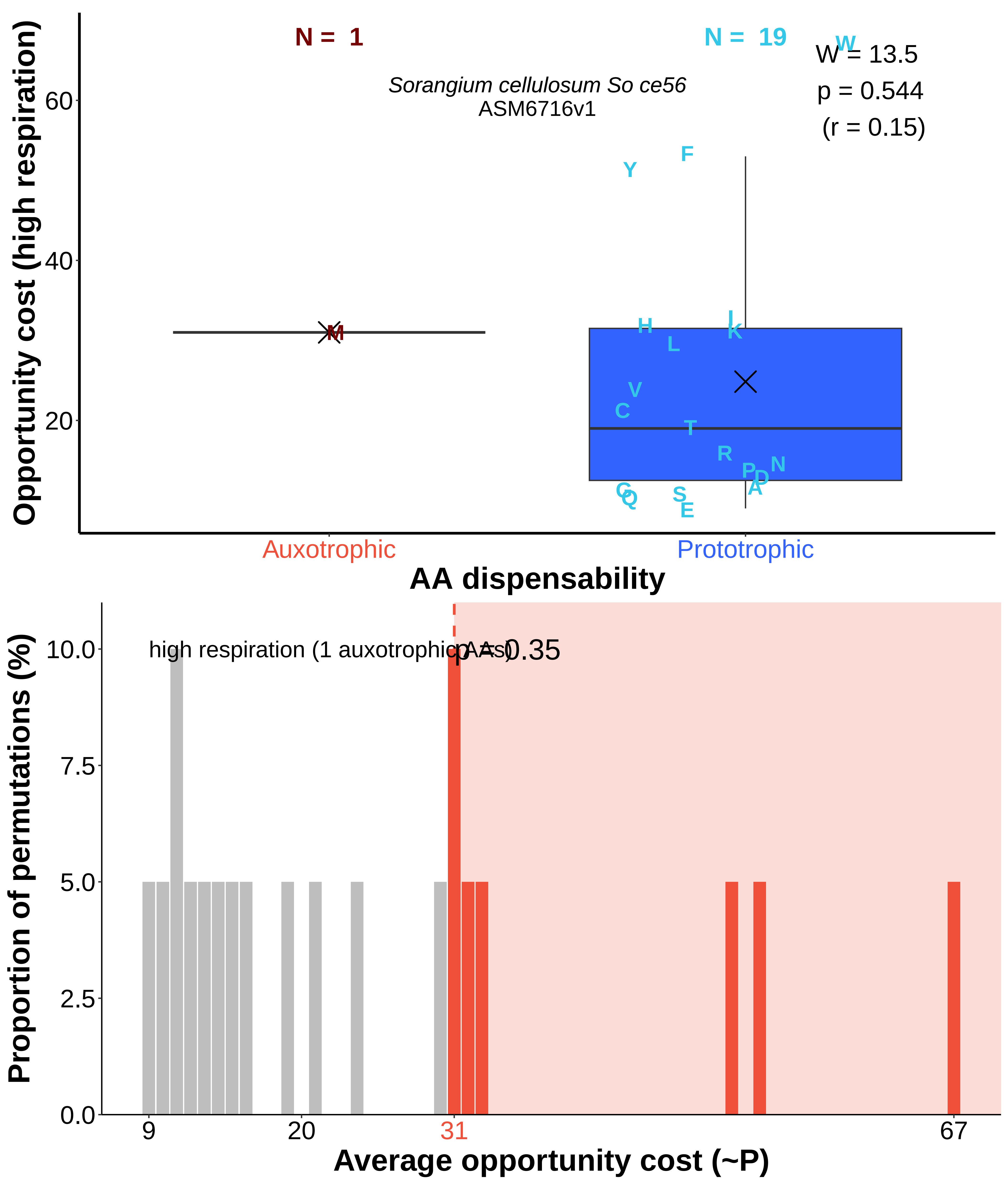

N = 1
N = 19
W = 13.5
p = 0.544
(r = 0.15)
Sorangium cellulosum So ce56
ASM6716v1
Opportunity cost (high respiration)
60
40
20
M
Y
F
W
H
I
K
L
V
C
T
R
P
N
D
A
G
Q
S
E
Auxotrophic
Prototrophic
AA dispensability
high respiration (1 auxotrophic AAs)
p = 0.35
Proportion of permutations (%)
10.0
7.5
5.0
2.5
0.0
9
20
31
67
Average opportunity cost (~P)

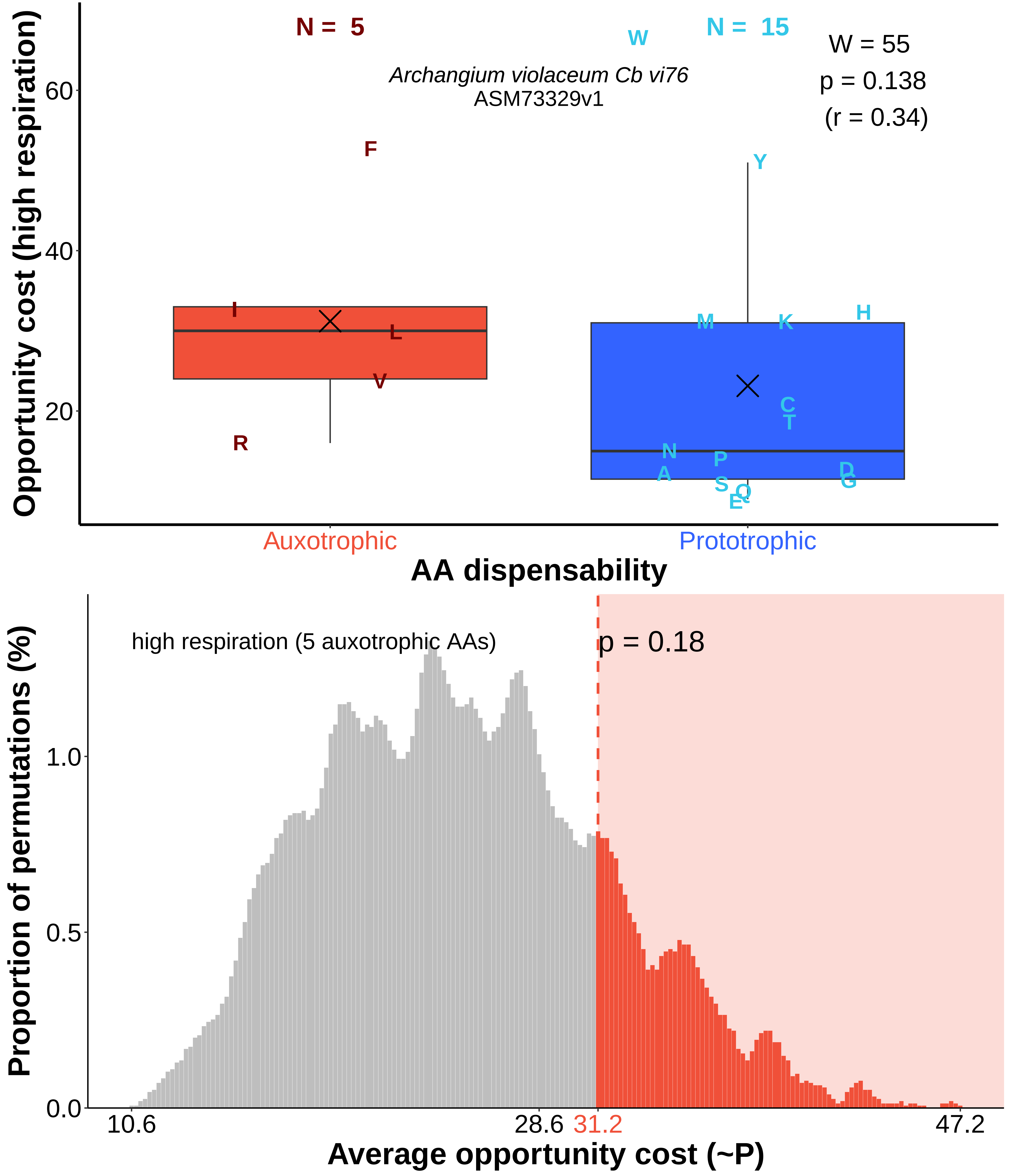
N = 5
N = 15
W = 55
p = 0.138
(r = 0.34)
Archangium violaceum Cb vi76
ASM73329v1
Opportunity cost (high respiration)
60
40
20
Auxotrophic
Prototrophic
AA dispensability
high respiration (5 auxotrophic AAs)
p = 0.18
Proportion of permutations (%)
1.0
0.5
0.0
10.6
28.6
31.2
47.2
Average opportunity cost (~P)

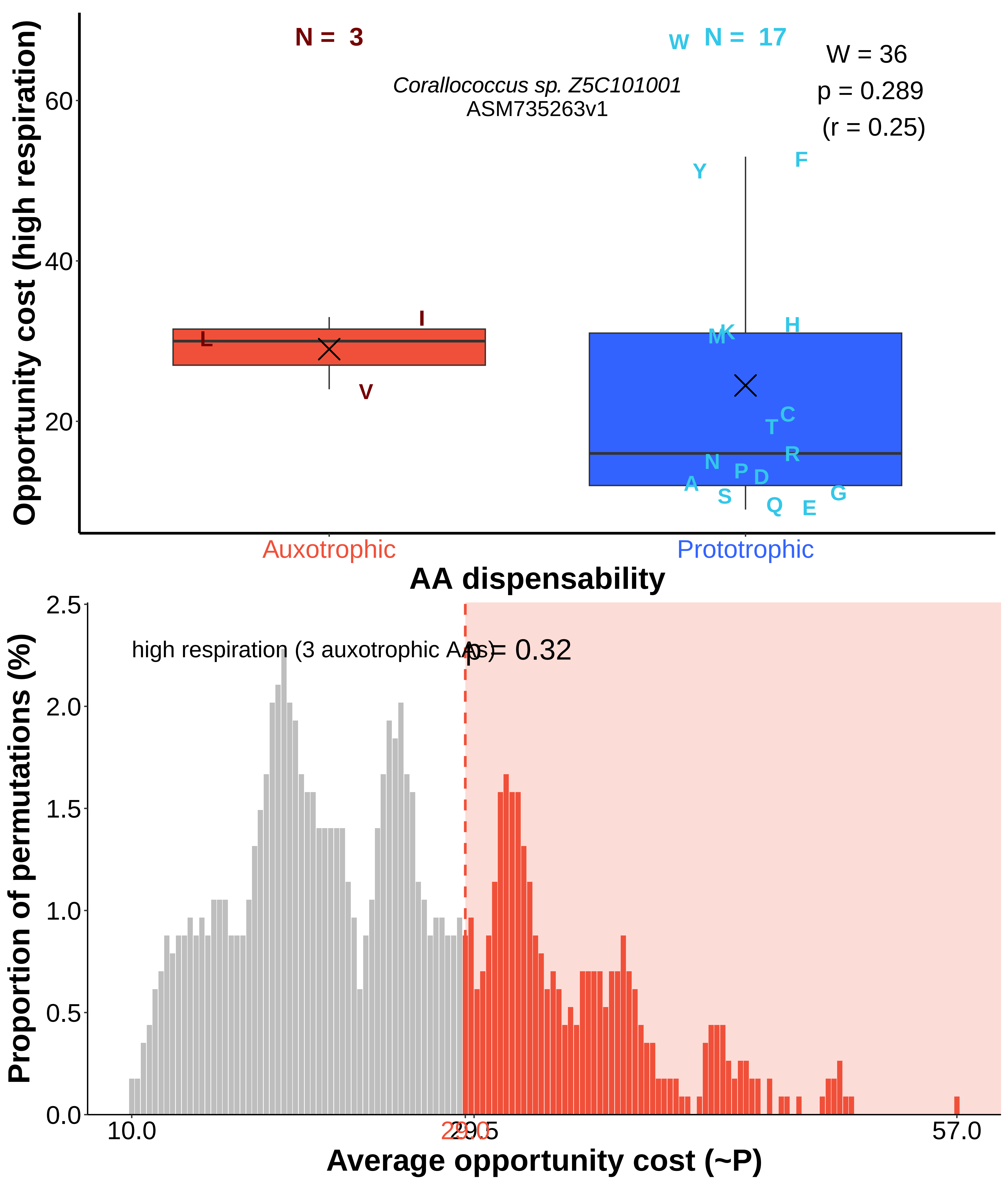

N = 3
N = 17
W
Corallococcus sp. Z5C101001
ASM735263v1
W = 36
p = 0.289
(r = 0.25)
Opportunity cost (high respiration)
60
40
20
Y
F
I
L
V
M
K
H
C
T
R
N
P
D
A
S
Q
E
G
Auxotrophic
Prototrophic
AA dispensability
high respiration (3 auxotrophic AAs)
p = 0.32
Proportion of permutations (%)
2.5
2.0
1.5
1.0
0.5
0.0
10.0
29.0
29.5
57.0
Average opportunity cost (~P)

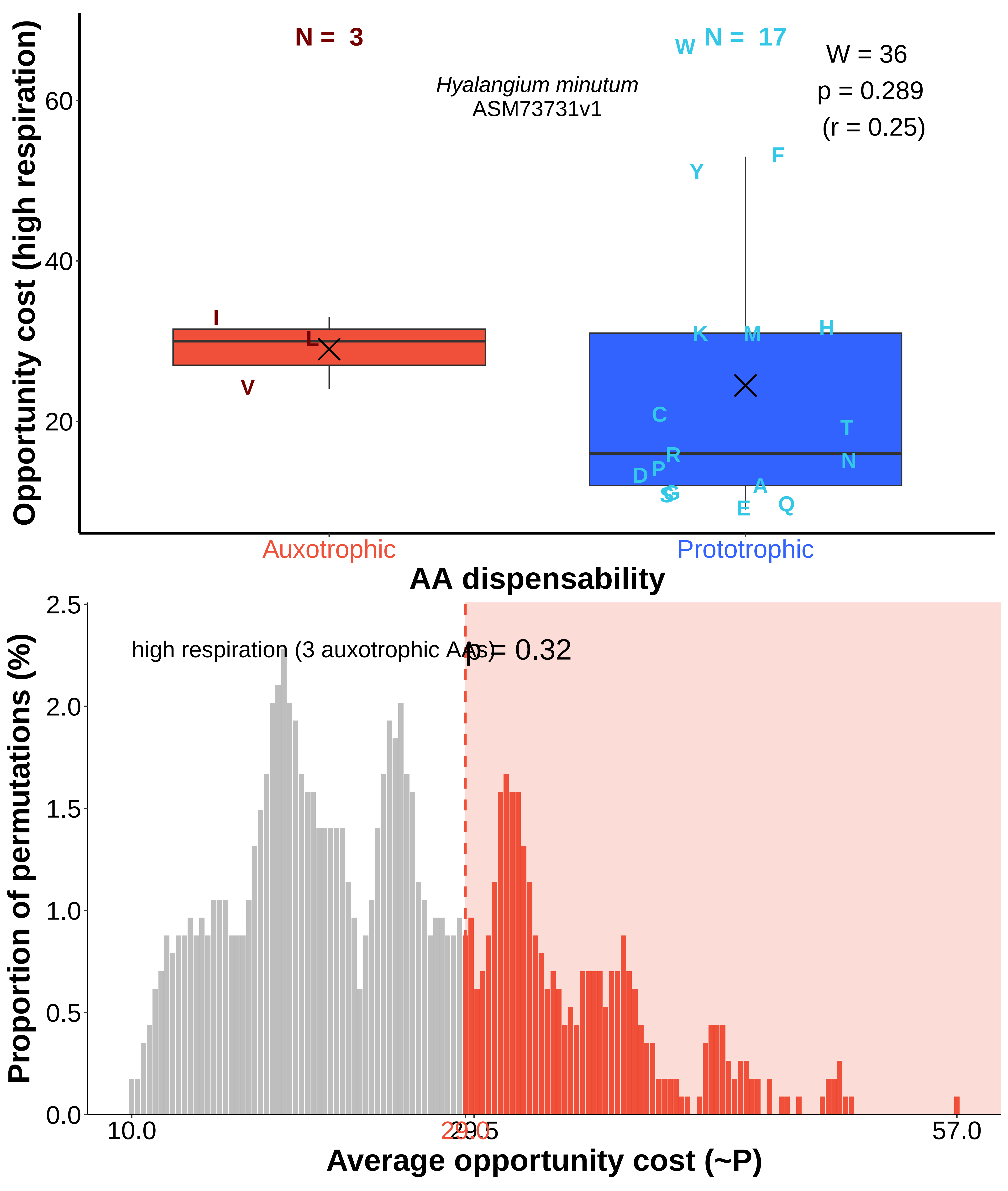
N = 3
W N = 17
W = 36
p = 0.289
(r = 0.25)
Hyalangium minutum
ASM73731v1
Opportunity cost (high respiration)
60
40
20
I
L
V
F
Y
K
M
H
C
T
R
N
D
P
S
G
A
E
Q
Auxotrophic
Prototrophic
AA dispensability
high respiration (3 auxotrophic AAs)
p = 0.32
Proportion of permutations (%)
2.5
2.0
1.5
1.0
0.5
0.0
10.0
29.0
29.5
57.0
Average opportunity cost (~P)

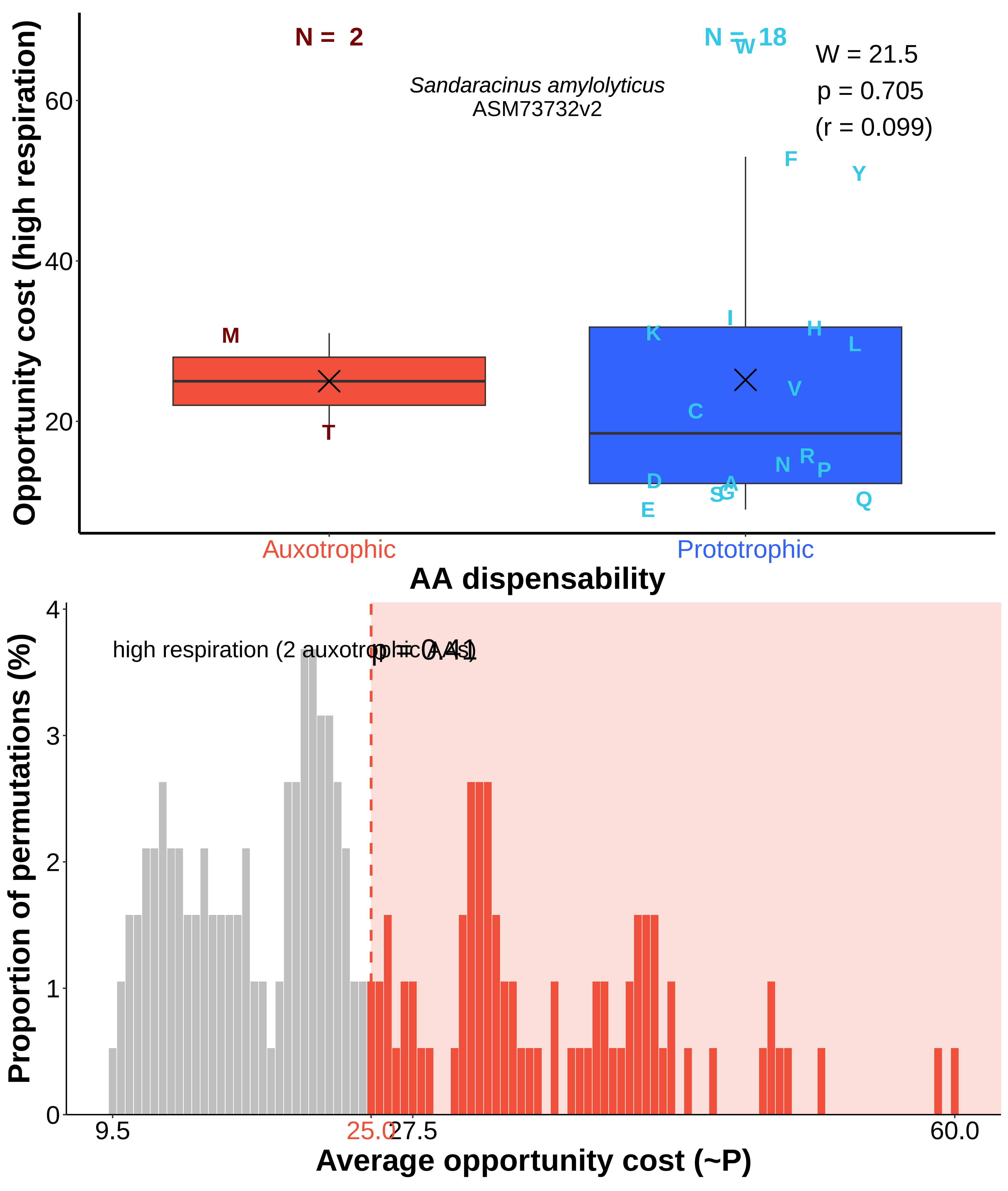

N = 2
N = 18
W
W = 21.5
p = 0.705
(r = 0.099)
Sandaracinus amylolyticus
ASM73732v2
Opportunity cost (high respiration)
60
40
20
M
T
F
Y
I
K
H
L
V
C
N
R
P
D
A
S
G
Q
E
Auxotrophic
Prototrophic
AA dispensability
Proportion of permutations (%)
high respiration (2 auxotrophic AAs)
p = 0.41
4
3
2
1
0
9.5
25.0
27.5
60.0
Average opportunity cost (~P)

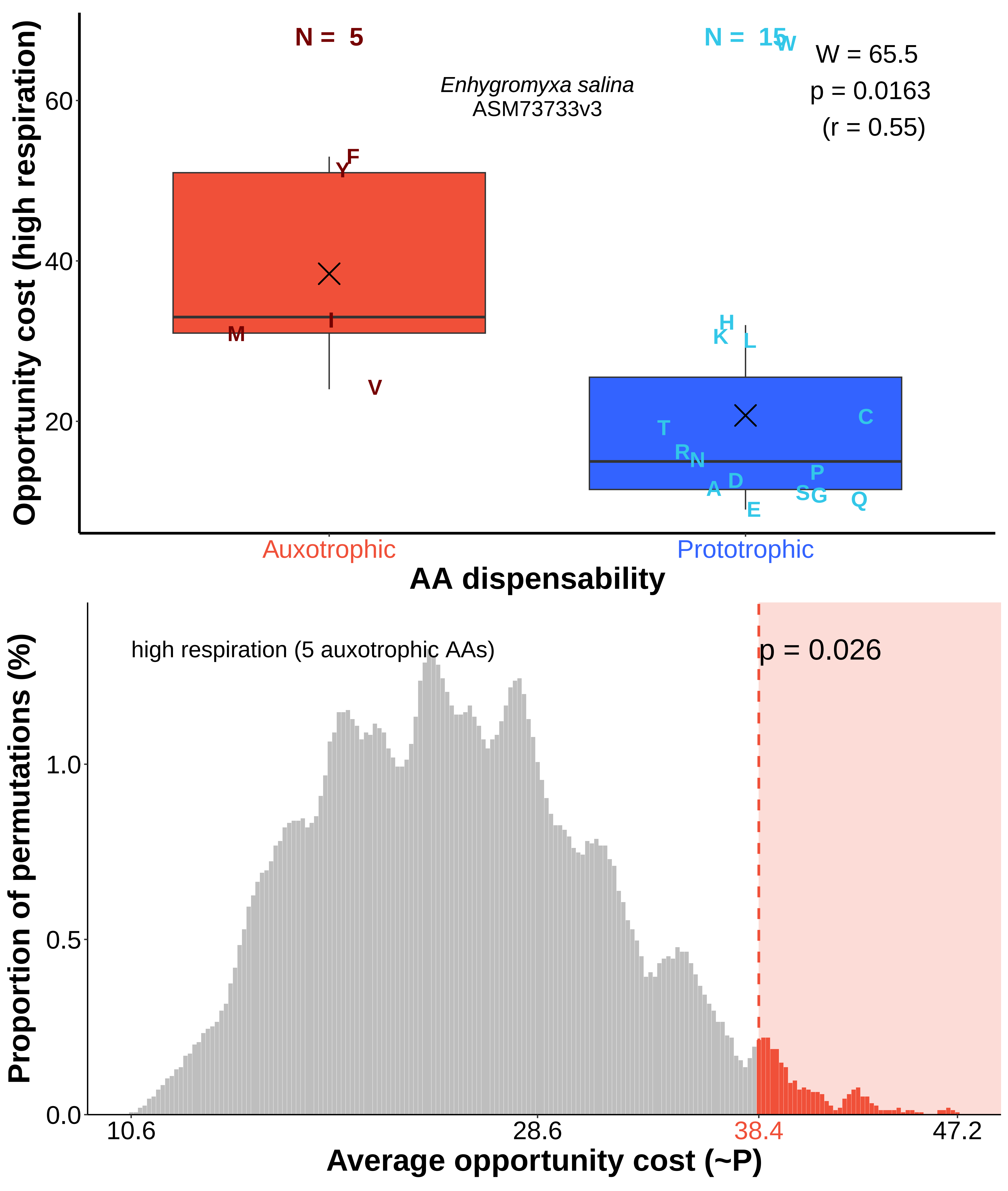
N = 5
N = 15
W = 65.5
p = 0.0163
(r = 0.55)
Enhygromyxa salina
ASM73733v3
Opportunity cost (high respiration)
Auxotrophic
Prototrophic
AA dispensability
high respiration (5 auxotrophic AAs)
p = 0.026
Proportion of permutations (%)
Average opportunity cost (~P)

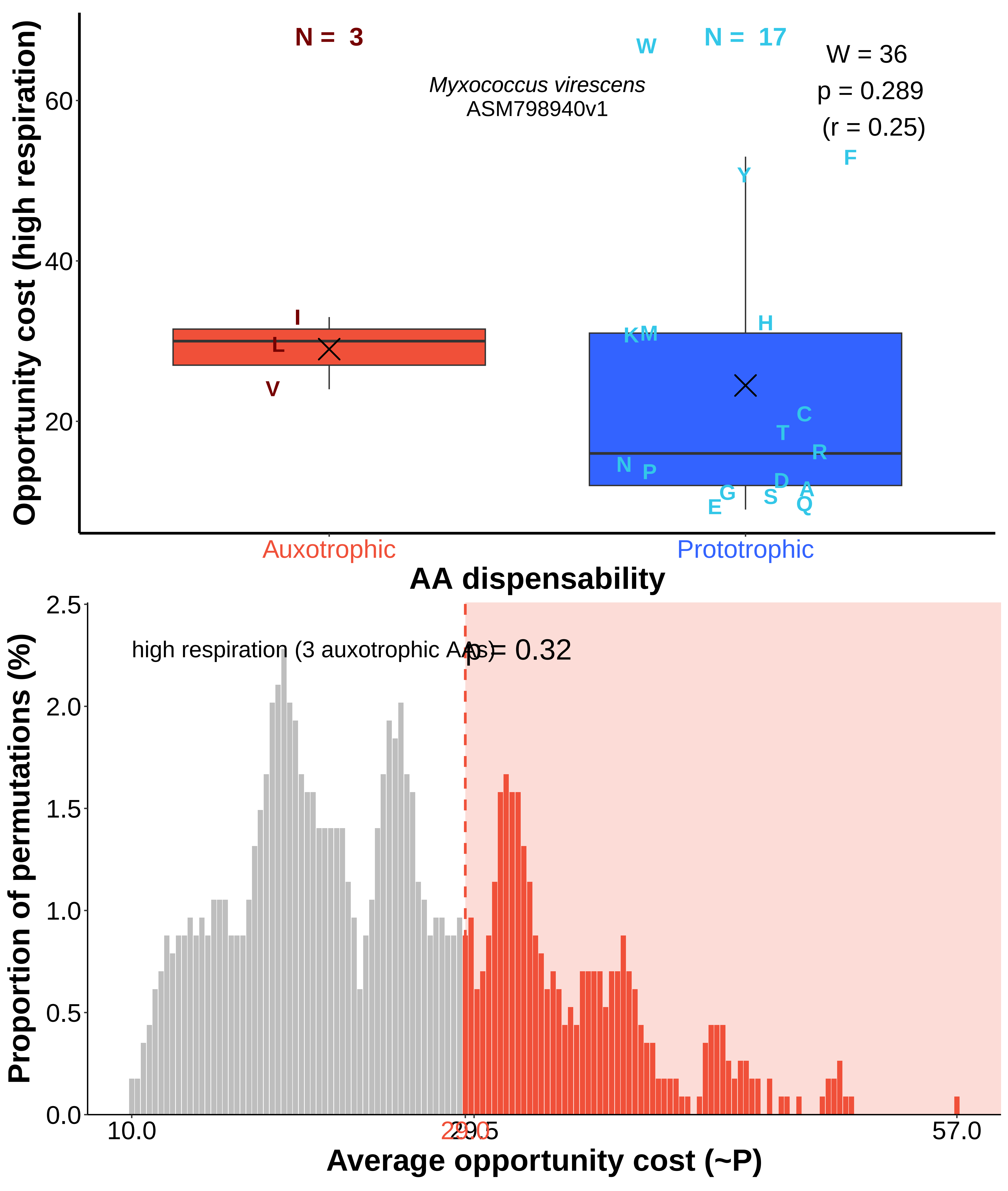
N = 3
N = 17
W
Myxococcus virescens
ASM798940v1
W = 36
p = 0.289
(r = 0.25)
F
Y
I
L
V
H
KM
C
T
R
N
P
D
G
A
S
E
Q
60
40
20
Opportunity cost (high respiration)
Auxotrophic
Prototrophic
AA dispensability
high respiration (3 auxotrophic AAs)
p = 0.32
2.5
2.0
1.5
1.0
0.5
0.0
Proportion of permutations (%)
10.0
29.0
29.5
57.0
Average opportunity cost (~P)

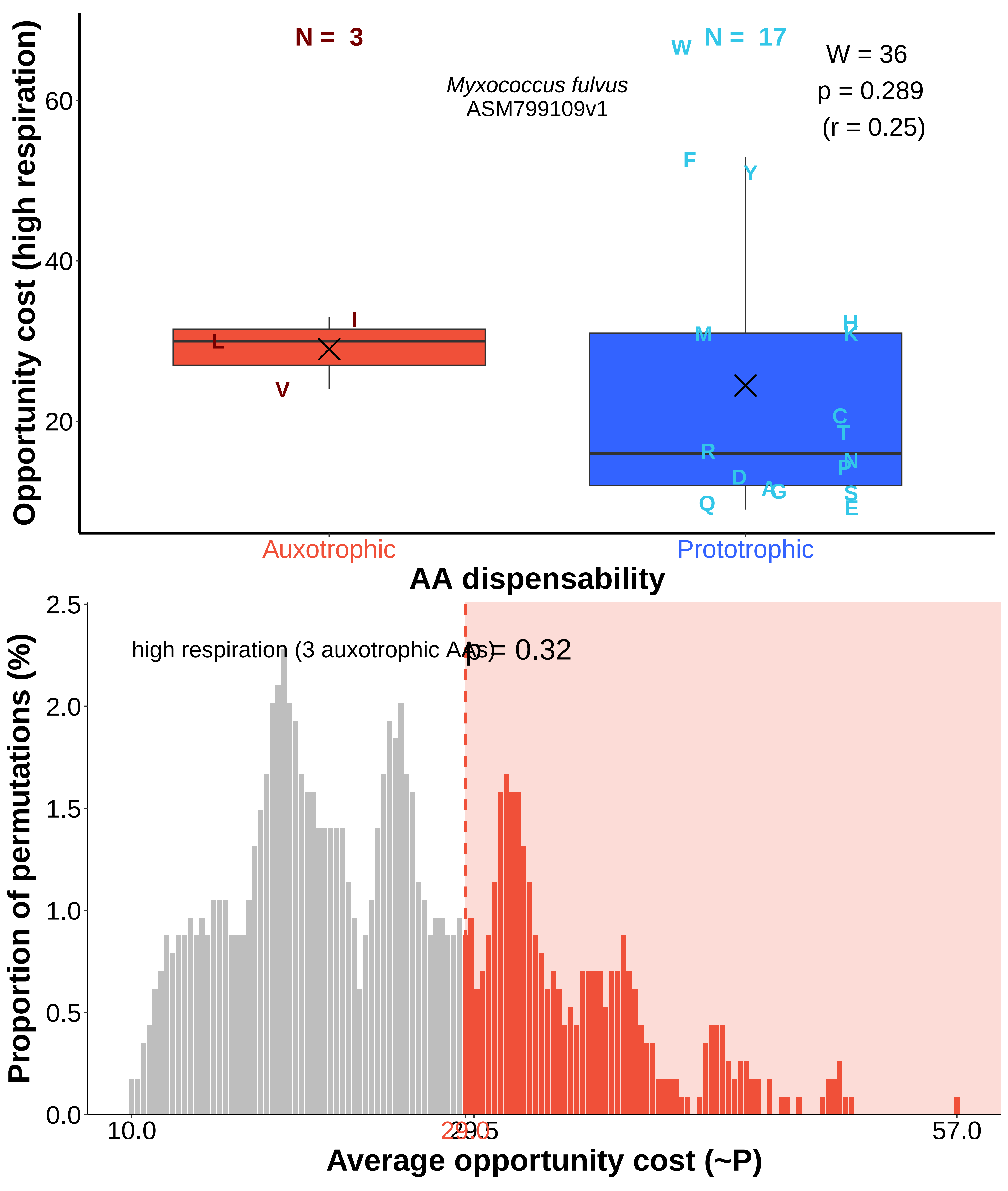
N = 3
W
N = 17
W = 36
p = 0.289
(r = 0.25)
Myxococcus fulvus
ASM799109v1
Opportunity cost (high respiration)
60
40
20
F
Y
I
L
V
M
H
K
C
T
R
N
P
D
A
G
S
Q
E
Auxotrophic
Prototrophic
AA dispensability
high respiration (3 auxotrophic AAs)
p = 0.32
Proportion of permutations (%)
2.5
2.0
1.5
1.0
0.5
0.0
10.0
29.0
29.5
57.0
Average opportunity cost (~P)

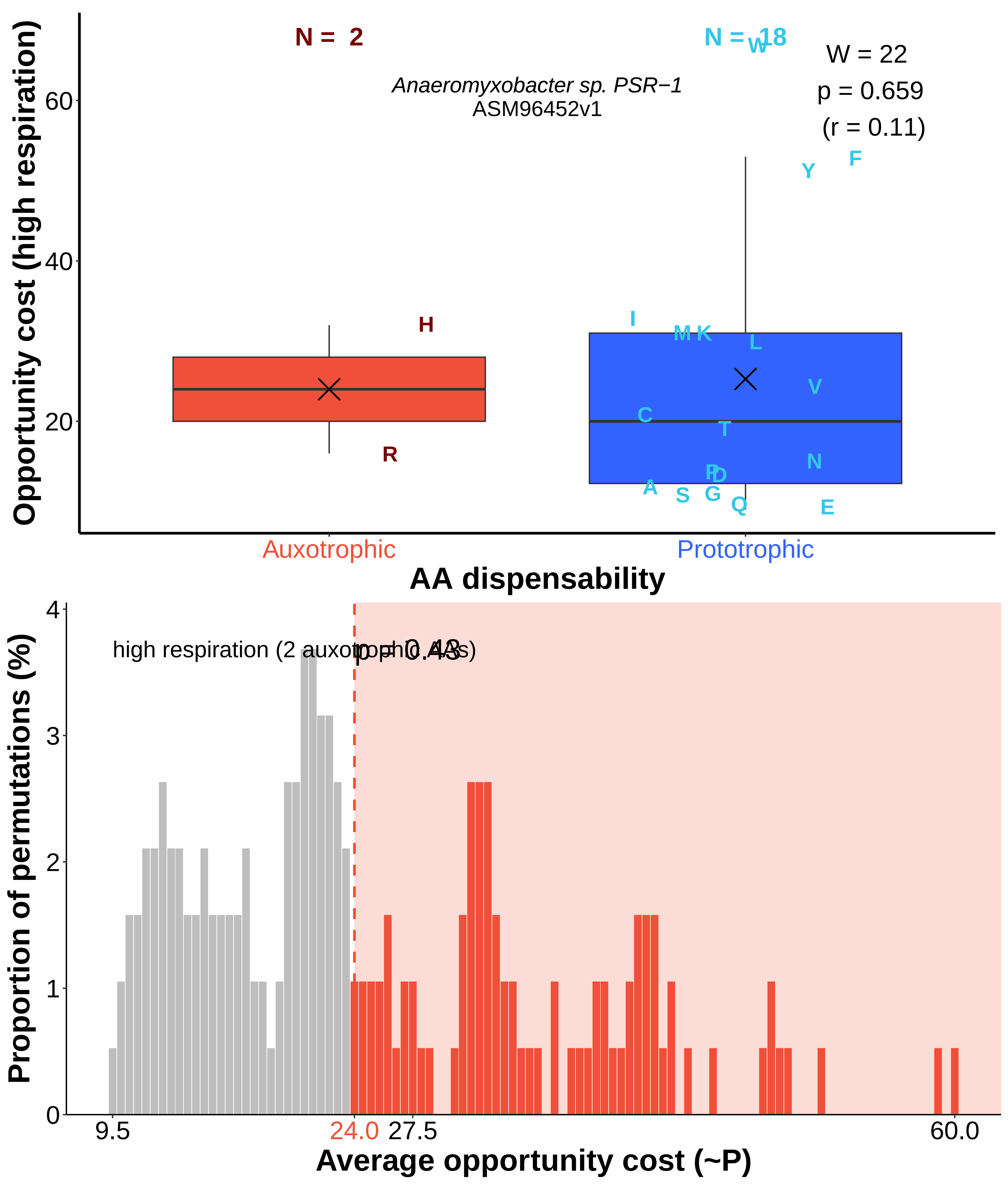

N = 2
N = 18
W = 22
p = 0.659
(r = 0.11)
Anaeromyxobacter sp. PSR−1
ASM96452v1
Opportunity cost (high respiration)
60
40
20
H
R
W
Y
F
I
M K
L
V
C
T
N
P
D
A
S
G
Q
E
Auxotrophic
Prototrophic
AA dispensability
Proportion of permutations (%)
4
3
2
1
0
high respiration (2 auxotrophic AAs)
p = 0.43
9.5
24.0
27.5
60.0
Average opportunity cost (~P)

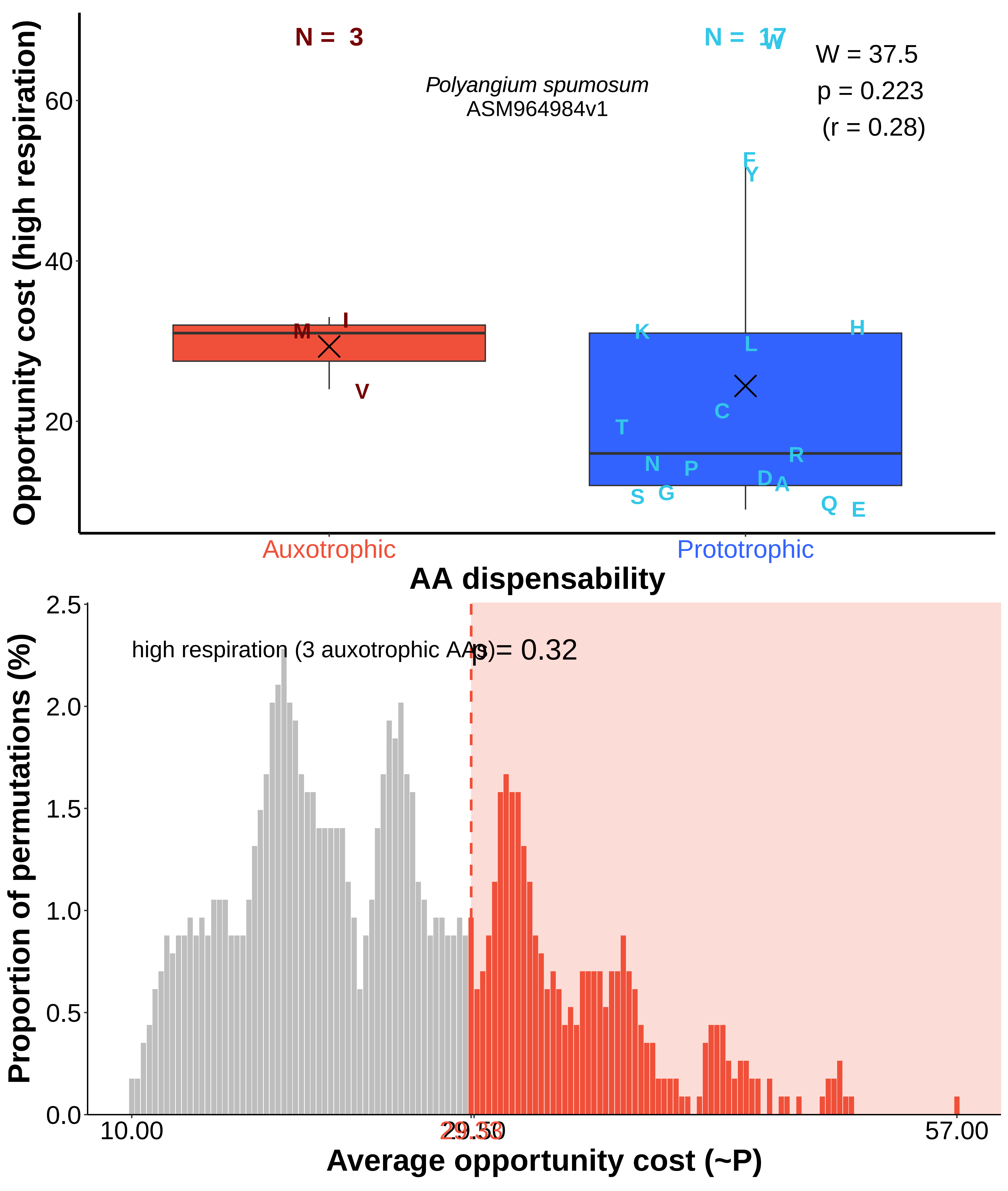
N = 3
N = 17
W
W = 37.5
p = 0.223
(r = 0.28)
Polyangium spumosum
ASM964984v1
Opportunity cost (high respiration)
60
40
20
F
Y
I
M
V
K
H
L
C
T
R
N
P
D
A
S
G
Q
E
Auxotrophic
Prototrophic
AA dispensability
high respiration (3 auxotrophic AAs)
p = 0.32
Proportion of permutations (%)
2.5
2.0
1.5
1.0
0.5
0.0
10.00
29.50
29.33
57.00
Average opportunity cost (~P)

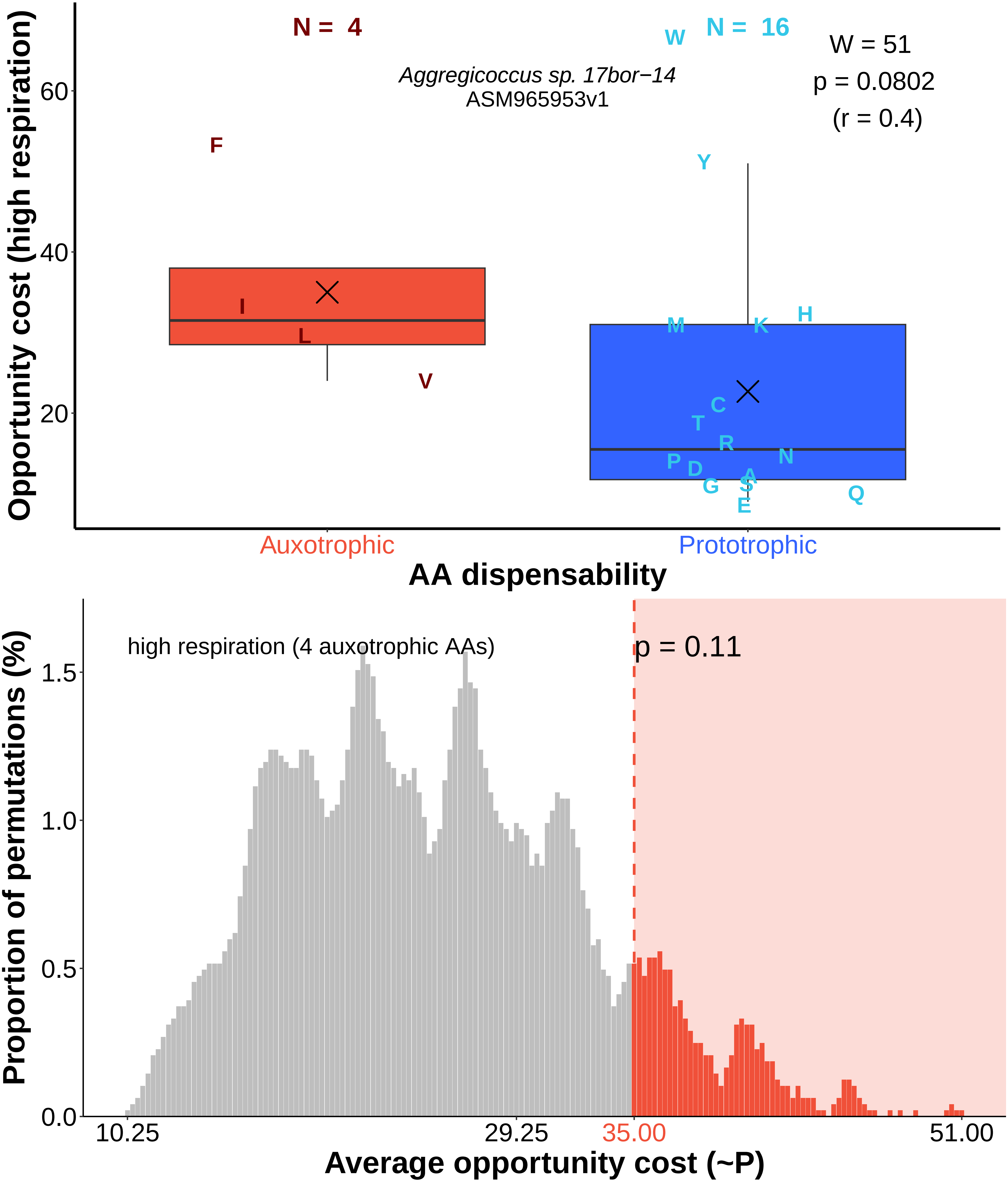

N = 4
N = 16
W
W = 51
p = 0.0802
(r = 0.4)
Aggregicoccus sp. 17bor−14
ASM965953v1
F
Y
I
L
V
M
K
H
C
T
R
N
P
D
A
G
S
E
Q
60
40
20
Opportunity cost (high respiration)
Auxotrophic
Prototrophic
AA dispensability
high respiration (4 auxotrophic AAs)
p = 0.11
1.5
1.0
0.5
0.0
Proportion of permutations (%)
10.25
29.25
35.00
51.00
Average opportunity cost (~P)

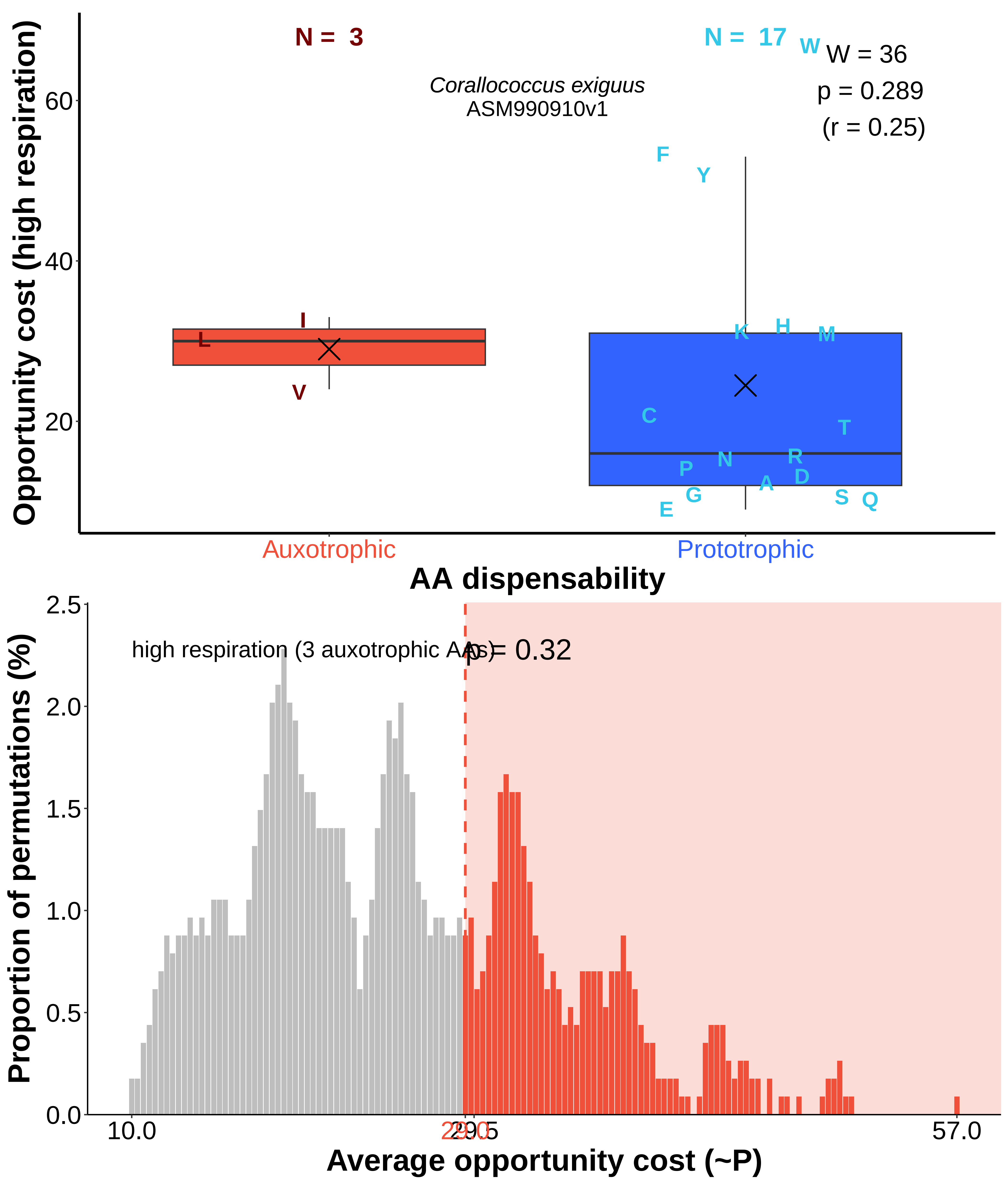
N = 3
N = 17
W
W = 36
p = 0.289
(r = 0.25)
Corallococcus exiguus
ASM990910v1
Opportunity cost (high respiration)
60
40
20
I
L
V
F
Y
K
H
M
C
T
N
R
P
D
A
G
S
Q
E
Auxotrophic
Prototrophic
AA dispensability
high respiration (3 auxotrophic AAs)
p = 0.32
Proportion of permutations (%)
2.5
2.0
1.5
1.0
0.5
0.0
10.0
29.0
29.5
57.0
Average opportunity cost (~P)

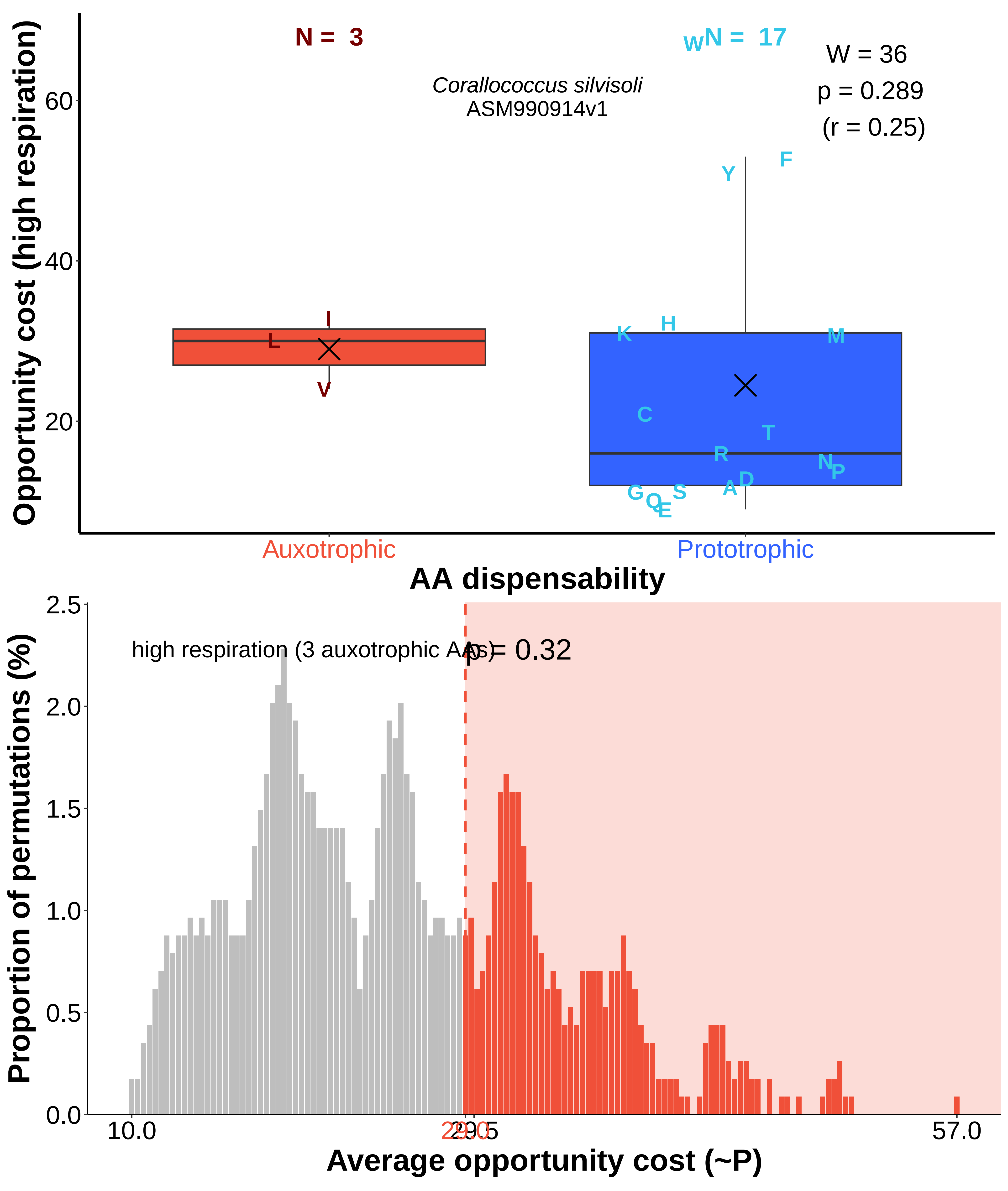

N = 3
WN = 17
W = 36
p = 0.289
(r = 0.25)
Corallococcus silvisoli
ASM990914v1
Opportunity cost (high respiration)
60
40
20
I
L
V
Y
F
K
H
M
C
T
R
N
P
D
A
G
Q
E
S
Auxotrophic
Prototrophic
AA dispensability
Proportion of permutations (%)
2.5
2.0
1.5
1.0
0.5
0.0
high respiration (3 auxotrophic AAs)
p = 0.32
10.0
29.0
29.5
57.0
Average opportunity cost (~P)

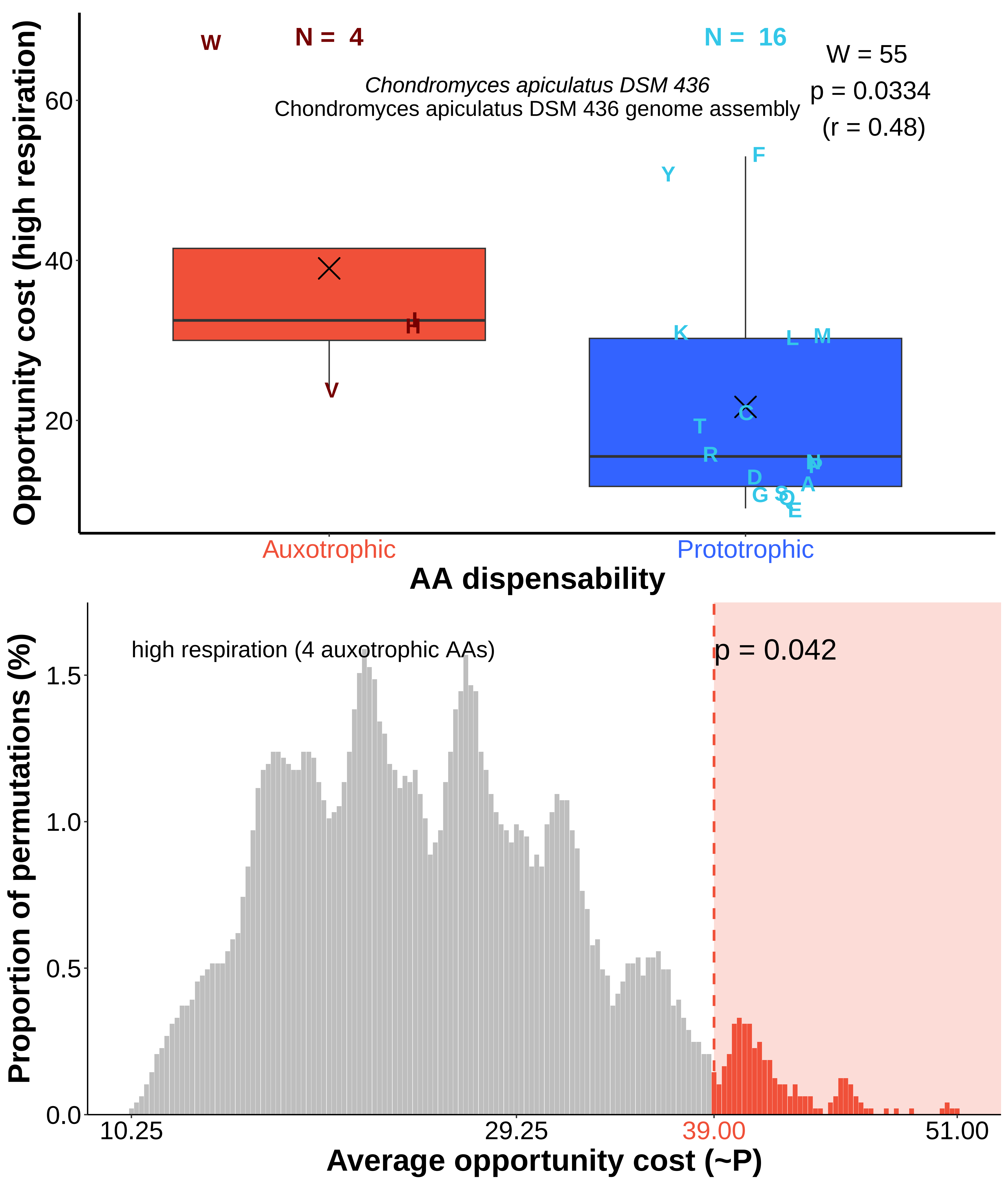

W
N = 4
N = 16
W = 55
p = 0.0334
(r = 0.48)
Chondromyces apiculatus DSM 436
Chondromyces apiculatus DSM 436 genome assembly
Opportunity cost (high respiration)
60
40
20
Y
F
K
L
M
H
I
V
C
T
R
N
P
D
A
G
S
Q
E
Auxotrophic
Prototrophic
AA dispensability
high respiration (4 auxotrophic AAs)
p = 0.042
Proportion of permutations (%)
1.5
1.0
0.5
0.0
10.25
29.25
39.00
51.00
Average opportunity cost (~P)

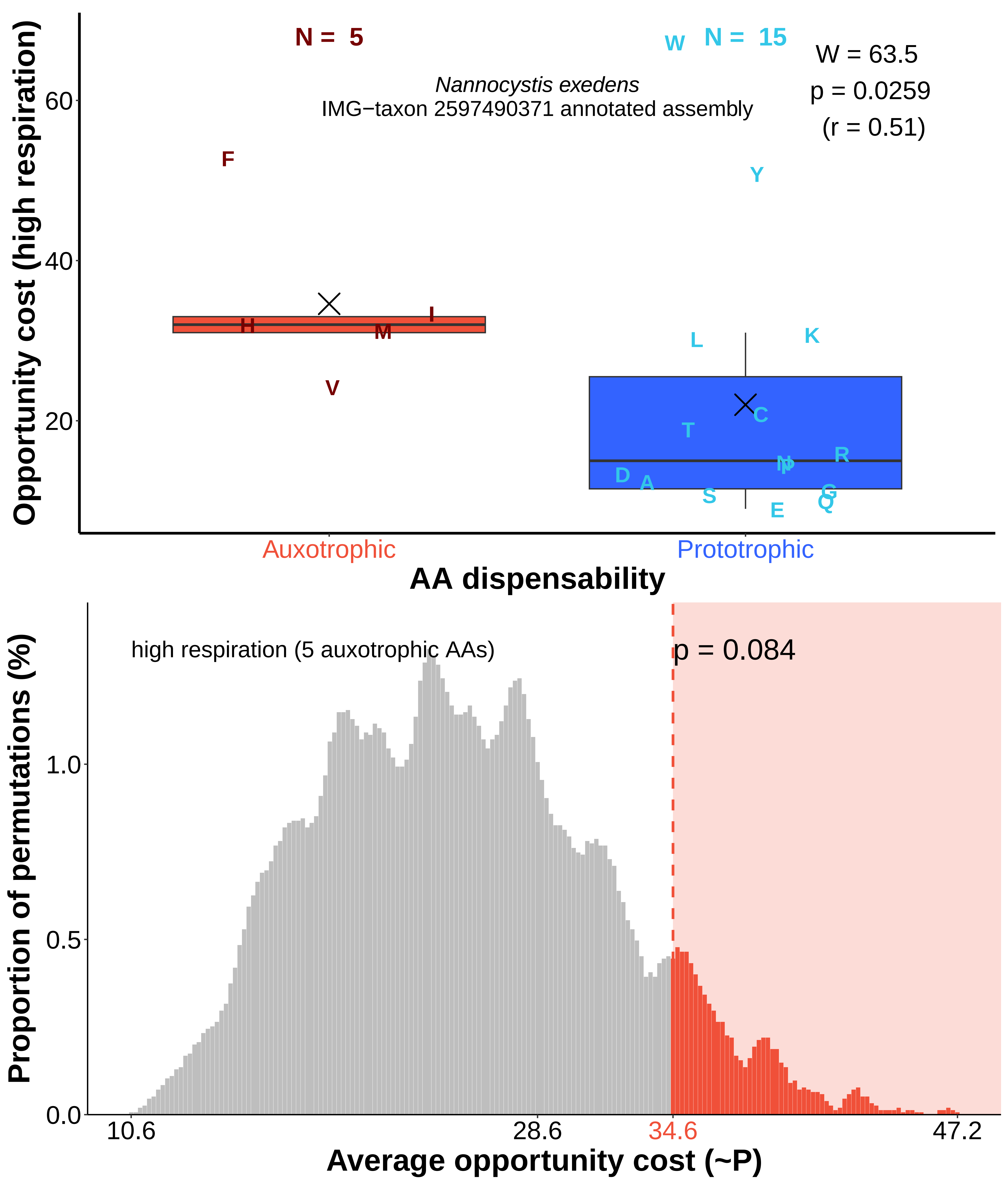

N = 5
W N = 15
W = 63.5
p = 0.0259
(r = 0.51)
Nannocystis exedens
IMG−taxon 2597490371 annotated assembly
Opportunity cost (high respiration)
60
40
20
F
Y
H
I
M
V
L
K
C
T
R
N
P
D
A
S
G
Q
E
Auxotrophic
Prototrophic
AA dispensability
high respiration (5 auxotrophic AAs)
p = 0.084
Proportion of permutations (%)
1.0
0.5
0.0
10.6
28.6
34.6
47.2
Average opportunity cost (~P)

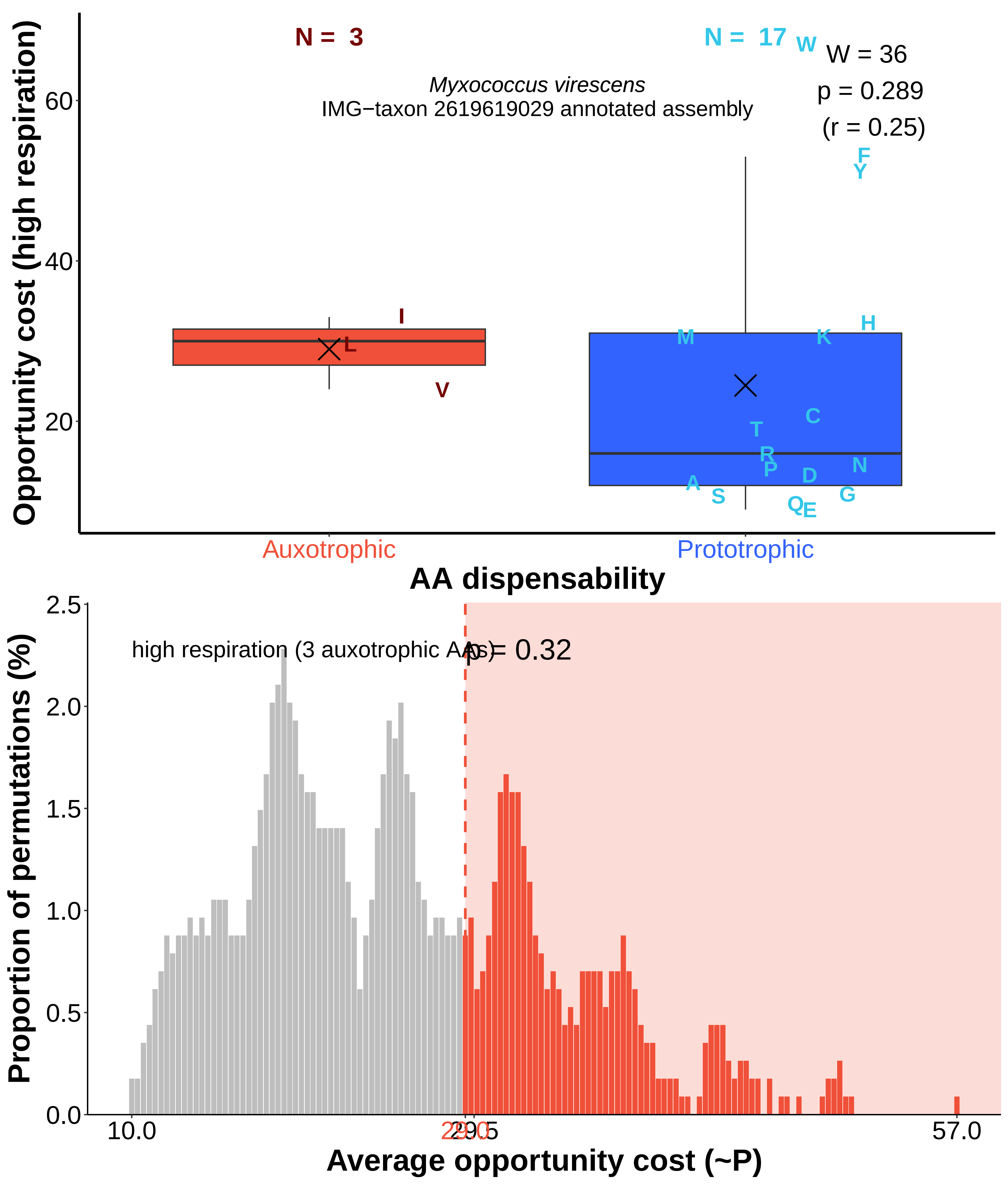

N = 3
N = 17
W
W = 36
p = 0.289
(r = 0.25)
Myxococcus virescens
IMG−taxon 2619619029 annotated assembly
Opportunity cost (high respiration)
60
40
20
F
Y
I
L
V
H
M
K
C
T
R
P
D
N
A
S
Q
E
G
Auxotrophic
Prototrophic
AA dispensability
high respiration (3 auxotrophic AAs)
p = 0.32
Proportion of permutations (%)
2.5
2.0
1.5
1.0
0.5
0.0
10.0
29.0
29.5
57.0
Average opportunity cost (~P)

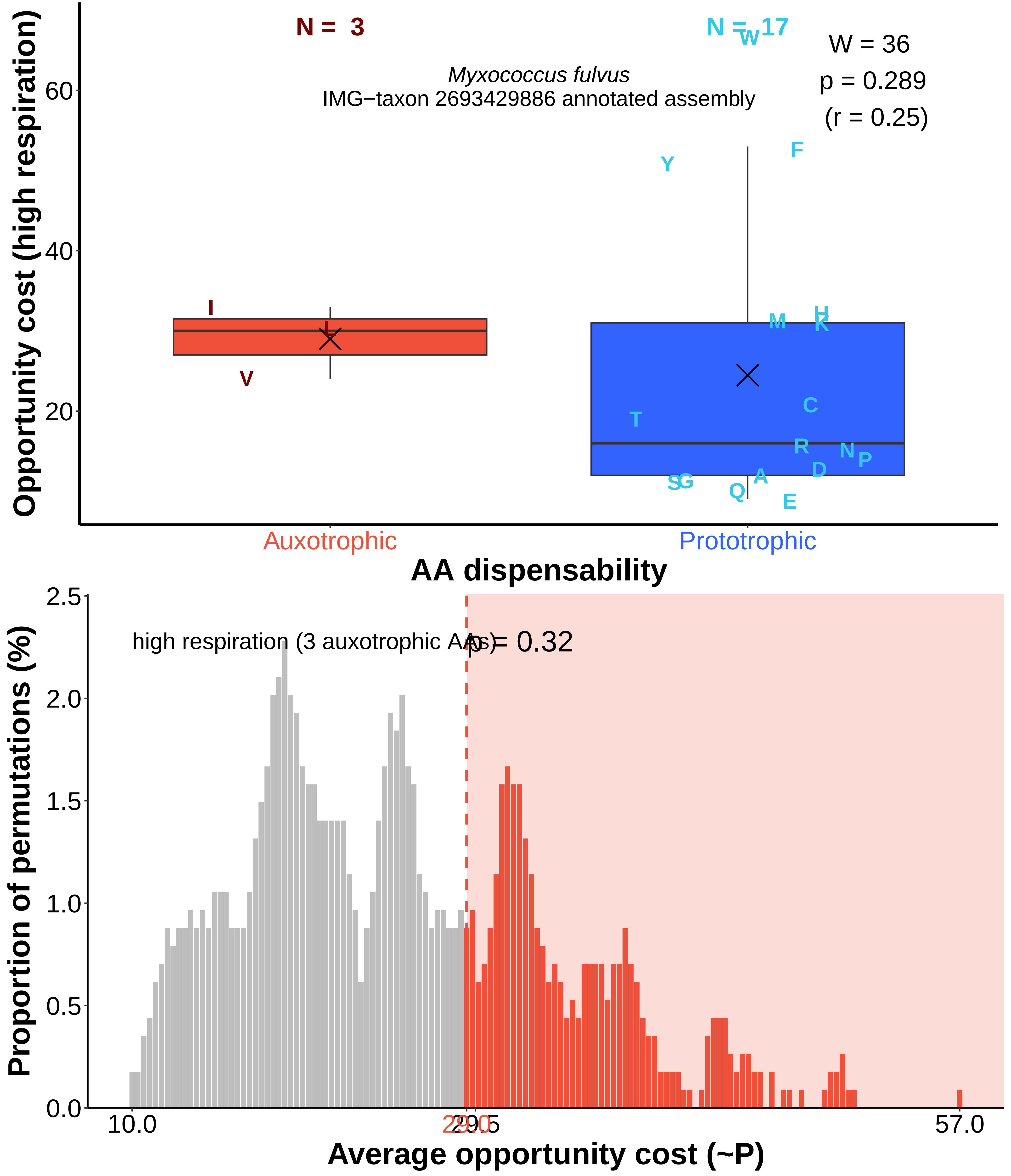

N = 3
N = 17
W = 36
p = 0.289
(r = 0.25)
Myxococcus fulvus
IMG−taxon 2693429886 annotated assembly
Opportunity cost (high respiration)
60
40
20
I
L
V
Y
F
W
M
H
K
C
T
R
N
P
D
S
G
A
Q
E
Auxotrophic
Prototrophic
AA dispensability
Proportion of permutations (%)
2.5
2.0
1.5
1.0
0.5
0.0
high respiration (3 auxotrophic AAs)
p = 0.32
10.0
29.0
29.5
57.0
Average opportunity cost (~P)

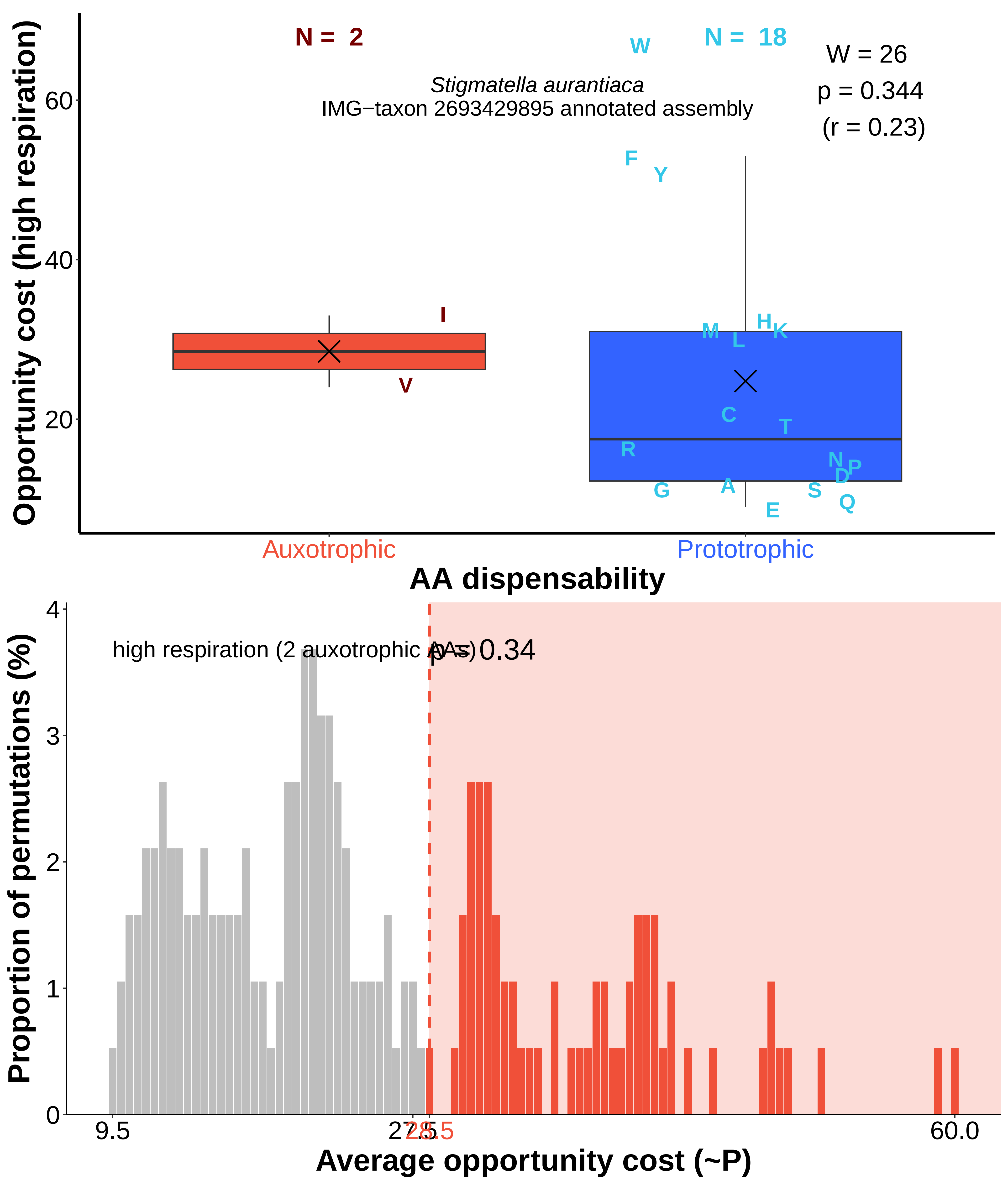

N = 2
N = 18
W = 26
p = 0.344
(r = 0.23)
Stigmatella aurantiaca
IMG−taxon 2693429895 annotated assembly
Opportunity cost (high respiration)
60
40
20
W
F
Y
I
V
M
H
K
L
C
T
R
N
P
D
G
A
S
Q
E
Auxotrophic
Prototrophic
AA dispensability
high respiration (2 auxotrophic AAs)
p = 0.34
Proportion of permutations (%)
4
3
2
1
0
9.5
27.5
28.5
60.0
Average opportunity cost (~P)

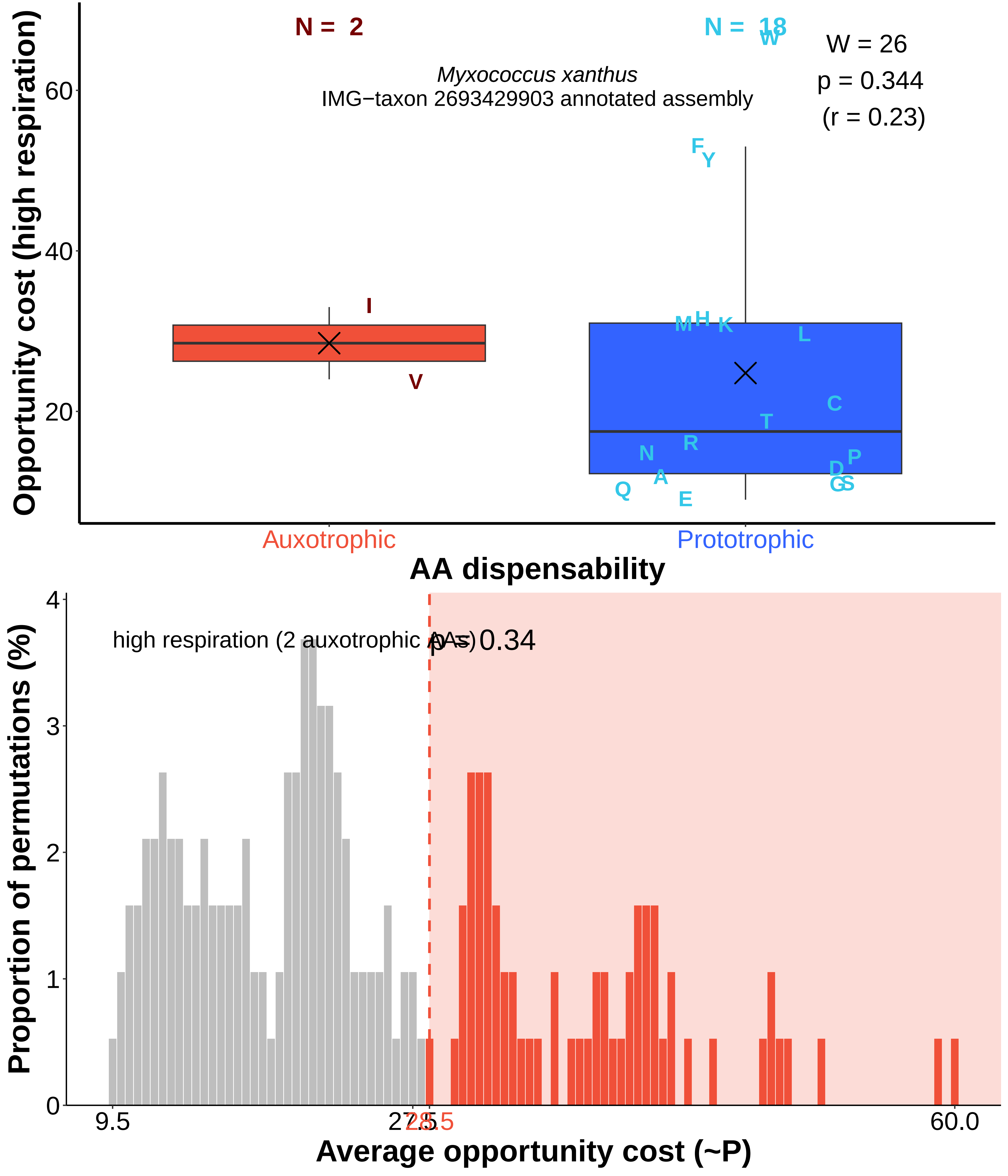

N = 2
N = 18
W = 26
p = 0.344
(r = 0.23)
Myxococcus xanthus
IMG−taxon 2693429903 annotated assembly
Opportunity cost (high respiration)
60
40
20
I
V
W
F
Y
M
H
K
L
C
T
R
N
P
D
A
G
S
Q
E
Auxotrophic
Prototrophic
AA dispensability
high respiration (2 auxotrophic AAs)
p = 0.34
Proportion of permutations (%)
4
3
2
1
0
9.5
27.5
28.5
60.0
Average opportunity cost (~P)

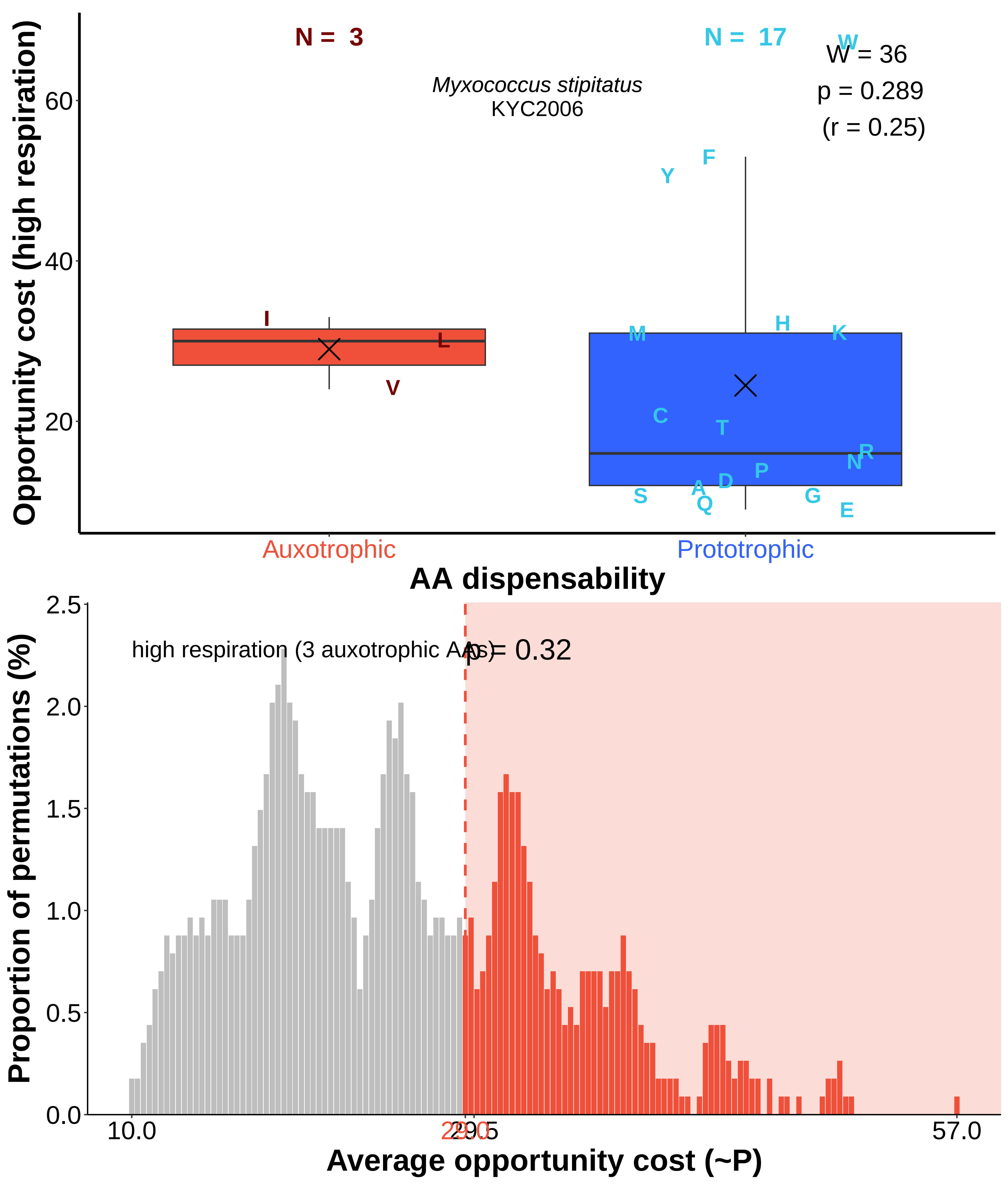
N = 3
N = 17
W = 36
p = 0.289
(r = 0.25)
Myxococcus stipitatus
KYC2006
Opportunity cost (high respiration)
Auxotrophic
Prototrophic
AA dispensability
high respiration (3 auxotrophic AAs)
p = 0.32
Proportion of permutations (%)
Average opportunity cost (~P)
10.0
29.0
57.0

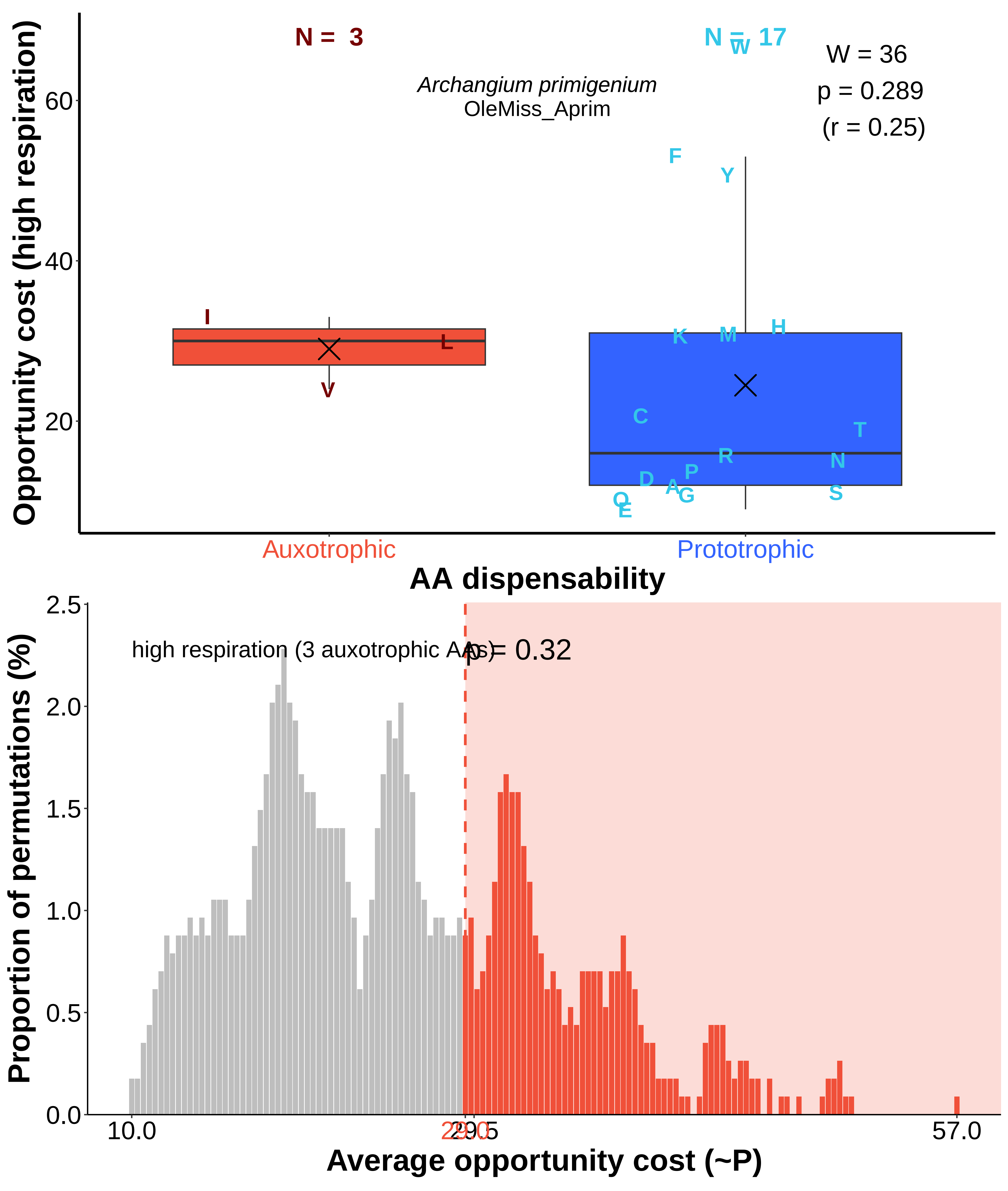

N = 3
N = 17
W
W = 36
p = 0.289
(r = 0.25)
Archangium primigenium
OleMiss_Aprim
Opportunity cost (high respiration)
60
40
20
I
L
V
F
Y
K
M
H
C
T
R
N
P
D
A
G
Q
E
S
Auxotrophic
Prototrophic
AA dispensability
Proportion of permutations (%)
2.5
2.0
1.5
1.0
0.5
0.0
high respiration (3 auxotrophic AAs)
p = 0.32
10.0
29.0
29.5
57.0
Average opportunity cost (~P)

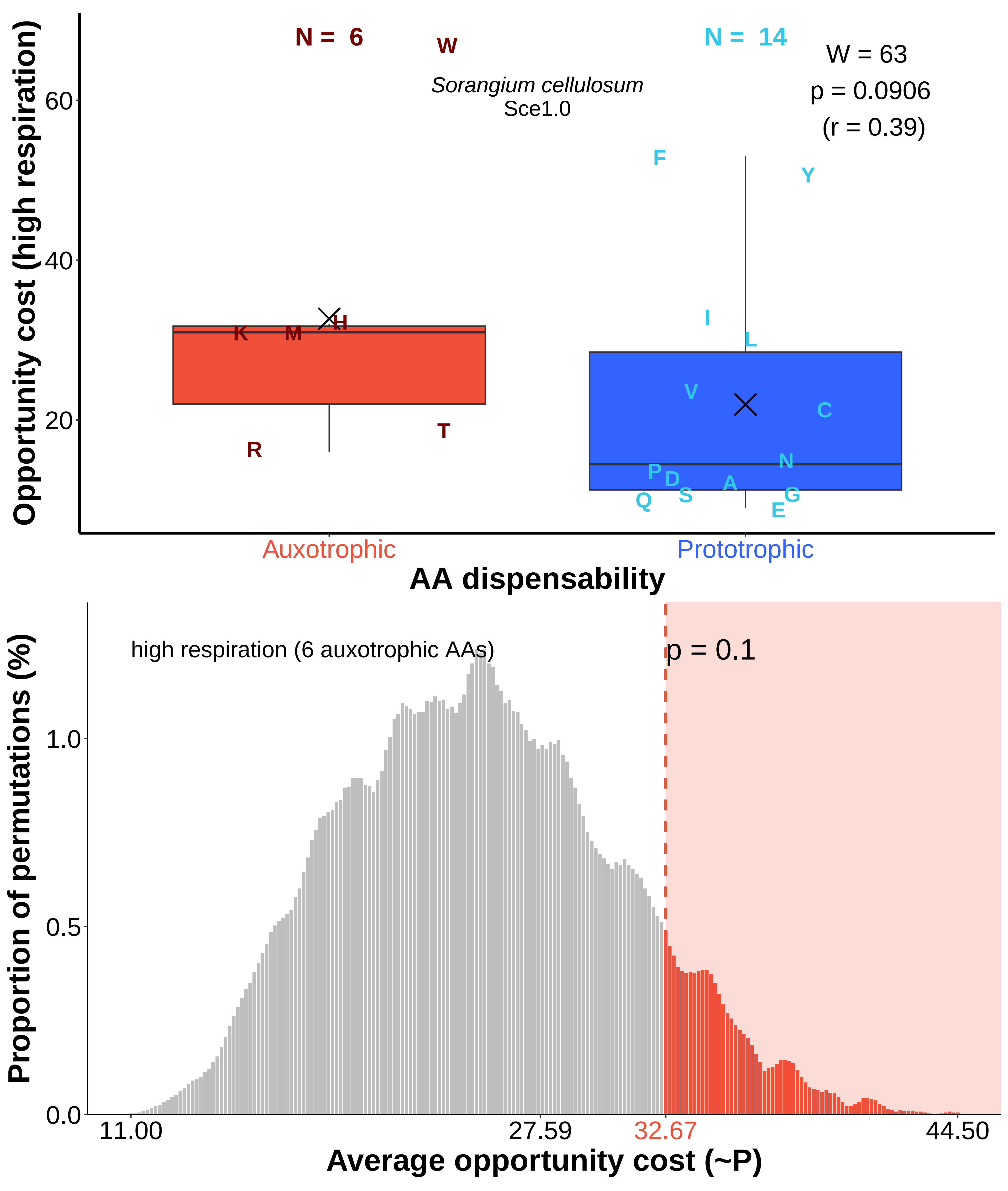

N = 6
W
N = 14
W = 63
p = 0.0906
(r = 0.39)
Sorangium cellulosum
Sce1.0
F
Y
I
L
K
M
H
V
C
T
R
N
P
D
S
A
G
Q
E
Opportunity cost (high respiration)
60
40
20
Auxotrophic
Prototrophic
AA dispensability
high respiration (6 auxotrophic AAs)
p = 0.1
Proportion of permutations (%)
1.0
0.5
0.0
11.00
27.59
32.67
44.50
Average opportunity cost (~P)

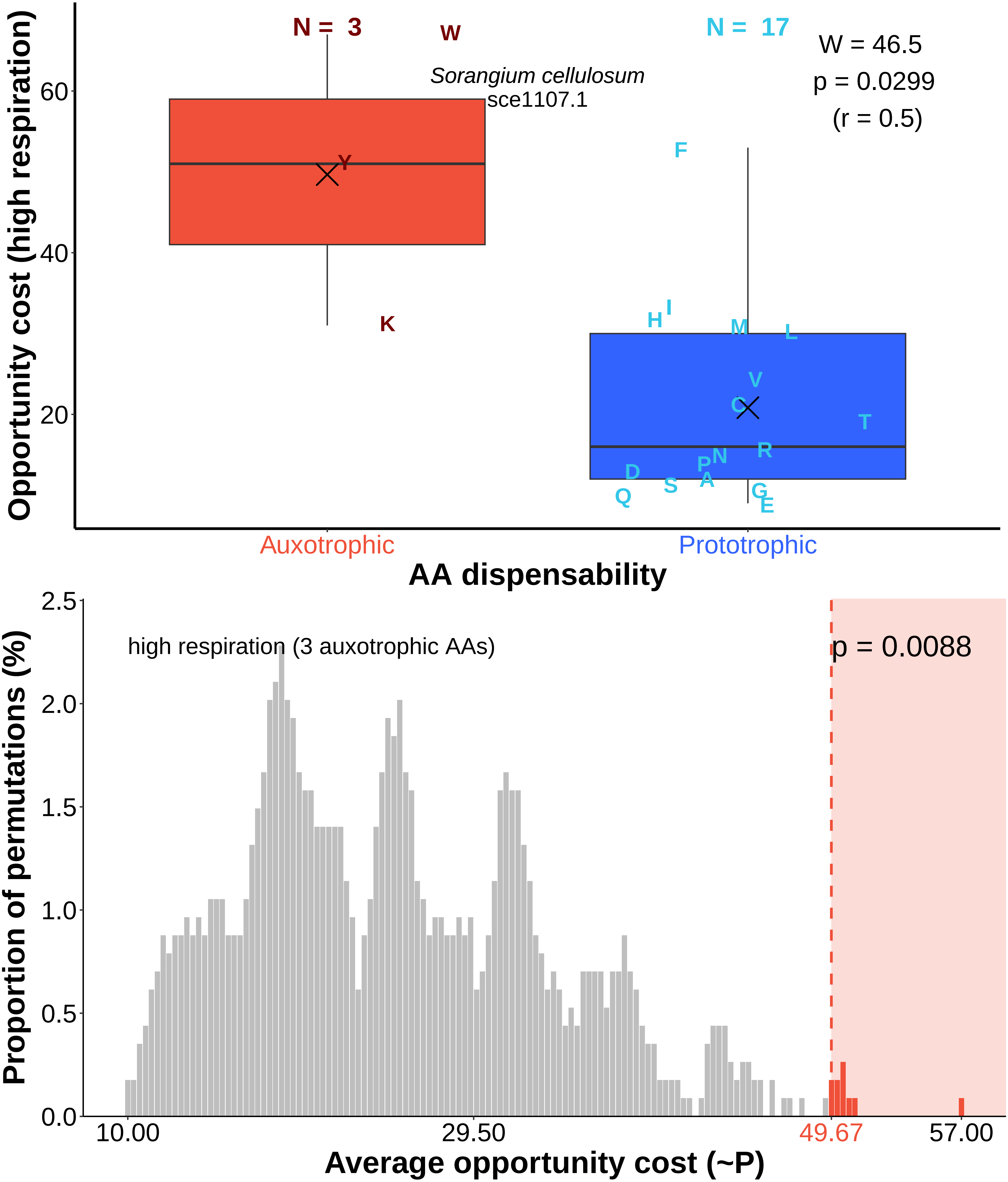

N = 3
N = 17
W = 46.5
p = 0.0299
(r = 0.5)
Sorangium cellulosum
sce1107.1
W
Y
K
F
H
I
M
L
V
C
T
R
N
P
D
A
S
Q
G
E
60
40
20
Opportunity cost (high respiration)
Auxotrophic
Prototrophic
AA dispensability
high respiration (3 auxotrophic AAs)
p = 0.0088
2.5
2.0
1.5
1.0
0.5
0.0
Proportion of permutations (%)
10.00
29.50
49.67
57.00
Average opportunity cost (~P)

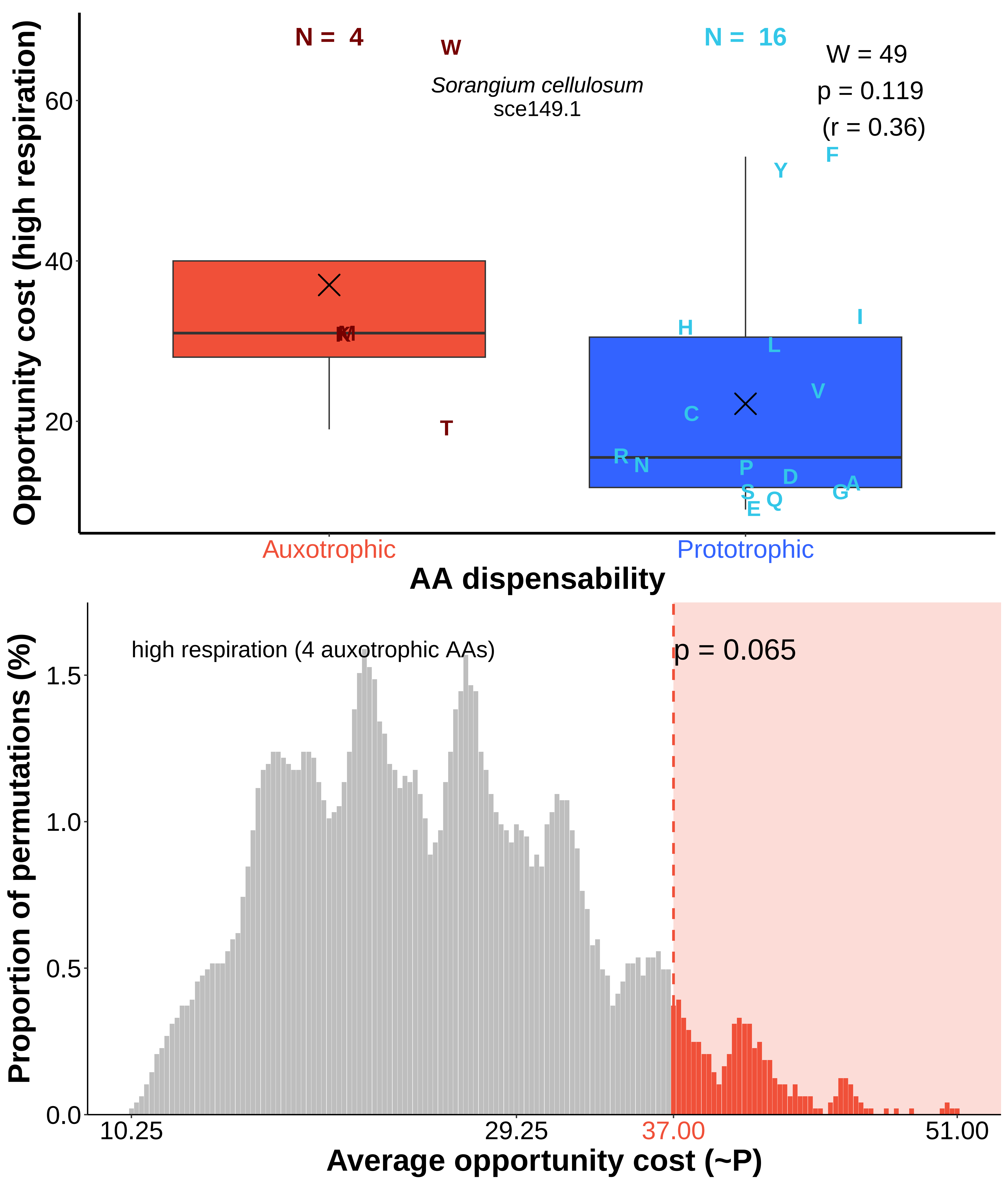

N = 4
N = 16
W = 49
p = 0.119
(r = 0.36)
Sorangium cellulosum
sce149.1
Opportunity cost (high respiration)
Auxotrophic
Prototrophic
AA dispensability
W T K M
H L C V I Y F R N P D S Q E G A
60
40
20
high respiration (4 auxotrophic AAs)
p = 0.065
Proportion of permutations (%)
1.5
1.0
0.5
0.0
10.25
29.25
37.00
51.00
Average opportunity cost (~P)

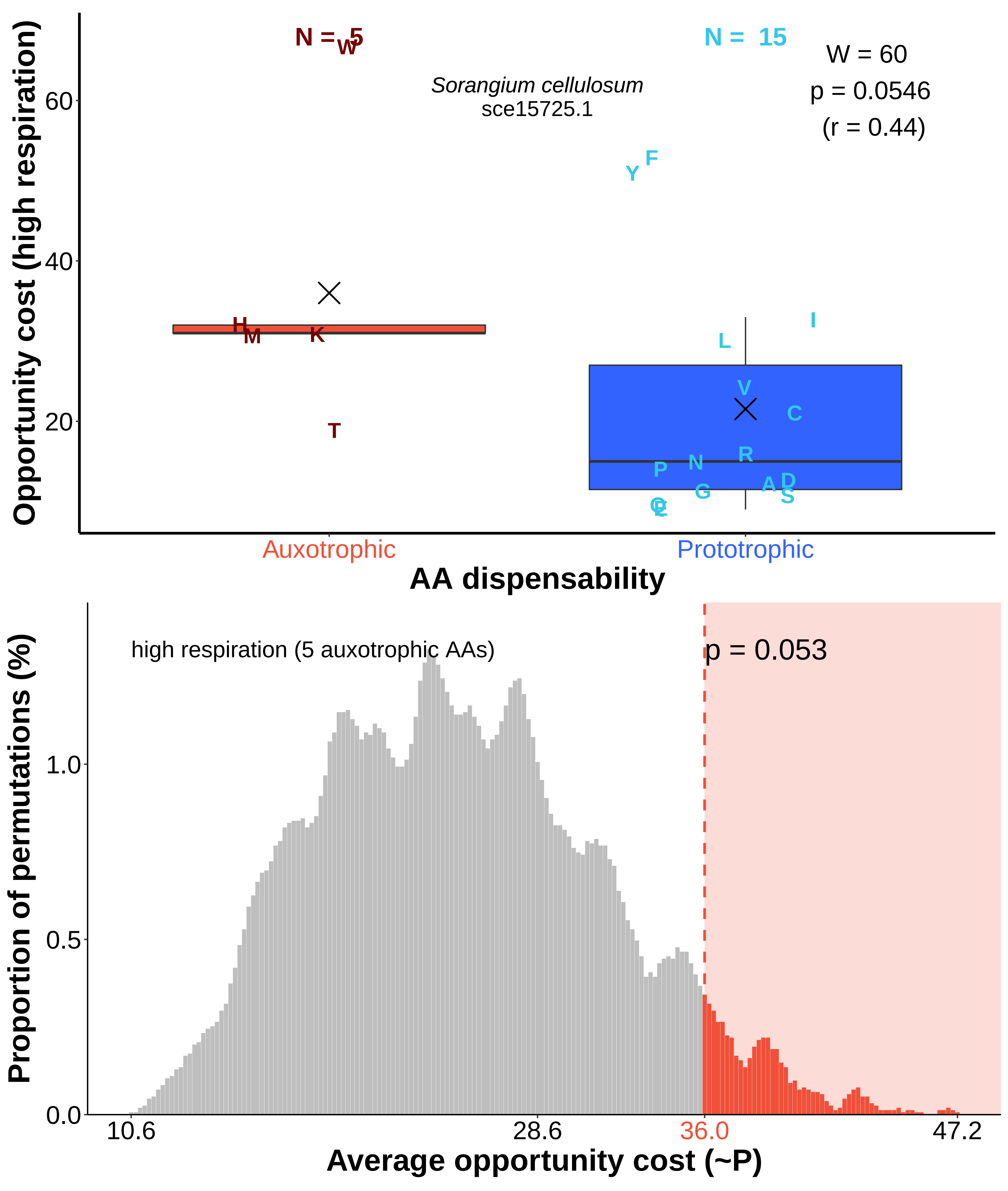

N = 5
N = 15
W = 60
p = 0.0546
(r = 0.44)
Sorangium cellulosum
sce15725.1
Opportunity cost (high respiration)
60
40
20
Auxotrophic
Prototrophic
AA dispensability
high respiration (5 auxotrophic AAs)
p = 0.053
Proportion of permutations (%)
1.0
0.5
0.0
10.6
28.6
36.0
47.2
Average opportunity cost (~P)

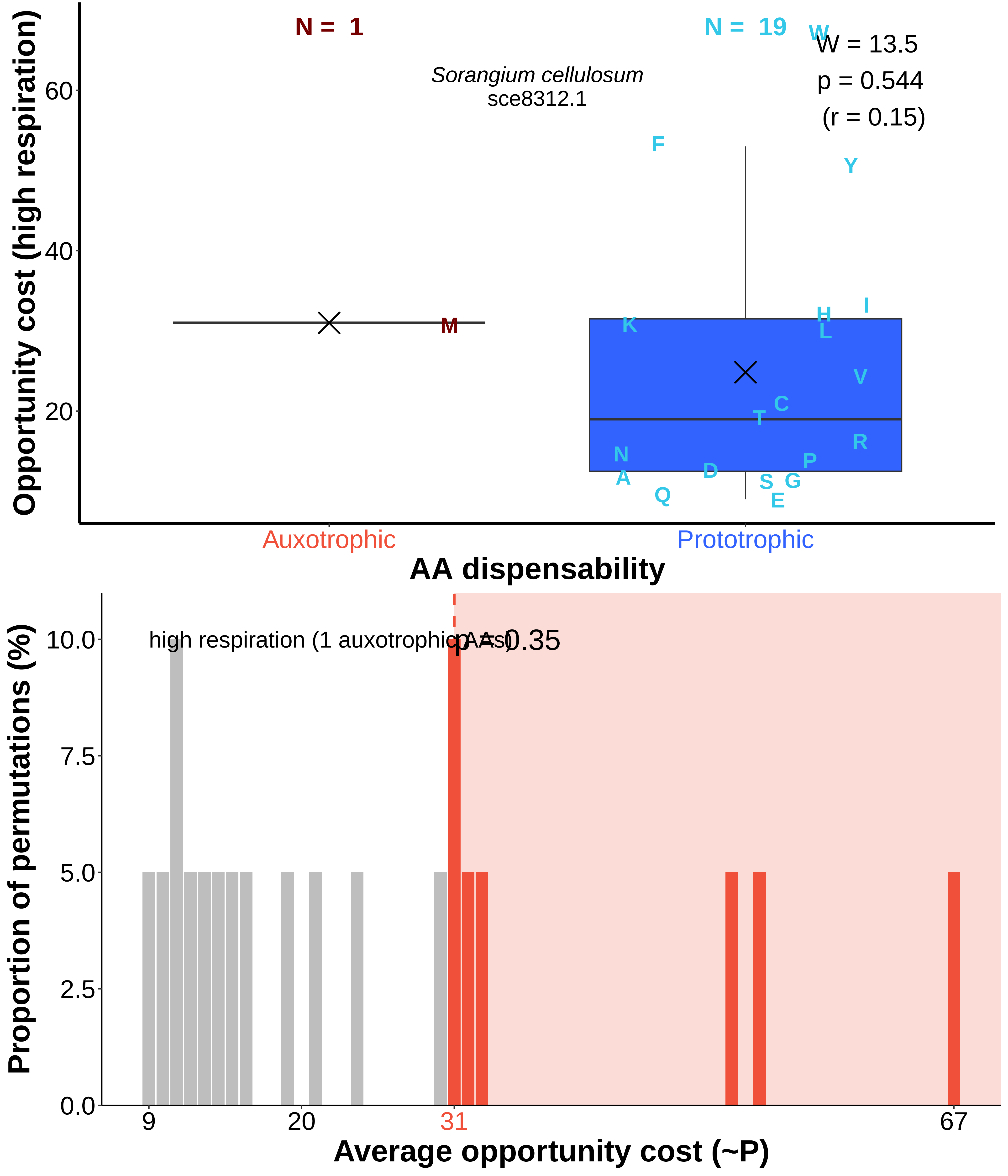
N = 1
N = 19
W = 13.5
p = 0.544
(r = 0.15)
Sorangium cellulosum
sce8312.1
Opportunity cost (high respiration)
60
40
20
F
Y
W
I
H
L
K
M
V
C
T
R
N
P
D
A
S
G
Q
E
Auxotrophic
Prototrophic
AA dispensability
Proportion of permutations (%)
10.0
7.5
5.0
2.5
0.0
high respiration (1 auxotrophic AAs)
p = 0.35
9
20
31
67
Average opportunity cost (~P)

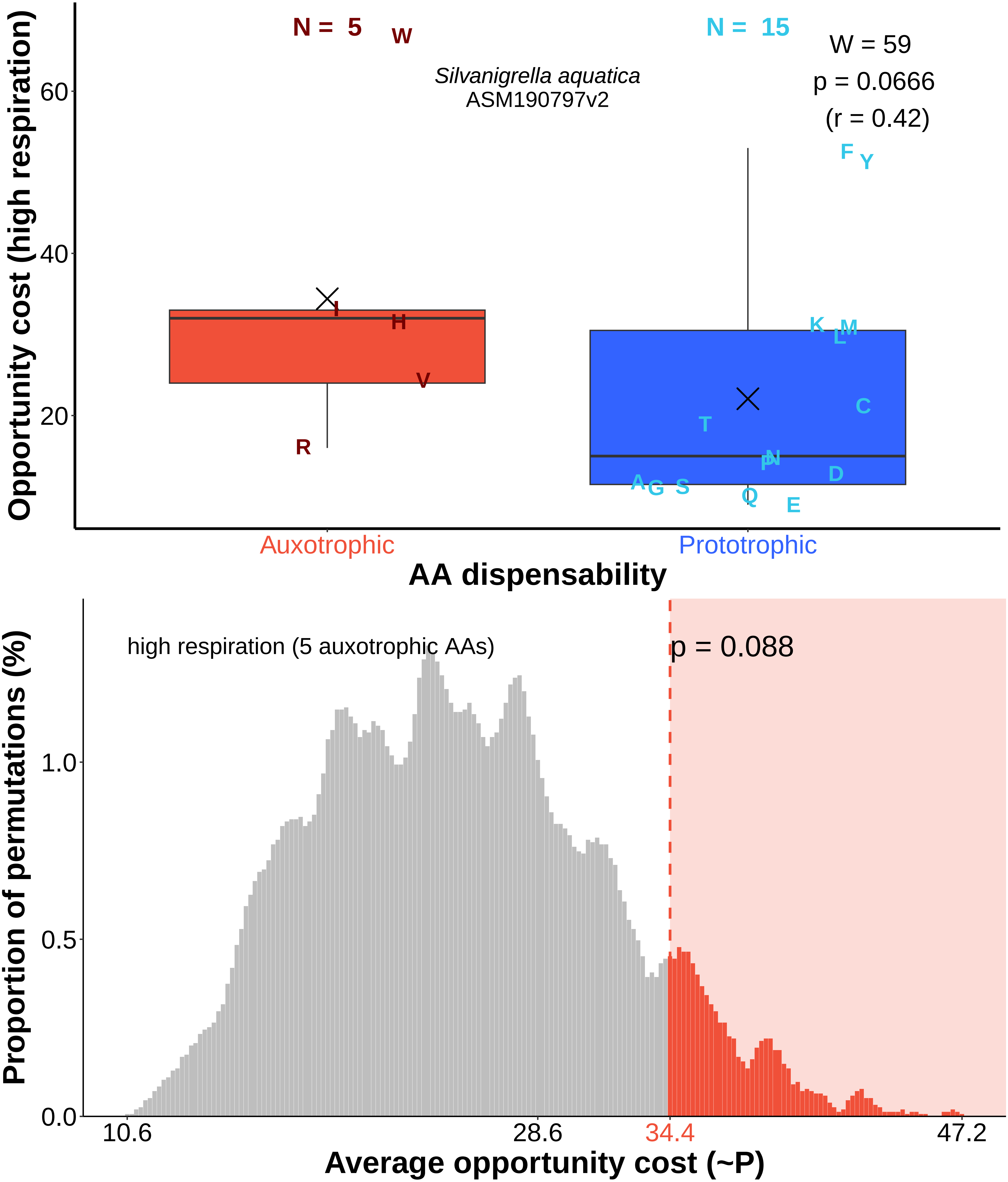
N = 5
W
N = 15
W = 59
p = 0.0666
(r = 0.42)
Silvanigrella aquatica
ASM190797v2
F
Y
I
H
V
R
K
M
L
C
T
N
P
D
A
G
S
Q
E
60
40
20
Opportunity cost (high respiration)
Auxotrophic
Prototrophic
AA dispensability
high respiration (5 auxotrophic AAs)
p = 0.088
1.0
0.5
0.0
Proportion of permutations (%)
10.6
28.6
34.4
47.2
Average opportunity cost (~P)

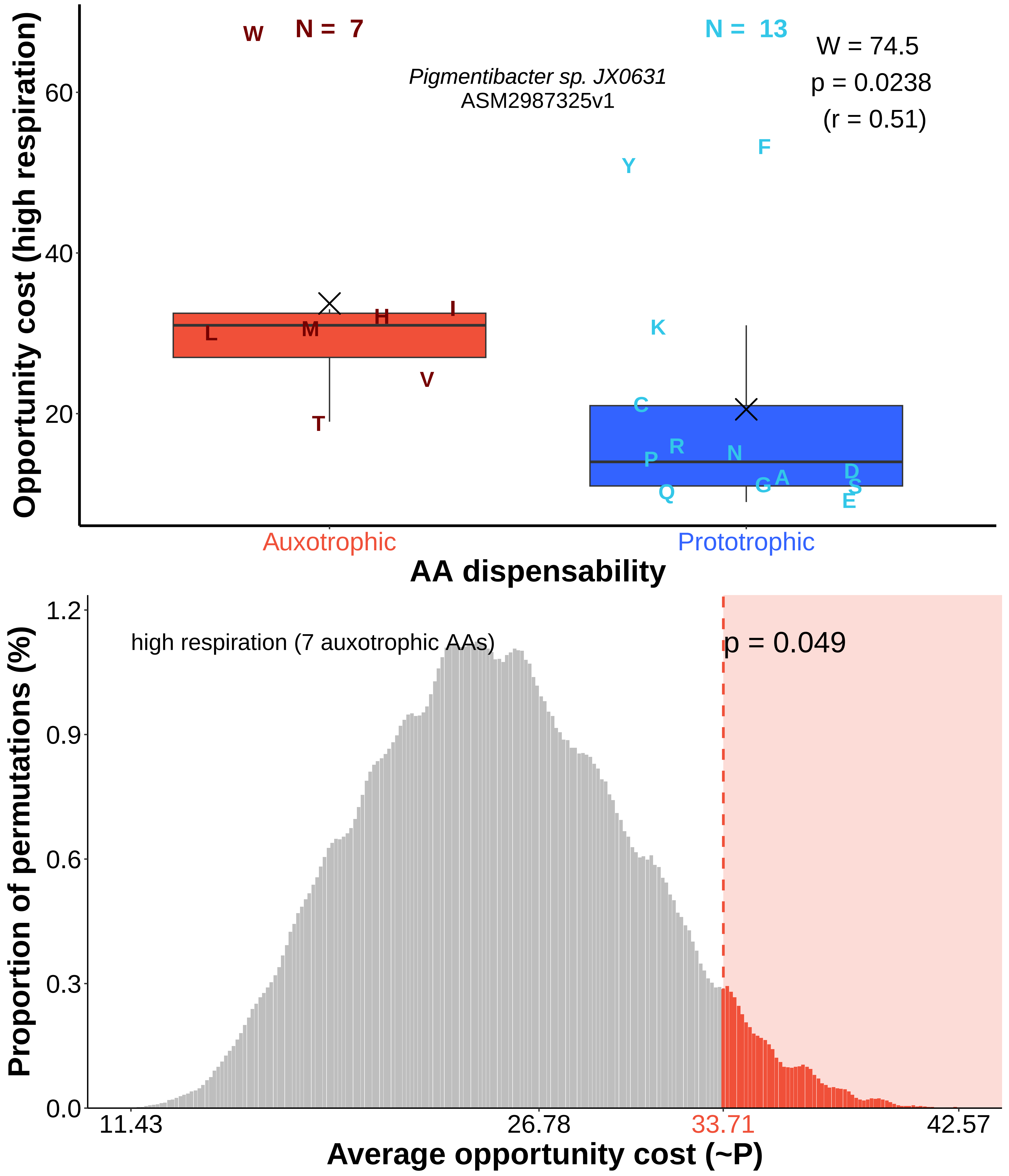

W
N = 7
N = 13
W = 74.5
p = 0.0238
(r = 0.51)
Pigmentibacter sp. JX0631
ASM2987325v1
Opportunity cost (high respiration)
60
40
20
F
Y
K
C
R
P
N
Q
G
A
D
S
E
L
M
H
I
V
T
Auxotrophic
Prototrophic
AA dispensability
Proportion of permutations (%)
1.2
0.9
0.6
0.3
0.0
high respiration (7 auxotrophic AAs)
p = 0.049
11.43
26.78
33.71
42.57
Average opportunity cost (~P)

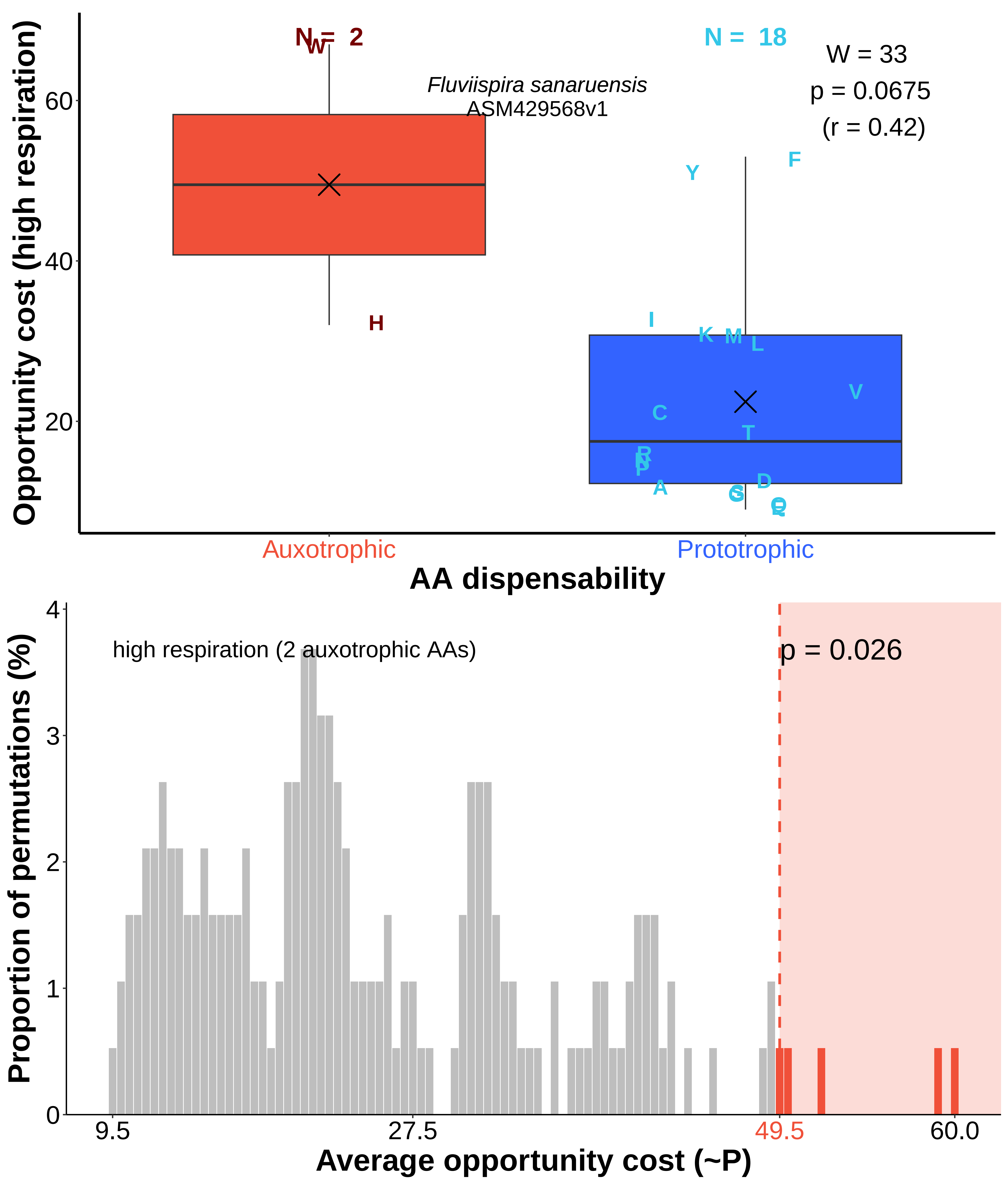

N = 2
W
N = 18
W = 33
p = 0.0675
(r = 0.42)
Fluviispira sanaruensis
ASM429568v1
H
Y
F
I
K
M
L
V
C
T
R
N
P
A
G
S
D
Q
E
60
40
20
Opportunity cost (high respiration)
Auxotrophic
Prototrophic
AA dispensability
high respiration (2 auxotrophic AAs)
p = 0.026
4
3
2
1
0
Proportion of permutations (%)
9.5
27.5
49.5
60.0
Average opportunity cost (~P)

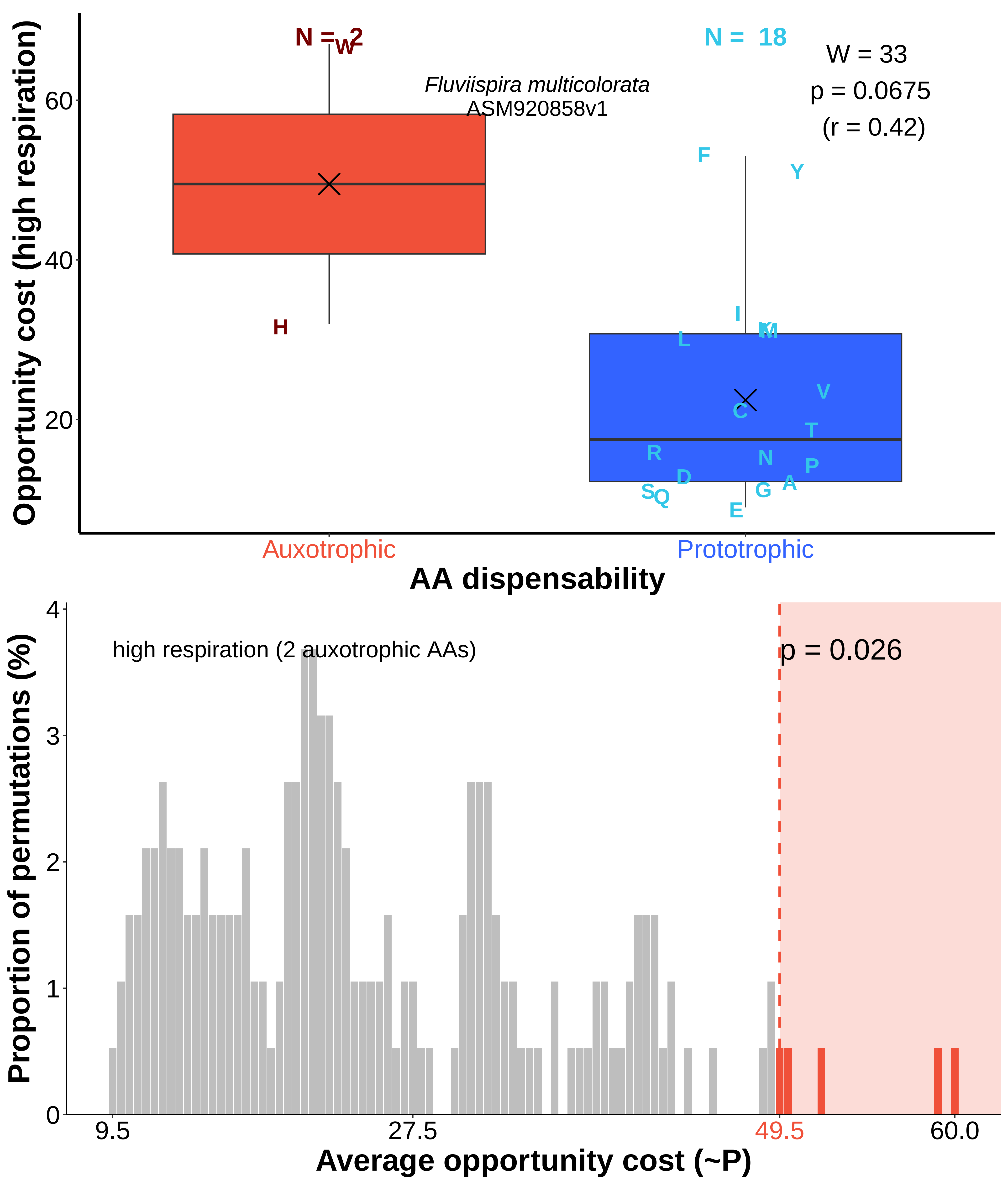

N = 2
W
N = 18
W = 33
p = 0.0675
(r = 0.42)
Fluviispira multicolorata
ASM920858v1
60
40
20
F
Y
I
L
M
V
C
T
R
N
P
D
A
S
Q
G
E
H
Opportunity cost (high respiration)
Auxotrophic
Prototrophic
AA dispensability
high respiration (2 auxotrophic AAs)
p = 0.026
Proportion of permutations (%)
4
3
2
1
0
9.5
27.5
49.5
60.0
Average opportunity cost (~P)

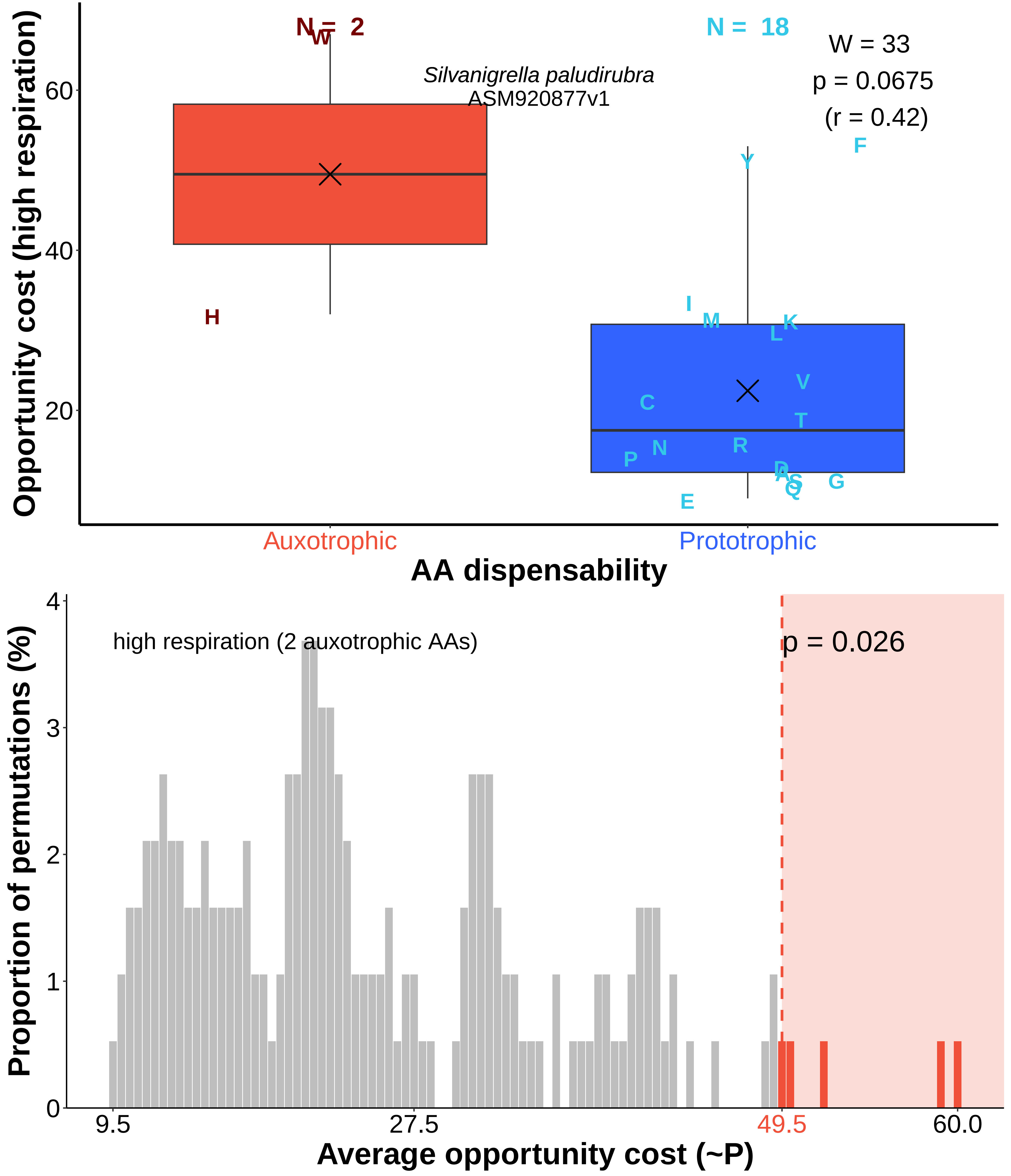

N = 2
N = 18
W = 33
p = 0.0675
(r = 0.42)
Silvanigrella paludirubra
ASM920877v1
W
H
Y
F
I
M
K
L
V
C
T
R
N
P
D
A
S
Q
G
E
Opportunity cost (high respiration)
60
40
20
Auxotrophic
Prototrophic
AA dispensability
high respiration (2 auxotrophic AAs)
p = 0.026
Proportion of permutations (%)
4
3
2
1
0
9.5
27.5
49.5
60.0
Average opportunity cost (~P)

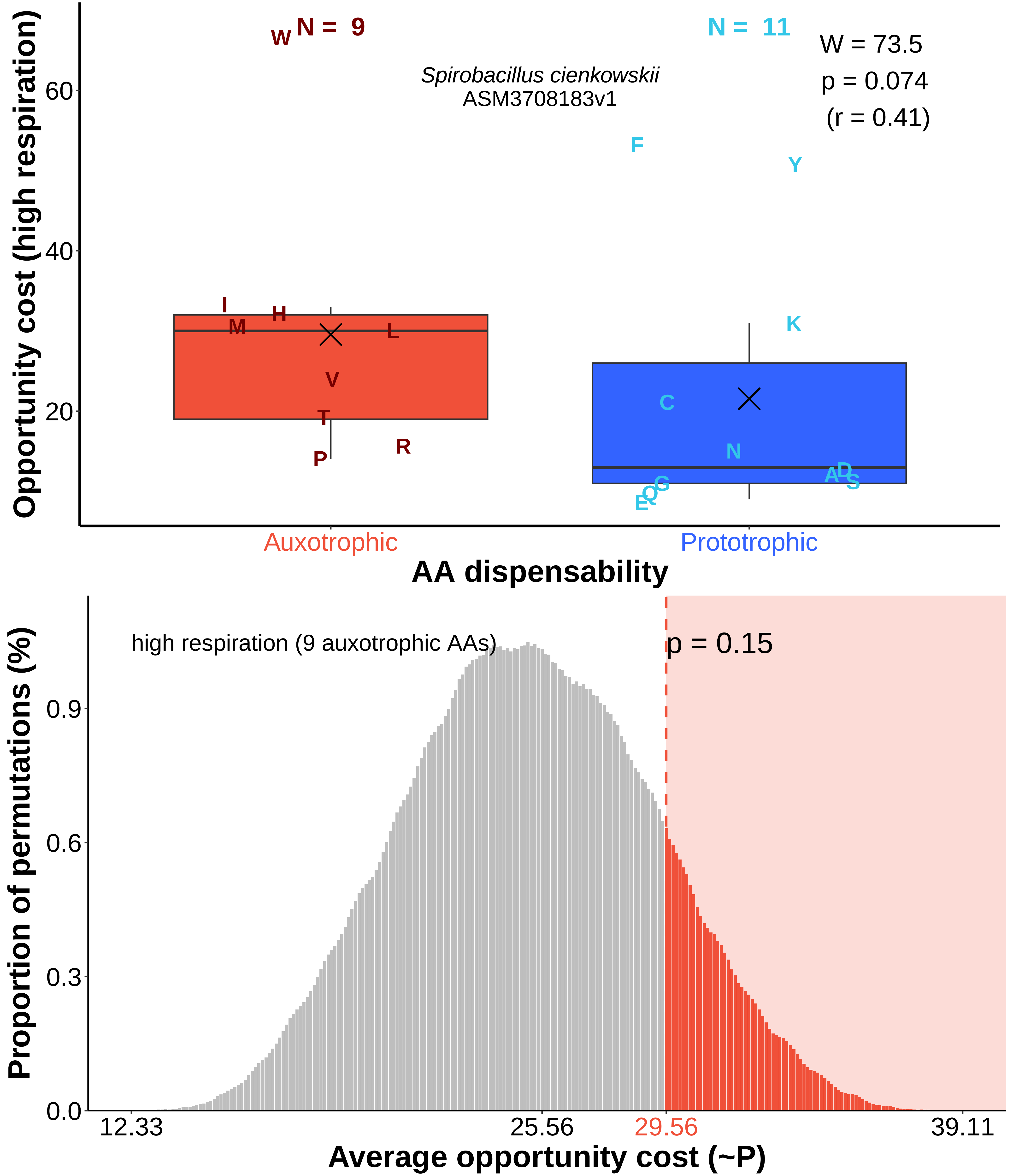

N = 9
N = 11
W = 73.5
p = 0.074
(r = 0.41)
Spirobacillus cienkowskii
ASM3708183v1
Opportunity cost (high respiration)
Auxotrophic
Prototrophic
AA dispensability
high respiration (9 auxotrophic AAs)
p = 0.15
Proportion of permutations (%)
12.33
25.56
29.56
39.11
Average opportunity cost (~P)

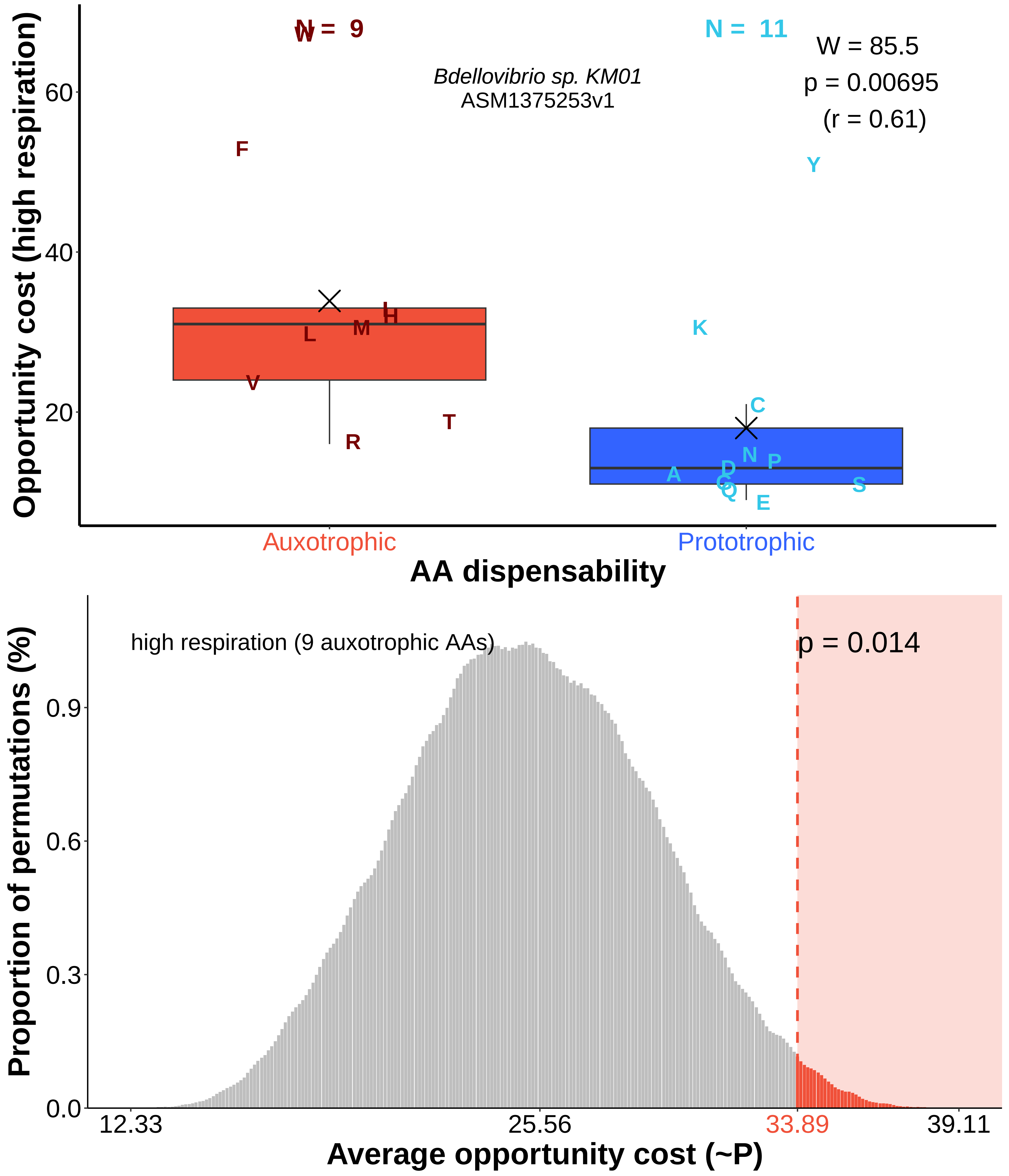
N = 9
W
N = 11
W = 85.5
p = 0.00695
(r = 0.61)
Bdellovibrio sp. KM01
ASM1375253v1
F
Y
I
H
M
L
K
V
C
T
R
N
P
D
A
G
Q
S
E
Opportunity cost (high respiration)
60
40
20
Auxotrophic
Prototrophic
AA dispensability
high respiration (9 auxotrophic AAs)
p = 0.014
Proportion of permutations (%)
0.9
0.6
0.3
0.0
12.33
25.56
33.89
39.11
Average opportunity cost (~P)

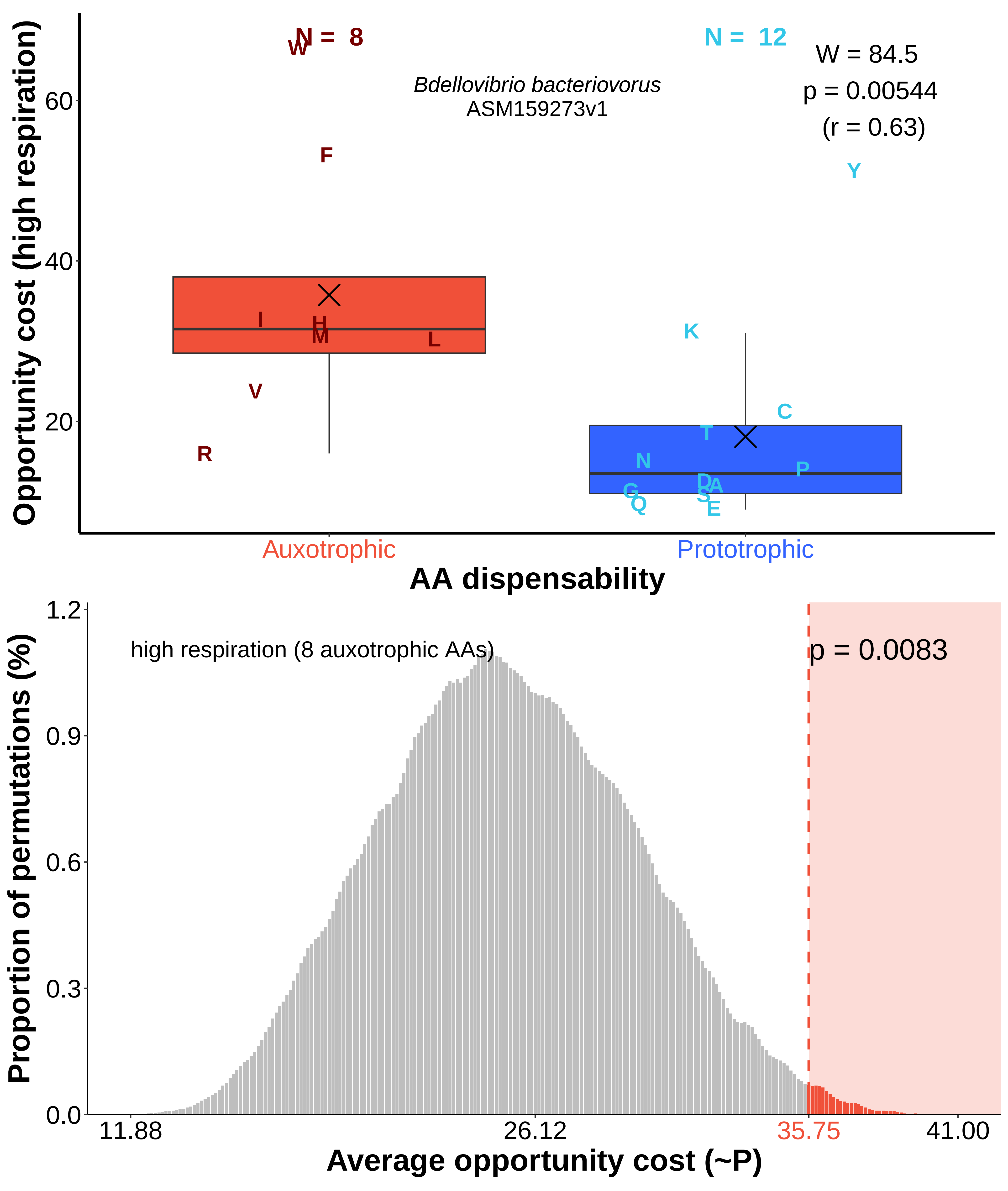

N = 8
N = 12
W = 84.5
p = 0.00544
(r = 0.63)
Bdellovibrio bacteriovorus
ASM159273v1
W
F
I
H
M
L
V
R
Y
K
C
T
N
P
D
A
G
S
Q
E
60
40
20
Opportunity cost (high respiration)
Auxotrophic
Prototrophic
AA dispensability
high respiration (8 auxotrophic AAs)
p = 0.0083
1.2
0.9
0.6
0.3
0.0
Proportion of permutations (%)
11.88
26.12
35.75
41.00
Average opportunity cost (~P)

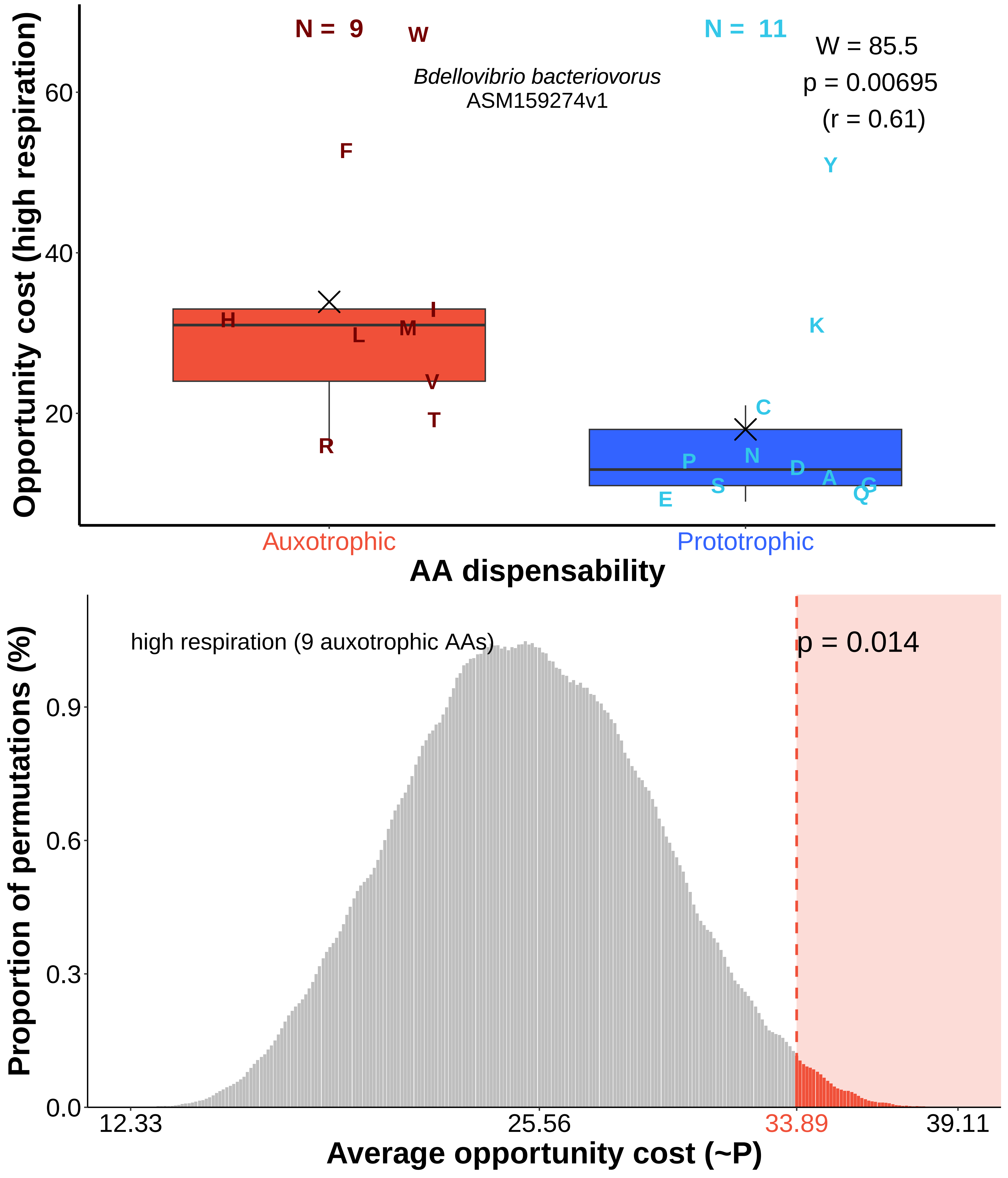

N = 9
N = 11
Bdellovibrio bacteriovorus
ASM159274v1
W = 85.5
p = 0.00695
(r = 0.61)
Opportunity cost (high respiration)
Auxotrophic
Prototrophic
AA dispensability
high respiration (9 auxotrophic AAs)
p = 0.014
Proportion of permutations (%)
12.33
25.56
33.89
39.11
Average opportunity cost (~P)

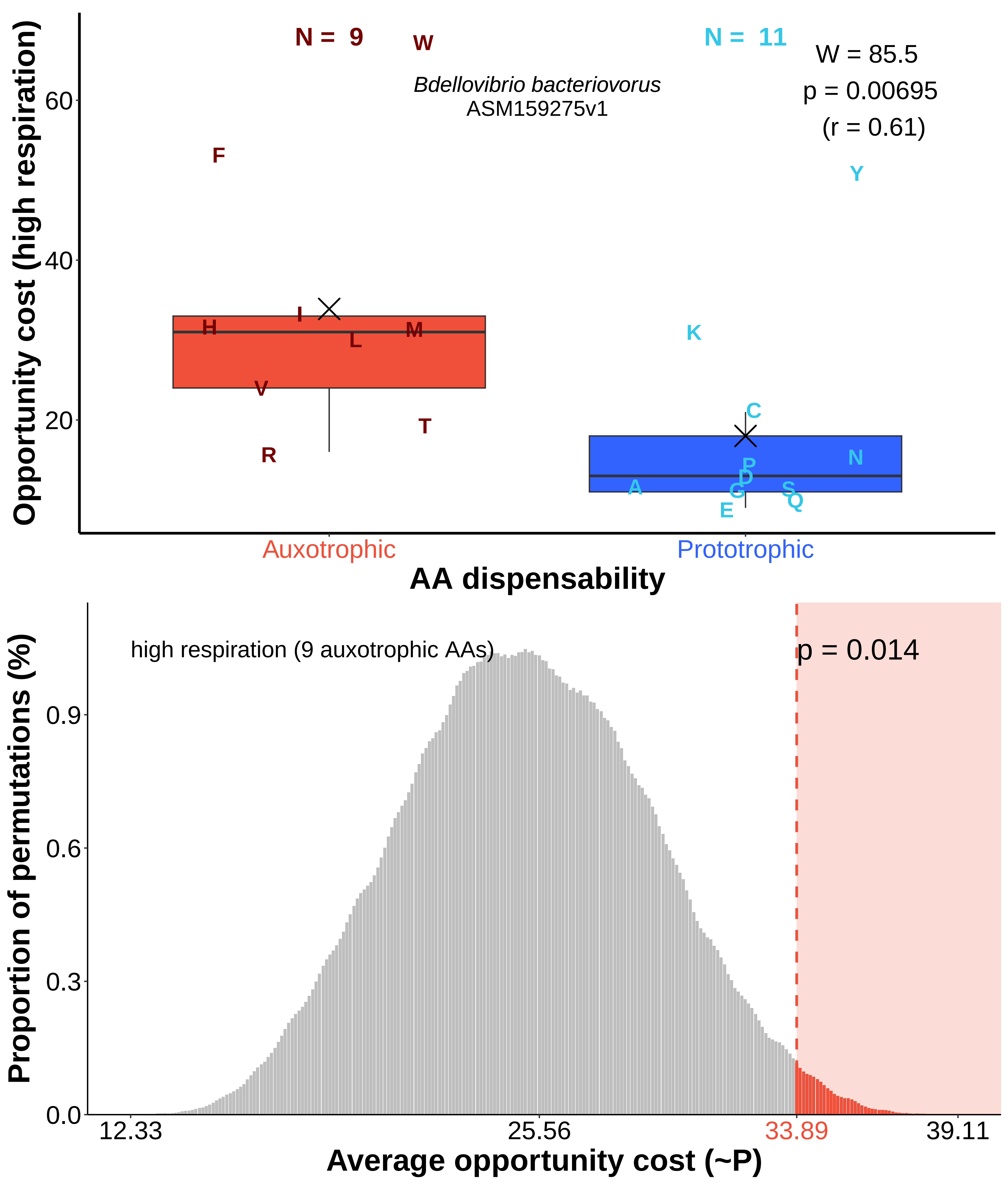

N = 9
N = 11
W = 85.5
p = 0.00695
(r = 0.61)
Bdellovibrio bacteriovorus
ASM159275v1
Opportunity cost (high respiration)
60
40
20
W
F
Y
I
H
L
M
K
V
T
R
C
P
N
D
A
G
S
Q
E
Auxotrophic
Prototrophic
AA dispensability
high respiration (9 auxotrophic AAs)
p = 0.014
Proportion of permutations (%)
0.9
0.6
0.3
0.0
12.33
25.56
33.89
39.11
Average opportunity cost (~P)

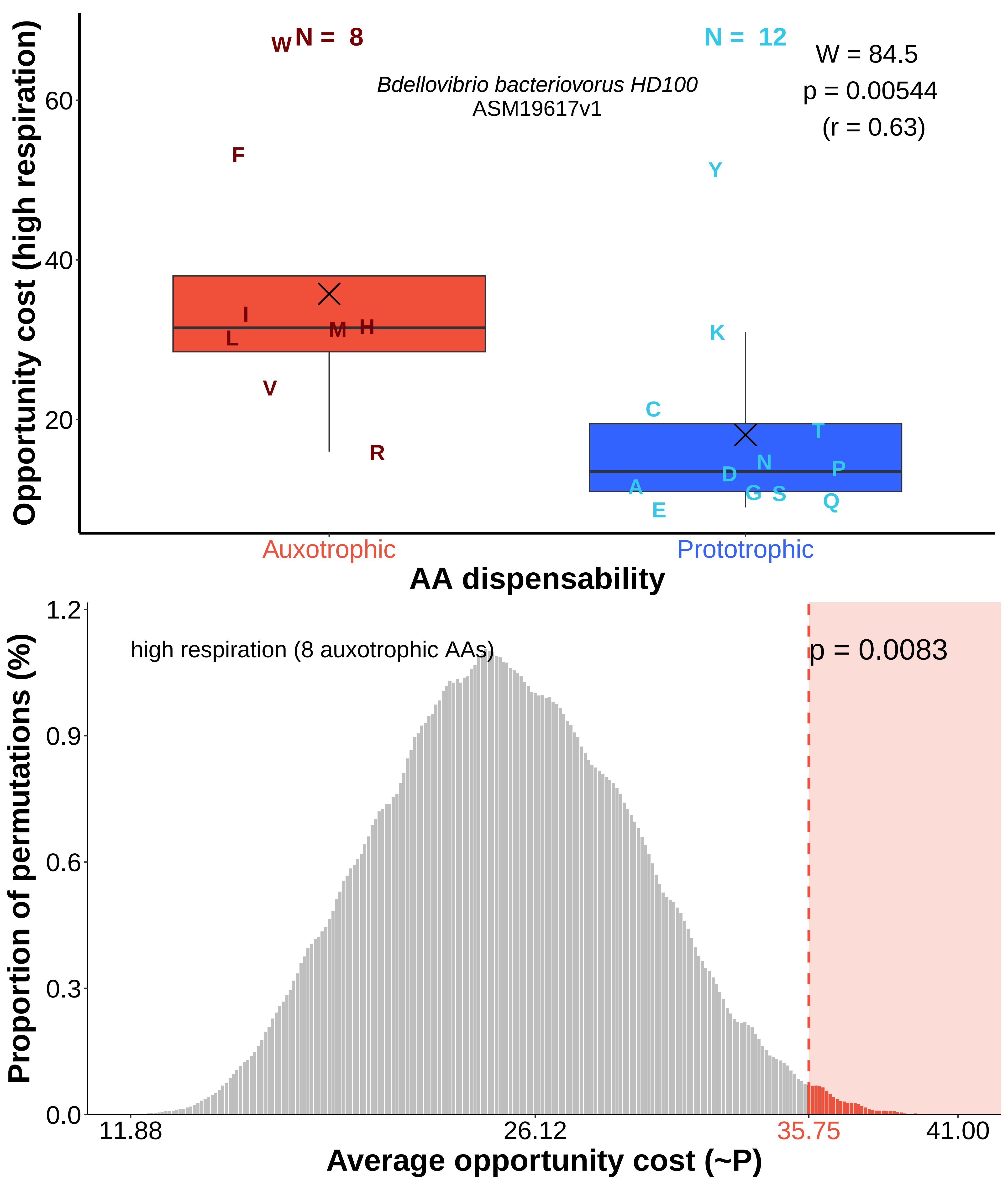

W
N = 8
N = 12
W = 84.5
p = 0.00544
(r = 0.63)
Bdellovibrio bacteriovorus HD100
ASM19617v1
F
Y
I
M
H
L
K
V
C
T
R
N
D
P
A
G
S
Q
E
60
40
20
Opportunity cost (high respiration)
Auxotrophic
Prototrophic
AA dispensability
1.2
0.9
0.6
0.3
0.0
high respiration (8 auxotrophic AAs)
p = 0.0083
Proportion of permutations (%)
11.88
26.12
35.75
41.00
Average opportunity cost (~P)

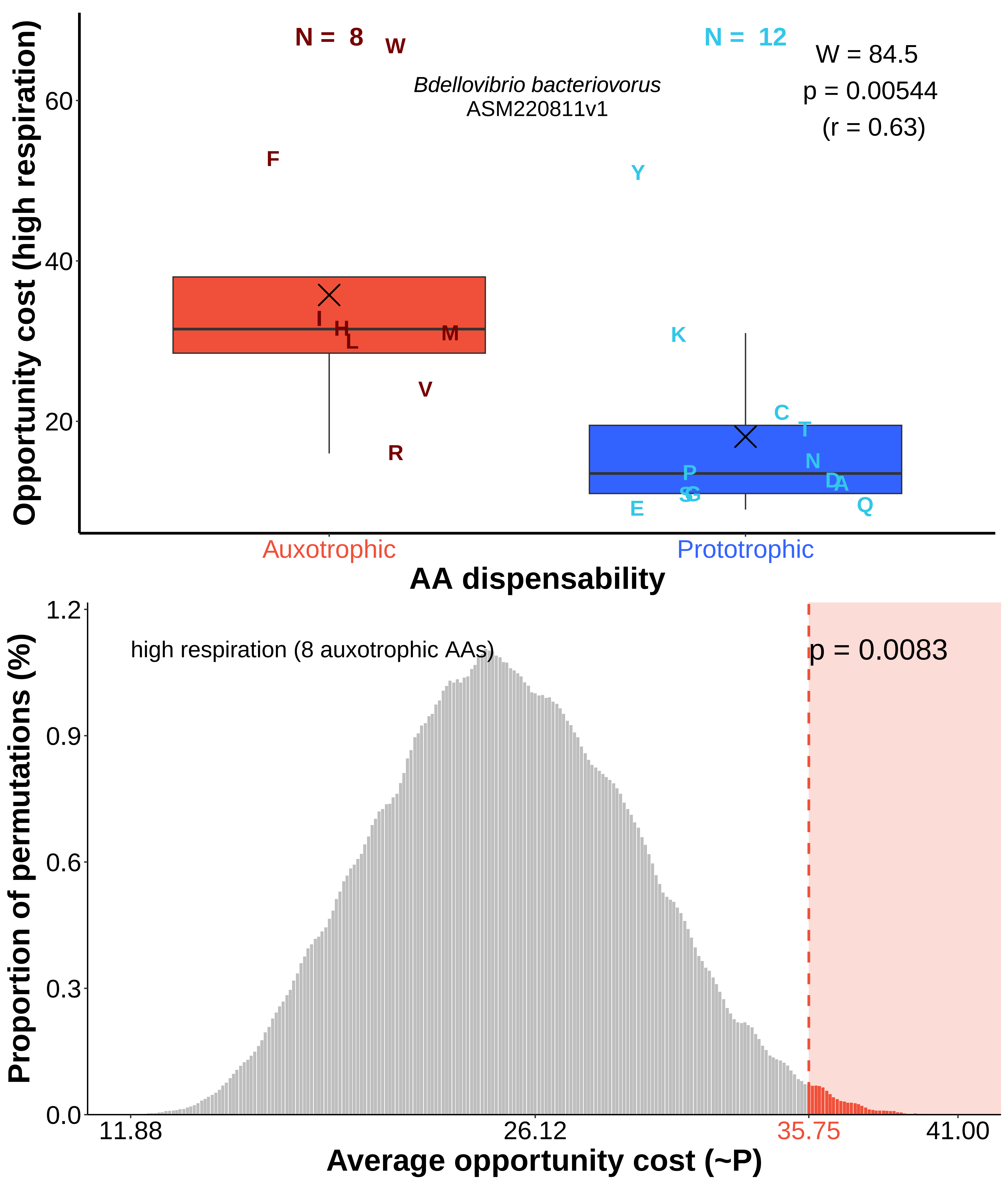
N = 8
W
N = 12
W = 84.5
p = 0.00544
(r = 0.63)
Bdellovibrio bacteriovorus
ASM220811v1
F
Y
I
H
L
M
K
V
C
T
R
N
P
D
A
S
G
E
Q
60
40
20
Opportunity cost (high respiration)
Auxotrophic
Prototrophic
AA dispensability
1.2
0.9
0.6
0.3
0.0
high respiration (8 auxotrophic AAs)
p = 0.0083
Proportion of permutations (%)
11.88
26.12
35.75
41.00
Average opportunity cost (~P)

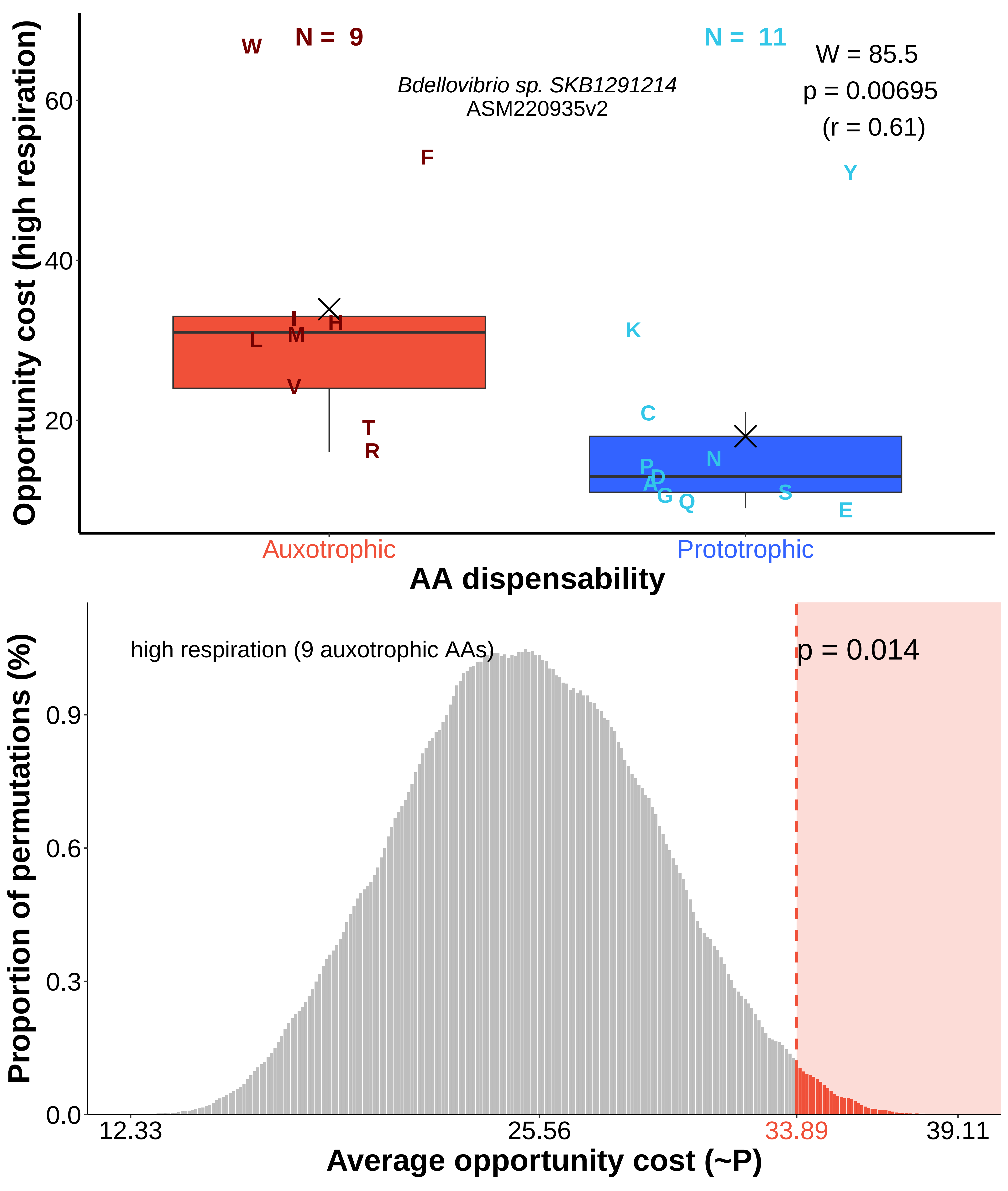

W
N = 9
N = 11
W = 85.5
p = 0.00695
(r = 0.61)
Bdellovibrio sp. SKB1291214
ASM220935v2
F
Y
I
H
M
L
V
T
R
K
C
N
P
D
A
G
Q
S
E
60
40
20
Opportunity cost (high respiration)
Auxotrophic
Prototrophic
AA dispensability
high respiration (9 auxotrophic AAs)
p = 0.014
0.9
0.6
0.3
0.0
Proportion of permutations (%)
12.33
25.56
33.89
39.11
Average opportunity cost (~P)

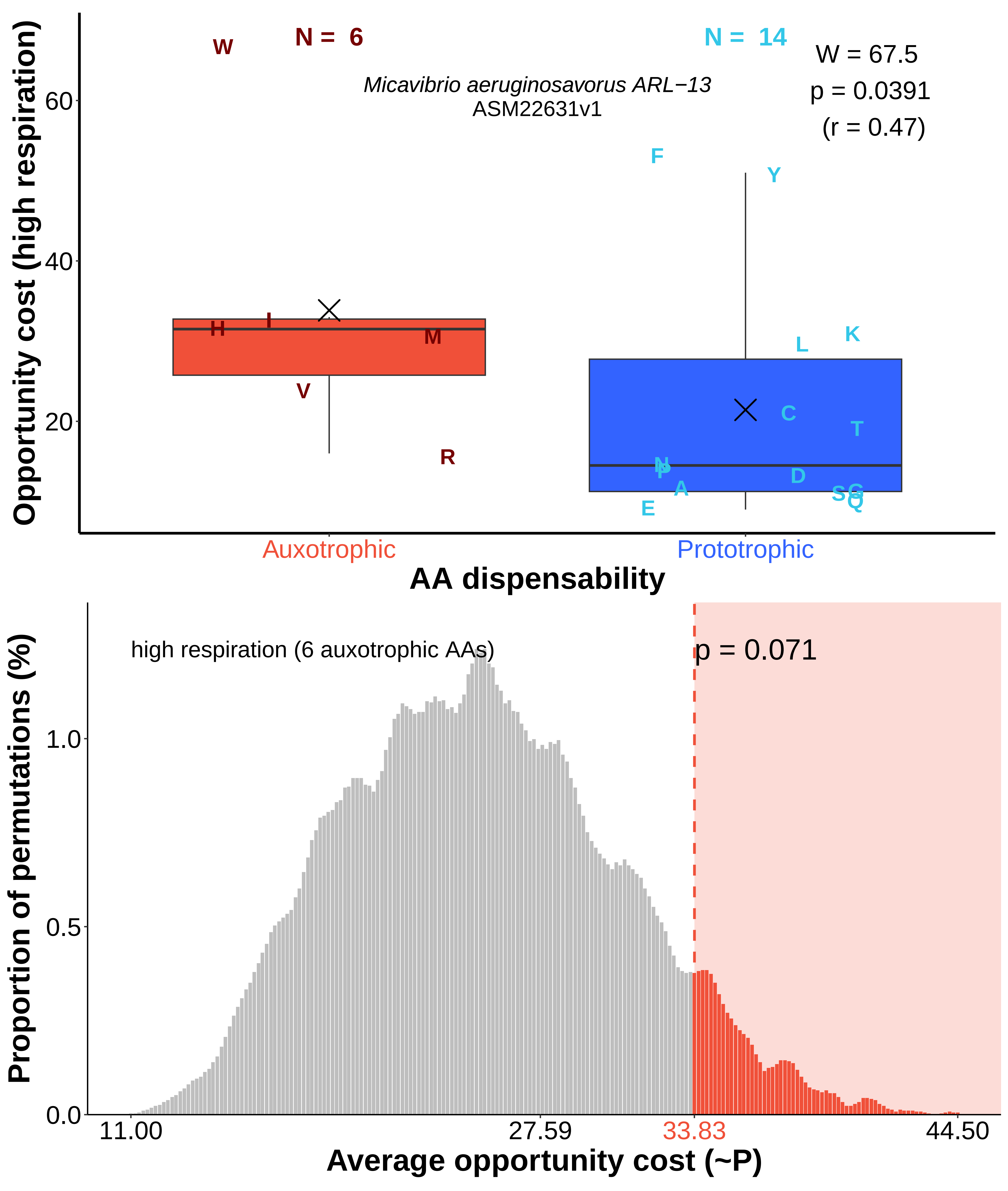

N = 6
N = 14
W = 67.5
p = 0.0391
(r = 0.47)
Micavibrio aeruginosavorus ARL−13
ASM22631v1
W
F
Y
I
H
M
L
K
V
C
T
R
N
P
D
A
S
G
Q
E
Opportunity cost (high respiration)
60
40
20
Auxotrophic
Prototrophic
AA dispensability
high respiration (6 auxotrophic AAs)
p = 0.071
Proportion of permutations (%)
1.0
0.5
0.0
11.00
27.59
33.83
44.50
Average opportunity cost (~P)

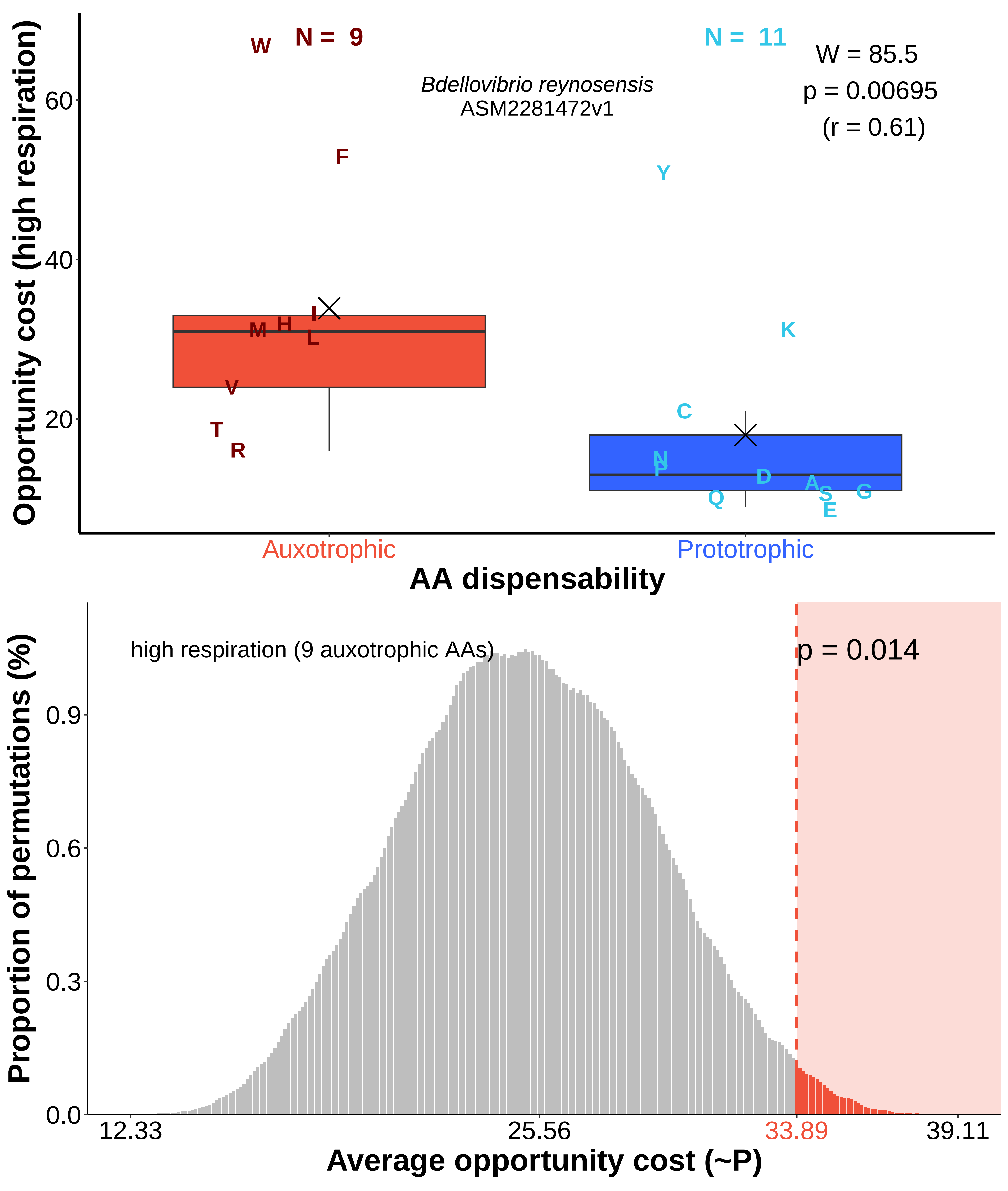
N = 9
N = 11
W = 85.5
p = 0.00695
(r = 0.61)
Bdellovibrio reynosensis
ASM2281472v1
W
F
I
H
M
L
V
T
R
Y
K
C
N
P
D
A
S
G
Q
E
60
40
20
Opportunity cost (high respiration)
Auxotrophic
Prototrophic
AA dispensability
high respiration (9 auxotrophic AAs)
p = 0.014
0.9
0.6
0.3
0.0
Proportion of permutations (%)
12.33
25.56
33.89
39.11
Average opportunity cost (~P)

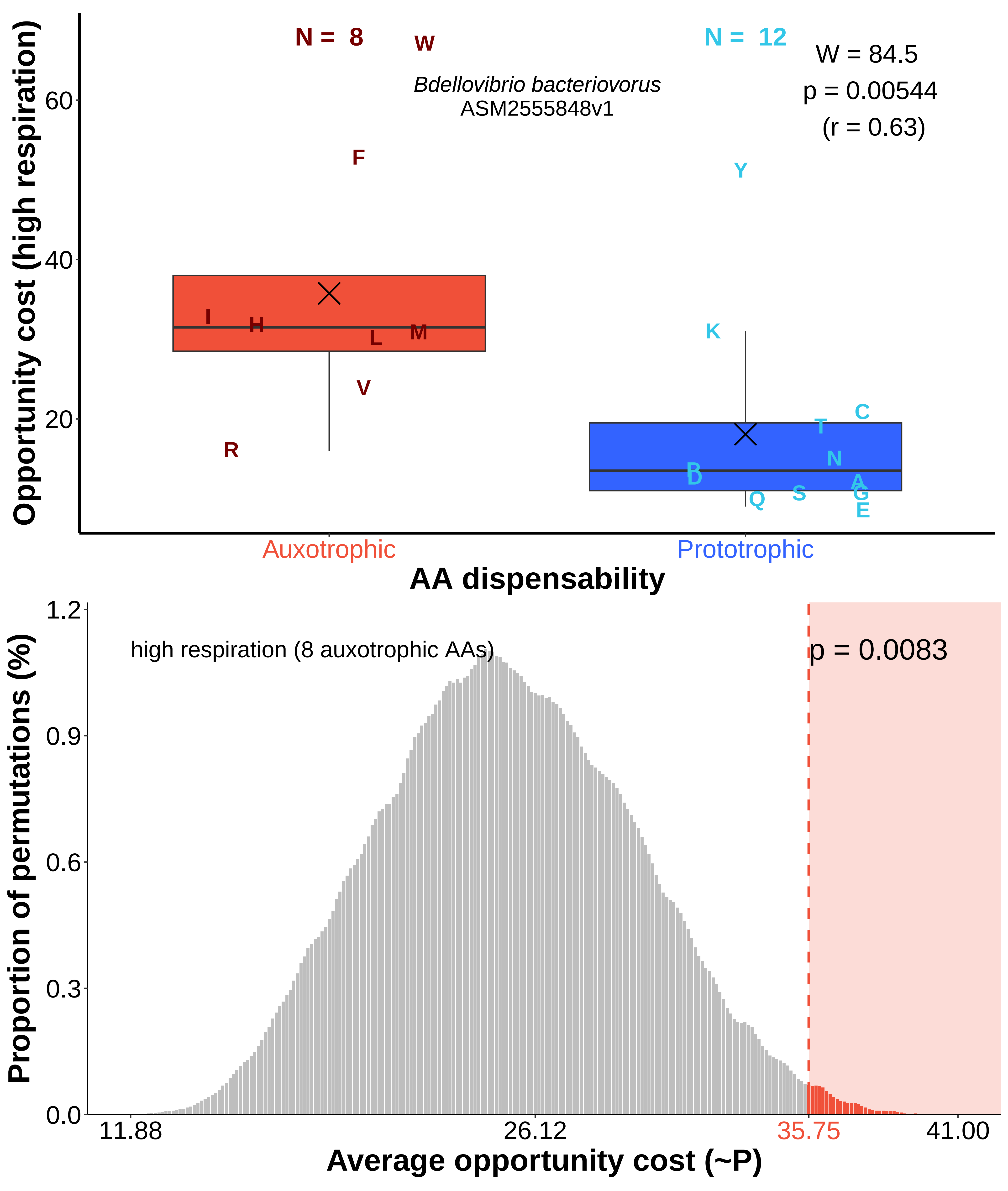
N = 8
W
N = 12
W = 84.5
p = 0.00544
(r = 0.63)
Bdellovibrio bacteriovorus
ASM2555848v1
F
Y
K
I
H
L
M
V
R
C
T
N
B
D
A
G
Q
S
E
60
40
20
Opportunity cost (high respiration)
Auxotrophic
Prototrophic
AA dispensability
high respiration (8 auxotrophic AAs)
p = 0.0083
1.2
0.9
0.6
0.3
0.0
Proportion of permutations (%)
11.88
26.12
35.75
41.00
Average opportunity cost (~P)

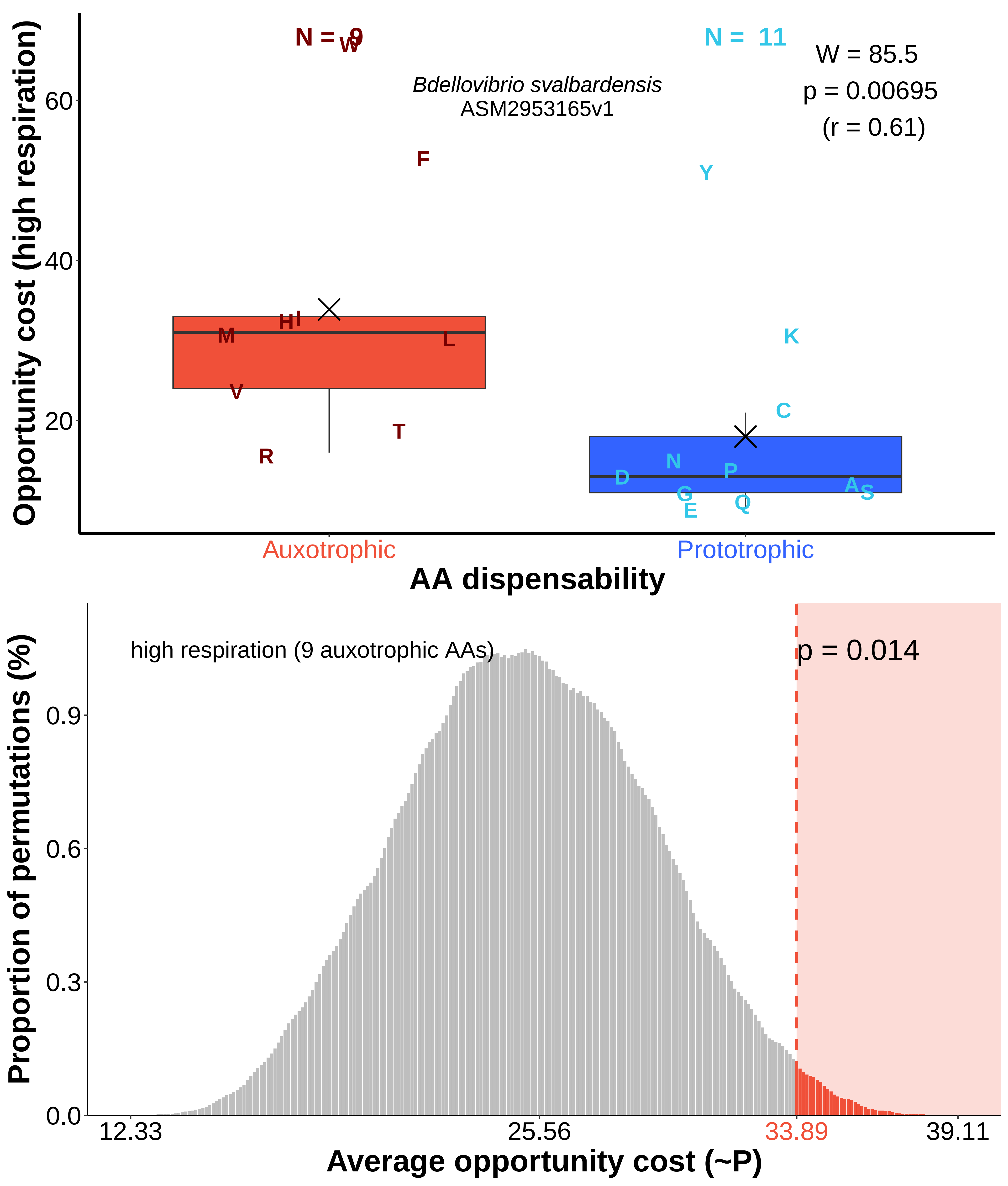

N = 9
N = 11
W = 85.5
p = 0.00695
(r = 0.61)
Bdellovibrio svalbardensis
ASM2953165v1
W
F
Y
H
I
M
L
K
V
C
T
R
N
D
P
G
A
S
Q
E
60
40
20
Opportunity cost (high respiration)
Auxotrophic
Prototrophic
AA dispensability
high respiration (9 auxotrophic AAs)
p = 0.014
0.9
0.6
0.3
0.0
Proportion of permutations (%)
12.33
25.56
33.89
39.11
Average opportunity cost (~P)

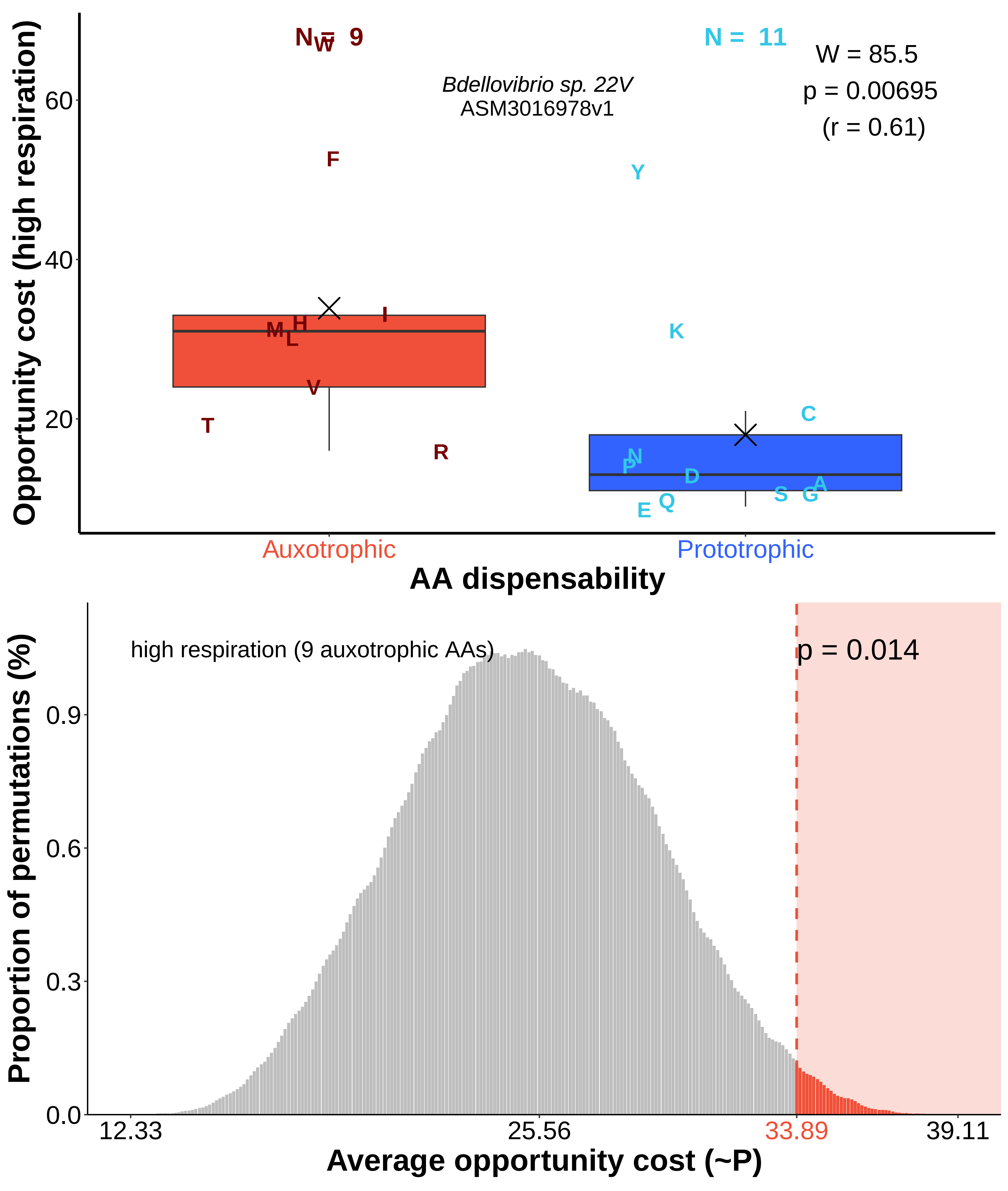

N = 9
N = 11
W = 85.5
p = 0.00695
(r = 0.61)
Bdellovibrio sp. 22V
ASM3016978v1
Opportunity cost (high respiration)
60
40
20
F
Y
I
M
H
L
K
V
T
C
R
N
P
D
A
S
G
E
Q
W
Auxotrophic
Prototrophic
AA dispensability
high respiration (9 auxotrophic AAs)
p = 0.014
Proportion of permutations (%)
0.9
0.6
0.3
0.0
12.33
25.56
33.89
39.11
Average opportunity cost (~P)

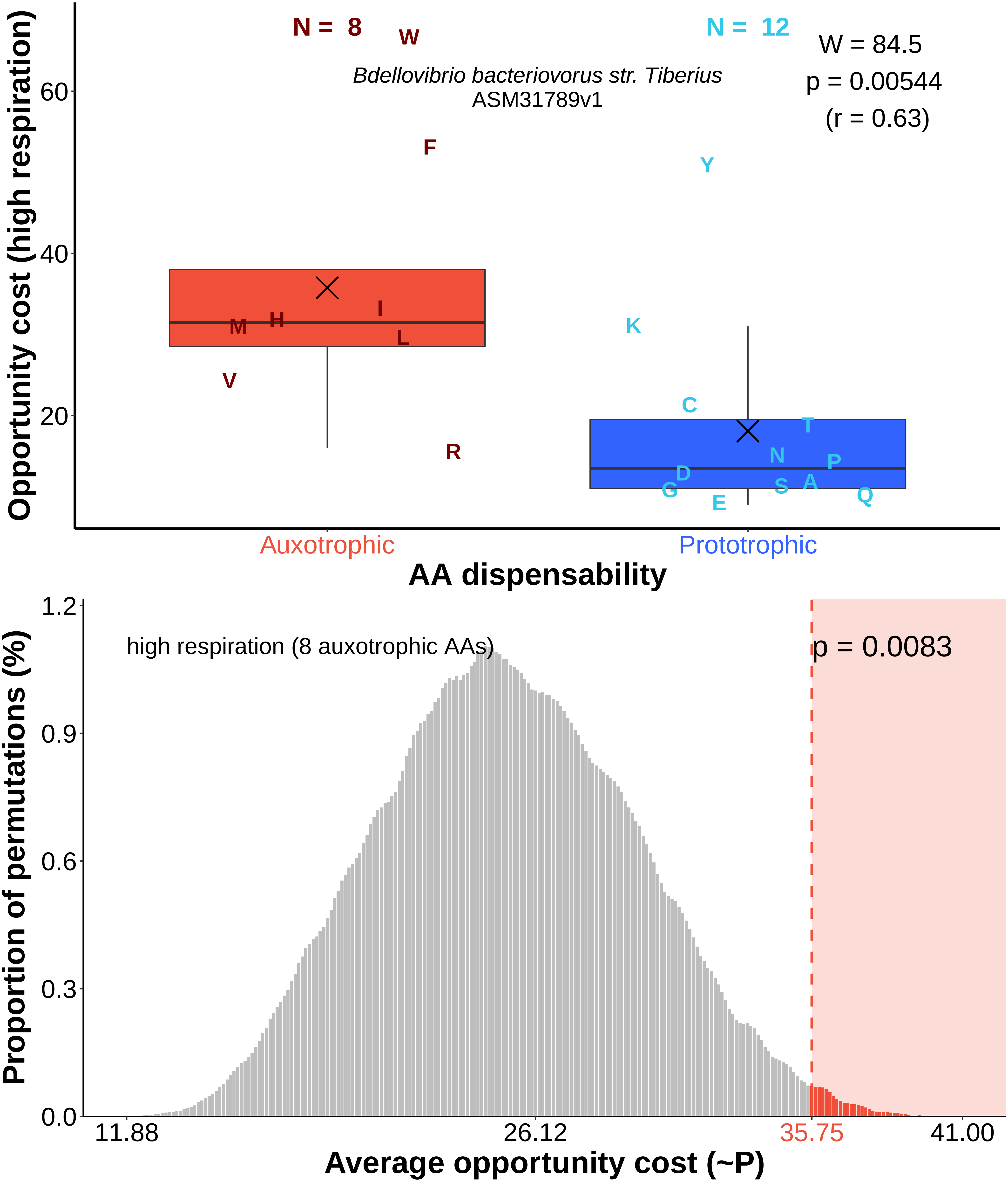

N = 8
N = 12
W = 84.5
p = 0.00544
(r = 0.63)
Bdellovibrio bacteriovorus str. Tiberius
ASM31789v1
Opportunity cost (high respiration)
Auxotrophic
Prototrophic
AA dispensability
W F I H M L V R
Y K C T N P D S A G E Q
60
40
20
high respiration (8 auxotrophic AAs)
p = 0.0083
Proportion of permutations (%)
1.2
0.9
0.6
0.3
0.0
11.88
26.12
35.75
41.00
Average opportunity cost (~P)

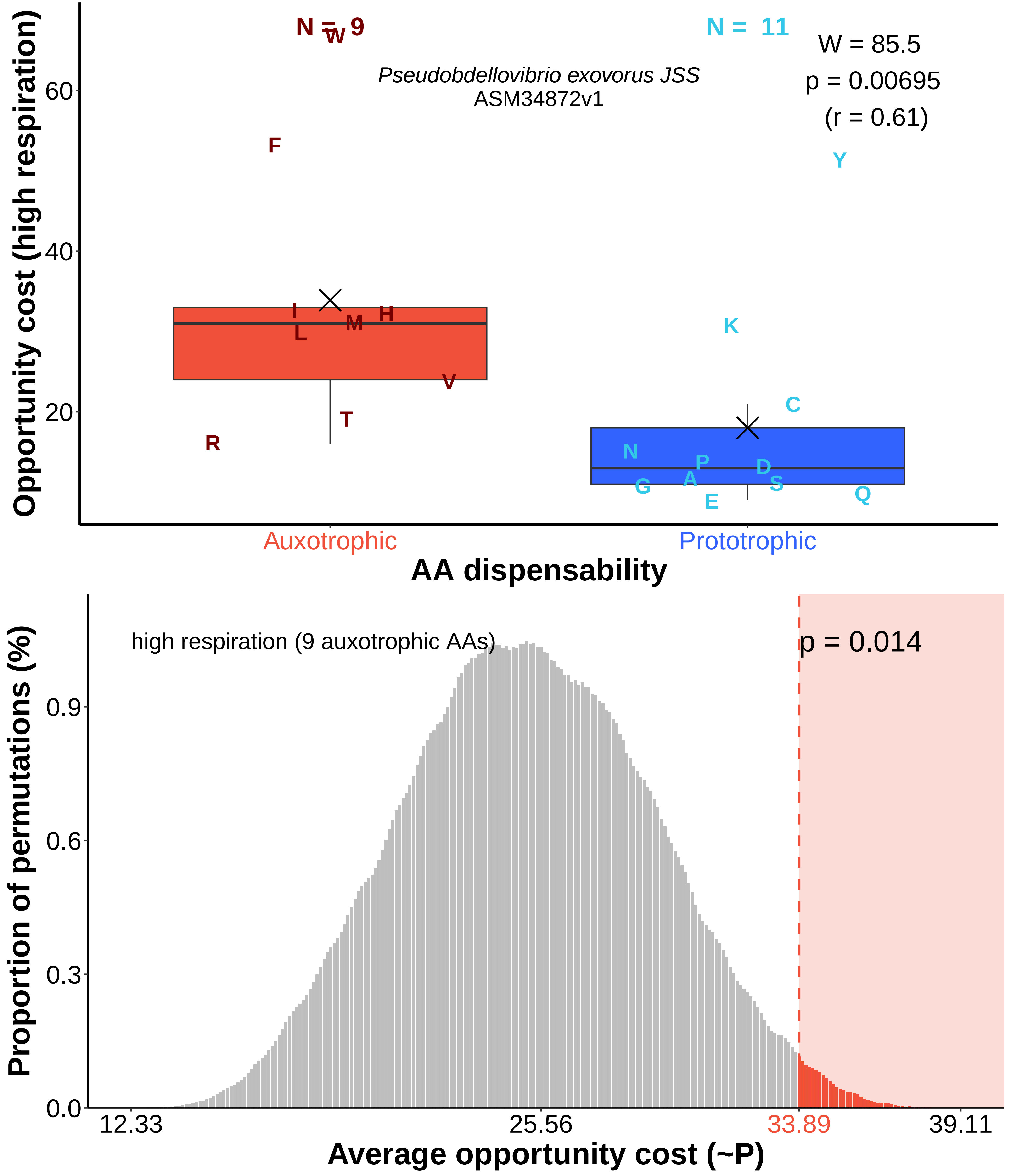

N = 9
N = 11
W = 85.5
p = 0.00695
(r = 0.61)
Pseudobdellovibrio exovorus JSS
ASM34872v1
Opportunity cost (high respiration)
60
40
20
W
F
I
H
M
L
V
T
R
Y
K
C
N
P
D
A
S
G
Q
E
Auxotrophic
Prototrophic
AA dispensability
high respiration (9 auxotrophic AAs)
p = 0.014
Proportion of permutations (%)
0.9
0.6
0.3
0.0
12.33
25.56
33.89
39.11
Average opportunity cost (~P)

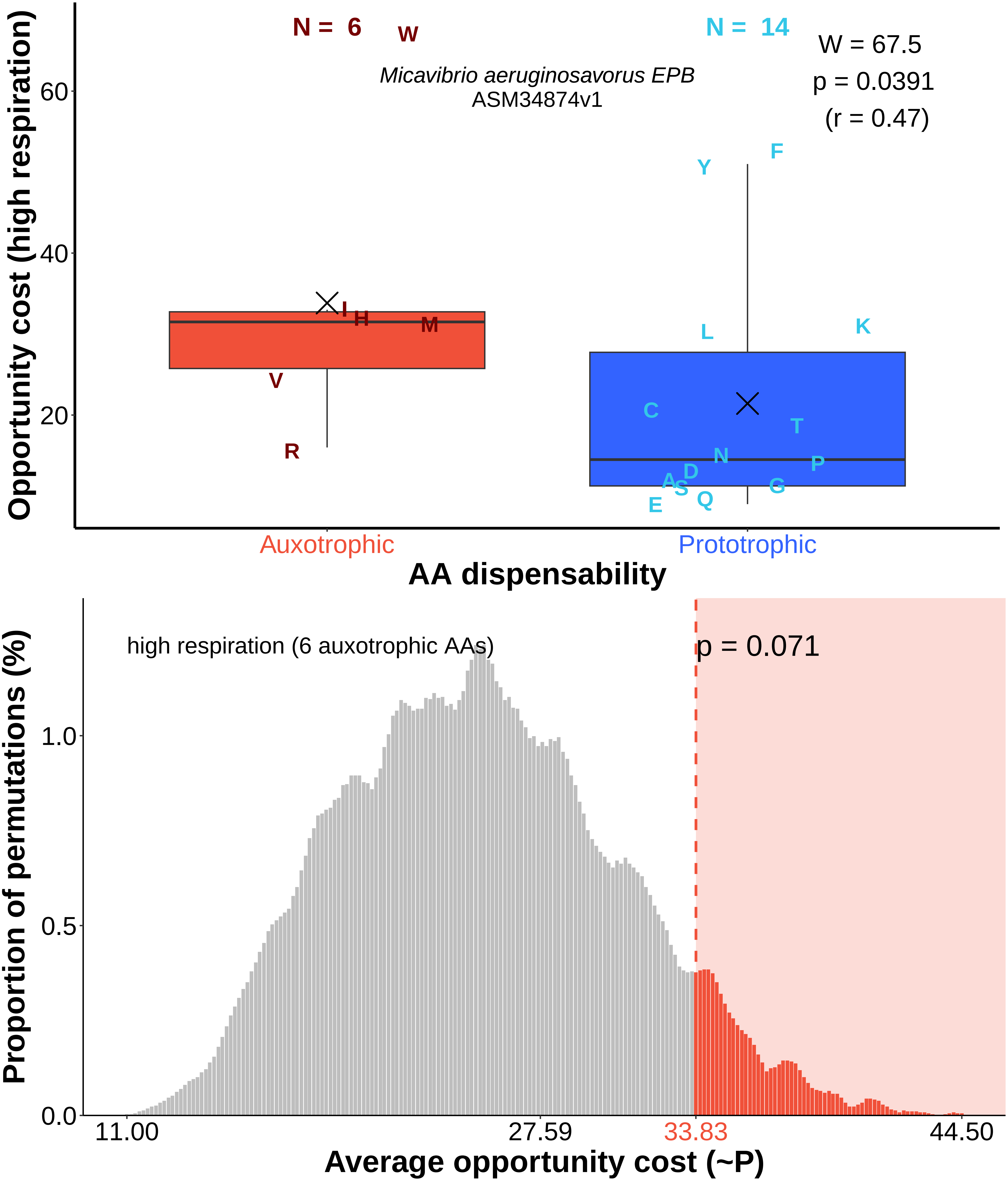

N = 6
W
N = 14
W = 67.5
p = 0.0391
(r = 0.47)
Micavibrio aeruginosavorus EPB
ASM34874v1
Opportunity cost (high respiration)
60
40
20
I
H
M
V
R
Y
F
L
K
C
T
N
P
D
A
S
G
Q
E
Auxotrophic
Prototrophic
AA dispensability
high respiration (6 auxotrophic AAs)
p = 0.071
Proportion of permutations (%)
1.0
0.5
0.0
11.00
27.59
33.83
44.50
Average opportunity cost (~P)

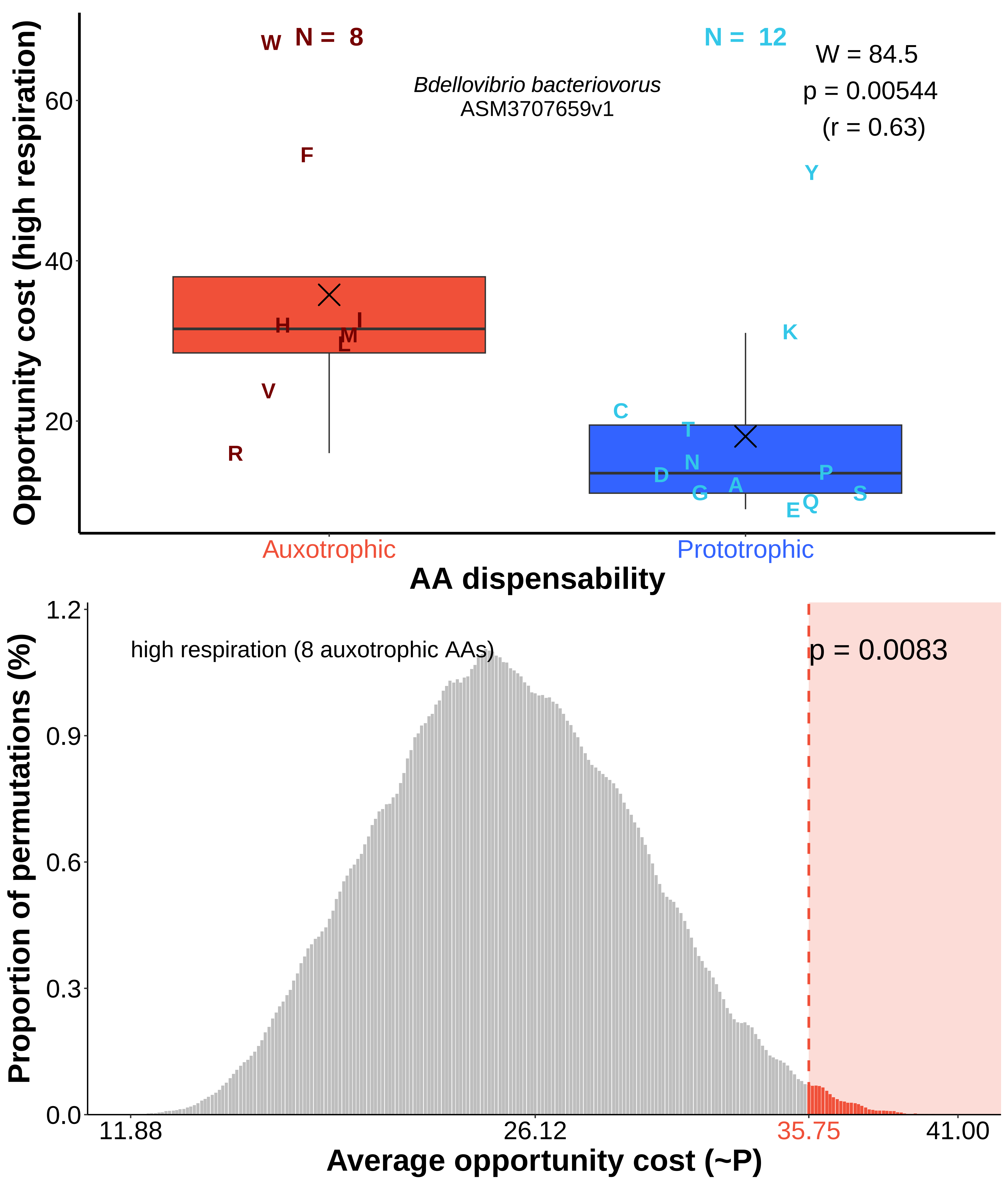

W N = 8
N = 12
W = 84.5
p = 0.00544
(r = 0.63)
Bdellovibrio bacteriovorus
ASM3707659v1
F
Y
H
I
M
L
K
V
C
T
R
N
D
P
A
G
S
E
Q
Opportunity cost (high respiration)
60
40
20
Auxotrophic
Prototrophic
AA dispensability
high respiration (8 auxotrophic AAs)
p = 0.0083
Proportion of permutations (%)
1.2
0.9
0.6
0.3
0.0
11.88
26.12
35.75
41.00
Average opportunity cost (~P)

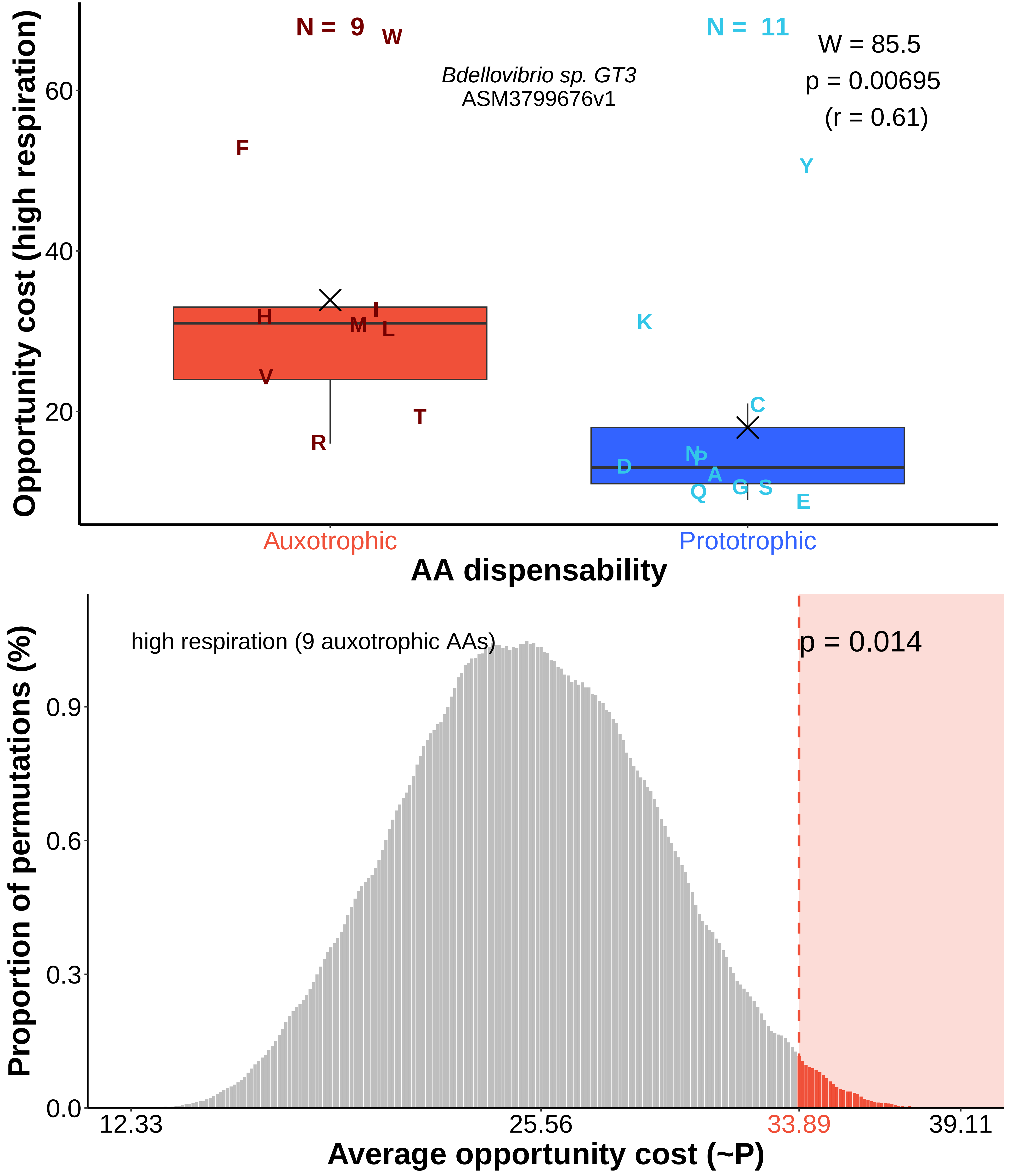

N = 9
N = 11
W = 85.5
p = 0.00695
(r = 0.61)
Bdellovibrio sp. GT3
ASM3799676v1
Opportunity cost (high respiration)
60
40
20
F
W
H
I
M
L
V
T
R
Y
K
C
N
P
D
A
Q
G
S
E
Auxotrophic
Prototrophic
AA dispensability
high respiration (9 auxotrophic AAs)
p = 0.014
Proportion of permutations (%)
0.9
0.6
0.3
0.0
12.33
25.56
33.89
39.11
Average opportunity cost (~P)

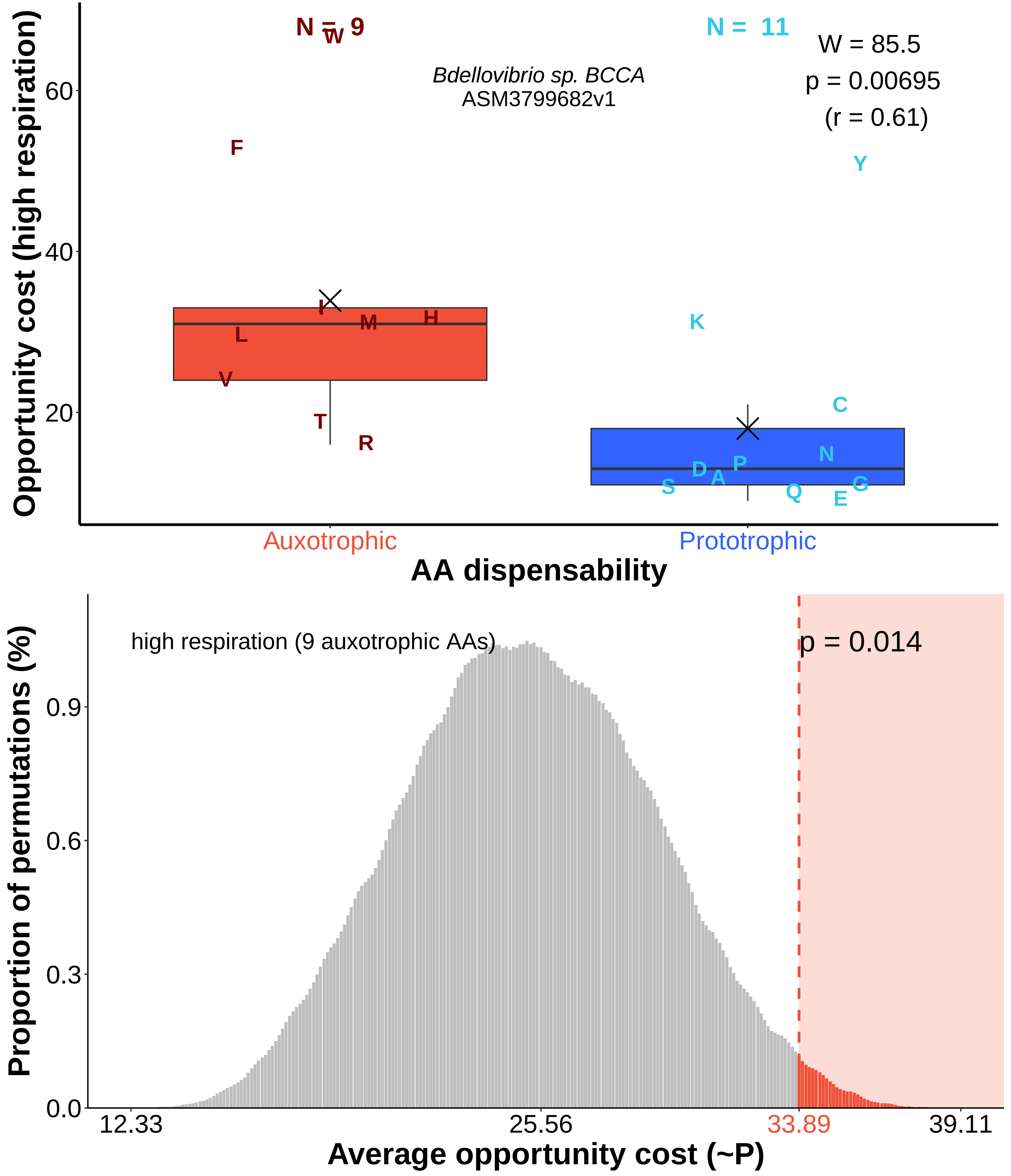
N = 9
N = 11
W = 85.5
p = 0.00695
(r = 0.61)
Bdellovibrio sp. BCCA
ASM3799682v1
Opportunity cost (high respiration)
60
40
20
F
W
I
M
H
L
V
T
R
K
Y
C
N
D
P
A
S
Q
G
E
Auxotrophic
Prototrophic
AA dispensability
high respiration (9 auxotrophic AAs)
p = 0.014
Proportion of permutations (%)
0.9
0.6
0.3
0.0
12.33
25.56
33.89
39.11
Average opportunity cost (~P)

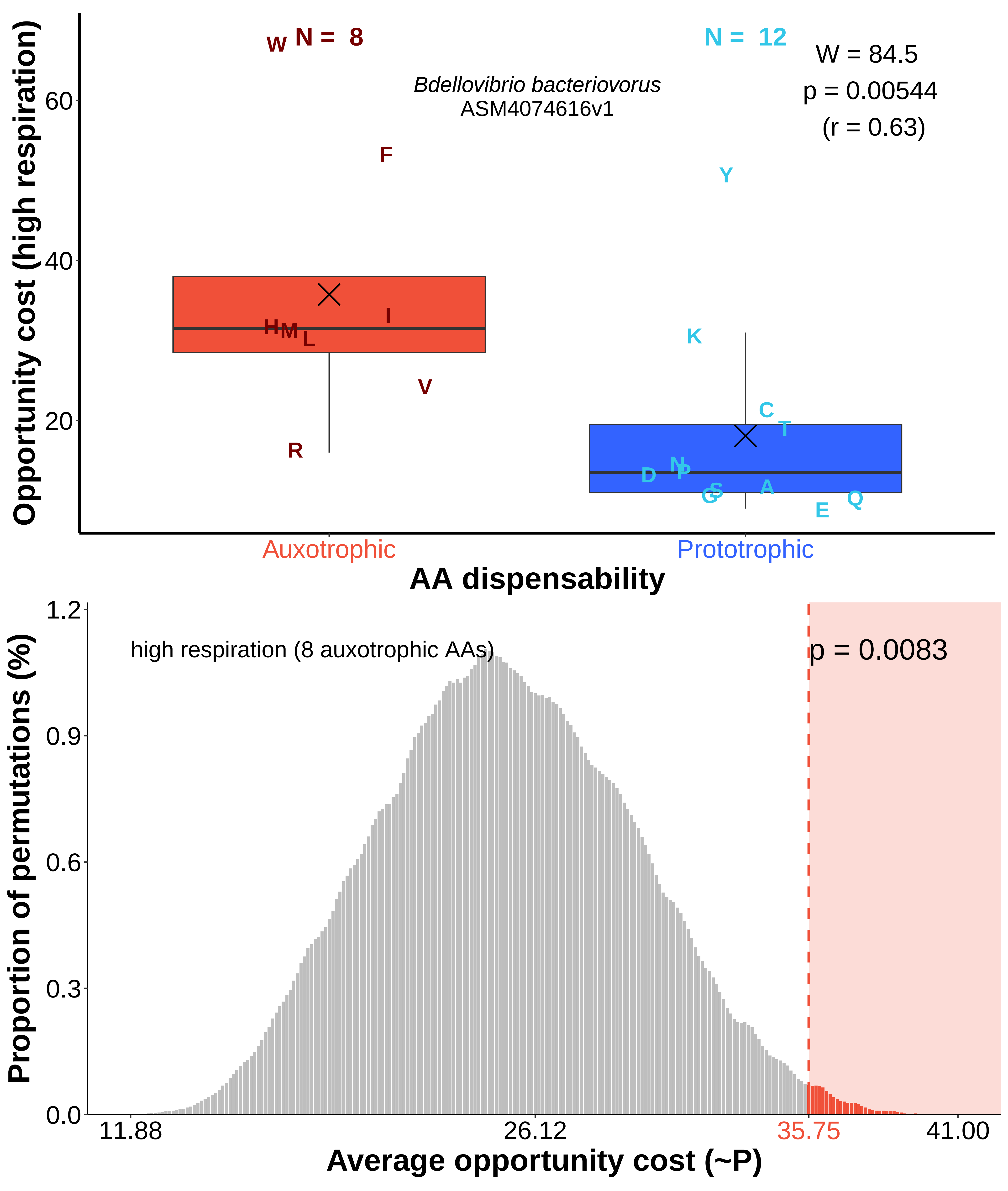

W N = 8
N = 12
W = 84.5
p = 0.00544
(r = 0.63)
Bdellovibrio bacteriovorus
ASM4074616v1
F
Y
I
H M L
K
V
C T
R
N P D
G S A
E Q
Opportunity cost (high respiration)
60
40
20
Auxotrophic
Prototrophic
AA dispensability
high respiration (8 auxotrophic AAs)
p = 0.0083
Proportion of permutations (%)
1.2
0.9
0.6
0.3
0.0
11.88
26.12
35.75
41.00
Average opportunity cost (~P)

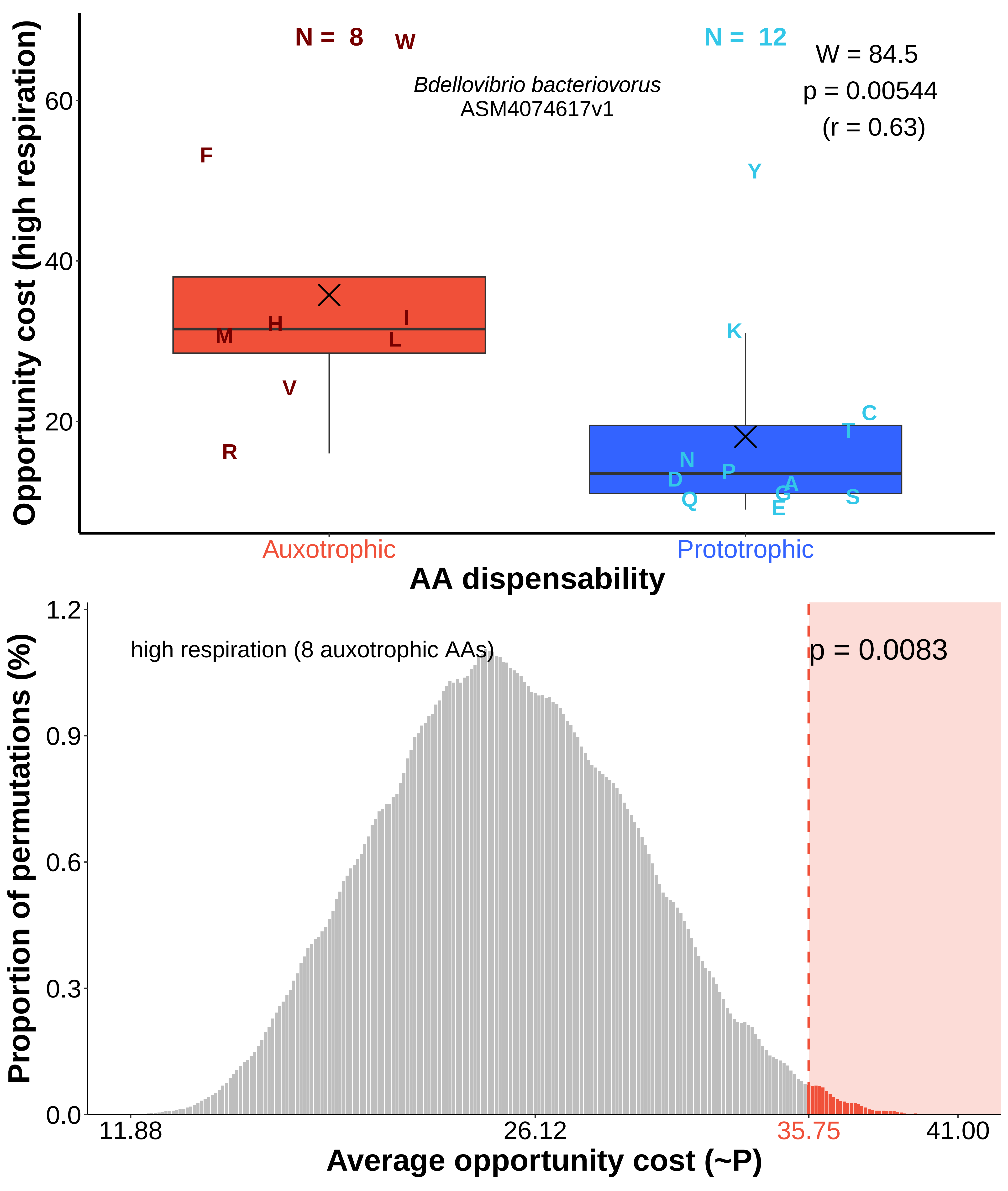
N = 8
N = 12
W = 84.5
p = 0.00544
(r = 0.63)
Bdellovibrio bacteriovorus
ASM4074617v1
Opportunity cost (high respiration)
60
40
20
F
W
H
M
I
L
V
R
Y
K
C
T
N
D
P
A
G
Q
E
S
Auxotrophic
Prototrophic
AA dispensability
high respiration (8 auxotrophic AAs)
p = 0.0083
Proportion of permutations (%)
1.2
0.9
0.6
0.3
0.0
11.88
26.12
35.75
41.00
Average opportunity cost (~P)

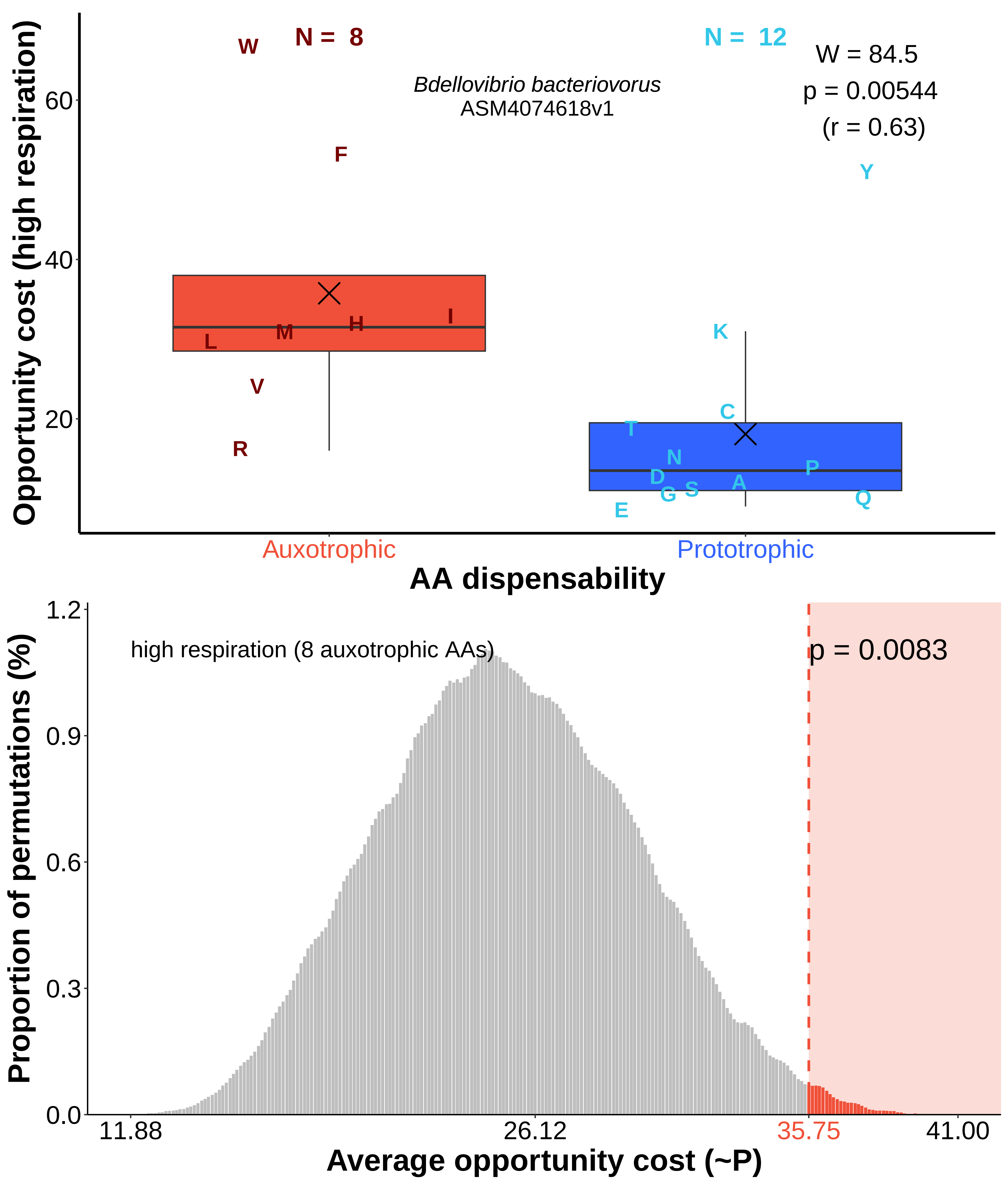

N = 8
N = 12
W = 84.5
p = 0.00544
(r = 0.63)
Bdellovibrio bacteriovorus
ASM4074618v1
Opportunity cost (high respiration)
W
F
L
M
H
I
V
R
K
C
T
N
D
G
S
A
P
Q
E
Y
60
40
20
Auxotrophic
Prototrophic
AA dispensability
high respiration (8 auxotrophic AAs)
p = 0.0083
Proportion of permutations (%)
1.2
0.9
0.6
0.3
0.0
11.88
26.12
35.75
41.00
Average opportunity cost (~P)

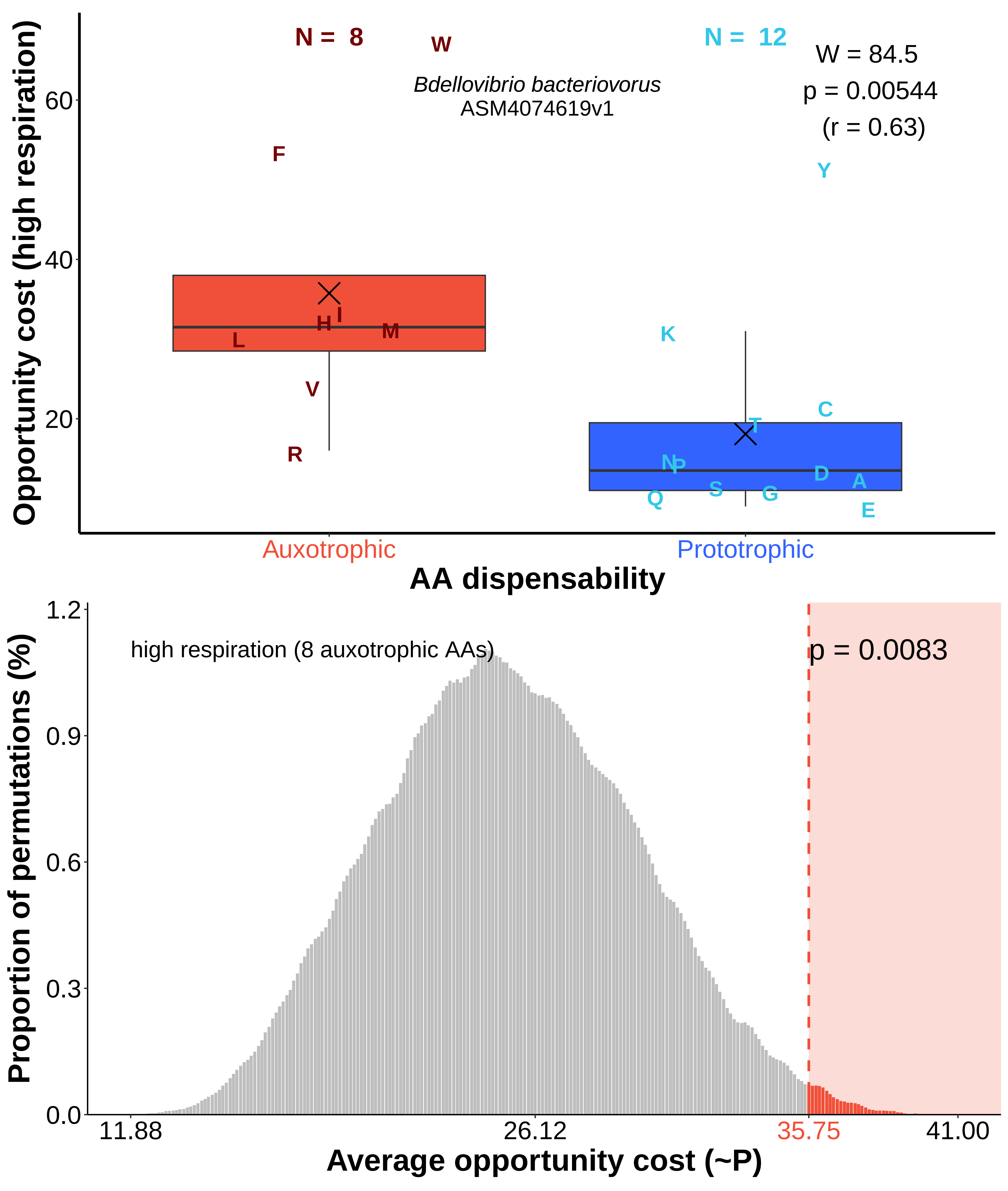

N = 8
N = 12
W = 84.5
p = 0.00544
(r = 0.63)
Bdellovibrio bacteriovorus
ASM4074619v1
Opportunity cost (high respiration)
Auxotrophic
Prototrophic
AA dispensability
high respiration (8 auxotrophic AAs)
p = 0.0083
Proportion of permutations (%)
11.88
26.12
35.75
41.00
Average opportunity cost (~P)

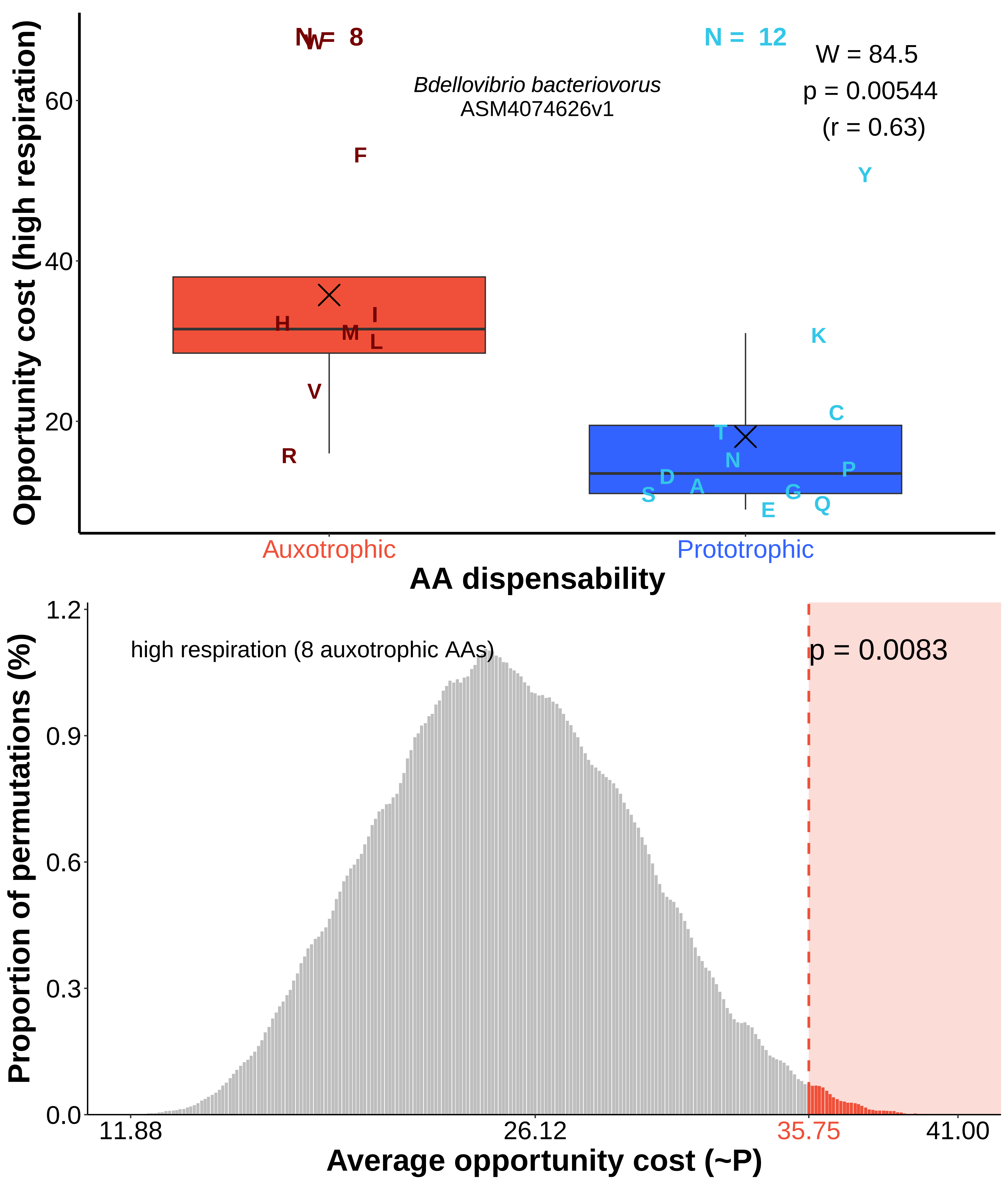

N = 8
N = 12
W = 84.5
p = 0.00544
(r = 0.63)
Bdellovibrio bacteriovorus
ASM4074626v1
Opportunity cost (high respiration)
60
40
20
W
F
Y
H
M
I
L
K
V
C
R
T
N
D
P
A
G
S
E
Q
Auxotrophic
Prototrophic
AA dispensability
Proportion of permutations (%)
1.2
0.9
0.6
0.3
0.0
high respiration (8 auxotrophic AAs)
p = 0.0083
11.88
26.12
35.75
41.00
Average opportunity cost (~P)

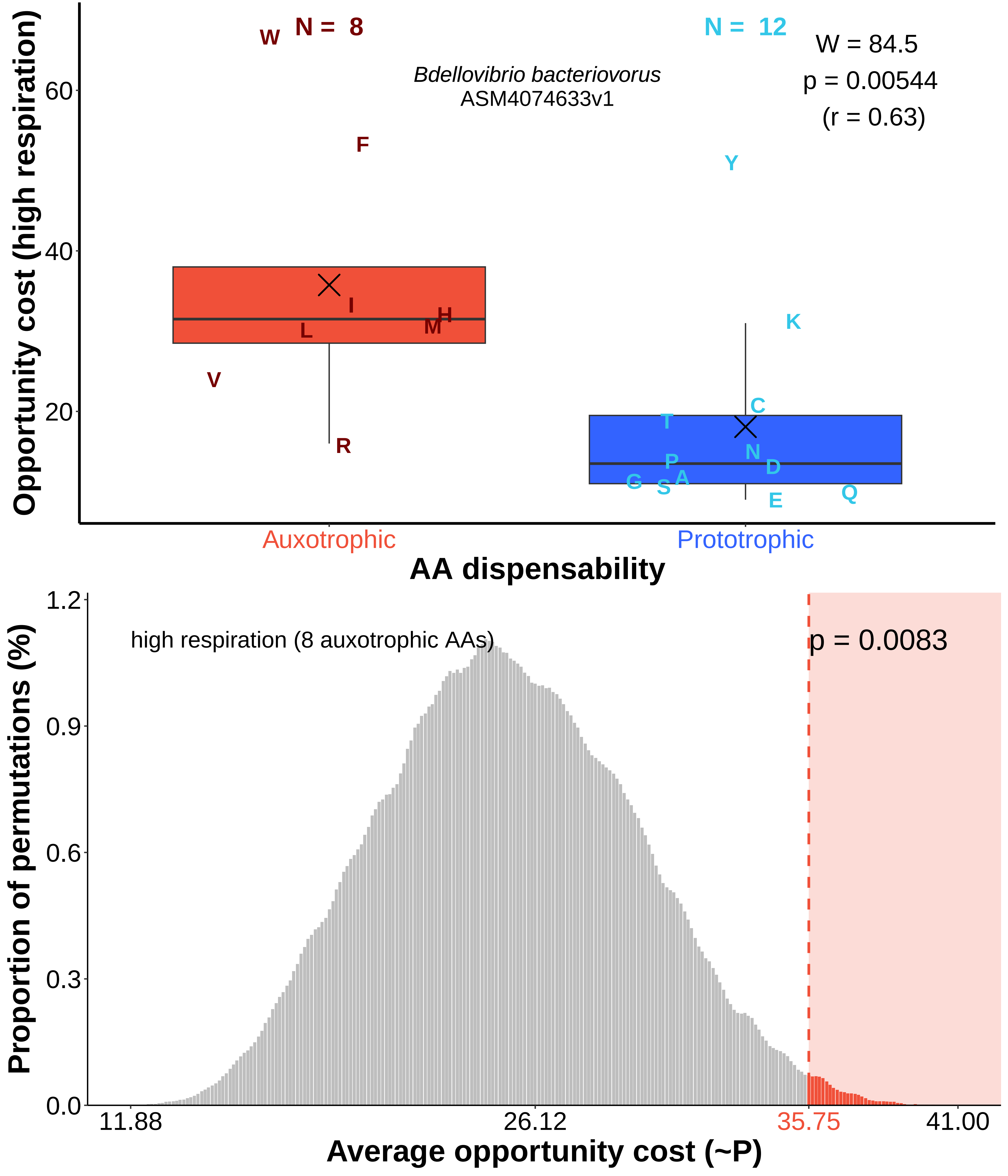

N = 8
N = 12
W = 84.5
p = 0.00544
(r = 0.63)
Bdellovibrio bacteriovorus
ASM4074633v1
Opportunity cost (high respiration)
Auxotrophic
Prototrophic
AA dispensability
W
F
I
H
M
L
V
R
Y
K
C
T
N
P
D
A
G
S
E
Q
high respiration (8 auxotrophic AAs)
p = 0.0083
Proportion of permutations (%)
11.88
26.12
35.75
41.00
Average opportunity cost (~P)

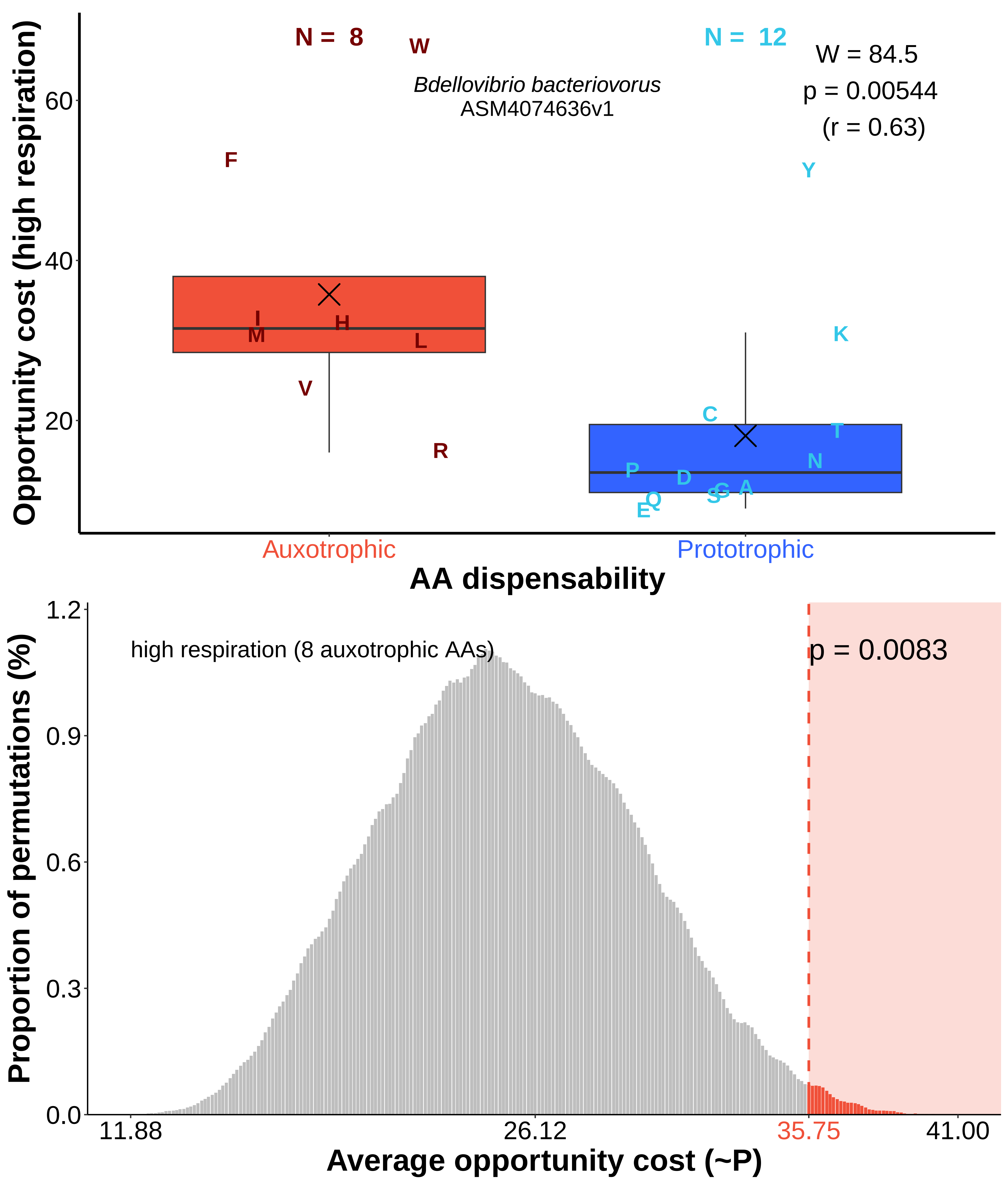

N = 8
N = 12
W = 84.5
p = 0.00544
(r = 0.63)
Bdellovibrio bacteriovorus
ASM4074636v1
Opportunity cost (high respiration)
60
40
20
W
F
I
H
M
L
V
R
Y
K
C
T
N
P
D
Q
S
G
A
E
Auxotrophic
Prototrophic
AA dispensability
high respiration (8 auxotrophic AAs)
p = 0.0083
Proportion of permutations (%)
1.2
0.9
0.6
0.3
0.0
11.88
26.12
35.75
41.00
Average opportunity cost (~P)

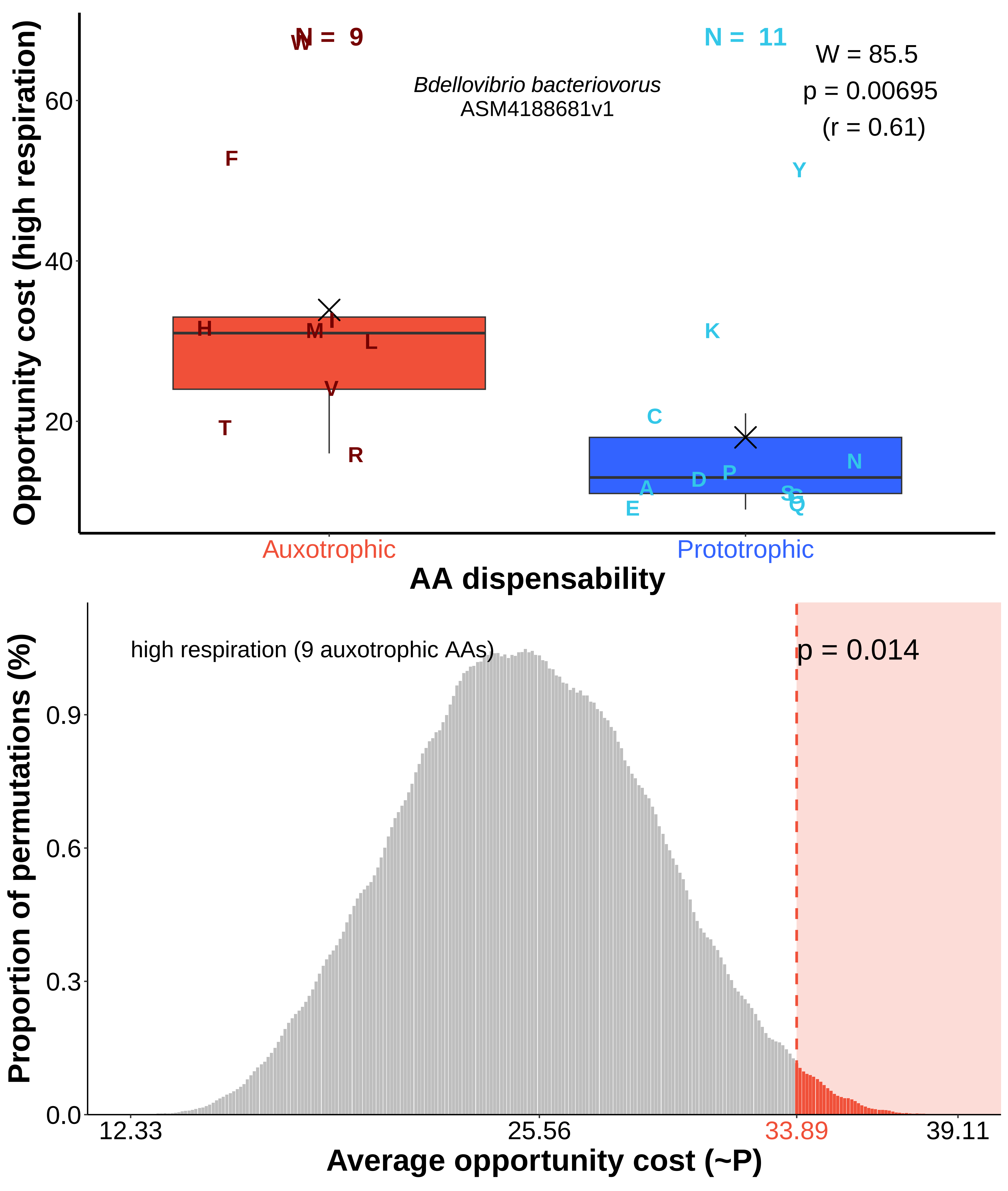
N = 9
N = 11
W = 85.5
p = 0.00695
(r = 0.61)
Bdellovibrio bacteriovorus
ASM4188681v1
Opportunity cost (high respiration)
Auxotrophic
Prototrophic
AA dispensability
high respiration (9 auxotrophic AAs)
p = 0.014
Proportion of permutations (%)
12.33
25.56
33.89
39.11
Average opportunity cost (~P)

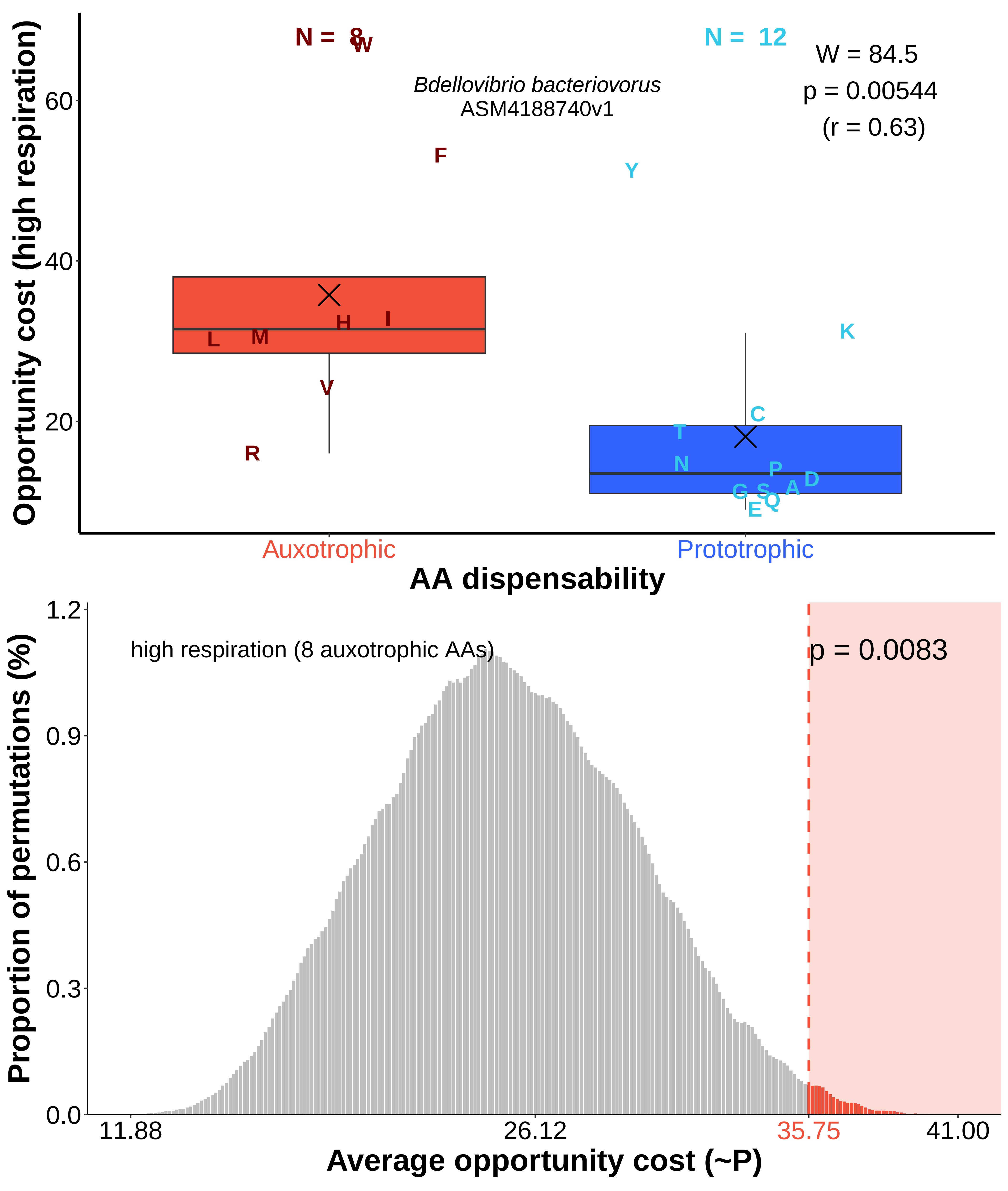
N = 8
N = 12
W = 84.5
p = 0.00544
(r = 0.63)
Bdellovibrio bacteriovorus
ASM4188740v1
Opportunity cost (high respiration)
Auxotrophic
Prototrophic
AA dispensability
high respiration (8 auxotrophic AAs)
p = 0.0083
Proportion of permutations (%)
11.88
26.12
35.75
41.00
Average opportunity cost (~P)

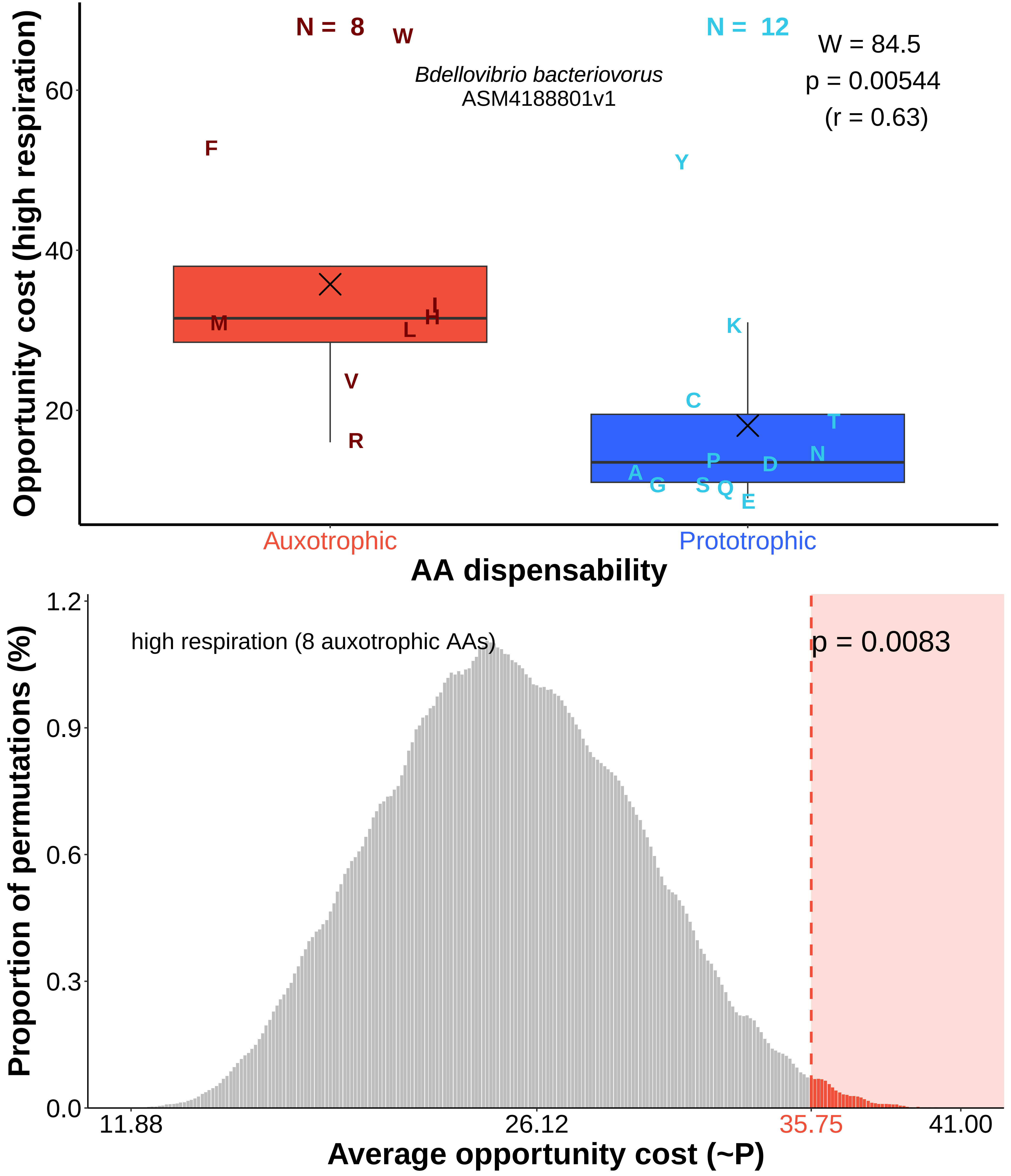
N = 8
N = 12
W = 84.5
p = 0.00544
(r = 0.63)
Bdellovibrio bacteriovorus
ASM4188801v1
Opportunity cost (high respiration)
60
40
20
F
W
M
I
H
L
V
R
Y
K
C
T
P
D
N
A
G
S
Q
E
Auxotrophic
Prototrophic
AA dispensability
high respiration (8 auxotrophic AAs)
p = 0.0083
Proportion of permutations (%)
1.2
0.9
0.6
0.3
0.0
11.88
26.12
35.75
41.00
Average opportunity cost (~P)

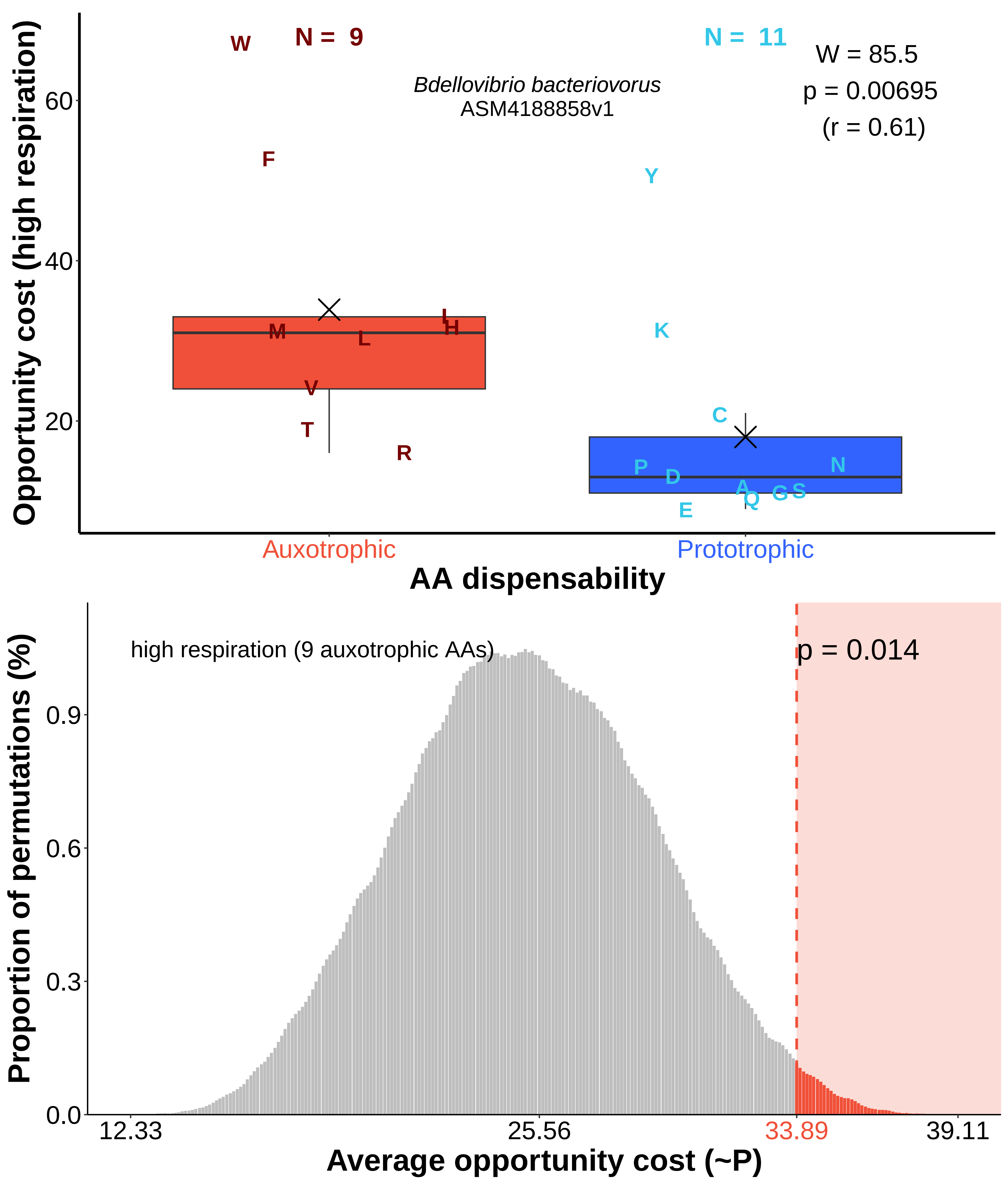

W
N = 9
N = 11
W = 85.5
p = 0.00695
(r = 0.61)
Bdellovibrio bacteriovorus
ASM4188858v1
F
Y
K
M
L
I
H
V
T
R
C
P
D
N
A
Q
G
S
E
60
40
20
Opportunity cost (high respiration)
Auxotrophic
Prototrophic
AA dispensability
high respiration (9 auxotrophic AAs)
p = 0.014
0.9
0.6
0.3
0.0
Proportion of permutations (%)
12.33
25.56
33.89
39.11
Average opportunity cost (~P)

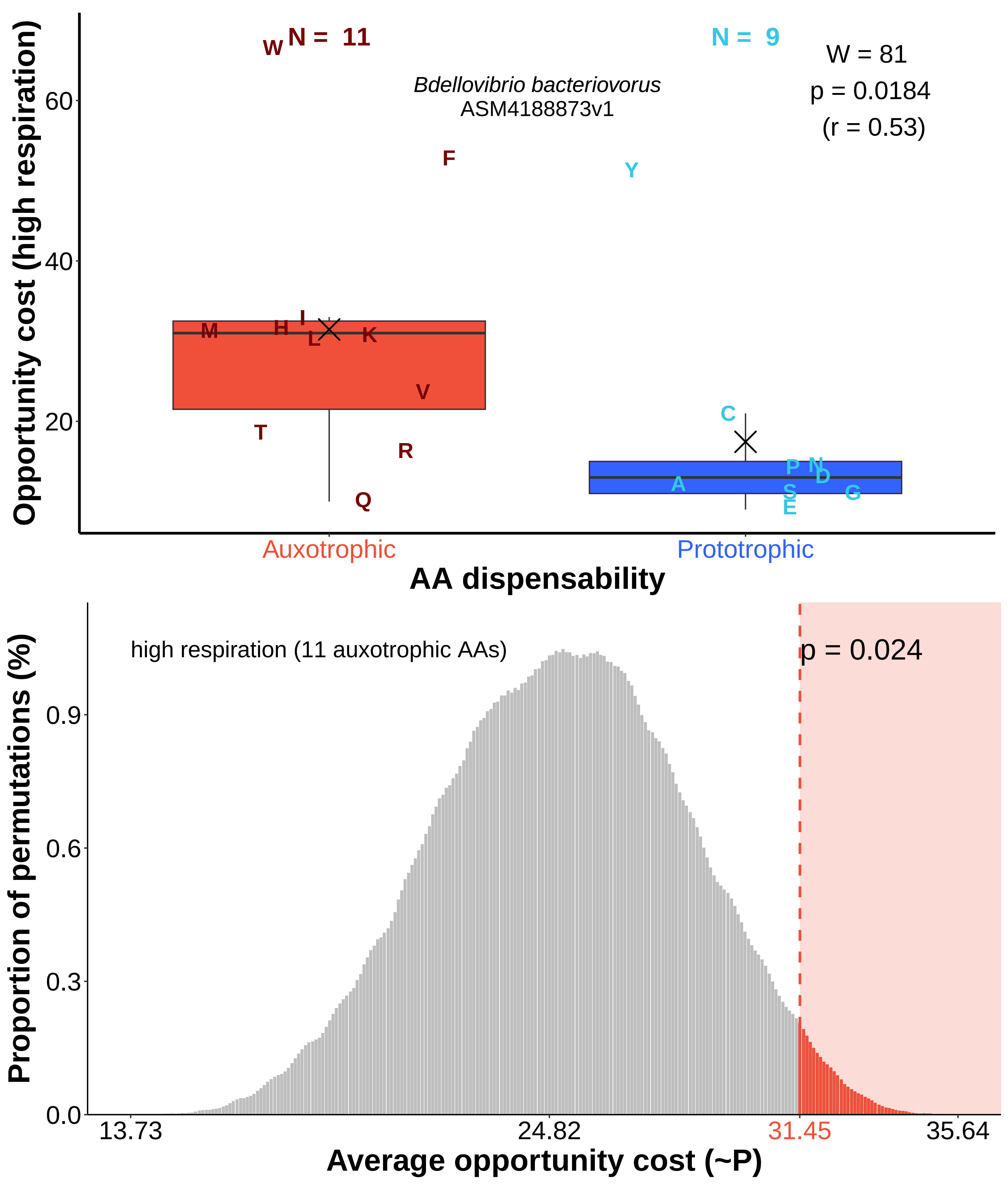

W N = 11
N = 9
W = 81
p = 0.0184
(r = 0.53)
Bdellovibrio bacteriovorus
ASM4188873v1
Opportunity cost (high respiration)
60
40
20
F
Y
I
M
H
L
K
V
T
R
Q
C
P
N
D
A
S
G
E
Auxotrophic
Prototrophic
AA dispensability
high respiration (11 auxotrophic AAs)
p = 0.024
Proportion of permutations (%)
0.9
0.6
0.3
0.0
13.73
24.82
31.45
35.64
Average opportunity cost (~P)

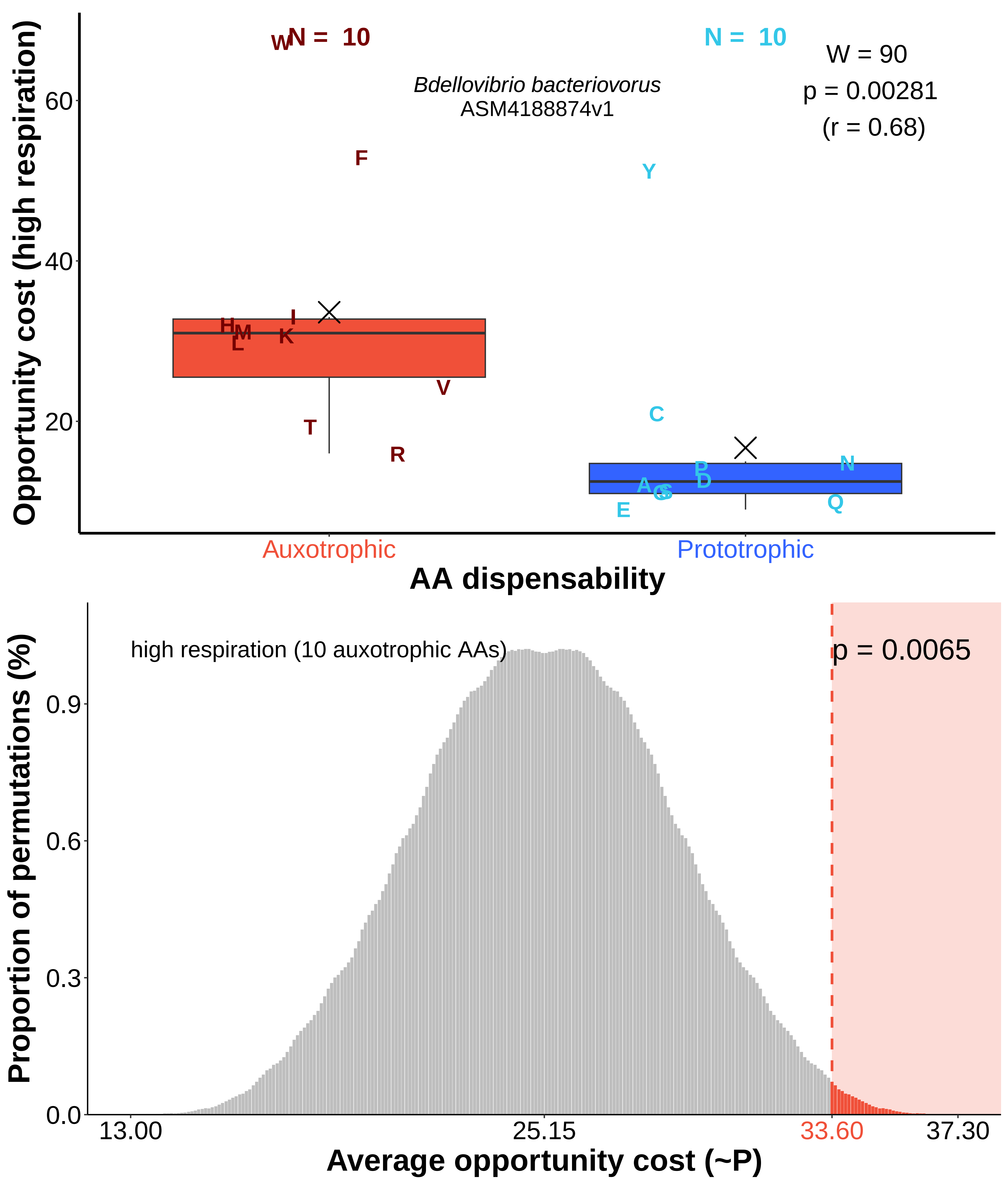
N = 10
N = 10
Bdellovibrio bacteriovorus
ASM4188874v1
W = 90
p = 0.00281
(r = 0.68)
Opportunity cost (high respiration)
Auxotrophic
Prototrophic
AA dispensability
high respiration (10 auxotrophic AAs)
p = 0.0065
Proportion of permutations (%)
13.00
25.15
33.60
37.30
Average opportunity cost (~P)

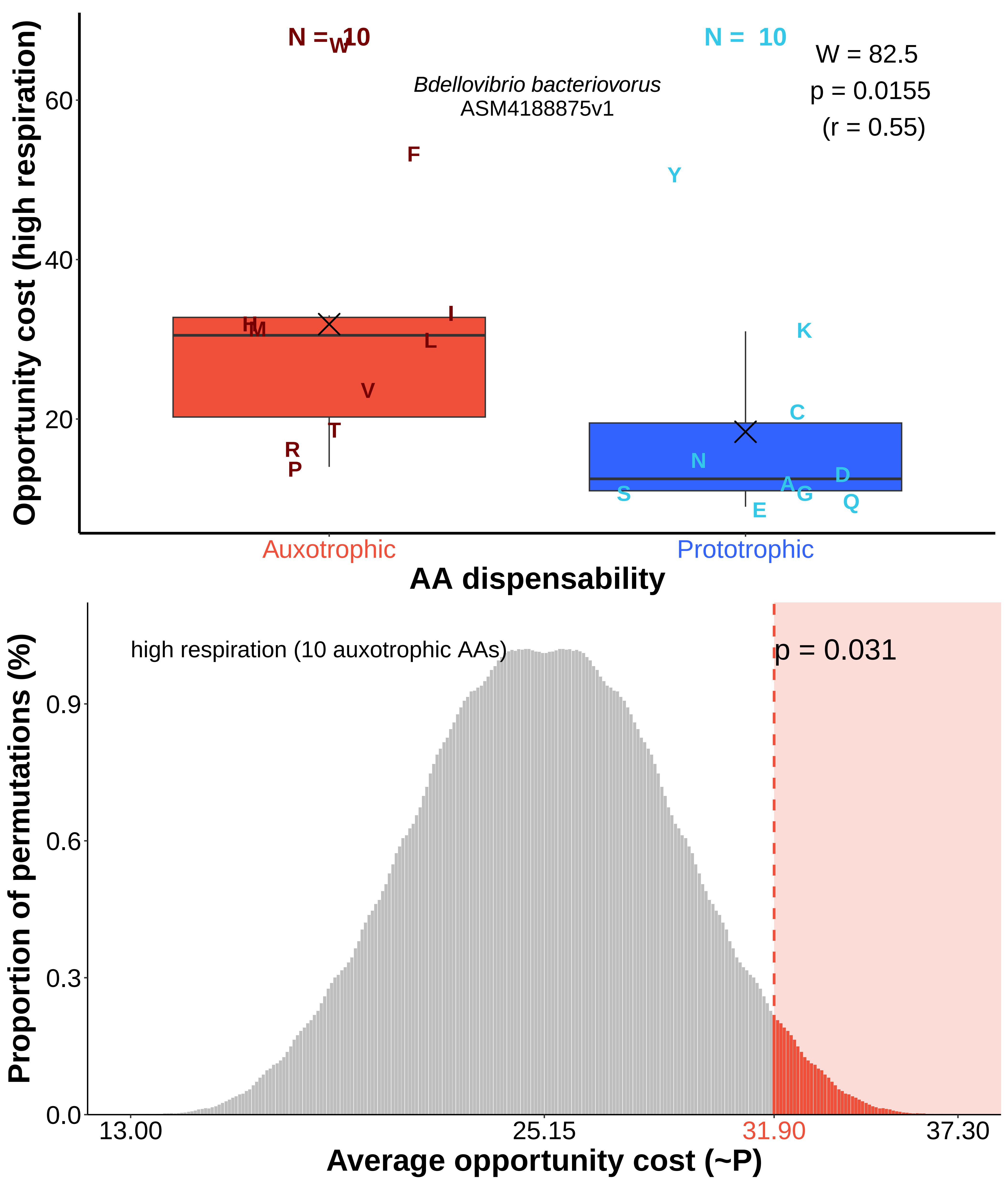

N = 10
N = 10
W = 82.5
p = 0.0155
(r = 0.55)
Bdellovibrio bacteriovorus
ASM4188875v1
Opportunity cost (high respiration)
Auxotrophic
Prototrophic
AA dispensability
high respiration (10 auxotrophic AAs)
p = 0.031
Proportion of permutations (%)
13.00
25.15
31.90
37.30
Average opportunity cost (~P)

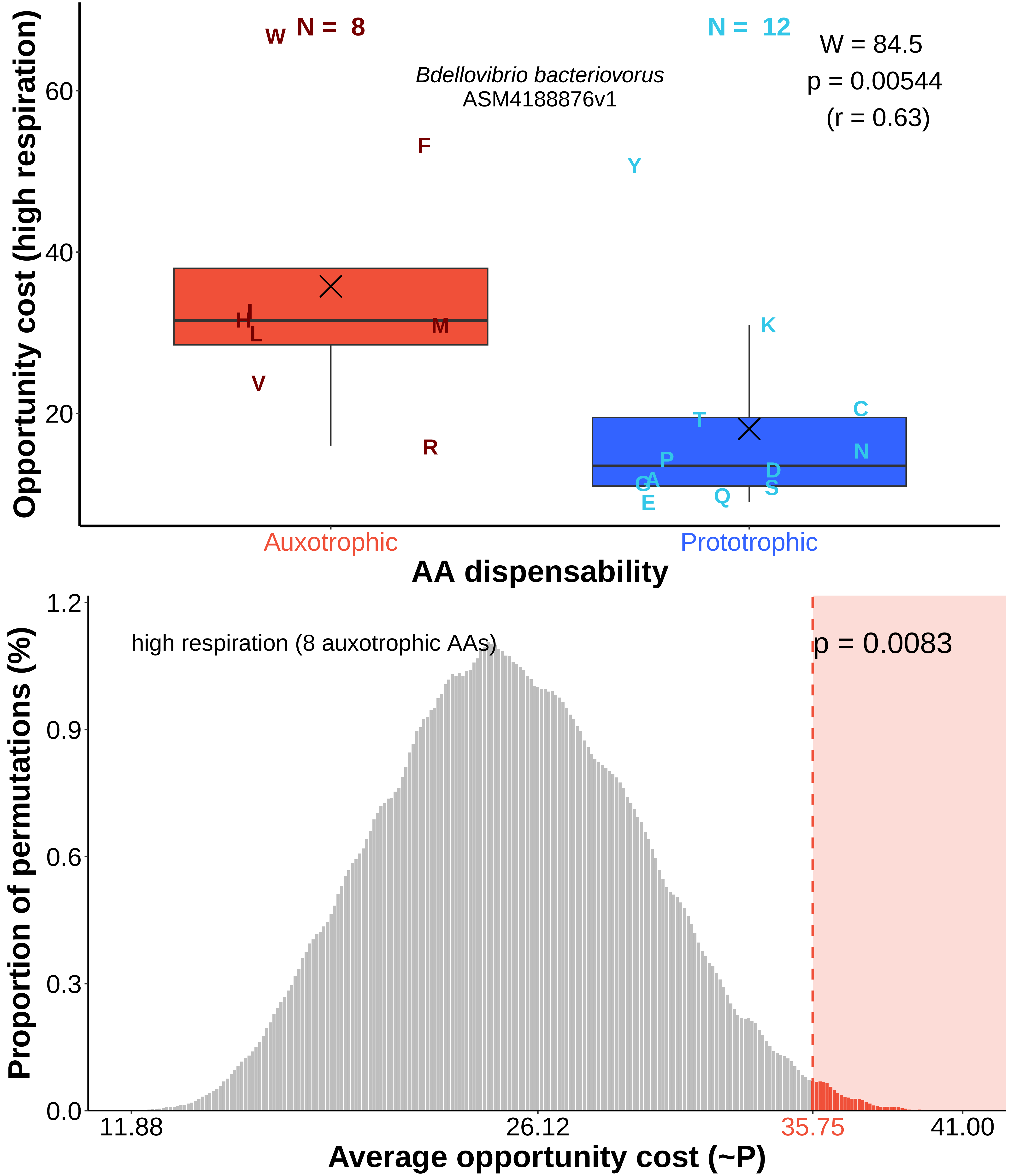

N = 8
N = 12
W = 84.5
p = 0.00544
(r = 0.63)
Bdellovibrio bacteriovorus
ASM4188876v1
Opportunity cost (high respiration)
Auxotrophic
Prototrophic
AA dispensability
high respiration (8 auxotrophic AAs)
p = 0.0083
Proportion of permutations (%)
11.88
26.12
35.75
41.00
Average opportunity cost (~P)

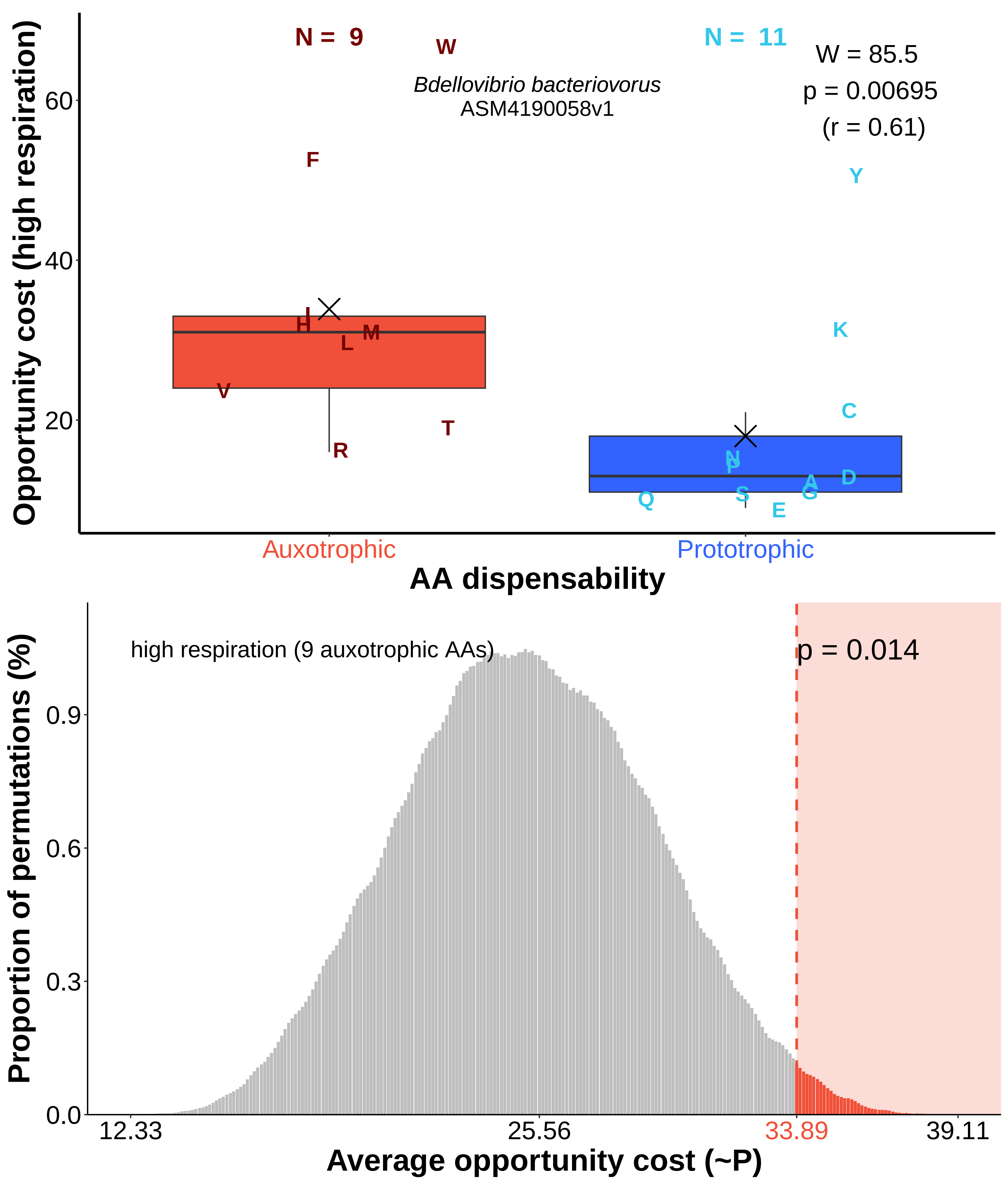

N = 9
N = 11
W = 85.5
p = 0.00695
(r = 0.61)
Bdellovibrio bacteriovorus
ASM4190058v1
Opportunity cost (high respiration)
60
40
20
W
F
I
H
M
L
V
T
R
Y
K
C
N
P
D
A
G
Q
S
E
Auxotrophic
Prototrophic
AA dispensability
high respiration (9 auxotrophic AAs)
p = 0.014
Proportion of permutations (%)
0.9
0.6
0.3
0.0
12.33
25.56
33.89
39.11
Average opportunity cost (~P)

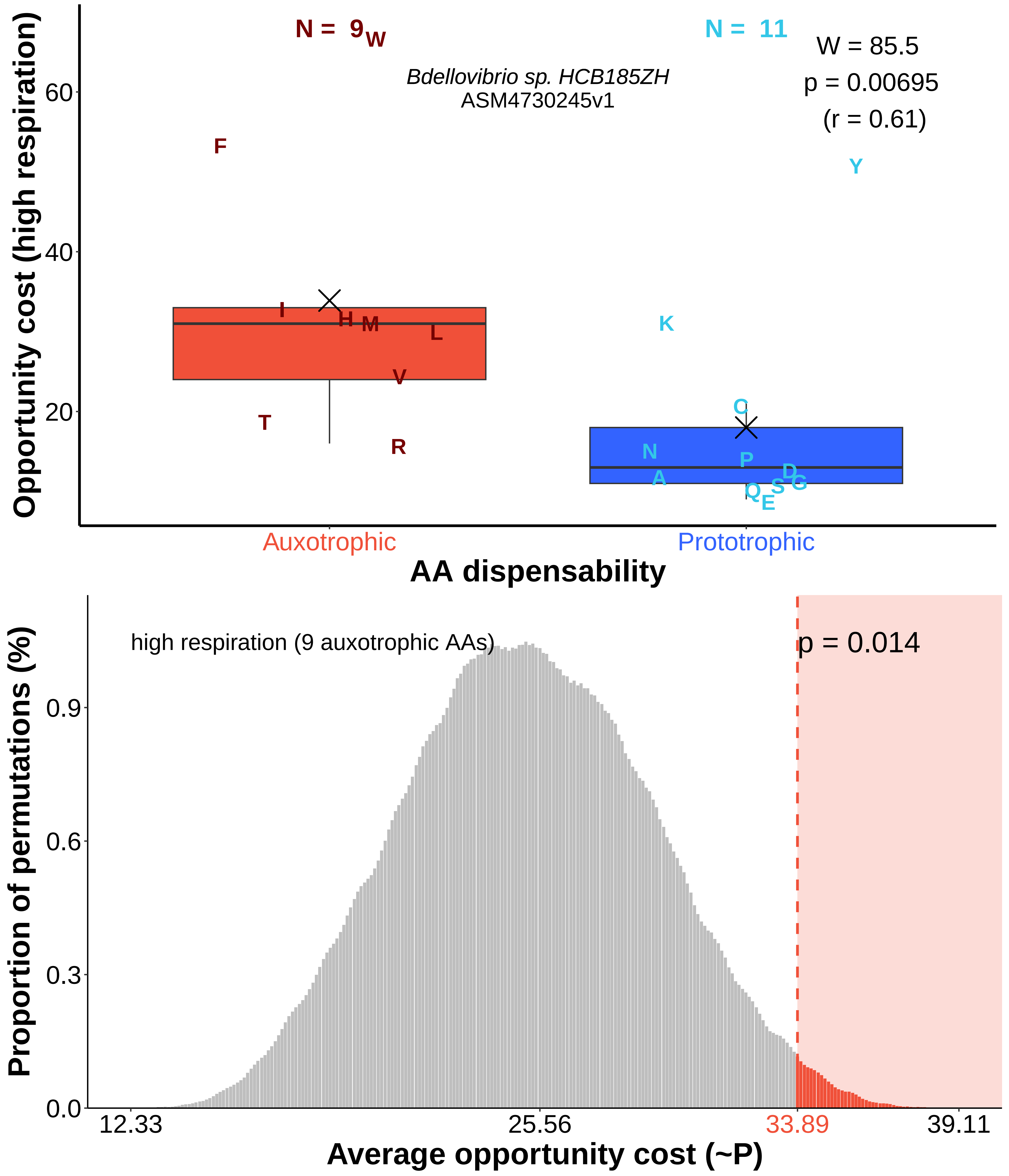

N = 9
N = 11
Bdellovibrio sp. HCB185ZH
ASM4730245v1
W = 85.5
p = 0.00695
(r = 0.61)
Opportunity cost (high respiration)
60
40
20
F
I
H
M
L
V
T
R
W
K
Y
C
N
P
A
D
G
S
Q
E
Auxotrophic
Prototrophic
AA dispensability
high respiration (9 auxotrophic AAs)
p = 0.014
Proportion of permutations (%)
0.9
0.6
0.3
0.0
12.33
25.56
33.89
39.11
Average opportunity cost (~P)

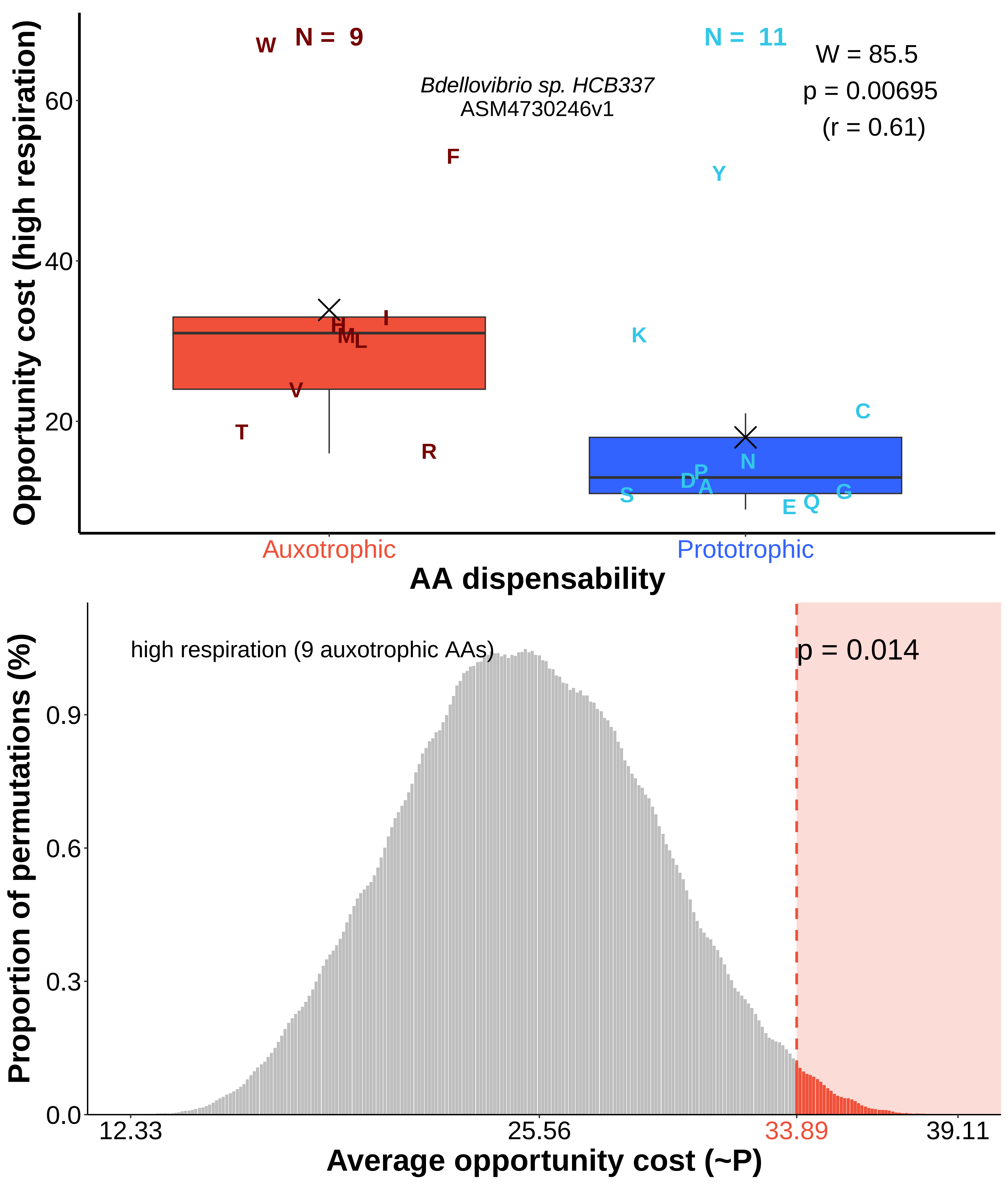

W
N = 9
N = 11
W = 85.5
p = 0.00695
(r = 0.61)
Bdellovibrio sp. HCB337
ASM4730246v1
F
Y
I
H
M
L
K
V
T
R
C
N
P
D
A
S
G
E
Q
60
40
20
Opportunity cost (high respiration)
Auxotrophic
Prototrophic
AA dispensability
high respiration (9 auxotrophic AAs)
p = 0.014
0.9
0.6
0.3
0.0
Proportion of permutations (%)
12.33
25.56
33.89
39.11
Average opportunity cost (~P)

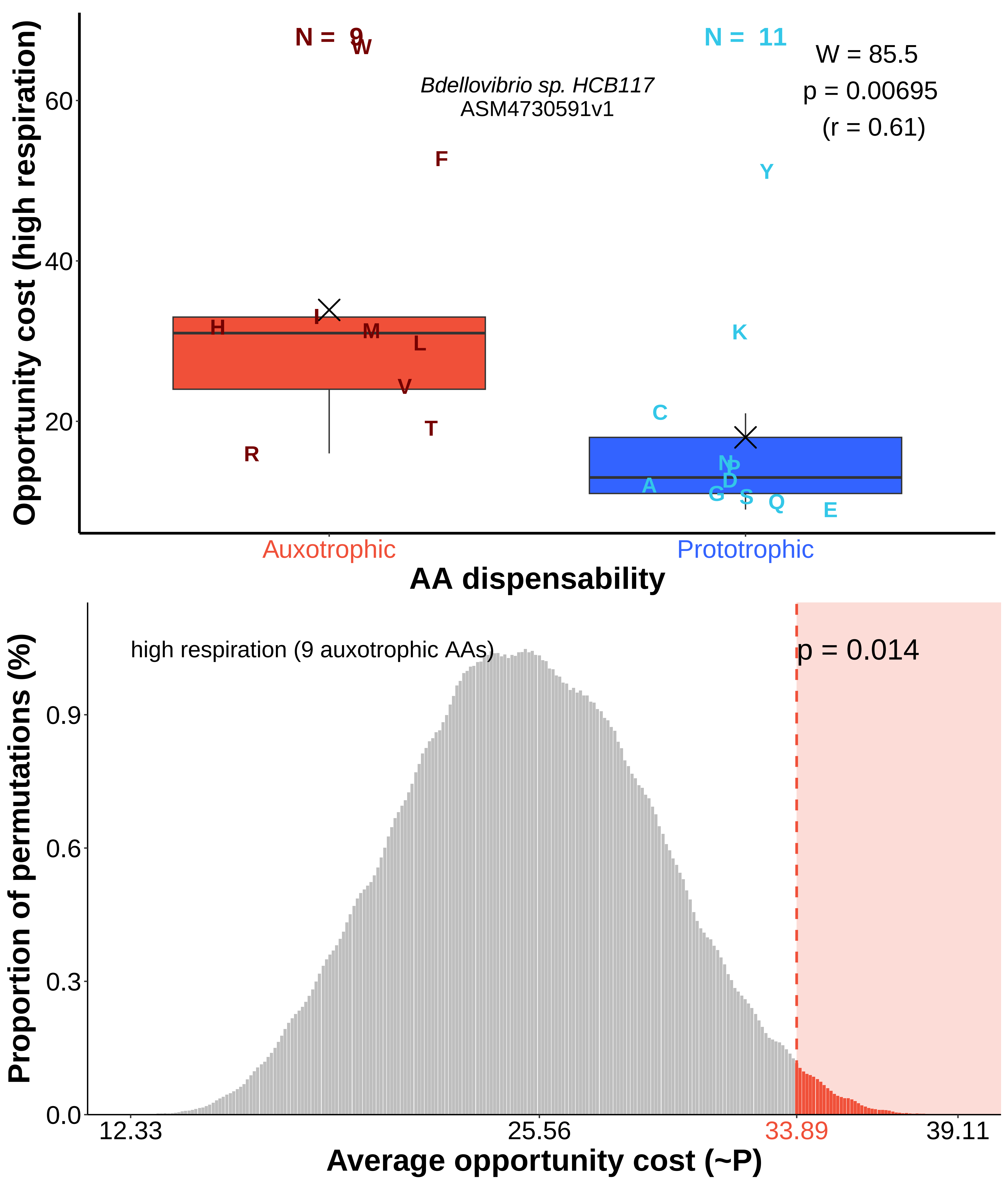
N = 9
N = 11
W = 85.5
p = 0.00695
(r = 0.61)
Bdellovibrio sp. HCB117
ASM4730591v1
Opportunity cost (high respiration)
Auxotrophic
Prototrophic
AA dispensability
high respiration (9 auxotrophic AAs)
p = 0.014
Proportion of permutations (%)
12.33
25.56
33.89
39.11
Average opportunity cost (~P)

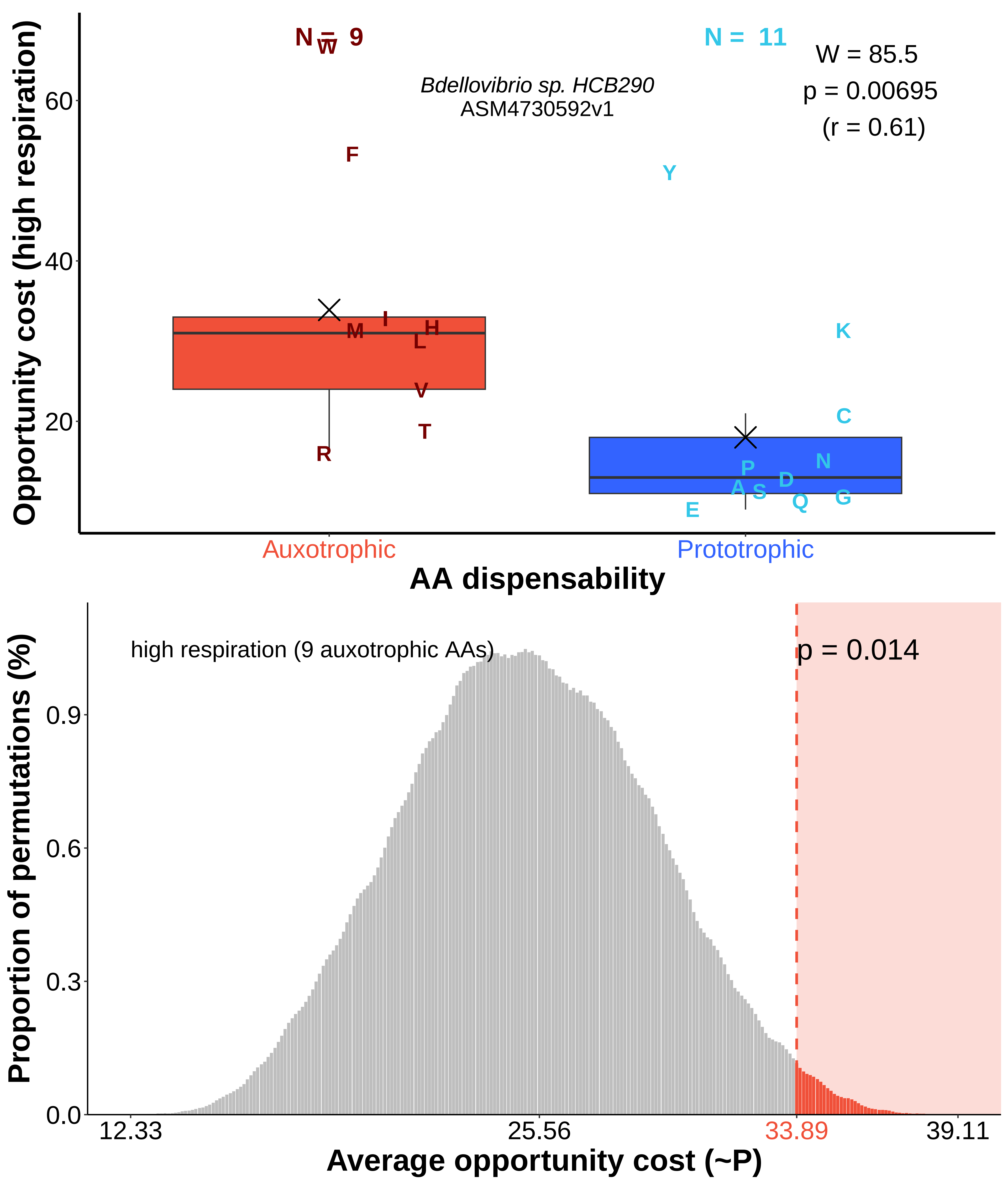
N = 9
N = 11
W = 85.5
p = 0.00695
(r = 0.61)
Bdellovibrio sp. HCB290
ASM4730592v1
F
W
Y
M
I
H
L
K
V
C
T
R
P
N
D
A
S
Q
G
E
Opportunity cost (high respiration)
60
40
20
Auxotrophic
Prototrophic
AA dispensability
high respiration (9 auxotrophic AAs)
p = 0.014
Proportion of permutations (%)
0.9
0.6
0.3
0.0
12.33
25.56
33.89
39.11
Average opportunity cost (~P)

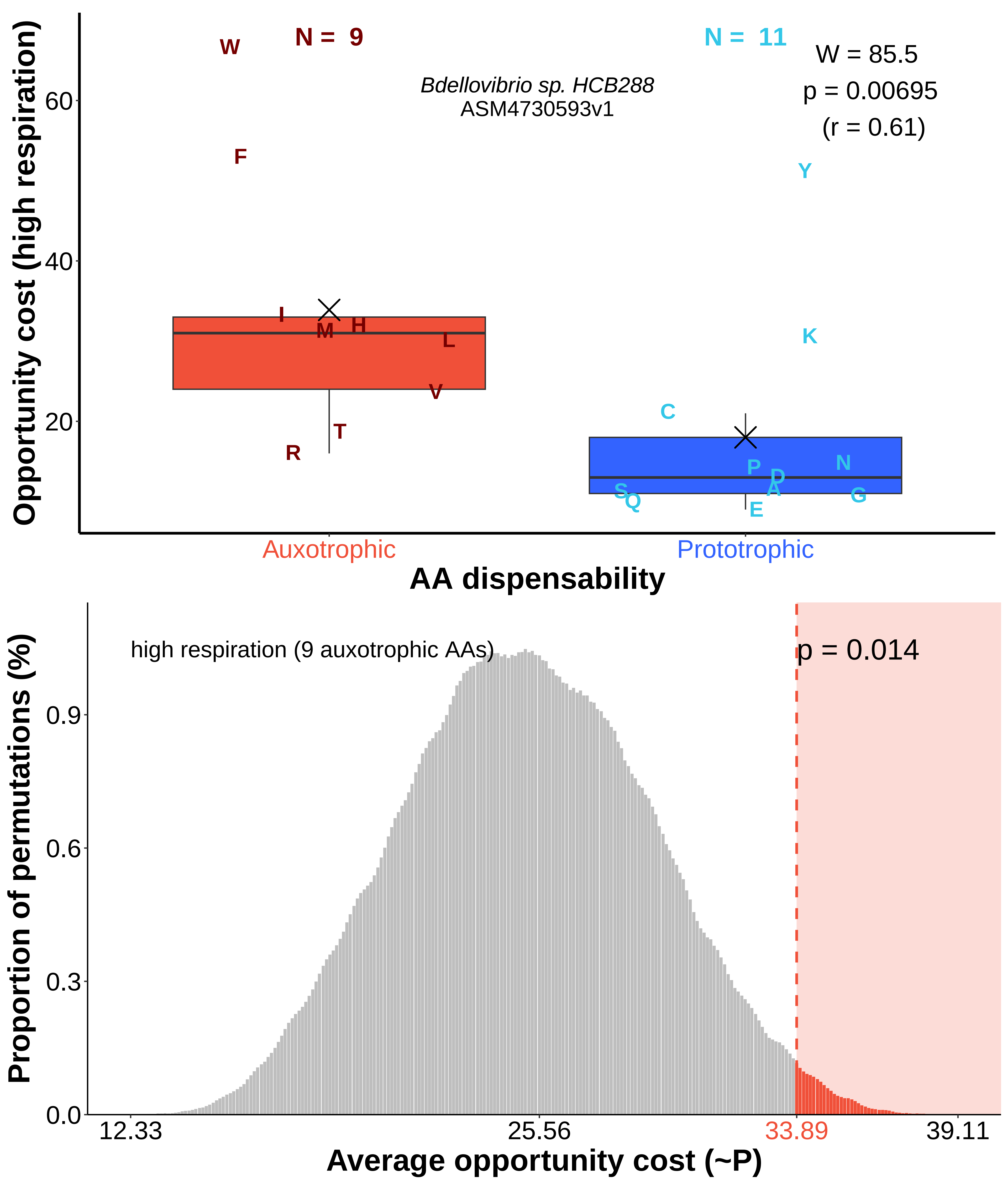

N = 9
N = 11
W = 85.5
p = 0.00695
(r = 0.61)
Bdellovibrio sp. HCB288
ASM4730593v1
Opportunity cost (high respiration)
60
40
20
W
F
I
M
H
L
V
T
R
Y
K
C
P
D
A
N
S
Q
G
E
Auxotrophic
Prototrophic
AA dispensability
high respiration (9 auxotrophic AAs)
p = 0.014
Proportion of permutations (%)
0.9
0.6
0.3
0.0
12.33
25.56
33.89
39.11
Average opportunity cost (~P)

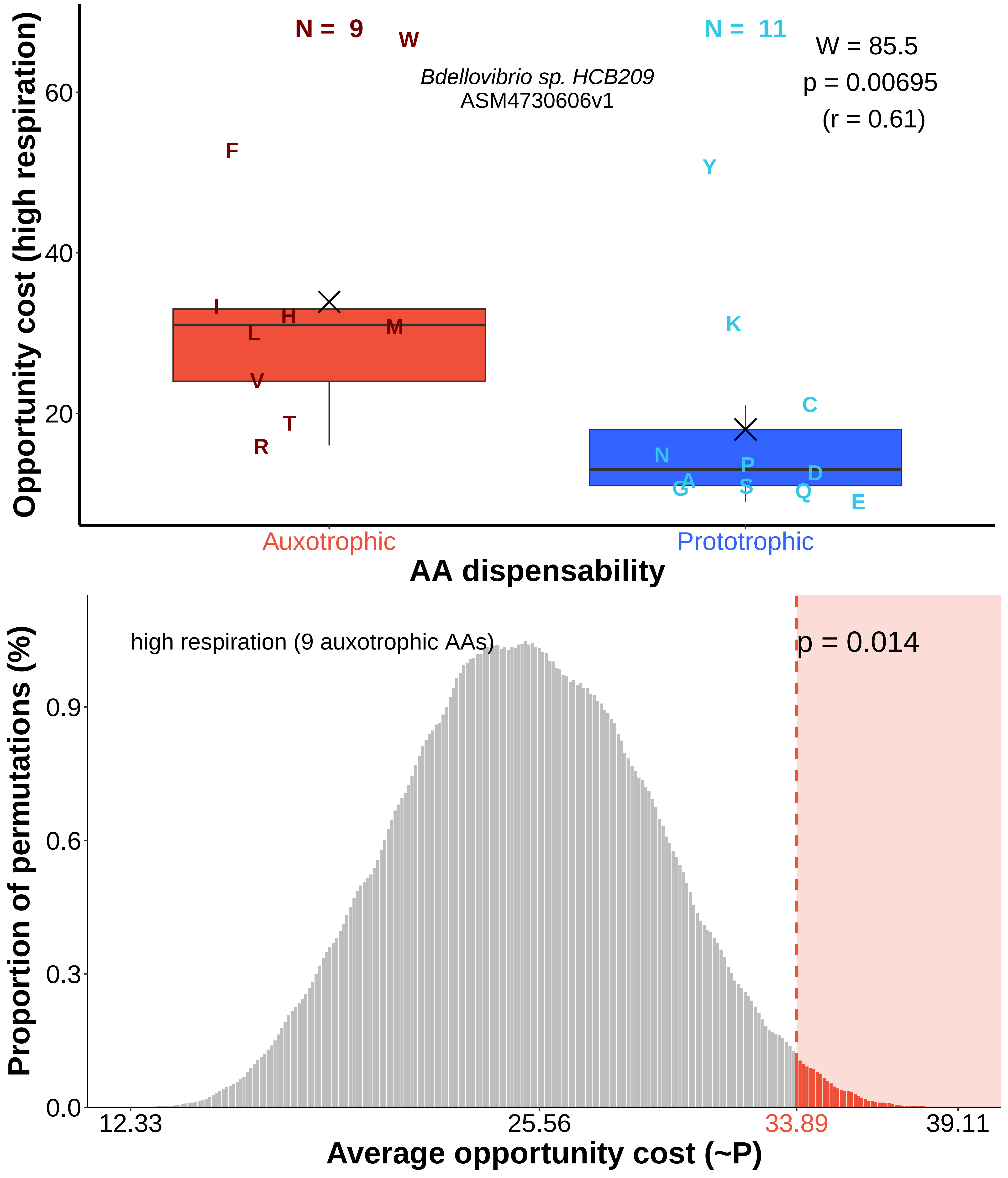

N = 9
N = 11
W = 85.5
p = 0.00695
(r = 0.61)
Bdellovibrio sp. HCB209
ASM4730606v1
Opportunity cost (high respiration)
Auxotrophic
Prototrophic
AA dispensability
high respiration (9 auxotrophic AAs)
p = 0.014
Proportion of permutations (%)
12.33
25.56
33.89
39.11
Average opportunity cost (~P)

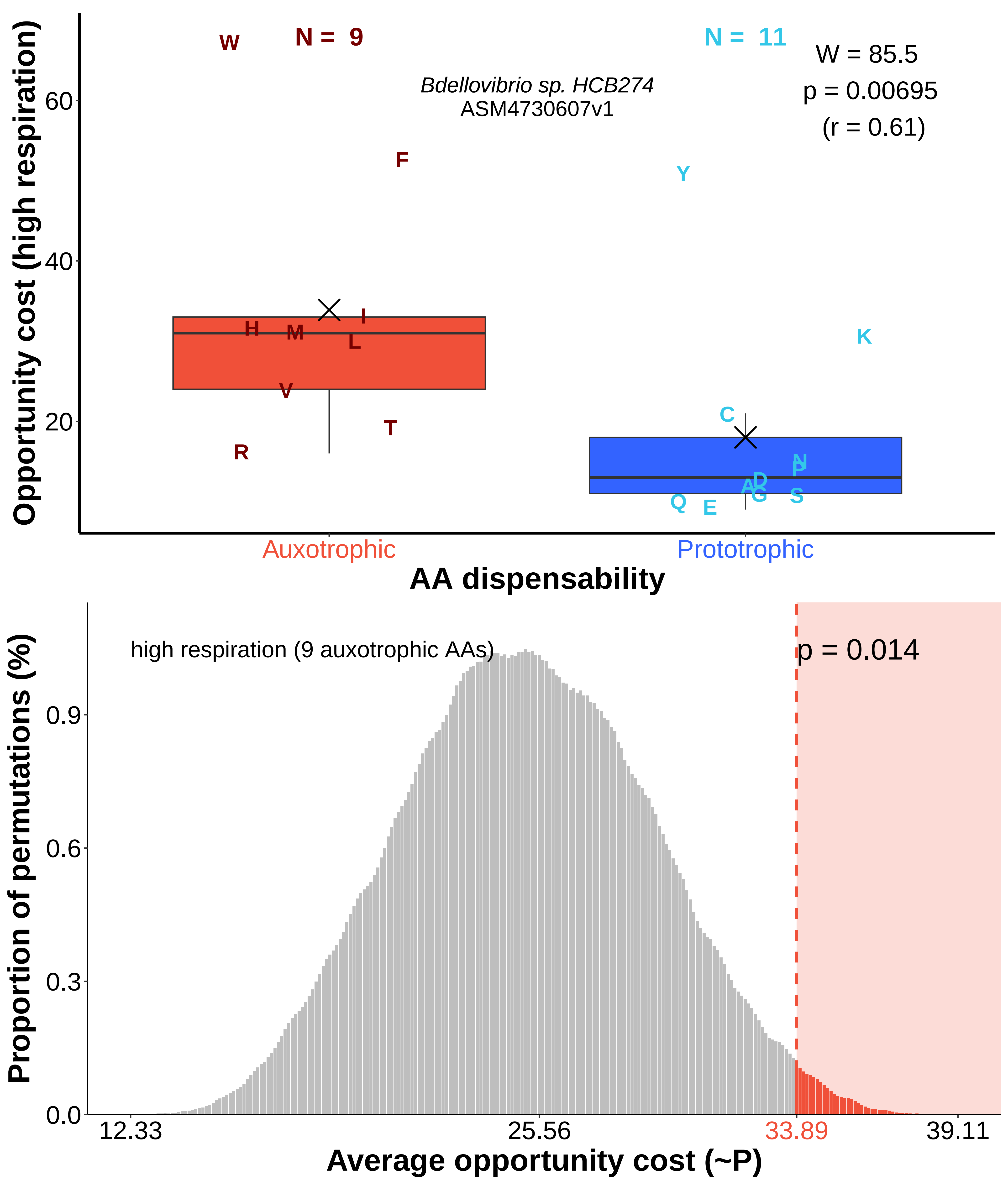
W
N = 9
N = 11
W = 85.5
p = 0.00695
(r = 0.61)
Bdellovibrio sp. HCB274
ASM4730607v1
F
Y
I
H
M
L
K
V
C
T
R
N
P
D
A
G
S
Q
E
Opportunity cost (high respiration)
60
40
20
Auxotrophic
Prototrophic
AA dispensability
high respiration (9 auxotrophic AAs)
p = 0.014
Proportion of permutations (%)
0.9
0.6
0.3
0.0
12.33
25.56
33.89
39.11
Average opportunity cost (~P)

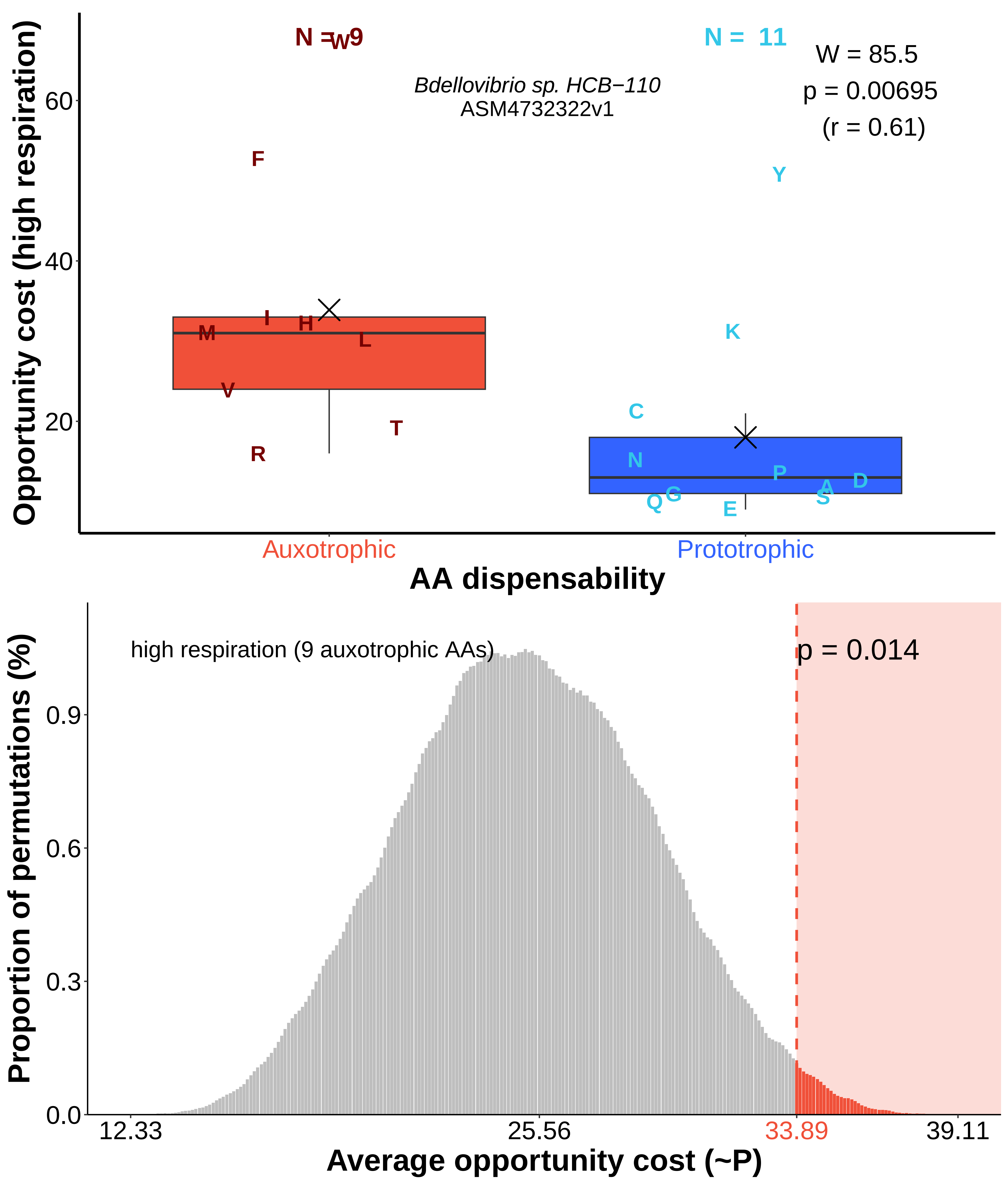

N = 9
W
N = 11
W = 85.5
p = 0.00695
(r = 0.61)
Bdellovibrio sp. HCB−110
ASM4732322v1
Opportunity cost (high respiration)
60
40
20
F
Y
I
H
M
L
K
V
C
T
R
N
P
D
A
S
Q
G
E
Auxotrophic
Prototrophic
AA dispensability
high respiration (9 auxotrophic AAs)
p = 0.014
Proportion of permutations (%)
0.9
0.6
0.3
0.0
12.33
25.56
33.89
39.11
Average opportunity cost (~P)

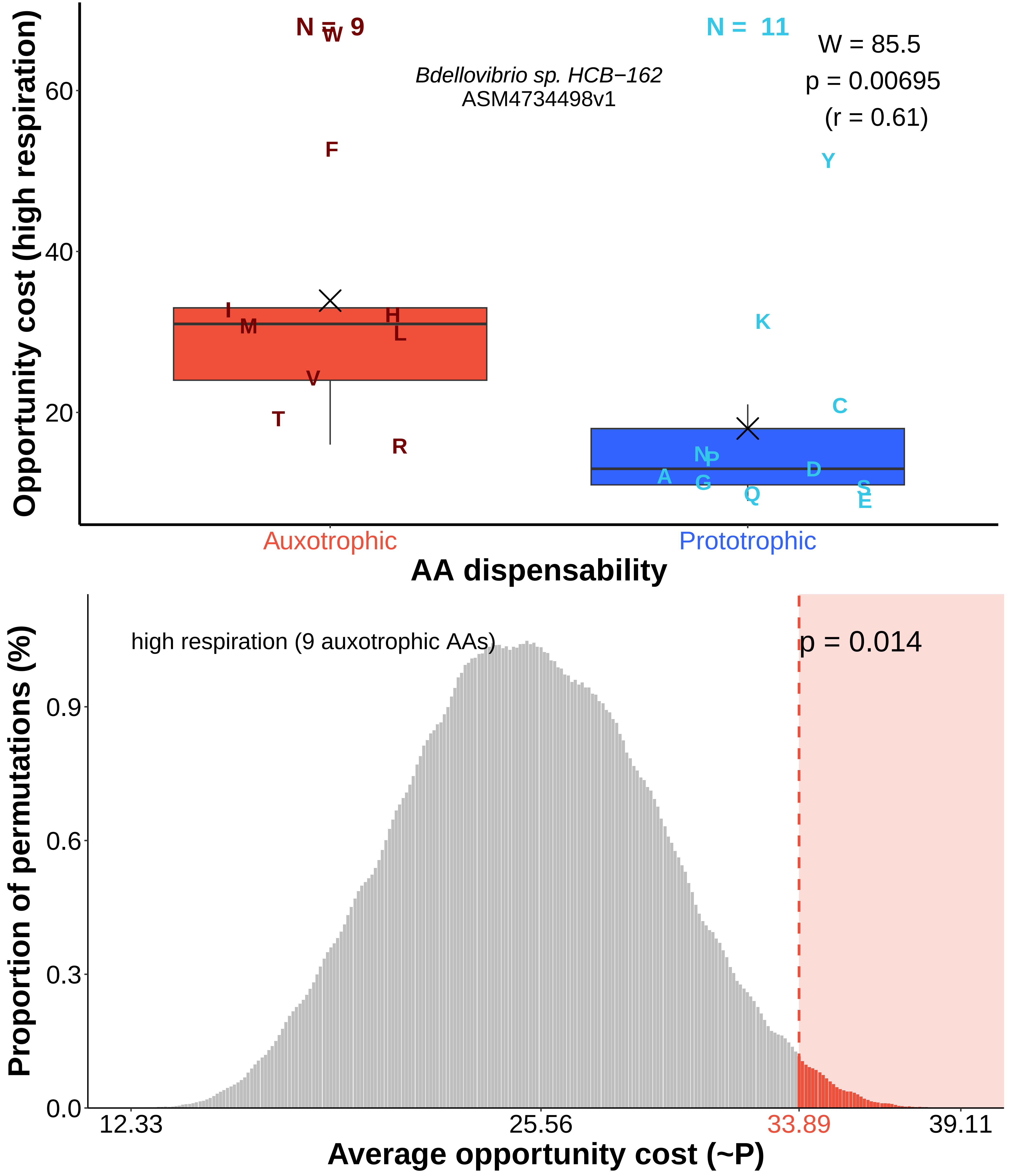

N = 9
N = 11
W = 85.5
p = 0.00695
(r = 0.61)
Bdellovibrio sp. HCB−162
ASM4734498v1
Opportunity cost (high respiration)
60
40
20
W
F
I
M
H
L
V
T
R
Y
K
C
N
P
D
A
G
Q
S
E
Auxotrophic
Prototrophic
AA dispensability
high respiration (9 auxotrophic AAs)
p = 0.014
Proportion of permutations (%)
0.9
0.6
0.3
0.0
12.33
25.56
33.89
39.11
Average opportunity cost (~P)

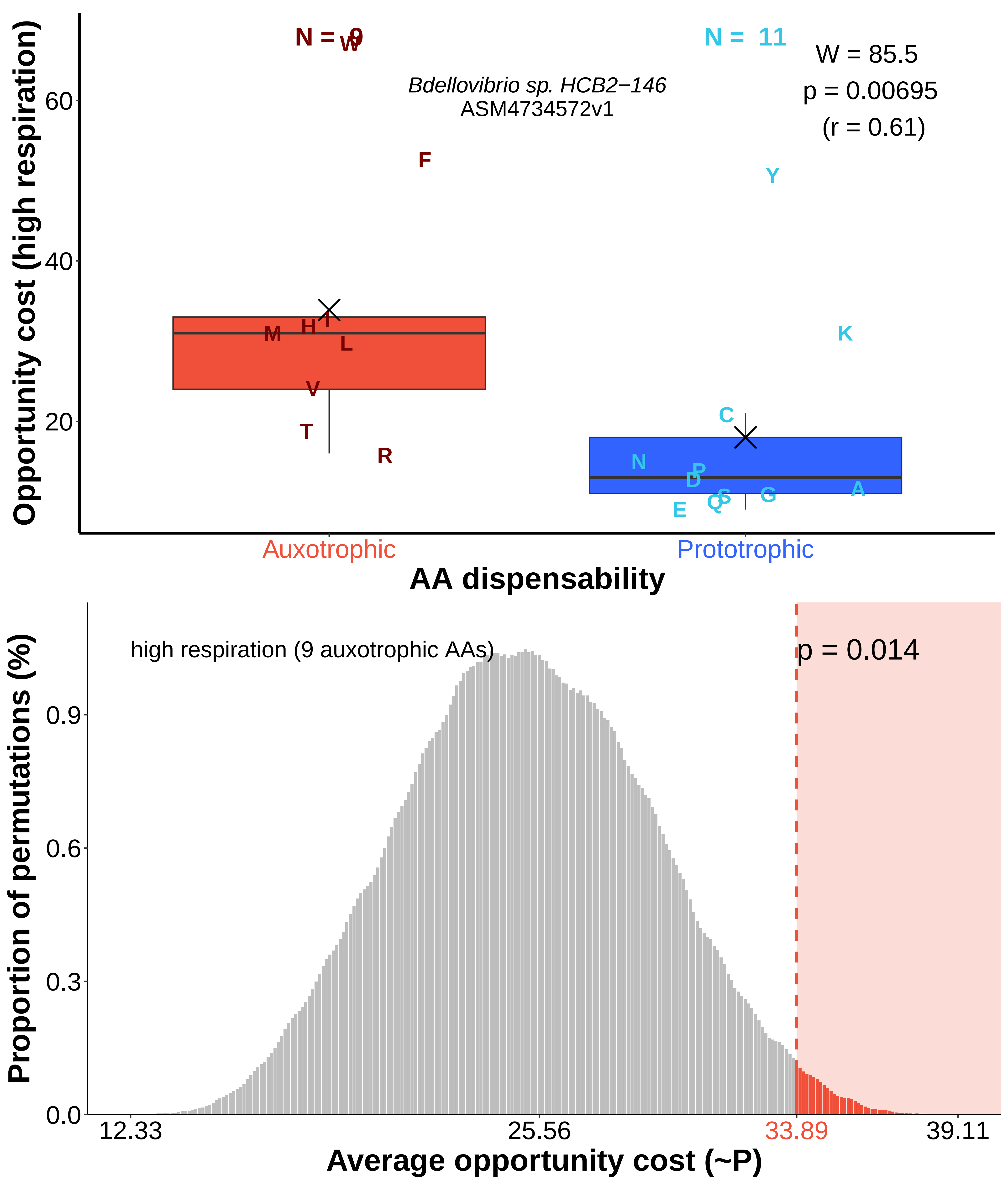
N = 9
N = 11
W = 85.5
p = 0.00695
(r = 0.61)
Bdellovibrio sp. HCB2−146
ASM4734572v1
Opportunity cost (high respiration)
Auxotrophic
Prototrophic
AA dispensability
high respiration (9 auxotrophic AAs)
p = 0.014
Proportion of permutations (%)
Average opportunity cost (~P)
12.33
25.56
33.89
39.11

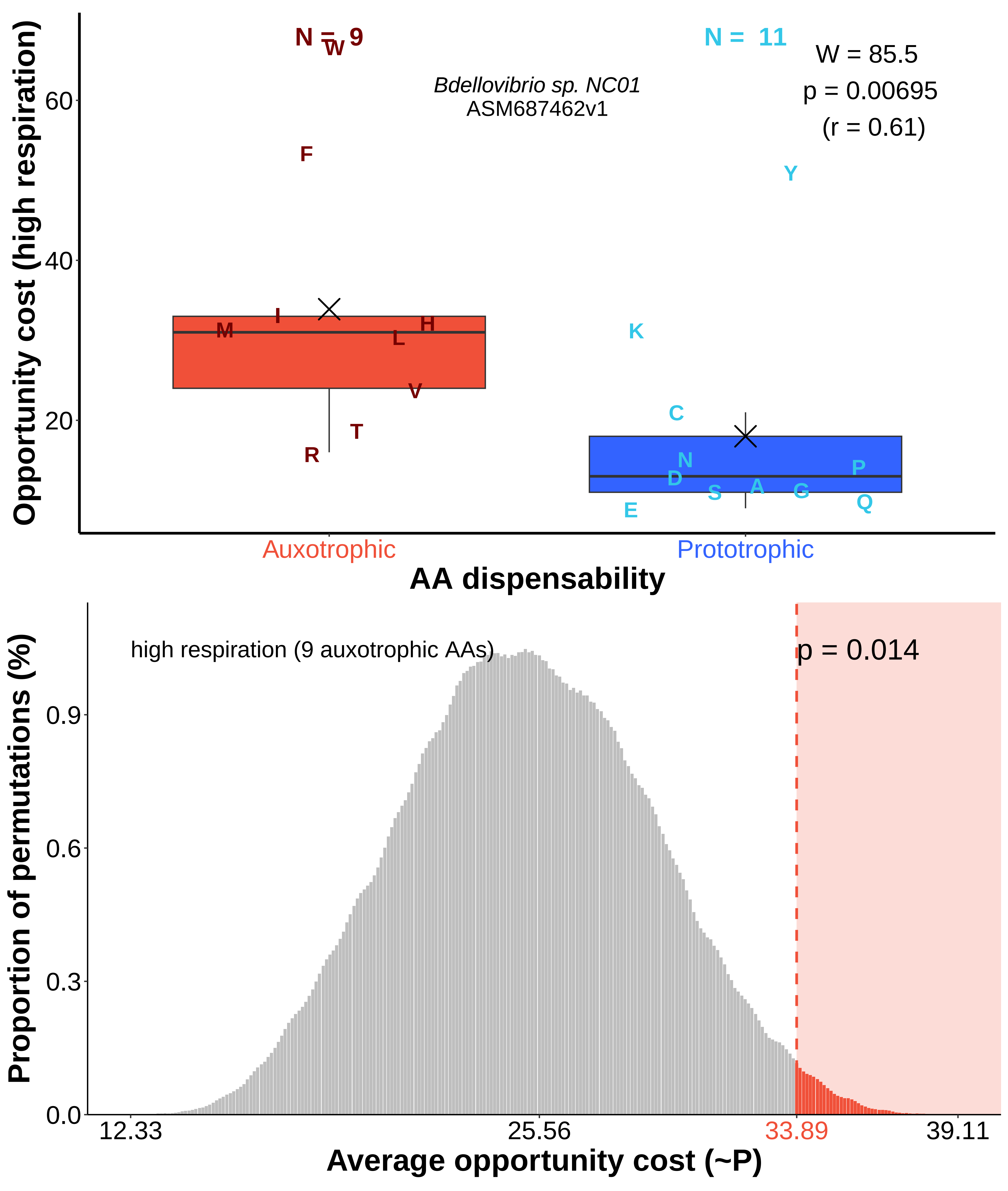
N = 9
N = 11
W = 85.5
p = 0.00695
(r = 0.61)
Bdellovibrio sp. NC01
ASM687462v1
Opportunity cost (high respiration)
60
40
20
W
F
I
M
L
H
V
T
R
Y
K
C
N
D
S
A
G
P
Q
E
Auxotrophic
Prototrophic
AA dispensability
high respiration (9 auxotrophic AAs)
p = 0.014
Proportion of permutations (%)
0.9
0.6
0.3
0.0
12.33
25.56
33.89
39.11
Average opportunity cost (~P)

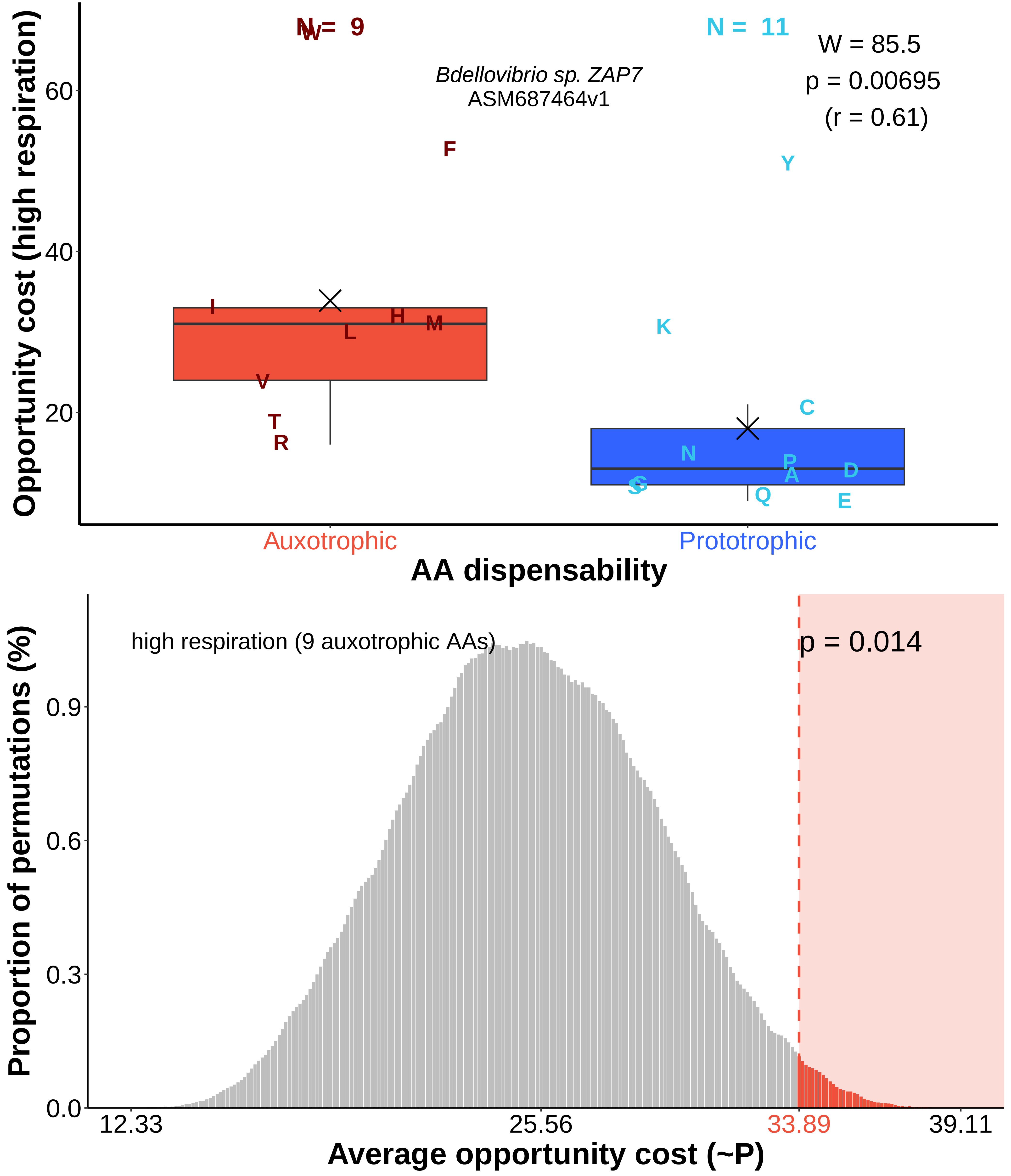
N = 9
N = 11
W = 85.5
p = 0.00695
(r = 0.61)
Bdellovibrio sp. ZAP7
ASM687464v1
Opportunity cost (high respiration)
60
40
20
F
Y
I
H
M
L
V
T
R
K
C
N
P
A
D
S
G
Q
E
W
Auxotrophic
Prototrophic
AA dispensability
Proportion of permutations (%)
high respiration (9 auxotrophic AAs)
p = 0.014
0.9
0.6
0.3
0.0
12.33
25.56
33.89
39.11
Average opportunity cost (~P)

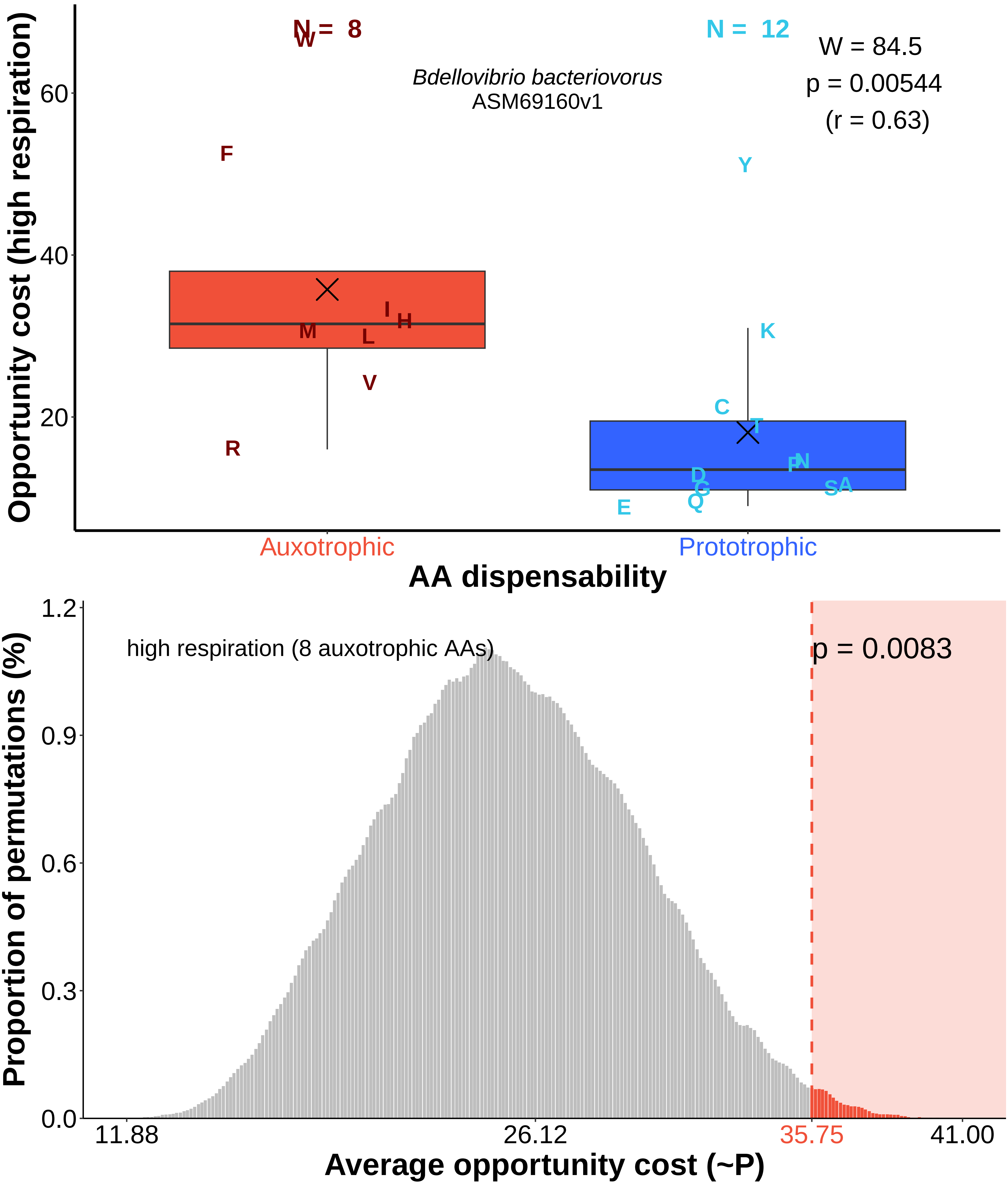

N = 8
W
N = 12
W = 84.5
p = 0.00544
(r = 0.63)
Bdellovibrio bacteriovorus
ASM69160v1
F
Y
I
H
M
L
K
V
C
T
R
D
G
P
N
S
A
Q
E
60
40
20
Opportunity cost (high respiration)
Auxotrophic
Prototrophic
AA dispensability
high respiration (8 auxotrophic AAs)
p = 0.0083
1.2
0.9
0.6
0.3
0.0
Proportion of permutations (%)
11.88
26.12
35.75
41.00
Average opportunity cost (~P)

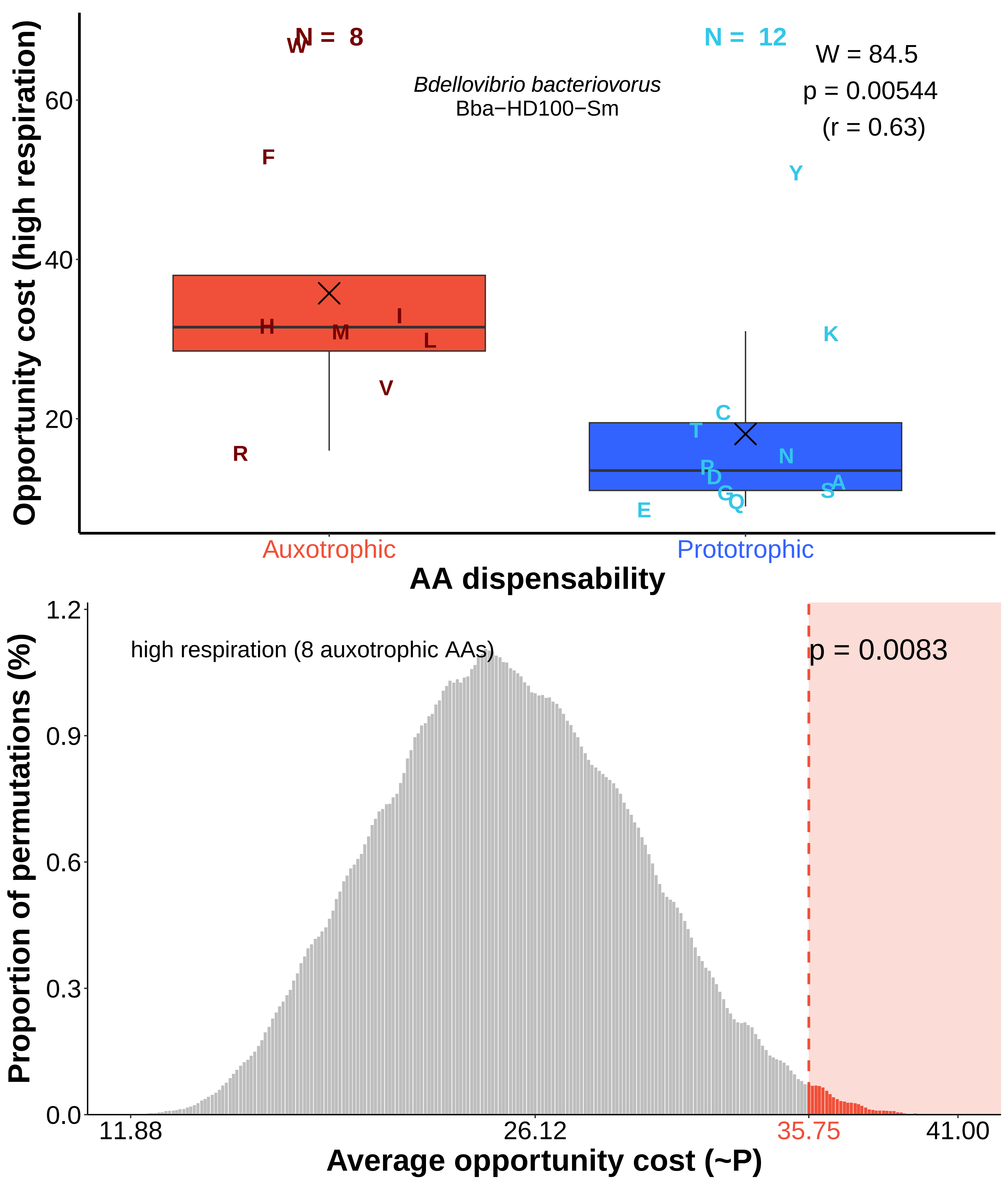

N = 8
N = 12
W = 84.5
p = 0.00544
(r = 0.63)
Bdellovibrio bacteriovorus
Bba−HD100−Sm
Opportunity cost (high respiration)
60
40
20
W
F
H
M
I
L
V
R
Y
K
C
T
N
P
D
G
Q
S
A
E
Auxotrophic
Prototrophic
AA dispensability
Proportion of permutations (%)
1.2
0.9
0.6
0.3
0.0
high respiration (8 auxotrophic AAs)
p = 0.0083
11.88
26.12
35.75
41.00
Average opportunity cost (~P)
